# Solvent-Controlled Regiodivergent Friedel–Crafts Reactions of 1‑Naphthol with In Situ Generated Aza‑*o*‑Quinone Methides

**DOI:** 10.1021/acs.joc.5c01250

**Published:** 2025-07-18

**Authors:** Si-Kai Liu, Salha Alotaibi, Jen-Yu Kuan, Theo P. Gonçalves, Kuo-Wei Huang, Jeng-Liang Han

**Affiliations:** † Department of Chemistry, 34916 National Chung Hsing University, Taichung City 40227, Taiwan; ‡ Physical Science and Engineering Division and Center for Renewable Energy and Storage Technologies, 127355 King Abdullah University of Science and Technology, Thuwal 23955-6900, Saudi Arabia

## Abstract

In this study, we reported a solvent-controlled site-selectivity switchable Friedel–Crafts reaction of 1-naphthols with in situ generated aza-*o*-quinone methides. The *ortho*-selective Friedel–Crafts reaction was achieved in toluene, while the *para*-selective Friedel–Crafts reaction was accomplished in acetonitrile. With this protocol, a range of functionalized triarylmethanes were prepared. Moreover, theoretical mechanistic investigations provided insights into the site-selective reaction pathway.

## Graphical abstract

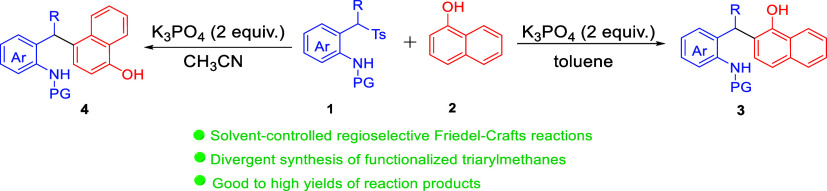

## Introduction

Achieving switchable regioselectivity of substrates with multiple reaction sites is still challenging, especially when aiming to produce complex structures from identical starting materials.^1,2^ The regioselective switch of side reactions can be controlled by the proper choice of various reaction conditions, such as catalysts, ligands, solvents, temperatures, and additives.^3–7^ Therefore, the development of efficient synthetic methodologies for regiodivergent reactions is still highly demanded.

Aza-*o*-quinone methides (N-*o*-QMs), the nitrogen-based analogs of *o*-quinone methides (*o*-QMs), are versatile and highly reactive intermediates for the synthesis of useful nitrogenous compounds^8^ through various reactions such as 1,4-conjugate additions^9^ and cycloaddition reactions.^10^ However, due to the high reactivity of aza-*o*-QMs,^11^ only limited stable and readily available aza-*o*-QM precursors can be applied to organic synthesis. Accordingly, the development of mild and efficient approaches for aza-*o*-QM generation and subsequent new reaction systems would be highly in demand (Scheme 1).

**Scheme 1:**
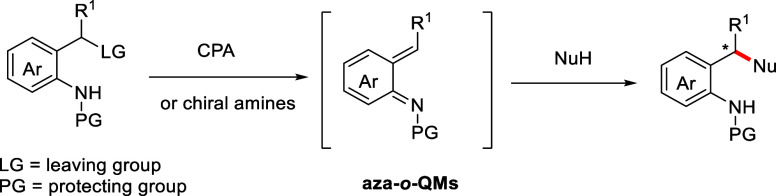
Catalytic Asymmetric 1,4-Conjugate Addition Reactions of Aza-*o*-QMs

1-Naphthols, crucial aromatic feedstocks,^12^ have found applications in the *ortho*-selective Friedel–Crafts alkylation when employing basic conditions.^13^ However, the *para*-selective organocatalytic enantioselective Friedel–Crafts alkylation of 1-naphthols remains uncommon. Typically, these regioselective reactions at the C4-position are catalyzed by acidic conditions.^14^ An intriguing development was reported by Li and co-workers, who achieved site-selectivity switchable enantioselective Friedel–Crafts reactions of unprotected 1-naphthols with 1-azadienes by two chiral complementary squaramide and phosphoric acid.^14b^ We recently developed an organocatalyst-controlled site-selectivity switchable Friedel–Crafts reaction of 1-naphthols and 2,3-dioxopyrrolidines. The *o*-selective Friedel–Crafts reaction was achieved with chiral tertiary amines, while the *p*-selective Friedel–Crafts reaction was accomplished by Bro̷nsted acids or Lewis acids.^14e^ However, to the best of our knowledge, the switchable site-selective Friedel–Crafts reaction of 1-naphthols controlled by solvents has not been reported. Therefore, continuing our efforts to develop organocatalytic asymmetric Friedel–Crafts reactions,^14e,15^ we herein report a base-mediated and solvent-controlled site-selectivity switchable Friedel–Crafts reaction of 1-naphthol with aza-*o*-QMs, affording the triarylmethanes in moderate to high reaction yields (Scheme 2).

**Scheme 2:**
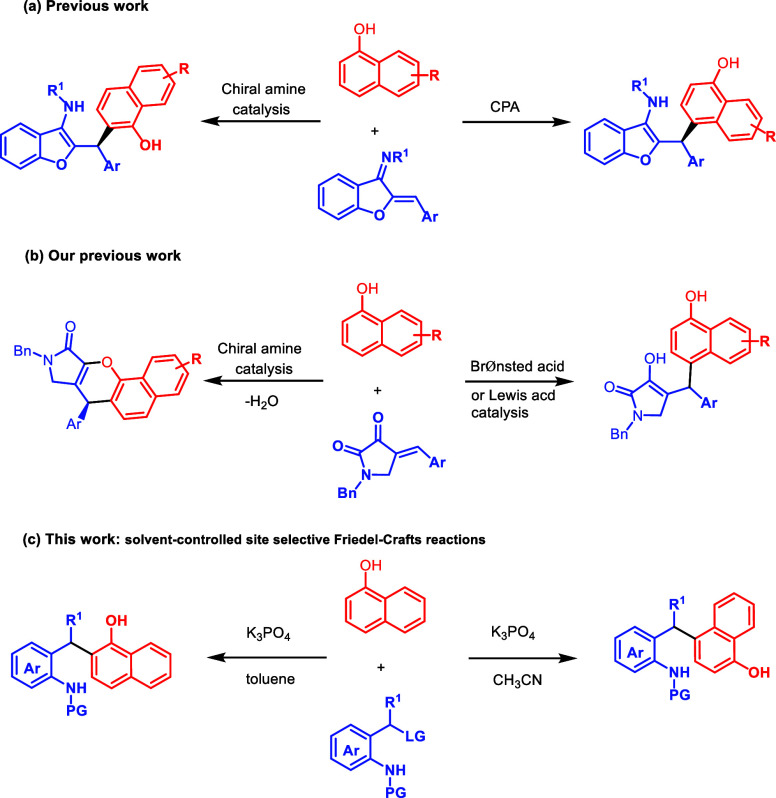
Site-Selective Friedel–Crafts Reaction of 1-Naphthol with Aza-*o*-QMs

## Results and Discussion

A model reaction of 2-(tosylmethyl)­aniline **1a** with 1-naphthol **2** (1.5 equiv) using Cs_2_CO_3_ (1.2 equiv) as the inorganic base was first studied in various solvents (Table 1). Notably, reactions in less polar solvents, such as toluene, mesitylene, *o*-xylene, bromobenzene, and CH_2_Cl_2_, led to the *ortho*-substituted product **3a** as the major product (Table 1, entries 1–5), with the highest product yield observed in toluene (Table 1, entry 3). In contrast, the *para*-substituted compound **4a** was obtained as the major product when the reactions were performed in polar solvents (Table 1, entries 6–11), with the highest product yield observed in CH_3_CN (55% yield, entry 10).

**Table 1:**
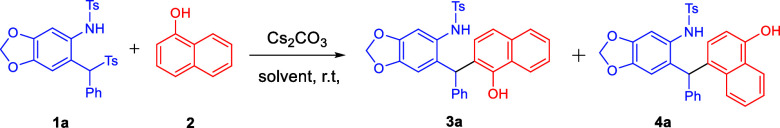
Solvent Screening^a^

entry	solvent	time (h)	yield of **3a** (%)^b^	yield of **4a** (%)^b^
1	mesitylene	144	23	13
2	bromobenzene	72	33	17
3	toluene	72	39	30
4	*o*-xylene	72	29	21
5	CH_2_Cl_2_	72	35	28
6	1,2-DCE	72	27	30
7	THF	72	34	41
8	EA	168	33	35
9	CHCl_3_	72	23	25
10	CH_3_CN	48	trace	55
11	DMF	72	trace	54

a Unless otherwise noted, the reaction was carried out by using 0.10 mmol of **1a**, 0.15 mmol of **2**, and 0.12 mmol of Cs_2_CO_3_ in 2.0 mL of solvent at r.t. (25–28 °C) for the indicated time.

b Isolated yields.

With the optimum solvent identified (toluene), other reaction parameters were investigated. As shown in Table 2, the reaction performed with other inorganic bases and K_3_PO_4_ gave the highest product yield (76%) (Table 2, entries 1–5). No reactivity was observed when organic bases were used (Table 2, entries 6–8). Examination of the reaction temperature did not improve the outcomes (Table 2, entries 9 and 10). Increasing the amounts of base to 2.0 equiv led to better product yield (80%) (Table 2, entries 11 and 12). Hence, the optimal conditions were finally chosen by conducting the reaction at room temperature in 2.0 mL of toluene with 2.0 equiv of K_3_PO_4_ for 72 h (entry 11). Notably, when the solvent was changed to CH_3_CN, **4a** was obtained as the major product, and the product yield increased to 72% (Table 2, entry 13).

**Table 2:** Optimization of Reaction Conditions^a^

entry	base	time (h)	yield of **3a** (%)^b^	yield of **4a** (%)^b^
1	K_2_CO_3_	96	31	16
2	Na_2_CO_3_	168	41	7
3	K_3_PO_4_	72	60	16
4	NaHCO_3_	120	trace	trace
5	NaOH	72	31	13
6	Et_3_N	72	trace	trace
7	DABCO	72	trace	trace
8	DBU	72	trace	trace
9^c^	K_3_PO_4_	168	trace	trace
10^d^	K_3_PO_4_	72	51	13
11^e^	K_3_PO_4_	72	62	18
12^f^	K_3_PO_4_	72	37	14
13^g^	K_3_PO_4_	72	14	58

a Unless otherwise noted, the reaction was carried out by using 0.10 mmol of **1a**, 0.15 mmol of **2**, and 0.12 mmol of bases in 2.0 mL of toluene at r.t. (25–28 °C) for indicated time.

b Isolated yields.

c At 0 °C.

d At 40 °C.

e 0.20 mmol of K_3_PO_4_.

f 0.30 mmol of K_3_PO_4_.

g Solvent = CH_3_CN (2.0 mL).

After our optimized reaction conditions were established, the scope of the *ortho*-selective Friedel–Crafts reaction was then evaluated by reacting a series of 2-(tosylmethyl)­anilines **1** with 1-naphthol **2** (Scheme 3).

**Scheme 3:**
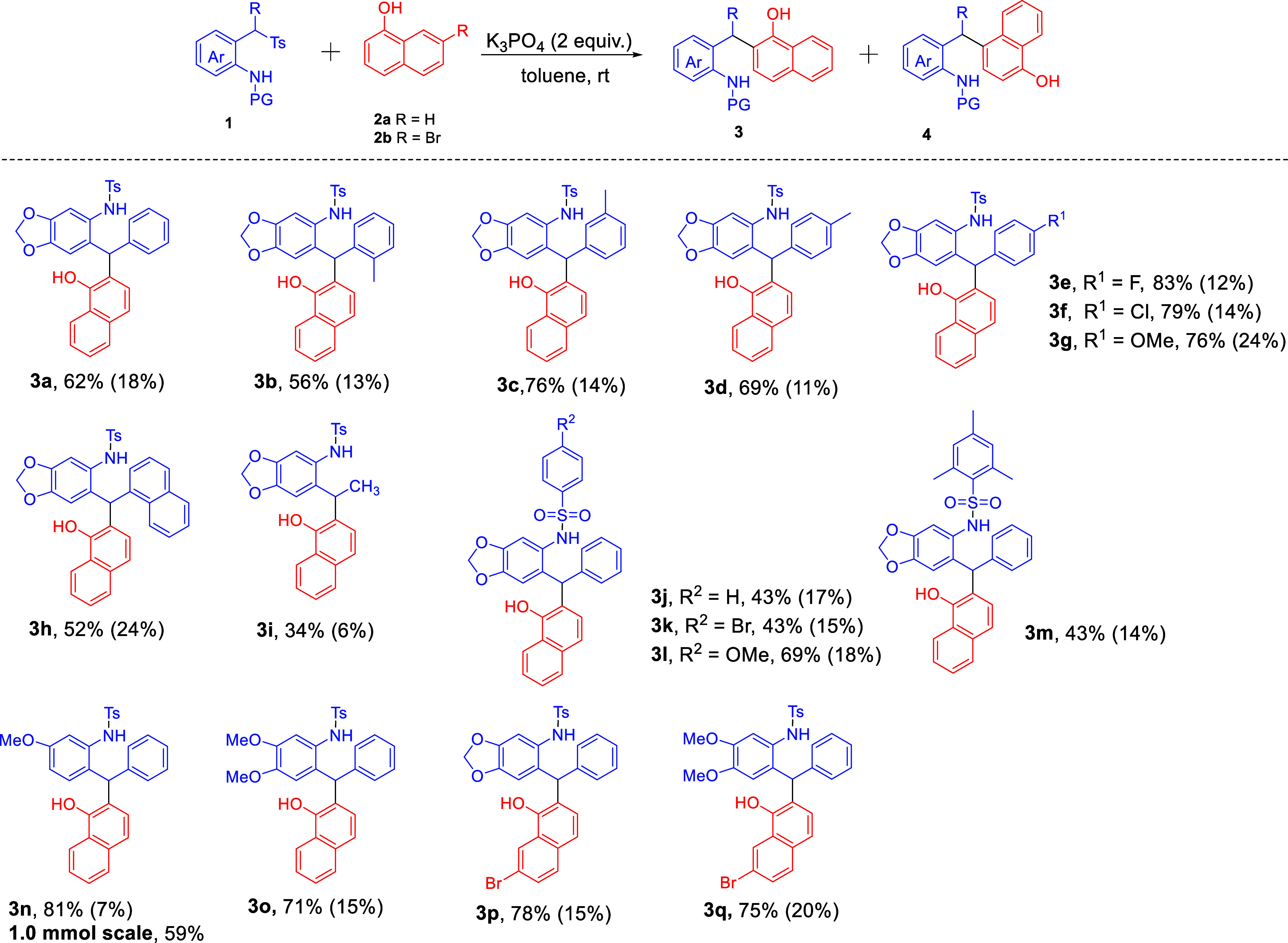
Scope of the *ortho*-Selective Friedel–Crafts Reaction^a^,^b^,^c^

a Unless otherwise noted, the solution of 0.1 mmol of **1**, 0.15 mmol of **2**, and 0.20 mmol of K_3_PO_4_ in 2.0 mL of toluene was stirred at r.t. (25–28 °C) for 72 h.
b Isolated yields.
c The values in the parentheses are *para*-substituted products **4**.

In addition to 2-(tosylmethyl)­anilines (**1b–g**), bearing electron-rich as well as electron-deficient substituents on different positions of the aryl ring, 2-naphthyl-substituted 2-(tosylmethyl)­aniline (**1h**) was also tolerated and afforded the corresponding *ortho*-substituted products (**3b**–**h**) in moderate to good yield. Only in the case of methyl-substituted 2-(tosylmethyl)­aniline (**1i**), product (**3i**) was obtained in low yield. It is possible that the in situ generated aza-*o*-QM was unstable due to the lack of additional phenyl resonance stabilization. The presence of other substituents on the sulfonamide of the 2-(tosylmethyl)­anilines **1j–m** could not improve the reactivity and provided the products (**3j–m**) in moderate yield. Replacement of the dioxolane moiety on the aniline ring of the newly formed *ortho*-substituted product **3** is possible through the choice of anilines **1**. This feature is demonstrated with monomethoxyaniline **1n** and dimethoxyaniline **1o**, which gave rise to the corresponding *ortho*-substituted products **3n** and **3o**, respectively, with good to high product yields. 1-Naphthol with a Br substituent (**2b**) worked well in this reaction, and the desired products **3p** and **3q** were delivered with 78 and 75% yields, respectively.

A larger-scale reaction (1.0 mmol) of **1n** could smoothly take place to give product **3n** with a slightly decreased yield (59%). It is possible that the in situ generated aza-*o*-QM decomposed because many side products were found.

Having showcased the scope of *ortho*-selective Friedel–Crafts alkylation, we next turned our attention to the scope of *para*-selective Friedel–Crafts alkylation performed in acetonitrile. As shown in Scheme 4, the 2-(tosylmethyl)­anilines with different electron-donating, electron-withdrawing, 2-naphthyl, and sulfonyl groups worked well with 1-naphthol (**2a**), obtaining the corresponding products **4a–** **4o** in 45–63% isolated yields. 1-Naphthol (**2b**) worked well in this reaction, and the desired products **4p** and **4q** were delivered with moderate yields. A larger-scale reaction (0.7 mmol) of **1m** could smoothly take place to give product **4m** in lower yield (40%).

**Scheme 4:**
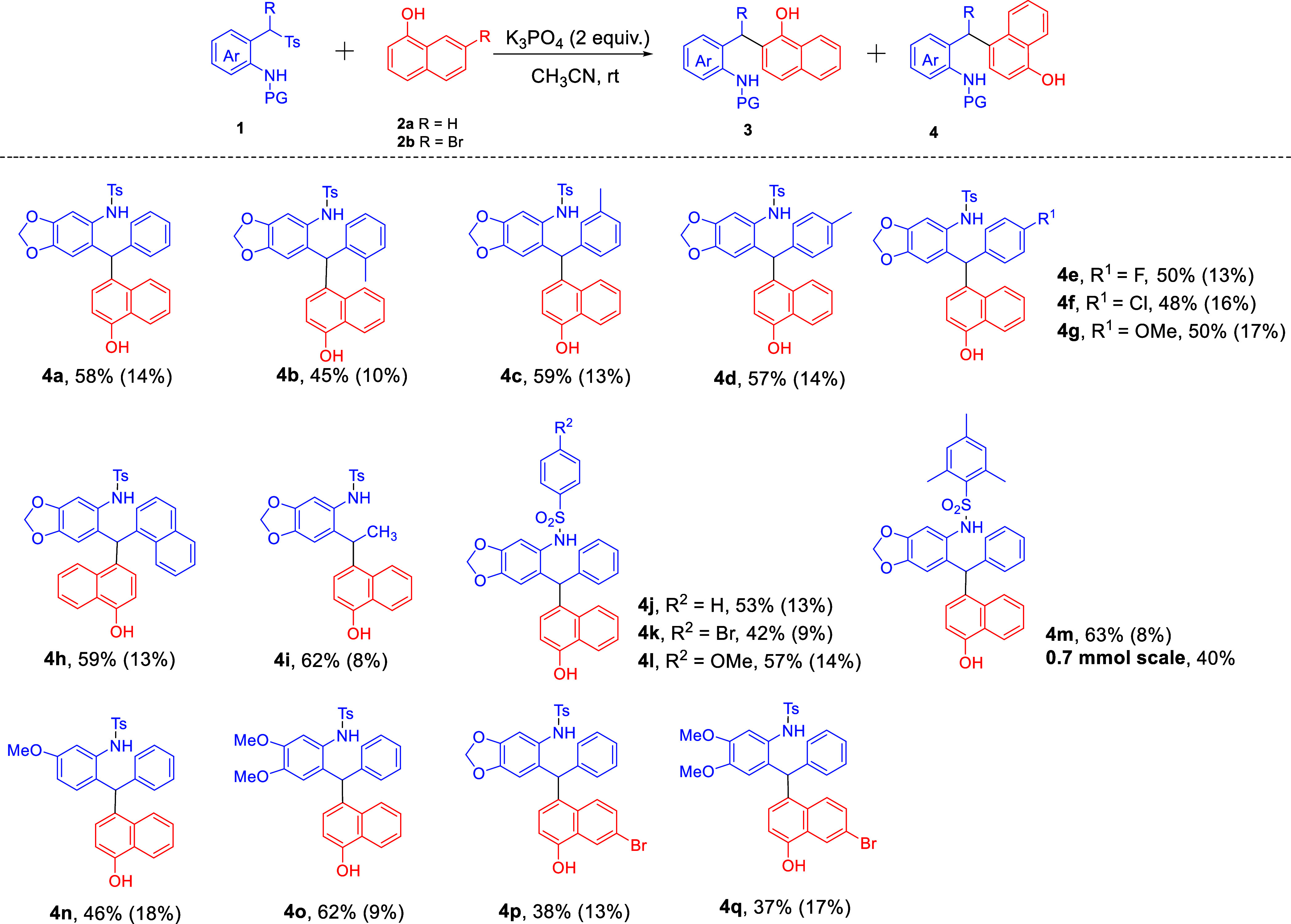
Scope of the *para*-Selective Friedel–Crafts Reaction^a^,^b^,^c^

a Unless otherwise noted, the solution of 0.1 mmol of **1**, 0.15 mmol of **2**, and 0.20 mmol of K_3_PO_4_ in 2.0 mL of CH_3_CN was stirred at r.t. (25–28 °C) for 72 h.
b Isolated yields.
c The values in the parentheses are *ortho*-substituted products **3.**

We then carried out the functionalization of *ortho*-selective Friedel–Crafts product **3a** and *para*-selective Friedel–Crafts product **4a**. Treatment of **3a** and **4a** with 4-bromobenzoic chloride **5** under basic conditions resulted in the esterification of hydroxy groups, leading to the formation of the ester products **6** and **7** in 83 and 31% reaction yields, respectively (Scheme 5).

**Scheme 5:**
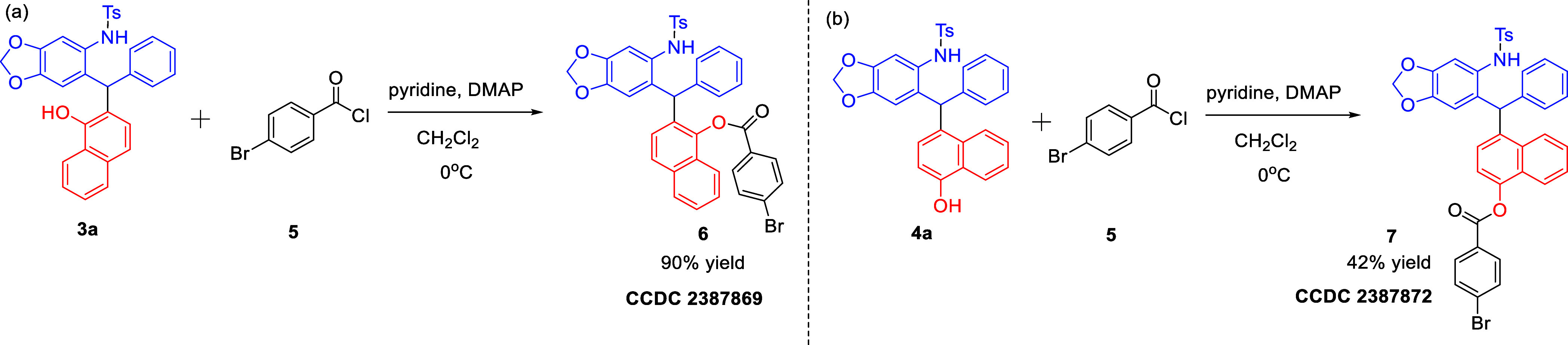
Transformation of **3a** and **4a**

The structure of *ortho*-selective Friedel–Crafts products and *para*-selective Friedel–Crafts products was confirmed on the basis of the X-ray crystallographic analysis of compounds **6** (CCDC 2387869) and **7** (CCDC 2387872).^16,17^ With the advantage of the crystallographic structure of products **6** and **7**, all other product structures were deduced by referring to that.

Several control experiments were carried out under optimal reaction conditions to demonstrate the importance of the O–H group in **2a**.

As shown in Scheme 6, the reaction of **1a** with methyl-substituted naphthol **8** under conditions with toluene (Scheme 6a) or CH_3_CN (Scheme 6b) did not provide the desired products. Notably, *ortho*-selective Friedel–Crafts product **3a** could not transform into *para*-selective Friedel–Crafts product **4a** under basic conditions, with CH_3_CN as the solvent (Scheme 6c). In addition, product **4a** could not be transformed into **3a** under basic conditions, with toluene as the solvent (Scheme 6d). These results indicated that both *ortho*-selective and *para*-selective Friedel–Crafts reactions are not reversible.

**Scheme 6:**
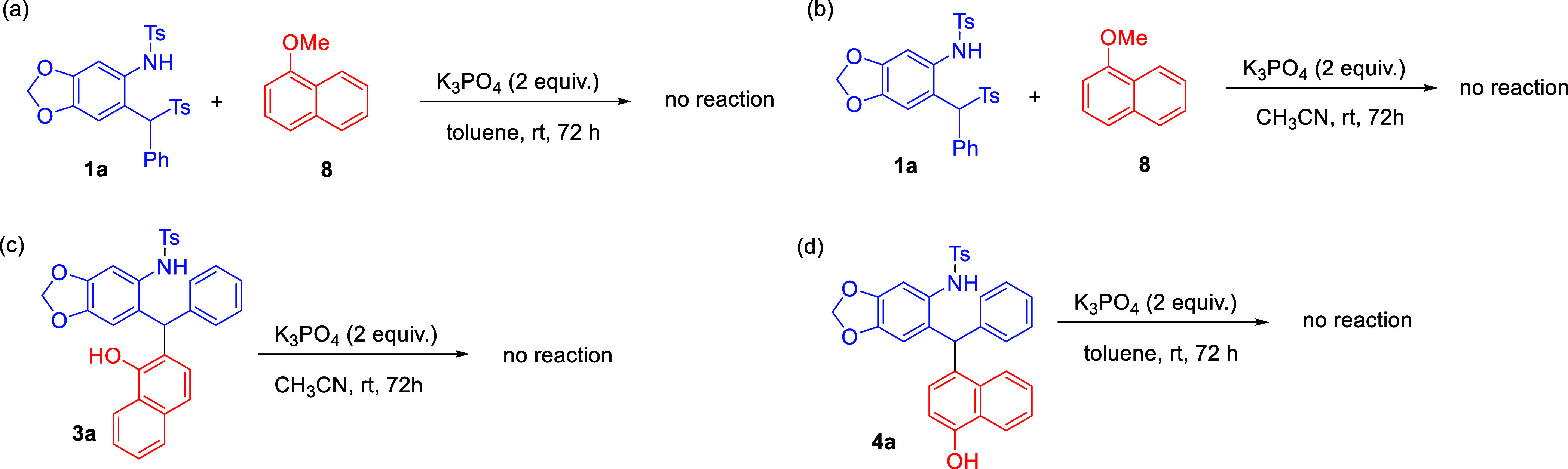
Control Experiments

To understand solvent-dependent selectivity, we computed reaction profile energies (Δ*G*_25 °C_) at the DFT level to gain deeper insight into the reaction process. In solvents like toluene, potassium cations favor the form of intermolecular interactions between substrates, which is preferential in *ortho* geometry and dictates the kinetic formation of this product (Figure 1).

**Figure 1:**
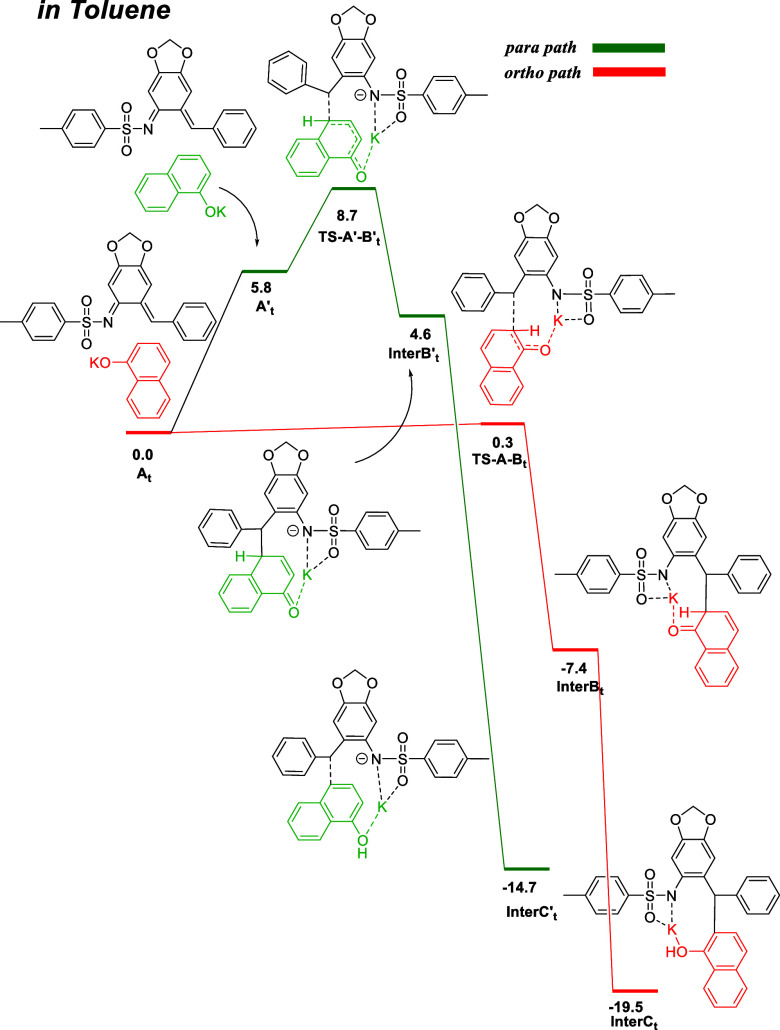
Key steps computed for the formation of product in toluene. Δ*G*_25 °C_ (kcal/mol) at TPSS0-D4/def2TZVPP-SMD (toluene).

In the lowest transition state conformer (**TS-A-Bt**), the potassium cation is coordinated by the naphthoxy and −NT moieties. The formation of the *ortho*-substituted product has a low (Δ*G*_25 °C_) energy barrier of 0.3 kcal/mol, and the product **InterCt** is formed at 19.5 kcal/mol. On the other hand, the *para*-substituted product represents a higher energy path (8.7 kcal/mol) through transition state **TS-A′-B**′**t,** and the **InterC**′**t** product is formed at −14.7 kcal/mol.

However, in coordinating solvents, such as acetonitrile, the interactions between the solvent and cation are stronger and enable different geometrical arrangements (Figure 2). Therefore, the path leading to the formation of a *para*-substituted product through transition state **TS-A-Ba** is more favored than the path with the **TS-A**′**-B**′**a** transition state by 0.6 kcal/mol. The energy of formation for both products is in a similar range (−11.9 for **InterC**′**a** and −12.1 kcal/mol for **InterCa**). The second potassium cation is required to interact with the anionic charge formed on nitrogen (−NTs) during the reaction course in acetonitrile solvent. Additionally, we found that up to 4 acetonitrile molecules can interact with one potassium cation in the transition state by applying Quantum Cluster Grow methodology following the molecular dynamics (MD) simulation prior to extensive conformational analysis with CREST (iMTD-sMTD algorithm).^17^

**Figure 2:**
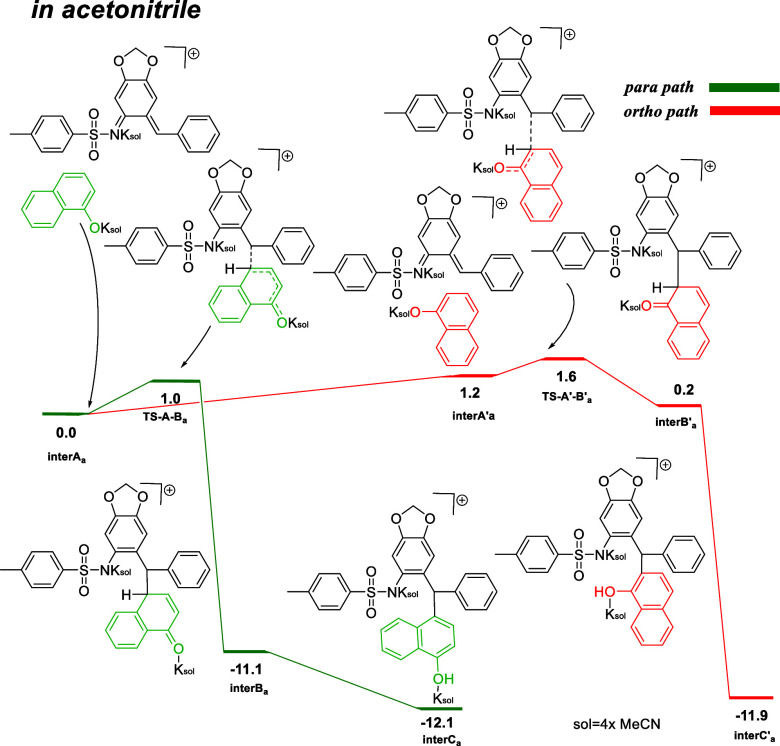
Key steps computed for the formation of product in acetonitrile. Δ*G*_25 °C_ (kcal/mol) at TPSS0-D4/def2TZVPP-SMD (acetonitrile) with explicit acetonitrile microsolvation.

Thus, our finding point’s reaction selectivity is related to the maximization of interactions between potassium solvent and solute varying from solvents. Therefore, in less coordinating solvents, such as toluene, potassium cations prefer to form intermolecular interactions between substrates, preferentially in *ortho* geometry and dictating this product formation kinetically (Figure 3). In the presence of a coordination solvent, the solvent–cation–solute interaction is preferential. Therefore, the favored conformation of the transition state maximizes the cation–solvent interaction with tilt angle geometries, making the *para* path preferential.^18^ Such a transition state complements a recent finding on innate solvent-mediated chemoselectivity, discovered by Gogoi and Jindal.^19^ During the reaction, the concentration of the product influences the selectivity, which is responsible for the formation of the para product in toluene solvent (SA, more comprehensive discussion with data is available in the Supporting Information).

**Figure 3:**
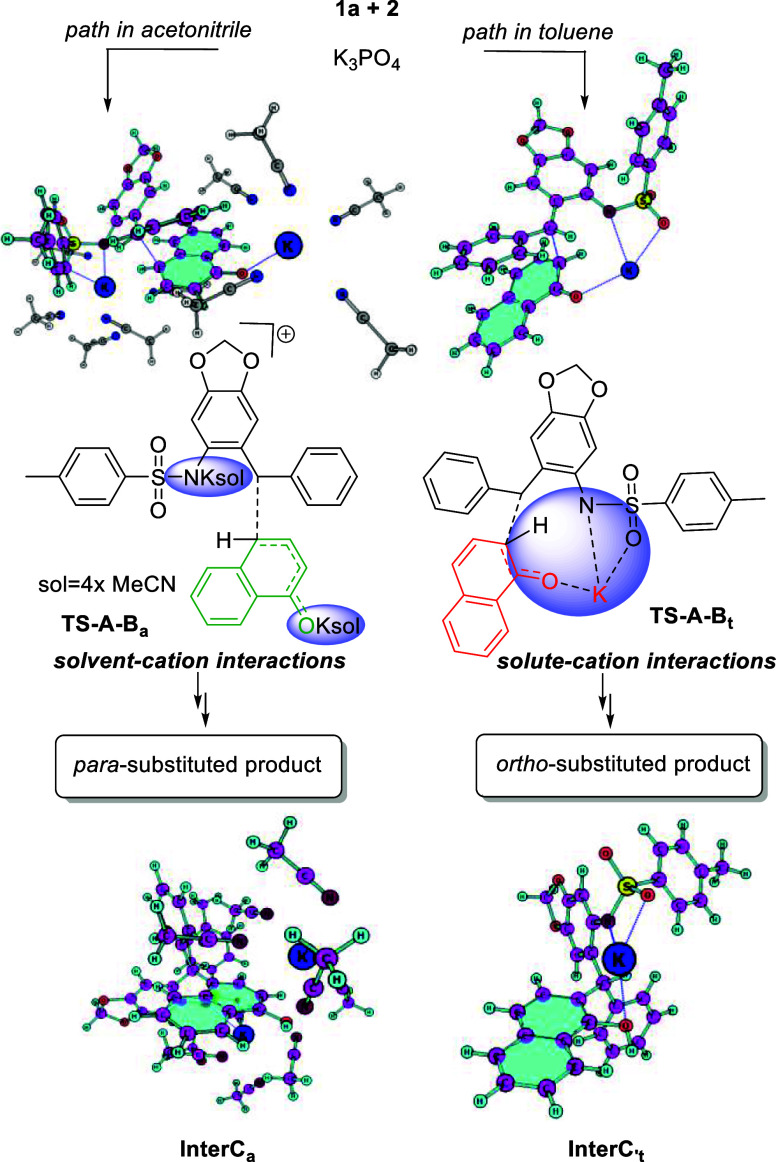
Pictorial representation of the solvent-induced selectivity.

In summary, we have developed the first solvent-controlled regioselective site-selectivity switchable Friedel–Crafts reaction of 1-naphthols and aza-*o*-QMs. In toluene, the *ortho-*selective Friedel–Crafts reaction was achieved, while the *para*-selective Friedel–Crafts reaction was accomplished in acetonitrile solvent.

Theoretical mechanistic investigations showed that potassium cations favor the formation of intermolecular interactions between substrates, preferentially in *ortho* geometry, in noncoordinating solvents, such as toluene. However, in coordinating solvents, such as acetonitrile, the interactions between the solvent and cation are stronger, leading to the formation of a *para*-selective pathway.

## Experimental Section

All commercially available reagents were used without further purification unless otherwise stated. All reaction solvents were purified before use. Proton nuclear magnetic resonance (^1^H NMR) spectra were recorded on a commercial instrument at 400 MHz. Carbon-13 nuclear magnetic resonance (^13^C­{^1^H} NMR) spectra were recorded at 100 MHz. The proton signal for residual nondeuterated solvent (δ 7.26 for CHCl_3_) was used as an internal reference for ^1^H NMR spectra. For ^13^C­{^1^H} NMR spectra, chemical shifts are reported relative to the δ 77.0 resonance of CHCl_3_. Coupling constants are reported in Hz. Melting points were determined on a BUCHI B-545 melting point apparatus and were uncorrected. High-resolution mass spectra were recorded on a Thermo Fisher Scientific LTQ Orbitrap XL mass spectrometer. The single crystal was measured by a Bruker D8 VENTURE X-ray single-crystal diffractometer. Analytical thin-layer chromatography (TLC) was performed on silica gel 60 F254 precoated plates with visualization under UV light. Column chromatography was generally performed using 40–63 μm (230–400 mesh) silica gel, typically using a 50–100:1 weight ratio of silica gel to crude product. 2-(Tosylmethyl)­anilines were prepared according to known procedures.^9d,9e,16^

### General Procedure for the Synthesis of **3**

In a 7 mL glass vial, 2-(tosylmethyl)­anilines **1** (0.1 mmol) and 1-naphthol **2** (0.15 mmol) were dissolved in 2.0 mL of anhydrous toluene. K_3_PO_4_ (0.20 mmol) was added, and the reaction mixture was then stirred at room temperature for 72 h. After the reaction was completed (confirmed by TLC), the reaction was purified by column chromatography to obtain the pure products **3**.

### Synthesis of 1.0 mmol Scale of **3n**

In a 25 mL RBF, 2-(tosylmethyl)­aniline **1n** (521 ng, 1 mmol) and 1-naphthol **2** (216 mg, 0.15 mmol) were dissolved in 20 mL of anhydrous toluene. K_3_PO_4_ (424 mg, 2 mmol) was added, and the reaction was then stirred at room temperature for 72 h. After the reaction was completed (confirmed by TLC), the reaction was purified by column chromatography (hexane/EA 30:1 to 3:1) to obtain pure product **3n** in 59% yield (300 mg).

### General Procedure for the Synthesis of **4**

In a 7 mL glass vial, 2-(tosylmethyl)­anilines **1** (0.1 mmol) and 1-naphthol **2** (0.14 mmol) were dissolved in 2.0 mL of anhydrous CH_3_CN. K_3_PO_4_ (0.20 mmol) was added, and the reaction was then stirred at room temperature for 72 h. After the reaction was completed (confirmed by TLC), the reaction was purified by column chromatography to obtain the pure products **4**.

### Synthesis of 0.7 mmol Scale of **4m**

In a 25 mL RBF, 2-(tosylmethyl)­aniline **1m** (394 ng, 0.7 mmol) and 1-naphthol **2** (151 mg, 0.15 mmol) were dissolved in 15 mL of anhydrous MeCN. K_3_PO_4_ (297 mg, 2 mmol) was added, and the reaction was then stirred at room temperature for 72 h. After the reaction was completed (confirmed by TLC), the reaction was purified by column chromatography (hexane/EA 30:1 to 3:1) to obtain pure product **4n** in 40% yield (220 mg).

### *N*-(6-((1-Hydroxynaphthalen-2-yl)­(phenyl)­methyl)­benzo­[*d*]­[1,3]­dioxol-5-yl)-4-methylbenzenesulfonamide (**3a**)

Purified by silica gel column chromatography eluting with hexane/EA (30:1 to 3:1); 62% yield (33 mg); yellow powder; mp: 217–218 °C; ^1^H NMR­(400 MHz, CDCl_3_): δ 8.24–8.22 (m, 1H), 7.77–7.72 (m, 1H), 7.71 (d, *J* = 8.1 Hz, 2H), 7.49–7.47 (m, 2H), 7.33–7.25 (m, 6H), 6.91 (s, 1H), 6.89 (s, 1H), 6.81 (d, *J* = 8.5 Hz, 1H), 6.67 (bs, 1H), 6.40 (s, 1H), 6.38 (s, 1H), 6.11 (bs, 1H), 5.98 (s, 1H), 5.92 (s, 1H), 5.88 (s, 1H), 2.45 (s, 3H); ^13^C­{^1^H} NMR (100 MHz, CDCl_3_): δ 149.0, 147.5, 146.6, 144.3, 141.0, 136.2, 135.0, 133.7, 129.9, 129.3, 128.7, 127.6, 127.5, 127.2, 126.9, 126.2, 125.5, 124.8, 121.9, 120.9, 120.0, 109.6, 108.9, 101.7, 45.4, 21.6; HRMS (ESI) *m*/*z*: [M + Na]^+^ calcd for C_31_H_25_NO_5_NaS: 546.1346; found: 546.1355.

### *N*-(6-((1-Hydroxynaphthalen-2-yl)­(*o*-tolyl)­methyl)­benzo­[*d*]­[1,3]­dioxol-5-yl)-4-methylbenzenesulfonamide (**3b**)

Purified by silica gel column chromatography eluting with hexane/EA (30:1 to 3:1); 56% yield (30 mg); white powder; mp: 242–243 °C; ^1^H NMR­(400 MHz, CDCl_3_): δ 8.24–8.22 (m, 1H), 7.70–7.68 (m, 1H), 7.65 (d, *J* = 8.2 Hz, 2H), 7.42–7.39 (m, 2H), 7.24–7.19 (m, 3H), 7.15–7.10 (m, 2H), 7.05–7.01 (m, 1H), 6.76 (s, 1H), 6.66–6.62 (m, 2H), 6.34 (s, 1H), 6.18 (s, 1H), 6.08 (s, 1H), 5.82 (dd, *J* = 14.5, 1.2 Hz, 2H), 5.68 (s, 1H), 2.39 (s, 3H), 2.14 (s, 3H); ^13^C­{^1^H} NMR (100 MHz, CDCl_3_): δ 149.7, 147.7, 146.6, 144.4, 139.6, 137.3, 136.2, 133.8, 130.9, 129.9, 128.7, 127.6, 127.3, 127.1, 126.8, 126.5, 126.1, 126.0, 125.2, 124.9, 122.4, 119.7, 119.3, 110.2, 109.1, 101.7, 42.8, 21.6, 19.5; HRMS (ESI) *m*/*z*: [M + Na]^+^ calcd for C_32_H_27_NO_5_NaS: 560.1502; found: 560.1503.

### *N*-(6-((1-Hydroxynaphthalen-2-yl)­(*m*-tolyl)­methyl)­benzo­[*d*]­[1,3]­dioxol-5-yl)-4-methylbenzenesulfonamide (**3c**)

Purified by silica gel column chromatography eluting with hexane/EA (30:1 to 3:1); 76% yield (41 mg); white powder; mp: 214–215 °C; ^1^H NMR­(400 MHz, CDCl_3_): δ 8.19–8.17 (m, 1H), 7.72–7.70 (m, 1H), 7.67 (d, *J* = 8.0 Hz, 2H), 7.43–7.40 (m, 2H), 7.28–7.24 (m, 3H), 7.09 (t, *J* = 7.5 Hz, 1H), 6.99 (d, *J* = 7.6 Hz, 1H), 6.76 (d, *J* = 8.6 Hz, 1H), 6.67 (s, 1H), 6.62 (d, *J* = 7.6 Hz, 1H), 6.53 (s, 1H), 6.36 (s, 1H), 6.34 (s, 1H), 6.09 (s, 1H), 5.87 (s, 1H), 5.84 (s, 1H), 5.83 (s, 1H), 2.40 (s, 3H), 2.22 (s, 3H); ^13^C­{^1^H} NMR (100 MHz, CDCl_3_): δ 149.0, 147.4, 146.6, 144.2, 140.9, 138.3, 136.2, 134.8, 133.7, 130.0, 129.8, 128.5, 127.8, 127.6, 127.4, 127.3, 126.9, 126.3, 126.2, 125.4, 124.8, 121.9, 121.0, 120.0, 109.6, 108.9, 101.7, 45.4, 21.6, 21.5; HRMS (ESI) *m*/*z*: [M + Na]^+^ calcd for C_32_H_27_NO_5_NaS: 560.1502; found: 560.1503.

### *N*-(6-((1-Hydroxynaphthalen-2-yl)­(*p*-tolyl)­methyl)­benzo­[*d*]­[1,3]­dioxol-5-yl)-4-methylbenzenesulfonamide (**3d**)

Purified by silica gel column chromatography eluting with hexane/EA (30:1 to 3:1); 69% yield (37 mg); yellow powder; mp: 123–124 °C; ^1^H NMR­(400 MHz, CDCl_3_): δ 8.24–8.22 (m, 1H), 7.77–7.75 (m, 1H), 7.72 (d, *J* = 8.2 Hz, 2H), 7.49–7.44 (m, 2H), 7.33–7.29 (m, 3H), 7.07 (d, *J* = 7.8 Hz, 2H), 6.82 (d, *J* = 8.6 Hz, 1H), 6.79 (d, *J* = 7.8 Hz, 2H), 6.56 (s, 1H), 6.40 (d, *J* = 6.8 Hz, 2H), 6.14 (s, 1H), 5.91 (s, 1H), 5.88 (s, 2H), 2.45 (s, 3H), 2.32 (s, 3H); ^13^C­{^1^H} NMR (100 MHz, CDCl_3_): δ 149.0, 147.4, 146.6, 144.3, 137.8, 136.6, 136.2, 134.9, 133.7, 129.8, 129.4, 129.2, 127.5, 127.4, 127.2, 126.9, 126.2, 125.4, 124.8, 121.9, 121.0, 120.0, 109.5, 108.9, 101.7, 45.1, 21.6, 21.0; HRMS (ESI) *m*/*z*: [M + Na]^+^ calcd for C_32_H_27_NO_5_NaS: 560.1502; found: 560.1503.

### *N*-(6-((4-Fluorophenyl)­(1-hydroxynaphthalen-2-yl)­methyl)­benzo­[*d*]­[1,3]­dioxol-5-yl)-4-methylbenzenesulfonamide (**3e**)

Purified by silica gel column chromatography eluting with hexane/EA (30:1 to 3:1); 83% yield (45 mg); white powder; mp: 87–88 °C; ^1^H NMR­(400 MHz, CDCl_3_): δ 8.24–8.22 (m, 1H), 7.78–7.75 (m, 1H), 7.71 (d, *J* = 8.4 Hz, 2H), 7.51–7.44 (m, 2H), 7.33 (d, *J* = 8.6 Hz, 1H), 7.29 (d, *J* = 8.0 Hz, 2H), 6.92 (t, *J* = 8.7 Hz, 2H), 6.85–6.80 (m, 4H), 6.39 (s, 1H), 6.34 (s, 1H), 6.22 (s, 1H), 6.03 (s, 1H), 5.92 (d, *J* = 1.2 Hz, 1H), 5.89 (d, *J* = 1.2 Hz, 1H), 2.44 (s, 3H); ^13^C­{^1^H} NMR (100 MHz, CDCl_3_): δ 161.6 (d, *J* = 244 Hz), 149.0, 147.6, 146.7, 144.4, 137.0, 136.0, 135.1, 133.7, 130.8 (d, *J* = 7.6 Hz), 129.8, 127.6, 127.5, 127.0, 126.8, 126.3, 125.6, 124.8, 121.8, 120.8, 120.1, 115.4 (d, *J* = 21.3 Hz), 109.2 (d, *J* = 47.0 Hz), 101.8, 44.4, 21.6;^19^ F NMR (376 MHz, CDCl_3_): δ −115.7 (s, 1F)_;_ HRMS (ESI) *m*/*z*: [M + Na]^+^ calcd for C_31_H_24_NO_5_FNaS: 564.1251; found: 564.1247.

### *N*-(6-((4-Chlorophenyl)­(1-hydroxynaphthalen-2-yl)­methyl)­benzo­[*d*]­[1,3]­dioxol-5-yl)-4-methylbenzenesulfonamide (**3f**)

Purified by silica gel column chromatography eluting with hexane/EA (30:1 to 3:1); 79% yield (44 mg); yellow powder; mp: 193–194 °C; ^1^H NMR­(400 MHz, CDCl_3_): δ 8.24–8.21 (m, 1H), 7.78–7.76 (m, 1H), 7.70 (d, *J* = 8.2 Hz, 2H), 7.52–7.45 (m, 2H), 7.33 (d, *J* = 8.5 Hz, 1H), 7.28 (d, *J* = 8.2 Hz, 2H), 7.20 (d, *J* = 8.5 Hz, 2H), 6.92 (s, 1H), 6.82–6.78 (m, 3H), 6.39 (s, 1H), 6.32 (s, 1H), 6.26 (s, 1H), 6.05 (s, 1H), 5.93 (d, *J* = 1.4 Hz, 1H), 5.88 (d, *J* = 1.4 Hz, 1H), 2.44 (s, 3H); ^13^C­{^1^H} NMR (100 MHz, CDCl_3_): δ 148.9, 147.5, 146.7, 144.3, 140.0, 135.9, 134.7, 133.7, 132.5, 130.6, 129.8, 128.5, 127.5, 127.0, 126.9, 126.3, 125.6, 124.8, 121.8, 120.8, 120.2, 109.4, 108.9, 101.8, 44.3, 21.6; HRMS (ESI) *m*/*z*: [M – H]^−^ calcd for C_31_H_23_NO_5_SCl: 556.0990; found: 556.0991.

### *N*-(6-((1-Hydroxynaphthalen-2-yl)­(4-methoxyphenyl)­methyl)­benzo­[*d*]­[1,3]­dioxol-5-yl)-4-methylbenzenesulfonamide (**3g**)

Purified by silica gel column chromatography eluting with hexane/EA (30:1 to 3:1); 76% yield (42 mg); brown powder; mp: 97–98 °C; ^1^H NMR­(400 MHz, CDCl_3_): δ 8.24–8.22 (m, 1H), 7.77–7.75 (m, 1H), 7.72 (d, *J* = 8.2 Hz, 2H), 7.50–7.44 (m, 2H), 7.33–7.29 (m, 3H), 6.83–6.87 (m, 5H), 6.61 (bs, 1H), 6.41 (s, 1H), 6.38 (s, 1H), 6.20 (s, 1H), 5.91 (s, 1H), 5.88 (s, 2H), 3.78–3.77 (s, 3H), 2.45 (s, 3H); ^13^C­{^1^H} NMR (100 MHz, CDCl_3_): δ 158.4, 149.0, 147.4, 146.6, 144.3, 136.2, 135.0, 133.7, 132.8, 130.3, 129.8, 127.5, 127.4, 127.2, 126.9, 126.2, 125.4, 124.8, 121.9, 121.1, 120.0, 114.0, 109.4, 108.9, 101.7, 55.2, 44.7, 21.6; HRMS (ESI) *m*/*z*: [M + Na]^+^ calcd for C_32_H_27_NO_6_NaS: 576.1451; found: 576.1457.

### *N*-(6-((1-Hydroxynaphthalen-2-yl)­(naphthalen-1-yl)­methyl)­benzo­[*d*]­[1,3]­dioxol-5-yl)-4-methylbenzenesulfonamide (**3h**)

Purified by silica gel column chromatography eluting with hexane/EA (30:1 to 3:1); 52% yield (30 mg); brown powder; mp: 214–215 °C; ^1^H NMR­(400 MHz, CDCl_3_): δ 8.30 (d, *J* = 7.8 Hz, 1H), 7.86 (d, *J* = 8.2 Hz, 1H), 7.79 (d, *J* = 8.4 Hz, 1H), 7.74–7.69 (m, 4H), 7.51–7.43 (m, 3H), 7.37–7.26 (m, 4H), 7.21 (d, *J* = 8.6 Hz, 1H), 6.84 (d, *J* = 7.0 Hz, 1H), 6.75 (s, 1H), 6.71 (s, 1H), 6.67 (d, *J* = 8.6 Hz, 1H), 6.39 (s, 1H), 6.37 (s, 1H), 5.92 (s, 1H), 5.89 (s, 1H), 5.83 (s, 1H), 2.44 (s, 3H); ^13^C­{^1^H} NMR (100 MHz, CDCl_3_): δ 149.1, 147.6, 146.7, 144.4, 137.2, 136.3, 135.0, 134.0, 133.8, 131.7, 129.9, 128.6, 128.2, 127.7, 127.4, 127.1, 126.8, 126.7, 126.2, 126.0, 125.1, 125.0, 124.0, 122.2, 120.5, 120.1, 110.4, 109.0, 101.8, 42.4, 21.7; HRMS (ESI) *m*/*z*: [M + Na]^+^ calcd for C_35_H_27_NO_5_NaS: 596.1502; found: 596.1508.

### *N*-(6-(1-(1-Hydroxynaphthalen-2-yl)­ethyl)­benzo­[*d*]­[1,3]­dioxol-5-yl)-4-methylbenzenesulfonamide (**3i**)

Purified by silica gel column chromatography eluting with hexane/EA (30:1 to 3:1); 34% yield (16 mg); orange powder; mp: 94–95 °C; ^1^H NMR­(400 MHz, CDCl_3_): δ 8.11 (d, *J* = 8.0 Hz, 1H), 7.76 (d, *J* = 7.4 Hz, 1H), 7.68 (d, *J* = 8.2 Hz, 2H), 7.48–7.41 (m, 3H), 7.34 (d, *J* = 8.6 Hz, 1H), 7.24 (d, *J* = 8.0 Hz, 2H), 7.18 (s, 1H), 6.99 (s, 1H), 6.62 (s, 1H), 6.45 (s, 1H), 5.89–5.84 (m, 2H), 4.66 (q, *J* = 7.0 Hz, 1H), 2.41 (s, 3H), 1.35 (d, *J* = 7.0 Hz, 3H); ^13^C­{^1^H} NMR (100 MHz, CDCl_3_): δ 147.7, 147.2, 146.0, 144.1, 136.8, 135.8, 133.3, 129.7, 127.6, 127.5, 126.3, 125.9, 125.5, 124.8, 124.4, 123.5, 121.0, 120.6, 107.7, 107.0, 101.5, 32.2, 21.6, 20.5; HRMS (ESI) *m*/*z*: [M + Na]^+^ calcd for C_26_H_23_NO_5_NaS: 484.1189; found: 484.1180.

### *N*-(6-((1-Hydroxynaphthalen-2-yl)­(phenyl)­methyl)­benzo­[*d*]­[1,3]­dioxol-5-yl)­benzenesulfonamide (**3j**)

Purified by silica gel column chromatography eluting with hexane/EA (30:1 to 3:1); 43% yield (22 mg); light yellow powder; mp: 193–194 °C; ^1^H NMR­(400 MHz, CDCl_3_): δ 8.24–8.22 (m, 1H), 7.85 (d, *J* = 7.4 Hz, 2H), 7.78–7.75 (m, 1H), 7.64 (t, *J* = 7.4 Hz, 1H), 7.55–7.48 (m, 4H), 7.33–7.28 (m, 4H), 6.92 (d, *J* = 6.5 Hz, 2H), 6.81 (d, *J* = 8.5 Hz, 1H), 6.60 (s, 1H), 6.40 (s, 1H), 6.33 (s, 1H), 6.08 (s, 1H), 6.00 (s, 1H), 5.92 (s, 1H), 5.8 (s, 1H); ^13^C­{^1^H} NMR (100 MHz, CDCl_3_): δ 149.1, 147.6, 146.6, 141.0, 139.1, 135.2, 133.7, 133.4, 129.33, 129.26, 128.7, 127.6, 127.5, 127.2, 127.0, 126.6, 126.3, 125.4, 124.8, 122.0, 120.8, 120.0, 109.6, 108.9, 101.8, 45.5; HRMS (ESI) *m*/*z*: [M + Na]^+^ calcd for C_30_H_23_NO_5_NaS: 532.1189; found: 532.1198.

### 4-Bromo-*N*-(6-((1-hydroxynaphthalen-2-yl)­(phenyl)­methyl)­benzo­[*d*]­[1,3]­dioxol-5-yl)­benzenesulfonamide (**3k**)

Purified by silica gel column chromatography eluting with hexane/EA (30:1 to 3:1); 43% yield (25 mg); light yellow powder; mp: 187–188 °C; ^1^H NMR­(400 MHz, CDCl_3_): δ 8.19–8.16 (m, 1H), 7.79–7.77 (m, 1H), 7.68 (dd, *J* = 19.5, 6.8 Hz, 2H), 7.63 (dd, *J* = 19.5, 6.8 Hz, 2H), 7.52–7.47 (m, 2H), 7.35–7.27 (m, 4H), 6.90–6.88 (m, 2H), 6.82 (d, *J* = 8.5 Hz, 1H), 6.46 (s, 1H), 6.40 (s, 2H), 6.16 (s, 1H), 5.94 (d, *J* = 1.4 Hz, 1H), 5.91 (d, *J* = 1.4 Hz, 2H); ^13^C­{^1^H} NMR (100 MHz, CDCl_3_): δ 148.8, 147.5, 146.7, 140.9, 138.2, 134.7, 133.7, 132.5, 129.2, 129.0, 128.7, 128.4, 127.6, 127.1, 127.0, 126.5, 126.3, 125.6, 124.7, 121.6, 121.0, 120.3, 109.7, 108.6, 101.8, 45.2; HRMS (ESI) *m*/*z*: [M – H]^−^ calcd for C_30_H_21_NO_5_SBr: 586.0329; found: 586.0330.

### *N*-(6-((1-Hydroxynaphthalen-2-yl)­(phenyl)­methyl)­benzo­[*d*]­[1,3]­dioxol-5-yl)-4-methoxybenzenesulfonamide (**3l**)

Purified by silica gel column chromatography eluting with hexane/EA (30:1 to 3:1); 69% yield (37 mg); orange powder; mp: 214–215 °C; ^1^H NMR­(400 MHz, CDCl_3_): δ 8.25–8.22 (m, 1H), 7.77–7.75 (m, 3H), 7.49–7.46 (m, 2H), 7.32 (d, *J* = 8.6 Hz, 1H), 7.29–7.23 (m, 3H), 6.97–6.92 (m, 4H), 6.82 (d, *J* = 8.6 Hz, 1H), 6.75 (s, 1H), 6.41 (s, 1H), 6.35 (s, 1H), 6.11 (s, 1H), 6.04 (s, 1H), 5.91 (s, 1H), 5.88 (s, 1H), 3.88 (s, 3H); ^13^C­{^1^H} NMR (100 MHz, CDCl_3_): δ 163.4, 149.1, 147.5, 146.6, 141.1, 135.1, 133.7, 130.6, 129.8, 129.3, 128.6, 127.5, 127.3, 127.0, 126.9, 126.2, 125.4, 124.9, 122.0, 121.0, 120.0, 114.4, 109.6, 108.9, 101.7, 55.7, 45.4; HRMS (ESI) *m*/*z*: [M + Na]^+^ calcd for C_31_H_25_NO_6_NaS: 562.1295; found: 562.1288.

### *N*-(6-((1-Hydroxynaphthalen-2-yl)­(phenyl)­methyl)­benzo­[*d*]­[1,3]­dioxol-5-yl)-2,4,6-trimethylbenzenesulfonamide (**3m**)

Purified by silica gel column chromatography eluting with hexane/EA (30:1 to 3:1); 43% yield (24 mg); light yellow powder; mp: 207–208 °C; ^1^H NMR­(400 MHz, CDCl_3_): δ 8.31–8.29 (m, 1H), 7.76–7.75 (m, 1H), 7.47–7.46 (m, 2H), 7.35–7.25 (m, 4H), 7.05 (d, *J* = 7.2 Hz, 2H), 6.99 (s, 2H), 6.81–6.76 (m, 2H), 6.43 (s, 1H), 6.20 (s, 1H), 6.08 (s, 1H), 5.98 (s, 1H), 5.89 (s, 1H), 5.86 (s, 1H), 2.51 (s, 6H), 2.34 (s, 3H); ^13^C­{^1^H} NMR (100 MHz, CDCl_3_): δ 149.6, 147.6, 146.6, 143.1, 141.1, 139.7, 135.6, 133.7, 133.2, 132.2, 129.5, 128.9, 127.3, 127.2, 127.1, 126.7, 126.2, 125.2, 125.0, 122.5, 120.8, 119.5, 109.8, 109.2, 101.7, 45.8, 23.2, 21.0; HRMS (ESI) *m*/*z*: [M + Na]^+^ calcd for C_33_H_29_NO_5_NaS: 574.1659; found: 574.1654.

### *N*-(2-((1-Hydroxynaphthalen-2-yl)­(phenyl)­methyl)-5-methoxyphenyl)-4-methylbenzenesulfonamide (**3n**)

Purified by silica gel column chromatography eluting with hexane/EA (30:1 to 3:1); 81% yield (41 mg); brown powder; mp: 95–96 °C; ^1^H NMR­(400 MHz, CDCl_3_): δ 8.19–8.17 (m, 1H), 7.78–7.76 (m, 1H), 7.64 (d, *J* = 8.2 Hz, 2H), 7.49–7.45 (m, 2H), 7.33 (d, *J* = 8.6 Hz, 1H), 7.26–7.22 (m, 5H), 6.89–6.79 (m, 4H), 6.68–6.66 (m, 2H), 6.42 (s, 1H), 6.33 (s, 1H), 5.85 (s, 1H), 3.66 (s, 3H), 2.42 (s, 3H); ^13^C­{^1^H} NMR (100 MHz, CDCl_3_): δ 158.9, 148.8, 144.2, 141.1, 136.1, 134.9, 133.7, 130.6, 129.7, 129.3, 128.6, 127.6, 127.5, 127.3, 126.9, 126.2, 125.5, 124.8, 121.7, 121.1, 120.3, 113.1, 112.1, 55.3, 44.9, 21.6; HRMS (ESI) *m*/*z*: [M + Na]^+^ calcd for C_31_H_27_NO_4_NaS: 532.1553; found: 532.1549.

### *N*-(2-((1-Hydroxynaphthalen-2-yl)­(phenyl)­methyl)-4,5-dimethoxyphenyl)-4-methylbenzenesulfonamide (**3o**)

Purified by silica gel column chromatography eluting with hexane/EA (30:1 to 3:1); 71% yield (38 mg); white powder; mp: 191–192 °C; ^1^H NMR­(400 MHz, CDCl_3_): δ 8.24–8.22 (m, 1H), 7.77–7.75 (m, 1H), 7.69 (d, *J* = 8.2 Hz, 2H), 7.50–7.45 (m, 2H), 7.33–7.24 (m, 6H), 6.89–6.87 (m, 2H), 6.83 (d, *J* = 8.6 Hz, 1H), 6.56 (s, 1H), 6.41 (s, 1H), 6.36 (s, 1H), 6.10 (s, 1H), 5.97 (s, 1H), 3.59 (s, 3H), 3.54 (s, 3H), 2.44 (s, 3H); ^13^C­{^1^H} NMR (100 MHz, CDCl_3_): δ 149.1, 148.6, 147.7, 144.2, 141.3, 136.2, 133.7, 132.9, 129.7, 129.3, 128.6, 127.8, 127.5, 127.3, 126.9, 126.2, 126.0, 125.4, 124.9, 122.0, 121.0, 120.0, 112.3, 111.7, 55.7, 45.1, 21.6; HRMS (ESI) *m*/*z*: [M + Na]^+^ calcd for C_32_H_29_NO_5_NaS: 562.1659; found: 562.1661.

### *N*-(6-((7-Bromo-1-hydroxynaphthalen-2-yl)­(phenyl)­methyl)­benzo­[*d*]­[1,3]­dioxol-5-yl)-4-methylbenzenesulfonamide (**3p**)

Purified by silica gel column chromatography eluting with hexane/EA (30:1 to 3:1); 78% yield (47 mg); white powder; mp: 284–285 °C; ^1^H NMR­(400 MHz, CDCl_3_): δ 8.46 (d, *J* = 2.0 Hz, 1H), 7.71 (d, *J* = 8.3 Hz, 2H), 7.61 (d, *J* = 8.7 Hz, 1H), 7.52 (dd, *J* = 8.7, 2.0 Hz, 1H), 7.32–7.24 (m, 6H), 7.06 (s, 1H), 6.96–6.94 (m, 2H), 6.82 (d, *J* = 8.4 Hz, 1H), 6.38 (s, 1H), 6.21 (s, 1H), 6.12 (d, *J* = 3.4 Hz, 2H), 5.91 (d, *J* = 1.2 Hz, 1H), 5.88 (d, *J* = 1.4 Hz, 1H), 2.45 (s, 3H); ^13^C­{^1^H} NMR (100 MHz, CDCl_3_): δ 148.5, 147.7, 146.6, 144.4, 140.9, 135.9, 135.4, 132.0, 129.9, 129.5, 129.3, 129.0, 128.7, 127.7, 127.6, 127.0, 126.7, 126.1, 124.9, 122.2, 119.6, 119.4, 109.5, 109.1, 101.8, 45.5, 21.6; HRMS (ESI) *m*/*z*: [M + Na]^+^ calcd for C_31_H_24_NO_5_NaSBr: 624.0451; found: 624.0458.

### *N*-(2-((7-Bromo-1-hydroxynaphthalen-2-yl)­(phenyl)­methyl)-4,5-dimethoxyphenyl)-4-methylbenzenesulfonamide (**3q**)

Purified by silica gel column chromatography eluting with hexane/EA (30:1 to 3:1); 75% yield (46 mg); white powder; mp: 239–240 °C; ^1^H NMR­(400 MHz, DMSO-d6): δ 9.48 (s, 1H), 9.05 (s, 1H), 8.42 (d, *J* = 1.5 Hz, 1H), 7.77 (d, *J* = 8.7 Hz, 1H), 7.56–7.51 (m, 3H), 7.32 (d, *J* = 8.6 Hz, 1H), 7.23–7.21 (m, 4H), 7.18–7.14 (m, 1H), 6.92–6.90 (m, 3H), 6.54 (s, 1H), 6.38 (s, 1H), 6.16 (s, 1H), 3.45 (s, 3H), 3.37 (s, 3H), 2.28 (s, 3H); ^13^C­{^1^H} NMR (100 MHz, DMSO-d6): δ 148.6, 147.1, 146.6, 143.9, 143.0, 136.9, 133.9, 131.5, 130.0, 129.4, 128.8, 128.4, 128.2, 128.0, 127.2, 126.9, 126.2, 125.8, 125.8, 124.3, 119.0, 118.2, 113.6, 111.1, 55.3, 55.0, 43.3, 20.9; HRMS (ESI) *m*/*z*: [M + Na]^+^ calcd for C_32_H_28_NO_5_NaSBr: 640.0764; found: 640.0760.

### *N*-(6-((4-Hydroxynaphthalen-1-yl)­(phenyl)­methyl)­benzo­[*d*]­[1,3]­dioxol-5-yl)-4-methylbenzenesulfonamide (**4a**)

Purified by silica gel column chromatography eluting with hexane/EA (30:1 to 3:1); 58% yield (30 mg); light yellow powder; mp: 213–214 °C; ^1^H NMR­(400 MHz, CDCl_3_): δ 8.22 (d, *J* = 8.2 Hz, 1H), 7.69 (d, *J* = 8.0 Hz, 2H), 7.45 (t, *J* = 7.4 Hz, 1H), 7.38 (d, *J* = 7.8 Hz, 2H), 7.32 (t, *J* = 7.5 Hz, 1H), 7.24–7.20 (m, 4H), 7.07 (s, 1H), 6.63–6.56 (m, 4H), 6.02 (s, 1H), 5.91 (s, 2H), 5.83 (s, 1H), 5.60 (s, 1H), 5.40 (s, 1H), 2.50 (s, 3H); ^13^C­{^1^H} NMR (100 MHz, CDCl_3_): δ 151.3, 146.5, 146.3, 144.1, 141.8, 137.1, 132.9, 132.4, 129.9, 129.2, 129.1, 128.8, 127.4, 127.3, 127.2, 127.0, 126.9, 125.2, 125.0, 123.7, 122.5, 109.9, 108.4, 107.7, 101.5, 47.8, 21.6; HRMS (ESI) *m*/*z*: [M + Na]^+^ calcd for C_31_H_25_NO_5_NaS: 546.1346; found: 546.1342

### *N*-(6-((4-Hydroxynaphthalen-1-yl)­(*o*-tolyl)­methyl)­benzo­[*d*]­[1,3]­dioxol-5-yl)-4-methylbenzenesulfonamide (**4b**)

Purified by silica gel column chromatography eluting with hexane/EA (30:1 to 3:1); 45% yield (24 mg); yellow powder; mp: 189–190 °C; ^1^H NMR­(400 MHz, CDCl_3_): δ 8.23 (d, *J* = 8.2 Hz, 1H), 7.71 (bs, 2H), 7.48–7.31 (m, 5H), 7.18 (bs, 2H), 7.02 (bs, 2H), 6.63–6.53 (m, 3H), 5.97 (s, 1H), 5.88 (d, *J* = 4.8 Hz, 2H), 5.81–5.78 (m, 2H), 5.54 (s, 1H), 2.46 (s, 3H), 1.92 (s, 3H); ^13^C­{^1^H} NMR (100 MHz, CDCl_3_): δ 151.2, 146.5, 146.1, 144.0, 139.9, 137.7, 132.7, 129.9, 129.1, 127.4, 127.3, 127.2, 126.9, 126.4, 125.1, 123.6, 122.4, 109.7, 107.8, 107.5, 101.5, 44.9, 21.7, 19.1; HRMS (ESI) *m*/*z*: [M + Na]^+^ calcd for C_32_H_27_NO_5_NaS: 560.1502; found: 560.1496.

### *N*-(6-((4-Hydroxynaphthalen-1-yl)­(*m*-tolyl)­methyl)­benzo­[*d*]­[1,3]­dioxol-5-yl)-4-methylbenzenesulfonamide (**4c**)

Purified by silica gel column chromatography eluting with hexane/EA (30:1 to 3:1); 59% yield (32 mg); yellow powder; mp: 120–121 °C; ^1^H NMR­(400 MHz, CDCl_3_): δ 8.21 (d, *J* = 8.4 Hz, 1H), 7.69 (d, *J* = 7.8 Hz, 2H), 7.44 (t, *J* = 7.4 Hz, 1H), 7.38 (d, *J* = 7.6 Hz, 2H), 7.32 (t, *J* = 7.3 Hz, 1H), 7.22 (d, *J* = 8.4 Hz, 1H), 7.09–7.06 (m, 2H), 7.01 (d, *J* = 7.6 Hz, 1H), 6.63 (d, *J* = 7.8 Hz, 1H), 6.56 (d, *J* = 7.6 Hz, 1H), 6.45 (bs, 2H), 6.02 (s, 1H), 5.91 (s, 2H), 5.87 (s, 1H), 5.78 (s, 1H), 5.33 (s, 1H), 2.50 (s, 3H), 2.23 (s, 3H); ^13^C­{^1^H} NMR (100 MHz, CDCl_3_): δ 151.3, 146.4, 146.2, 144.0, 141.6, 138.3, 137.2, 132.9, 132.4, 129.91, 129.86, 129.1, 128.6, 127.8, 127.4, 127.3, 127.1, 126.8, 126.3, 125.1, 125.0, 123.7, 122.5, 109.9, 108.4, 107.7, 101.5, 47.8, 21.7, 21.5; HRMS (ESI) *m*/*z*: [M + Na]^+^ calcd for C_32_H_27_NO_5_NaS: 560.1502; found: 560.1497.

### *N*-(6-((4-Hydroxynaphthalen-1-yl)­(*p*-tolyl)­methyl)­benzo­[*d*]­[1,3]­dioxol-5-yl)-4-methylbenzenesulfonamide (**4d**)

Purified by silica gel column chromatography eluting with hexane/EA (30:1 to 3:1); 57% yield (31 mg); light yellow powder; mp: 88–89 °C; ^1^H NMR­(400 MHz, CDCl_3_): δ 8.21 (d, *J* = 8.4 Hz, 1H), 7.69 (d, *J* = 8.0 Hz, 2H), 7.45 (t, *J* = 7.6 Hz, 1H), 7.37 (d, *J* = 8.2 Hz, 2H), 7.32–7.30 (m, 1H), 7.17 (d, *J* = 8.4 Hz, 1H), 7.08 (s, 1H), 7.02 (d, *J* = 7.8 Hz, 2H), 6.64 (d, *J* = 7.8 Hz, 1H), 6.58–6.54 (m, 3H), 6.01 (s, 1H), 5.91 (s, 2H), 5.85 (s, 1H), 5.42 (bs, 1H), 5.33 (s, 1H), 2.50 (s, 3H), 2.30 (s, 3H); ^13^C­{^1^H} NMR (100 MHz, CDCl_3_): δ 151.2, 146.4, 146.2, 144.1, 138.6, 137.3, 136.7, 132.9, 132.5, 129.9, 129.6, 129.1, 127.4, 127.2, 126.9, 125.2, 125.0, 123.8, 122.4, 109.9, 108.3, 107.7, 101.5, 47.5, 21.7, 21.0; HRMS (ESI) *m*/*z*: [M + Na]^+^ calcd for C_32_H_27_NO_5_NaS: 560.1502; found: 560.1495.

### *N*-(6-((4-Fluorophenyl)­(4-hydroxynaphthalen-1-yl)­methyl)­benzo­[*d*]­[1,3]­dioxol-5-yl)-4-methylbenzenesulfonamide (**4e**)

Purified by silica gel column chromatography eluting with hexane/EA (30:1 to 3:1); 50% yield (27 mg); yellow powder; mp: 127–128 °C; ^1^H NMR­(400 MHz, CDCl_3_): δ 8.20 (d, *J* = 8.2 Hz, 1H), 7.64 (d, *J* = 7.6 Hz, 2H), 7.44 (t, *J* = 7.4 Hz, 1H), 7.35–7.31 (m, 3H), 7.24–7.23 (m, 1H), 7.00 (s, 1H), 6.85 (t, *J* = 8.1 Hz, 2H), 6.62–6.51 (m, 4H), 5.99 (s, 1H), 5.89 (s, 2H), 5.76 (s, 1H), 5.64 (s, 1H), 5.39 (s, 1H), 2.47 (s, 3H); ^13^C­{^1^H} NMR (100 MHz, CDCl_3_): δ 161.6 (d, *J* = 245 Hz),151.5, 146.5 (d, *J* = 15 Hz), 144.1, 137.6, 137.1, 133.0, 132.3, 130.7 (d, *J* = 8.0 Hz), 129.9, 129.0, 127.4, 127.1, 127.0, 125.3, 125.1, 123.6, 122.6, 115.6 (d, *J* = 21.3 Hz), 109.8, 108.6, 107.7, 101.6, 47.0, 21.6; ^19^F NMR (376 MHz, CDCl_3_): δ −115.4 (s, 1F)_;_ HRMS (ESI) *m*/*z*: [M + Na]^+^ calcd for C_31_H_24_NO_5_FNaS: 564.1251; found: 564.1259.

### *N*-(6-((4-Chlorophenyl)­(4-hydroxynaphthalen-1-yl)­methyl)­benzo­[*d*]­[1,3]­dioxol-5-yl)-4-methylbenzenesulfonamide (**4f**)

Purified by silica gel column chromatography eluting with hexane/EA (30:1 to 3:1); 48% yield (27 mg); white powder; mp: 225–226 °C; ^1^H NMR­(400 MHz, CDCl_3_): δ 8.23 (d, *J* = 8.2 Hz, 1H), 7.66 (d, *J* = 8.2 Hz, 2H), 7.48 (t, *J* = 7.2 Hz, 1H), 7.39–7.35 (m, 3H), 7.27–7.25 (m, 1H), 7.15 (d, *J* = 8.4 Hz, 2H), 7.03 (s, 1H), 6.65 (d, *J* = 7.8 Hz, 1H), 6.55 (d, *J* = 7.8 Hz, 1H), 6.52 (d, *J* = 8.4 Hz, 2H), 6.00 (s, 1H), 5.94 (d, *J* = 2.1 Hz, 2H), 5.74 (s, 1H), 5.46 (s, 1H), 5.38 (s, 1H), 2.51 (s, 3H); ^13^C­{^1^H} NMR (100 MHz, CDCl_3_): δ 151.4, 146.6, 146.5, 144.2, 140.5, 137.0, 132.7, 132.3, 130.5, 129.9, 128.9, 128.7, 127.4, 127.1, 125.4, 125.0, 123.6, 122.6, 109.8, 108.7, 107.7, 101.7, 47.1, 21.7; HRMS (ESI) *m*/*z*: [M + Na]^+^ calcd for C_31_H_24_NO_5_NaSCl: 580.0956; found: 580.0954.

### *N*-(6-((4-Hydroxynaphthalen-1-yl)­(4-methoxyphenyl)­methyl)­benzo­[*d*]­[1,3]­dioxol-5-yl)-4-methylbenzenesulfonamide (**4g**)

Purified by silica gel column chromatography eluting with hexane/EA (30:1 to 3:1); 50% yield (28 mg); light yellow powder; mp: 220–221 °C; ^1^H NMR­(400 MHz, CDCl_3_): δ 8.21 (d, *J* = 8.2 Hz, 1H), 7.68 (d, *J* = 7.8 Hz, 2H), 7.45 (t, *J* = 7.2 Hz, 1H), 7.37 (d, *J* = 7.6 Hz, 2H), 7.30 (t, *J* = 7.5 Hz, 1H), 7.20–7.17 (m, 1H), 7.07 (s, 1H), 6.75 (d, *J* = 8.2 Hz, 2H), 6.64–6.54 (m, 4H), 6.01 (s, 1H), 5.91 (s, 2H), 5.88 (s, 1H), 5.69 (s, 1H), 5.33 (s, 1H), 3.76 (s, 3H), 2.49 (s, 3H); ^113^C­{^1^H} NMR (100 MHz, CDCl_3_): δ 158.4, 151.3, 146.4, 146.2, 144.1, 137.2, 133.6, 133.0, 132.4, 130.2, 129.9, 129.5, 127.4, 127.2, 126.8, 125.1, 125.0, 123.8, 122.4, 114.2, 109.8, 108.3, 107.7, 101.5, 55.2, 47.1, 21.7; HRMS (ESI) *m*/*z*: [M + Na]^+^ calcd for C_32_H_27_NO_6_NaS: 576.1451; found: 576.1458.

### *N*-(6-((4-Hydroxynaphthalen-1-yl)­(naphthalen-1-yl)­methyl)­benzo­[*d*]­[1,3]­dioxol-5-yl)-4-methylbenzenesulfonamide (**4h**)

Purified by silica gel column chromatography eluting with hexane/EA (30:1 to 3:1); 59% yield (34 mg); light yellow powder; mp: 223–224 °C; ^1^H NMR­(400 MHz, acetone-d6): δ 9.06 (s, 1H), 8.33 (d, *J* = 8.0 Hz, 1H), 7.94 (d, *J* = 8.0 Hz, 1H), 7.86–7.81 (m, 3H), 7.69 (d, *J* = 8.2 Hz, 2H), 7.56 (s, 1H), 7.48 (q, *J* = 7.1 Hz, 2H), 7.42–7.38 (m, 4H), 7.31 (t, *J* = 7.7 Hz, 1H), 7.04 (s, 1H), 6.80 (d, *J* = 7.1 Hz, 1H), 6.74 (d, *J* = 7.8 Hz, 1H), 6.65 (s, 1H), 6.57 (d, *J* = 7.8 Hz, 1H), 6.07 (s, 1H), 5.91 (s, 2H), 2.44 (s, 3H); ^13^C­{^1^H} NMR (100 MHz, acetone-d6): δ 153.5, 147.4, 147.3, 144.7, 140.4, 139.3, 136.1, 135.1, 133.7, 132.7, 130.7, 130.4, 129.5, 128.9, 128.6, 128.4, 128.2, 128.1, 127.5, 127.1, 126.6, 126.2, 125.5, 125.2, 125.0, 123.6, 110.6, 109.1, 108.2, 102.7, 44.9, 21.6; HRMS (ESI) *m*/*z*: [M + Na]^+^ calcd for C_35_H_27_NO_5_NaS: 596.1502; found: 596.1495.

### *N*-(6-(1-(4-Hydroxynaphthalen-1-yl)­ethyl)­benzo­[*d*]­[1,3]­dioxol-5-yl)-4-methylbenzenesulfonamide (**4i**)

Purified by silica gel column chromatography eluting with hexane/EA (30:1 to 3:1); 62% yield (29 mg); brown powder; mp: 148–149 °C; ^1^H NMR­(400 MHz, CDCl_3_): δ 8.25–8.23 (m, 1H), 7.72–7.70 (m, 1H), 7.55–7.50 (m, 4H), 7.28 (d, *J* = 8.0 Hz, 2H), 6.89 (s, 1H), 6.81 (s, 1H), 6.78 (d, *J* = 7.8 Hz, 1H), 6.64 (d, *J* = 7.8 Hz, 1H), 5.97 (dd, *J* = 8.3, 1.3 Hz, 2H), 5.80 (s, 1H), 5.45 (s, 1H), 4.25 (q, *J* = 7.1 Hz, 1H), 2.45 (s, 3H), 1.37 (d, *J* = 7.0 Hz, 3H); ^13^C­{^1^H} NMR (100 MHz, CDCl_3_): δ 150.8, 146.4, 146.2, 143.8, 136.8, 134.0, 132.6, 131.9, 129.5, 127.3, 127.2, 127.0, 125.2, 124.9, 124.5, 122.8, 122.7, 108.2, 107.9, 107.6, 101.5, 35.0, 21.6, 21.0; HRMS (ESI-triple Q) *m*/*z*: [M + Na]^+^ calcd for C_26_H_23_NO_5_NaS: 484.1189; found: 484.1191.

### *N*-(6-((4-Hydroxynaphthalen-1-yl)­(phenyl)­methyl)­benzo­[*d*]­[1,3]­dioxol-5-yl)­benzenesulfonamide (**4j**)

Purified by silica gel column chromatography eluting with hexane/EA (30:1 to 3:1); 53% yield (27 mg); white powder; mp: 233–234 °C; ^1^H NMR­(400 MHz, acetone-d6): δ 9.03 (s, 1H), 8.30–8.28 (m, 1H), 7.83–7.81 (m, 2H), 7.75–7.72 (m, 3H), 7.65–7.61 (m, 2H), 7.47–7.39 (m, 2H), 7.26–7.19 (m, 3H), 6.86 (d, *J* = 6.9 Hz, 2H), 6.77 (d, *J* = 7.8 Hz, 1H), 6.60 (d, *J* = 7.7 Hz, 2H), 6.27 (s, 1H), 6.16 (s, 1H), 5.95 (s, 2H); ^13^C­{^1^H} NMR (100 MHz, acetone-d6): δ 153.3, 147.4, 144.5, 141.8, 136.5, 133.8, 133.7, 130.2, 130.2, 129.3, 128.5, 128.3, 128.2, 127.3, 127.2, 126.4, 125.3, 123.5, 110.9, 109.3, 108.0, 102.7, 48.0; HRMS (ESI) *m*/*z*: [M + Na]^+^ calcd for C_30_H_23_NO_5_NaS: 532.1189; found: 532.1196.

### 4-Bromo-*N*-(6-((4-hydroxynaphthalen-1-yl)­(phenyl)­methyl)­benzo­[*d*]­[1,3]­dioxol-5-yl)­benzenesulfonamide (**4k**)

Purified by silica gel column chromatography eluting with hexane/EA (30:1 to 3:1); 42% yield (25 mg); white powder; mp: 162–163 °C; ^1^H NMR­(400 MHz, CDCl_3_): δ 8.23 (d, *J* = 8.4 Hz, 1H), 7.71 (d, *J* = 8.6 Hz, 2H), 7.63 (d, *J* = 8.7 Hz, 2H), 7.48 (t, *J* = 7.0 Hz, 1H), 7.38 (t, *J* = 7.7 Hz, 1H), 7.29–7.21 (m, 4H), 7.05 (s, 1H), 6.71–6.67 (m, 2H), 6.64 (d, *J* = 7.8 Hz, 1H), 6.57 (d, *J* = 7.9 Hz, 1H), 6.06 (s, 1H), 5.93 (s, 2H), 5.88 (s, 1H), 5.41 (s, 1H); ^13^C­{^1^H} NMR (100 MHz, CDCl_3_): δ 151.5, 146.6, 146.4, 141.5, 139.0, 132.9, 132.5, 132.4, 129.2, 128.95, 128.89, 128.3, 127.3, 127.1, 126.7, 125.3, 125.1, 123.4, 122.6, 110.1, 108.1, 107.7, 101.6, 48.0; HRMS (ESI) *m*/*z*: [M – H]^−^ calcd for C_30_H_21_NO_5_SBr: 586.0329; found: 586.0320.

### *N*-(6-((4-Hydroxynaphthalen-1-yl)­(phenyl)­methyl)­benzo­[*d*]­[1,3]­dioxol-5-yl)-4-methoxybenzenesulfonamide (**4l**)

Purified by silica gel column chromatography eluting with hexane/EA (30:1 to 3:1); 57% yield (30 mg); brown powder; mp: 108–109 °C; ^1^H NMR­(400 MHz, CDCl_3_): δ 8.22 (d, *J* = 8.2 Hz, 1H), 7.72 (d, *J* = 6.9 Hz, 2H), 7.46–7.42 (m, 1H), 7.34–7.30 (m, 1H), 7.26–7.20 (m, 4H), 7.06 (s, 1H), 7.02 (d, *J* = 6.9 Hz, 2H), 6.74–6.71 (m, 2H), 6.63 (d, *J* = 7.8 Hz, 1H), 6.56 (d, *J* = 7.8 Hz, 1H), 6.04 (s, 1H), 5.90 (s, 2H), 5.87 (s, 1H), 5.84 (s, 1H), 5.48 (s, 1H), 3.90 (s, 3H); ^13^C­{^1^H} NMR (100 MHz, CDCl_3_): δ 163.3, 151.4, 146.5, 146.2, 141.8, 132.8, 132.4, 131.7, 129.5, 129.3, 129.1, 128.8, 127.32, 127.27, 127.0, 126.9, 125.1, 125.0, 123.7, 122.5, 114.4, 109.9, 108.2, 107.7, 101.5, 55.7, 47.9; HRMS (ESI) *m*/*z*: [M + Na]^+^ calcd for C_31_H_25_NO_6_NaS: 562.1295; found: 562.1300.

### *N*-(6-((4-Hydroxynaphthalen-1-yl)­(phenyl)­methyl)­benzo­[*d*]­[1,3]­dioxol-5-yl)-2,4,6-trimethylbenzenesulfonamide (**4m**)

Purified by silica gel column chromatography eluting with hexane/EA (30:1 to 3:1); 63% yield (35 mg); brown powder; mp: 142–143 °C; ^1^H NMR­(400 MHz, CDCl_3_): δ 8.21 (d, *J* = 8.4 Hz, 1H), 7.45 (t, *J* = 7.5 Hz, 1H), 7.37 (d, *J* = 8.2 Hz, 1H), 7.31 (d, *J* = 7.0 Hz, 1H), 7.26–7.22 (m, 3H), 7.01 (s, 2H), 6.87 (s, 1H), 6.77–6.75 (m, 2H), 6.64 (d, *J* = 7.8 Hz, 1H), 6.53 (d, *J* = 7.8 Hz, 1H), 6.06 (s, 1H), 5.92 (s, 1H), 5.89 (s, 2H), 5.72 (s, 1H), 5.46 (s, 1H), 2.40 (s, 6H), 2.35 (s, 3H); ^13^C­{^1^H} NMR (100 MHz, CDCl_3_): δ 151.1, 146.35, 146.26, 142.7, 142.2, 139.3, 134.8, 133.7, 132.6, 132.3, 130.0, 129.4, 128.8, 127.2, 126.9, 126.8, 125.1, 124.9, 124.3, 122.2, 110.0, 109.0, 107.6, 101.5, 47.9, 23.7, 21.0; HRMS (ESI) *m*/*z*: [M + Na]^+^ calcd for C_33_H_29_NO_5_NaS: 574.1659; found: 574.1667.

### *N*-(2-((4-Hydroxynaphthalen-1-yl)­(phenyl)­methyl)-5-methoxyphenyl)-4-methylbenzenesulfonamide (**4n**)

Purified by silica gel column chromatography eluting with hexane/EA (30:1 to 3:1); 46% yield (23 mg); dark yellow powder; mp: 157–158 °C; ^1^H NMR­(400 MHz, CDCl_3_): δ 8.23 (d, *J* = 8.4 Hz, 1H), 7.62 (d, *J* = 8.2 Hz, 2H), 7.46 (t, *J* = 7.1 Hz, 1H), 7.33 (d, *J* = 8.2 Hz, 2H), 7.29 (dd, *J* = 6.7, 1.1 Hz, 1H), 7.24–7.22 (m, 4H), 7.17 (d, *J* = 2.7 Hz, 1H), 6.73–6.71 (m, 2H), 6.64 (d, *J* = 7.8 Hz, 1H), 6.57–6.54 (m, 2H), 6.41 (d, *J* = 8.6 Hz, 1H), 6.07 (s, 1H), 5.56 (s, 1H), 5.42 (s, 1H), 3.78 (s, 3H), 2.48 (s, 3H); ^13^C­{^1^H} NMR (100 MHz, CDCl_3_): δ 158.8, 151.3, 144.1, 141.8, 137.0, 134.8, 132.4, 130.9, 129.8, 129.3, 129.2, 128.8, 127.4, 127.3, 127.0, 126.9, 125.1, 125.0, 123.7, 122.5, 111.9, 110.7, 107.7, 55.4, 47.5, 21.6; HRMS (ESI) *m*/*z*: [M + Na]^+^ calcd for C_31_H_27_NO_4_NaS: 532.1553; found: 532.1558.

### *N*-(2-((4-Hydroxynaphthalen-1-yl)­(phenyl)­methyl)-4,5-dimethoxyphenyl)-4-methylbenzenesulfonamide (**4o**)

Purified by silica gel column chromatography eluting with hexane/EA (30:1 to 3:1); 62% yield (34 mg); brown powder; mp: 111–112 °C; ^1^H NMR­(400 MHz, CDCl_3_): δ 8.23 (d, *J* = 8.0 Hz, 1H), 7.65 (d, *J* = 8.4 Hz, 2H), 7.47–7.43 (m, 1H), 7.36 (d, *J* = 8.0 Hz, 2H), 7.31 (td, *J* = 7.6, 1.2 Hz, 1H), 7.24–7.19 (m, 5H), 7.11 (s, 1H), 6.65–6.61 (m, 3H), 6.54 (d, *J* = 7.8 Hz, 1H), 6.01 (s, 1H), 5.85 (s, 1H), 5.38 (s, 1H), 3.87 (s, 3H), 3.43 (s, 3H), 2.50 (s, 3H); ^13^C­{^1^H} NMR (100 MHz, CDCl_3_): δ 151.4, 147.7, 147.2, 144.1, 141.9, 137.0, 132.4, 131.2, 129.8, 129.2, 129.1, 128.6, 127.5, 127.2, 126.9, 126.8, 126.4, 125.1, 125.0, 123.8, 122.5, 113.0, 110.9, 107.7, 56.0, 55.6, 47.6, 21.6; HRMS (ESI) *m*/*z*: [M + Na]^+^ calcd for C_32_H_29_NO_5_NaS: 562.1659; found: 562.1667.

### *N*-(6-((6-Bromo-4-hydroxynaphthalen-1-yl)­(phenyl)­methyl)­benzo­[*d*]­[1,3]­dioxol-5-yl)-4-methylbenzenesulfonamide (**4p**)

Purified by silica gel column chromatography eluting with hexane/EA (30:1 to 3:1); 38% yield (23 mg); white powder; mp: 111–112 °C; ^1^H NMR­(400 MHz, CDCl_3_): δ 8.39 (d, *J* = 2.0 Hz, 1H), 7.69 (d, *J* = 8.3 Hz, 2H), 7.38–7.33 (m, 3H), 7.24–7.22 (m, 3H), 7.04 (d, *J* = 9.2 Hz, 1H), 7.01 (s, 1H), 6.69–6.63 (m, 3H), 6.56 (d, *J* = 7.9 Hz, 1H), 5.99 (s, 1H), 5.91 (s, 2H), 5.88 (s, 1H), 5.82 (s, 1H), 5.40 (s, 1H), 2.50 (s, 3H); ^13^C­{^1^H} NMR (100 MHz, CDCl_3_): δ 150.5, 146.6, 146.4, 144.2, 141.4, 137.1, 132.8, 130.9, 130.2, 129.9, 129.5, 129.2, 128.9, 127.6, 127.4, 127.2, 127.0, 126.3, 125.6, 125.1, 119.4, 109.8, 108.6, 108.5, 101.6, 47.9, 21.6; HRMS (ESI) *m*/*z*: [M + Na]^+^ calcd for C_31_H_24_NO_5_NaSBr: 624.0451; found: 624.0443.

### *N*-(2-((6-Bromo-4-hydroxynaphthalen-1-yl)­(phenyl)­methyl)-4,5-dimethoxyphenyl)-4-methylbenzenesulfonamide (**4q**)

Purified by silica gel column chromatography eluting with hexane/EA (30:1 to 3:1); 37% yield (23 mg); white powder; mp: 111–112 °C; ^1^H NMR (400 MHz, CDCl_3_): δ 8.40 (d, *J* = 2.4 Hz, 1H), 7.65 (d, *J* = 8.0 Hz, 2H), 7.37–7.33 (m, 3H), 7.24–7.21 (m, 3H), 7.06–7.04 (m, 2H), 6.66–6.63 (m, 3H), 6.54 (d, *J* = 7.8 Hz, 1H), 6.16 (s, 1H), 5.98 (s, 1H), 5.82 (s, 1H), 5.37 (s, 1H), 3.85 (s, 3H), 3.43 (s, 3H), 2.50 (s, 3H); ^13^C­{^1^H} NMR (100 MHz, CDCl_3_): δ 150.6, 147.7, 147.4, 144.2, 141.6, 137.1, 131.0, 130.9, 130.2, 129.8, 129.4, 129.2, 128.8, 127.6, 127.5, 127.1, 126.3, 125.6, 125.1, 119.4, 112.8, 111.0, 108.6, 56.0, 55.6, 47.6, 21.6; HRMS (ESI) *m*/*z*: [M + Na]^+^ calcd for C_32_H_28_NO_5_NaSBr: 640.0764; found: 640.0757.

### 2-((6-((4-Methylphenyl)­sulfonamido)­benzo­[*d*]­[1,3]­dioxol-5-yl)­(phenyl)­methyl)­naphthalen-1-yl 4-Bromobenzoate (**6**)

In a 7 mL glass vial, **3a** (120 mg, 0.23 mmol) was dissolved in 2.0 mL of CH_2_Cl_2_, and then, 4-bromobenzoyl chloride (101 mg, 0.46 mmol), pyridine (0.1 mL, 1.24 mmol), and DMAP (3 mg, 0.023 mmol) were added sequentially. The reaction was stirred at room temperature for 1h, and then, the residue was diluted with 1 M HCl, and the aqueous solution was extracted with CH_2_Cl_2_. The combined organic layers were dried over Na_2_SO_4_, and the solvent was removed in vacuo. The residue was purified by silica gel column chromatography eluting with hexane/EA (10:1 to 3:1) to afford the product **6**.

142 mg (90% yield); white powder; mp: 210–211 °C; ^1^H NMR (400 MHz, CDCl_3_): δ 7.94–7.92 (m, 2H), 7.88–7.85 (m, 1H), 7.72–7.66 (m, 4H), 7.53–7.41 (m, 4H), 7.30 (s, 1H), 7.18–7.10 (m, 4H), 6.88–6.63 (m, 4H), 6.27–6.23 (m, 2H), 5.97–5.88 (m, 2H), 4.97 (s, 1H), 2.32 (s, 3H); ^13^C­{^1^H} NMR (100 MHz, CDCl_3_): δ 165.2, 146.7, 145.9, 144.5, 143.3, 141.7, 136.6, 133.9, 132.1, 129.5, 129.2, 128.8, 128.3, 127.9, 127.5, 127.2, 126.9, 126.7, 125.9, 121.1, 109.8, 107.7, 101.6, 46.0, 21.4; HRMS (ESI) *m*/*z*: [M + Na]^+^ calcd for C_38_H_28_BrNO_6_NaS: 728.0713; found: 728.0723.

### 4-((6-((4-Methylphenyl)­sulfonamido)­benzo­[*d*]­[1,3]­dioxol-5-yl)­(phenyl)­methyl)­naphthalen-1-yl 4-Bromobenzoate (**7**)

In a 7 mL glass vial, **4a** (73 mg, 0.14 mmol) was dissolved in 2.0 mL of CH_2_Cl_2_, and then, 4-bromobenzoyl chloride (61 mg, 0.28 mmol), pyridine (0.1 mL, 1.24 mmol), and DMAP (3 mg, 0.023 mmol) were added sequentially. The reaction was stirred at room temperature for 1 h, and then, the residue was diluted with 1 M HCl, and the aqueous solution was extracted with CH_2_Cl_2_. The combined organic layers were dried over Na_2_SO_4_, and the solvent was removed in vacuo. The residue was purified by silica gel column chromatography eluting with hexane/EA (10:1 to 3:1) to afford the product **7**.

42 mg (42% yield); white powder; mp: 224–225 °C; ^1^H NMR (400 MHz, CDCl_3_): δ 8.18 (d, *J* = 8.8 Hz, 2H), 7.92 (d, *J* = 8.4 Hz, 1H), 7.74–7.71 (m, 4H), 7.50–7.46 (m, 1H), 7.38 (d, *J* = 8.1 Hz, 2H), 7.35–7.34 (m, 2H), 7.29–7.25 (m, 3H), 7.21 (d, *J* = 8.0 Hz, 1H), 7.00 (s, 1H), 6.81–6.77 (m, 3H), 6.06 (s, 1H), 5.92 (s, 2H), 5.84 (s, 1H), 5.68 (s, 1H), 2.48 (s, 3H); ^13^C­{^1^H} NMR (100 MHz, CDCl_3_): δ 164.4, 146.7, 146.5, 146.2, 144.1, 141.4, 137.4, 135.8, 132.9, 132.6, 132.2, 131.8, 129.9, 129.4, 129.2, 129.0, 128.1, 127.5, 127.3, 127.1, 127.04, 126.99, 126.5, 124.4, 121.8, 117.4, 109.9, 108.6, 101.6, 48.1, 21.6; HRMS (ESI) *m*/*z*: [M + Na]^+^ calcd for C_38_H_28_BrNO_6_NaS: 728.0713; found: 728.0717.

## Supplementary Material



## Data Availability

The data underlying this study are available in the published article and its Supporting Information.

## Notes

General information, X-ray analysis data, computational methods, description of computational simulations, Cartesian coordinates, and NMR spectra of all compounds (PDF)
