# Supplementary material for: Solvent-Controlled Regiodivergent Friedel–Crafts Reactions of 1‑Naphthol with In Situ Generated Aza‑o‑Quinone Methides
Source: J Org Chem. 2025 Jul 18;90(30):10846–57. doi: 10.1021/acs.joc.5c01250 (PMC12322957; doi:10.1021/acs.joc.5c01250)
Supplement: Supplementary file 1 [file jo5c01250_si_001.pdf]

Supporting Information

Solvent-controlled Regiodivergent Friedel-Crafts Reactions of 1-Naphthol with *in situ* Generated Aza-*o*-quinone Methides

Si-Kai Liu [a], Salha Alotaibi[b], Jen-Yu Kuan[a], Theo P Gonçalves[b], Kuo-Wei Huang*[b] and Jeng-Liang Han*[a]

[a] *Department of Chemistry, National Chung Hsing University, Taichung City, 40227, Taiwan.*

[b] *Physical Science and Engineering Division and Center for Renewable Energy and Storage Technologies, King Abdullah University of Science and Technology, Thuwal23955-6900, Saudi Arabia*

E-mail: jlhan@nchu.edu.tw

kuowei.huang@kaust.edu.sa

Table of Contents:

1. **General Experimental Details……………………………………..…....S2**
2. **Starting Materials…………………..………..………………………….....S3**
3. **Computational Methods……………………………………………………….S9**
4. **Description of Computational Simulations…………………………………….S11**
5. **Cartesian Coordinates……………………………………………………..S26**
6. **References…………………………………………………………………S527**
7. **X-Ray Analysis Data……………………….……………………………...S529**
8. **Copies of NMR Spectra of Products……………………………………S557**

1. General Experimental Details

All commercially available reagents were used without further purification unless otherwise stated. All reaction solvents were purified before use. Proton nuclear magnetic resonance (^{1}H NMR) spectra were recorded on a commercial instrument at 400 MHz. Carbon-13 nuclear magnetic resonance (^{13}C{^{1}H } NMR) spectra were recorded at 100 MHz. The proton signal for residual non-deuterated solvent (δ 7.26 for $CHCl_3$) was used as an internal reference for ^{1}H NMR spectra. For ^{13}C{^{1}H} NMR spectra, chemical shifts are reported relative to the δ 77.0 resonance of $CHCl_3$. Coupling constants are reported in Hz. Melting points were determined on a BUCHI B-545 melting point apparatus and are uncorrected. High resolution mass spectra were recorded on a Thermo Fisher Scientific LTQ Orbitrap XL mass spectrometer. The single crystal was measured by Bruker D8 VENTURE X-ray Single Crystal Diffractometer. Analytical thin-layer chromatography (TLC) was performed on silica gel 60 F254 pre-coated plates with visualization under UV light. Column chromatography was generally performed using 40-63 μm (230-400 mesh) silica gel, typically using a 50-100:1 weight ratio of silica gel to crude product.

2. Starting Materials

2.1 Preparation of 2-(tosylmethyl)anilines 1

2-(tosylmethyl)anilines **1** were prepared according to known procedures. [1,2]

General procedure for the synthesis of 1

In a 50 mL double-necked round-bottom flask, nitrobenzaldehydes **S1** (1.70 mmol, 1.0 equiv) was dissolved in 12 mL methanol. Iron powder (0.33 g, 5.95 mmole), ammonium chloride (0.45 g, 8.41 mmole) and 10 mL distilled water were added respectively. The reaction mixture was placed in an oil bath and heated to 90 °C for 2 hours. After the reaction was completed (confirmed by TLC), the resulting reaction mixture was filtered with Celite and then concentrated under reduced pressure to remove methanol. The mixture was diluted with water and was extracted by DCM. The organic layer was dried over Na_2SO_4, filtered, and concentrated to afford crude anilines **S2**.

The mixture of **S2** (1.70 mmol, 1.0 equiv) and pyridine (0.30 mL, 3.8 mmol, 2.2 equiv.) in DCM (3 mL) was slowly added a solution of tosyl chloride (0.65 g, 3.4 mmol, 2.0 equiv.) in DCM (2 mL). The mixture was stirred at 40°C (oil bath) for 8h. The resulting reaction mixture was quenched and washed with 1 N of aqueous HCl, saturated aqueous $NaHCO_3$, and brine. The organic layer was dried over Na_2SO_4, filtered, concentrated and purified by flash column (DCM) to provide **S3**.

In a 50 mL double-necked round-bottom flask, magnesium (0.21 g, 8.5 mmol, 5.0 equiv.) and a crytal of iodine was added ether (3 mL). The mixture was heated to reflux (oil bath) and bromobenzenes (8.5 mmol, 5.0 equiv.) was added slowly. After 2h, the reaction mixture was cooled to 0°C and **S3** (1.70 mmol, 1.0 equiv) in anhydrous THF (3 mL) was added. After the reaction was completed (confirmed by TLC), The mixture was quenched with sat. $NH_4Cl_{(aq)}$ and was extracted by DCM. The organic layer was dried over Na_2SO_4, filtered, and concentrated to afford crude **S4**.

In a 25 mL double-necked round-bottom flask, sodium *p*-toluenesulfinate (0.34 g, 2.1 mmol, 1.2 equiv.) and *p*-toluenesulfonic acid (0.6 g, 3.40 mmol, 2.0 equiv.) were dissolve in DCM (10 mL). Then a solution of crude **S4** in DCM (5 mL) was added and stirred at rt for 2h. After the reaction was completed (confirmed by TLC), The mixture was quenched with sat. $NaHCO_{3(aq)}$ and was extracted by DCM. The organic layer was dried over Na_2SO_4, filtered, concentrated and purified by flash column (DCM) to afford **1**.

Scheme S1. 2-(tosylmethyl)anilines **1** were prepared according to the following routes.[1,2]

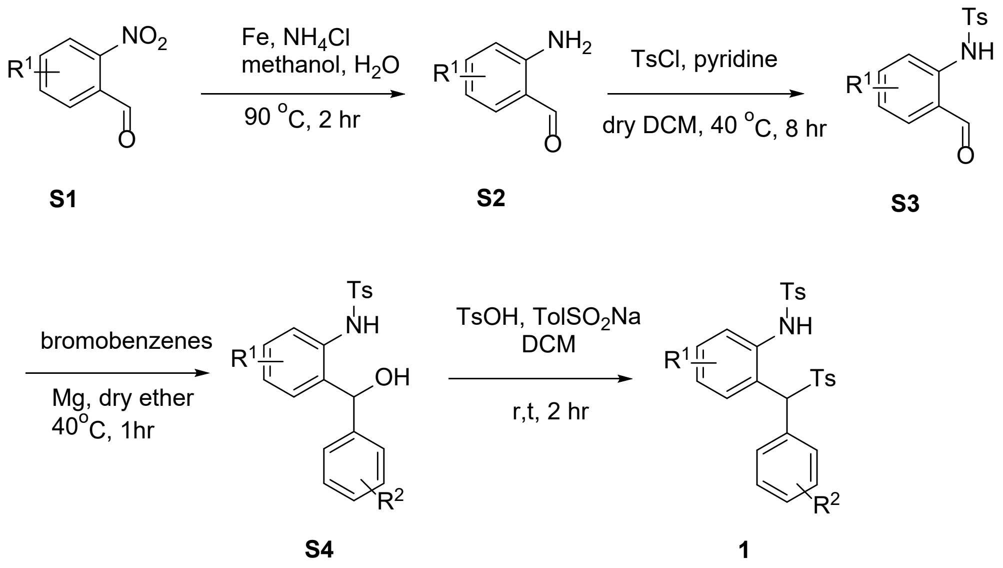

Scheme S2. 2-(tosylmethyl)anilines **1** were used in this work.

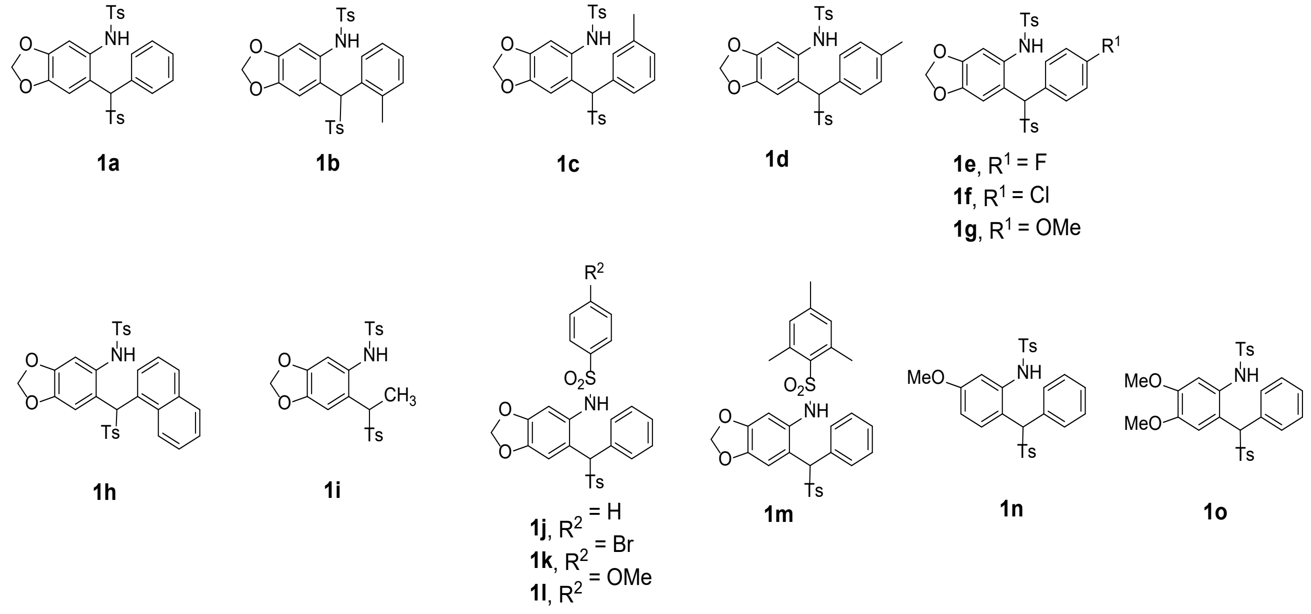

4-Methyl-*N*-(6-(phenyl(tosyl)methyl)benzo[*d*][1,3]dioxol-5-yl)benzenesulfonamide (1a)

Purified by silica gel column chromatography eluting with CH_2Cl_2; 70% yield (375 mg); Yellow powder; mp: 188-189 °C; ^{1}H NMR(400 MHz, $CDCl_3$): δ 7.72 (d, *J* = 8.2 Hz, 2H), 7.57 (d, *J* = 8.2 Hz, 2H), 7.34 (s, 1H), 7.29 (d, *J* = 8.0 Hz, 2H), 7.25-7.16 (m, 7H), 7.05 (s, 1H), 6.56 (s, 1H), 5.94 (d, *J* = 1.4 Hz, 1H), 5.92 (d, *J* = 1.4 Hz, 1H), 5.72 (s, 1H), 2.43 (s, 3H), 2.35 (s, 3H); ^{13}C{^{1}H} NMR (100 MHz, $CDCl_3$): δ 148.2, 147.4, 144.9, 144.1, 137.6, 134.9, 132.6, 130.0, 129.6, 129.5, 128.9, 128.7, 127.4, 123.4, 110.4, 109.6, 102.1, 70.0, 21.7, 21.7; HRMS (ESI) *m/z*: $[M+Na]^+$ calcd for $C_{28}H_{25}NO_6NaS_2$: 558.1016; found: 558.1015.

4-Methyl-*N*-(6-(*o*-tolyl(tosyl)methyl)benzo[*d*][1,3]dioxol-5-yl)benzenesulfonamide (1b)

Purified by silica gel column chromatography eluting with CH_2Cl_2; 73% yield (398 mg); White powder; mp: 201-202 °C; ^{1}H NMR(400 MHz, $CDCl_3$): δ 8.10 (d, *J* = 7.3 Hz, 1H), 7.84 (d, *J* = 8.2 Hz, 2H), 7.70 (s, 1H), 7.64 (d, *J* = 8.4 Hz, 2H), 7.36 (d, *J* = 8.0 Hz, 2H), 7.25-7.15 (m, 5H), 6.99 (d, *J* = 7.6 Hz, 1H), 6.44 (s, 1H), 6.25 (s, 1H), 5.92 (dd, *J* = 8.2, 1.4 Hz, 2H), 2.47 (s, 3H), 2.36 (s, 3H), 1.99 (s, 3H); ^{13}C{^{1}H} NMR (100 MHz, $CDCl_3$): δ 148.1, 146.8, 144.8, 143.9, 138.2, 135.2, 130.9, 130.6, 129.9, 129.5, 128.7, 128.6, 127.2, 125.9, 110.6, 107.3, 102.0, 65.8, 21.6, 19.6; HRMS (ESI) *m/z*: $[M+Na]^+$ calcd for $C_{29}H_{27}NO_6NaS_2$: 572.1172; found: 572.1178.

4-Methyl-*N*-(6-(*m*-tolyl(tosyl)methyl)benzo[*d*][1,3]dioxol-5-yl)benzene sulfonamide (1c)

Purified by silica gel column chromatography eluting with CH_2Cl_2; 72% yield (394 mg); White powder; mp: 102-103 °C; ^{1}H NMR(400 MHz, $CDCl_3$): δ 8.10 (d, *J* = 7.7 Hz, 1H), 7.83 (d, *J* = 8.2 Hz, 2H), 7.70 (s, 1H), 7.64 (d, *J* = 8.2 Hz, 2H), 7.36 (d, *J* = 8.2 Hz, 2H), 7.25-7.15 (m, 5H), 6.98 (d, *J* = 7.4 Hz, 1H), 6.44 (s, 1H), 6.25 (s, 1H), 5.92 (dd, *J* = 8.2, 1.3 Hz, 2H), 2.47 (s, 3H), 2.36 (s, 3H), 1.99 (s, 3H); ^{13}C{^{1}H} NMR (100 MHz, $CDCl_3$): δ 148.1, 147.2, 144.8, 143.8, 138.4, 137.6, 134.8, 132.2, 130.5, 129.9, 129.5, 129.4, 129.4, 128.8, 128.4, 127.3, 126.8, 123.2, 110.3, 109.4, 102.0, 69.9, 21.6, 21.4; HRMS (ESI) *m/z*: $[M+Na]^+$ calcd for $C_{29}H_{27}NO_6NaS_2$: 572.1172; found: 572.1171.

4-Methyl-*N*-(6-(*p*-tolyl(tosyl)methyl)benzo[*d*][1,3]dioxol-5-yl)benzenesulfonamide (1d)

Purified by silica gel column chromatography eluting with CH_2Cl_2; 74% yield (405 mg); Yellow powder; mp: 183-184 °C; ^{1}H NMR(400 MHz, $CDCl_3$): δ 7.72 (d, *J* = 8.2 Hz, 2H), 7.58 (d, *J* = 8.4 Hz, 2H), 7.39 (s, 1H), 7.29 (d, *J* = 8.1 Hz, 2H), 7.19 (d, *J* = 8.1 Hz, 2H), 7.10 (d, *J* = 8.1 Hz, 2H), 7.05 (s, 1H), 7.01 (d, *J* = 8.1 Hz, 2H), 6.59 (s, 1H), 5.93 (dd, *J* = 9.0, 1.2 Hz, 2H), 5.65 (s, 1H), 2.44 (s, 3H), 2.36 (s, 3H), 2.27 (s, 3H); $^{13}C\{^1H\}$ NMR (100 MHz, $CDCl_3$): δ 148.0, 147.2, 144.7, 143.9, 138.5, 137.5, 134.9, 129.9, 129.7, 129.5, 129.3, 128.7, 127.2, 123.3, 110.2, 109.4, 102.0, 69.6, 21.6, 21.5, 21.1; HRMS (ESI) *m/z*: $[M+Na]^+$ calcd for $C_{29}H_{27}NO_6NaS_2$: 572.1172; found: 572.1171.

***N*-(6-((4-fluorophenyl)(tosyl)methyl)benzo[*d*][1,3]dioxol-5-yl)-4-methylbenzene sulfonamide (1e)**

Purified by silica gel column chromatography eluting with CH_2Cl_2; 72% yield (398 mg); White powder; mp: 192-193 °C; ^{1}H NMR(400 MHz, $CDCl_3$): δ 7.71 (d, *J* = 8.2 Hz, 2H), 7.60 (d, *J* = 8.2 Hz, 2H), 7.35-7.29 (m, 4H), 7.21 (d, *J* = 8.1 Hz, 2H), 7.16 (s, 1H), 7.03 (s, 1H), 6.92 (t, *J* = 8.6 Hz, 2H), 6.45 (s, 1H), 5.95 (dd, *J* = 7.3, 1.1 Hz, 2H), 5.86 (s, 1H), 2.44 (s, 3H), 2.38 (s, 3H); $^{13}C\{^1H\}$ NMR (100 MHz, $CDCl_3$): δ 162.6 (d, *J* = 247.7 Hz) ,δ 148.1, 147.5, 145.0, 144.0, 137.2, 134.7, 131.8, 131.7, 129.9, 129.6, 129.3, 128.7, 128.4, 127.4, 123.7, 115.6 (d, *J* = 21.5 Hz), 110.0, 109.6, 102.1, 68.9, 21.63, 21.56;^{19}F NMR (376 MHZ, $CDCl_3$): δ -112.3 (s, 1F); HRMS (ESI) *m/z*: $[M+Na]^+$ calcd for $C_{28}H_{24}NO_6FNaS_2$: 576.0921; found: 576.0918.

***N*-(6-((4-chlorophenyl)(tosyl)methyl)benzo[*d*][1,3]dioxol-5-yl)-4-methylbenzenesulfonamide (1f)**

Purified by silica gel column chromatography eluting with CH_2Cl_2; 52% yield (297 mg); White powder; mp: 203-204 °C; ^{1}H NMR(400 MHz, $CDCl_3$): δ 7.71 (d, *J* = 8.4 Hz, 2H), 7.61 (d, *J* = 8.2 Hz, 2H), 7.31-7.28 (m, 4H), 7.24-7.20 (m, 4H), 7.11 (s, 1H), 7.00 (s, 1H), 6.44 (s, 1H), 5.95 (dd, *J* = 7.4, 1.4 Hz, 2H), 5.85 (s, 1H), 2.45 (s, 3H), 2.39 (s, 3H); $^{13}C\{^1H\}$ NMR (100 MHz, $CDCl_3$): δ 148.2, 147.5, 145.1, 144.1, 137.2, 134.7, 134.7, 131.2, 131.1, 129.9, 129.7, 129.3, 128.9, 128.7, 127.4, 123.5, 110.1, 109.6, 102.1, 68.9, 21.7, 21.6; HRMS (ESI-triple Q) *m/z*: $[M+Na]^+$ calcd for $C_{28}H_{24}NO_6NaS_2Cl$:

592.0626; found: 592.0633.

***N*-(6-((4-methoxyphenyl)(tosyl)methyl)benzo[*d*][1,3]dioxol-5-yl)-4-methylbenzenesulfonamide (1g)**

Purified by silica gel column chromatography eluting with CH_2Cl_2; 63% yield (353 mg); Yellow powder; mp: 173-174 °C; ^1H NMR(400 MHz, $CDCl_3$): δ 7.72 (d, J = 8.2 Hz, 2H), 7.58 (d, J = 8.2 Hz, 2H), 7.34 (s, 1H), 7.30 (d, J = 8.0 Hz, 2H), 7.20-7.16 (m, 4H), 7.05 (s, 1H), 6.77-6.72 (m, 2H), 6.56 (s, 1H), 5.94 (dd, J = 7.4, 1.4 Hz, 2H), 5.67 (s, 1H), 3.75 (s, 3H), 2.45 (s, 3H), 2.37 (s, 3H); $^{13}C\{^1H\}$ NMR (100 MHz, $CDCl_3$): δ 159.6, 148.0, 147.3, 144.7, 143.9, 137.5, 134.9, 131.2, 129.9, 129.5, 129.3, 128.7, 127.3, 124.3, 123.6, 116.0, 114.8, 114.0, 110.2, 109.4, 102.0, 69.3, 55.2, 21.6, 21.5; HRMS (ESI-triple Q) *m/z*: $[M+Na]^+$ calcd for $C_{29}H_{27}NO_7NaS_2$: 588.1121; found: 588.1130.

4-Methyl-*N*-(6-(naphthalen-1-yl(tosyl)methyl)benzo[*d*][1,3]dioxol-5-yl)benzenesulfonamide (1h)

Purified by silica gel column chromatography eluting with CH_2Cl_2; 53% yield (313 mg); Yellow powder; mp: 240-241 °C; ^1H NMR(400 MHz, $CDCl_3$): δ 8.38 (d, J = 7.3 Hz, 1H), 7.93-7.91 (m, 3H), 7.88-7.85 (m, 2H), 7.77 (d, J = 8.2 Hz, 1H), 7.72 (m, 3H), 7.49 (t, J = 7.8 Hz, 1H), 7.44-7.37 (m, 3H), 7.11 (d, J = 8.1 Hz, 2H), 7.02 (s, 1H), 6.85 (s, 1H), 6.48 (s, 1H), 5.90 (d, J = 1.4 Hz, 1H), 5.84 (d, J = 1.4 Hz, 1H), 2.49 (s, 3H), 2.26 (s, 3H); ^{13}C $\{^1H\}$NMR (100 MHz, $CDCl_3$): δ 148.4, 146.7, 144.8, 144.0, 138.3, 135.0, 133.7, 131.2, 130.5, 130.0, 129.6, 129.5, 128.7, 128.2, 127.6, 127.3, 126.0, 124.6, 122.7, 121.9, 110.6, 107.1, 101.9, 65.3, 21.6, 21.5; HRMS (ESI-triple Q) *m/z*: $[M+Na]^+$ calcd for $C_{32}H_{27}NO_6NaS_2$: 608.1185; found: 608.1175.

4-Methyl-*N*-(6-(1-tosylethyl)benzo[*d*][1,3]dioxol-5-yl)benzenesulfonamide(1i)

Purified by silica gel column chromatography eluting with CH_2Cl_2; 72% yield (341 mg); White powder; mp: 183-184 °C; ^1H NMR(400 MHz, $CDCl_3$): δ 7.63 (d, J = 8.1 Hz, 2H), 7.55 (d, J = 8.1 Hz, 2H), 7.31 (d, J = 8.0 Hz, 2H), 7.27-7.25 (m, 2H), 7.14 (s, 1H), 6.66 (s, 1H), 6.53 (s, 1H), 5.97 (d, J = 3.0 Hz,

2H), 4.25 (q, J = 7.0 Hz, 1H), 2.45 (s, 3H), 2.40 (s, 3H), 1.23 (d, J = 7.0 Hz, 3H); $^{13}C\{^1H\}$ NMR (100 MHz, $CDCl_3$): δ 148.1, 147.2, 145.2, 143.8, 137.1, 133.1, 129.7, 129.7, 129.1, 127.0, 124.0, 109.5, 107.9, 102.0, 59.6, 21.7, 21.5, 14.3; HRMS (ESI-triple Q) *m/z*: $[M+Na]^+$ calcd for $C_{23}H_{23}NO_6NaS_2$: 496.0859; found: 496.0864.

N-(6-(phenyl(tosyl)methyl)benzo[*d*][1,3]dioxol-5-yl)benzenesulfonamide (1j)

Purified by silica gel column chromatography eluting with CH_2Cl_2; 54% yield (282 mg); White powder; mp: 195-196 °C; ^1H NMR(400 MHz, $CDCl_3$): δ 7.88-7.86 (m, 2H), 7.65-7.61 (m, 1H), 7.59 (d, J = 8.4 Hz, 2H), 7.55-7.51 (m, 2H), 7.40 (s, 1H), 7.28-7.19 (m, 7H), 7.06 (s, 1H), 6.54 (s, 1H), 5.94 (dd, J = 9.2, 1.4 Hz, 2H), 5.75 (s, 1H), 2.36 (s, 3H); $^{13}C\{^1H\}$ NMR (100 MHz, $CDCl_3$): 13C-NMR (101 MHz, CHLOROFORM-D) δ 148.1, 147.4, 144.8, 140.4, 134.8, 133.0, 132.4, 129.9, 129.5, 129.3, 129.2, 128.8, 128.6, 127.2, 123.3, 110.3, 109.4, 102.1, 69.9, 21.6; HRMS (ESI-triple Q) *m/z*: $[M+Na]^+$ calcd for $C_{27}H_{23}NO_6NaS_2$: 544.0859; found: 544.0868.

4-Bromo-N-(6-(phenyl(tosyl)methyl)benzo[*d*][1,3]dioxol-5-yl)benzenesulfonamide (1k)

Purified by silica gel column chromatography eluting with CH_2Cl_2; 64% yield (221 mg); Yellow powder; mp: 138-139 °C; ^1H NMR(400 MHz, $CDCl_3$): δ 7.70 (d, J = 8.5 Hz, 2H), 7.64-7.62 (m, 2H), 7.57 (d, J = 8.2 Hz, 2H), 7.51 (s, 1H), 7.26-7.24 (m, 5H), 7.18 (d, J = 8.1 Hz, 2H), 7.09 (s, 1H), 6.57 (s, 1H), 5.95 (d, J = 9.3 Hz, 2H), 5.68 (s, 1H), 2.36 (s, 3H); $^{13}C\{^1H\}$ NMR (100 MHz, $CDCl_3$): δ 148.2, 147.5, 144.9, 139.2, 134.6, 132.5, 132.2, 129.8, 129.5, 128.9, 128.7, 128.0, 123.3, 110.3, 109.4, 102.1, 77.3, 77.0, 76.7, 69.9, 21.6; HRMS (ESI-triple Q) *m/z*: $[M+Na]^+$ calcd for $C_{27}H_{22}NO_6NaS_2Br$: 621.9964; found: 621.9970.

4-Methoxy-N-(6-(phenyl(tosyl)methyl)benzo[*d*][1,3]dioxol-5-yl)benzenesulfonamide (1l)

Purified by silica gel column chromatography eluting with CH_2Cl_2; 63% yield (348 mg); Yellow powder; mp: 185-186 °C; ^1H NMR(400 MHz, $CDCl_3$): δ 7.76 (dd, J = 7.0, 1.9 Hz, 2H), 7.58 (d, J =

8.2 Hz, 2H), 7.32-7.30 (m, 2H), 7.26-7.17 (m, 6H), 7.11 (s, 1H), 6.96-6.94 (m, 2H), 6.52 (s, 1H), 5.92 (d, *J* = 1.2 Hz, 2H), 5.81 (s, 1H), 3.85 (s, 3H), 2.35 (s, 3H); $^{13}C\{^1H\}$ NMR (100 MHz, $CDCl_3$): δ 163.1, 148.0, 147.3, 144.8, 134.8, 132.5, 131.7, 129.9, 129.5, 129.4, 128.7, 128.6, 123.5, 114.4, 110.1, 109.4, 102.0, 69.7, 55.6, 21.6; HRMS (ESI-triple Q) *m/z*: $[M+Na]^+$ calcd for $C_{28}H_{25}NO_7NaS_2$: 574.0965; found: 574.0971.

***N*-(5-methoxy-2-(phenyl(tosyl)methyl)phenyl)-4-methylbenzenesulfonamide (1n)**

Purified by silica gel column chromatography eluting with CH_2Cl_2; 78% yield (401 mg); White powder; mp: 162-163 °C; ^1H NMR(400 MHz, $CDCl_3$): δ 7.79 (s, 1H), 7.72 (d, *J* = 7.8 Hz, 2H), 7.56 (d, *J* = 7.8 Hz, 2H), 7.47 (d, *J* = 8.8 Hz, 1H), 7.27 (d, *J* = 8.0 Hz, 2H), 7.22-7.15 (m, 7H), 6.77-6.71 (m, 2H), 5.64 (s, 1H), 3.66 (s, 3H), 2.42 (s, 3H), 2.35 (s, 3H); $^{13}C\{^1H\}$ NMR (100 MHz, $CDCl_3$): δ 160.1, 144.7, 143.8, 137.4, 136.7, 134.7, 132.4, 131.9, 130.0, 129.8, 129.4, 128.8, 128.5, 127.2, 120.0, 113.5, 113.1, 69.8, 55.3, 21.6, 21.5; HRMS (ESI-triple Q) *m/z*: $[M+Na]^+$ calcd for $C_{28}H_{27}NO_5NaS_2$: 544.1223; found: 544.1223.

***N*-(4,5-dimethoxy-2-(phenyl(tosyl)methyl)phenyl)-4-methylbenzenesulfonamide (1o)**

Purified by silica gel column chromatography eluting with CH_2Cl_2; 75% yield (416 mg); White powder; mp: 95-96 °C; ^1H NMR(400 MHz, $CDCl_3$): δ 7.67 (d, *J* = 8.4 Hz, 2H), 7.55 (d, *J* = 8.4 Hz, 2H), 7.27 (s, 2H), 7.25-7.17 (m, 8H), 7.10 (s, 1H), 6.51 (s, 1H), 5.72 (s, 1H), 3.77 (s, 3H), 3.62 (s, 3H), 2.42 (s, 3H), 2.36 (s, 3H); $^{13}C\{^1H\}$ NMR (100 MHz, $CDCl_3$): δ 149.2, 148.2, 144.7, 143.8, 137.2, 134.9, 132.5, 129.9, 129.7, 129.5, 128.8, 128.6, 128.2, 127.5, 121.7, 112.8, 112.0, 69.7, 55.9, 55.7, 21.6, 21.5; HRMS (ESI-triple Q) *m/z*: $[M+Na]^+$ calcd for $C_{29}H_{29}NO_6NaS_2$: 574.1329; found: 574.1320.

3. Computational Methods

All geometries were calculated at M06L level of theory.[3] The potassium atom was described by SDD basis set, and the rest of the atoms were described by 6-31G(d) basis set (BS1 basis set). The ground

states and transition states geometries were confirmed by the absence or presence of the imaginary frequency. The Gibbs free energy (ΔG_{25^oC}) corrections were calculated at 25ºC (298.15K) and 1 atm. The resulting structures were used for the energy (single points) calculations at M06L/6-31G(d)/SDD level with the incorporation of the solvent effects with acetonitrile (ACN) and toluene (toluene) via SMD implicit solvation model.[4] All geometry optimization and solvent calculations were performed with ultrafine integration grid (*integral(Grid=UltraFine)*) with Gaussian 16 rev.C program.[5] In order to access accurate energies, resulting structures were used for the energy (single points) calculations at TPSS0-D4/def2-TZVPP/def2-SD (BS2 basis set) level of theory with Orca 5.0.4 software in a vacuum.[6-8] The def2-SD effective core potential basis set was used for potassium atoms together with tight SCF criteria and auxiliary basis sets (*TightSCF, def2/J, def2/JK, and def2-SD TightSCF def2-TZVPP Def2-TZVPP/C def2/J def2/JK def2-SD, DEFGRID3*). The reported Gibbs Free Energies in solvent are designated as ΔG_{25^oC}(TPSS0-D4/BS2//M06L/BS1/THF(acetonitrile or toluene)) and are composed of the electronic energy at TPSS0-D4 level and Gibbs free energy corrections with solvent effect from M06L method level. The Mullikan charges were calculated with Gaussian16. The *Hirschfeld* atomic charges and dual descriptor surface were calculated with MultiWFN software.[9] Post-processing visualization was carried out with the ChemCraft software.[10]

General notes for conformational analysis. Ground state and transition state conformers were generated in three different approaches. We use "by hand" generation, systematic search with Spartan(MMFF)[11] and finally conformational analysis with iMTD-sMTD algorithm with GFN2//GFNFF composite method as implanted in CREST and xTB softwares.[12] Conformer–Rotamer Ensemble Sampling Tool (CREST) software uses extensive metadynamic sampling (MTD). Such new developed protocol found to be very efficient conformer search with metal complexes with application to ground and transition states geometries. The CREST conformational search were conducted with applying constraint potential (*force constant=1.5*) to the two carbons of the transition state, allowing rest of the moiety to participate in metadynamic sampling. The generation of the cluster with explicit acetonitrile was created using Quantum Cluster Grow (QCG)protocol implemented in CREST.[13] The

obtained cluster was used for DFT calculations and conformational analysis for the ground and transition state.

General notes for methodology. Studied system show basis set and method dependence but main trends are preserved. The energy barriers (Gibbs Free Energies) obtained at TPSS0-D4/def2-TZVPP/def2-SD were lower than those at M06L/6-31G(d)/SDD. While M06L and TPSS0-D4 provided slightly different energies, the overall picture of selectivity was preserved at both calculation levels. The rigid rotor harmonic oscillator treatment using GoodVibes did not result in diferent outcome, thus it was not applied.[14]

4. Description of Computational Simulations

We analyzed different scenarios and possible pathways to understand the dynamics of the reaction. We also analyzed the formation of the intermediates, charges, and dual descriptors (Figure S1 to Figure S6). Our conclusion points towards different characters of the transition states depending on the solvent. In less coordinating solvents such as toluene, potassium cations prefer to form intermolecular interactions between substrates preferential in *ortho* geometry and dictating this product formation kinetically (Figure S10, Figure S11). The transition state for the *ortho* path in toluene is 8.7 kcal/mol and *para* path is 0.3 kcal/mol (Figure S11). The para intermediate is thermodynamically less favorable in toluene (4.6 kcal/mol) than *para* intermediates (-7.4 kcal/mol). A similar selectivity trend is observed in acetonitrile solvent (Figure S11), favoring *ortho* path with 0.9 kcal/mol free energy barrier and para path is disfavored due to the higher energy (8.8 kcal/mol). Interestingly, if the reaction is computed with an anionic mechanism (Figure S8, Figure S9), energy barriers become lower, and energy difference becomes less remarkable. The *ortho* path has barrier of 0.4-0.5 kcal/mol (toluene/acetonitrile) and para path has barrier of 0.5 kcal/mol (acetonitrile) and it is spontaneous in toluene. However, in coordinating solvents such as acetonitrile, the interactions between solvent and cation are more energetically important. Such solvent-cation aggregate forms a more energetically lower path and favors the para product. We found that up to 4 acetonitrile molecules can interact with

potassium in a transition state from Quantum Cluster Grow with molecular dynamic (MD) simulation. Therefore, the implicit solvation model is inappropriate in accounting for such effects. Additionally, a second potassium cation is required to interact with anionic charge formed on nitrogen during the reaction course. In the experimental scenario, this is likely to be accomplished by excess potassium salt, *i.e.,* phosphate or organic intermediated. The reaction path calculated for two potassiums in the implicit model is resumed in Figure S12 and Figure S13. Regardless of the solvent via the implicit solvation method, the selectivity remains the same. The calculated energy barrier is always lower for *ortho* path (6.8kcal/mol acetonitrile and 6.9kcal/mol toluene) and higher for the *para* path (17.7 kcal/mol acetonitrile and 23.2 kcal/mol toluene). Such results explain the selectivity observed in toluene. As the calculated models did not align with experimental selectivity in acetonitrile, we extended the study to explicit micro-solvation with implicit solvent (explicit-implicit solvent cluster). We found that up to 4 acetonitrile molecules can interact with potassium cation in the transition state by applying Quantum Cluster Grow methodology following the molecular dynamic (MD) simulation. After large conformational analysis (CREST software) we found that the computed mechanism favors the experimentally observed para product (Figure S15). At a higher level of the theory (TPSS0-D4/def2TZVPP SMD(acetonitrile)), the para path has lower energy (1.0 kcal/mol) than ortho path (1.6 kcal/mol) for the lowest conformation. The same trend is reflected at the lower method level (Figure S14) but the energy difference is favoring more the para path.

Therefore, due to the bulkiness of the solvent and its ability to form an interaction with potassium, only an explicit micro-solvation approach can account for the selectivity. In conclusion, due to the lack of strong interaction, the toluene solvent follows the ortho path, where close contact ion-pair can form strong solute interactions. Due to much higher solvent-potassium interactions in acetonitrile, para-path is more favored as potassium prefers to be solvated rather than interact with another part of the solute. Those results align with "innate solvent-mediated chemoselectivity" discussed by Gogoi and Jindal.[15] The acetonitrile-potassium-solute interactions can be rationalized by solvation clusters with tilt angles model as reported by Spångberg and Hermansson.[16] Our approach also included *Quantum Cluster*

Growth (QCG) and explicit solvation method. Unfortunately, in the current software version, we (v3.0) were not able to converge all metadynamic simulations required for explicit solvation Gibbs Free Energies.[13] A new software version was released at the time of the submission.

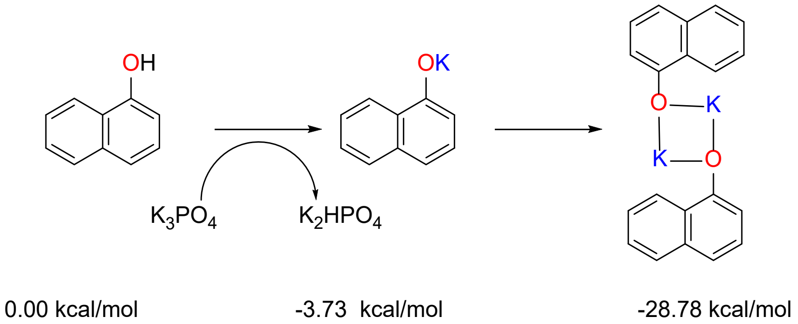

Figure S1. Deprotonation reaction of 1-naphthol to potassium naphthalen-1-olate in vacuum at PBE-D3/6-31G(d).

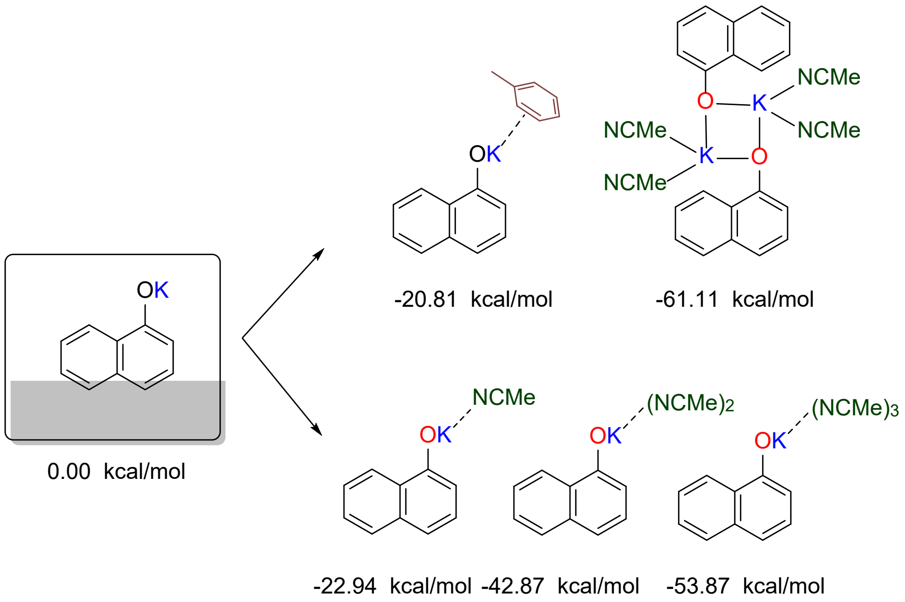

Figure S2. Potassium-1-naphtholate binding with solvent at PBE-D3/6-31G(d).

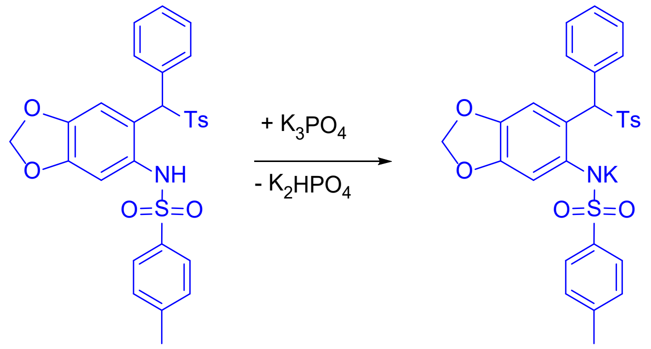

0 kcal/mol   -21.74 kcal/mol

Figure S3. Deprotonation of the benzenesulfonamide at PBE-D3/6-31G(d).

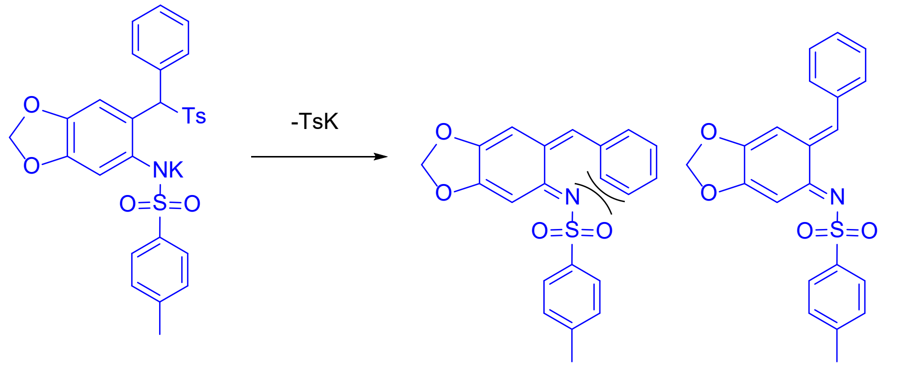

-21.74 kcal/mol

0 kcal/mol   16.57 kcal/mol   14.17 kcal/mol

Figure S4. Elimination of TsK and formation of enamine moiety at PBE-D3/6-31G(d).

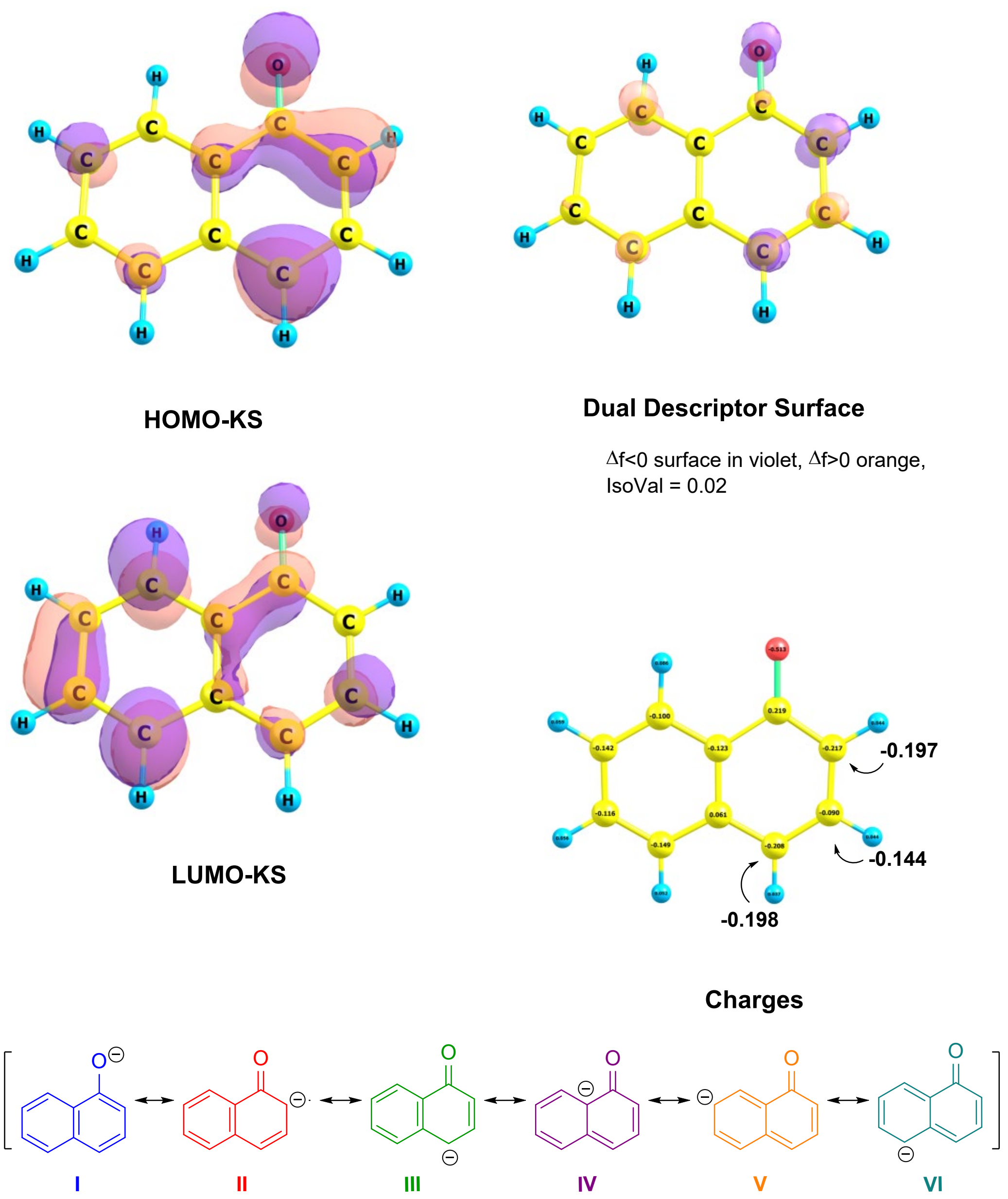

Figure S5. Frontier Kohn-Sham orbitals, dual descriptors, Mulliken charges (black), and *Hirshfeld* atomic *charge (black)* of the naphtholoxy anion.

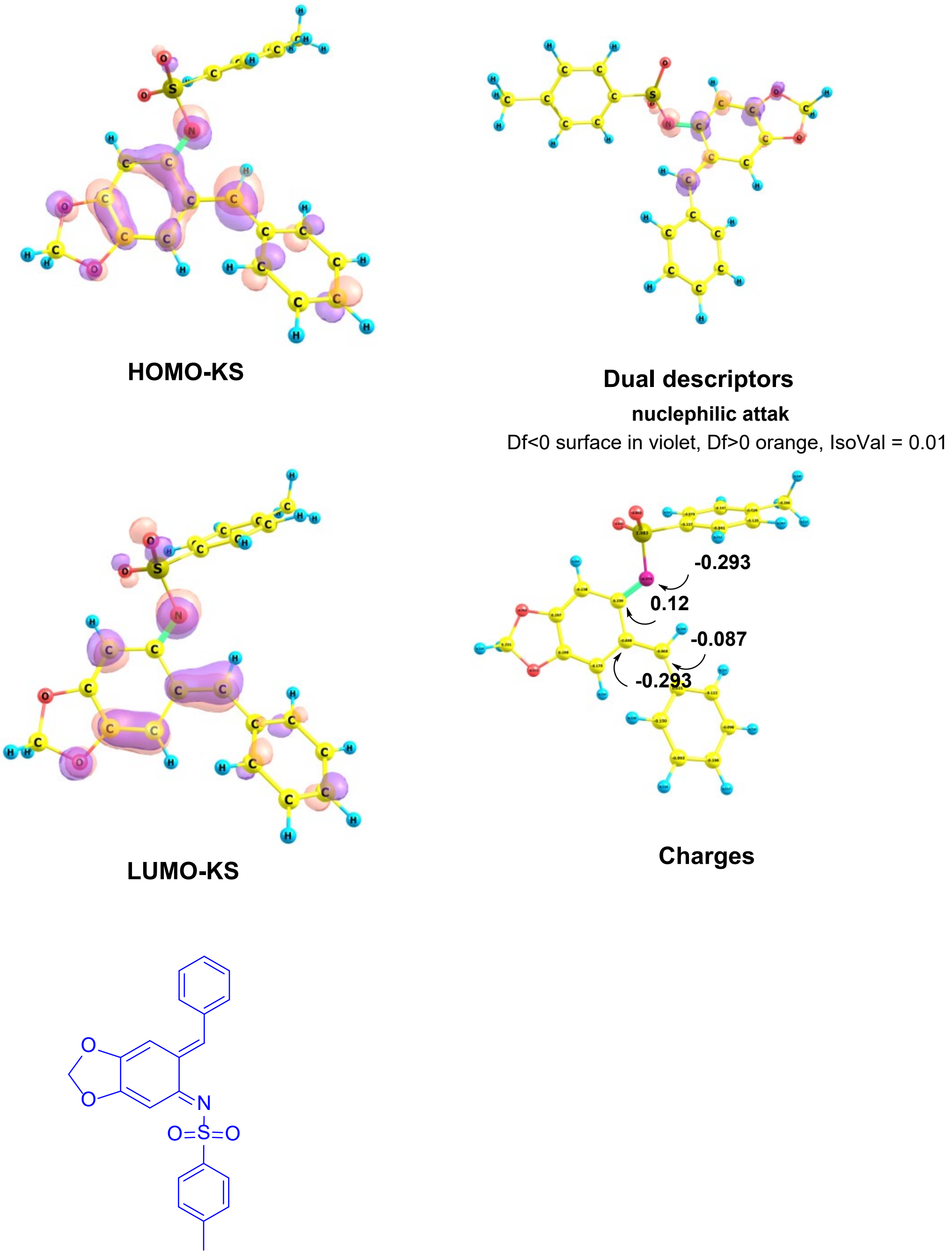

Figure S6. Frontier Kohn-Sham orbitals, dual descriptors, Mulliken charges (black), and Hirshfeld atomic *charge* (black) of the enamine.

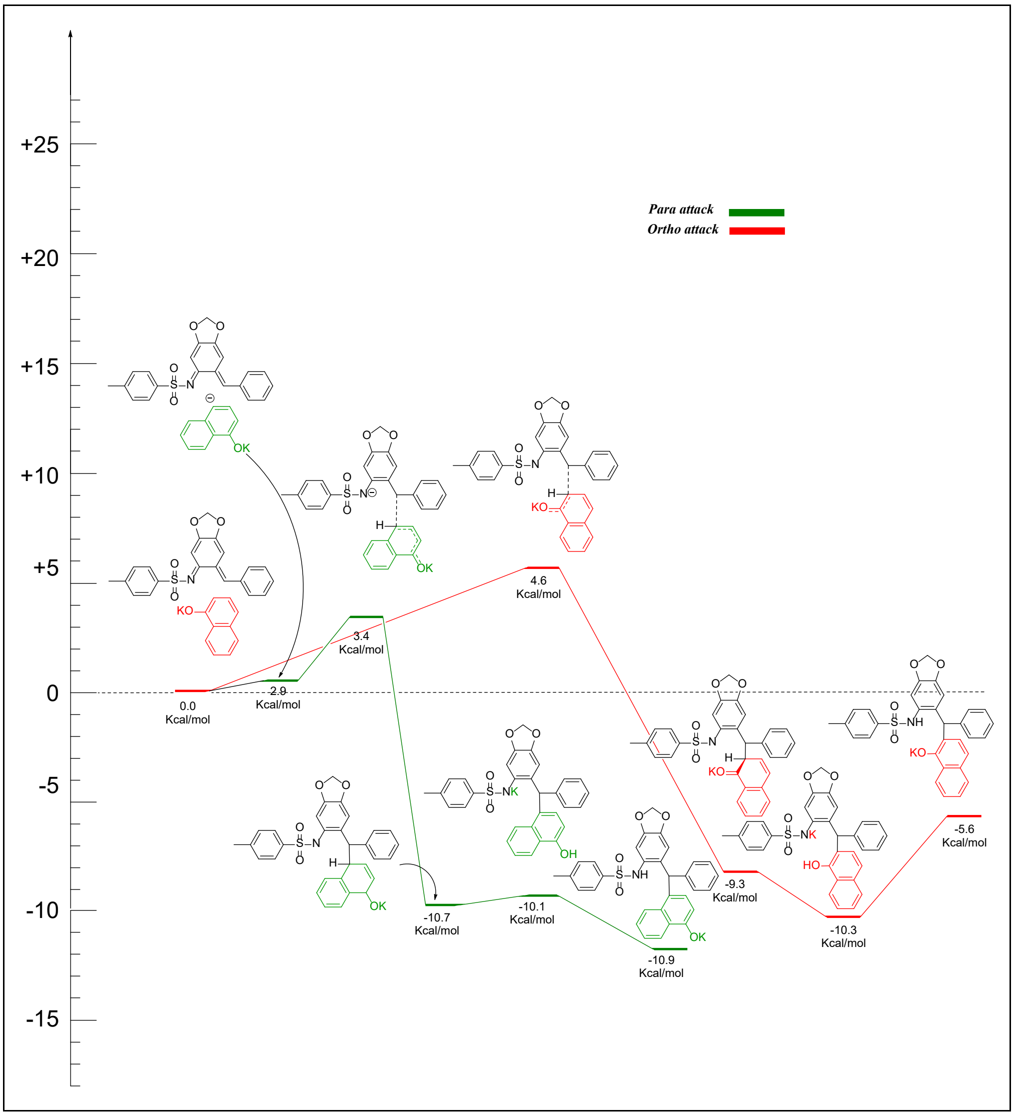

Figure S7. Fidel-Crafts reaction and *ortho*- and *para*- selectivity in a vacuum with potassium at M06L/6-31G(d).

<table>
<tr><td></td><td colspan="3">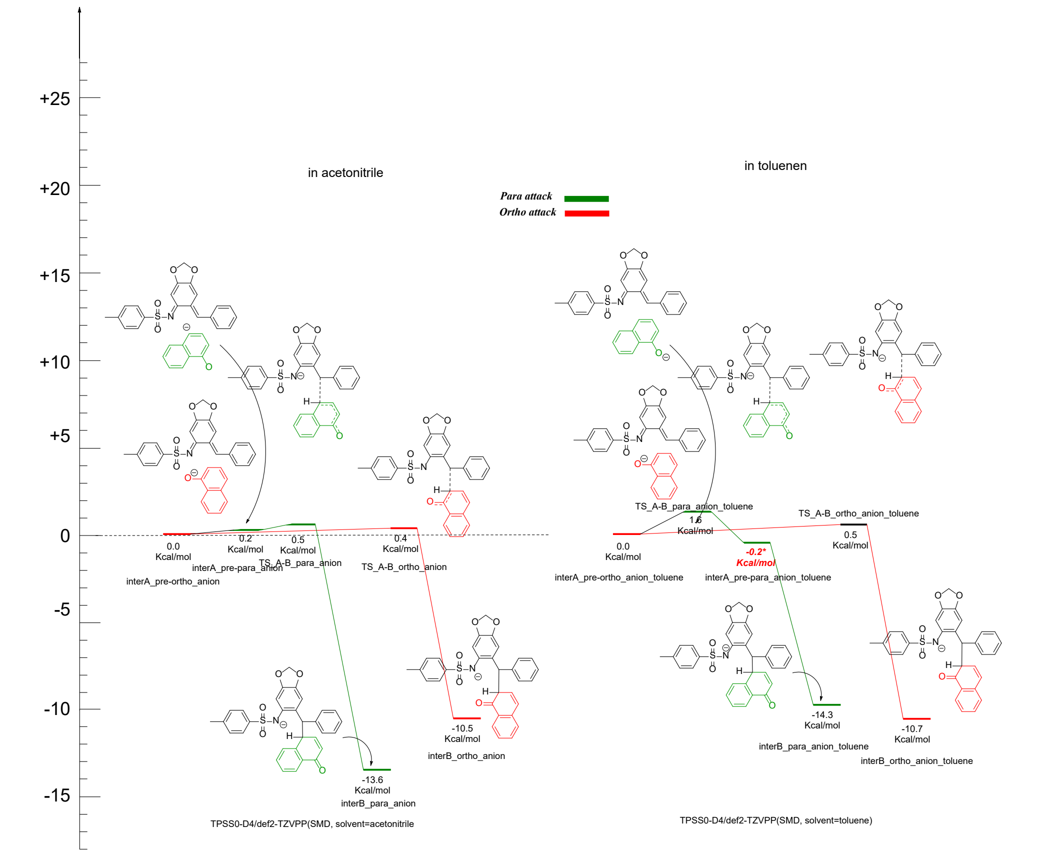
</td></tr>
<tr><td rowspan="4">Lowest conformational geometry in implicit solvent</td><td>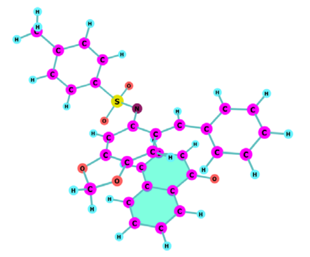</td><td>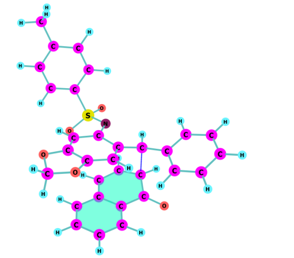</td><td>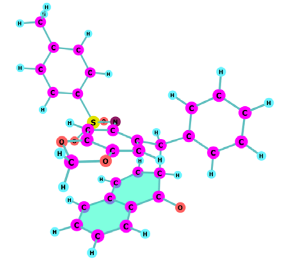</td></tr>
<tr><td>C-C distance = 2.92</td><td>C-C distance = 2.15</td><td>C-C distance = 1.59</td></tr>
<tr><td>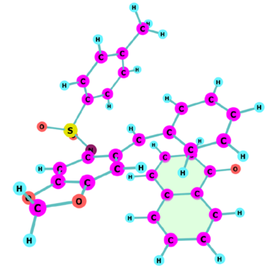</td><td>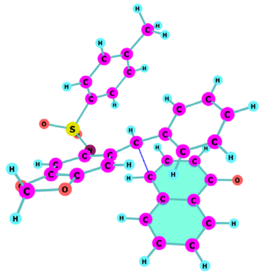</td><td>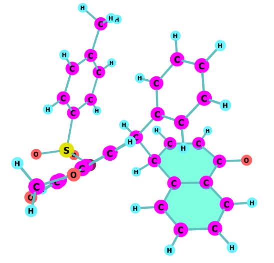</td></tr>
<tr><td>C-C distance = 2.59</td><td>C-C distance = 2.29</td><td>C-C distance = 1.59</td></tr>
</table>

Figure S8. Free energy profile for an anionic key step at M06L/6-31G(d) with acetonitrile and toluene in implicit solvent.

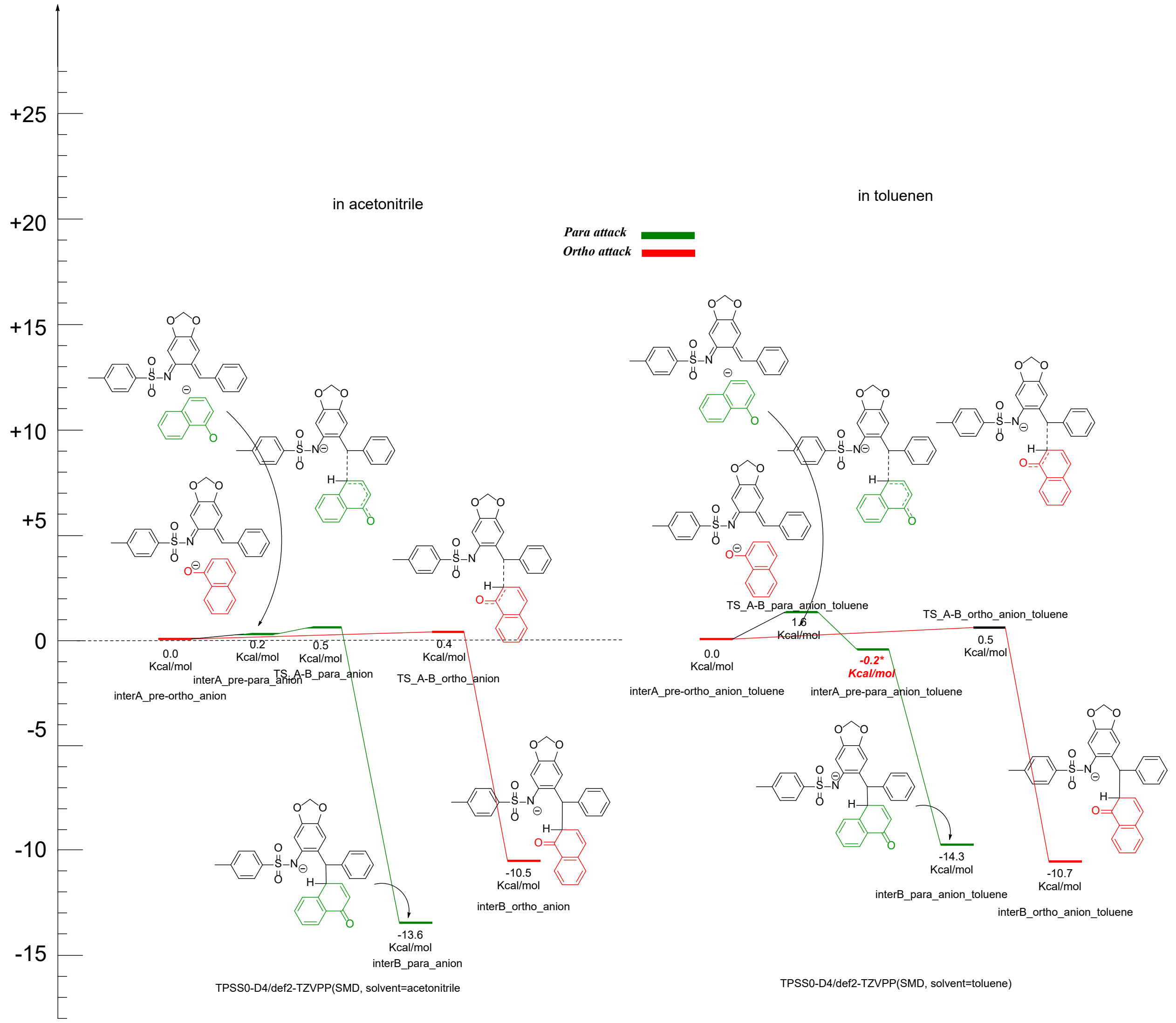

Figure S9. Free energy profile for an anionic key step at TPSS0-D4/def2TZVPP with acetonitrile and toluene as implicit solvent. NOTE: formation of the para is spontaneous under anionic condition in toluene.

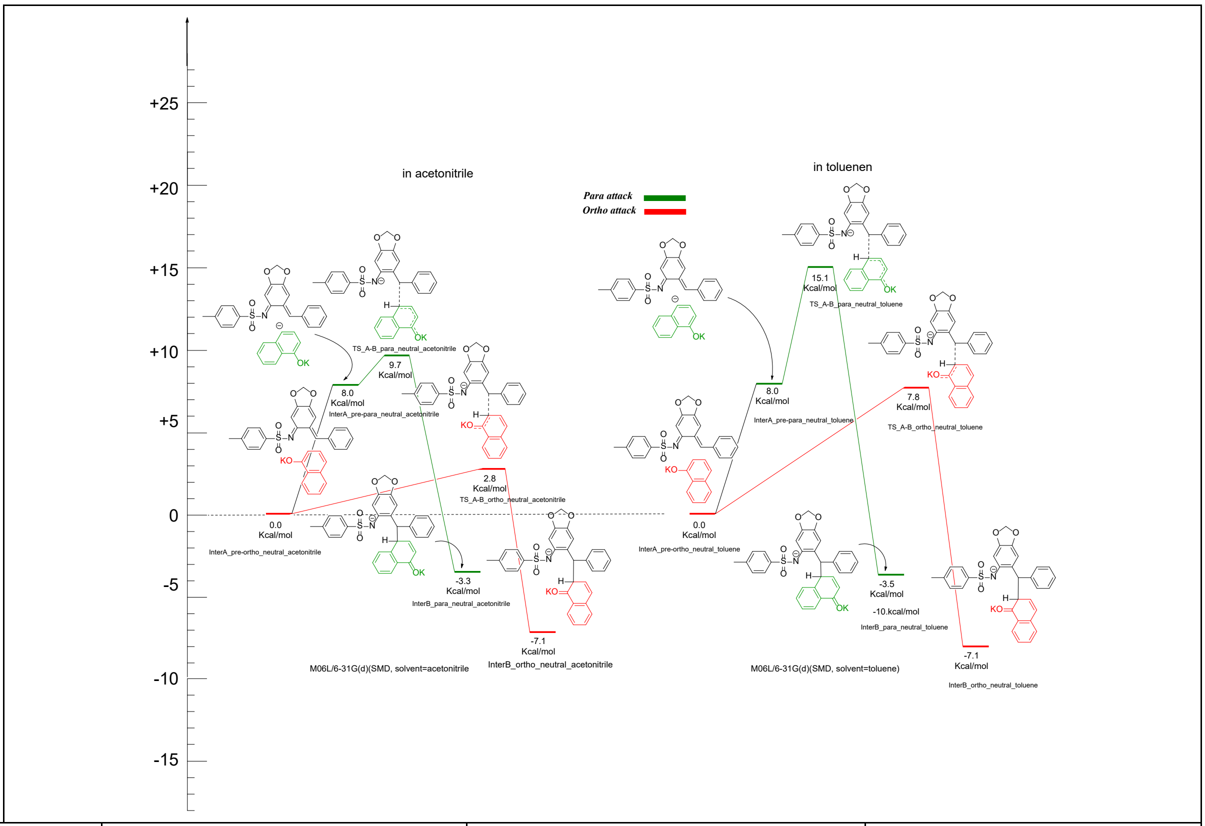

Lowest conformation geometry in acetonitrile implicit solvent	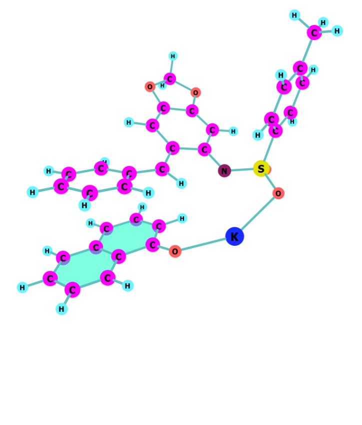	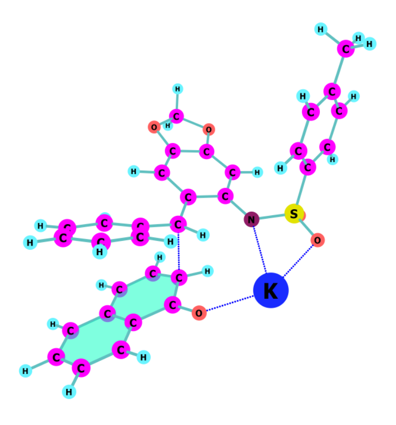	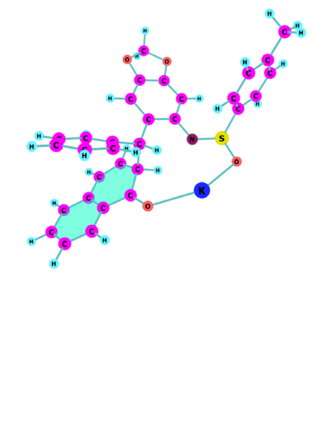
	C-C distance = 2.78	C-C distance = 2.17	C-C distance = 1.61
	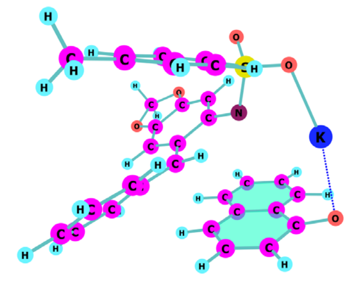	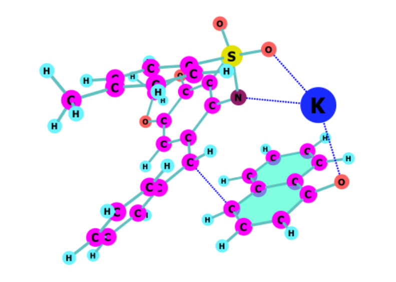	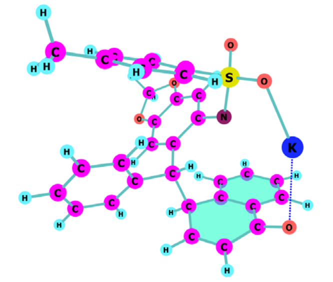
	C-C distance = 2.65	C-C distance = 2.31	C-C distance = 1.59

Lowest conformation geometry in toluene implicit solvent	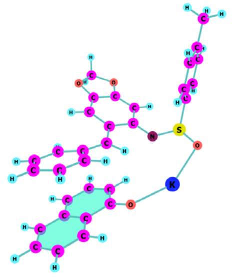	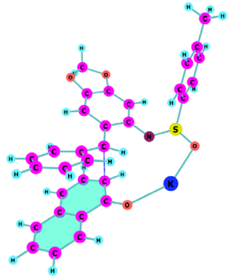	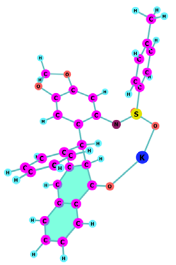
	C-C distance = 2.78	C-C distance = 2.17	C-C distance = 1.61
	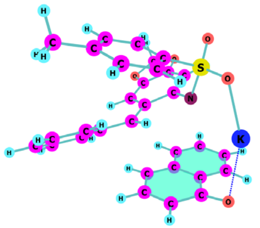	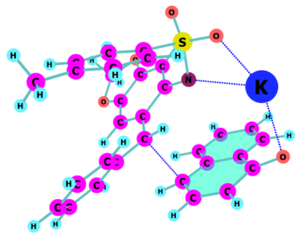	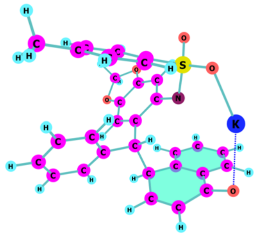
	C-C distance = 2.65	C-C distance = 2.31	C-C distance = 1.59

Figure S9. Free energy profile for neutral key step at M06L/6-31G(d) with acetonitrile and toluene with implicit solvent.

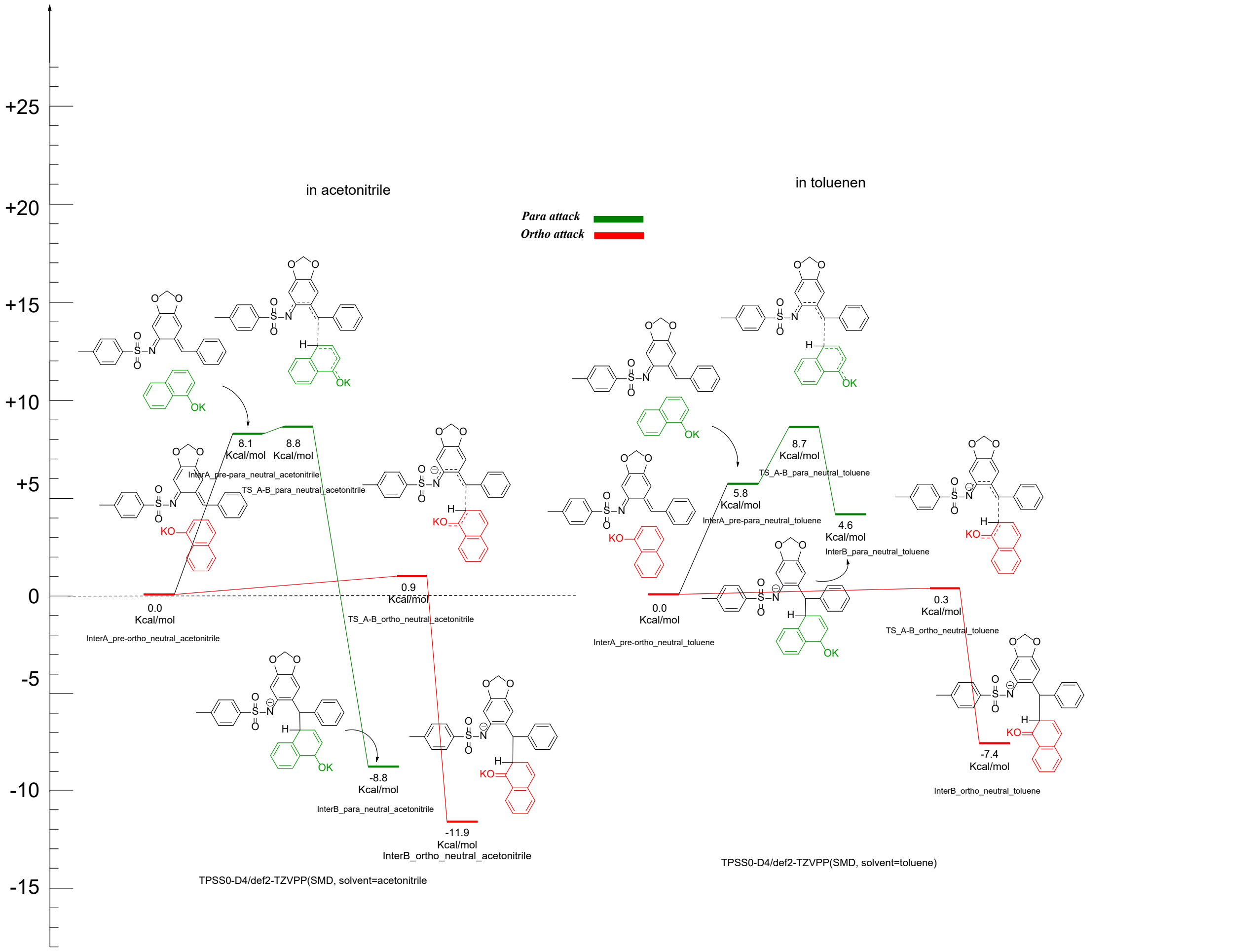

Figure S10. Free energy profile for neutral key step at TPSS0-D4/def2TZVPP with acetonitrile and toluene with implicit solvent.

	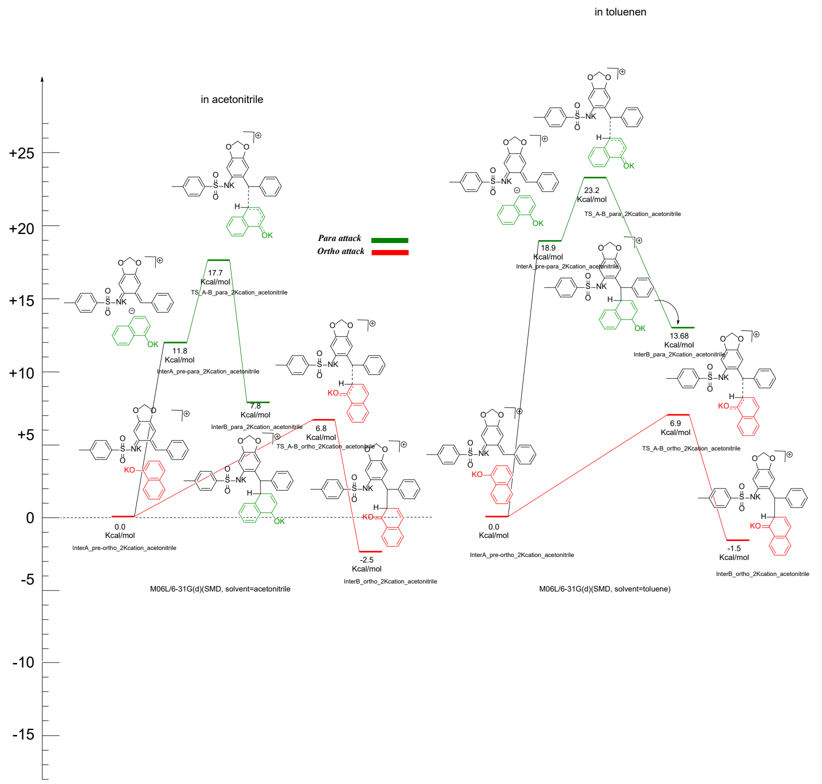		
Lowest conformation geometry in acetonitrile implicit solvent	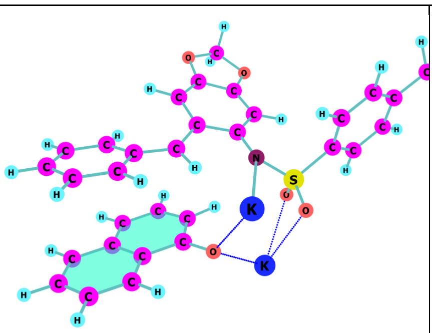	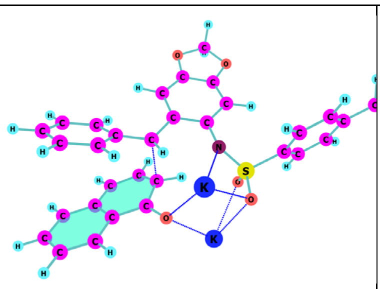	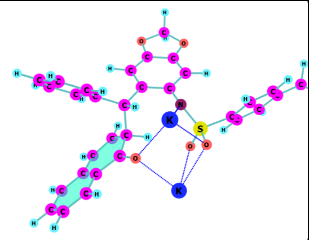
	C-C distance = 2.94	C-C distance = 2.10	C-C distance = 1.59
	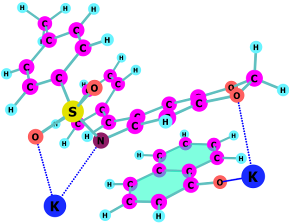	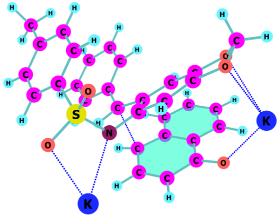	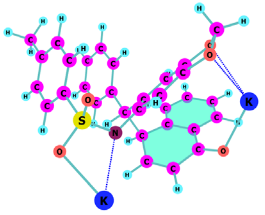
	C-C distance = 2.81	C-C distance = 2.26	C-C distance = 1.61

Lowest conformation geometry in toluene implicit solvent			
	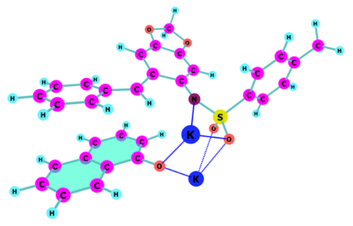	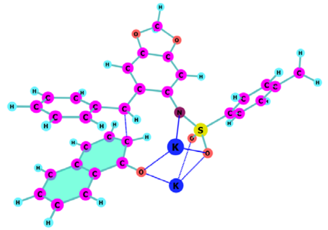	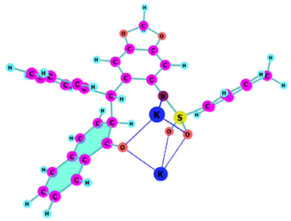
	C-C distance = 2.94	C-C distance = 2.10	C-C distance = 1.59
	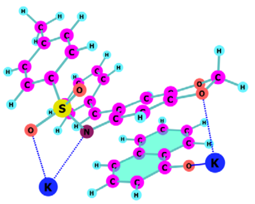	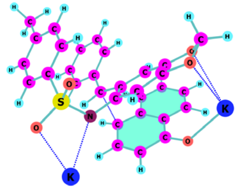	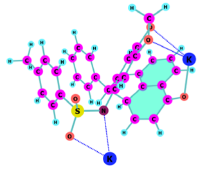
	C-C distance = 2.81	C-C distance = 2.26	C-C distance = 1.61

Figure S11. Free energy profile for cationic key step at M06L/6-31G(d) with acetonitrile and toluene with implicit solvent.

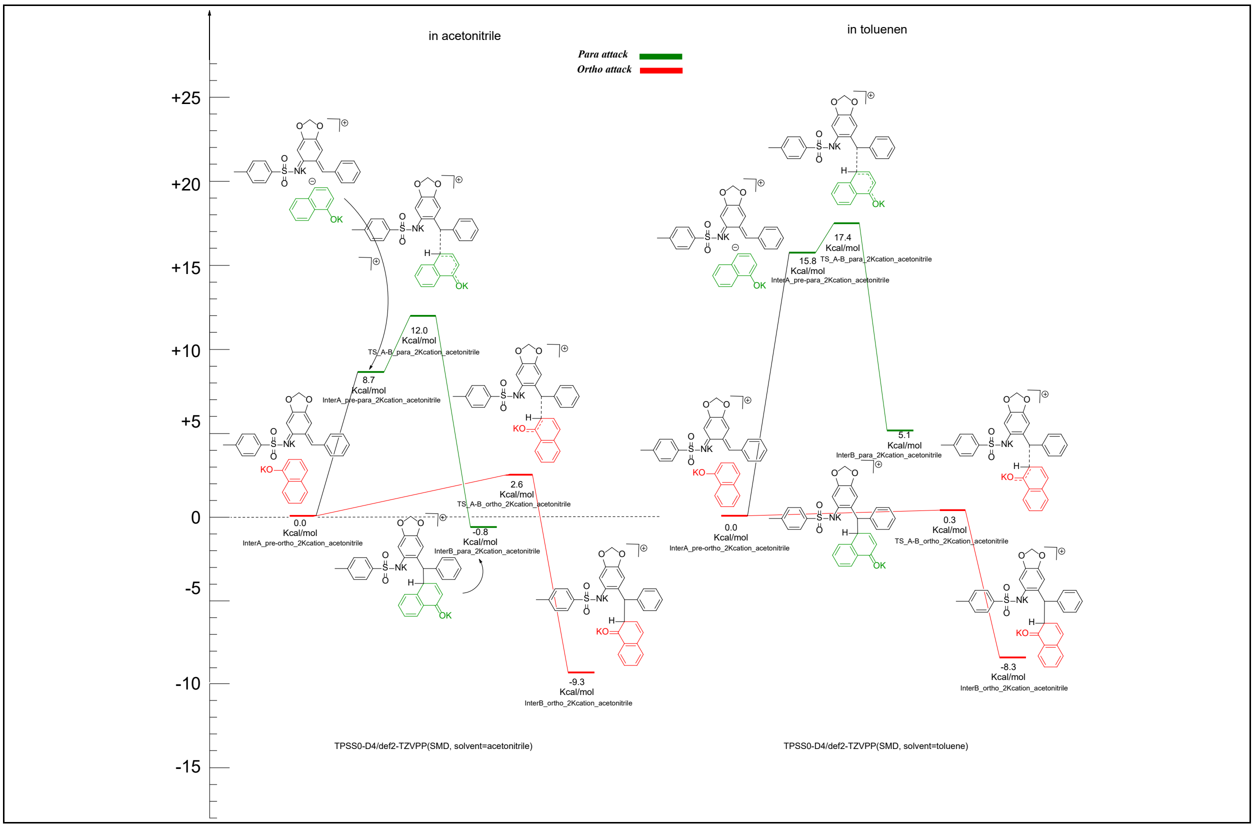

Figure S12. Free energy profile for cationic key step at TPSS0-D4/def2TZVPP with acetonitrile and toluene with implicit solvent.

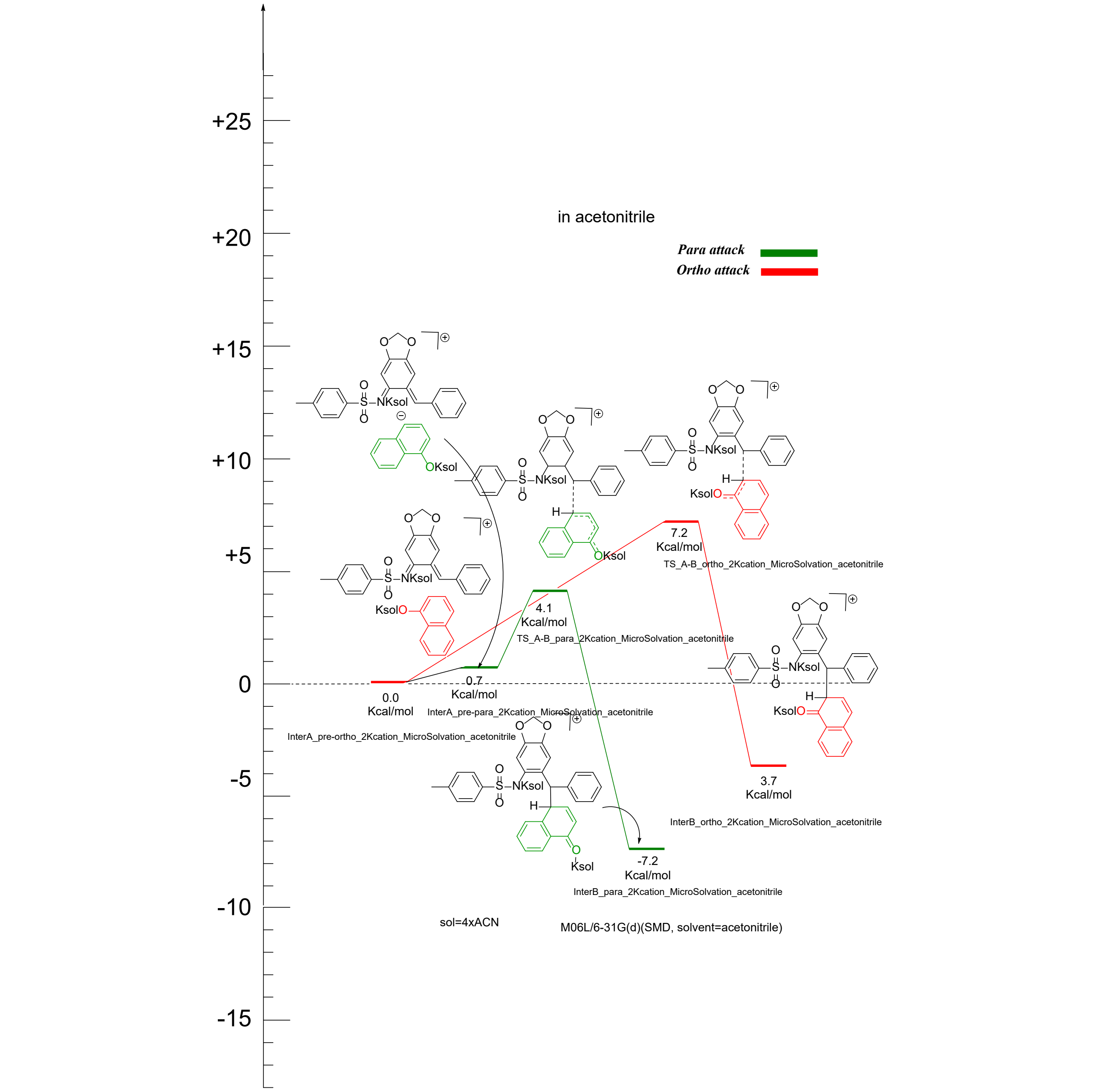

Lowest conformation geometry in acetonitrile implicit solvent	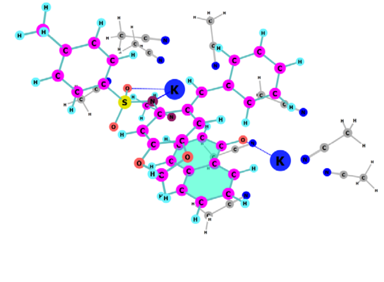	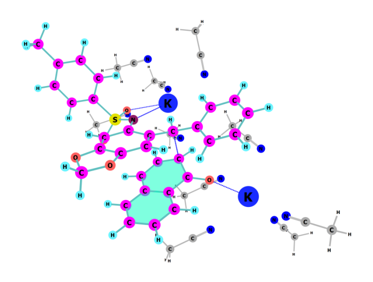	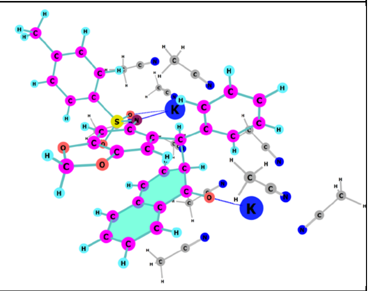
	C-C distance = 3.51	C-C distance = 2.12	C-C distance = 1.60

	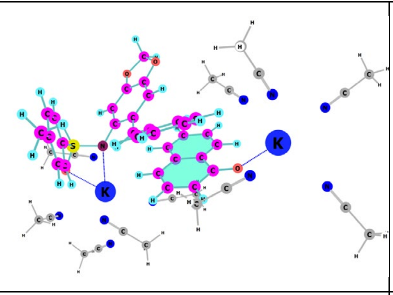	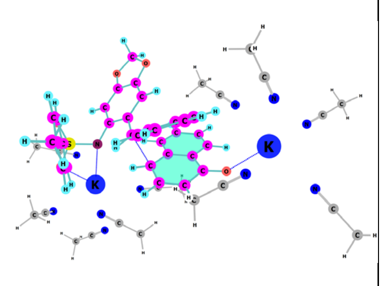	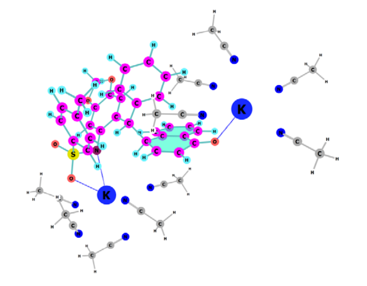
	C-C distance = 2.69	C-C distance = 2.25	C-C distance = 1.58

Figure S13. Free energy profile for cation key step at M06L/6-31G(d) with microsolvated with acetonitrile with acetonitrile as implicit solvent.

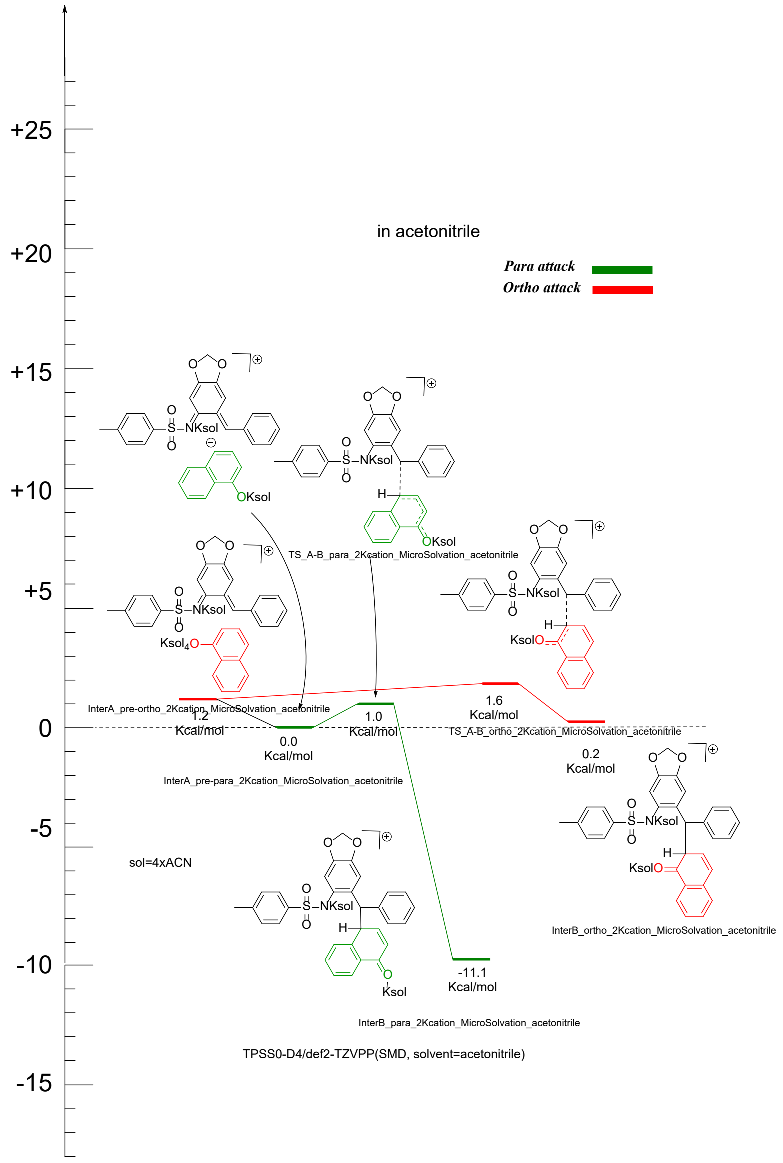

Figure S14. Free energy profile for neutral key step at TPSS0-D4/def2TZVPP with microsolvated with acetonitrile with acetonitrile as implicit solvent.

5. Cartesian Coordinates

Table S1. Computed energies for the anionic transition state conformational analysis in acetonitrile.

	M06-L/BS1							TPSS0-D4/BS2
	1st frequency (cm-1)	Electronic energy	ZPE corr	Thermal corr	Enthalpy corr:	Gibbs corr	Electronic energy in acetonitrile	Electronic energy
pre-ortho TS								
TS_ortho_anion_conf2	-352.5	-2024.57129	0.47706	0.50873	0.50968	0.41141	-2024.66146	-2025.40274
TS_ortho_anion_conf3	-350.3	-2024.56947	0.47775	0.50895	0.50989	0.41362	-2024.65973	-2025.40580
TS_ortho_anion_conf4	-279.3	-2024.55913	0.47667	0.50831	0.50925	0.41114	-2024.65699	-2025.39226
TS_ortho_anion_conf5	-318.1	-2024.56553	0.47681	0.50821	0.50916	0.41386	-2024.65985	-2025.39693
TS_ortho_anion_conf6	-356.3	-2024.56966	0.47763	0.50896	0.50991	0.41242	-2024.65942	-2025.40448
TS_ortho_anion_conf7	-254.3	-2024.56474	0.47691	0.50858	0.50952	0.41058	-2024.65892	-2025.39784
TS_ortho_anion_conf8	-320.4	-2024.56555	0.47712	0.50847	0.50942	0.41418	-2024.65982	-2025.39694
TS_ortho_anion_conf9	-356.1	-2024.56967	0.47758	0.50892	0.50986	0.41259	-2024.65939	-2025.40456
TS_ortho_anion_conf10	-251.5	-2024.56466	0.47694	0.50862	0.50956	0.41069	-2024.65894	-2025.39759
TS_ortho_anion_conf11	-279.8	-2024.55912	0.47673	0.50835	0.50929	0.41155	-2024.65692	-2025.39228
TS_ortho_anion_conf12	-353.1	-2024.56942	0.47772	0.50894	0.50989	0.41333	-2024.65977	-2025.40578
TS_ortho_anion_conf13	-257.3	-2024.56475	0.47692	0.50858	0.50953	0.41038	-2024.65895	-2025.39788
TS_ortho_anion_conf14	-280.1	-2024.55911	0.47695	0.50848	0.50942	0.41223	-2024.65702	-2025.39227
TS_ortho_anion_conf15	-351.0	-2024.56943	0.47756	0.50887	0.50982	0.41247	-2024.65980	-2025.40584
TS_ortho_anion_conf16	-257.3	-2024.56474	0.47680	0.50851	0.50945	0.40981	-2024.65886	-2025.39788
TS_ortho_anion_conf17	-280.8	-2024.55915	0.47674	0.50834	0.50929	0.41153	-2024.65693	-2025.39229
pre-para TS								
TS_para_anion_conf1	-299.7	-2024.56462	0.47701	0.50875	0.50969	0.41210	-2024.65676	-2025.39553
TS_para_anion_conf2	-358.7	-2024.57017	0.47833	0.50908	0.51003	0.41676	-2024.66113	-2025.40797
TS_para_anion_conf3	-193.2	-2024.57312	0.47722	0.50862	0.50956	0.41352	-2024.66120	-2025.40632
TS_para_anion_conf4	-286.2	-2024.56244	0.47748	0.50869	0.50964	0.41377	-2024.65566	-2025.39953
TS_para_anion_conf5	-199.8	-2024.57113	0.47715	0.50872	0.50967	0.41201	-2024.65993	-2025.40575

Table S2. Computed energies for the anionic path in acetonitrile.

	M06-L/BS1							TPSS0-D4/BS2
	1st frequency (cm-1)	Electronic energy	ZPE corr	Thermal corr	Enthalpy corr:	Gibbs corr	Electronic energy in acetonitrile	Electronic energy
Path for ortho product								
interA_pre-ortho_anion.log	15.7	-2024.57632	0.47704	0.50924	0.51018	0.41161	-2024.66918	-2025.40362
TS_A-B_ortho_anion.log	-351.1	-2024.56944	0.47744	0.50879	0.50973	0.41197	-2024.65981	-2025.40579
interB_ortho_anion.log	11.6	-2024.58623	0.47979	0.51127	0.51221	0.41374	-2024.67608	-2025.42548
Path for para product								
interA_pre-para_anion.log	12.2	-2024.57176	0.47699	0.50941	0.51035	0.40989	-2024.66089	-2025.40522
TS_A-B_para_anion.log	-207.4	-2024.57109	0.47696	0.50865	0.50959	0.41049	-2024.65987	-2025.40580
interB_para_anion.log	17.5	-2024.59178	0.48013	0.51155	0.51249	0.41503	-2024.68011	-2025.43325

interB_ortho_anion.log

C 12.3603986676 -15.088675179 -1.8222374977
C 12.7644369731 -16.5786215136 -1.446831103
C 14.2357139185 -17.5347582763 -3.2088754581
C 12.7381228734 -14.7726676053 -5.6126804982
C 11.4711843463 -15.1584468421 -5.9763550192
C 10.4694616176 -15.4187369724 -5.053665806
C 10.7083153473 -15.3220337136 -3.7039029367
C 12.0060415211 -14.9785245342 -3.2872295573
C 13.0346606705 -14.6717848031 -4.2243149767
C 9.722112669 -15.9720633291 -7.0495346674
H 13.5020711377 -14.5824191179 -6.3599073641
H 9.9365635043 -15.5540888685 -2.9698596554
H 8.9776710987 -15.5710566925 -7.7481597658
O 10.9601680474 -15.309983181 -7.2536057885
O 9.2948471237 -15.7391447872 -5.7214189154
H 9.8593494273 -17.0598702915 -7.2107435632
N 14.2485620324 -14.2748995704 -3.7032083318
S 15.611651756 -14.5025142474 -4.4873956843
O 15.6246488376 -15.6498294011 -5.4238272627
O 16.7060716854 -14.40898228 -3.5091485079
C 9.8220169874 -14.3309195081 0.9755494915
C 9.5760347677 -12.9834246788 0.7405099775

C 10.7002882666 -15.0484645217 0.1614143064
H 8.8895357336 -12.4271479098 1.3784050427
H 10.8468465284 -16.1109329089 0.3374147226
C 10.2142331993 -12.3534207068 -0.3286807802
C 11.3481283566 -14.4284482142 -0.9100415368
H 10.0329906803 -11.2976314654 -0.5297186226
C 11.0826493814 -13.0691279445 -1.1413469319
H 11.5782317296 -12.5798286377 -1.980713754
H 9.3218590597 -14.8412643283 1.7987715461
C 15.8270429078 -13.167456584 -6.9259291764
C 15.9555380186 -12.0273219255 -7.7180798876
C 15.8238440668 -13.0531825042 -5.5397328546
H 15.9593637768 -12.1213709542 -8.8056937737
C 16.0825094758 -10.7611559238 -7.1456831351
C 15.9536766027 -11.7935730031 -4.9501417918
H 15.9547927369 -11.7170823089 -3.8639939233
C 16.0824422749 -10.6650046323 -5.7464479412
H 16.1864722078 -9.6829760877 -5.2807640897
H 15.7388000156 -14.1589962786 -7.3672132827
C 16.2055984232 -9.5321731776 -7.9966018278
H 16.3085992678 -9.7859084926 -9.0582426738
H 15.3261447273 -8.8798692687 -7.901536741
H 17.0758512915 -8.9240198649 -7.7149762165
H 13.3006342617 -14.5281115395 -1.7075409827
H 15.1984938635 -17.5777898963 -3.7140731696
C 14.0981220193 -16.8350954575 -2.0720310486
C 11.698860616 -17.5829658908 -1.862175429
C 11.8571553014 -18.2226553958 -3.1835767079
C 13.0976867753 -18.1511109819 -3.8582767468
C 13.1903938027 -18.7009607604 -5.1457992204
C 12.0911208113 -19.3108654701 -5.7353345621
C 10.8761677079 -19.4086637115 -5.0475928182
C 10.7676425306 -18.8656768934 -3.7751129756
H 14.9536191175 -16.3033101408 -1.6570130809
H 12.8388344536 -16.6123921011 -0.3496552846
O 10.7270887054 -17.8252207003 -1.1473854567
H 9.8319653824 -18.8949244556 -3.2184569405
H 10.0175080669 -19.8873883408 -5.516921291

H 12.1770899963 -19.7155719837 -6.7439130122
H 14.1330698494 -18.6102052442 -5.6847570596

TS_A-B_ortho_anion.log
C 12.389104 -14.757886 -2.084501
C 13.246031 -16.603913 -1.376059
C 13.993413 -17.507389 -3.49864
C 13.283589 -15.013388 -5.732177
C 12.061867 -15.378304 -6.226014
C 10.91141 -15.43816 -5.438053
C 10.951576 -15.189856 -4.098169
C 12.201092 -14.870049 -3.504897
C 13.381083 -14.707961 -4.34008
C 10.413068 -16.210677 -7.437366
H 14.160245 -14.982474 -6.369264
H 10.048589 -15.25397 -3.50205
H 9.828422 -15.8587 -8.294984
O 11.733872 -15.689056 -7.52546
O 9.825462 -15.745097 -6.241871
H 10.466045 -17.316484 -7.421418
N 14.497957 -14.247293 -3.716172
S 15.94627 -14.257136 -4.407806
O 16.236424 -15.442738 -5.240058
O 16.912972 -13.894758 -3.366047
C 9.159041 -14.6785 -0.051219
C 9.465597 -13.678905 0.867231
C 10.085483 -15.069295 -1.011974
H 8.736041 -13.378921 1.618807
H 9.838294 -15.894227 -1.673406
C 10.724527 -13.081474 0.828226
C 11.34813 -14.45399 -1.094257
H 10.987411 -12.307444 1.548843
C 11.648794 -13.472765 -0.130844
H 12.632246 -13.002731 -0.160914
H 8.192376 -15.178955 -0.010448
C 16.081933 -13.054355 -6.916067
C 16.050932 -11.957988 -7.776352
C 15.933955 -12.86294 -5.547096

H 16.168629 -12.109916 -8.850615
C 15.871266 -10.663014 -7.288583
C 15.757661 -11.574096 -5.039849
H 15.647352 -11.438716 -3.965346
C 15.727394 -10.489803 -5.904554
H 15.589849 -9.483399 -5.505221
H 16.231805 -14.065484 -7.290904
C 15.82502 -9.484182 -8.214479
H 16.028578 -9.777492 -9.250868
H 14.841965 -8.993797 -8.200102
H 16.560107 -8.716925 -7.936599
H 13.332628 -14.262476 -1.8569
H 14.747445 -17.587412 -4.280839
C 14.277332 -16.858703 -2.330935
C 12.041031 -17.430067 -1.389107
C 11.714528 -18.027198 -2.697197
C 12.681893 -18.032807 -3.740208
C 12.306514 -18.568549 -4.988859
C 11.030578 -19.069633 -5.196171
C 10.085945 -19.067375 -4.160789
C 10.44072 -18.557843 -2.920075
H 15.262033 -16.420047 -2.179654
H 13.516929 -16.212698 -0.394746
O 11.298702 -17.5558 -0.400482
H 9.739578 -18.539598 -2.085129
H 9.083209 -19.456494 -4.333558
H 10.762867 -19.474796 -6.173376
H 13.036188 -18.566614 -5.800288

interA_pre-ortho_anion.log

C 12.2865408206 -14.4135315688 -2.1088648558
C 13.5013575947 -16.9302213697 -1.2793612929
C 13.9559175436 -17.4751971605 -3.5994724929
C 13.4210360687 -15.0226986257 -5.627780921
C 12.2167414713 -15.3600260962 -6.1674143435
C 11.0055220806 -15.2989562321 -5.4527366367
C 10.9638843771 -14.9532647423 -4.1434696891
C 12.1986067793 -14.6571661455 -3.4772890026

C 13.4518498735 -14.6031579616 -4.2580026606
C 10.5878188961 -16.1292239583 -7.4581689868
H 14.3339542699 -15.0819269538 -6.2090012975
H 10.0215705432 -14.9001653702 -3.6103456781
H 10.0999027512 -15.7342856072 -8.3568913238
O 11.9547811274 -15.7342744904 -7.458496497
O 9.9663865161 -15.5920948029 -6.3098354411
H 10.5350644505 -17.2319024842 -7.4290918182
N 14.5260980635 -14.1090703501 -3.6179631468
S 16.0074510513 -14.1157260023 -4.272131366
O 16.3596517654 -15.3578491597 -4.9852614107
O 16.9195083107 -13.6248849798 -3.2376250436
C 9.1290010718 -14.9718898204 -0.0842959882
C 9.3556060235 -14.0992691703 0.9755229107
C 10.0673542146 -15.1165578168 -1.0983287009
H 8.6176247251 -13.9945532047 1.7701567857
H 9.9018258746 -15.8610969439 -1.8717900069
C 10.5558441378 -13.3871353862 1.0302824213
C 11.2638678409 -14.368796604 -1.0925737385
H 10.7592824318 -12.7203192843 1.8678315716
C 11.4951274321 -13.5327391901 0.0226826549
H 12.4337006648 -12.9796391671 0.0673294393
H 8.2229707589 -15.5761302643 -0.1095203841
C 16.1843114896 -13.1286703554 -6.8640352662
C 16.134079117 -12.1149510539 -7.8189752693
C 15.9565021662 -12.8273290334 -5.5264771596
H 16.3142328074 -12.3517493006 -8.8687210284
C 15.8545776822 -10.7963909172 -7.457021834
C 15.6821809001 -11.5135833448 -5.1426155391
H 15.5114561953 -11.2916601548 -4.0906481772
C 15.6313432871 -10.5128398258 -6.1022321437
H 15.4153841487 -9.4864101109 -5.8013055742
H 16.4086735811 -14.1577507406 -7.1396140423
C 15.7863622844 -9.7073047443 -8.4856029695
H 16.061220249 -10.0747628329 -9.480918852
H 14.7761712752 -9.2822750495 -8.5604980205
H 16.458341716 -8.8736479346 -8.2421309321
H 13.2690008436 -14.0724994198 -1.7851640805

H 14.6416623958 -17.4783084267 -4.4455434631
C 14.3702790157 -16.9957750165 -2.3665613589
C 12.1656455453 -17.4631811015 -1.3313695819
C 11.7298045086 -17.9368951155 -2.6581549364
C 12.6239881738 -17.9347953217 -3.7713945333
C 12.1336557506 -18.3829183579 -5.021322609
C 10.8233354954 -18.8060239117 -5.166256426
C 9.944859785 -18.7872239528 -4.0720775569
C 10.4081070375 -18.36119394 -2.8360845276
H 15.3844676057 -16.6081233044 -2.2670262247
H 13.8334417127 -16.5362356535 -0.3189386699
O 11.4003095355 -17.5030909146 -0.3388991707
H 9.7648518745 -18.3431257591 -1.9551801929
H 8.9118965892 -19.112134597 -4.1948525859
H 10.4725176363 -19.1603743441 -6.1375019758
H 12.8124830648 -18.387737551 -5.8769573586

interB_para_anion.log

C 11.4185041605 -15.7132707413 -2.0395393392
C 12.6390294574 -16.6840093264 -1.7053911966
C 9.6141532119 -15.2805300528 1.2963020809
C 9.7029850008 -13.9160293702 1.4375558526
C 10.3467963755 -13.1146519869 0.5090704247
C 10.9318600599 -13.6432775206 -0.6193604578
C 10.8549539715 -15.033994359 -0.8121796514
C 10.1977622867 -15.866369475 0.1426409393
C 9.3954778484 -11.8005381242 2.008305404
H 9.09903022 -15.9110174035 2.0130783802
H 11.4129946356 -12.996324849 -1.3476911664
H 8.4149898352 -11.4252651791 1.653953822
O 9.2478260174 -13.1287060411 2.480784843
O 10.3219363095 -11.7952625101 0.9373794284
H 9.7727117054 -11.1655296356 2.8195261188
N 10.2154740336 -17.239549993 -0.0646502409
S 8.8304856697 -18.018425392 -0.154648728
O 7.7818246264 -17.5628775672 0.781389984
O 9.1050747983 -19.4578021015 -0.240567082
C 13.0124352509 -13.120373808 -4.375208841

C 12.2655635242 -13.3069061095 -5.5354781378
C 12.7430194034 -13.872068426 -3.2343046642
H 12.4809513056 -12.7204576793 -6.4280179449
H 13.3419247503 -13.7128699031 -2.3387074458
C 11.2458028643 -14.2548637141 -5.5441699846
C 11.7189254375 -14.8299533347 -3.2262013742
H 10.6570316575 -14.4188795277 -6.4468753003
C 10.981364734 -15.0064563304 -4.4028184011
H 10.190135653 -15.7578777287 -4.41513762
H 13.817549528 -12.3868020646 -4.3558908099
C 7.6841273064 -16.2531572723 -1.9549235732
C 7.3997460536 -15.7902452073 -3.2336095213
C 8.2367178565 -17.525546356 -1.7813292152
H 6.9869840693 -14.7884369953 -3.365259675
C 7.6449325654 -16.5813771106 -4.3628259414
C 8.4842301184 -18.3274659295 -2.8938112018
H 8.9182318724 -19.3138997533 -2.7376766355
C 8.1837966204 -17.8576248755 -4.1698090134
H 8.379111672 -18.4879274879 -5.0397135492
H 7.4911024185 -15.6337968867 -1.0800169777
C 7.3556430821 -16.0593157408 -5.7389240267
H 6.2769588553 -15.9601239067 -5.9260212914
H 7.792781281 -15.0629912166 -5.8906540673
H 7.7611115113 -16.7205929183 -6.5141834323
H 10.6411935821 -16.4251962619 -2.3546242002
H 12.1981192419 -17.312117415 -0.9095562673
C 12.8670996047 -17.5340892791 -2.8968138287
C 14.0117169947 -17.5696651174 -3.6020883928
C 15.1760451804 -16.7723520398 -3.2261798855
C 15.0625669468 -16.004183148 -1.9607657165
C 13.872657606 -16.0023411825 -1.2058100954
C 13.8669634643 -15.3323660191 0.0271664201
C 14.9881709844 -14.64026163 0.4658704992
C 16.1523986 -14.613145007 -0.307658733
C 16.1879412603 -15.305638369 -1.506507012
H 12.0019735401 -18.1179633927 -3.2239997173
H 14.1171176431 -18.1839797513 -4.495202949
O 16.207557723 -16.7595921804 -3.9040166233

H 17.0806737185 -15.3370700075 -2.128999431
H 17.0286920356 -14.0666729718 0.0397642747
H 14.9565541118 -14.1205637973 1.4229021115
H 12.9678381474 -15.355826519 0.6400187757

TS_A-B_para_anion.log
C 11.116725 -15.520712 -1.975655
C 12.824938 -16.959113 -1.466451
C 9.45598 -15.289143 1.378442
C 9.717114 -13.97368 1.664715
C 10.484029 -13.158119 0.834173
C 11.010787 -13.613893 -0.342201
C 10.752358 -14.963812 -0.709194
C 10.003092 -15.825772 0.182538
C 9.712872 -11.91631 2.504638
H 8.862757 -15.921306 2.029343
H 11.550793 -12.94199 -0.999718
H 8.810187 -11.324895 2.263621
O 9.333264 -13.258205 2.774673
O 10.603412 -11.903567 1.405919
H 10.213686 -11.497566 3.387212
N 9.924348 -17.152525 -0.140384
S 8.511463 -17.904312 -0.196418
O 7.454825 -17.346959 0.670183
O 8.744652 -19.351315 -0.175841
C 13.189129 -13.278239 -4.230582
C 12.745773 -13.700042 -5.48103
C 12.668739 -13.838095 -3.069392
H 13.165714 -13.266127 -6.387215
H 13.081447 -13.53962 -2.10834
C 11.787349 -14.709579 -5.562338
C 11.678955 -14.839454 -3.126531
H 11.452618 -15.070195 -6.534627
C 11.277012 -15.279115 -4.404361
H 10.544287 -16.085795 -4.465274
H 13.974787 -12.527717 -4.153729
C 7.434057 -16.256396 -2.142295
C 7.253998 -15.843223 -3.457377

C 8.032397 -17.489209 -1.878485
H 6.794912 -14.874325 -3.660519
C 7.661711 -16.643388 -4.530866
C 8.4314 -18.307423 -2.934169
H 8.895238 -19.265915 -2.707424
C 8.239076 -17.885948 -4.24608
H 8.551777 -18.527797 -5.071783
H 7.122347 -15.629669 -1.307868
C 7.525248 -16.160606 -5.943882
H 6.577977 -15.631531 -6.10766
H 8.32834 -15.455745 -6.204671
H 7.57696 -16.987277 -6.662352
H 10.519331 -16.395384 -2.227655
H 12.192607 -17.310442 -0.652731
C 12.854569 -17.70221 -2.67282
C 13.768514 -17.454323 -3.661673
C 14.817175 -16.466641 -3.508911
C 14.867394 -15.782991 -2.194473
C 13.897436 -16.049957 -1.192298
C 13.974457 -15.342533 0.026936
C 14.97166 -14.406961 0.241591
C 15.933333 -14.155876 -0.749999
C 15.876332 -14.843127 -1.951007
H 12.068276 -18.445208 -2.827295
H 13.74065 -17.991514 -4.608862
O 15.63324 -16.20268 -4.41022
H 16.59456 -14.668551 -2.751522
H 16.718161 -13.420608 -0.572327
H 15.009446 -13.864917 1.18648
H 13.223659 -15.536918 0.793932

interA_pre-para_anion.log
C 11.0059973912 -15.398279677 -2.0009950231
C 12.8977207976 -17.0827073404 -1.4716874407
C 9.4366232456 -15.2735203776 1.3886130974
C 9.7409204529 -13.9797620633 1.7206058281
C 10.5243702587 -13.1515865358 0.9120879093
C 11.020857142 -13.5689551926 -0.287910908

C 10.7168846748 -14.8984026219 -0.7107019325
C 9.9610416254 -15.7812080388 0.1688925715
C 9.8368701238 -11.9652433505 2.6639045714
H 8.8366848137 -15.9132829062 2.0255792704
H 11.565887549 -12.8832887191 -0.9260971118
H 8.9630268128 -11.3082257921 2.5057888044
O 9.3874848678 -13.2991905591 2.858749583
O 10.6803522116 -11.9250887874 1.5297247409
H 10.3972405496 -11.6396561338 3.5507387786
N 9.8607474778 -17.0870895652 -0.1974952205
S 8.4502399348 -17.8558758686 -0.2073258184
O 7.4033428499 -17.2862887293 0.6620694129
O 8.6965125181 -19.299325034 -0.1591658748
C 13.2937460199 -13.3064938837 -4.1744288551
C 12.9433762345 -13.784741943 -5.4350441792
C 12.6770555988 -13.7994063576 -3.0329147561
H 13.446910997 -13.408012202 -6.3236520291
H 13.036204121 -13.475105772 -2.0589900258
C 11.9764826428 -14.7839300179 -5.5438902542
C 11.6709241063 -14.7879664315 -3.1173700085
H 11.7193962005 -15.1932091671 -6.5203904034
C 11.3687358354 -15.2889618205 -4.4048547497
H 10.6326292843 -16.0911441048 -4.4833812704
H 14.0932264694 -12.5739070188 -4.0725282363
C 7.3123145029 -16.2689436298 -2.1680189139
C 7.1345531783 -15.8674623464 -3.487877339
C 7.9634803711 -17.4706108268 -1.892886292
H 6.6333062829 -14.9220926748 -3.7014892815
C 7.5999232669 -16.6473970694 -4.5514542234
C 8.4156725134 -18.2737647034 -2.9394621538
H 8.9200082534 -19.2091109809 -2.7032321766
C 8.2274274789 -17.8635265266 -4.2543228537
H 8.5846541528 -18.4913025361 -5.0726132484
H 6.9609961966 -15.6554389403 -1.3396725872
C 7.4748905434 -16.1731899772 -5.9682700449
H 6.5956311356 -15.532988114 -6.1089868593
H 8.3510612513 -15.5797810417 -6.26871669
H 7.3999383445 -17.0094541866 -6.6739035176

H 10.4527783185 -16.3039998726 -2.2432314521
H 12.2248976884 -17.3873454956 -0.6720371474
C 12.8623142094 -17.7475419998 -2.7063879348
C 13.7166130105 -17.4198443549 -3.7368988992
C 14.7324780616 -16.4031910636 -3.5980314665
C 14.8267757863 -15.775246416 -2.2608960888
C 13.9085280932 -16.1132718217 -1.2262981312
C 13.9879787652 -15.4121114349 0.0000102433
C 14.9470447973 -14.4367218543 0.1995862666
C 15.8730292741 -14.1344712539 -0.81443454
C 15.8046697941 -14.7998498156 -2.0263286734
H 12.0899415626 -18.5050718714 -2.8595494965
H 13.6465840486 -17.9095137202 -4.7076994413
O 15.4822360248 -16.0578816541 -4.5350959162
H 16.4832047809 -14.5740737787 -2.8487163807
H 16.6332866414 -13.3715125274 -0.6459737117
H 14.9854576406 -13.9021859346 1.1489628281
H 13.2661622233 -15.6477075652 0.783884653

TS_ortho_anion_conf2
C 12.276099 -14.499929 -1.074385
C 13.273833 -16.21937 -1.922243
C 13.002339 -16.231502 -4.348787
C 15.328415 -12.584258 0.055661
C 15.182026 -11.47946 -0.733724
C 14.113143 -11.307176 -1.617356
C 13.144075 -12.256356 -1.760878
C 13.235315 -13.443182 -0.981658
C 14.336735 -13.612138 -0.04268
C 15.307705 -9.436457 -1.60152
H 16.149157 -12.676761 0.758896
H 12.297776 -12.08793 -2.418327
H 14.909383 -8.637195 -0.949309
O 16.006042 -10.381872 -0.804473
O 14.245703 -10.089983 -2.266359
H 15.996664 -9.009842 -2.342283
N 14.335083 -14.750191 0.6782
S 15.585299 -15.153034 1.624216

O 15.947289 -14.144901 2.642063
O 16.704016 -15.703649 0.836531
C 9.759012 -14.085447 -3.892184
C 8.619127 -14.620342 -3.299634
C 10.953372 -14.006385 -3.183016
H 7.688952 -14.690864 -3.862265
H 11.848717 -13.647035 -3.68302
C 8.685392 -15.087044 -1.98707
C 11.036881 -14.462934 -1.855128
H 7.805997 -15.527743 -1.5186
C 9.877132 -15.015357 -1.279694
H 9.942819 -15.420987 -0.270878
H 9.727791 -13.746735 -4.927527
C 15.28936 -16.762515 3.806575
C 14.787466 -17.851814 4.512893
C 14.833149 -16.508915 2.515995
H 15.14111 -18.048031 5.526705
C 13.82671 -18.695795 3.950019
C 13.874584 -17.337174 1.932371
H 13.481809 -17.132131 0.937581
C 13.377891 -18.415536 2.653949
H 12.616613 -19.045917 2.191975
H 16.020233 -16.090109 4.251721
C 13.282002 -19.866472 4.71351
H 13.718922 -19.933711 5.717024
H 12.191706 -19.804543 4.830616
H 13.485383 -20.816386 4.200755
H 12.202428 -15.108143 -0.169895
H 13.313494 -15.903294 -5.341487
C 13.689225 -15.84561 -3.233308
C 12.190631 -17.17417 -1.745259
C 11.454283 -17.562224 -2.962513
C 11.860178 -17.092507 -4.240484
C 11.110507 -17.486501 -5.366258
C 10.001543 -18.305199 -5.236656
C 9.605323 -18.76352 -3.972213
C 10.332367 -18.39207 -2.853164
H 14.556571 -15.190002 -3.327964

H 13.989475 -16.144951 -1.101023
O 11.843263 -17.590779 -0.620063
H 10.055032 -18.718138 -1.851362
H 8.727739 -19.401922 -3.873411
H 9.434791 -18.593404 -6.122462
H 11.415577 -17.127918 -6.351066

TS_ortho_anion_conf3

C 0.960988 0.084629 0.750289
C 1.511099 -0.190954 -1.314772
C 3.412973 1.311021 -1.40013
C 3.406478 2.788477 1.689172
C 4.304265 2.03025 2.388349
C 4.09808 0.679568 2.673704
C 3.000995 0.013309 2.214258
C 2.059223 0.722546 1.423358
C 2.221442 2.151966 1.208458
C 6.12243 1.219248 3.342468
H 3.598586 3.83446 1.478283
H 2.865975 -1.038828 2.437151
H 6.626332 1.363562 4.304841
O 5.489638 2.434268 2.958354
O 5.134171 0.220629 3.471345
H 6.845611 0.929382 2.555615
N 1.198588 2.780681 0.570319
S 1.313562 4.279195 0.00723
O 2.627815 4.636734 -0.564847
O 0.120357 4.51399 -0.81283
C 0.346111 -3.53419 1.797836
C -1.043001 -3.538394 1.882448
C 1.031359 -2.385695 1.416669
H -1.573897 -4.442197 2.179754
H 2.110287 -2.432316 1.301639
C -1.747959 -2.379433 1.561351
C 0.343075 -1.193714 1.125026
H -2.836646 -2.368446 1.60773
C -1.062813 -1.233672 1.180402
H -1.616152 -0.327472 0.932124

H 0.90829 -4.442392 2.011642
C 2.129902 6.193044 1.85857
C 1.952905 6.988197 2.98977
C 1.113215 5.337614 1.449574
H 2.751182 7.660779 3.308222
C 0.768981 6.941025 3.726887
C -0.080658 5.280399 2.171247
H -0.870139 4.609763 1.836423
C -0.246194 6.074897 3.296249
H -1.180794 6.029125 3.858068
H 3.047779 6.233493 1.27443
C 0.585009 7.783351 4.953977
H -0.349414 8.359031 4.918548
H 1.40885 8.49515 5.081585
H 0.539576 7.17044 5.864865
H 0.22525 0.820356 0.426683
H 3.826147 2.308605 -1.545029
C 2.094962 1.06606 -1.66068
C 2.37439 -1.359057 -1.158735
C 3.764275 -1.052709 -0.772735
C 4.2554 0.278793 -0.871354
C 5.574217 0.531563 -0.442452
C 6.366426 -0.485811 0.067016
C 5.876824 -1.796072 0.154692
C 4.586673 -2.068329 -0.277054
H 1.453109 1.870488 -2.015069
H 0.499607 -0.416787 -1.654192
O 1.956878 -2.525098 -1.257674
H 4.165681 -3.073278 -0.231677
H 6.502997 -2.588395 0.563174
H 7.382355 -0.263618 0.397539
H 5.958156 1.551363 -0.502076

TS_ortho_anion_conf4
C 1.195382 0.916018 0.860911
C 1.470505 -0.149975 -1.10925
C 3.373549 0.829771 -2.260922
C 2.022419 -1.868032 3.249162

C 0.752355 -2.230053 3.589166
C -0.387048 -1.570977 3.114705
C -0.299519 -0.529409 2.242527
C 0.992513 -0.111262 1.814959
C 2.185566 -0.76673 2.351727
C -1.045195 -3.300554 4.329006
H 2.886505 -2.417596 3.604089
H -1.191844 -0.00658 1.914514
H -1.496262 -3.394244 5.324956
O 0.365766 -3.22221 4.46055
O -1.511286 -2.117844 3.711336
H -1.30371 -4.177334 3.70692
N 3.365183 -0.238972 1.961791
S 4.787389 -0.718242 2.566727
O 4.966772 -2.174662 2.727729
O 5.821101 0.033836 1.854378
C -1.96945 2.564275 -0.473329
C -1.562005 3.895888 -0.53542
C -1.101081 1.578687 -0.018495
H -2.24426 4.665358 -0.894537
H -1.423293 0.539192 -0.031472
C -0.263152 4.224511 -0.15281
C 0.205625 1.892337 0.401245
H 0.079633 5.257291 -0.212518
C 0.604604 3.237614 0.299364
H 1.623622 3.497251 0.585905
H -2.972069 2.285946 -0.797359
C 4.676564 -0.889331 5.344915
C 4.632973 -0.342234 6.626384
C 4.763012 -0.050732 4.239556
H 4.568231 -1.001398 7.493758
C 4.671462 1.038662 6.823307
C 4.807502 1.333309 4.416396
H 4.884974 1.974918 3.540386
C 4.763114 1.867441 5.696037
H 4.801831 2.949691 5.832379
H 4.664764 -1.966396 5.189269
C 4.610118 1.627932 8.201123

H 5.43975 2.323419 8.385807
H 4.650058 0.851476 8.973913
H 3.684179 2.198166 8.358732
H 2.220512 1.282712 0.837946
H 3.807573 1.612603 -2.885061
C 2.037622 0.83047 -1.969322
C 2.254497 -1.289756 -0.667127
C 3.69543 -1.253566 -0.980792
C 4.234788 -0.207103 -1.774984
C 5.611899 -0.235846 -2.075363
C 6.421868 -1.252867 -1.604784
C 5.880379 -2.280395 -0.819293
C 4.52921 -2.27951 -0.522584
H 1.393322 1.621699 -2.358827
H 0.393198 -0.306402 -1.112157
O 1.733001 -2.231961 -0.040273
H 4.08117 -3.045122 0.108171
H 6.526342 -3.06061 -0.418878
H 7.487548 -1.2479 -1.834933
H 6.033388 0.567949 -2.682214

TS_ortho_anion_conf5

C -1.572754 1.418246 -0.662342
C -0.792975 1.950277 1.350505
C 1.459546 2.861808 1.190468
C -1.665702 -2.336669 -0.43935
C -2.998052 -2.495457 -0.204654
C -3.900998 -1.425074 -0.149057
C -3.495607 -0.136086 -0.307147
C -2.111997 0.114669 -0.532166
C -1.17596 -1.004163 -0.623493
C -4.999681 -3.284847 0.340283
H -0.986454 -3.181956 -0.469961
H -4.216686 0.674655 -0.304529
H -5.730318 -3.890631 -0.210527
O -3.689351 -3.672521 -0.041046
O -5.18285 -1.920454 0.018983
H -5.120902 -3.434371 1.428964

N 0.105829 -0.690912 -0.886385
S 1.183753 -1.838619 -1.254269
O 0.985808 -2.352752 -2.623424
O 1.404401 -2.863256 -0.213971
C -4.100959 4.264153 -0.49765
C -3.619929 5.097645 -1.505886
C -3.462029 3.064289 -0.205839
H -4.12195 6.037869 -1.730339
H -3.824863 2.454731 0.619757
C -2.476811 4.721003 -2.207765
C -2.323872 2.650065 -0.922362
H -2.075953 5.367781 -2.987864
C -1.838825 3.521965 -1.913507
H -0.939981 3.235171 -2.459901
H -4.977432 4.557588 0.079645
C 3.878946 -1.481687 -1.040188
C 5.069222 -0.771431 -1.132075
C 2.667155 -0.838829 -1.285569
H 6.015825 -1.274064 -0.924978
C 5.074262 0.588939 -1.455069
C 2.65271 0.510141 -1.630263
H 1.699476 1.004309 -1.800954
C 3.850182 1.212509 -1.709249
H 3.83247 2.277717 -1.947715
H 3.869148 -2.531513 -0.752736
C 6.35261 1.371887 -1.459585
H 7.209189 0.757887 -1.764467
H 6.300783 2.236689 -2.132184
H 6.58284 1.762176 -0.456643
H -0.574552 1.42266 -1.096124
H 2.137491 3.691252 0.980564
C 0.104247 3.033851 1.129484
C -0.295376 0.66251 1.80736
C 1.170378 0.499733 1.839023
C 2.025713 1.599727 1.563715
C 3.418862 1.409337 1.67322
C 3.944504 0.180937 2.027069
C 3.091848 -0.904363 2.282259

C 1.721242 -0.737297 2.195515
H -0.31058 4.009684 0.86682
H -1.823556 2.165038 1.627716
O -1.072622 -0.251482 2.140675
H 1.031494 -1.562532 2.3614
H 3.508889 -1.882801 2.519487
H 5.026239 0.05021 2.082645
H 4.081987 2.249015 1.452031

TS_ortho_anion_conf6
C -1.391236 1.448013 -0.127153
C -0.601013 2.294564 1.666885
C 1.40213 3.319876 0.758976
C 1.089795 2.795237 -2.621956
C 1.840963 1.668491 -2.805254
C 1.506984 0.434186 -2.241499
C 0.431816 0.292562 -1.415994
C -0.352271 1.439282 -1.120838
C -0.067027 2.69975 -1.786519
C 3.459961 0.229696 -3.2326
H 1.348493 3.732151 -3.105767
H 0.197518 -0.673375 -0.983428
H 3.843498 -0.265022 -4.132063
O 2.966738 1.517513 -3.581035
O 2.397868 -0.52627 -2.69375
H 4.257773 0.346054 -2.473531
N -0.938348 3.716765 -1.570141
S -0.568319 5.216003 -2.022119
O -0.727383 5.45577 -3.469693
O 0.668967 5.740173 -1.405581
C -2.415488 -2.012737 1.119084
C -3.800065 -1.873395 1.140381
C -1.600405 -0.954672 0.731609
H -4.432895 -2.705622 1.447085
H -0.522541 -1.076559 0.783491
C -4.366112 -0.650194 0.785097
C -2.151199 0.27692 0.333277
H -5.447583 -0.518015 0.80911

C -3.551056 0.405318 0.398683
H -3.996377 1.360497 0.118096
H -1.957107 -2.951678 1.427088
C -1.667131 6.991851 -0.219355
C -2.726011 7.671533 0.380459
C -1.926212 6.061456 -1.217556
H -2.524029 8.400747 1.166616
C -4.046394 7.434838 -0.004455
C -3.237758 5.815711 -1.624718
H -3.422341 5.088031 -2.412625
C -4.283993 6.49563 -1.017851
H -5.310474 6.299893 -1.332677
H -0.634268 7.167972 0.074787
C -5.186508 8.15859 0.648071
H -4.830914 8.901945 1.370816
H -5.811334 8.683562 -0.086905
H -5.850927 7.469368 1.186905
H -2.02692 2.326347 -0.239948
H 1.897993 4.162561 0.279495
C 0.114574 3.439268 1.196273
C 0.152305 1.142435 2.160471
C 1.499695 0.991247 1.580822
C 2.092381 2.068017 0.864383
C 3.359737 1.859282 0.283858
C 4.00657 0.63881 0.401838
C 3.419665 -0.41406 1.116947
C 2.179449 -0.223525 1.708879
H -0.413245 4.382543 1.064072
H -1.569984 2.437311 2.147131
O -0.330608 0.305832 2.941696
H 1.687228 -1.01332 2.277336
H 3.931323 -1.372254 1.199428
H 4.984273 0.499905 -0.06241
H 3.8182 2.673675 -0.279587

TS_ortho_anion_conf7

C -1.077515 0.935841 0.26394
C -0.230769 2.072691 2.00603

C 0.960984 4.036312 1.18243
C -4.693688 2.005496 0.492312
C -4.813065 2.5745 -0.745448
C -3.764189 2.632759 -1.662781
C -2.532445 2.126947 -1.366997
C -2.329494 1.522983 -0.093758
C -3.428851 1.450092 0.859911
C -5.494119 3.712553 -2.525913
H -5.516049 2.013687 1.197854
H -1.743488 2.122967 -2.111375
H -6.203756 3.467067 -3.325977
O -5.938687 3.12531 -1.310143
O -4.221406 3.190028 -2.847011
H -5.430858 4.809004 -2.394887
N -3.145655 0.854272 2.04367
S -4.281515 0.57065 3.163885
O -5.232408 1.67468 3.40041
O -3.596026 -0.006187 4.319988
C 1.750753 1.843263 -2.096362
C 2.492794 0.66527 -2.074945
C 0.569897 1.946692 -1.370055
H 3.422881 0.592222 -2.637511
H 0.037967 2.894409 -1.342673
C 2.049958 -0.409127 -1.304427
C 0.10125 0.8684 -0.594976
H 2.634617 -1.327452 -1.26037
C 0.876711 -0.306902 -0.570164
H 0.554921 -1.126536 0.069476
H 2.107723 2.702459 -2.66365
C -6.565746 -0.559667 2.046567
C -7.280772 -1.604709 1.4636
C -5.249148 -0.762946 2.443341
H -8.315334 -1.445824 1.1549
C -6.696734 -2.856481 1.266591
C -4.647804 -2.00956 2.259363
H -3.61804 -2.153367 2.581859
C -5.368959 -3.042053 1.677675
H -4.898845 -4.016734 1.536836

H -7.023909 0.412795 2.216403
C -7.460942 -3.9769 0.626457
H -8.508228 -3.70349 0.453217
H -7.031309 -4.258765 -0.344642
H -7.452338 -4.883527 1.245999
H -1.142402 0.136557 1.003676
H 0.961011 5.070942 0.836482
C -0.195594 3.43009 1.589337
C 0.999344 1.306557 2.129057
C 2.237562 1.988473 1.694112
C 2.208758 3.330334 1.228458
C 3.418432 3.920045 0.811632
C 4.608759 3.213251 0.848745
C 4.63001 1.888987 1.307535
C 3.450206 1.291779 1.722968
H -1.137191 3.981615 1.558009
H -1.099083 1.699423 2.549828
O 1.016886 0.121662 2.512265
H 3.417432 0.261683 2.076519
H 5.567477 1.33361 1.328678
H 5.532295 3.690491 0.519194
H 3.403139 4.950221 0.45063

TS_ortho_anion_conf8

C 12.327617 -14.379704 -1.120393
C 13.206714 -16.053793 -2.286861
C 12.191158 -16.223049 -4.494136
C 10.426013 -16.268742 1.520773
C 11.307579 -16.127213 2.549402
C 12.501792 -15.401504 2.442735
C 12.874025 -14.799493 1.280899
C 12.016384 -14.922752 0.150841
C 10.755805 -15.652459 0.27147
C 12.434689 -16.333704 4.449632
H 9.512029 -16.843372 1.626606
H 13.780317 -14.204976 1.231849
H 12.267558 -15.937167 5.458932
O 11.181983 -16.594174 3.83663

O 13.128757 -15.3749 3.676986
H 13.018025 -17.271718 4.49763
N 9.979293 -15.688284 -0.826874
S 8.462217 -16.24369 -0.755548
O 7.558034 -15.273941 -0.107345
O 8.315434 -17.645617 -0.315279
C 15.60619 -12.448915 -1.316249
C 15.226289 -11.370643 -2.113486
C 14.687374 -13.434573 -0.973913
H 15.949196 -10.60129 -2.381642
H 15.01931 -14.293372 -0.393281
C 13.914831 -11.301692 -2.578871
C 13.350411 -13.369543 -1.408528
H 13.603686 -10.474986 -3.217106
C 12.998244 -12.288864 -2.236667
H 11.975248 -12.235048 -2.61001
H 16.635119 -12.53216 -0.967077
C 7.086326 -17.112095 -2.94422
C 6.681731 -17.084661 -4.272841
C 8.069878 -16.230161 -2.500816
H 5.921603 -17.786553 -4.620907
C 7.255521 -16.192042 -5.183277
C 8.63904 -15.319737 -3.387265
H 9.420146 -14.65153 -3.032949
C 8.232399 -15.308726 -4.71698
H 8.70356 -14.615482 -5.416404
H 6.668303 -17.830411 -2.241355
C 6.881581 -16.230877 -6.634443
H 5.831974 -16.514969 -6.78069
H 7.040371 -15.262465 -7.1242
H 7.488784 -16.967897 -7.181539
H 11.463181 -14.317413 -1.778716
H 12.099893 -15.884187 -5.527837
C 13.10792 -15.65785 -3.651458
C 12.437476 -17.183692 -1.790199
C 11.439229 -17.755287 -2.713688
C 11.350031 -17.296415 -4.054847
C 10.428139 -17.92355 -4.918357

C 9.619088 -18.951376 -4.471631
C 9.702854 -19.387272 -3.140082
C 10.610836 -18.797154 -2.278855
H 13.758891 -14.857568 -4.010887
H 14.131758 -15.860575 -1.746703
O 12.612844 -17.631528 -0.641638
H 10.684666 -19.092959 -1.234118
H 9.035038 -20.168647 -2.778135
H 8.896258 -19.406034 -5.150709
H 10.349265 -17.568167 -5.948445

TS_ortho_anion_conf9
C 12.52555 -14.84145 -1.359077
C 13.471308 -16.173962 -2.73169
C 12.232515 -15.604582 -4.739292
C 10.312318 -12.938648 -3.740705
C 9.121062 -13.54522 -3.456326
C 8.971789 -14.47855 -2.426965
C 10.031108 -14.888221 -1.673282
C 11.314519 -14.353412 -1.961343
C 11.453525 -13.309226 -2.963046
C 7.074175 -14.368609 -3.533772
H 10.393659 -12.187865 -4.520787
H 9.892379 -15.620739 -0.886198
H 6.069287 -13.974101 -3.345524
O 7.8973 -13.327516 -4.045575
O 7.635096 -14.830101 -2.324655
H 7.043597 -15.19471 -4.270648
N 12.679898 -12.744436 -3.08949
S 13.022987 -11.799643 -4.345642
O 12.506585 -10.423947 -4.210033
O 12.820101 -12.447573 -5.65888
C 11.981391 -17.155798 1.62891
C 12.946807 -16.667612 2.504561
C 11.825894 -16.603111 0.362276
H 13.064884 -17.106815 3.494661
H 11.1042 -17.045355 -0.318409
C 13.774417 -15.624732 2.091873

C 12.624767 -15.525637 -0.06172
H 14.54518 -15.238813 2.758672
C 13.618039 -15.074089 0.826972
H 14.264818 -14.256185 0.507397
H 11.350176 -17.992889 1.924679
C 15.654527 -12.187566 -5.079243
C 17.032651 -12.084703 -4.896587
C 14.79441 -11.702704 -4.102349
H 17.709263 -12.468658 -5.661689
C 17.567197 -11.501727 -3.746856
C 15.306826 -11.106917 -2.949469
H 14.618623 -10.728464 -2.196127
C 16.680316 -11.013568 -2.7769
H 17.081121 -10.552769 -1.872401
H 15.228292 -12.644482 -5.97022
C 19.049938 -11.403519 -3.543789
H 19.601411 -11.763822 -4.419838
H 19.368823 -10.370002 -3.353259
H 19.382283 -11.996719 -2.680905
H 13.343527 -14.136997 -1.510265
H 12.099621 -14.9822 -5.622964
C 13.362979 -15.490023 -3.982562
C 12.650042 -17.361672 -2.502336
C 11.384504 -17.391671 -3.258782
C 11.181128 -16.495499 -4.344782
C 9.935697 -16.526042 -5.004433
C 8.935941 -17.397889 -4.601438
C 9.1472 -18.282641 -3.53527
C 10.372327 -18.279738 -2.883975
H 14.132428 -14.774559 -4.268552
H 14.433493 -16.186225 -2.21783
O 12.946611 -18.237015 -1.672452
H 10.582304 -18.953599 -2.052656
H 8.353927 -18.959529 -3.220181
H 7.977572 -17.397089 -5.123197
H 9.763583 -15.834234 -5.830759

TS_ortho_anion_conf10

C 11.897654 -15.555392 -1.304059
C 13.469255 -16.987105 -2.029995
C 14.13347 -16.375171 -4.296942
C 13.919585 -13.460869 1.104411
C 13.707097 -12.237234 0.532171
C 12.945668 -12.056408 -0.621702
C 12.36652 -13.111013 -1.264071
C 12.538534 -14.41771 -0.724526
C 13.330008 -14.606068 0.484441
C 13.531217 -10.055455 0.141117
H 14.55175 -13.574283 1.977206
H 11.734242 -12.950196 -2.130527
H 12.762455 -9.536769 0.743243
O 14.185646 -11.019879 0.95423
O 12.92443 -10.711346 -0.95379
H 14.271173 -9.332924 -0.227855
N 13.438877 -15.882855 0.92479
S 14.149268 -16.264345 2.330102
O 15.387488 -15.524701 2.645564
O 14.185439 -17.724791 2.399503
C 10.481809 -14.884459 -4.758616
C 9.340473 -15.681316 -4.730086
C 11.313622 -14.804191 -3.647608
H 8.696293 -15.749564 -5.606023
H 12.235751 -14.231454 -3.70742
C 9.044167 -16.412641 -3.580111
C 11.02374 -15.525502 -2.473457
H 8.166956 -17.058377 -3.554692
C 9.876042 -16.34162 -2.471629
H 9.673251 -16.948005 -1.590712
H 10.743876 -14.339109 -5.664943
C 13.183847 -14.650069 4.380321
C 12.210321 -14.245069 5.292334
C 12.938731 -15.737406 3.549609
H 12.403774 -13.393865 5.947259
C 10.988029 -14.911485 5.386441
C 11.723025 -16.419649 3.629853

H 11.550852 -17.272341 2.975395
C 10.762217 -16.007701 4.541641
H 9.813909 -16.543802 4.606022
H 14.145164 -14.143734 4.31804
C 9.936692 -14.470214 6.360651
H 10.292217 -13.648825 6.993504
H 9.029403 -14.120962 5.849011
H 9.625053 -15.28856 7.023451
H 11.692852 -16.370242 -0.607747
H 14.736665 -15.775455 -4.980055
C 14.270042 -16.249246 -2.941453
C 12.546735 -18.002478 -2.513688
C 12.429792 -18.130344 -3.981953
C 13.211607 -17.323863 -4.851366
C 13.041681 -17.480023 -6.241285
C 12.135696 -18.39389 -6.753079
C 11.36683 -19.186105 -5.889371
C 11.519238 -19.046481 -4.518979
H 14.983352 -15.530829 -2.532948
H 13.762586 -17.050077 -0.981551
O 11.838579 -18.684921 -1.749464
H 10.935184 -19.632292 -3.809815
H 10.650105 -19.899518 -6.295686
H 12.021275 -18.496101 -7.832772
H 13.637029 -16.861514 -6.915692

TS_ortho_anion_conf11
C 11.420546 -15.28304 -2.00975
C 13.508646 -16.137581 -2.049382
C 14.227183 -16.320165 -4.363938
C 9.518129 -18.240126 -0.67751
C 9.401492 -17.840106 0.621009
C 9.872522 -16.609256 1.090685
C 10.517363 -15.731647 0.273832
C 10.706156 -16.090537 -1.090852
C 10.168278 -17.355158 -1.593479
C 9.089737 -17.753006 2.816245
H 9.175205 -19.214046 -1.006772

H 10.842089 -14.76583 0.646499
H 8.188757 -17.661013 3.435843
O 8.782507 -18.506483 1.653504
O 9.523488 -16.466374 2.42363
H 9.891763 -18.265103 3.379389
N 10.314873 -17.564049 -2.918977
S 9.649528 -18.820616 -3.691382
O 9.743322 -20.125796 -3.008304
O 10.084487 -18.733963 -5.085995
C 12.788312 -12.017321 -0.604109
C 12.555235 -11.155968 -1.674956
C 12.424461 -13.35673 -0.680828
H 12.843766 -10.10747 -1.612504
H 12.667173 -14.021618 0.145963
C 11.968761 -11.660509 -2.833817
C 11.803546 -13.881189 -1.830422
H 11.796369 -11.006705 -3.688509
C 11.607827 -13.000429 -2.909885
H 11.160883 -13.393648 -3.822886
H 13.273732 -11.645649 0.298083
C 6.985112 -19.135366 -2.94111
C 5.638964 -18.773138 -2.943026
C 7.895986 -18.408637 -3.699223
H 4.924197 -19.345854 -2.349607
C 5.184592 -17.687689 -3.692956
C 7.459814 -17.322562 -4.460134
H 8.185193 -16.770352 -5.055068
C 6.117901 -16.970497 -4.454945
H 5.778444 -16.123133 -5.053371
H 7.338886 -19.992123 -2.370873
C 3.738784 -17.28899 -3.683584
H 3.126912 -18.004349 -3.121825
H 3.592263 -16.301831 -3.223855
H 3.325613 -17.224284 -4.699103
H 11.254397 -15.565926 -3.048142
H 14.638674 -15.872878 -5.270238
C 14.098246 -15.58372 -3.219082
C 13.200226 -17.555048 -1.975468

C 13.333677 -18.319923 -3.229734
C 13.848324 -17.701477 -4.39999
C 13.987964 -18.483125 -5.564843
C 13.628183 -19.818111 -5.573496
C 13.119942 -20.419557 -4.413301
C 12.985952 -19.675042 -3.255043
H 14.405248 -14.535632 -3.205618
H 13.665779 -15.650839 -1.088418
O 12.825107 -18.082003 -0.910515
H 12.567629 -20.102625 -2.34562
H 12.803533 -21.461641 -4.431577
H 13.728743 -20.401074 -6.489299
H 14.379086 -18.012026 -6.468796

TS_ortho_anion_conf12
C 12.229711 -15.198856 -2.048771
C 13.561808 -16.884049 -1.905953
C 14.266151 -17.003749 -4.223214
C 12.794909 -14.270559 -5.652999
C 11.647653 -14.786947 -6.188624
C 10.624272 -15.330233 -5.410464
C 10.737393 -15.434584 -4.055869
C 11.932577 -14.980715 -3.438348
C 12.95523 -14.320431 -4.234456
C 10.12511 -15.662583 -7.526991
H 13.573461 -13.85861 -6.285106
H 9.935068 -15.870205 -3.471545
H 9.394816 -15.261529 -8.238931
O 11.274135 -14.823607 -7.512356
O 9.564236 -15.677808 -6.232505
H 10.434669 -16.686832 -7.811521
N 13.992223 -13.774891 -3.544376
S 15.335315 -13.261774 -4.257654
O 15.804564 -14.095749 -5.383106
O 16.288852 -12.951391 -3.187377
C 9.284648 -16.471593 0.019329
C 9.433952 -15.702382 1.1694
C 10.180127 -16.35035 -1.037726

H 8.730438 -15.803811 1.995184
H 10.071691 -17.008058 -1.895051
C 10.506536 -14.816717 1.26133
C 11.247957 -15.435848 -0.982596
H 10.647747 -14.216987 2.160168
C 11.400169 -14.696876 0.205602
H 12.237318 -14.002004 0.279019
H 8.47184 -17.193217 -0.052555
C 14.974039 -11.474278 -6.36108
C 14.623299 -10.23827 -6.901609
C 14.908363 -11.671417 -4.986513
H 14.676911 -10.084967 -7.980779
C 14.2025 -9.187388 -6.085633
C 14.494373 -10.630301 -4.153361
H 14.452676 -10.796739 -3.078301
C 14.146505 -9.404236 -4.701433
H 13.823885 -8.591815 -4.047873
H 15.312299 -12.294266 -6.992381
C 13.811071 -7.861637 -6.667292
H 14.001311 -7.822834 -7.746127
H 12.743595 -7.650123 -6.515742
H 14.362089 -7.032823 -6.203025
H 13.04829 -14.557879 -1.722317
H 14.931689 -16.693273 -5.027832
C 14.517038 -16.623804 -2.935835
C 12.587415 -17.95686 -2.092638
C 12.267862 -18.263579 -3.499202
C 13.091601 -17.760344 -4.544004
C 12.715737 -18.035705 -5.874386
C 11.574756 -18.771317 -6.156024
C 10.773641 -19.270936 -5.120314
C 11.134166 -19.022245 -3.803812
H 15.386597 -16.008841 -2.711164
H 13.839003 -16.699633 -0.867413
O 12.006612 -18.513372 -1.145714
H 10.542989 -19.393534 -2.966103
H 9.874991 -19.842275 -5.35002
H 11.30236 -18.966915 -7.194431

H 13.331805 -17.642478 -6.684797

TS_ortho_anion_conf13

C -0.981415 -0.290653 0.498137
C -1.831472 -0.418373 -1.57527
C -3.862456 0.928558 -1.701058
C 1.908329 2.069297 -0.102322
C 1.548612 3.053299 0.776441
C 0.396071 2.993254 1.55962
C -0.460027 1.934134 1.484455
C -0.154798 0.871206 0.587407
C 1.054154 0.929543 -0.224132
C 1.36726 4.951313 1.913897
H 2.789016 2.165199 -0.726337
H -1.318427 1.869087 2.144501
H 1.939529 5.360136 2.756075
O 2.238503 4.204066 1.075285
O 0.356585 4.093627 2.402101
H 0.915643 5.771604 1.325167
N 1.262411 -0.125211 -1.048653
S 2.614953 -0.288601 -1.925895
O 3.101928 0.940489 -2.582903
O 2.438389 -1.484642 -2.748934
C -4.357402 0.134087 2.173389
C -4.568339 -1.152646 2.661609
C -3.185091 0.44772 1.495199
H -5.492891 -1.397092 3.183481
H -3.063775 1.437219 1.061398
C -3.598507 -2.13248 2.451835
C -2.190413 -0.526038 1.282342
H -3.76305 -3.148878 2.80818
C -2.431171 -1.82636 1.766524
H -1.695732 -2.601149 1.557861
H -5.125155 0.897329 2.297896
C 4.898981 0.158907 -0.400368
C 5.829444 -0.186311 0.578696
C 3.855872 -0.710952 -0.695481
H 6.650067 0.495136 0.809065

C 5.731669 -1.392354 1.273516
C 3.741902 -1.924246 -0.013843
H 2.92122 -2.595255 -0.261482
C 4.673224 -2.256724 0.958837
H 4.584586 -3.206106 1.489511
H 4.980837 1.091787 -0.955016
C 6.727662 -1.760431 2.332547
H 7.51523 -1.003757 2.425904
H 6.253602 -1.864575 3.318002
H 7.215122 -2.72104 2.118235
H -0.464352 -1.1978 0.181386
H -4.355759 1.891694 -1.839558
C -2.502326 0.814748 -1.791231
C -2.588219 -1.642828 -1.365978
C -4.057441 -1.495023 -1.286377
C -4.675132 -0.226349 -1.449849
C -6.077945 -0.146965 -1.345336
C -6.840928 -1.273371 -1.086486
C -6.224893 -2.521957 -0.92478
C -4.846078 -2.620406 -1.025085
H -1.89619 1.700004 -1.99311
H -0.794286 -0.529996 -1.892905
O -2.041623 -2.748484 -1.192893
H -4.323727 -3.567829 -0.895964
H -6.827859 -3.405124 -0.714601
H -7.925214 -1.186622 -1.008428
H -6.559133 0.825335 -1.467505

TS_ortho_anion_conf14
C -1.63379 -0.119074 1.074473
C -1.681357 0.332496 -1.136002
C -3.931898 1.027863 -1.734543
C -1.014225 -3.826519 0.984687
C 0.324208 -3.816861 1.245893
C 1.047222 -2.647753 1.506829
C 0.456368 -1.421495 1.484566
C -0.934117 -1.345509 1.188451
C -1.700229 -2.571971 0.965784

C 2.46891 -4.343788 1.463114
H -1.543325 -4.743884 0.75393
H 1.0244 -0.529512 1.727212
H 3.026701 -4.879573 2.241509
O 1.167271 -4.898763 1.354911
O 2.354671 -2.98106 1.820522
H 2.984942 -4.435665 0.489539
N -3.027856 -2.404894 0.787026
S -4.063042 -3.644156 0.684868
O -3.633024 -4.780728 -0.153337
O -5.386209 -3.066572 0.445577
C 0.446961 3.01616 1.667915
C -0.460859 3.799534 2.378711
C 0.097769 1.740776 1.239302
H -0.18504 4.799522 2.71106
H 0.803127 1.170443 0.637824
C -1.733035 3.293768 2.637937
C -1.171338 1.197967 1.516427
H -2.462916 3.899011 3.175217
C -2.08136 2.019312 2.20688
H -3.081696 1.632609 2.401072
H 1.435557 3.408411 1.430176
C -3.591737 -5.538781 2.67079
C -3.616573 -5.998381 3.986584
C -4.104983 -4.282644 2.369091
H -3.216438 -6.986032 4.222128
C -4.145807 -5.216568 5.014142
C -4.643574 -3.487844 3.382397
H -5.049319 -2.510347 3.127406
C -4.662351 -3.95427 4.688933
H -5.086925 -3.332774 5.479383
H -3.194257 -6.151816 1.864102
C -4.156593 -5.703606 6.43262
H -3.841989 -6.751444 6.501667
H -3.479233 -5.117238 7.068915
H -5.154474 -5.625462 6.884312
H -2.715816 -0.239452 1.102086
H -4.706818 1.793911 -1.792891

C -2.681757 1.333893 -1.272859
C -1.895904 -1.005382 -1.659083
C -3.258689 -1.311268 -2.133742
C -4.252641 -0.297629 -2.174108
C -5.530001 -0.63126 -2.668514
C -5.813669 -1.914032 -3.099429
C -4.826242 -2.908535 -3.053549
C -3.562124 -2.601085 -2.583126
H -2.450665 2.352689 -0.954142
H -0.637606 0.627175 -1.042171
O -0.97763 -1.847286 -1.682784
H -2.781633 -3.355591 -2.504709
H -5.061884 -3.927369 -3.35819
H -6.812541 -2.155392 -3.463672
H -6.299558 0.142652 -2.698046

TS_ortho_anion_conf15
C -1.593548 0.422585 0.500295
C -1.553426 -0.297019 -1.530447
C -3.792808 0.35004 -2.198514
C -4.99336 2.038048 0.52547
C -5.682677 1.072913 1.206116
C -5.066938 -0.046459 1.768285
C -3.731708 -0.276563 1.617244
C -2.964787 0.651004 0.864345
C -3.58398 1.868896 0.364546
C -7.24269 -0.270132 2.006504
H -5.502488 2.893118 0.095302
H -3.275684 -1.159283 2.050506
H -7.954475 -0.221666 2.83847
O -7.026724 1.041109 1.498188
O -6.010964 -0.78095 2.467629
H -7.63032 -0.910127 1.190449
N -2.746667 2.774657 -0.207525
S -3.273946 4.046955 -1.031709
O -4.461106 3.801045 -1.875804
O -2.098454 4.658286 -1.66074
C 0.073555 -2.499577 2.305526

C 1.316556 -1.941646 2.589429
C -0.890642 -1.7737 1.61436
H 2.067814 -2.516824 3.129696
H -1.826513 -2.259363 1.355123
C 1.596426 -0.646829 2.155571
C -0.644043 -0.451854 1.200355
H 2.569453 -0.198898 2.355737
C 0.633572 0.076527 1.465188
H 0.85373 1.090143 1.128831
H -0.146685 -3.522749 2.607522
C -5.131088 5.656085 0.278457
C -5.522574 6.556152 1.268354
C -3.810691 5.225469 0.219301
H -6.55934 6.894003 1.312887
C -4.610445 7.035233 2.209424
C -2.882418 5.69726 1.14944
H -1.851189 5.353578 1.089546
C -3.282494 6.591727 2.131379
H -2.55498 6.958821 2.857467
H -5.836927 5.284941 -0.462769
C -5.033914 7.992449 3.283465
H -6.077523 8.30331 3.157677
H -4.943366 7.547379 4.283886
H -4.41583 8.900273 3.289089
H -1.126839 1.344687 0.154862
H -4.486588 1.092696 -2.590936
C -2.449317 0.594719 -2.195484
C -1.96722 -1.677069 -1.287827
C -3.425082 -1.879867 -1.199547
C -4.315416 -0.856324 -1.62752
C -5.69942 -1.075736 -1.474963
C -6.17876 -2.252178 -0.919798
C -5.294905 -3.259089 -0.508373
C -3.929497 -3.06799 -0.663019
H -2.068842 1.536548 -2.586812
H -0.478801 -0.161348 -1.657149
O -1.15778 -2.598538 -1.08742
H -3.205578 -3.824953 -0.359828

H -5.67893 -4.177901 -0.066675
H -7.254635 -2.396307 -0.808297
H -6.391493 -0.292267 -1.788123

TS_ortho_anion_conf16
C 11.916972 -14.845851 -1.267183
C 13.17175 -16.692125 -1.494969
C 14.060945 -16.792405 -3.765166
C 14.302845 -12.652496 0.676014
C 14.376921 -11.608563 -0.204169
C 13.687456 -11.581952 -1.416154
C 12.893885 -12.620795 -1.805208
C 12.770462 -13.746976 -0.942668
C 13.482042 -13.770202 0.328681
C 14.94833 -9.764963 -1.301852
H 14.883376 -12.660708 1.590957
H 12.313653 -12.560611 -2.719723
H 14.717399 -8.706638 -1.126837
O 15.068296 -10.430479 -0.051368
O 13.900447 -10.36134 -2.038048
H 15.901577 -9.861256 -1.854507
N 13.304371 -14.880646 1.084709
S 13.908355 -15.016374 2.582191
O 15.290651 -14.529572 2.759554
O 13.591562 -16.368451 3.041292
C 10.752544 -14.832475 -4.877018
C 9.471686 -15.360477 -4.737882
C 11.555047 -14.625662 -3.760948
H 8.850108 -15.531593 -5.616093
H 12.576428 -14.276878 -3.891359
C 9.002577 -15.694354 -3.467875
C 11.094795 -14.947758 -2.469668
H 8.011091 -16.130252 -3.349141
C 9.80366 -15.497729 -2.351788
H 9.458374 -15.802614 -1.365683
H 11.143468 -14.603405 -5.868063
C 13.387464 -12.742435 4.099473
C 12.550337 -11.888981 4.816624

C 12.871207 -13.908316 3.546177
H 12.956004 -10.975038 5.253665
C 11.197031 -12.182373 4.988265
C 11.52047 -14.22207 3.709556
H 11.134556 -15.142145 3.274178
C 10.696985 -13.365219 4.425014
H 9.6421 -13.612764 4.55456
H 14.446925 -12.524884 3.978291
C 10.293742 -11.2589 5.750117
H 10.849357 -10.421346 6.187552
H 9.511678 -10.833157 5.106667
H 9.776121 -11.777118 6.56841
H 11.529164 -15.393793 -0.406987
H 14.810432 -16.507012 -4.504701
C 14.154618 -16.368725 -2.468023
C 12.080438 -17.596611 -1.821296
C 12.0078 -18.049374 -3.227162
C 12.98533 -17.647658 -4.176386
C 12.852968 -18.09835 -5.504622
C 11.79637 -18.909853 -5.882659
C 10.834151 -19.300992 -4.941502
C 10.947905 -18.868778 -3.629529
H 14.98354 -15.725799 -2.165797
H 13.397307 -16.555963 -0.436924
O 11.210388 -17.924891 -0.992814
H 10.218123 -19.139238 -2.867039
H 10.000291 -19.934129 -5.244088
H 11.714161 -19.244797 -6.917221
H 13.599402 -17.791613 -6.239827

TS_ortho_anion_conf17

C 11.593768 -14.623781 -2.070927
C 13.421063 -15.906089 -1.746825
C 14.106027 -16.844286 -3.881887
C 9.033237 -16.63633 -0.19168
C 8.994771 -15.899531 0.955163
C 9.733683 -14.725695 1.138688
C 10.577466 -14.254581 0.17976

C 10.696825 -14.986137 -1.035723
C 9.885105 -16.184725 -1.247653
C 8.681203 -15.182134 3.031291
H 8.477357 -17.561001 -0.294315
H 11.113566 -13.322468 0.324622
H 7.818145 -14.735841 3.541227
O 8.222573 -16.1222 2.072064
O 9.408538 -14.1698 2.365189
H 9.333715 -15.696695 3.760732
N 9.997841 -16.756059 -2.465489
S 9.069354 -17.990134 -2.948357
O 8.847798 -19.061881 -1.957724
O 9.531932 -18.36517 -4.285083
C 13.671257 -11.498974 -1.415612
C 13.664563 -10.912549 -2.680169
C 13.003105 -12.696326 -1.187062
H 14.191225 -9.975522 -2.856618
H 13.069395 -13.163171 -0.206133
C 12.993584 -11.553471 -3.719593
C 12.293681 -13.345799 -2.215171
H 12.99335 -11.120071 -4.719514
C 12.327799 -12.751596 -3.489977
H 11.814733 -13.255731 -4.308872
H 14.216654 -11.0263 -0.599011
C 6.394488 -17.50519 -2.339999
C 5.1691 -16.868208 -2.529596
C 7.459513 -17.217672 -3.185953
H 4.332841 -17.096651 -1.866657
C 4.989162 -15.940163 -3.555975
C 7.297562 -16.295818 -4.2217
H 8.139313 -16.089297 -4.880375
C 6.073787 -15.667505 -4.40163
H 5.948271 -14.949781 -5.214332
H 6.532136 -18.242405 -1.551371
C 3.674776 -15.244686 -3.751203
H 2.907096 -15.636342 -3.073724
H 3.754501 -14.164683 -3.565379
H 3.300598 -15.357592 -4.777578

H 11.379007 -15.119073 -3.016705
H 14.62254 -16.75354 -4.838828
C 14.138086 -15.823857 -2.972368
C 12.787968 -17.148979 -1.341507
C 12.753053 -18.224971 -2.350002
C 13.414696 -18.066485 -3.596602
C 13.383041 -19.137456 -4.512701
C 12.719756 -20.312612 -4.211082
C 12.067589 -20.458703 -2.978466
C 12.095981 -19.42551 -2.058993
H 14.681556 -14.904412 -3.200801
H 13.678754 -15.23422 -0.92998
O 12.287777 -17.281962 -0.208095
H 11.574498 -19.493637 -1.105994
H 11.514539 -21.370642 -2.757408
H 12.693571 -21.122918 -4.940058
H 13.886419 -19.020182 -5.474407

TS_para_anion_conf1
C 12.495247 -16.030478 -1.99853
C 14.09394 -17.12719 -0.892721
C 11.313113 -13.3674 0.40069
C 12.091541 -12.337979 -0.037832
C 12.972737 -12.450985 -1.120223
C 13.126424 -13.624763 -1.794862
C 12.364508 -14.756444 -1.382801
C 11.408484 -14.628555 -0.279841
C 12.955559 -10.332726 -0.456569
H 10.638199 -13.245807 1.241678
H 13.795554 -13.684574 -2.646645
H 12.297635 -9.670027 -1.048295
O 12.168883 -11.063404 0.471248
O 13.618108 -11.241515 -1.313927
H 13.698437 -9.737031 0.089678
N 10.657546 -15.70373 0.008352
S 9.444329 -15.581247 1.086024
O 8.384288 -14.648262 0.65647
O 9.919765 -15.435396 2.474099

C 14.634619 -16.149669 -5.155142
C 13.836244 -16.94152 -5.977408
C 14.222584 -15.82798 -3.866251
H 14.16271 -17.193929 -6.985331
H 14.890785 -15.252785 -3.228033
C 12.623779 -17.420911 -5.48537
C 12.992905 -16.283317 -3.357076
H 11.993009 -18.05447 -6.108131
C 12.215941 -17.102406 -4.195948
H 11.27348 -17.490938 -3.811076
H 15.597461 -15.787775 -5.514873
C 7.421807 -17.40544 0.67741
C 6.905679 -18.697873 0.593188
C 8.778746 -17.230785 0.917063
H 5.840796 -18.842275 0.401243
C 7.728452 -19.817007 0.739677
C 9.615581 -18.333819 1.075804
H 10.675123 -18.18212 1.260338
C 9.094744 -19.615689 0.980611
H 9.772171 -20.463192 1.089202
H 6.791059 -16.52829 0.548112
C 7.1697 -21.205428 0.638097
H 7.65174 -21.778834 -0.164945
H 7.328416 -21.775713 1.563228
H 6.091743 -21.194577 0.436769
H 11.719698 -16.729195 -1.682865
H 14.809713 -16.435401 -1.337321
C 13.983112 -18.433414 -1.444658
C 13.416244 -19.471436 -0.756009
C 12.97296 -19.335282 0.614647
C 13.220973 -18.019494 1.240718
C 13.788149 -16.95018 0.500817
C 13.996058 -15.716226 1.146748
C 13.622401 -15.535365 2.467536
C 13.055066 -16.590093 3.191277
C 12.868131 -17.818754 2.580344
H 14.305595 -18.582606 -2.477654
H 13.273467 -20.446529 -1.220342

O 12.412531 -20.261986 1.234861
H 12.419298 -18.658899 3.109354
H 12.736795 -16.434592 4.220682
H 13.754278 -14.561072 2.937668
H 14.429595 -14.890936 0.579556

TS_para_anion_conf2
C 12.186121 -16.800564 -1.109857
C 14.290611 -17.141841 -0.693632
C 10.552855 -19.709513 0.603841
C 10.494869 -19.207708 1.879669
C 10.89828 -17.908579 2.197565
C 11.396145 -17.056382 1.254716
C 11.520953 -17.536617 -0.080137
C 11.123882 -18.890859 -0.402692
C 10.60562 -19.017285 4.066468
H 10.242917 -20.719184 0.356969
H 11.785871 -16.084838 1.543912
H 9.899533 -19.007259 4.903313
O 10.083507 -19.83906 3.025974
O 10.747243 -17.703593 3.553993
H 11.596589 -19.402514 4.360303
N 11.379141 -19.371961 -1.663324
S 10.147696 -19.705564 -2.61726
O 8.976256 -20.298118 -1.942773
O 10.652752 -20.349231 -3.835455
C 11.441912 -13.07787 -0.812558
C 12.150873 -12.580841 -1.902317
C 11.436746 -14.440551 -0.528714
H 12.155057 -11.51323 -2.118731
H 10.832936 -14.808284 0.296043
C 12.833555 -13.473208 -2.730869
C 12.14889 -15.35229 -1.325943
H 13.368812 -13.107243 -3.606786
C 12.823213 -14.831116 -2.44816
H 13.341247 -15.526659 -3.109907
H 10.875974 -12.398724 -0.175451
C 8.850086 -17.293 -2.223129

C 8.642985 -15.939107 -2.472224
C 9.638516 -18.046747 -3.092281
H 8.042372 -15.347643 -1.778411
C 9.213872 -15.310136 -3.581371
C 10.208118 -17.436962 -4.21114
H 10.829517 -18.038993 -4.872376
C 9.990205 -16.08607 -4.451696
H 10.447359 -15.610287 -5.322125
H 8.415263 -17.776103 -1.349005
C 9.016076 -13.844928 -3.830407
H 9.970816 -13.339391 -4.027268
H 8.368764 -13.65686 -4.699352
H 8.558031 -13.349819 -2.96583
H 12.246861 -17.371194 -2.037831
H 14.631086 -16.584056 -1.566666
C 14.316449 -18.565842 -0.743367
C 14.116647 -19.317143 0.380405
C 13.997049 -18.720424 1.693148
C 14.283471 -17.269159 1.769993
C 14.450402 -16.501472 0.591245
C 14.660848 -15.114605 0.71523
C 14.714648 -14.513725 1.96215
C 14.554183 -15.281097 3.124987
C 14.335727 -16.644578 3.022013
H 14.332188 -19.05377 -1.717207
H 13.985494 -20.396086 0.318002
O 13.670504 -19.368385 2.705711
H 14.172534 -17.270896 3.898325
H 14.59064 -14.801547 4.1029
H 14.877114 -13.438512 2.036801
H 14.764459 -14.513386 -0.189616

TS_para_anion_conf3

C 12.055095 -17.244759 -1.017229
C 14.296331 -17.008134 -0.593616
C 11.932848 -16.839148 -4.741963
C 11.468004 -18.081756 -5.087892
C 11.253454 -19.093873 -4.153204

C 11.475322 -18.904061 -2.817123
C 11.938052 -17.628809 -2.392827
C 12.205549 -16.594004 -3.369265
C 10.570331 -19.823501 -6.134122
H 12.097144 -16.052353 -5.469904
H 11.237232 -19.685438 -2.10339
H 9.480401 -19.736008 -6.299201
O 11.178819 -18.558632 -6.34604
O 10.821928 -20.235969 -4.80387
H 11.00061 -20.553986 -6.831134
N 12.77458 -15.429883 -2.922434
S 12.090313 -14.020813 -3.25315
O 11.427897 -13.922714 -4.568455
O 13.017931 -12.956013 -2.853907
C 11.414553 -19.94664 1.573205
C 10.924932 -19.171902 2.621097
C 11.785725 -19.354064 0.371659
H 10.657686 -19.637749 3.568324
H 12.236101 -19.968263 -0.40422
C 10.815814 -17.791096 2.45967
C 11.648123 -17.965143 0.170784
H 10.470969 -17.16724 3.283762
C 11.179353 -17.199375 1.259189
H 11.12163 -16.1157 1.140679
H 11.546471 -21.019744 1.704459
C 9.634341 -14.813067 -2.25448
C 8.709011 -14.98915 -1.232145
C 10.764767 -14.022877 -2.037834
H 7.833112 -15.617943 -1.399705
C 8.886585 -14.386183 0.018364
C 10.946395 -13.396428 -0.807064
H 11.831895 -12.781159 -0.65902
C 10.010479 -13.575064 0.206577
H 10.164426 -13.091807 1.173408
H 9.495792 -15.292009 -3.22304
C 7.924911 -14.643866 1.139202
H 7.947513 -13.842493 1.88739
H 6.892836 -14.740987 0.779944

H 8.16701 -15.579909 1.663357
H 12.03134 -16.162506 -0.895691
H 14.366645 -16.505384 -1.556348
C 14.713788 -18.350397 -0.446721
C 14.835378 -18.94145 0.784333
C 14.60355 -18.213692 2.017451
C 14.310729 -16.770288 1.864861
C 14.19133 -16.185411 0.579937
C 13.886268 -14.812087 0.477558
C 13.718206 -14.046582 1.618364
C 13.847429 -14.626618 2.890923
C 14.140261 -15.974781 3.005744
H 14.870973 -18.943475 -1.350631
H 15.098225 -19.994222 0.885899
O 14.650961 -18.757547 3.135571
H 14.233661 -16.465375 3.973955
H 13.712166 -14.015295 3.783356
H 13.483758 -12.98543 1.526024
H 13.774771 -14.374805 -0.517858

TS_para_anion_conf4

C 11.399232 -14.981893 -1.501845
C 13.605619 -14.916292 -1.001562
C 11.182591 -17.745167 -4.020612
C 11.20374 -18.819236 -3.162756
C 11.202414 -18.676936 -1.774608
C 11.219835 -17.448566 -1.17653
C 11.24152 -16.300702 -2.018818
C 11.232958 -16.446734 -3.459523
C 11.559561 -20.788824 -2.239221
H 11.160119 -17.858125 -5.099139
H 11.286647 -17.356218 -0.097466
H 11.01104 -21.732794 -2.157746
O 11.221815 -20.156785 -3.473546
O 11.177926 -19.927089 -1.188376
H 12.652507 -20.951845 -2.205154
N 11.308561 -15.321444 -4.244837
S 10.035455 -14.922799 -5.11228

O 9.303912 -16.058898 -5.707336
O 10.388049 -13.790265 -5.976696
C 9.328511 -14.540967 1.64788
C 9.643294 -13.228714 1.992098
C 9.891461 -15.134062 0.522852
H 9.201785 -12.7684 2.875138
H 9.592176 -16.140215 0.240468
C 10.510822 -12.501419 1.175053
C 10.79614 -14.431369 -0.292448
H 10.747215 -11.464959 1.41425
C 11.068142 -13.092444 0.05113
H 11.734064 -12.516317 -0.592875
H 8.623979 -15.10895 2.255134
C 8.161229 -15.140419 -3.083621
C 7.453966 -14.659763 -1.985101
C 8.938858 -14.267966 -3.844864
H 6.854508 -15.347238 -1.38479
C 7.508907 -13.312382 -1.619413
C 9.008521 -12.919417 -3.491075
H 9.630813 -12.255638 -4.089515
C 8.29593 -12.451043 -2.394392
H 8.357471 -11.39603 -2.118778
H 8.119756 -16.192514 -3.363026
C 6.750337 -12.798492 -0.432772
H 5.910088 -12.154068 -0.729635
H 6.337796 -13.619405 0.165994
H 7.392585 -12.201981 0.228087
H 11.562868 -14.247668 -2.293048
H 13.599418 -13.829655 -1.085434
C 13.597876 -15.500281 0.291864
C 13.903887 -16.818633 0.497243
C 14.321793 -17.689519 -0.583751
C 14.490005 -17.039267 -1.902214
C 14.177313 -15.669684 -2.089689
C 14.359835 -15.108612 -3.365558
C 14.842157 -15.869716 -4.415176
C 15.153169 -17.221928 -4.222579
C 14.972925 -17.796785 -2.976089

H 13.260085 -14.887773 1.130502
H 13.820626 -17.271701 1.484562
O 14.532357 -18.904794 -0.42471
H 15.192967 -18.846673 -2.787066
H 15.518882 -17.820061 -5.056761
H 14.949409 -15.419193 -5.40061
H 14.066432 -14.072739 -3.534868

TS_para_anion_conf5
C 11.205455 -15.705386 -2.005016
C 13.046447 -15.287151 -0.702075
C 10.447989 -12.219726 -3.160966
C 10.72238 -12.364786 -4.496698
C 11.21844 -13.548122 -5.041668
C 11.444099 -14.658643 -4.276966
C 11.153641 -14.580155 -2.88641
C 10.690772 -13.332621 -2.312484
C 10.852674 -12.12025 -6.701589
H 10.064968 -11.2966 -2.740718
H 11.771263 -15.585103 -4.735397
H 9.896114 -12.266645 -7.236582
O 10.607417 -11.420745 -5.489937
O 11.430575 -13.375017 -6.398188
H 11.546358 -11.537636 -7.32166
N 10.580988 -13.265937 -0.950568
S 9.226036 -12.7636 -0.260218
O 8.410389 -11.827915 -1.058593
O 9.503196 -12.433562 1.140755
C 12.550202 -18.921766 -3.506082
C 11.818918 -19.861127 -2.784368
C 12.363587 -17.561283 -3.289531
H 11.977543 -20.925563 -2.950547
H 12.995411 -16.850744 -3.81777
C 10.910109 -19.426666 -1.820126
C 11.430482 -17.09859 -2.340362
H 10.352365 -20.151046 -1.227098
C 10.733579 -18.069267 -1.592148
H 10.040548 -17.726995 -0.821472

H 13.300912 -19.248119 -4.224677
C 7.647103 -14.72947 -1.400416
C 7.122549 -16.014878 -1.468836
C 8.317144 -14.314405 -0.248638
H 6.608506 -16.340525 -2.374642
C 7.251105 -16.908211 -0.39892
C 8.442311 -15.183964 0.833467
H 8.967835 -14.840484 1.72291
C 7.905956 -16.465534 0.755756
H 8.004386 -17.14544 1.604038
H 7.55219 -14.037666 -2.236285
C 6.741268 -18.314124 -0.50737
H 5.769495 -18.360809 -1.014937
H 7.429974 -18.945188 -1.088046
H 6.628578 -18.782313 0.477687
H 10.600197 -15.557264 -1.11225
H 12.651266 -14.278298 -0.594932
C 12.802447 -16.225719 0.331206
C 13.430149 -17.441821 0.377686
C 14.444973 -17.81985 -0.584309
C 14.792131 -16.786915 -1.589309
C 14.114934 -15.539172 -1.621958
C 14.46436 -14.607672 -2.623624
C 15.443396 -14.903649 -3.556091
C 16.115465 -16.135535 -3.511412
C 15.78937 -17.060213 -2.533283
H 12.032467 -15.978358 1.06612
H 13.188107 -18.177336 1.143857
O 15.002621 -18.932122 -0.5767
H 16.2767 -18.032713 -2.471459
H 16.88805 -16.360223 -4.246787
H 15.693007 -14.174764 -4.327105
H 13.937536 -13.653206 -2.658488

Table S3. Computed energies for the anionic transition state conformational analysis in toluene.

	M06-L/BS1							TPSS0-D4/BS2
	1st frequency (cm-1)	Electronic energy	ZPE corr	Thermal corr	Enthalpy corr:	Gibbs corr	Electronic energy in acetonitrile	Electronic energy
pre-ortho TS								
TS_A-B_ortho_anion_toluene_conf2	-352.51	-2024.57129	0.47706	0.50873	0.50968	0.41141	-2024.63264	-2025.40274
TS_A-B_ortho_anion_toluene_conf3	-350.27	-2024.56947	0.47775	0.50895	0.50989	0.41362	-2024.63050	-2025.40580
TS_A-B_ortho_anion_toluene_conf4	-279.29	-2024.55913	0.47667	0.50831	0.50925	0.41114	-2024.62374	-2025.39226
TS_A-B_ortho_anion_toluene_conf5	-318.11	-2024.56553	0.47681	0.50821	0.50916	0.41386	-2024.62832	-2025.39693
TS_A-B_ortho_anion_toluene_conf6	-356.31	-2024.56966	0.47763	0.50896	0.50991	0.41242	-2024.63057	-2025.40448
TS_A-B_ortho_anion_toluene_conf7	-254.29	-2024.56474	0.47691	0.50858	0.50952	0.41058	-2024.62792	-2025.39784
TS_A-B_ortho_anion_toluene_conf8	-320.44	-2024.56555	0.47712	0.50847	0.50942	0.41418	-2024.62829	-2025.39694
TS_A-B_ortho_anion_toluene_conf9	-356.10	-2024.56967	0.47758	0.50892	0.50986	0.41259	-2024.63045	-2025.40456
TS_A-B_ortho_anion_toluene_conf10	-251.49	-2024.56466	0.47694	0.50862	0.50956	0.41069	-2024.62778	-2025.39759
TS_A-B_ortho_anion_toluene_conf11	-279.80	-2024.55912	0.47673	0.50835	0.50929	0.41155	-2024.62399	-2025.39228
TS_A-B_ortho_anion_toluene_conf12	-353.08	-2024.56942	0.47772	0.50894	0.50989	0.41333	-2024.63048	-2025.40578
TS_A-B_ortho_anion_toluene_conf13	-257.33	-2024.56475	0.47692	0.50858	0.50953	0.41038	-2024.62786	-2025.39788
TS_A-B_ortho_anion_toluene_conf14	-280.09	-2024.55911	0.47695	0.50848	0.50942	0.41223	-2024.62390	-2025.39227
TS_A-B_ortho_anion_toluene_conf15	-351.02	-2024.56943	0.47756	0.50887	0.50982	0.41247	-2024.63059	-2025.40584
TS_A-B_ortho_anion_toluene_conf16	-257.25	-2024.56474	0.47680	0.50851	0.50945	0.40981	-2024.62775	-2025.39788
TS_A-B_ortho_anion_toluene_conf17	-280.83	-2024.55915	0.47674	0.50834	0.50929	0.41153	-2024.62384	-2025.39229
TS_A-B_ortho_anion_toluene_conf18	-299.69	-2024.56462	0.47701	0.50875	0.50969	0.41210	-2024.62664	-2025.39553
TS_A-B_ortho_anion_toluene_conf19	-358.68	-2024.57017	0.47833	0.50908	0.51003	0.41676	-2024.63043	-2025.40797
TS_A-B_ortho_anion_toluene_conf20	-193.23	-2024.57312	0.47722	0.50862	0.50956	0.41352	-2024.63312	-2025.40632
pre-para TS								
TS_A-B_para_anion_toluene_conf2	-286.15	-2024.56244	0.47748	0.50869	0.50964	0.41377	-2024.62395	-2025.39953
TS_A-B_para_anion_toluene_conf3	-199.84	-2024.57113	0.47715	0.50872	0.50967	0.41201	-2024.63179	-2025.40575
TS_A-B_para_anion_toluene_conf4	-300.13	-2024.56461	0.47687	0.50869	0.50964	0.41130	-2024.62676	-2025.39555
TS_A-B_para_anion_toluene_conf5	-198.89	-2024.57316	0.47724	0.50869	0.50963	0.41320	-2024.63315	-2025.40642
TS_A-B_para_anion_toluene_conf6	-359.29	-2024.57021	0.47795	0.50895	0.50989	0.41559	-2024.63030	-2025.40803
TS_A-B_para_anion_toluene_conf7	-163.70	-2024.55160	0.47609	0.50802	0.50896	0.40982	-2024.61451	-2025.38320
TS_A-B_para_anion_toluene_conf8	-201.51	-2024.57111	0.47713	0.50871	0.50965	0.41215	-2024.63180	-2025.40580
TS_A-B_para_anion_toluene_conf9	-153.17	-2024.55164	0.47615	0.50792	0.50887	0.41114	-2024.61467	-2025.38293
TS_A-B_para_anion_toluene_conf10	-29.94	-2024.56359	0.47689	0.50862	0.50956	0.40986	-2024.62566	-2025.39734
TS_A-B_para_anion_toluene_conf11	-9.32	-2024.56360	0.47690	0.50860	0.50954	0.41037	-2024.62568	-2025.39686
TS_A-B_para_anion_toluene_conf12	-364.58	-2024.56359	0.47818	0.50940	0.51035	0.41305	-2024.62553	-2025.40172

TS_A-B_para_anion_toluene_conf13	-61.00	-2024.56310	0.47707	0.50882	0.50976	0.40917	-2024.62538	-2025.39828
TS_A-B_para_anion_toluene_conf14	-107.90	-2024.56314	0.47706	0.50884	0.50978	0.40979	-2024.62537	-2025.39896
TS_A-B_para_anion_toluene_conf15	-375.02	-2024.56002	0.47766	0.50897	0.50991	0.41379	-2024.62075	-2025.39609

Table S4. Computed energies for the anionic path in toluene.

	M06-L/BS1							TPSS0-D4/BS2
	1st frequency (cm-1)	Electronic energy	ZPE corr	Thermal corr	Enthalpy corr:	Gibbs corr	Electronic energy in acetonitrile	Electronic energy
Path for ortho product								
interA_pre-ortho_anion_toluene	15.75	-2024.57632	0.477041	0.509238	0.510182	0.411611	-2024.638348	**-2025.40522**
TS_A-B_ortho_anion_toluene	-351.10	-2024.56944	0.477435	0.508787	0.509731	0.411968	-2024.630536	-2025.40579
interB_ortho_anion_toluene	11.55	-2024.58623	0.479786	0.511268	0.512212	0.413741	-2024.647246	-2025.42548
Path for para product								
interA_pre-para_anion_toluene	11.94	-2024.57176	0.476957	0.509391	0.510335	0.409747	-2024.632616	-2025.40203
TS_A-B_para_anion_toluene	-207.38	-2024.57109	0.476957	0.508647	0.509592	0.41049	-2024.631679	-2025.40580
interB_para_anion_toluene	17.52	-2024.59178	0.480125	0.511545	0.512489	0.41503	-2024.651954	-2025.43325

interB_ortho_anion_toluene

C 12.3603987699 -15.0886752957 -1.8222373413
C 12.7644370027 -16.5786216879 -1.4468310734
C 14.2357138654 -17.5347583435 -3.2088755509
C 12.738123174 -14.772667461 -5.6126802885
C 11.471184652 -15.1584466424 -5.9763548894
C 10.4694618676 -15.4187367848 -5.0536657404
C 10.7083155311 -15.3220335954 -3.703902855
C 12.0060416975 -14.9785244837 -3.2872293978
C 13.0346609031 -14.6717847201 -4.2243147471
C 9.7221130128 -15.9720630502 -7.0495346649
H 13.5020714728 -14.58241897 -6.3599071201
H 9.9365636531 -15.5540887752 -2.969859618
H 8.977671493 -15.5710563503 -7.7481597815
O 10.9601684258 -15.3099829322 -7.2536056912
O 9.2948474071 -15.7391445678 -5.7214189238
H 9.8593497434 -17.0598700075 -7.2107436104
N 14.2485622433 -14.2748995589 -3.7032080061

S 15.6116519786 -14.5025140641 -4.4873953885
O 15.6246492122 -15.6498293406 -5.4238268197
O 16.7060718849 -14.4089818084 -3.5091482212
C 9.8220170081 -14.3309198066 0.9755496196
C 9.5760348019 -12.9834249619 0.7405102005
C 10.7002883102 -15.0484647646 0.1614144067
H 8.8895357518 -12.4271482388 1.3784052878
H 10.8468465476 -16.1109331696 0.3374147371
C 10.2142332646 -12.3534209119 -0.3286804956
C 11.3481284347 -14.4284483779 -0.9100413667
H 10.0329907459 -11.2976316577 -0.5297182702
C 11.0826494659 -13.0691280904 -1.1413466735
H 11.5782318422 -12.5798287283 -1.9807134471
H 9.3218590618 -14.8412646871 1.798771625
C 15.8270433115 -13.1674567624 -6.9259290673
C 15.9555382785 -12.0273222144 -7.7180799596
C 15.8238440597 -13.0531824266 -5.5397327676
H 15.9593643668 -12.1213714466 -8.8056938266
C 16.0825091703 -10.7611560665 -7.1456834074
C 15.9536760407 -11.7935727764 -4.9501419044
H 15.9547918589 -11.7170818779 -3.8639940497
C 16.08244156 -10.6650045128 -5.7464482323
H 16.186471056 -9.6829758463 -5.2807645406
H 15.7388008613 -14.1589965736 -7.3672130019
C 16.2055979286 -9.5321734355 -7.9966022941
H 16.3085993923 -9.7859089155 -9.0582430409
H 15.3261438691 -8.879869936 -7.901537758
H 17.0758503675 -8.9240196439 -7.714976394
H 13.3006343815 -14.5281117092 -1.7075407062
H 15.1984937934 -17.5777899508 -3.7140732957
C 14.0981220237 -16.8350956479 -2.0720310591
C 11.6988605879 -17.582965977 -1.8621754674
C 11.857155213 -18.2226553429 -3.1835768197
C 13.0976866731 -18.1511109231 -3.8582768801
C 13.1903936433 -18.7009605703 -5.1457994145
C 12.0911206052 -19.3108651476 -5.7353348039
C 10.8761675054 -19.4086633798 -5.0475930505
C 10.7676423901 -18.8656767093 -3.775113141

H 14.9536191587 -16.3033104263 -1.6570130422
H 12.8388344911 -16.6123923556 -0.3496552595
O 10.7270886807 -17.8252208029 -1.1473855002
H 9.8319652478 -18.8949242737 -3.2184570963
H 10.0175078271 -19.8873879068 -5.5169215587
H 12.1770897417 -19.7155715546 -6.7439133007
H 14.1330696901 -18.6102050535 -5.6847572541

TS_A-B_ortho_anion_toluene
C 12.389104 -14.757886 -2.084501
C 13.246031 -16.603913 -1.376059
C 13.993413 -17.507389 -3.49864
C 13.283589 -15.013388 -5.732177
C 12.061867 -15.378304 -6.226014
C 10.91141 -15.43816 -5.438053
C 10.951576 -15.189856 -4.098169
C 12.201092 -14.870049 -3.504897
C 13.381083 -14.707961 -4.34008
C 10.413068 -16.210677 -7.437366
H 14.160245 -14.982474 -6.369264
H 10.048589 -15.25397 -3.50205
H 9.828422 -15.8587 -8.294984
O 11.733872 -15.689056 -7.52546
O 9.825462 -15.745097 -6.241871
H 10.466045 -17.316484 -7.421418
N 14.497957 -14.247293 -3.716172
S 15.94627 -14.257136 -4.407806
O 16.236424 -15.442738 -5.240058
O 16.912972 -13.894758 -3.366047
C 9.159041 -14.6785 -0.051219
C 9.465597 -13.678905 0.867231
C 10.085483 -15.069295 -1.011974
H 8.736041 -13.378921 1.618807
H 9.838294 -15.894227 -1.673406
C 10.724527 -13.081474 0.828226
C 11.34813 -14.45399 -1.094257
H 10.987411 -12.307444 1.548843
C 11.648794 -13.472765 -0.130844

H 12.632246 -13.002731 -0.160914
H 8.192376 -15.178955 -0.010448
C 16.081933 -13.054355 -6.916067
C 16.050932 -11.957988 -7.776352
C 15.933955 -12.86294 -5.547096
H 16.168629 -12.109916 -8.850615
C 15.871266 -10.663014 -7.288583
C 15.757661 -11.574096 -5.039849
H 15.647352 -11.438716 -3.965346
C 15.727394 -10.489803 -5.904554
H 15.589849 -9.483399 -5.505221
H 16.231805 -14.065484 -7.290904
C 15.82502 -9.484182 -8.214479
H 16.028578 -9.777492 -9.250868
H 14.841965 -8.993797 -8.200102
H 16.560107 -8.716925 -7.936599
H 13.332628 -14.262476 -1.8569
H 14.747445 -17.587412 -4.280839
C 14.277332 -16.858703 -2.330935
C 12.041031 -17.430067 -1.389107
C 11.714528 -18.027198 -2.697197
C 12.681893 -18.032807 -3.740208
C 12.306514 -18.568549 -4.988859
C 11.030578 -19.069633 -5.196171
C 10.085945 -19.067375 -4.160789
C 10.44072 -18.557843 -2.920075
H 15.262033 -16.420047 -2.179654
H 13.516929 -16.212698 -0.394746
O 11.298702 -17.5558 -0.400482
H 9.739578 -18.539598 -2.085129
H 9.083209 -19.456494 -4.333558
H 10.762867 -19.474796 -6.173376
H 13.036188 -18.566614 -5.800288

InterA_pre-ortho_anion_toluene

C 12.2865408226 -14.4135315753 -2.108864847
C 13.5013575706 -16.9302213721 -1.2793612646
C 13.955917559 -17.4751971575 -3.5994724631

C 13.4210360786 -15.022698637 -5.6277809128
C 12.2167414811 -15.3600261051 -6.1674143399
C 11.0055220871 -15.298956238 -5.4527366359
C 10.9638843811 -14.9532647496 -4.1434696858
C 12.1986067833 -14.6571661517 -3.4772889959
C 13.4518498805 -14.6031579697 -4.2580026522
C 10.5878189063 -16.1292239579 -7.4581689902
H 14.3339542827 -15.0819269671 -6.2090012863
H 10.0215705451 -14.9001653758 -3.6103456762
H 10.0999027667 -15.7342855985 -8.3568913271
O 11.9547811405 -15.7342744989 -7.458496494
O 9.9663865244 -15.5920948068 -6.3098354436
H 10.5350644543 -17.2319024842 -7.4290918287
N 14.5260980682 -14.109070345 -3.6179631403
S 16.0074510575 -14.1157259871 -4.2721313566
O 16.3596517839 -15.3578491466 -4.9852613919
O 16.91950831 -13.6248849494 -3.2376250354
C 9.1290010628 -14.9718898011 -0.0842959783
C 9.355606016 -14.0992691422 0.9755229147
C 10.0673542089 -15.1165578108 -1.0983286884
H 8.6176247159 -13.9945531661 1.7701567876
H 9.901825866 -15.8610969436 -1.8717899898
C 10.5558441352 -13.3871353631 1.0302824223
C 11.2638678401 -14.3687966032 -1.0925737292
H 10.7592824303 -12.7203192545 1.8678315679
C 11.4951274325 -13.5327391805 0.0226826589
H 12.4337006681 -12.9796391606 0.0673294406
H 8.2229707464 -15.5761302415 -0.109520372
C 16.1843115076 -13.128670355 -6.8640352626
C 16.1340791275 -12.1149510612 -7.8189752741
C 15.9565021646 -12.8273290274 -5.52647716
H 16.3142328328 -12.3517493126 -8.86872103
C 15.8545776653 -10.7963909261 -7.4570218514
C 15.68218087 -11.5135833403 -5.1426155519
H 15.5114561483 -11.2916601456 -4.090648193
C 15.6313432497 -10.5128398287 -6.1022321649
H 15.4153840888 -9.4864101153 -5.8013056052
H 16.4086736187 -14.1577507393 -7.1396140292

C 15.7863622586 -9.7073047618 -8.4856029959
H 16.0612202398 -10.0747628532 -9.4809188733
H 14.7761712421 -9.2822750856 -8.5604980599
H 16.4583416732 -8.8736479382 -8.2421309576
H 13.2690008468 -14.0724994291 -1.7851640689
H 14.6416624238 -17.4783084168 -4.4455434246
C 14.3702790097 -16.9957750094 -2.3665613213
C 12.1656455251 -17.4631811187 -1.3313695739
C 11.7298045084 -17.936895126 -2.6581549391
C 12.6239881903 -17.9347953251 -3.7713945255
C 12.1336557851 -18.382918358 -5.0213226114
C 10.8233355319 -18.8060239144 -5.1662564494
C 9.9448598044 -18.7872239612 -4.0720775917
C 10.4081070386 -18.3611939524 -2.8360845526
H 15.384467596 -16.608123288 -2.267026172
H 13.8334416717 -16.5362356534 -0.3189386351
O 11.4003095024 -17.5030909458 -0.3388991711
H 9.7648518626 -18.3431257755 -1.955180226
H 8.9118966101 -19.1121346068 -4.1948526369
H 10.4725176878 -19.1603743448 -6.1375020061
H 12.8124831125 -18.387737544 -5.8769573518

interB_para_anion_toluene

C 11.418504233 -15.7132709032 -2.039539292
C 12.639029589 -16.6840094008 -1.7053911219
C 9.6141531942 -15.2805301713 1.2963020592
C 9.702984805 -13.9160294641 1.437555745
C 10.3467960736 -13.1146520609 0.5090702547
C 10.9318598341 -13.6432776005 -0.6193605924
C 10.8549539472 -15.0339944624 -0.8121796719
C 10.1977623647 -15.8663695898 0.1426409635
C 9.395477345 -11.8005382231 2.0083051195
H 9.0990302461 -15.9110175444 2.0130783753
H 11.4129943233 -12.9963249161 -1.3476913523
H 8.4149892802 -11.4252654651 1.6539534796
O 9.2478257096 -13.128706119 2.4807846779
O 10.321935833 -11.7952625575 0.9373791647
H 9.7727110844 -11.1655296021 2.8195257855

N 10.2154741702 -17.2395501075 -0.064650194
S 8.8304857862 -18.0184254651 -0.154648714
O 7.7818248151 -17.5628776562 0.7813900838
O 9.1050748781 -19.4578021768 -0.2405671769
C 13.0124350927 -13.1203741078 -4.3752090958
C 12.2655634526 -13.3069066908 -5.5354784066
C 12.7430192669 -13.8720685668 -3.2343048082
H 12.4809512104 -12.7204583833 -6.4280183008
H 13.3419245228 -13.71286981 -2.3387075686
C 11.2458028961 -14.2548644098 -5.5441701514
C 11.7189254108 -14.8299535908 -3.2262014221
H 10.6570317689 -14.4188804455 -6.4468754793
C 10.9813647833 -15.0064568606 -4.4028184535
H 10.190135806 -15.757878371 -4.4151375927
H 13.8175492744 -12.3868022568 -4.355891132
C 7.6841273606 -16.2531571321 -1.9549233018
C 7.3997460681 -15.7902448933 -3.2336091726
C 8.2367179651 -17.5255462284 -1.7813291337
H 6.9869840301 -14.7884366813 -3.3652591766
C 7.6449326163 -16.5813766164 -4.3628257182
C 8.4842302294 -18.3274656302 -2.8938112374
H 8.9182320069 -19.3138994669 -2.7376768207
C 8.1837967122 -17.8576243884 -4.1698089837
H 8.3791117568 -18.4879268778 -5.039713609
H 7.4911024425 -15.6337968759 -1.0800166189
C 7.3556431874 -16.0593149819 -5.7389237137
H 6.2769590133 -15.9601216284 -5.9260204428
H 7.7927827181 -15.0629910802 -5.8906540629
H 7.7611103128 -16.7205928065 -6.5141832462
H 10.6411936581 -16.4251964452 -2.3546241149
H 12.1981194636 -17.3121175805 -0.9095562123
C 12.8670997897 -17.5340893244 -2.8968137751
C 14.0117171375 -17.5696650063 -3.602088406
C 15.1760452563 -16.7723518128 -3.2261799368
C 15.0625670252 -16.004183017 -1.9607657122
C 13.8726577303 -16.0023412153 -1.2058100201
C 13.8669636348 -15.3323661996 0.0271665776
C 14.9881711258 -14.6402617495 0.4658706405

C 16.1523986729 -14.6131449222 -0.3076586856
C 16.1879413051 -15.3056381669 -1.5065070333
H 12.0019737644 -18.1179635091 -3.2239996433
H 14.1171178011 -18.1839795853 -4.4952029986
O 16.2075577463 -16.7595917887 -3.9040167496
H 17.0806737227 -15.3370696684 -2.1289995197
H 17.0286920862 -14.0666728431 0.0397643106
H 14.9565542829 -14.1205640312 1.4229023169
H 12.9678383807 -15.3558268677 0.6400190177

TS_A-B_para_anion_toluene
C 11.116725 -15.520712 -1.975655
C 12.824938 -16.959113 -1.466451
C 9.45598 -15.289143 1.378442
C 9.717114 -13.97368 1.664715
C 10.484029 -13.158119 0.834173
C 11.010787 -13.613893 -0.342201
C 10.752358 -14.963812 -0.709194
C 10.003092 -15.825772 0.182538
C 9.712872 -11.91631 2.504638
H 8.862757 -15.921306 2.029343
H 11.550793 -12.94199 -0.999718
H 8.810187 -11.324895 2.263621
O 9.333264 -13.258205 2.774673
O 10.603412 -11.903567 1.405919
H 10.213686 -11.497566 3.387212
N 9.924348 -17.152525 -0.140384
S 8.511463 -17.904312 -0.196418
O 7.454825 -17.346959 0.670183
O 8.744652 -19.351315 -0.175841
C 13.189129 -13.278239 -4.230582
C 12.745773 -13.700042 -5.48103
C 12.668739 -13.838095 -3.069392
H 13.165714 -13.266127 -6.387215
H 13.081447 -13.53962 -2.10834
C 11.787349 -14.709579 -5.562338
C 11.678955 -14.839454 -3.126531
H 11.452618 -15.070195 -6.534627

C 11.277012 -15.279115 -4.404361
H 10.544287 -16.085795 -4.465274
H 13.974787 -12.527717 -4.153729
C 7.434057 -16.256396 -2.142295
C 7.253998 -15.843223 -3.457377
C 8.032397 -17.489209 -1.878485
H 6.794912 -14.874325 -3.660519
C 7.661711 -16.643388 -4.530866
C 8.4314 -18.307423 -2.934169
H 8.895238 -19.265915 -2.707424
C 8.239076 -17.885948 -4.24608
H 8.551777 -18.527797 -5.071783
H 7.122347 -15.629669 -1.307868
C 7.525248 -16.160606 -5.943882
H 6.577977 -15.631531 -6.10766
H 8.32834 -15.455745 -6.204671
H 7.57696 -16.987277 -6.662352
H 10.519331 -16.395384 -2.227655
H 12.192607 -17.310442 -0.652731
C 12.854569 -17.70221 -2.67282
C 13.768514 -17.454323 -3.661673
C 14.817175 -16.466641 -3.508911
C 14.867394 -15.782991 -2.194473
C 13.897436 -16.049957 -1.192298
C 13.974457 -15.342533 0.026936
C 14.97166 -14.406961 0.241591
C 15.933333 -14.155876 -0.749999
C 15.876332 -14.843127 -1.951007
H 12.068276 -18.445208 -2.827295
H 13.74065 -17.991514 -4.608862
O 15.63324 -16.20268 -4.41022
H 16.59456 -14.668551 -2.751522
H 16.718161 -13.420608 -0.572327
H 15.009446 -13.864917 1.18648
H 13.223659 -15.536918 0.793932

interA_pre-para_anion_toluene

C 11.0059747719 -15.3988724098 -2.0021683376

C 12.9040092543 -17.0750764674 -1.4697413465
C 9.4373248674 -15.2723228261 1.3878103726
C 9.7368194616 -13.9766376666 1.7166791121
C 10.5166320073 -13.1473124132 0.9058688628
C 11.0141240567 -13.5655596242 -0.2933972331
C 10.7151321517 -14.8971835093 -0.7129726797
C 9.9630305811 -15.7807754483 0.1689888205
C 9.827530694 -11.960126171 2.6568204756
H 8.8402250777 -15.9128603093 2.0266423065
H 11.5561292163 -12.8792724764 -0.9334873557
H 8.9521722907 -11.3046406771 2.501120087
O 9.38099565 -13.294797316 2.8532631052
O 10.6677262344 -11.9186080103 1.5202219775
H 10.389819317 -11.633328019 3.542084482
N 9.8674640433 -17.0878793185 -0.1943423126
S 8.4598990113 -17.8619574648 -0.2018448016
O 7.4112204836 -17.2943908889 0.6667515455
O 8.7115968698 -19.3043753303 -0.1506221519
C 13.2862946823 -13.3035843909 -4.1800253363
C 12.9373986001 -13.7854468491 -5.4396440767
C 12.6713146739 -13.7962954822 -3.0374812661
H 13.439630525 -13.4088169033 -6.3290309877
H 13.029326799 -13.4687750839 -2.0642261192
C 11.9737554477 -14.7880213203 -5.5464412503
C 11.6685056633 -14.7883683312 -3.1199094363
H 11.7178543083 -15.199976652 -6.5221292783
C 11.3678029678 -15.2928344867 -4.4063658763
H 10.6344052458 -16.0976537556 -4.483235982
H 14.0833930263 -12.5682014237 -4.0796344797
C 7.3158795611 -16.2831737734 -2.1655181159
C 7.1356653582 -15.8855142504 -3.4861984552
C 7.9707461582 -17.4822837179 -1.8880156127
H 6.6314788114 -14.9421339942 -3.701677436
C 7.6022164851 -16.6667920281 -4.5482681733
C 8.4242639206 -18.2867398582 -2.9330047271
H 8.9314942531 -19.2200495898 -2.6949161582
C 8.2335384789 -17.8803487597 -4.2487061538
H 8.5917344042 -18.5091555022 -5.0657804283

H 6.9635799129 -15.6686234812 -1.3383637673
C 7.4743803859 -16.1965733126 -5.9661636766
H 6.59345129 -15.5588117354 -6.1075046555
H 8.3487984962 -15.6019035864 -6.2692094557
H 7.4005584065 -17.0348437769 -6.6695350148
H 10.4563171972 -16.3072896023 -2.2423621445
H 12.2317646158 -17.3813370435 -0.6702366105
C 12.8722458875 -17.7414870801 -2.703685827
C 13.7261645153 -17.4116782591 -3.7338335026
C 14.7377944813 -16.3907384223 -3.5954024705
C 14.8284523098 -15.7607623968 -2.2589680752
C 13.9107520753 -16.1013270753 -1.2247057329
C 13.9863612345 -15.3984525658 0.0008656566
C 14.9412009575 -14.4188658038 0.2000597346
C 15.8667000518 -14.1139410052 -0.8136069582
C 15.8020845837 -14.7810190842 -2.0247751496
H 12.1029032077 -18.502146354 -2.8566752031
H 13.6588883084 -17.9028006448 -4.7040964062
O 15.4868582984 -16.0435502484 -4.5323199976
H 16.4803670782 -14.5533656167 -2.84685418
H 16.6236468503 -13.3476290342 -0.6454597113
H 14.9766487301 -13.8830489303 1.1488296039
H 13.2648847154 -15.6361284413 0.7844279651

TS_A-B_ortho_anion_toluene

C 12.389104 -14.757886 -2.084501
C 13.246031 -16.603913 -1.376059
C 13.993413 -17.507389 -3.49864
C 13.283589 -15.013388 -5.732177
C 12.061867 -15.378304 -6.226014
C 10.91141 -15.43816 -5.438053
C 10.951576 -15.189856 -4.098169
C 12.201092 -14.870049 -3.504897
C 13.381083 -14.707961 -4.34008
C 10.413068 -16.210677 -7.437366
H 14.160245 -14.982474 -6.369264
H 10.048589 -15.25397 -3.50205
H 9.828422 -15.8587 -8.294984

O 11.733872 -15.689056 -7.52546
O 9.825462 -15.745097 -6.241871
H 10.466045 -17.316484 -7.421418
N 14.497957 -14.247293 -3.716172
S 15.94627 -14.257136 -4.407806
O 16.236424 -15.442738 -5.240058
O 16.912972 -13.894758 -3.366047
C 9.159041 -14.6785 -0.051219
C 9.465597 -13.678905 0.867231
C 10.085483 -15.069295 -1.011974
H 8.736041 -13.378921 1.618807
H 9.838294 -15.894227 -1.673406
C 10.724527 -13.081474 0.828226
C 11.34813 -14.45399 -1.094257
H 10.987411 -12.307444 1.548843
C 11.648794 -13.472765 -0.130844
H 12.632246 -13.002731 -0.160914
H 8.192376 -15.178955 -0.010448
C 16.081933 -13.054355 -6.916067
C 16.050932 -11.957988 -7.776352
C 15.933955 -12.86294 -5.547096
H 16.168629 -12.109916 -8.850615
C 15.871266 -10.663014 -7.288583
C 15.757661 -11.574096 -5.039849
H 15.647352 -11.438716 -3.965346
C 15.727394 -10.489803 -5.904554
H 15.589849 -9.483399 -5.505221
H 16.231805 -14.065484 -7.290904
C 15.82502 -9.484182 -8.214479
H 16.028578 -9.777492 -9.250868
H 14.841965 -8.993797 -8.200102
H 16.560107 -8.716925 -7.936599
H 13.332628 -14.262476 -1.8569
H 14.747445 -17.587412 -4.280839
C 14.277332 -16.858703 -2.330935
C 12.041031 -17.430067 -1.389107
C 11.714528 -18.027198 -2.697197
C 12.681893 -18.032807 -3.740208

C 12.306514 -18.568549 -4.988859
C 11.030578 -19.069633 -5.196171
C 10.085945 -19.067375 -4.160789
C 10.44072 -18.557843 -2.920075
H 15.262033 -16.420047 -2.179654
H 13.516929 -16.212698 -0.394746
O 11.298702 -17.5558 -0.400482
H 9.739578 -18.539598 -2.085129
H 9.083209 -19.456494 -4.333558
H 10.762867 -19.474796 -6.173376
H 13.036188 -18.566614 -5.800288

TS_A-B_ortho_anion_toluene_conf2
C 12.276099 -14.499929 -1.074385
C 13.273833 -16.21937 -1.922243
C 13.002339 -16.231502 -4.348787
C 15.328415 -12.584258 0.055661
C 15.182026 -11.47946 -0.733724
C 14.113143 -11.307176 -1.617356
C 13.144075 -12.256356 -1.760878
C 13.235315 -13.443182 -0.981658
C 14.336735 -13.612138 -0.04268
C 15.307705 -9.436457 -1.60152
H 16.149157 -12.676761 0.758896
H 12.297776 -12.08793 -2.418327
H 14.909383 -8.637195 -0.949309
O 16.006042 -10.381872 -0.804473
O 14.245703 -10.089983 -2.266359
H 15.996664 -9.009842 -2.342283
N 14.335083 -14.750191 0.6782
S 15.585299 -15.153034 1.624216
O 15.947289 -14.144901 2.642063
O 16.704016 -15.703649 0.836531
C 9.759012 -14.085447 -3.892184
C 8.619127 -14.620342 -3.299634
C 10.953372 -14.006385 -3.183016
H 7.688952 -14.690864 -3.862265
H 11.848717 -13.647035 -3.68302

C 8.685392 -15.087044 -1.98707
C 11.036881 -14.462934 -1.855128
H 7.805997 -15.527743 -1.5186
C 9.877132 -15.015357 -1.279694
H 9.942819 -15.420987 -0.270878
H 9.727791 -13.746735 -4.927527
C 15.28936 -16.762515 3.806575
C 14.787466 -17.851814 4.512893
C 14.833149 -16.508915 2.515995
H 15.14111 -18.048031 5.526705
C 13.82671 -18.695795 3.950019
C 13.874584 -17.337174 1.932371
H 13.481809 -17.132131 0.937581
C 13.377891 -18.415536 2.653949
H 12.616613 -19.045917 2.191975
H 16.020233 -16.090109 4.251721
C 13.282002 -19.866472 4.71351
H 13.718922 -19.933711 5.717024
H 12.191706 -19.804543 4.830616
H 13.485383 -20.816386 4.200755
H 12.202428 -15.108143 -0.169895
H 13.313494 -15.903294 -5.341487
C 13.689225 -15.84561 -3.233308
C 12.190631 -17.17417 -1.745259
C 11.454283 -17.562224 -2.962513
C 11.860178 -17.092507 -4.240484
C 11.110507 -17.486501 -5.366258
C 10.001543 -18.305199 -5.236656
C 9.605323 -18.76352 -3.972213
C 10.332367 -18.39207 -2.853164
H 14.556571 -15.190002 -3.327964
H 13.989475 -16.144951 -1.101023
O 11.843263 -17.590779 -0.620063
H 10.055032 -18.718138 -1.851362
H 8.727739 -19.401922 -3.873411
H 9.434791 -18.593404 -6.122462
H 11.415577 -17.127918 -6.351066

TS_A-B_ortho_anion_toluene_conf3

C 0.960988 0.084629 0.750289
C 1.511099 -0.190954 -1.314772
C 3.412973 1.311021 -1.40013
C 3.406478 2.788477 1.689172
C 4.304265 2.03025 2.388349
C 4.09808 0.679568 2.673704
C 3.000995 0.013309 2.214258
C 2.059223 0.722546 1.423358
C 2.221442 2.151966 1.208458
C 6.12243 1.219248 3.342468
H 3.598586 3.83446 1.478283
H 2.865975 -1.038828 2.437151
H 6.626332 1.363562 4.304841
O 5.489638 2.434268 2.958354
O 5.134171 0.220629 3.471345
H 6.845611 0.929382 2.555615
N 1.198588 2.780681 0.570319
S 1.313562 4.279195 0.00723
O 2.627815 4.636734 -0.564847
O 0.120357 4.51399 -0.81283
C 0.346111 -3.53419 1.797836
C -1.043001 -3.538394 1.882448
C 1.031359 -2.385695 1.416669
H -1.573897 -4.442197 2.179754
H 2.110287 -2.432316 1.301639
C -1.747959 -2.379433 1.561351
C 0.343075 -1.193714 1.125026
H -2.836646 -2.368446 1.60773
C -1.062813 -1.233672 1.180402
H -1.616152 -0.327472 0.932124
H 0.90829 -4.442392 2.011642
C 2.129902 6.193044 1.85857
C 1.952905 6.988197 2.98977
C 1.113215 5.337614 1.449574
H 2.751182 7.660779 3.308222
C 0.768981 6.941025 3.726887
C -0.080658 5.280399 2.171247

H -0.870139 4.609763 1.836423
C -0.246194 6.074897 3.296249
H -1.180794 6.029125 3.858068
H 3.047779 6.233493 1.27443
C 0.585009 7.783351 4.953977
H -0.349414 8.359031 4.918548
H 1.40885 8.49515 5.081585
H 0.539576 7.17044 5.864865
H 0.22525 0.820356 0.426683
H 3.826147 2.308605 -1.545029
C 2.094962 1.06606 -1.66068
C 2.37439 -1.359057 -1.158735
C 3.764275 -1.052709 -0.772735
C 4.2554 0.278793 -0.871354
C 5.574217 0.531563 -0.442452
C 6.366426 -0.485811 0.067016
C 5.876824 -1.796072 0.154692
C 4.586673 -2.068329 -0.277054
H 1.453109 1.870488 -2.015069
H 0.499607 -0.416787 -1.654192
O 1.956878 -2.525098 -1.257674
H 4.165681 -3.073278 -0.231677
H 6.502997 -2.588395 0.563174
H 7.382355 -0.263618 0.397539
H 5.958156 1.551363 -0.502076

TS_A-B_ortho_anion_toluene_conf4
C 1.195382 0.916018 0.860911
C 1.470505 -0.149975 -1.10925
C 3.373549 0.829771 -2.260922
C 2.022419 -1.868032 3.249162
C 0.752355 -2.230053 3.589166
C -0.387048 -1.570977 3.114705
C -0.299519 -0.529409 2.242527
C 0.992513 -0.111262 1.814959
C 2.185566 -0.76673 2.351727
C -1.045195 -3.300554 4.329006
H 2.886505 -2.417596 3.604089

H -1.191844 -0.00658 1.914514
H -1.496262 -3.394244 5.324956
O 0.365766 -3.22221 4.46055
O -1.511286 -2.117844 3.711336
H -1.30371 -4.177334 3.70692
N 3.365183 -0.238972 1.961791
S 4.787389 -0.718242 2.566727
O 4.966772 -2.174662 2.727729
O 5.821101 0.033836 1.854378
C -1.96945 2.564275 -0.473329
C -1.562005 3.895888 -0.53542
C -1.101081 1.578687 -0.018495
H -2.24426 4.665358 -0.894537
H -1.423293 0.539192 -0.031472
C -0.263152 4.224511 -0.15281
C 0.205625 1.892337 0.401245
H 0.079633 5.257291 -0.212518
C 0.604604 3.237614 0.299364
H 1.623622 3.497251 0.585905
H -2.972069 2.285946 -0.797359
C 4.676564 -0.889331 5.344915
C 4.632973 -0.342234 6.626384
C 4.763012 -0.050732 4.239556
H 4.568231 -1.001398 7.493758
C 4.671462 1.038662 6.823307
C 4.807502 1.333309 4.416396
H 4.884974 1.974918 3.540386
C 4.763114 1.867441 5.696037
H 4.801831 2.949691 5.832379
H 4.664764 -1.966396 5.189269
C 4.610118 1.627932 8.201123
H 5.43975 2.323419 8.385807
H 4.650058 0.851476 8.973913
H 3.684179 2.198166 8.358732
H 2.220512 1.282712 0.837946
H 3.807573 1.612603 -2.885061
C 2.037622 0.83047 -1.969322
C 2.254497 -1.289756 -0.667127

C 3.69543 -1.253566 -0.980792
C 4.234788 -0.207103 -1.774984
C 5.611899 -0.235846 -2.075363
C 6.421868 -1.252867 -1.604784
C 5.880379 -2.280395 -0.819293
C 4.52921 -2.27951 -0.522584
H 1.393322 1.621699 -2.358827
H 0.393198 -0.306402 -1.112157
O 1.733001 -2.231961 -0.040273
H 4.08117 -3.045122 0.108171
H 6.526342 -3.06061 -0.418878
H 7.487548 -1.2479 -1.834933
H 6.033388 0.567949 -2.682214

TS_A-B_ortho_anion_toluene_conf5
C -1.572754 1.418246 -0.662342
C -0.792975 1.950277 1.350505
C 1.459546 2.861808 1.190468
C -1.665702 -2.336669 -0.43935
C -2.998052 -2.495457 -0.204654
C -3.900998 -1.425074 -0.149057
C -3.495607 -0.136086 -0.307147
C -2.111997 0.114669 -0.532166
C -1.17596 -1.004163 -0.623493
C -4.999681 -3.284847 0.340283
H -0.986454 -3.181956 -0.469961
H -4.216686 0.674655 -0.304529
H -5.730318 -3.890631 -0.210527
O -3.689351 -3.672521 -0.041046
O -5.18285 -1.920454 0.018983
H -5.120902 -3.434371 1.428964
N 0.105829 -0.690912 -0.886385
S 1.183753 -1.838619 -1.254269
O 0.985808 -2.352752 -2.623424
O 1.404401 -2.863256 -0.213971
C -4.100959 4.264153 -0.49765
C -3.619929 5.097645 -1.505886
C -3.462029 3.064289 -0.205839

H -4.12195 6.037869 -1.730339
H -3.824863 2.454731 0.619757
C -2.476811 4.721003 -2.207765
C -2.323872 2.650065 -0.922362
H -2.075953 5.367781 -2.987864
C -1.838825 3.521965 -1.913507
H -0.939981 3.235171 -2.459901
H -4.977432 4.557588 0.079645
C 3.878946 -1.481687 -1.040188
C 5.069222 -0.771431 -1.132075
C 2.667155 -0.838829 -1.285569
H 6.015825 -1.274064 -0.924978
C 5.074262 0.588939 -1.455069
C 2.65271 0.510141 -1.630263
H 1.699476 1.004309 -1.800954
C 3.850182 1.212509 -1.709249
H 3.83247 2.277717 -1.947715
H 3.869148 -2.531513 -0.752736
C 6.35261 1.371887 -1.459585
H 7.209189 0.757887 -1.764467
H 6.300783 2.236689 -2.132184
H 6.58284 1.762176 -0.456643
H -0.574552 1.42266 -1.096124
H 2.137491 3.691252 0.980564
C 0.104247 3.033851 1.129484
C -0.295376 0.66251 1.80736
C 1.170378 0.499733 1.839023
C 2.025713 1.599727 1.563715
C 3.418862 1.409337 1.67322
C 3.944504 0.180937 2.027069
C 3.091848 -0.904363 2.282259
C 1.721242 -0.737297 2.195515
H -0.31058 4.009684 0.86682
H -1.823556 2.165038 1.627716
O -1.072622 -0.251482 2.140675
H 1.031494 -1.562532 2.3614
H 3.508889 -1.882801 2.519487
H 5.026239 0.05021 2.082645

H 4.081987 2.249015 1.452031

TS_A-B_ortho_anion_toluene_conf6

C -1.391236 1.448013 -0.127153
C -0.601013 2.294564 1.666885
C 1.40213 3.319876 0.758976
C 1.089795 2.795237 -2.621956
C 1.840963 1.668491 -2.805254
C 1.506984 0.434186 -2.241499
C 0.431816 0.292562 -1.415994
C -0.352271 1.439282 -1.120838
C -0.067027 2.69975 -1.786519
C 3.459961 0.229696 -3.2326
H 1.348493 3.732151 -3.105767
H 0.197518 -0.673375 -0.983428
H 3.843498 -0.265022 -4.132063
O 2.966738 1.517513 -3.581035
O 2.397868 -0.52627 -2.69375
H 4.257773 0.346054 -2.473531
N -0.938348 3.716765 -1.570141
S -0.568319 5.216003 -2.022119
O -0.727383 5.45577 -3.469693
O 0.668967 5.740173 -1.405581
C -2.415488 -2.012737 1.119084
C -3.800065 -1.873395 1.140381
C -1.600405 -0.954672 0.731609
H -4.432895 -2.705622 1.447085
H -0.522541 -1.076559 0.783491
C -4.366112 -0.650194 0.785097
C -2.151199 0.27692 0.333277
H -5.447583 -0.518015 0.80911
C -3.551056 0.405318 0.398683
H -3.996377 1.360497 0.118096
H -1.957107 -2.951678 1.427088
C -1.667131 6.991851 -0.219355
C -2.726011 7.671533 0.380459
C -1.926212 6.061456 -1.217556
H -2.524029 8.400747 1.166616

C -4.046394 7.434838 -0.004455
C -3.237758 5.815711 -1.624718
H -3.422341 5.088031 -2.412625
C -4.283993 6.49563 -1.017851
H -5.310474 6.299893 -1.332677
H -0.634268 7.167972 0.074787
C -5.186508 8.15859 0.648071
H -4.830914 8.901945 1.370816
H -5.811334 8.683562 -0.086905
H -5.850927 7.469368 1.186905
H -2.02692 2.326347 -0.239948
H 1.897993 4.162561 0.279495
C 0.114574 3.439268 1.196273
C 0.152305 1.142435 2.160471
C 1.499695 0.991247 1.580822
C 2.092381 2.068017 0.864383
C 3.359737 1.859282 0.283858
C 4.00657 0.63881 0.401838
C 3.419665 -0.41406 1.116947
C 2.179449 -0.223525 1.708879
H -0.413245 4.382543 1.064072
H -1.569984 2.437311 2.147131
O -0.330608 0.305832 2.941696
H 1.687228 -1.01332 2.277336
H 3.931323 -1.372254 1.199428
H 4.984273 0.499905 -0.06241
H 3.8182 2.673675 -0.279587

TS_A-B_ortho_anion_toluene_conf7

C -1.077515 0.935841 0.26394
C -0.230769 2.072691 2.00603
C 0.960984 4.036312 1.18243
C -4.693688 2.005496 0.492312
C -4.813065 2.5745 -0.745448
C -3.764189 2.632759 -1.662781
C -2.532445 2.126947 -1.366997
C -2.329494 1.522983 -0.093758
C -3.428851 1.450092 0.859911

C -5.494119 3.712553 -2.525913
H -5.516049 2.013687 1.197854
H -1.743488 2.122967 -2.111375
H -6.203756 3.467067 -3.325977
O -5.938687 3.12531 -1.310143
O -4.221406 3.190028 -2.847011
H -5.430858 4.809004 -2.394887
N -3.145655 0.854272 2.04367
S -4.281515 0.57065 3.163885
O -5.232408 1.67468 3.40041
O -3.596026 -0.006187 4.319988
C 1.750753 1.843263 -2.096362
C 2.492794 0.66527 -2.074945
C 0.569897 1.946692 -1.370055
H 3.422881 0.592222 -2.637511
H 0.037967 2.894409 -1.342673
C 2.049958 -0.409127 -1.304427
C 0.10125 0.8684 -0.594976
H 2.634617 -1.327452 -1.26037
C 0.876711 -0.306902 -0.570164
H 0.554921 -1.126536 0.069476
H 2.107723 2.702459 -2.66365
C -6.565746 -0.559667 2.046567
C -7.280772 -1.604709 1.4636
C -5.249148 -0.762946 2.443341
H -8.315334 -1.445824 1.1549
C -6.696734 -2.856481 1.266591
C -4.647804 -2.00956 2.259363
H -3.61804 -2.153367 2.581859
C -5.368959 -3.042053 1.677675
H -4.898845 -4.016734 1.536836
H -7.023909 0.412795 2.216403
C -7.460942 -3.9769 0.626457
H -8.508228 -3.70349 0.453217
H -7.031309 -4.258765 -0.344642
H -7.452338 -4.883527 1.245999
H -1.142402 0.136557 1.003676
H 0.961011 5.070942 0.836482

C -0.195594 3.43009 1.589337
C 0.999344 1.306557 2.129057
C 2.237562 1.988473 1.694112
C 2.208758 3.330334 1.228458
C 3.418432 3.920045 0.811632
C 4.608759 3.213251 0.848745
C 4.63001 1.888987 1.307535
C 3.450206 1.291779 1.722968
H -1.137191 3.981615 1.558009
H -1.099083 1.699423 2.549828
O 1.016886 0.121662 2.512265
H 3.417432 0.261683 2.076519
H 5.567477 1.33361 1.328678
H 5.532295 3.690491 0.519194
H 3.403139 4.950221 0.45063

TS_A-B_ortho_anion_toluene_conf8
C 12.327617 -14.379704 -1.120393
C 13.206714 -16.053793 -2.286861
C 12.191158 -16.223049 -4.494136
C 10.426013 -16.268742 1.520773
C 11.307579 -16.127213 2.549402
C 12.501792 -15.401504 2.442735
C 12.874025 -14.799493 1.280899
C 12.016384 -14.922752 0.150841
C 10.755805 -15.652459 0.27147
C 12.434689 -16.333704 4.449632
H 9.512029 -16.843372 1.626606
H 13.780317 -14.204976 1.231849
H 12.267558 -15.937167 5.458932
O 11.181983 -16.594174 3.83663
O 13.128757 -15.3749 3.676986
H 13.018025 -17.271718 4.49763
N 9.979293 -15.688284 -0.826874
S 8.462217 -16.24369 -0.755548
O 7.558034 -15.273941 -0.107345
O 8.315434 -17.645617 -0.315279
C 15.60619 -12.448915 -1.316249

C 15.226289 -11.370643 -2.113486
C 14.687374 -13.434573 -0.973913
H 15.949196 -10.60129 -2.381642
H 15.01931 -14.293372 -0.393281
C 13.914831 -11.301692 -2.578871
C 13.350411 -13.369543 -1.408528
H 13.603686 -10.474986 -3.217106
C 12.998244 -12.288864 -2.236667
H 11.975248 -12.235048 -2.61001
H 16.635119 -12.53216 -0.967077
C 7.086326 -17.112095 -2.94422
C 6.681731 -17.084661 -4.272841
C 8.069878 -16.230161 -2.500816
H 5.921603 -17.786553 -4.620907
C 7.255521 -16.192042 -5.183277
C 8.63904 -15.319737 -3.387265
H 9.420146 -14.65153 -3.032949
C 8.232399 -15.308726 -4.71698
H 8.70356 -14.615482 -5.416404
H 6.668303 -17.830411 -2.241355
C 6.881581 -16.230877 -6.634443
H 5.831974 -16.514969 -6.78069
H 7.040371 -15.262465 -7.1242
H 7.488784 -16.967897 -7.181539
H 11.463181 -14.317413 -1.778716
H 12.099893 -15.884187 -5.527837
C 13.10792 -15.65785 -3.651458
C 12.437476 -17.183692 -1.790199
C 11.439229 -17.755287 -2.713688
C 11.350031 -17.296415 -4.054847
C 10.428139 -17.92355 -4.918357
C 9.619088 -18.951376 -4.471631
C 9.702854 -19.387272 -3.140082
C 10.610836 -18.797154 -2.278855
H 13.758891 -14.857568 -4.010887
H 14.131758 -15.860575 -1.746703
O 12.612844 -17.631528 -0.641638
H 10.684666 -19.092959 -1.234118

H 9.035038 -20.168647 -2.778135
H 8.896258 -19.406034 -5.150709
H 10.349265 -17.568167 -5.948445

TS_A-B_ortho_anion_toluene_conf9
C 12.52555 -14.84145 -1.359077
C 13.471308 -16.173962 -2.73169
C 12.232515 -15.604582 -4.739292
C 10.312318 -12.938648 -3.740705
C 9.121062 -13.54522 -3.456326
C 8.971789 -14.47855 -2.426965
C 10.031108 -14.888221 -1.673282
C 11.314519 -14.353412 -1.961343
C 11.453525 -13.309226 -2.963046
C 7.074175 -14.368609 -3.533772
H 10.393659 -12.187865 -4.520787
H 9.892379 -15.620739 -0.886198
H 6.069287 -13.974101 -3.345524
O 7.8973 -13.327516 -4.045575
O 7.635096 -14.830101 -2.324655
H 7.043597 -15.19471 -4.270648
N 12.679898 -12.744436 -3.08949
S 13.022987 -11.799643 -4.345642
O 12.506585 -10.423947 -4.210033
O 12.820101 -12.447573 -5.65888
C 11.981391 -17.155798 1.62891
C 12.946807 -16.667612 2.504561
C 11.825894 -16.603111 0.362276
H 13.064884 -17.106815 3.494661
H 11.1042 -17.045355 -0.318409
C 13.774417 -15.624732 2.091873
C 12.624767 -15.525637 -0.06172
H 14.54518 -15.238813 2.758672
C 13.618039 -15.074089 0.826972
H 14.264818 -14.256185 0.507397
H 11.350176 -17.992889 1.924679
C 15.654527 -12.187566 -5.079243
C 17.032651 -12.084703 -4.896587

C 14.79441 -11.702704 -4.102349
H 17.709263 -12.468658 -5.661689
C 17.567197 -11.501727 -3.746856
C 15.306826 -11.106917 -2.949469
H 14.618623 -10.728464 -2.196127
C 16.680316 -11.013568 -2.7769
H 17.081121 -10.552769 -1.872401
H 15.228292 -12.644482 -5.97022
C 19.049938 -11.403519 -3.543789
H 19.601411 -11.763822 -4.419838
H 19.368823 -10.370002 -3.353259
H 19.382283 -11.996719 -2.680905
H 13.343527 -14.136997 -1.510265
H 12.099621 -14.9822 -5.622964
C 13.362979 -15.490023 -3.982562
C 12.650042 -17.361672 -2.502336
C 11.384504 -17.391671 -3.258782
C 11.181128 -16.495499 -4.344782
C 9.935697 -16.526042 -5.004433
C 8.935941 -17.397889 -4.601438
C 9.1472 -18.282641 -3.53527
C 10.372327 -18.279738 -2.883975
H 14.132428 -14.774559 -4.268552
H 14.433493 -16.186225 -2.21783
O 12.946611 -18.237015 -1.672452
H 10.582304 -18.953599 -2.052656
H 8.353927 -18.959529 -3.220181
H 7.977572 -17.397089 -5.123197
H 9.763583 -15.834234 -5.830759

TS_A-B_ortho_anion_toluene_conf10
C 11.897654 -15.555392 -1.304059
C 13.469255 -16.987105 -2.029995
C 14.13347 -16.375171 -4.296942
C 13.919585 -13.460869 1.104411
C 13.707097 -12.237234 0.532171
C 12.945668 -12.056408 -0.621702
C 12.36652 -13.111013 -1.264071

C 12.538534 -14.41771 -0.724526
C 13.330008 -14.606068 0.484441
C 13.531217 -10.055455 0.141117
H 14.55175 -13.574283 1.977206
H 11.734242 -12.950196 -2.130527
H 12.762455 -9.536769 0.743243
O 14.185646 -11.019879 0.95423
O 12.92443 -10.711346 -0.95379
H 14.271173 -9.332924 -0.227855
N 13.438877 -15.882855 0.92479
S 14.149268 -16.264345 2.330102
O 15.387488 -15.524701 2.645564
O 14.185439 -17.724791 2.399503
C 10.481809 -14.884459 -4.758616
C 9.340473 -15.681316 -4.730086
C 11.313622 -14.804191 -3.647608
H 8.696293 -15.749564 -5.606023
H 12.235751 -14.231454 -3.70742
C 9.044167 -16.412641 -3.580111
C 11.02374 -15.525502 -2.473457
H 8.166956 -17.058377 -3.554692
C 9.876042 -16.34162 -2.471629
H 9.673251 -16.948005 -1.590712
H 10.743876 -14.339109 -5.664943
C 13.183847 -14.650069 4.380321
C 12.210321 -14.245069 5.292334
C 12.938731 -15.737406 3.549609
H 12.403774 -13.393865 5.947259
C 10.988029 -14.911485 5.386441
C 11.723025 -16.419649 3.629853
H 11.550852 -17.272341 2.975395
C 10.762217 -16.007701 4.541641
H 9.813909 -16.543802 4.606022
H 14.145164 -14.143734 4.31804
C 9.936692 -14.470214 6.360651
H 10.292217 -13.648825 6.993504
H 9.029403 -14.120962 5.849011
H 9.625053 -15.28856 7.023451

H 11.692852 -16.370242 -0.607747
H 14.736665 -15.775455 -4.980055
C 14.270042 -16.249246 -2.941453
C 12.546735 -18.002478 -2.513688
C 12.429792 -18.130344 -3.981953
C 13.211607 -17.323863 -4.851366
C 13.041681 -17.480023 -6.241285
C 12.135696 -18.39389 -6.753079
C 11.36683 -19.186105 -5.889371
C 11.519238 -19.046481 -4.518979
H 14.983352 -15.530829 -2.532948
H 13.762586 -17.050077 -0.981551
O 11.838579 -18.684921 -1.749464
H 10.935184 -19.632292 -3.809815
H 10.650105 -19.899518 -6.295686
H 12.021275 -18.496101 -7.832772
H 13.637029 -16.861514 -6.915692

TS_A-B_ortho_anion_toluene_conf11
C 11.420546 -15.28304 -2.00975
C 13.508646 -16.137581 -2.049382
C 14.227183 -16.320165 -4.363938
C 9.518129 -18.240126 -0.67751
C 9.401492 -17.840106 0.621009
C 9.872522 -16.609256 1.090685
C 10.517363 -15.731647 0.273832
C 10.706156 -16.090537 -1.090852
C 10.168278 -17.355158 -1.593479
C 9.089737 -17.753006 2.816245
H 9.175205 -19.214046 -1.006772
H 10.842089 -14.76583 0.646499
H 8.188757 -17.661013 3.435843
O 8.782507 -18.506483 1.653504
O 9.523488 -16.466374 2.42363
H 9.891763 -18.265103 3.379389
N 10.314873 -17.564049 -2.918977
S 9.649528 -18.820616 -3.691382
O 9.743322 -20.125796 -3.008304

O 10.084487 -18.733963 -5.085995
C 12.788312 -12.017321 -0.604109
C 12.555235 -11.155968 -1.674956
C 12.424461 -13.35673 -0.680828
H 12.843766 -10.10747 -1.612504
H 12.667173 -14.021618 0.145963
C 11.968761 -11.660509 -2.833817
C 11.803546 -13.881189 -1.830422
H 11.796369 -11.006705 -3.688509
C 11.607827 -13.000429 -2.909885
H 11.160883 -13.393648 -3.822886
H 13.273732 -11.645649 0.298083
C 6.985112 -19.135366 -2.94111
C 5.638964 -18.773138 -2.943026
C 7.895986 -18.408637 -3.699223
H 4.924197 -19.345854 -2.349607
C 5.184592 -17.687689 -3.692956
C 7.459814 -17.322562 -4.460134
H 8.185193 -16.770352 -5.055068
C 6.117901 -16.970497 -4.454945
H 5.778444 -16.123133 -5.053371
H 7.338886 -19.992123 -2.370873
C 3.738784 -17.28899 -3.683584
H 3.126912 -18.004349 -3.121825
H 3.592263 -16.301831 -3.223855
H 3.325613 -17.224284 -4.699103
H 11.254397 -15.565926 -3.048142
H 14.638674 -15.872878 -5.270238
C 14.098246 -15.58372 -3.219082
C 13.200226 -17.555048 -1.975468
C 13.333677 -18.319923 -3.229734
C 13.848324 -17.701477 -4.39999
C 13.987964 -18.483125 -5.564843
C 13.628183 -19.818111 -5.573496
C 13.119942 -20.419557 -4.413301
C 12.985952 -19.675042 -3.255043
H 14.405248 -14.535632 -3.205618
H 13.665779 -15.650839 -1.088418

O 12.825107 -18.082003 -0.910515
H 12.567629 -20.102625 -2.34562
H 12.803533 -21.461641 -4.431577
H 13.728743 -20.401074 -6.489299
H 14.379086 -18.012026 -6.468796

TS_A-B_ortho_anion_toluene_conf12
C 12.229711 -15.198856 -2.048771
C 13.561808 -16.884049 -1.905953
C 14.266151 -17.003749 -4.223214
C 12.794909 -14.270559 -5.652999
C 11.647653 -14.786947 -6.188624
C 10.624272 -15.330233 -5.410464
C 10.737393 -15.434584 -4.055869
C 11.932577 -14.980715 -3.438348
C 12.95523 -14.320431 -4.234456
C 10.12511 -15.662583 -7.526991
H 13.573461 -13.85861 -6.285106
H 9.935068 -15.870205 -3.471545
H 9.394816 -15.261529 -8.238931
O 11.274135 -14.823607 -7.512356
O 9.564236 -15.677808 -6.232505
H 10.434669 -16.686832 -7.811521
N 13.992223 -13.774891 -3.544376
S 15.335315 -13.261774 -4.257654
O 15.804564 -14.095749 -5.383106
O 16.288852 -12.951391 -3.187377
C 9.284648 -16.471593 0.019329
C 9.433952 -15.702382 1.1694
C 10.180127 -16.35035 -1.037726
H 8.730438 -15.803811 1.995184
H 10.071691 -17.008058 -1.895051
C 10.506536 -14.816717 1.26133
C 11.247957 -15.435848 -0.982596
H 10.647747 -14.216987 2.160168
C 11.400169 -14.696876 0.205602
H 12.237318 -14.002004 0.279019
H 8.47184 -17.193217 -0.052555

C 14.974039 -11.474278 -6.36108
C 14.623299 -10.23827 -6.901609
C 14.908363 -11.671417 -4.986513
H 14.676911 -10.084967 -7.980779
C 14.2025 -9.187388 -6.085633
C 14.494373 -10.630301 -4.153361
H 14.452676 -10.796739 -3.078301
C 14.146505 -9.404236 -4.701433
H 13.823885 -8.591815 -4.047873
H 15.312299 -12.294266 -6.992381
C 13.811071 -7.861637 -6.667292
H 14.001311 -7.822834 -7.746127
H 12.743595 -7.650123 -6.515742
H 14.362089 -7.032823 -6.203025
H 13.04829 -14.557879 -1.722317
H 14.931689 -16.693273 -5.027832
C 14.517038 -16.623804 -2.935835
C 12.587415 -17.95686 -2.092638
C 12.267862 -18.263579 -3.499202
C 13.091601 -17.760344 -4.544004
C 12.715737 -18.035705 -5.874386
C 11.574756 -18.771317 -6.156024
C 10.773641 -19.270936 -5.120314
C 11.134166 -19.022245 -3.803812
H 15.386597 -16.008841 -2.711164
H 13.839003 -16.699633 -0.867413
O 12.006612 -18.513372 -1.145714
H 10.542989 -19.393534 -2.966103
H 9.874991 -19.842275 -5.35002
H 11.30236 -18.966915 -7.194431
H 13.331805 -17.642478 -6.684797

TS_A-B_ortho_anion_toluene_conf13

C -0.981415 -0.290653 0.498137
C -1.831472 -0.418373 -1.57527
C -3.862456 0.928558 -1.701058
C 1.908329 2.069297 -0.102322
C 1.548612 3.053299 0.776441

C 0.396071 2.993254 1.55962
C -0.460027 1.934134 1.484455
C -0.154798 0.871206 0.587407
C 1.054154 0.929543 -0.224132
C 1.36726 4.951313 1.913897
H 2.789016 2.165199 -0.726337
H -1.318427 1.869087 2.144501
H 1.939529 5.360136 2.756075
O 2.238503 4.204066 1.075285
O 0.356585 4.093627 2.402101
H 0.915643 5.771604 1.325167
N 1.262411 -0.125211 -1.048653
S 2.614953 -0.288601 -1.925895
O 3.101928 0.940489 -2.582903
O 2.438389 -1.484642 -2.748934
C -4.357402 0.134087 2.173389
C -4.568339 -1.152646 2.661609
C -3.185091 0.44772 1.495199
H -5.492891 -1.397092 3.183481
H -3.063775 1.437219 1.061398
C -3.598507 -2.13248 2.451835
C -2.190413 -0.526038 1.282342
H -3.76305 -3.148878 2.80818
C -2.431171 -1.82636 1.766524
H -1.695732 -2.601149 1.557861
H -5.125155 0.897329 2.297896
C 4.898981 0.158907 -0.400368
C 5.829444 -0.186311 0.578696
C 3.855872 -0.710952 -0.695481
H 6.650067 0.495136 0.809065
C 5.731669 -1.392354 1.273516
C 3.741902 -1.924246 -0.013843
H 2.92122 -2.595255 -0.261482
C 4.673224 -2.256724 0.958837
H 4.584586 -3.206106 1.489511
H 4.980837 1.091787 -0.955016
C 6.727662 -1.760431 2.332547
H 7.51523 -1.003757 2.425904

H 6.253602 -1.864575 3.318002
H 7.215122 -2.72104 2.118235
H -0.464352 -1.1978 0.181386
H -4.355759 1.891694 -1.839558
C -2.502326 0.814748 -1.791231
C -2.588219 -1.642828 -1.365978
C -4.057441 -1.495023 -1.286377
C -4.675132 -0.226349 -1.449849
C -6.077945 -0.146965 -1.345336
C -6.840928 -1.273371 -1.086486
C -6.224893 -2.521957 -0.92478
C -4.846078 -2.620406 -1.025085
H -1.89619 1.700004 -1.99311
H -0.794286 -0.529996 -1.892905
O -2.041623 -2.748484 -1.192893
H -4.323727 -3.567829 -0.895964
H -6.827859 -3.405124 -0.714601
H -7.925214 -1.186622 -1.008428
H -6.559133 0.825335 -1.467505

TS_A-B_ortho_anion_toluene_conf14
C -1.63379 -0.119074 1.074473
C -1.681357 0.332496 -1.136002
C -3.931898 1.027863 -1.734543
C -1.014225 -3.826519 0.984687
C 0.324208 -3.816861 1.245893
C 1.047222 -2.647753 1.506829
C 0.456368 -1.421495 1.484566
C -0.934117 -1.345509 1.188451
C -1.700229 -2.571971 0.965784
C 2.46891 -4.343788 1.463114
H -1.543325 -4.743884 0.75393
H 1.0244 -0.529512 1.727212
H 3.026701 -4.879573 2.241509
O 1.167271 -4.898763 1.354911
O 2.354671 -2.98106 1.820522
H 2.984942 -4.435665 0.489539
N -3.027856 -2.404894 0.787026

S -4.063042 -3.644156 0.684868
O -3.633024 -4.780728 -0.153337
O -5.386209 -3.066572 0.445577
C 0.446961 3.01616 1.667915
C -0.460859 3.799534 2.378711
C 0.097769 1.740776 1.239302
H -0.18504 4.799522 2.71106
H 0.803127 1.170443 0.637824
C -1.733035 3.293768 2.637937
C -1.171338 1.197967 1.516427
H -2.462916 3.899011 3.175217
C -2.08136 2.019312 2.20688
H -3.081696 1.632609 2.401072
H 1.435557 3.408411 1.430176
C -3.591737 -5.538781 2.67079
C -3.616573 -5.998381 3.986584
C -4.104983 -4.282644 2.369091
H -3.216438 -6.986032 4.222128
C -4.145807 -5.216568 5.014142
C -4.643574 -3.487844 3.382397
H -5.049319 -2.510347 3.127406
C -4.662351 -3.95427 4.688933
H -5.086925 -3.332774 5.479383
H -3.194257 -6.151816 1.864102
C -4.156593 -5.703606 6.43262
H -3.841989 -6.751444 6.501667
H -3.479233 -5.117238 7.068915
H -5.154474 -5.625462 6.884312
H -2.715816 -0.239452 1.102086
H -4.706818 1.793911 -1.792891
C -2.681757 1.333893 -1.272859
C -1.895904 -1.005382 -1.659083
C -3.258689 -1.311268 -2.133742
C -4.252641 -0.297629 -2.174108
C -5.530001 -0.63126 -2.668514
C -5.813669 -1.914032 -3.099429
C -4.826242 -2.908535 -3.053549
C -3.562124 -2.601085 -2.583126

H -2.450665 2.352689 -0.954142
H -0.637606 0.627175 -1.042171
O -0.97763 -1.847286 -1.682784
H -2.781633 -3.355591 -2.504709
H -5.061884 -3.927369 -3.35819
H -6.812541 -2.155392 -3.463672
H -6.299558 0.142652 -2.698046

TS_A-B_ortho_anion_toluene_conf15
C -1.593548 0.422585 0.500295
C -1.553426 -0.297019 -1.530447
C -3.792808 0.35004 -2.198514
C -4.99336 2.038048 0.52547
C -5.682677 1.072913 1.206116
C -5.066938 -0.046459 1.768285
C -3.731708 -0.276563 1.617244
C -2.964787 0.651004 0.864345
C -3.58398 1.868896 0.364546
C -7.24269 -0.270132 2.006504
H -5.502488 2.893118 0.095302
H -3.275684 -1.159283 2.050506
H -7.954475 -0.221666 2.83847
O -7.026724 1.041109 1.498188
O -6.010964 -0.78095 2.467629
H -7.63032 -0.910127 1.190449
N -2.746667 2.774657 -0.207525
S -3.273946 4.046955 -1.031709
O -4.461106 3.801045 -1.875804
O -2.098454 4.658286 -1.66074
C 0.073555 -2.499577 2.305526
C 1.316556 -1.941646 2.589429
C -0.890642 -1.7737 1.61436
H 2.067814 -2.516824 3.129696
H -1.826513 -2.259363 1.355123
C 1.596426 -0.646829 2.155571
C -0.644043 -0.451854 1.200355
H 2.569453 -0.198898 2.355737
C 0.633572 0.076527 1.465188

H 0.85373 1.090143 1.128831
H -0.146685 -3.522749 2.607522
C -5.131088 5.656085 0.278457
C -5.522574 6.556152 1.268354
C -3.810691 5.225469 0.219301
H -6.55934 6.894003 1.312887
C -4.610445 7.035233 2.209424
C -2.882418 5.69726 1.14944
H -1.851189 5.353578 1.089546
C -3.282494 6.591727 2.131379
H -2.55498 6.958821 2.857467
H -5.836927 5.284941 -0.462769
C -5.033914 7.992449 3.283465
H -6.077523 8.30331 3.157677
H -4.943366 7.547379 4.283886
H -4.41583 8.900273 3.289089
H -1.126839 1.344687 0.154862
H -4.486588 1.092696 -2.590936
C -2.449317 0.594719 -2.195484
C -1.96722 -1.677069 -1.287827
C -3.425082 -1.879867 -1.199547
C -4.315416 -0.856324 -1.62752
C -5.69942 -1.075736 -1.474963
C -6.17876 -2.252178 -0.919798
C -5.294905 -3.259089 -0.508373
C -3.929497 -3.06799 -0.663019
H -2.068842 1.536548 -2.586812
H -0.478801 -0.161348 -1.657149
O -1.15778 -2.598538 -1.08742
H -3.205578 -3.824953 -0.359828
H -5.67893 -4.177901 -0.066675
H -7.254635 -2.396307 -0.808297
H -6.391493 -0.292267 -1.788123

TS_A-B_ortho_anion_toluene_conf16

C 11.916972 -14.845851 -1.267183
C 13.17175 -16.692125 -1.494969
C 14.060945 -16.792405 -3.765166

C 14.302845 -12.652496 0.676014
C 14.376921 -11.608563 -0.204169
C 13.687456 -11.581952 -1.416154
C 12.893885 -12.620795 -1.805208
C 12.770462 -13.746976 -0.942668
C 13.482042 -13.770202 0.328681
C 14.94833 -9.764963 -1.301852
H 14.883376 -12.660708 1.590957
H 12.313653 -12.560611 -2.719723
H 14.717399 -8.706638 -1.126837
O 15.068296 -10.430479 -0.051368
O 13.900447 -10.36134 -2.038048
H 15.901577 -9.861256 -1.854507
N 13.304371 -14.880646 1.084709
S 13.908355 -15.016374 2.582191
O 15.290651 -14.529572 2.759554
O 13.591562 -16.368451 3.041292
C 10.752544 -14.832475 -4.877018
C 9.471686 -15.360477 -4.737882
C 11.555047 -14.625662 -3.760948
H 8.850108 -15.531593 -5.616093
H 12.576428 -14.276878 -3.891359
C 9.002577 -15.694354 -3.467875
C 11.094795 -14.947758 -2.469668
H 8.011091 -16.130252 -3.349141
C 9.80366 -15.497729 -2.351788
H 9.458374 -15.802614 -1.365683
H 11.143468 -14.603405 -5.868063
C 13.387464 -12.742435 4.099473
C 12.550337 -11.888981 4.816624
C 12.871207 -13.908316 3.546177
H 12.956004 -10.975038 5.253665
C 11.197031 -12.182373 4.988265
C 11.52047 -14.22207 3.709556
H 11.134556 -15.142145 3.274178
C 10.696985 -13.365219 4.425014
H 9.6421 -13.612764 4.55456
H 14.446925 -12.524884 3.978291

C 10.293742 -11.2589 5.750117
H 10.849357 -10.421346 6.187552
H 9.511678 -10.833157 5.106667
H 9.776121 -11.777118 6.56841
H 11.529164 -15.393793 -0.406987
H 14.810432 -16.507012 -4.504701
C 14.154618 -16.368725 -2.468023
C 12.080438 -17.596611 -1.821296
C 12.0078 -18.049374 -3.227162
C 12.98533 -17.647658 -4.176386
C 12.852968 -18.09835 -5.504622
C 11.79637 -18.909853 -5.882659
C 10.834151 -19.300992 -4.941502
C 10.947905 -18.868778 -3.629529
H 14.98354 -15.725799 -2.165797
H 13.397307 -16.555963 -0.436924
O 11.210388 -17.924891 -0.992814
H 10.218123 -19.139238 -2.867039
H 10.000291 -19.934129 -5.244088
H 11.714161 -19.244797 -6.917221
H 13.599402 -17.791613 -6.239827

TS_A-B_ortho_anion_toluene_conf17

C 11.593768 -14.623781 -2.070927
C 13.421063 -15.906089 -1.746825
C 14.106027 -16.844286 -3.881887
C 9.033237 -16.63633 -0.19168
C 8.994771 -15.899531 0.955163
C 9.733683 -14.725695 1.138688
C 10.577466 -14.254581 0.17976
C 10.696825 -14.986137 -1.035723
C 9.885105 -16.184725 -1.247653
C 8.681203 -15.182134 3.031291
H 8.477357 -17.561001 -0.294315
H 11.113566 -13.322468 0.324622
H 7.818145 -14.735841 3.541227
O 8.222573 -16.1222 2.072064
O 9.408538 -14.1698 2.365189

H 9.333715 -15.696695 3.760732
N 9.997841 -16.756059 -2.465489
S 9.069354 -17.990134 -2.948357
O 8.847798 -19.061881 -1.957724
O 9.531932 -18.36517 -4.285083
C 13.671257 -11.498974 -1.415612
C 13.664563 -10.912549 -2.680169
C 13.003105 -12.696326 -1.187062
H 14.191225 -9.975522 -2.856618
H 13.069395 -13.163171 -0.206133
C 12.993584 -11.553471 -3.719593
C 12.293681 -13.345799 -2.215171
H 12.99335 -11.120071 -4.719514
C 12.327799 -12.751596 -3.489977
H 11.814733 -13.255731 -4.308872
H 14.216654 -11.0263 -0.599011
C 6.394488 -17.50519 -2.339999
C 5.1691 -16.868208 -2.529596
C 7.459513 -17.217672 -3.185953
H 4.332841 -17.096651 -1.866657
C 4.989162 -15.940163 -3.555975
C 7.297562 -16.295818 -4.2217
H 8.139313 -16.089297 -4.880375
C 6.073787 -15.667505 -4.40163
H 5.948271 -14.949781 -5.214332
H 6.532136 -18.242405 -1.551371
C 3.674776 -15.244686 -3.751203
H 2.907096 -15.636342 -3.073724
H 3.754501 -14.164683 -3.565379
H 3.300598 -15.357592 -4.777578
H 11.379007 -15.119073 -3.016705
H 14.62254 -16.75354 -4.838828
C 14.138086 -15.823857 -2.972368
C 12.787968 -17.148979 -1.341507
C 12.753053 -18.224971 -2.350002
C 13.414696 -18.066485 -3.596602
C 13.383041 -19.137456 -4.512701
C 12.719756 -20.312612 -4.211082

C 12.067589 -20.458703 -2.978466
C 12.095981 -19.42551 -2.058993
H 14.681556 -14.904412 -3.200801
H 13.678754 -15.23422 -0.92998
O 12.287777 -17.281962 -0.208095
H 11.574498 -19.493637 -1.105994
H 11.514539 -21.370642 -2.757408
H 12.693571 -21.122918 -4.940058
H 13.886419 -19.020182 -5.474407

TS_A-B_ortho_anion_toluene_conf18
C 12.495247 -16.030478 -1.99853
C 14.09394 -17.12719 -0.892721
C 11.313113 -13.3674 0.40069
C 12.091541 -12.337979 -0.037832
C 12.972737 -12.450985 -1.120223
C 13.126424 -13.624763 -1.794862
C 12.364508 -14.756444 -1.382801
C 11.408484 -14.628555 -0.279841
C 12.955559 -10.332726 -0.456569
H 10.638199 -13.245807 1.241678
H 13.795554 -13.684574 -2.646645
H 12.297635 -9.670027 -1.048295
O 12.168883 -11.063404 0.471248
O 13.618108 -11.241515 -1.313927
H 13.698437 -9.737031 0.089678
N 10.657546 -15.70373 0.008352
S 9.444329 -15.581247 1.086024
O 8.384288 -14.648262 0.65647
O 9.919765 -15.435396 2.474099
C 14.634619 -16.149669 -5.155142
C 13.836244 -16.94152 -5.977408
C 14.222584 -15.82798 -3.866251
H 14.16271 -17.193929 -6.985331
H 14.890785 -15.252785 -3.228033
C 12.623779 -17.420911 -5.48537
C 12.992905 -16.283317 -3.357076
H 11.993009 -18.05447 -6.108131

C 12.215941 -17.102406 -4.195948
H 11.27348 -17.490938 -3.811076
H 15.597461 -15.787775 -5.514873
C 7.421807 -17.40544 0.67741
C 6.905679 -18.697873 0.593188
C 8.778746 -17.230785 0.917063
H 5.840796 -18.842275 0.401243
C 7.728452 -19.817007 0.739677
C 9.615581 -18.333819 1.075804
H 10.675123 -18.18212 1.260338
C 9.094744 -19.615689 0.980611
H 9.772171 -20.463192 1.089202
H 6.791059 -16.52829 0.548112
C 7.1697 -21.205428 0.638097
H 7.65174 -21.778834 -0.164945
H 7.328416 -21.775713 1.563228
H 6.091743 -21.194577 0.436769
H 11.719698 -16.729195 -1.682865
H 14.809713 -16.435401 -1.337321
C 13.983112 -18.433414 -1.444658
C 13.416244 -19.471436 -0.756009
C 12.97296 -19.335282 0.614647
C 13.220973 -18.019494 1.240718
C 13.788149 -16.95018 0.500817
C 13.996058 -15.716226 1.146748
C 13.622401 -15.535365 2.467536
C 13.055066 -16.590093 3.191277
C 12.868131 -17.818754 2.580344
H 14.305595 -18.582606 -2.477654
H 13.273467 -20.446529 -1.220342
O 12.412531 -20.261986 1.234861
H 12.419298 -18.658899 3.109354
H 12.736795 -16.434592 4.220682
H 13.754278 -14.561072 2.937668
H 14.429595 -14.890936 0.579556

TS_A-B_ortho_anion_toluene_conf19

C 12.186121 -16.800564 -1.109857

C 14.290611 -17.141841 -0.693632
C 10.552855 -19.709513 0.603841
C 10.494869 -19.207708 1.879669
C 10.89828 -17.908579 2.197565
C 11.396145 -17.056382 1.254716
C 11.520953 -17.536617 -0.080137
C 11.123882 -18.890859 -0.402692
C 10.60562 -19.017285 4.066468
H 10.242917 -20.719184 0.356969
H 11.785871 -16.084838 1.543912
H 9.899533 -19.007259 4.903313
O 10.083507 -19.83906 3.025974
O 10.747243 -17.703593 3.553993
H 11.596589 -19.402514 4.360303
N 11.379141 -19.371961 -1.663324
S 10.147696 -19.705564 -2.61726
O 8.976256 -20.298118 -1.942773
O 10.652752 -20.349231 -3.835455
C 11.441912 -13.07787 -0.812558
C 12.150873 -12.580841 -1.902317
C 11.436746 -14.440551 -0.528714
H 12.155057 -11.51323 -2.118731
H 10.832936 -14.808284 0.296043
C 12.833555 -13.473208 -2.730869
C 12.14889 -15.35229 -1.325943
H 13.368812 -13.107243 -3.606786
C 12.823213 -14.831116 -2.44816
H 13.341247 -15.526659 -3.109907
H 10.875974 -12.398724 -0.175451
C 8.850086 -17.293 -2.223129
C 8.642985 -15.939107 -2.472224
C 9.638516 -18.046747 -3.092281
H 8.042372 -15.347643 -1.778411
C 9.213872 -15.310136 -3.581371
C 10.208118 -17.436962 -4.21114
H 10.829517 -18.038993 -4.872376
C 9.990205 -16.08607 -4.451696
H 10.447359 -15.610287 -5.322125

H 8.415263 -17.776103 -1.349005
C 9.016076 -13.844928 -3.830407
H 9.970816 -13.339391 -4.027268
H 8.368764 -13.65686 -4.699352
H 8.558031 -13.349819 -2.96583
H 12.246861 -17.371194 -2.037831
H 14.631086 -16.584056 -1.566666
C 14.316449 -18.565842 -0.743367
C 14.116647 -19.317143 0.380405
C 13.997049 -18.720424 1.693148
C 14.283471 -17.269159 1.769993
C 14.450402 -16.501472 0.591245
C 14.660848 -15.114605 0.71523
C 14.714648 -14.513725 1.96215
C 14.554183 -15.281097 3.124987
C 14.335727 -16.644578 3.022013
H 14.332188 -19.05377 -1.717207
H 13.985494 -20.396086 0.318002
O 13.670504 -19.368385 2.705711
H 14.172534 -17.270896 3.898325
H 14.59064 -14.801547 4.1029
H 14.877114 -13.438512 2.036801
H 14.764459 -14.513386 -0.189616

TS_A-B_ortho_anion_toluene_conf20
C 12.055095 -17.244759 -1.017229
C 14.296331 -17.008134 -0.593616
C 11.932848 -16.839148 -4.741963
C 11.468004 -18.081756 -5.087892
C 11.253454 -19.093873 -4.153204
C 11.475322 -18.904061 -2.817123
C 11.938052 -17.628809 -2.392827
C 12.205549 -16.594004 -3.369265
C 10.570331 -19.823501 -6.134122
H 12.097144 -16.052353 -5.469904
H 11.237232 -19.685438 -2.10339
H 9.480401 -19.736008 -6.299201
O 11.178819 -18.558632 -6.34604

O 10.821928 -20.235969 -4.80387
H 11.00061 -20.553986 -6.831134
N 12.77458 -15.429883 -2.922434
S 12.090313 -14.020813 -3.25315
O 11.427897 -13.922714 -4.568455
O 13.017931 -12.956013 -2.853907
C 11.414553 -19.94664 1.573205
C 10.924932 -19.171902 2.621097
C 11.785725 -19.354064 0.371659
H 10.657686 -19.637749 3.568324
H 12.236101 -19.968263 -0.40422
C 10.815814 -17.791096 2.45967
C 11.648123 -17.965143 0.170784
H 10.470969 -17.16724 3.283762
C 11.179353 -17.199375 1.259189
H 11.12163 -16.1157 1.140679
H 11.546471 -21.019744 1.704459
C 9.634341 -14.813067 -2.25448
C 8.709011 -14.98915 -1.232145
C 10.764767 -14.022877 -2.037834
H 7.833112 -15.617943 -1.399705
C 8.886585 -14.386183 0.018364
C 10.946395 -13.396428 -0.807064
H 11.831895 -12.781159 -0.65902
C 10.010479 -13.575064 0.206577
H 10.164426 -13.091807 1.173408
H 9.495792 -15.292009 -3.22304
C 7.924911 -14.643866 1.139202
H 7.947513 -13.842493 1.88739
H 6.892836 -14.740987 0.779944
H 8.16701 -15.579909 1.663357
H 12.03134 -16.162506 -0.895691
H 14.366645 -16.505384 -1.556348
C 14.713788 -18.350397 -0.446721
C 14.835378 -18.94145 0.784333
C 14.60355 -18.213692 2.017451
C 14.310729 -16.770288 1.864861
C 14.19133 -16.185411 0.579937

C 13.886268 -14.812087 0.477558
C 13.718206 -14.046582 1.618364
C 13.847429 -14.626618 2.890923
C 14.140261 -15.974781 3.005744
H 14.870973 -18.943475 -1.350631
H 15.098225 -19.994222 0.885899
O 14.650961 -18.757547 3.135571
H 14.233661 -16.465375 3.973955
H 13.712166 -14.015295 3.783356
H 13.483758 -12.98543 1.526024
H 13.774771 -14.374805 -0.517858

TS_A-B_para_anion_toluene
C 11.116725 -15.520712 -1.975655
C 12.824938 -16.959113 -1.466451
C 9.45598 -15.289143 1.378442
C 9.717114 -13.97368 1.664715
C 10.484029 -13.158119 0.834173
C 11.010787 -13.613893 -0.342201
C 10.752358 -14.963812 -0.709194
C 10.003092 -15.825772 0.182538
C 9.712872 -11.91631 2.504638
H 8.862757 -15.921306 2.029343
H 11.550793 -12.94199 -0.999718
H 8.810187 -11.324895 2.263621
O 9.333264 -13.258205 2.774673
O 10.603412 -11.903567 1.405919
H 10.213686 -11.497566 3.387212
N 9.924348 -17.152525 -0.140384
S 8.511463 -17.904312 -0.196418
O 7.454825 -17.346959 0.670183
O 8.744652 -19.351315 -0.175841
C 13.189129 -13.278239 -4.230582
C 12.745773 -13.700042 -5.48103
C 12.668739 -13.838095 -3.069392
H 13.165714 -13.266127 -6.387215
H 13.081447 -13.53962 -2.10834
C 11.787349 -14.709579 -5.562338

C 11.678955 -14.839454 -3.126531
H 11.452618 -15.070195 -6.534627
C 11.277012 -15.279115 -4.404361
H 10.544287 -16.085795 -4.465274
H 13.974787 -12.527717 -4.153729
C 7.434057 -16.256396 -2.142295
C 7.253998 -15.843223 -3.457377
C 8.032397 -17.489209 -1.878485
H 6.794912 -14.874325 -3.660519
C 7.661711 -16.643388 -4.530866
C 8.4314 -18.307423 -2.934169
H 8.895238 -19.265915 -2.707424
C 8.239076 -17.885948 -4.24608
H 8.551777 -18.527797 -5.071783
H 7.122347 -15.629669 -1.307868
C 7.525248 -16.160606 -5.943882
H 6.577977 -15.631531 -6.10766
H 8.32834 -15.455745 -6.204671
H 7.57696 -16.987277 -6.662352
H 10.519331 -16.395384 -2.227655
H 12.192607 -17.310442 -0.652731
C 12.854569 -17.70221 -2.67282
C 13.768514 -17.454323 -3.661673
C 14.817175 -16.466641 -3.508911
C 14.867394 -15.782991 -2.194473
C 13.897436 -16.049957 -1.192298
C 13.974457 -15.342533 0.026936
C 14.97166 -14.406961 0.241591
C 15.933333 -14.155876 -0.749999
C 15.876332 -14.843127 -1.951007
H 12.068276 -18.445208 -2.827295
H 13.74065 -17.991514 -4.608862
O 15.63324 -16.20268 -4.41022
H 16.59456 -14.668551 -2.751522
H 16.718161 -13.420608 -0.572327
H 15.009446 -13.864917 1.18648
H 13.223659 -15.536918 0.793932

TS_A-B_para_anion_toluene_conf2

C 11.399232 -14.981893 -1.501845
C 13.605619 -14.916292 -1.001562
C 11.182591 -17.745167 -4.020612
C 11.20374 -18.819236 -3.162756
C 11.202414 -18.676936 -1.774608
C 11.219835 -17.448566 -1.17653
C 11.24152 -16.300702 -2.018818
C 11.232958 -16.446734 -3.459523
C 11.559561 -20.788824 -2.239221
H 11.160119 -17.858125 -5.099139
H 11.286647 -17.356218 -0.097466
H 11.01104 -21.732794 -2.157746
O 11.221815 -20.156785 -3.473546
O 11.177926 -19.927089 -1.188376
H 12.652507 -20.951845 -2.205154
N 11.308561 -15.321444 -4.244837
S 10.035455 -14.922799 -5.11228
O 9.303912 -16.058898 -5.707336
O 10.388049 -13.790265 -5.976696
C 9.328511 -14.540967 1.64788
C 9.643294 -13.228714 1.992098
C 9.891461 -15.134062 0.522852
H 9.201785 -12.7684 2.875138
H 9.592176 -16.140215 0.240468
C 10.510822 -12.501419 1.175053
C 10.79614 -14.431369 -0.292448
H 10.747215 -11.464959 1.41425
C 11.068142 -13.092444 0.05113
H 11.734064 -12.516317 -0.592875
H 8.623979 -15.10895 2.255134
C 8.161229 -15.140419 -3.083621
C 7.453966 -14.659763 -1.985101
C 8.938858 -14.267966 -3.844864
H 6.854508 -15.347238 -1.38479
C 7.508907 -13.312382 -1.619413
C 9.008521 -12.919417 -3.491075
H 9.630813 -12.255638 -4.089515

C 8.29593 -12.451043 -2.394392
H 8.357471 -11.39603 -2.118778
H 8.119756 -16.192514 -3.363026
C 6.750337 -12.798492 -0.432772
H 5.910088 -12.154068 -0.729635
H 6.337796 -13.619405 0.165994
H 7.392585 -12.201981 0.228087
H 11.562868 -14.247668 -2.293048
H 13.599418 -13.829655 -1.085434
C 13.597876 -15.500281 0.291864
C 13.903887 -16.818633 0.497243
C 14.321793 -17.689519 -0.583751
C 14.490005 -17.039267 -1.902214
C 14.177313 -15.669684 -2.089689
C 14.359835 -15.108612 -3.365558
C 14.842157 -15.869716 -4.415176
C 15.153169 -17.221928 -4.222579
C 14.972925 -17.796785 -2.976089
H 13.260085 -14.887773 1.130502
H 13.820626 -17.271701 1.484562
O 14.532357 -18.904794 -0.42471
H 15.192967 -18.846673 -2.787066
H 15.518882 -17.820061 -5.056761
H 14.949409 -15.419193 -5.40061
H 14.066432 -14.072739 -3.534868

TS_A-B_para_anion_toluene_conf3
C 11.205455 -15.705386 -2.005016
C 13.046447 -15.287151 -0.702075
C 10.447989 -12.219726 -3.160966
C 10.72238 -12.364786 -4.496698
C 11.21844 -13.548122 -5.041668
C 11.444099 -14.658643 -4.276966
C 11.153641 -14.580155 -2.88641
C 10.690772 -13.332621 -2.312484
C 10.852674 -12.12025 -6.701589
H 10.064968 -11.2966 -2.740718
H 11.771263 -15.585103 -4.735397

H 9.896114 -12.266645 -7.236582
O 10.607417 -11.420745 -5.489937
O 11.430575 -13.375017 -6.398188
H 11.546358 -11.537636 -7.32166
N 10.580988 -13.265937 -0.950568
S 9.226036 -12.7636 -0.260218
O 8.410389 -11.827915 -1.058593
O 9.503196 -12.433562 1.140755
C 12.550202 -18.921766 -3.506082
C 11.818918 -19.861127 -2.784368
C 12.363587 -17.561283 -3.289531
H 11.977543 -20.925563 -2.950547
H 12.995411 -16.850744 -3.81777
C 10.910109 -19.426666 -1.820126
C 11.430482 -17.09859 -2.340362
H 10.352365 -20.151046 -1.227098
C 10.733579 -18.069267 -1.592148
H 10.040548 -17.726995 -0.821472
H 13.300912 -19.248119 -4.224677
C 7.647103 -14.72947 -1.400416
C 7.122549 -16.014878 -1.468836
C 8.317144 -14.314405 -0.248638
H 6.608506 -16.340525 -2.374642
C 7.251105 -16.908211 -0.39892
C 8.442311 -15.183964 0.833467
H 8.967835 -14.840484 1.72291
C 7.905956 -16.465534 0.755756
H 8.004386 -17.14544 1.604038
H 7.55219 -14.037666 -2.236285
C 6.741268 -18.314124 -0.50737
H 5.769495 -18.360809 -1.014937
H 7.429974 -18.945188 -1.088046
H 6.628578 -18.782313 0.477687
H 10.600197 -15.557264 -1.11225
H 12.651266 -14.278298 -0.594932
C 12.802447 -16.225719 0.331206
C 13.430149 -17.441821 0.377686
C 14.444973 -17.81985 -0.584309

C 14.792131 -16.786915 -1.589309
C 14.114934 -15.539172 -1.621958
C 14.46436 -14.607672 -2.623624
C 15.443396 -14.903649 -3.556091
C 16.115465 -16.135535 -3.511412
C 15.78937 -17.060213 -2.533283
H 12.032467 -15.978358 1.06612
H 13.188107 -18.177336 1.143857
O 15.002621 -18.932122 -0.5767
H 16.2767 -18.032713 -2.471459
H 16.88805 -16.360223 -4.246787
H 15.693007 -14.174764 -4.327105
H 13.937536 -13.653206 -2.658488

TS_A-B_para_anion_toluene_conf4
C -13.744531 15.033775 0.314878
C -14.558127 17.042349 0.849574
C -11.70928 15.612023 -2.81082
C -12.65931 15.292413 -3.734535
C -13.943969 14.853237 -3.391627
C -14.335711 14.741022 -2.091524
C -13.406969 15.078606 -1.064668
C -12.048335 15.496784 -1.419901
C -13.730804 14.721863 -5.597188
H -10.72209 15.948763 -3.110222
H -15.326824 14.373974 -1.847015
H -13.476979 13.714801 -5.976073
O -12.554861 15.344845 -5.104258
O -14.670735 14.616559 -4.545907
H -14.15259 15.333903 -6.405021
N -11.192328 15.722532 -0.410192
S -9.617964 15.985922 -0.72634
O -8.956388 14.82558 -1.354367
O -9.365784 17.297346 -1.351681
C -17.119902 13.46786 1.157401
C -16.767804 12.663213 2.238701
C -16.161733 14.238276 0.507221
H -17.520648 12.062687 2.747405

H -16.47556 14.897557 -0.299917
C -15.443171 12.652399 2.671324
C -14.815002 14.225635 0.913345
H -15.15166 12.041331 3.525012
C -14.488166 13.426239 2.023573
H -13.456409 13.424486 2.37403
H -18.156066 13.508171 0.82244
C -7.926323 15.304095 1.33438
C -7.435795 15.411098 2.63494
C -9.022394 16.069251 0.95638
H -6.577555 14.808929 2.939078
C -8.028905 16.268945 3.563996
C -9.624184 16.937288 1.865718
H -10.483562 17.527124 1.560268
C -9.134485 17.030185 3.160138
H -9.633465 17.702304 3.858837
H -7.480713 14.625818 0.609645
C -7.500923 16.375605 4.964008
H -6.661655 15.690385 5.133621
H -8.274716 16.144839 5.708151
H -7.149015 17.391541 5.188877
H -12.874702 15.150922 0.962299
H -15.435203 16.794908 0.251117
C -14.655541 16.945943 2.265238
C -13.771319 17.571234 3.102151
C -12.730515 18.446265 2.607733
C -12.717853 18.66527 1.145867
C -13.630046 17.989766 0.295014
C -13.569117 18.232248 -1.090939
C -12.614748 19.083841 -1.620578
C -11.70944 19.739764 -0.778782
C -11.773033 19.53538 0.589452
H -15.42634 16.29423 2.682942
H -13.816421 17.427798 4.181117
O -11.890157 18.982311 3.358755
H -11.078071 20.021059 1.273736
H -10.94246 20.385863 -1.202382
H -12.557487 19.22712 -2.699377

H -14.267855 17.711747 -1.747946

TS_A-B_para_anion_toluene_conf5

C -12.798867 16.00663 1.232654
C -14.554118 17.351976 0.637718
C -14.531867 12.770554 1.994016
C -14.242028 12.669431 3.330227
C -13.546545 13.656075 4.028459
C -13.086583 14.788235 3.414504
C -13.345997 14.946435 2.026073
C -14.10999 13.944486 1.313349
C -14.183382 12.102651 5.477193
H -15.067251 11.99948 1.451235
H -12.479699 15.502556 3.96059
H -13.627011 11.319946 6.008266
O -14.537007 11.632297 4.185165
O -13.366948 13.250088 5.338809
H -15.102741 12.361612 6.034791
N -14.453341 14.212447 0.013977
S -14.100242 13.164062 -1.143789
O -14.156109 11.740596 -0.757781
O -14.787058 13.585729 -2.370188
C -10.825391 18.677403 3.074485
C -9.926626 19.050177 2.079039
C -11.769261 17.684726 2.836719
H -9.204786 19.844551 2.262322
H -12.508819 17.463632 3.602262
C -9.983638 18.423552 0.834486
C -11.822201 17.010716 1.599386
H -9.31187 18.730958 0.033559
C -10.920103 17.428324 0.597988
H -10.983701 16.955084 -0.383731
H -10.818642 19.191168 4.034802
C -11.421151 13.082783 -0.452782
C -10.110219 13.541128 -0.511558
C -12.357367 13.537935 -1.38341
H -9.384072 13.193528 0.224984
C -9.704796 14.454939 -1.491345

C -11.966627 14.430111 -2.378972
H -12.709412 14.770243 -3.098134
C -10.650265 14.877465 -2.431348
H -10.352474 15.586347 -3.206394
H -11.733294 12.378385 0.3173
C -8.3087 15.00115 -1.50121
H -7.568507 14.240884 -1.22195
H -8.199604 15.827095 -0.783366
H -8.032805 15.393962 -2.487084
H -12.778941 15.750443 0.174141
H -15.11198 16.43854 0.439925
C -14.775046 18.091901 1.821609
C -14.250956 19.345861 2.001809
C -13.488492 20.020905 0.968965
C -13.382931 19.303701 -0.322128
C -13.935834 18.00901 -0.481018
C -13.795185 17.351049 -1.720677
C -13.13579 17.968663 -2.769177
C -12.59695 19.256004 -2.611885
C -12.722548 19.91081 -1.398558
H -15.319926 17.609925 2.636682
H -14.37526 19.884045 2.941265
O -12.960051 21.133509 1.145284
H -12.306692 20.903924 -1.232291
H -12.078073 19.733569 -3.443236
H -13.034953 17.450121 -3.72336
H -14.207381 16.344545 -1.827008
TS_A-B_para_anion_toluene_conf6
C -12.961452 15.885678 0.811924
C -14.587595 17.324785 0.805066
C -10.526979 17.437818 3.206372
C -9.899389 18.306045 2.34923
C -10.184781 18.353708 0.982525
C -11.125254 17.544791 0.413114
C -11.840919 16.652082 1.261218
C -11.560945 16.622244 2.681455
C -8.966267 20.103036 1.494747
H -10.309534 17.404245 4.268405

H -11.390845 17.66469 -0.632928
H -7.949036 20.45783 1.298964
O -8.951679 19.254382 2.639398
O -9.418605 19.337594 0.391168
H -9.670676 20.932413 1.675784
N -12.358251 15.858973 3.497707
S -11.721265 14.597903 4.233663
O -10.323509 14.772003 4.674107
O -12.695894 14.063938 5.191982
C -12.421767 14.635961 -2.74554
C -13.590614 13.906871 -2.946741
C -12.188335 15.286571 -1.537115
H -13.770897 13.401636 -3.894829
H -11.245301 15.804367 -1.387698
C -14.515452 13.808398 -1.905754
C -13.125814 15.237846 -0.491531
H -15.420219 13.214336 -2.031635
C -14.280322 14.45791 -0.703116
H -14.996888 14.362713 0.113888
H -11.673809 14.695361 -3.535771
C -10.617162 13.509813 1.93973
C -10.675559 12.764589 0.768101
C -11.653238 13.423587 2.872806
H -9.872752 12.850559 0.033011
C -11.761098 11.923528 0.497148
C -12.734658 12.580143 2.625679
H -13.535608 12.530179 3.36161
C -12.778886 11.834689 1.451501
H -13.630903 11.180025 1.256119
H -9.776943 14.17287 2.142312
C -11.841404 11.170265 -0.796434
H -10.960657 10.533961 -0.960069
H -11.903401 11.856957 -1.652247
H -12.728468 10.526222 -0.832128
H -13.420764 15.327454 1.629118
H -15.336995 16.590041 0.508665
C -14.543929 17.741417 2.167301
C -13.785378 18.809255 2.556794

C -13.092558 19.649039 1.603622
C -13.399558 19.393742 0.177339
C -14.156306 18.260021 -0.207814
C -14.370175 18.027589 -1.580082
C -13.861641 18.893597 -2.534211
C -13.118255 20.017276 -2.145185
C -12.88884 20.255849 -0.800512
H -14.991971 17.098922 2.924313
H -13.631761 19.033482 3.610997
O -12.283108 20.53362 1.941251
H -12.289083 21.098296 -0.458055
H -12.715023 20.691391 -2.900558
H -14.037017 18.69542 -3.59157
H -14.930159 17.141661 -1.884306

TS_A-B_para_anion_toluene_conf7
C -12.606047 16.996957 1.268246
C -14.712295 17.873129 1.88212
C -9.926572 19.550645 1.841437
C -9.644673 19.864059 0.539636
C -10.348672 19.328768 -0.542339
C -11.361559 18.430367 -0.369348
C -11.6969 18.046638 0.960493
C -11.006564 18.654644 2.089232
C -8.702174 20.587086 -1.340337
H -9.376368 19.970972 2.67512
H -11.849014 17.980697 -1.22812
H -7.796866 20.031408 -1.647192
O -8.698208 20.744245 0.069766
O -9.858841 19.866458 -1.719721
H -8.715526 21.576982 -1.815138
N -11.455758 18.352759 3.331436
S -10.467477 17.929473 4.521058
O -9.058539 18.346835 4.370548
O -11.136474 18.162594 5.801894
C -14.163771 15.253205 -1.729844
C -14.129696 13.926515 -1.300733
C -13.675232 16.270183 -0.919192

H -14.514514 13.131825 -1.93839
H -13.767982 17.305288 -1.24499
C -13.61845 13.634191 -0.037465
C -13.125068 15.992826 0.347487
H -13.60401 12.604806 0.320609
C -13.130695 14.650642 0.775414
H -12.734383 14.422216 1.766168
H -14.59006 15.500005 -2.701929
C -9.527529 15.558086 3.427137
C -9.651485 14.215847 3.083633
C -10.480814 16.151437 4.253627
H -8.906235 13.753361 2.433766
C -10.723412 13.446082 3.5487
C -11.55285 15.400576 4.733958
H -12.298396 15.884521 5.36373
C -11.665131 14.059684 4.383538
H -12.511163 13.476876 4.753618
H -8.699354 16.162365 3.059998
C -10.883003 12.016754 3.122905
H -11.589871 11.478944 3.765933
H -9.930226 11.472078 3.143323
H -11.262993 11.943827 2.093088
H -12.514921 16.652377 2.297238
H -15.150977 17.256745 1.100349
C -14.514828 19.254173 1.638682
C -14.285168 20.146287 2.647532
C -14.31408 19.750501 4.044374
C -14.602811 18.321988 4.307333
C -14.83135 17.417488 3.239886
C -15.146828 16.078316 3.546664
C -15.220742 15.649783 4.861957
C -14.971926 16.54494 5.911764
C -14.663679 17.867301 5.627088
H -14.491569 19.596114 0.600769
H -14.080613 21.196171 2.44397
O -14.145962 20.562564 4.966827
H -14.448442 18.590897 6.411357
H -15.013198 16.200032 6.944677

H -15.466316 14.609358 5.079308
H -15.325239 15.379189 2.727015

TS_A-B_para_anion_toluene_conf8
C -12.549622 16.740489 1.612779
C -14.680853 16.975431 2.427064
C -11.643475 13.448166 3.163464
C -10.659669 13.870385 4.020491
C -10.314616 15.212554 4.170969
C -10.924769 16.201882 3.451459
C -11.937158 15.825227 2.525475
C -12.331603 14.43593 2.409778
C -8.927939 13.997574 5.407744
H -11.910733 12.403546 3.051132
H -10.59616 17.231002 3.542713
H -7.947361 13.7904 4.940742
O -9.896945 13.107166 4.872796
O -9.322184 15.326859 5.128436
H -8.865904 13.854271 6.494414
N -13.414645 14.14833 1.624791
S -13.319084 13.037386 0.475157
O -12.334894 11.962203 0.706401
O -14.675077 12.669229 0.05786
C -11.609722 20.391231 2.053859
C -11.689939 20.832925 0.736303
C -11.864546 19.06174 2.371404
H -11.500668 21.877842 0.495243
H -11.85719 18.757543 3.415441
C -12.051133 19.933041 -0.266072
C -12.206783 18.129435 1.3719
H -12.144402 20.270879 -1.297948
C -12.320657 18.60946 0.051186
H -12.626959 17.908931 -0.727923
H -11.379234 21.094391 2.853152
C -11.263543 14.301094 -0.878753
C -10.756927 15.24501 -1.764269
C -12.63587 14.0481 -0.844736
H -9.684993 15.44862 -1.781003

C -11.599284 15.951023 -2.63114
C -13.489577 14.730908 -1.709188
H -14.556648 14.519186 -1.666614
C -12.96906 15.667396 -2.597242
H -13.638452 16.201028 -3.274505
H -10.607707 13.757211 -0.200268
C -11.051637 17.013212 -3.536795
H -11.755432 17.265043 -4.338937
H -10.106065 16.708413 -4.00297
H -10.84475 17.941641 -2.984617
H -12.986293 16.236071 0.752331
H -14.700513 15.887563 2.473811
C -15.278009 17.62082 1.315877
C -15.483813 18.974085 1.27975
C -15.173982 19.829409 2.407136
C -14.666371 19.137623 3.615877
C -14.450861 17.733912 3.620187
C -13.934638 17.133932 4.789097
C -13.639798 17.896128 5.906047
C -13.863494 19.282026 5.897351
C -14.374798 19.88783 4.761614
H -15.510444 17.008512 0.441012
H -15.88826 19.463674 0.394718
O -15.324019 21.06424 2.379436
H -14.551484 20.96168 4.709295
H -13.632488 19.875594 6.781917
H -13.233039 17.415647 6.795821
H -13.758137 16.057595 4.791671

TS_A-B_para_anion_toluene_conf9

C -12.668008 16.595206 1.519119
C -14.799725 17.315274 2.257574
C -11.193769 19.096654 -0.846834
C -11.31587 18.389555 -2.011838
C -11.936967 17.139438 -2.082619
C -12.447761 16.52477 -0.976366
C -12.339654 17.198037 0.274122
C -11.753543 18.530146 0.33488

C -11.126669 17.626093 -4.092444
H -10.712457 20.066503 -0.800457
H -12.866945 15.526398 -1.047039
H -10.152128 17.161556 -4.330801
O -10.91337 18.764172 -3.272165
O -11.940649 16.702619 -3.395643
H -11.632433 17.93547 -5.016621
N -11.80023 19.178857 1.522755
S -10.522708 19.915273 2.152565
O -9.448369 20.270857 1.203374
O -10.977634 20.92455 3.110062
C -13.904239 13.004751 1.319271
C -13.237662 12.44363 2.40861
C -13.738746 14.348081 1.005398
H -13.369352 11.389908 2.650793
H -14.313072 14.781511 0.18763
C -12.41957 13.253258 3.19469
C -12.892719 15.176107 1.768548
H -11.903367 12.836101 4.059558
C -12.257517 14.598391 2.88505
H -11.620282 15.232265 3.50306
H -14.572025 12.39105 0.715099
C -8.944218 17.671651 2.571237
C -8.611874 16.499075 3.242449
C -9.886355 18.539888 3.120622
H -7.886866 15.811612 2.802766
C -9.209311 16.171308 4.46416
C -10.488364 18.235855 4.341496
H -11.24594 18.908668 4.742381
C -10.145635 17.062456 5.002941
H -10.635594 16.815526 5.947125
H -8.4954 17.920562 1.610827
C -8.872465 14.88927 5.166563
H -9.654496 14.607162 5.882623
H -7.930834 14.958979 5.730363
H -8.755668 14.059037 4.457769
H -12.257976 17.137881 2.369566
H -15.081806 16.266629 2.322076

C -15.227893 18.075631 1.142807
C -15.200297 19.441856 1.138199
C -14.810053 20.20611 2.309084
C -14.440728 19.414839 3.505321
C -14.472147 17.997578 3.478846
C -14.155641 17.294161 4.658965
C -13.813138 17.970341 5.818416
C -13.763113 19.371433 5.830916
C -14.072417 20.078845 4.678274
H -15.535653 17.539832 0.241218
H -15.480636 20.014113 0.255339
O -14.819389 21.446571 2.325147
H -14.031232 21.165989 4.640523
H -13.474751 19.899345 6.739574
H -13.573178 17.408323 6.722095
H -14.178665 16.202493 4.642378

TS_A-B_para_anion_toluene_conf10
C -12.276225 16.581429 0.940953
C -14.624519 17.07548 0.518188
C -12.672612 12.95411 1.804031
C -12.211804 12.97456 3.097863
C -11.793498 14.148556 3.719755
C -11.814067 15.356145 3.077765
C -12.28382 15.394348 1.735119
C -12.726455 14.17881 1.092557
C -11.692636 12.503338 5.205397
H -13.008488 12.038132 1.324841
H -11.419563 16.244396 3.560282
H -10.841471 11.95809 5.632806
O -12.047127 11.916809 3.960032
O -11.328406 13.852799 4.988472
H -12.559613 12.453452 5.889699
N -13.32112 14.223417 -0.147499
S -12.510247 13.918003 -1.472546
O -13.421686 14.089624 -2.612489
O -11.192669 14.584226 -1.52487
C -11.660758 19.828118 2.792032

C -11.139609 20.604482 1.760948
C -12.029142 18.507925 2.563437
H -10.876908 21.645927 1.939313
H -12.504155 17.94365 3.361789
C -10.992452 20.048763 0.489135
C -11.861025 17.91308 1.292668
H -10.619616 20.656019 -0.33459
C -11.357295 18.732784 0.257843
H -11.272694 18.305437 -0.741623
H -11.825349 20.267556 3.774819
C -13.110268 11.230683 -1.755249
C -12.860825 9.873322 -1.604866
C -12.123717 12.160956 -1.422655
H -13.632237 9.148478 -1.872063
C -11.63093 9.413057 -1.114314
C -10.894538 11.722501 -0.938345
H -10.135124 12.461302 -0.689145
C -10.65653 10.358181 -0.786897
H -9.692775 10.016499 -0.405274
H -14.064579 11.591627 -2.135285
C -11.371833 7.944207 -0.95597
H -11.388892 7.419417 -1.92131
H -12.130429 7.461143 -0.325557
H -10.39389 7.75501 -0.498315
H -12.282434 16.379595 -0.127922
H -14.694474 16.022142 0.250425
C -15.000766 17.529003 1.795625
C -15.03745 18.867097 2.109256
C -14.736873 19.895779 1.135118
C -14.457741 19.416991 -0.236562
C -14.413486 18.029141 -0.528227
C -14.089246 17.614787 -1.838764
C -13.845756 18.551607 -2.827376
C -13.914801 19.925232 -2.54046
C -14.214319 20.346329 -1.25633
H -15.207189 16.785737 2.569123
H -15.281105 19.208187 3.115468
O -14.707917 21.107439 1.424983

H -14.252537 21.401469 -0.987456
H -13.7212 20.655102 -3.326967
H -13.595297 18.216652 -3.833579
H -14.022624 16.545756 -2.055556

TS_A-B_para_anion_toluene_conf11
C -12.242783 16.511837 0.972283
C -14.620235 17.030037 0.557876
C -12.659299 12.870804 1.760562
C -12.192465 12.86168 3.052291
C -11.764988 14.020232 3.697218
C -11.781642 15.241082 3.081248
C -12.25692 15.310261 1.74133
C -12.70994 14.110583 1.075244
C -11.664602 12.342611 5.146594
H -13.002936 11.966842 1.26437
H -11.379409 16.116555 3.580414
H -10.813189 11.784385 5.556409
O -12.029678 11.785159 3.890971
O -11.295652 13.694935 4.956931
H -12.527385 12.281487 5.835162
N -13.310808 14.185943 -0.159558
S -12.513513 13.897899 -1.496742
O -13.432715 14.098398 -2.625513
O -11.191145 14.554445 -1.546581
C -11.634969 19.719031 2.891419
C -11.12302 20.520559 1.874958
C -11.998375 18.402702 2.635663
H -10.865192 21.559276 2.074787
H -12.467375 17.81872 3.423368
C -10.979786 19.993826 0.590306
C -11.833993 17.836444 1.350773
H -10.615095 20.621353 -0.221782
C -11.339979 18.681929 0.331549
H -11.259807 18.277789 -0.677886
H -11.797684 20.136385 3.884084
C -13.137123 11.221269 -1.826595
C -12.897151 9.859353 -1.70455

C -12.140931 12.137249 -1.483279
H -13.676074 9.145839 -1.980313
C -11.667355 9.380166 -1.232219
C -10.911679 11.679981 -1.016859
H -10.144597 12.407852 -0.759165
C -10.683191 10.311192 -0.893837
H -9.719282 9.954711 -0.526437
H -14.091241 11.596846 -2.192665
C -11.418896 7.906547 -1.104071
H -11.456747 7.399225 -2.078077
H -12.171308 7.419006 -0.469657
H -10.435352 7.700706 -0.666052
H -12.261804 16.334047 -0.100769
H -14.68973 15.982352 0.268758
C -14.981714 17.456233 1.847545
C -15.000195 18.787563 2.193785
C -14.691734 19.83515 1.243457
C -14.426958 19.386007 -0.140855
C -14.398297 18.004588 -0.465215
C -14.080845 17.61799 -1.786258
C -13.831276 18.575104 -2.753436
C -13.887438 19.942438 -2.434534
C -14.178295 20.336315 -1.139874
H -15.192462 16.697274 2.604457
H -15.233408 19.106871 3.209588
O -14.642703 21.039316 1.562474
H -14.204637 21.385251 -0.846323
H -13.689911 20.68856 -3.204672
H -13.586226 18.261484 -3.767825
H -14.024317 16.553728 -2.027719

TS_A-B_para_anion_toluene_conf12
C -12.781192 16.243804 0.401415
C -14.631563 17.28304 0.806776
C -10.450256 18.150643 2.63508
C -10.197757 19.229132 1.824724
C -10.662061 19.294153 0.511948
C -11.435648 18.307056 -0.029906

C -11.766318 17.18243 0.780353
C -11.259286 17.100694 2.130422
C -9.872602 21.265962 1.066692
H -10.092606 18.09222 3.659044
H -11.880611 18.444745 -1.010655
H -9.009473 21.883507 0.795996
O -9.494804 20.371297 2.110653
O -10.264438 20.489459 -0.053127
H -10.729487 21.869098 1.409394
N -11.613139 16.097073 3.00341
S -11.048888 14.624241 2.810715
O -11.700619 13.740906 3.785933
O -10.961601 14.162449 1.407902
C -12.682994 15.541253 -3.341961
C -13.696815 14.595181 -3.454934
C -12.355121 16.087845 -2.104392
H -13.952391 14.172364 -4.425745
H -11.526446 16.786128 -2.039147
C -14.363943 14.175259 -2.303304
C -13.040883 15.70964 -0.939027
H -15.137951 13.411071 -2.366456
C -14.034265 14.7201 -1.071541
H -14.546093 14.376242 -0.172182
H -12.129789 15.855639 -4.226434
C -8.972397 14.477998 4.647215
C -7.656906 14.668713 5.060262
C -9.332994 14.759851 3.332477
H -7.373257 14.437263 6.088875
C -6.684113 15.15165 4.180553
C -8.374291 15.238462 2.437552
H -8.666433 15.441467 1.40824
C -7.066062 15.432374 2.863003
H -6.319566 15.805633 2.159421
H -9.733146 14.10039 5.327799
C -5.270108 15.369611 4.631661
H -4.5494 14.841044 3.993035
H -5.117403 15.021002 5.659982
H -4.991224 16.432129 4.602392

H -12.997502 15.521647 1.183665
H -15.246932 16.429547 0.52103
C -14.455923 17.55593 2.195158
C -13.90477 18.730825 2.621851
C -13.591217 19.807933 1.708627
C -14.063575 19.63355 0.316531
C -14.596055 18.393955 -0.115137
C -14.978532 18.257515 -1.463548
C -14.847518 19.315132 -2.348361
C -14.326656 20.542078 -1.912492
C -13.935978 20.691102 -0.592368
H -14.617721 16.748659 2.909234
H -13.633699 18.878398 3.665932
O -12.958282 20.824792 2.053664
H -13.499804 21.618251 -0.222493
H -14.221307 21.369095 -2.614325
H -15.147345 19.188863 -3.388669
H -15.366083 17.297137 -1.807368

TS_A-B_para_anion_toluene_conf13
C 0.8024622182 -0.6909744589 0.9870731786
C 1.1437384376 -1.8868242659 -1.0461773946
C -1.3208637226 1.9700744865 -0.5920069384
C -0.4878316205 3.0517626115 -0.4343093398
C 0.7634162785 2.9429773489 0.1643662127
C 1.2428427149 1.7496315575 0.6308753968
C 0.4194881151 0.5960690698 0.4991441801
C -0.8802300667 0.7080024784 -0.1224321613
C 0.3833114652 5.0885054338 -0.2651391206
H -2.297388764 2.0533603875 -1.0620780251
H 2.2019750621 1.7016827498 1.1347197923
H 0.0153293927 5.6832938263 0.5908428873
O -0.6997057162 4.352962799 -0.8207634706
O 1.3779503759 4.1822428652 0.1701034532
H 0.8017160137 5.7514657439 -1.0337257998
N -1.6425304 -0.4054715582 -0.3929429973
S -2.7183312557 -0.9459596152 0.6367201328
O -3.3634768855 -2.1289264922 0.0563771784

O -2.2264154167 -1.0336834836 2.0290233491
C 4.4157851884 -1.0012997471 2.0871750929
C 4.3003532622 -2.0036086129 3.0462267222
C 3.2936140992 -0.5361138365 1.4120474644
H 5.1848830738 -2.3722303753 3.5633913128
H 3.4199615202 0.1982081634 0.6199470731
C 3.0479297176 -2.5579060956 3.3111589023
C 2.0129366227 -1.0631926589 1.6818764883
H 2.9480611986 -3.3599059638 4.0414001841
C 1.9288334763 -2.1075409309 2.6271144025
H 0.9506421475 -2.5490375599 2.8195785966
H 5.3952209007 -0.5986861569 1.8320315098
C -4.977699517 0.3704966938 -0.2813532198
C -5.8790980434 1.4264199436 -0.3120619208
C -3.9823825215 0.3334219791 0.6964936514
H -6.6611572898 1.4479662845 -1.0736025055
C -5.8043864813 2.4700579355 0.6205434094
C -3.8958633235 1.3588240174 1.6343882375
H -3.1183182908 1.3117013318 2.3947206649
C -4.8016481365 2.4159696995 1.5910509261
H -4.731414684 3.2191769145 2.3267469598
H -5.0312325131 -0.4415984101 -1.0045511278
C -6.7809703402 3.607637291 0.5755287634
H -6.56809823 4.3518680268 1.3517207443
H -7.8147901345 3.2658100163 0.7229337037
H -6.7597355732 4.1263404389 -0.392505933
H -0.0408526543 -1.3528272416 1.1617540991
H 0.1649425039 -1.4983603812 -1.3252883246
C 1.2378102896 -3.1878022966 -0.5026954244
C 2.4448482545 -3.7981097454 -0.2769122138
C 3.7009214127 -3.1795507249 -0.6456437407
C 3.5938512367 -1.8614787178 -1.3142736379
C 2.3297507127 -1.2447177262 -1.5178847156
C 2.2917768581 0.032058731 -2.120844667
C 3.4581042325 0.6713540425 -2.5019172327
C 4.7024290253 0.0494411816 -2.3096957066
C 4.7601892765 -1.2047934413 -1.7250533528
H 0.3102837579 -3.680329351 -0.2029862856

H 2.5050806352 -4.7799965538 0.1908879561
O 4.8058475616 -3.7098833408 -0.4266546873
H 5.7056952295 -1.7152453537 -1.544189837
H 5.6169515696 0.5565273313 -2.6174230495
H 3.4080996647 1.6618936816 -2.954247414
H 1.3236820791 0.513022034 -2.267346382

TS_A-B_para_anion_toluene_conf14
C 0.844418 -0.220343 0.650545
C 2.499653 0.035233 -1.004008
C 2.80886 1.554054 3.312881
C 3.022361 0.507337 4.177877
C 2.555763 -0.776654 3.91651
C 1.856898 -1.077167 2.778814
C 1.600956 -0.02997 1.850805
C 2.085051 1.303584 2.121097
C 3.464065 -0.7672 5.942222
H 3.176654 2.55711 3.513439
H 1.463831 -2.07587 2.623463
H 2.744027 -0.665692 6.775029
O 3.701315 0.514087 5.37282
O 2.932189 -1.619778 4.946977
H 4.41213 -1.180144 6.310555
N 1.979056 2.30483 1.181992
S 0.737912 3.287745 1.151875
O 0.904199 4.209218 0.022111
O -0.567667 2.611938 1.314528
C 0.08599 -3.826434 -0.23941
C -1.09295 -3.663056 -0.961776
C 0.732145 -2.729375 0.316771
H -1.586768 -4.524996 -1.407723
H 1.689933 -2.875712 0.810891
C -1.614688 -2.382401 -1.141389
C 0.2094 -1.426676 0.171445
H -2.522045 -2.238136 -1.726339
C -0.96413 -1.283964 -0.599061
H -1.359773 -0.27952 -0.751428
H 0.532765 -4.814724 -0.13841

C 1.755204 5.396776 2.631497
C 1.980955 6.102938 3.805499
C 0.904095 4.290448 2.637006
H 2.640729 6.972767 3.795322
C 1.374072 5.719618 5.009371
C 0.288982 3.89737 3.822658
H -0.377588 3.03695 3.808649
C 0.526619 4.609742 4.995895
H 0.043466 4.299672 5.924214
H 2.224527 5.691301 1.69435
C 1.627902 6.487397 6.272723
H 1.093815 6.049515 7.124039
H 1.306994 7.534729 6.186851
H 2.695586 6.509164 6.530205
H 0.403779 0.698443 0.274257
H 2.802395 0.924335 -0.45261
C 1.643672 0.184649 -2.119947
C 1.351573 -0.860028 -2.957598
C 1.95732 -2.166129 -2.798073
C 2.953719 -2.282782 -1.707004
C 3.220753 -1.188842 -0.841238
C 4.143309 -1.371612 0.212184
C 4.776206 -2.588242 0.396761
C 4.519468 -3.659941 -0.47356
C 3.618329 -3.499717 -1.512845
H 1.159884 1.153978 -2.258818
H 0.646057 -0.748486 -3.780017
O 1.674674 -3.135464 -3.525843
H 3.376645 -4.311062 -2.198783
H 5.025915 -4.613758 -0.325427
H 5.478174 -2.712534 1.221369
H 4.338215 -0.53794 0.888311

TS_A-B_para_anion_toluene_conf15

C 1.018228 0.352371 0.027134
C 2.842938 -0.431034 -0.792346
C -0.934421 -2.833388 0.399982
C -1.456698 -2.902615 -0.860995

C -1.372694 -1.837006 -1.763427
C -0.671139 -0.708997 -1.4684
C 0.0049 -0.626468 -0.211244
C -0.217141 -1.664675 0.783228
C -2.198921 -3.575066 -2.815493
H -1.037286 -3.63901 1.120296
H -0.580065 0.087233 -2.198743
H -3.162914 -3.873609 -3.241436
O -2.178566 -3.917064 -1.430945
O -2.055086 -2.169901 -2.919574
H -1.34792 -4.069158 -3.311073
N 0.241072 -1.68444 2.061787
S 0.439422 -0.428366 3.02356
O 1.85644 -0.029255 3.200421
O -0.512819 0.683083 2.819744
C 0.809959 3.55399 -2.059979
C 0.991667 4.50816 -1.061769
C 0.816768 2.197145 -1.751595
H 0.985977 5.569693 -1.307298
H 0.713591 1.473687 -2.55631
C 1.188874 4.087094 0.251759
C 1.001567 1.754287 -0.4315
H 1.33492 4.819578 1.045134
C 1.199113 2.732161 0.560991
H 1.33345 2.400522 1.590597
H 0.668538 3.866262 -3.094564
C 0.643561 -2.383881 4.966804
C 0.342125 -2.952338 6.195423
C 0.008064 -1.203075 4.57806
H 0.834987 -3.878386 6.497065
C -0.590211 -2.360699 7.060228
C -0.918558 -0.599517 5.418288
H -1.40338 0.317552 5.08998
C -1.21223 -1.180855 6.651236
H -1.942965 -0.709013 7.310559
H 1.354818 -2.850011 4.286715
C -0.90968 -2.988545 8.384453
H -1.633438 -2.389743 8.949758

H -0.014117 -3.10086 9.01077
H -1.335852 -3.994524 8.267926
H 1.469046 0.28673 1.012072
H 3.461731 0.385436 -0.416424
C 2.557069 -0.47073 -2.186158
C 1.966168 -1.563049 -2.758013
C 1.69986 -2.768948 -2.002174
C 2.325917 -2.839293 -0.666457
C 2.933908 -1.696 -0.095037
C 3.54232 -1.808681 1.167593
C 3.560191 -3.01764 1.836807
C 2.942113 -4.145402 1.275037
C 2.329141 -4.051304 0.03885
H 2.717004 0.433663 -2.774846
H 1.627122 -1.553268 -3.794039
O 1.003743 -3.700221 -2.456685
H 1.824656 -4.900122 -0.420941
H 2.94055 -5.0913 1.817167
H 4.030686 -3.083647 2.81784
H 3.966171 -0.918936 1.631122

Table S5. Computed energies for the neutral transition state conformational analysis in acetonitrile.

	M06-L/BS1							TPSS0-D4/BS2
	1st frequency (cm-1)	Electronic energy	ZPE corr	Thermal corr	Enthalpy corr:	Gibbs corr	Electronic energy in acetonitrile	Electronic energy
pre-ortho TS								
TS_A-B_ortho_neutral_acetonitrile_conf2	-294.6	-2052.90302	0.47897	0.51249	0.51343	0.41197	-2052.94622	-2625.34095
TS_A-B_ortho_neutral_acetonitrile_conf23	-341.7	-2052.91355	0.47920	0.51281	0.51375	0.40977	-2052.95313	-2625.35381
TS_A-B_ortho_neutral_acetonitrile_conf4	-262.2	-2052.90406	0.47878	0.51226	0.51320	0.41229	-2052.94849	-2625.34267
TS_A-B_ortho_neutral_acetonitrile_conf5	-261.3	-2052.90409	0.47863	0.51217	0.51311	0.41156	-2052.94849	-2625.34257
TS_A-B_ortho_neutral_acetonitrile_conf6	-342.2	-2052.91359	0.47928	0.51285	0.51379	0.40972	-2052.95317	-2625.35385
TS_A-B_ortho_neutral_acetonitrile_conf7	-343.6	-2052.91357	0.47936	0.51293	0.51387	0.41033	-2052.95315	-2625.35379
pre-para TS								
TS_A-B_para_neutral_acetonitrile_conf2	-190.9	-2052.90604	0.47916	0.51259	0.51353	0.41193	-2052.94479	-2625.34450
TS_A-B_para_neutral_acetonitrile_conf3	-250.2	-2052.90234	0.47913	0.51258	0.51353	0.41181	-2052.94225	-2625.34163
TS_A-B_para_neutral_acetonitrile_conf4	-141.4	-2052.90126	0.47879	0.51250	0.51344	0.40937	-2052.94104	-2625.34002
TS_A-B_para_neutral_acetonitrile_conf5	-141.4	-2052.90126	0.47879	0.51250	0.51344	0.40937	-2052.94104	-2625.34002
TS_A-B_para_neutral_acetonitrile_conf6	-261.6	-2052.89763	0.47876	0.51261	0.51355	0.40826	-2052.93761	-2625.33890

Table S6. Computed energies for the neutral path in acetonitrile.

	M06-L/BS1							TPSS0-D4/BS2
	1st frequency (cm-1)	Electronic energy	ZPE corr	Thermal corr	Enthalpy corr:	Gibbs corr	Electronic energy in acetonitrile	Electronic energy
Path for ortho product								
InterA_pre-ortho_neutral_acetonitrile	15.6	-2052.91780	0.47848	0.51213	0.51308	0.40918	-2052.95811	- 2625.35506
TS_A-B_ortho_neutral_acetonitrile	-341.5	-2052.91356	0.47902	0.51270	0.51364	0.40867	-2052.95317	- 2625.35378
InterB_ortho_neutral_acetonitrile	9.2	-2052.92805	0.48163	0.51520	0.51614	0.41131	-2052.97162	- 2625.37286
Path for para product								
InterA_pre-para_neutral_acetonitrile	19.7	-2052.90677	0.47904	0.51329	0.51423	0.41069	-2052.94690	- 2625.34384
TS_A-B_para_neutral_acetonitrile	-197.4	-2052.90607	0.47895	0.51245	0.51340	0.41140	-2052.94495	- 2625.34463

InterB_para_neutral_acetonitrile	21.2	-2052.92847	0.48180	0.51508	0.51602	0.41522	-2052.96939	-<br>2625.37454

InterB_ortho_neutral_acetonitrile

C -1.199022 -0.4458 0.299955
C -1.845685 -0.421577 -1.171184
C -3.783908 1.021714 -1.737087
C 1.986271 1.586997 -0.230323
C 1.680892 2.684455 0.537202
C 0.503144 2.786968 1.258972
C -0.438468 1.787885 1.223024
C -0.175353 0.64425 0.444637
C 1.051937 0.517145 -0.275444
C 1.623735 4.685322 1.500353
H 2.906482 1.558285 -0.803451
H -1.348856 1.868007 1.808482
H 2.191199 5.076936 2.353148
O 2.45 3.799667 0.752322
O 0.494672 3.965847 1.973453
H 1.291782 5.511494 0.847596
N 1.26313 -0.677314 -0.966461
S 2.674447 -0.930177 -1.697335
O 3.093832 0.076444 -2.682177
O 2.608873 -2.332483 -2.184653
C -4.339686 0.168253 2.4183
C -4.417635 -1.02437 3.131915
C -3.287195 0.394942 1.536093
H -5.243955 -1.201073 3.818078
H -3.258257 1.324157 0.972496
C -3.42892 -1.988586 2.959147
C -2.28795 -0.56892 1.341385
H -3.475419 -2.926548 3.510111
C -2.38198 -1.759063 2.071948
H -1.622169 -2.527704 1.922897
H -5.107634 0.930013 2.542786
C 5.211818 -0.457183 -0.720466
C 6.20715 -0.50454 0.251542
C 3.937195 -0.92605 -0.420272

H 7.205755 -0.135252 0.017138
C 5.949306 -1.008409 1.528655
C 3.656562 -1.429229 0.849464
H 2.645231 -1.760939 1.083332
C 4.655933 -1.465812 1.812212
H 4.43119 -1.847635 2.808648
H 5.406996 -0.047756 -1.709373
C 7.019525 -1.050125 2.57703
H 7.969369 -0.655337 2.201251
H 6.74244 -0.460826 3.460515
H 7.202119 -2.073886 2.928297
H -0.656388 -1.402549 0.301669
H -4.204792 1.99569 -1.987072
C -2.464121 0.880862 -1.521743
C -2.755175 -1.617523 -1.275932
C -4.199485 -1.391098 -1.338763
C -4.700427 -0.089471 -1.587586
C -6.088511 0.088337 -1.660109
C -6.952973 -0.981536 -1.470991
C -6.452503 -2.261806 -1.213203
C -5.082046 -2.462025 -1.158096
H -1.794682 1.738811 -1.580441
H -0.962173 -0.58147 -1.812292
O -2.276904 -2.763594 -1.23904
H -4.659021 -3.445249 -0.962162
H -7.135991 -3.093937 -1.057614
H -8.028523 -0.820453 -1.5226
H -6.484793 1.083727 -1.85889
K 0.280363 -3.227529 -1.630351

TS_A-B_ortho_neutral_acetonitrile

C -1.044228 -0.415091 0.366015
C -1.956588 -0.359389 -1.605892
C -3.942312 1.045831 -1.553983
C 2.057673 1.589355 -0.407257
C 1.777227 2.699633 0.345002
C 0.626901 2.816303 1.125184
C -0.306946 1.824312 1.158068

C -0.085363 0.647143 0.387011
C 1.13179 0.509156 -0.390338
C 1.795601 4.699275 1.327118
H 2.949225 1.555472 -1.022769
H -1.172594 1.907191 1.803998
H 2.424398 5.003557 2.173347
O 2.539732 3.818854 0.485431
O 0.65226 4.010326 1.805605
H 1.485685 5.578311 0.741088
N 1.32606 -0.675506 -1.06464
S 2.783442 -0.987132 -1.711294
O 3.266245 -0.018023 -2.704644
O 2.700841 -2.397814 -2.157907
C -4.257501 0.245335 2.289595
C -4.514602 -1.025082 2.795681
C -3.123625 0.484349 1.520426
H -5.406562 -1.207502 3.392248
H -2.977078 1.46802 1.084685
C -3.63088 -2.066584 2.516495
C -2.215213 -0.551403 1.235894
H -3.828541 -3.068245 2.894295
C -2.504655 -1.832754 1.740902
H -1.832212 -2.654504 1.495433
H -4.956091 1.058912 2.478255
C 5.199697 -0.364043 -0.538488
C 6.123211 -0.379092 0.503125
C 3.955652 -0.957588 -0.356825
H 7.098164 0.087966 0.363728
C 5.821534 -0.975728 1.729443
C 3.630274 -1.557464 0.859586
H 2.64348 -1.997596 0.997723
C 4.558249 -1.56078 1.890995
H 4.301123 -2.020765 2.84542
H 5.427282 0.111211 -1.490358
C 6.816992 -0.992528 2.849307
H 7.717371 -0.42166 2.599343
H 6.397459 -0.568053 3.769935
H 7.132383 -2.015711 3.091669

H -0.619103 -1.380208 0.084559
H -4.417408 2.025209 -1.608234
C -2.59705 0.911572 -1.722918
C -2.748325 -1.568073 -1.485872
C -4.185318 -1.396908 -1.271165
C -4.769118 -0.102315 -1.310743
C -6.154784 0.014558 -1.096353
C -6.933668 -1.102345 -0.846625
C -6.35218 -2.377345 -0.808042
C -4.991902 -2.516065 -1.022006
H -1.97522 1.788908 -1.902447
H -0.952213 -0.463219 -2.020229
O -2.220257 -2.712601 -1.48957
H -4.507322 -3.49031 -0.991782
H -6.968907 -3.25125 -0.605606
H -8.00412 -0.988809 -0.680854
H -6.609523 1.005163 -1.125134
K 0.222207 -3.075066 -2.18569

InterA_pre-ortho_neutral_acetonitrile
C -0.9197768829 -0.3535875613 0.5297395304
C -2.1792321465 -0.3978367938 -1.9466534777
C -4.1981248737 0.896970004 -1.5923186375
C 2.1460595406 1.6116069113 -0.4084496386
C 1.8792824814 2.7388370716 0.3175552992
C 0.7409618863 2.8819399099 1.1276687476
C -0.1913405321 1.9014989253 1.2134125626
C 0.0016424597 0.6949992423 0.4582217457
C 1.2187630926 0.531423805 -0.347797081
C 1.9384254604 4.7552835842 1.267433984
H 3.0232150945 1.561673632 -1.0426678679
H -1.0346889046 1.9953239668 1.8867717638
H 2.5963004473 5.0429833042 2.0967527948
O 2.6414392222 3.8556210311 0.4079742863
O 0.793615777 4.0911912674 1.7720049589
H 1.6358653858 5.6384319774 0.6866767051
N 1.3937188049 -0.6551572233 -0.9903196476
S 2.8499988981 -1.0049924402 -1.6508946487

O 3.3311050065 -0.0469978143 -2.6547129657
O 2.7222049073 -2.4139127691 -2.0806429192
C -4.2777744389 0.5276250507 2.0285915924
C -4.6788659757 -0.7220697135 2.4912920618
C -3.0511775772 0.6861565377 1.3972366454
H -5.6489085153 -0.8429278515 2.9700089815
H -2.7950488347 1.6499254752 0.9671544879
C -3.8469742892 -1.8282444743 2.3040299008
C -2.1862833255 -0.4153035082 1.2153902571
H -4.1677746846 -2.8141671739 2.6352903936
C -2.6276647802 -1.6797346999 1.6664896241
H -1.9975060468 -2.5484597277 1.4792655156
H -4.9430354057 1.3841120677 2.1270502331
C 5.2064398283 -0.2698057485 -0.4242944654
C 6.1226571026 -0.28161886 0.6240158687
C 4.0215186444 -0.986015958 -0.3009600804
H 7.0526058947 0.278948764 0.5308180562
C 5.8706781254 -0.9963986536 1.797102248
C 3.7459829337 -1.7080158578 0.8606944425
H 2.8091570936 -2.2557093279 0.9491701465
C 4.6662256157 -1.7067119948 1.8983491943
H 4.4511581422 -2.2656559447 2.8091515307
H 5.3982375375 0.2886830629 -1.3381390221
C 6.8589073199 -1.0113624391 2.9230444858
H 7.7064397669 -0.3463878422 2.7273080802
H 6.3991689658 -0.6976675668 3.8684639458
H 7.2616599299 -2.0187095705 3.0899406223
H -0.5864316207 -1.3046427015 0.1130341891
H -4.7247746113 1.8511134619 -1.5857186782
C -2.870749937 0.8221476672 -1.9547744955
C -2.8150161513 -1.6260913581 -1.5874218424
C -4.234755941 -1.5414043302 -1.2494093983
C -4.9086354182 -0.28661844 -1.240455954
C -6.26556972 -0.2591053538 -0.8515136597
C -6.9323858704 -1.4175336152 -0.4991969404
C -6.2651657331 -2.65287234 -0.5209704809
C -4.932560878 -2.7036768431 -0.8866087706
H -2.3314477374 1.7308876603 -2.2259477904

H -1.1268063597 -0.4153638205 -2.2361499166
O -2.1954269105 -2.7351121595 -1.5189440756
H -4.3800532445 -3.6418982889 -0.8969621401
H -6.7955065431 -3.5628042482 -0.2437132859
H -7.9805082272 -1.3710055745 -0.2052828814
H -6.7844698133 0.7004707444 -0.8320261645
K 0.191747565 -2.9208985361 -2.3552129556

InterB_para_neutral_acetonitrile
C 12.790344 -16.910249 -0.747572
C 14.343354 -16.989676 -0.394944
C 11.031026 -14.003137 1.009274
C 11.33977 -13.000054 0.122858
C 12.067467 -13.228202 -1.038698
C 12.527133 -14.484316 -1.351723
C 12.225589 -15.542025 -0.470063
C 11.466825 -15.321044 0.708664
C 11.437254 -11.100893 -1.026395
H 10.448853 -13.817645 1.90517
H 13.093347 -14.663949 -2.264355
H 10.5395 -10.880057 -1.628851
O 11.045358 -11.66204 0.219558
O 12.251021 -12.044137 -1.713406
H 12.013622 -10.185467 -0.848014
N 11.197433 -16.397861 1.572307
S 9.654909 -16.669486 1.918912
O 8.870038 -15.493597 2.325474
O 9.651064 -17.831514 2.844834
C 13.001242 -17.594299 -4.521142
C 11.819624 -18.300285 -4.727532
C 13.337985 -17.149484 -3.244288
H 11.558648 -18.652534 -5.72391
H 14.273111 -16.604347 -3.106904
C 10.978087 -18.555029 -3.646743
C 12.501326 -17.398494 -2.150766
H 10.053295 -19.113348 -3.788271
C 11.317022 -18.108167 -2.374263
H 10.65071 -18.304977 -1.533747

H 13.668908 -17.38916 -5.356435
C 8.625161 -16.334647 -0.616582
C 8.084868 -16.783764 -1.8144
C 8.888343 -17.248033 0.407884
H 7.88867 -16.070455 -2.615299
C 7.779845 -18.136053 -2.012793
C 8.607299 -18.599748 0.225674
H 8.809263 -19.295606 1.036992
C 8.05084 -19.033325 -0.975614
H 7.818128 -20.090356 -1.110819
H 8.835289 -15.27698 -0.465725
C 7.158642 -18.598082 -3.296502
H 7.271864 -19.679258 -3.43728
H 6.08207 -18.381549 -3.32253
H 7.601857 -18.095328 -4.164939
H 12.296294 -17.591703 -0.041719
H 14.861721 -16.364664 -1.138498
C 14.749167 -18.414113 -0.546139
C 14.875258 -19.255776 0.498433
C 14.680691 -18.778818 1.860971
C 14.769413 -17.317054 2.061284
C 14.626516 -16.443063 0.971582
C 14.701638 -15.066305 1.206931
C 14.904532 -14.573354 2.489215
C 15.051283 -15.446619 3.570609
C 14.986494 -16.813525 3.353172
H 14.807408 -18.800988 -1.563793
H 15.038814 -20.322954 0.363207
O 14.419853 -19.537735 2.808767
H 15.133133 -17.525557 4.166223
H 15.233264 -15.055388 4.56973
H 14.95507 -13.498003 2.648337
H 14.583993 -14.375564 0.373705
K 12.116439 -18.160085 3.525413
TS_A-B_para_neutral_acetonitrile
C 12.213581 -16.798758 -0.94137
C 14.43337 -17.21712 -0.433417
C 11.183248 -13.929956 1.244344

C 11.471358 -12.890531 0.396599
C 12.005389 -13.08826 -0.880502
C 12.278386 -14.336511 -1.359159
C 12.007782 -15.456386 -0.519283
C 11.453717 -15.247771 0.802141
C 11.688447 -10.901954 -0.587554
H 10.738257 -13.769753 2.219607
H 12.662823 -14.465505 -2.364573
H 10.804805 -10.412248 -1.022406
O 11.312059 -11.557586 0.621482
O 12.19141 -11.87551 -1.491349
H 12.470402 -10.163349 -0.369261
N 11.165152 -16.336162 1.611919
S 9.610729 -16.56749 1.96506
O 8.845256 -15.351582 2.270531
O 9.577758 -17.66979 2.954214
C 12.883005 -17.344345 -4.653026
C 12.061848 -18.445595 -4.889332
C 12.961671 -16.782544 -3.384192
H 12.003622 -18.881675 -5.884749
H 13.647067 -15.953301 -3.219156
C 11.33423 -18.994299 -3.835657
C 12.217943 -17.30814 -2.312979
H 10.698707 -19.863543 -3.999871
C 11.422859 -18.440744 -2.56453
H 10.851266 -18.869655 -1.741344
H 13.476921 -16.924049 -5.46266
C 8.675553 -16.361774 -0.61377
C 8.219437 -16.873166 -1.821189
C 8.909351 -17.226912 0.458258
H 8.047023 -16.197868 -2.659522
C 7.973452 -18.242647 -1.982838
C 8.681317 -18.593036 0.316791
H 8.859436 -19.251206 1.164343
C 8.204811 -19.088741 -0.894549
H 8.010973 -20.156834 -0.999827
H 8.841458 -15.292176 -0.491105
C 7.47364 -18.776977 -3.290385

H 7.505189 -19.871907 -3.319464
H 6.43489 -18.475401 -3.479625
H 8.069175 -18.400915 -4.131845
H 11.847878 -17.519331 -0.209704
H 14.671533 -16.671585 -1.344491
C 14.404473 -18.632054 -0.463424
C 14.395798 -19.387992 0.683206
C 14.453199 -18.778675 1.982539
C 14.682667 -17.321604 2.013306
C 14.66166 -16.559285 0.817024
C 14.820886 -15.161994 0.906115
C 14.994985 -14.539765 2.129384
C 15.04184 -15.298144 3.307489
C 14.891147 -16.673839 3.240605
H 14.319872 -19.124137 -1.433581
H 14.294633 -20.470875 0.645497
O 14.252612 -19.414532 3.050016
H 14.960299 -17.293969 4.135822
H 15.212296 -14.808609 4.264883
H 15.105697 -13.45743 2.174996
H 14.79758 -14.571194 -0.009678
K 12.059155 -18.021237 3.669725

InterA_pre-para_neutral_acetonitrile
C 12.0206243857 -16.7687386217 -1.0297224901
C 14.5456001109 -17.3097485368 -0.4318435833
C 11.1961595951 -13.9400478582 1.2691202227
C 11.5101833376 -12.8900260736 0.4459470687
C 12.0209482142 -13.0696249192 -0.8481361302
C 12.2372772626 -14.3072634393 -1.3713613388
C 11.9375280734 -15.4485502316 -0.560482234
C 11.4167658614 -15.2536328975 0.7888929484
C 11.8123938918 -10.8811045213 -0.4742757264
H 10.7744364573 -13.7904713772 2.2561597667
H 12.5892317263 -14.415297912 -2.3901225486
H 10.9630736337 -10.3106032895 -0.8749129807
O 11.3990921396 -11.5630513491 0.7089777155
O 12.2338937485 -11.8469007854 -1.4254036148

H 12.6480716356 -10.2123849977 -0.2305324278
N 11.1128172931 -16.3479935599 1.5689199877
S 9.563560455 -16.5390788489 1.9836533583
O 8.8342146534 -15.3022128458 2.2885216973
O 9.5401576927 -17.6261434273 2.9870768346
C 12.9497244263 -17.3071330415 -4.6774104118
C 12.2006668419 -18.4519354791 -4.9486407477
C 12.9242484276 -16.7288656667 -3.415422378
H 12.2233939166 -18.8986201725 -5.9406766914
H 13.5629048623 -15.870510572 -3.2197885403
C 11.4436348194 -19.0311080691 -3.9318549333
C 12.1470859211 -17.2836736481 -2.3788966913
H 10.8669481937 -19.9348542248 -4.1234798677
C 11.4304483852 -18.4643565163 -2.665045477
H 10.8397010722 -18.9183487868 -1.8692835341
H 13.570380188 -16.8663862771 -5.4554273798
C 8.5794991257 -16.3445752196 -0.5742610606
C 8.1409139293 -16.8652673987 -1.7843028042
C 8.8351396835 -17.2061501949 0.4951628652
H 7.9518348253 -16.1930054498 -2.6214122311
C 7.9396362966 -18.2413815451 -1.9513471623
C 8.6450331437 -18.5777333127 0.351102804
H 8.8419380184 -19.2337168122 1.1961867157
C 8.1880982132 -19.0831757677 -0.8631035471
H 8.0267311113 -20.1561266163 -0.9724200823
H 8.7214578984 -15.2717890892 -0.448574838
C 7.4738808985 -18.789261175 -3.2657389424
H 7.5286495086 -19.8832937404 -3.2905494592
H 6.4331327469 -18.5090870843 -3.4752194273
H 8.077714989 -18.4040443994 -4.0970181504
H 11.6697888042 -17.501979351 -0.3032220824
H 14.66784592 -16.7713048283 -1.3687721738
C 14.4468652103 -18.7072201593 -0.4237945513
C 14.4127714001 -19.4308512874 0.7536759251
C 14.4789696475 -18.7894809916 2.0307891647
C 14.6956671387 -17.3324672511 2.0167571351
C 14.70622344 -16.6084225065 0.7909710638
C 14.8361418764 -15.2024625709 0.8399121436

C 14.9541342562 -14.5366761232 2.0451152917
C 14.9734939753 -15.2556804816 3.2504955011
C 14.8499307582 -16.6352895649 3.2254907713
H 14.3592089269 -19.2290823851 -1.3781822414
H 14.2887898782 -20.5122617677 0.7447149543
O 14.2829026043 -19.3960747972 3.1228294991
H 14.895304699 -17.2229533639 4.1440665263
H 15.1008025947 -14.7310484359 4.1960592233
H 15.0422276999 -13.4510377744 2.0587131964
H 14.8384593569 -14.6446092267 -0.0971498408
K 12.0486242026 -18.0637443822 3.603003941

TS_A-B_ortho_neutral_acetonitrile
C -1.044228 -0.415091 0.366015
C -1.956588 -0.359389 -1.605892
C -3.942312 1.045831 -1.553983
C 2.057673 1.589355 -0.407257
C 1.777227 2.699633 0.345002
C 0.626901 2.816303 1.125184
C -0.306946 1.824312 1.158068
C -0.085363 0.647143 0.387011
C 1.13179 0.509156 -0.390338
C 1.795601 4.699275 1.327118
H 2.949225 1.555472 -1.022769
H -1.172594 1.907191 1.803998
H 2.424398 5.003557 2.173347
O 2.539732 3.818854 0.485431
O 0.65226 4.010326 1.805605
H 1.485685 5.578311 0.741088
N 1.32606 -0.675506 -1.06464
S 2.783442 -0.987132 -1.711294
O 3.266245 -0.018023 -2.704644
O 2.700841 -2.397814 -2.157907
C -4.257501 0.245335 2.289595
C -4.514602 -1.025082 2.795681
C -3.123625 0.484349 1.520426
H -5.406562 -1.207502 3.392248
H -2.977078 1.46802 1.084685

C -3.63088 -2.066584 2.516495
C -2.215213 -0.551403 1.235894
H -3.828541 -3.068245 2.894295
C -2.504655 -1.832754 1.740902
H -1.832212 -2.654504 1.495433
H -4.956091 1.058912 2.478255
C 5.199697 -0.364043 -0.538488
C 6.123211 -0.379092 0.503125
C 3.955652 -0.957588 -0.356825
H 7.098164 0.087966 0.363728
C 5.821534 -0.975728 1.729443
C 3.630274 -1.557464 0.859586
H 2.64348 -1.997596 0.997723
C 4.558249 -1.56078 1.890995
H 4.301123 -2.020765 2.84542
H 5.427282 0.111211 -1.490358
C 6.816992 -0.992528 2.849307
H 7.717371 -0.42166 2.599343
H 6.397459 -0.568053 3.769935
H 7.132383 -2.015711 3.091669
H -0.619103 -1.380208 0.084559
H -4.417408 2.025209 -1.608234
C -2.59705 0.911572 -1.722918
C -2.748325 -1.568073 -1.485872
C -4.185318 -1.396908 -1.271165
C -4.769118 -0.102315 -1.310743
C -6.154784 0.014558 -1.096353
C -6.933668 -1.102345 -0.846625
C -6.35218 -2.377345 -0.808042
C -4.991902 -2.516065 -1.022006
H -1.97522 1.788908 -1.902447
H -0.952213 -0.463219 -2.020229
O -2.220257 -2.712601 -1.48957
H -4.507322 -3.49031 -0.991782
H -6.968907 -3.25125 -0.605606
H -8.00412 -0.988809 -0.680854
H -6.609523 1.005163 -1.125134
K 0.222207 -3.075066 -2.18569

TS_A-B_ortho_neutral_acetonitrile_conf2

C -10.480045 17.12583 -1.771509
C -11.269741 17.987538 0.13377
C -9.418035 19.267583 1.051455
C -12.932494 17.976918 -4.510173
C -13.675927 16.827119 -4.479627
C -13.419676 15.776067 -3.592654
C -12.38901 15.835421 -2.704948
C -11.578825 17.006377 -2.662713
C -11.840897 18.107907 -3.583824
C -15.033488 15.137153 -4.974607
H -13.082172 18.714702 -5.294606
H -12.17371 14.990994 -2.060311
H -14.682895 14.523249 -5.819927
O -14.75182 16.511118 -5.257751
O -14.348037 14.777112 -3.79126
H -16.111959 15.015456 -4.827526
N -11.013738 19.174125 -3.52661
S -11.243105 20.426559 -4.524457
O -11.310141 20.092185 -5.956985
O -12.374043 21.276755 -4.045194
C -9.529212 13.961284 0.132386
C -8.145671 13.974596 -0.034756
C -10.315618 14.963919 -0.422739
H -7.532609 13.189147 0.4031
H -11.386108 14.962939 -0.225297
C -7.555036 15.013567 -0.748848
C -9.740436 16.007742 -1.167079
H -6.474074 15.047022 -0.873523
C -8.342135 16.020239 -1.296329
H -7.875596 16.838478 -1.844054
H -10.00052 13.170245 0.713308
C -9.248097 22.065811 -5.358689
C -8.101328 22.834189 -5.203023
C -9.732515 21.330633 -4.277773
H -7.720613 23.407873 -6.048284
C -7.420876 22.878307 -3.981237
C -9.077377 21.360691 -3.049917

H -9.458647 20.77766 -2.214943
C -7.927933 22.130974 -2.913868
H -7.416627 22.153475 -1.951006
H -9.765968 22.014306 -6.313661
C -6.165076 23.682307 -3.831515
H -5.979987 23.955072 -2.786623
H -6.200358 24.605721 -4.421133
H -5.286638 23.119948 -4.176496
H -9.851572 17.991521 -1.981947
H -8.490096 19.326058 1.619216
C -10.09014 18.087586 0.927924
C -11.921817 19.172709 -0.367615
C -11.176329 20.433095 -0.23751
C -9.936863 20.465064 0.464356
C -9.261748 21.697911 0.586342
C -9.784544 22.857852 0.046436
C -11.012087 22.826959 -0.631233
C -11.6923 21.629831 -0.754343
H -9.694658 17.183408 1.390659
H -11.912775 17.115632 0.242747
O -13.064836 19.119458 -0.890459
H -12.640875 21.596716 -1.282496
H -11.418853 23.73611 -1.069647
H -9.241759 23.796918 0.144962
H -8.313222 21.721518 1.124219
K -14.358764 20.082973 -2.839011

TS_A-B_ortho_neutral_acetonitrile_conf3
C 0.888413 0.13675 0.529711
C 1.991302 -0.213153 -1.309966
C 3.807255 1.396013 -1.493948
C -2.327012 1.426779 -0.964306
C -2.2454 2.726157 -0.537531
C -1.193002 3.203013 0.243903
C -0.160582 2.391569 0.608514
C -0.175605 1.030689 0.187491
C -1.289514 0.52671 -0.593175
C -2.582369 4.895176 -0.155668

H -3.147256 1.107119 -1.59685
H 0.625769 2.76183 1.254967
H -3.317273 5.325371 0.536529
O -3.138753 3.728834 -0.761469
O -1.415251 4.5225 0.558376
H -2.324526 5.619751 -0.943427
N -1.283854 -0.810423 -0.923416
S -2.643835 -1.483398 -1.502777
O -3.176011 -0.899591 -2.741989
O -2.35809 -2.937176 -1.536143
C 3.790521 1.694023 2.43284
C 4.119728 0.648638 3.290074
C 2.728897 1.571608 1.542543
H 4.955399 0.748083 3.980384
H 2.531721 2.376145 0.840575
C 3.382254 -0.533604 3.244911
C 1.967828 0.389707 1.487699
H 3.638324 -1.363851 3.900854
C 2.329627 -0.661853 2.350373
H 1.774223 -1.597812 2.294212
H 4.377309 2.611185 2.440933
C -5.193766 -0.914211 -0.62088
C -6.174622 -0.774642 0.357258
C -3.897028 -1.245803 -0.245035
H -7.19163 -0.513157 0.064994
C -5.878931 -0.956378 1.710144
C -3.576861 -1.430328 1.100029
H -2.551993 -1.666843 1.383594
C -4.562925 -1.282834 2.064787
H -4.311374 -1.417966 3.116976
H -5.418904 -0.760964 -1.674194
C -6.934692 -0.802539 2.762243
H -7.087168 -1.735738 3.319554
H -7.898686 -0.515262 2.329537
H -6.660694 -0.038451 3.500667
H 0.598201 -0.915035 0.496058
H 4.183436 2.378993 -1.776949
C 2.508414 1.052921 -1.721182

C 2.884902 -1.238847 -0.808235
C 4.265091 -0.835806 -0.537546
C 4.715209 0.466581 -0.883364
C 6.049893 0.81101 -0.601139
C 6.905766 -0.092483 0.005164
C 6.455934 -1.375908 0.345246
C 5.148852 -1.738301 0.07029
H 1.823534 1.766786 -2.179338
H 1.051762 -0.547395 -1.753466
O 2.48104 -2.403243 -0.544337
H 4.764801 -2.723681 0.327978
H 7.131945 -2.07993 0.827298
H 7.93426 0.196131 0.217644
H 6.402131 1.809027 -0.863511
K 0.183398 -3.244967 -1.319709

TS_A-B_ortho_neutral_acetonitrile_conf4
C -1.550298 1.339074 -0.417924
C -0.90718 1.877872 1.683836
C 1.030204 3.315347 1.401649
C -1.805814 -2.419014 -0.62234
C -3.158769 -2.540485 -0.444935
C -4.000669 -1.443274 -0.232051
C -3.516174 -0.170626 -0.211899
C -2.116798 0.038899 -0.381384
C -1.231278 -1.102449 -0.591632
C -5.262308 -3.257541 -0.422212
H -1.202011 -3.281069 -0.896171
H -4.192837 0.669856 -0.107775
H -5.682665 -3.397769 -1.431232
O -3.897566 -3.688098 -0.430258
O -5.293937 -1.889585 -0.0649
H -5.818916 -3.844489 0.315866
N 0.080935 -0.8421 -0.769017
S 1.106464 -2.061736 -1.03178
O 0.866444 -2.795857 -2.28291
O 1.252128 -2.938855 0.169425
C -4.026271 4.231475 -0.296191

C -3.49672 5.058385 -1.285429
C -3.423633 3.015337 0.002333
H -3.969872 6.011171 -1.515162
H -3.819786 2.416642 0.820676
C -2.345051 4.663079 -1.960673
C -2.274757 2.587546 -0.684924
H -1.909879 5.306184 -2.723685
C -1.7388 3.450101 -1.655248
H -0.82978 3.149881 -2.175993
H -4.909145 4.543019 0.259502
C 3.81452 -1.894034 -0.987132
C 5.040828 -1.257358 -1.141635
C 2.638457 -1.173391 -1.181237
H 5.962181 -1.817491 -0.980256
C 5.113866 0.094506 -1.488252
C 2.685119 0.172498 -1.537748
H 1.75809 0.724048 -1.668639
C 3.917858 0.793629 -1.684268
H 3.954445 1.85407 -1.935726
H 3.762338 -2.941832 -0.697866
C 6.433122 0.789698 -1.634193
H 7.267828 0.144673 -1.339079
H 6.61217 1.10562 -2.670279
H 6.477642 1.697675 -1.018496
H -0.520535 1.350695 -0.773033
H 1.469589 4.296427 1.223401
C -0.322223 3.162343 1.49693
C -0.087515 0.732264 1.99152
C 1.365289 0.907628 1.846235
C 1.905984 2.195715 1.571402
C 3.306976 2.34135 1.506839
C 4.148215 1.262858 1.705039
C 3.613506 -0.00545 1.971732
C 2.242293 -0.167374 2.044533
H -0.979486 4.024098 1.379469
H -1.947796 1.803738 1.995489
O -0.600448 -0.35139 2.373578
H 1.818756 -1.152074 2.229463

H 4.274214 -0.860728 2.101686
H 5.228274 1.395186 1.647997
H 3.719974 3.329391 1.299534
K -0.486967 -2.849599 2.09532

TS_A-B_ortho_neutral_acetonitrile_conf5
C 12.408311 -14.658544 -1.12897
C 13.429101 -16.358262 -2.21947
C 12.819922 -16.052446 -4.551041
C 10.37179 -16.130509 1.684079
C 11.257627 -15.920497 2.707677
C 12.516675 -15.340091 2.518455
C 12.928062 -14.923093 1.289045
C 12.065752 -15.113263 0.170445
C 10.756813 -15.731792 0.358355
C 12.206596 -15.627064 4.696198
H 9.362352 -16.480563 1.88671
H 13.880665 -14.418766 1.173609
H 11.855354 -14.721452 5.216692
O 11.095321 -16.231112 4.026734
O 13.178439 -15.28821 3.72642
H 12.629547 -16.34461 5.407165
N 9.975351 -15.869344 -0.73348
S 8.503232 -16.516923 -0.583223
O 7.578191 -15.728956 0.244448
O 8.563211 -17.964947 -0.219807
C 15.649959 -12.67519 -1.392164
C 15.240858 -11.632768 -2.222122
C 14.752956 -13.666263 -1.01221
H 15.946304 -10.85936 -2.519812
H 15.111943 -14.502656 -0.415013
C 13.92744 -11.602906 -2.683248
C 13.416911 -13.6407 -1.447741
H 13.598088 -10.804367 -3.345825
C 13.033555 -12.599398 -2.30887
H 12.01006 -12.580902 -2.682783
H 16.681975 -12.725832 -1.049123
C 6.917317 -17.322923 -2.632314

C 6.407043 -17.276434 -3.9246
C 7.946074 -16.456766 -2.269715
H 5.609139 -17.961067 -4.213303
C 6.909163 -16.374554 -4.866837
C 8.457565 -15.542682 -3.187989
H 9.273568 -14.888101 -2.893208
C 7.938882 -15.511976 -4.475372
H 8.35773 -14.816326 -5.203206
H 6.538079 -18.040414 -1.907295
C 6.372179 -16.332497 -6.265051
H 5.707445 -17.178482 -6.47111
H 5.79974 -15.414053 -6.451012
H 7.181046 -16.352446 -7.006852
H 11.570484 -14.671395 -1.825037
H 12.976358 -15.583204 -5.521974
C 13.654743 -15.788265 -3.504596
C 12.445944 -17.396944 -2.035874
C 11.537397 -17.645854 -3.164855
C 11.741667 -16.983125 -4.408179
C 10.888021 -17.291674 -5.487059
C 9.870864 -18.217633 -5.352615
C 9.671904 -18.869548 -4.127412
C 10.502882 -18.585055 -3.059655
H 14.48201 -15.089198 -3.628361
H 14.22225 -16.329672 -1.47411
O 12.38499 -18.054985 -0.964833
H 10.347279 -19.075288 -2.101274
H 8.855871 -19.580903 -4.012943
H 9.218491 -18.438565 -6.19687
H 11.046808 -16.78466 -6.439735
K 10.757384 -18.992019 0.713717

TS_A-B_ortho_neutral_acetonitrile_conf6
C 11.9178778797 -15.770414348 -1.3255197918
C 13.5491553738 -16.9335850324 -2.1666535236
C 14.0939096289 -16.0945839113 -4.3850325887
C 13.8366729172 -13.9913440107 1.3926240377
C 13.7806314549 -12.7198807999 0.8852582763

C 13.1160317555 -12.4051266388 -0.3000197019
C 12.499445334 -13.3685365718 -1.04087598
C 12.5311322938 -14.7154044248 -0.5779804646
C 13.1875572944 -15.0349798806 0.6756837296
C 14.0264380434 -10.5391776188 0.5008335445
H 14.3913535128 -14.1952313245 2.3013399403
H 11.9476617502 -13.1039385871 -1.9347172823
H 13.5152032088 -9.7232670768 1.027204533
O 14.3084497575 -11.5878544009 1.4270953648
O 13.1849394304 -11.0499918475 -0.5196672025
H 14.9722274445 -10.1832008523 0.0638428739
N 13.1335848596 -16.341036028 1.1088830227
S 13.5818445532 -16.6861842092 2.6315062013
O 14.96384054 -16.3376072801 2.9897087935
O 13.1939103929 -18.1034915387 2.8246500618
C 10.4103808013 -14.7584881074 -4.6680561372
C 9.2901732917 -15.5832170857 -4.6990287167
C 11.2695235306 -14.7725697888 -3.5741899707
H 8.6245168955 -15.5718224658 -5.5601332605
H 12.1707920429 -14.1675583463 -3.6012851832
C 9.0375038536 -16.4375641978 -3.6266862892
C 11.0268778475 -15.6215440946 -2.4791484305
H 8.1723460649 -17.0980872208 -3.6452302315
C 9.8989325761 -16.4610915773 -2.5395163945
H 9.7187825729 -17.1530538519 -1.7170600555
H 10.6321257011 -14.1080821142 -5.5126378101
C 13.0502039752 -15.1209437027 4.8373649274
C 12.2124514109 -14.4078188609 5.6905059576
C 12.5174155795 -15.7228951583 3.7031070258
H 12.6282784451 -13.933457804 6.5794230831
C 10.8470741832 -14.28108782 5.4250749664
C 11.1571461381 -15.6095424259 3.4160827827
H 10.7561383639 -16.0664039175 2.512153934
C 10.3347311412 -14.8911642342 4.2720349493
H 9.2731122486 -14.7942280516 4.0435330556
H 14.1163485013 -15.2099493578 5.0349527053
C 9.9474217665 -13.5081373839 6.3408210781
H 10.4990932283 -13.0812485694 7.1848911736

H 9.4512805034 -12.6817170142 5.8162080539
H 9.1505144931 -14.1409916991 6.7519075181
H 11.6736415326 -16.6459248796 -0.7212751588
H 14.6193651764 -15.3962616401 -5.0362658797
C 14.2695425611 -16.0573701958 -3.0343080538
C 12.7512582078 -18.0119040714 -2.7167575999
C 12.5495842465 -18.0105994811 -4.1656715933
C 13.2190063883 -17.0621641477 -4.9845741142
C 12.990085507 -17.0955544703 -6.3724331477
C 12.1294398584 -18.0253747966 -6.930270009
C 11.47116471 -18.9586777393 -6.1172640022
C 11.6862045267 -18.9476208376 -4.7502219066
H 14.9316354199 -15.3159045433 -2.5865725557
H 13.9204018918 -17.0614716259 -1.1482660213
O 12.1919254826 -18.8685181486 -1.9806015133
H 11.1853736334 -19.6528219776 -4.0894066525
H 10.7911584236 -19.6836952831 -6.5610960529
H 11.9645199354 -18.0317268338 -8.0068363924
H 13.5000900586 -16.3701874454 -7.0070143244
K 12.7676228582 -19.1230436505 0.5044904018

TS_A-B_ortho_neutral_acetonitrile_conf7
C 11.933615 -15.028817 -1.265444
C 13.292997 -16.684073 -1.630191
C 14.081799 -16.612834 -3.931378
C 14.139526 -13.010044 1.035574
C 14.367692 -11.938149 0.21323
C 13.80563 -11.82492 -1.058348
C 13.008643 -12.806168 -1.567522
C 12.742049 -13.956495 -0.770619
C 13.290659 -14.052332 0.568896
C 15.080424 -10.035872 -0.701872
H 14.622312 -13.069828 2.004211
H 12.541742 -12.685692 -2.537552
H 14.743351 -9.024529 -0.441281
O 15.118547 -10.834293 0.480532
O 14.168577 -10.622158 -1.615864
H 16.087541 -10.004098 -1.145419

N 12.9452 -15.152416 1.321562
S 13.311944 -15.174288 2.903729
O 14.738462 -15.047884 3.232701
O 12.622049 -16.374681 3.432628
C 10.782761 -14.681084 -4.873597
C 9.511547 -15.240351 -4.788384
C 11.584426 -14.570555 -3.742324
H 8.890902 -15.329874 -5.678074
H 12.595939 -14.189643 -3.845844
C 9.046643 -15.702341 -3.557898
C 11.129593 -15.022984 -2.490376
H 8.059558 -16.154993 -3.48071
C 9.848365 -15.60214 -2.430085
H 9.496705 -15.993293 -1.475628
H 11.16649 -14.34304 -5.834849
C 13.147216 -13.026952 4.621277
C 12.493322 -11.957228 5.226142
C 12.491915 -13.761308 3.639212
H 13.006165 -11.378666 5.99449
C 11.192046 -11.604439 4.861141
C 11.193207 -13.426989 3.256036
H 10.699339 -13.994973 2.468628
C 10.555384 -12.354534 3.863231
H 9.544041 -12.086138 3.556982
H 14.165724 -13.294963 4.894016
C 10.488397 -10.453755 5.513958
H 11.147768 -9.912705 6.200659
H 10.115098 -9.735167 4.77372
H 9.617003 -10.79024 6.090709
H 11.487939 -15.637622 -0.476735
H 14.770008 -16.242085 -4.690833
C 14.216574 -16.243863 -2.626681
C 12.294465 -17.6847 -1.951854
C 12.14515 -18.037998 -3.363498
C 13.035211 -17.508489 -4.335971
C 12.851834 -17.875104 -5.681929
C 11.825665 -18.726193 -6.054891
C 10.949019 -19.244064 -5.091201

C 11.115148 -18.902028 -3.760598
H 15.011288 -15.559674 -2.328435
H 13.595322 -16.603349 -0.584536
O 11.536044 -18.175238 -1.072687
H 10.448398 -19.280128 -2.987736
H 10.140085 -19.907752 -5.391569
H 11.700201 -18.994983 -7.102864
H 13.531501 -17.472816 -6.4337
K 11.966874 -17.851517 1.434461

TS_A-B_para_neutral_acetonitrile
C 12.213581 -16.798758 -0.94137
C 14.43337 -17.21712 -0.433417
C 11.183248 -13.929956 1.244344
C 11.471358 -12.890531 0.396599
C 12.005389 -13.08826 -0.880502
C 12.278386 -14.336511 -1.359159
C 12.007782 -15.456386 -0.519283
C 11.453717 -15.247771 0.802141
C 11.688447 -10.901954 -0.587554
H 10.738257 -13.769753 2.219607
H 12.662823 -14.465505 -2.364573
H 10.804805 -10.412248 -1.022406
O 11.312059 -11.557586 0.621482
O 12.19141 -11.87551 -1.491349
H 12.470402 -10.163349 -0.369261
N 11.165152 -16.336162 1.611919
S 9.610729 -16.56749 1.96506
O 8.845256 -15.351582 2.270531
O 9.577758 -17.66979 2.954214
C 12.883005 -17.344345 -4.653026
C 12.061848 -18.445595 -4.889332
C 12.961671 -16.782544 -3.384192
H 12.003622 -18.881675 -5.884749
H 13.647067 -15.953301 -3.219156
C 11.33423 -18.994299 -3.835657
C 12.217943 -17.30814 -2.312979
H 10.698707 -19.863543 -3.999871

C 11.422859 -18.440744 -2.56453
H 10.851266 -18.869655 -1.741344
H 13.476921 -16.924049 -5.46266
C 8.675553 -16.361774 -0.61377
C 8.219437 -16.873166 -1.821189
C 8.909351 -17.226912 0.458258
H 8.047023 -16.197868 -2.659522
C 7.973452 -18.242647 -1.982838
C 8.681317 -18.593036 0.316791
H 8.859436 -19.251206 1.164343
C 8.204811 -19.088741 -0.894549
H 8.010973 -20.156834 -0.999827
H 8.841458 -15.292176 -0.491105
C 7.47364 -18.776977 -3.290385
H 7.505189 -19.871907 -3.319464
H 6.43489 -18.475401 -3.479625
H 8.069175 -18.400915 -4.131845
H 11.847878 -17.519331 -0.209704
H 14.671533 -16.671585 -1.344491
C 14.404473 -18.632054 -0.463424
C 14.395798 -19.387992 0.683206
C 14.453199 -18.778675 1.982539
C 14.682667 -17.321604 2.013306
C 14.66166 -16.559285 0.817024
C 14.820886 -15.161994 0.906115
C 14.994985 -14.539765 2.129384
C 15.04184 -15.298144 3.307489
C 14.891147 -16.673839 3.240605
H 14.319872 -19.124137 -1.433581
H 14.294633 -20.470875 0.645497
O 14.252612 -19.414532 3.050016
H 14.960299 -17.293969 4.135822
H 15.212296 -14.808609 4.264883
H 15.105697 -13.45743 2.174996
H 14.79758 -14.571194 -0.009678
K 12.059155 -18.021237 3.669725

TS_A-B_para_neutral_acetonitrile_conf2

C -12.96485 16.008814 0.739989

C -14.65365 17.597033 0.71641

C -11.161613 16.400247 -2.525174

C -11.868721 15.497911 -3.278617

C -12.935353 14.757452 -2.759214

C -13.341283 14.894526 -1.463798

C -12.649523 15.825117 -0.634065

C -11.545139 16.589669 -1.175136

C -12.626307 14.142818 -4.878967

H -10.313785 16.944388 -2.925178

H -14.149761 14.285327 -1.075899

H -12.082485 13.215153 -5.109839

O -11.686885 15.175578 -4.588467

O -13.448171 13.946026 -3.737374

H -13.246749 14.448639 -5.731155

N -10.853317 17.476141 -0.363341

S -9.308973 17.117852 -0.077027

O -8.551138 16.623494 -1.234078

O -8.754426 18.271332 0.669038

C -15.298166 13.447443 2.326112

C -14.639789 13.235154 3.536289

C -14.787238 14.341431 1.392586

H -15.044204 12.534218 4.263989

H -15.348468 14.522988 0.477904

C -13.472768 13.943818 3.813297

C -13.598865 15.049619 1.644341

H -12.953994 13.800445 4.760301

C -12.9689 14.84683 2.88562

H -12.054463 15.399235 3.102404

H -16.225448 12.919545 2.110752

C -9.739173 14.491314 0.618232

C -9.893892 13.447904 1.520699

C -9.381217 15.759497 1.083514

H -10.183123 12.461726 1.156802

C -9.682925 13.637371 2.892576

C -9.174687 15.972368 2.443742

H -8.886102 16.963043 2.787994

C -9.313988 14.910824 3.334865
H -9.136273 15.074319 4.398408
H -9.883341 14.328002 -0.449084
C -9.853301 12.49962 3.852504
H -9.086655 11.727591 3.705473
H -10.825387 12.007185 3.721264
H -9.785135 12.834226 4.893647
H -12.261833 16.677245 1.237254
H -15.318052 16.780836 0.439028
C -14.590162 18.006975 2.069599
C -14.001233 19.188359 2.449169
C -13.448109 20.093988 1.481434
C -13.692406 19.766686 0.063789
C -14.276161 18.52684 -0.303779
C -14.423602 18.23194 -1.674004
C -14.013696 19.127859 -2.645175
C -13.464664 20.364722 -2.279072
C -13.313664 20.674471 -0.937056
H -14.964907 17.322651 2.832344
H -13.889529 19.45259 3.498867
O -12.731607 21.080847 1.792519
H -12.923187 21.6434 -0.62152
H -13.175629 21.081572 -3.045808
H -14.128191 18.87441 -3.698
H -14.86285 17.276679 -1.961857
K -10.577688 20.128053 0.522101

TS_A-B_para_neutral_acetonitrile_conf3
C -12.62546 17.086859 1.385446
C -14.721157 17.837646 1.8651
C -9.928746 19.642397 1.905422
C -9.771992 20.066444 0.610048
C -10.569482 19.591034 -0.434258
C -11.537639 18.652876 -0.226903
C -11.737188 18.168433 1.098308
C -10.948959 18.700389 2.183325
C -9.093313 21.029202 -1.282491
H -9.289333 19.999709 2.705321

H -12.105174 18.258976 -1.062543
H -8.191091 20.662485 -1.793543
O -8.883519 20.977483 0.12795
O -10.199316 20.200484 -1.605704
H -9.309315 22.064544 -1.576674
N -11.211006 18.319469 3.488919
S -10.070647 17.561521 4.323572
O -8.688235 17.819595 3.895643
O -10.402839 17.777636 5.753128
C -13.932076 15.228405 -1.67131
C -13.642514 13.914065 -1.305268
C -13.622454 16.280286 -0.818223
H -13.884215 13.093119 -1.977634
H -13.902933 17.294814 -1.099066
C -13.06209 13.661278 -0.065341
C -13.011104 16.045164 0.425256
H -12.842821 12.63891 0.240036
C -12.763694 14.713085 0.794274
H -12.311993 14.515559 1.765894
H -14.413162 15.434543 -2.625826
C -9.508688 15.082605 3.228801
C -9.787933 13.740253 2.978597
C -10.368604 15.821029 4.034694
H -9.114849 13.160269 2.346808
C -10.916929 13.121547 3.521067
C -11.514144 15.229884 4.571594
H -12.193309 15.826121 5.180237
C -11.777443 13.891225 4.318003
H -12.673216 13.430382 4.736981
H -8.623537 15.563034 2.817756
C -11.212413 11.676474 3.255231
H -12.27747 11.512359 3.048064
H -10.960874 11.047118 4.119569
H -10.640488 11.296368 2.401487
H -12.516232 16.721081 2.406427
H -15.117159 17.119287 1.152137
C -14.66892 19.202252 1.494526
C -14.507828 20.203759 2.415674

C -14.478589 19.929482 3.827396
C -14.725878 18.534238 4.233809
C -14.837634 17.509409 3.261355
C -15.040747 16.184614 3.695902
C -15.146609 15.885034 5.04227
C -15.05785 16.903558 6.003267
C -14.848777 18.209674 5.594054
H -14.685005 19.452146 0.432177
H -14.390663 21.241873 2.111066
O -14.222337 20.81333 4.68471
H -14.820608 19.026638 6.316749
H -15.166548 16.667656 7.060368
H -15.310251 14.855195 5.357353
H -15.111224 15.392166 2.948936
K -12.022391 19.827616 5.70211

TS_A-B_para_neutral_acetonitrile_conf4
C -12.732451 16.29173 0.505243
C -14.782209 17.421084 0.75987
C -11.629383 17.39075 -2.905408
C -12.210185 16.400894 -3.660096
C -12.917216 15.34366 -3.081958
C -13.094023 15.247688 -1.731268
C -12.543314 16.269793 -0.90428
C -11.77459 17.336382 -1.500451
C -12.834376 15.007322 -5.282969
H -11.034082 18.182808 -3.350916
H -13.623566 14.403192 -1.303504
H -12.09604 14.301016 -5.691283
O -12.189468 16.251907 -5.013121
O -13.363794 14.500941 -4.067383
H -13.64871 15.171397 -5.999806
N -11.137416 18.317087 -0.747488
S -9.748203 17.908853 -0.058964
O -9.249074 19.13984 0.59814
O -9.797268 16.679067 0.751598
C -14.21348 13.169288 2.097869
C -13.368686 13.033836 3.197861

C -14.03374 14.213385 1.198405
H -13.511898 12.214534 3.89985
H -14.736242 14.326579 0.37436
C -12.350278 13.96298 3.399258
C -13.004498 15.153116 1.380569
H -11.68748 13.870115 4.257701
C -12.177037 15.016463 2.510415
H -11.370798 15.734458 2.652098
H -15.02683 12.462348 1.943903
C -7.876847 18.566975 -1.983894
C -7.086167 18.290524 -3.092041
C -8.616272 17.544232 -1.39266
H -6.498142 19.088165 -3.547279
C -7.026682 17.001227 -3.635461
C -8.572301 16.253468 -1.915608
H -9.150787 15.463216 -1.439557
C -7.782485 15.992643 -3.030636
H -7.746253 14.982638 -3.439647
H -7.915621 19.56727 -1.557204
C -6.152321 16.708369 -4.816816
H -5.101917 16.590477 -4.517963
H -6.180458 17.518391 -5.555174
H -6.448669 15.782246 -5.321286
H -12.206155 17.10463 0.996696
H -15.260108 16.492538 0.453748
C -14.66506 17.723317 2.135724
C -14.301828 18.971778 2.582249
C -14.052013 20.050529 1.668782
C -14.362328 19.791245 0.250501
C -14.714105 18.487974 -0.189171
C -14.939969 18.280598 -1.564858
C -14.828765 19.319405 -2.471344
C -14.504623 20.610495 -2.031281
C -14.277613 20.835242 -0.683231
H -14.802834 16.914398 2.854738
H -14.139574 19.167026 3.640383
O -13.532068 21.143496 2.017439
H -14.056754 21.834889 -0.305601

H -14.447779 21.431545 -2.744142
H -14.99955 19.133406 -3.53082
H -15.19806 17.278494 -1.908881
K -11.318093 20.763268 0.569095

TS_A-B_para_neutral_acetonitrile_conf5
C -12.732451 16.29173 0.505243
C -14.782209 17.421084 0.75987
C -11.629383 17.39075 -2.905408
C -12.210185 16.400894 -3.660096
C -12.917216 15.34366 -3.081958
C -13.094023 15.247688 -1.731268
C -12.543314 16.269793 -0.90428
C -11.77459 17.336382 -1.500451
C -12.834375 15.007322 -5.282969
H -11.034082 18.182808 -3.350916
H -13.623566 14.403192 -1.303504
H -12.09604 14.301016 -5.691283
O -12.189468 16.251907 -5.013121
O -13.363794 14.500941 -4.067383
H -13.64871 15.171396 -5.999806
N -11.137417 18.317088 -0.747488
S -9.748203 17.908853 -0.058964
O -9.249074 19.13984 0.59814
O -9.797268 16.679067 0.751598
C -14.21348 13.169288 2.097869
C -13.368686 13.033836 3.197862
C -14.03374 14.213385 1.198405
H -13.511897 12.214534 3.89985
H -14.736242 14.326579 0.374361
C -12.350278 13.96298 3.399258
C -13.004498 15.153116 1.380569
H -11.68748 13.870115 4.257701
C -12.177037 15.016464 2.510415
H -11.370798 15.734458 2.652098
H -15.02683 12.462348 1.943903
C -7.876847 18.566975 -1.983894
C -7.086167 18.290524 -3.092041

C -8.616272 17.544233 -1.39266
H -6.498142 19.088165 -3.547279
C -7.026682 17.001227 -3.635461
C -8.572301 16.253468 -1.915608
H -9.150787 15.463216 -1.439557
C -7.782485 15.992643 -3.030636
H -7.746252 14.982638 -3.439646
H -7.915621 19.56727 -1.557204
C -6.152321 16.708369 -4.816816
H -5.101917 16.590476 -4.517963
H -6.180457 17.518391 -5.555174
H -6.44867 15.782247 -5.321287
H -12.206155 17.10463 0.996696
H -15.260108 16.492538 0.453748
C -14.66506 17.723317 2.135723
C -14.301829 18.971778 2.582249
C -14.052013 20.050529 1.668782
C -14.362328 19.791245 0.250501
C -14.714105 18.487974 -0.189171
C -14.939969 18.280598 -1.564858
C -14.828765 19.319405 -2.471344
C -14.504623 20.610495 -2.031281
C -14.277613 20.835242 -0.68323
H -14.802834 16.914398 2.854738
H -14.139574 19.167026 3.640383
O -13.532068 21.143496 2.017439
H -14.056754 21.834889 -0.305601
H -14.447779 21.431545 -2.744142
H -14.99955 19.133406 -3.53082
H -15.19806 17.278494 -1.908881
K -11.318093 20.763268 0.569094

TS_A-B_para_neutral_acetonitrile_conf6
C 1.566374 0.294742 0.502043
C 3.031904 -0.338171 -1.120963
C -1.552643 1.220541 -1.368485
C -1.295424 2.551458 -1.591537
C -0.163191 3.183426 -1.075396

C 0.771215 2.503215 -0.346584
C 0.576347 1.11075 -0.132027
C -0.618836 0.471846 -0.615746
C -1.387341 4.709278 -2.145188
H -2.464887 0.744261 -1.716329
H 1.635815 3.018293 0.058568
H -2.036662 5.410188 -1.600836
O -2.055734 3.454862 -2.270567
O -0.182456 4.510473 -1.421005
H -1.156637 5.097088 -3.146605
N -0.922219 -0.866396 -0.371898
S -1.460096 -1.252724 1.084548
O -1.79857 -2.694933 1.011343
O -0.613329 -0.810858 2.207014
C 4.324292 2.118003 2.387031
C 4.315793 1.40343 3.584391
C 3.443063 1.784839 1.36618
H 5.007805 1.666162 4.382418
H 3.502119 2.317646 0.417777
C 3.427711 0.342889 3.744732
C 2.529738 0.727049 1.517392
H 3.417757 -0.225976 4.672798
C 2.551918 -0.000322 2.720605
H 1.840626 -0.814868 2.846843
H 5.031508 2.932889 2.242534
C -4.205928 -0.960268 0.9087
C -5.383155 -0.223974 0.976305
C -3.003157 -0.374965 1.296581
H -6.32641 -0.68681 0.684203
C -5.381748 1.103949 1.417176
C -2.978273 0.946923 1.740719
H -2.032522 1.391441 2.047368
C -4.161422 1.674638 1.794494
H -4.141407 2.708124 2.141543
H -4.210436 -1.996408 0.57598
C -6.657747 1.884146 1.513067
H -6.495267 2.953028 1.333593
H -7.10747 1.794604 2.511407

H -7.405539 1.530932 0.794131
H 1.236047 -0.730513 0.637279
H 3.81833 0.265442 -0.67415
C 2.419334 0.118378 -2.313676
C 1.645326 -0.69639 -3.09591
C 1.476057 -2.092711 -2.787295
C 2.278979 -2.62214 -1.670217
C 3.052404 -1.75254 -0.859474
C 3.78318 -2.300529 0.213454
C 3.755558 -3.659312 0.471367
C 3.005387 -4.52126 -0.342885
C 2.279386 -4.000131 -1.40011
H 2.497208 1.178358 -2.560843
H 1.106275 -0.310541 -3.959015
O 0.660286 -2.82839 -3.397506
H 1.722195 -4.650181 -2.076644
H 3.00842 -5.592775 -0.150808
H 4.328779 -4.063288 1.304144
H 4.372062 -1.632909 0.843464
K -0.96911 -3.393986 -1.399628

Table S7. Computed energies for the neutral transition state conformational analysis in acetonitrile.

	M06-L/BS1							TPSS0-D4/BS2
	1st frequency (cm-1)	Electronic energy	ZPE corr	Thermal corr	Enthalpy corr:	Gibbs corr	Electronic energy in acetonitrile	Electronic energy
pre-ortho TS								
TS_A-B_ortho_neutral_toluene	-341.5	-2052.91356	0.47902	0.51270	0.51364	0.40867	-2052.94512	-2625.35378
TS_A-B_ortho_neutral_toluene_conf2	-294.6	-2052.90302	0.47897	0.51249	0.51343	0.41197	-2052.93549	-2625.34095
TS_A-B_ortho_neutral_toluene_conf3	-341.7	-2052.91355	0.47920	0.51281	0.51375	0.40978	-2052.94508	-2625.35381
TS_A-B_ortho_neutral_toluene_conf4	-262.2	-2052.90406	0.47878	0.51226	0.51320	0.41229	-2052.93678	-2625.34267
TS_A-B_ortho_neutral_toluene_conf5	-261.3	-2052.90409	0.47863	0.51217	0.51311	0.41156	-2052.93681	-2625.34257
TS_A-B_ortho_neutral_toluene_conf6	-342.1	-2052.91359	0.47928	0.51285	0.51379	0.40971	-2052.94509	-2625.35385
TS_A-B_ortho_neutral_toluene_conf7	-343.6	-2052.91357	0.47936	0.51293	0.51387	0.41033	-2052.94510	-2625.35379
pre-para TS								
TS_A-B_para_neutral_toluene	-197.4	-2052.90607	0.47895	0.51245	0.51340	0.41140	-2052.93626	-2625.34463
TS_A-B_para_neutral_toluene_conf2	-190.9	-2052.90604	0.47916	0.51259	0.51353	0.41193	-2052.93614	-2625.34450
TS_A-B_para_neutral_toluene_conf3	-250.2	-2052.90234	0.47913	0.51258	0.51353	0.41181	-2052.93324	-2625.34163
TS_A-B_para_neutral_toluene_conf4	-141.4	-2052.90126	0.47879	0.51250	0.51344	0.40937	-2052.93233	-2625.34002
TS_A-B_para_neutral_toluene_conf5	-141.4	-2052.90126	0.47879	0.51250	0.51344	0.40937	-2052.93233	-2625.34002
TS_A-B_para_neutral_toluene_conf6	-261.6	-2052.89763	0.47876	0.51261	0.51355	0.40826	-2052.92908	-2625.33890

Table S8. Computed energies for the neutral path in acetonitrile.

	M06-L/BS1							TPSS0-D4/BS2
	1st frequency (cm-1)	Electronic energy	ZPE corr	Thermal corr	Enthalpy corr:	Gibbs corr	Electronic energy in acetonitrile	Electronic energy
Path for ortho product								
InterA_pre-ortho_neutral_toluene	7.5	-2052.91780	0.47847	0.51213	0.51307	0.40917	-2052.95811	-2625.34605
TS_A-B_ortho_neutral_toluene	-341.5	-2052.91356	0.47902	0.51270	0.51364	0.40867	-2052.94512	-2625.35378
InterB_ortho_neutral_toluene	9.2	-2052.92805	0.48163	0.51520	0.51614	0.41131	-2052.97162	-**2623.34464**
Path for para product								
InterA_pre-para_neutral_toluene	19.7	-2052.90677	0.47904	0.51329	0.51423	0.41070	-2052.94690	-2625.33855

TS_A-B_para_neutral_toluene	-197.4	-2052.90607	0.47895	0.51245	0.51340	0.41140	-2052.93626	-2625.34463
InterB_para_neutral_toluene	17.3	-2052.92847	0.48178	0.51508	0.51603	0.41488	-2052.96939	-2625.34385

InterA_pre-ortho_neutral_toluene

C -0.9198828746 -0.3538204563 0.5293838014
C -2.1790137715 -0.3978533346 -1.946943093
C -4.1977117256 0.8972589047 -1.5926342007
C 2.1458618681 1.611603514 -0.4086207001
C 1.8789865765 2.7387830875 0.3174294679
C 0.7406511171 2.8817599738 1.1275487836
C -0.1915820977 1.9012491044 1.2132367732
C 0.0014847623 0.6948179871 0.4579609031
C 1.2186484159 0.5313411817 -0.3480142869
C 1.9379557871 4.7551969366 1.2673916666
H 3.0230338224 1.5617667176 -1.0428193923
H -1.0349604182 1.994982515 1.8865750391
H 2.5958034308 5.0429389214 2.0967165534
O 2.6410552642 3.8556218514 0.4079094866
O 0.7932111528 4.0909826446 1.7719470599
H 1.6353118993 5.6383305274 0.6866545919
N 1.3936968324 -0.6552952857 -0.9904199216
S 2.8500068526 -1.0052734797 -1.6508683462
O 3.3311723026 -0.0474689647 -2.6548406476
O 2.7222094607 -2.4142654236 -2.0803797428
C -4.2775513628 0.527075058 2.0291509809
C -4.6786465131 -0.7227569281 2.4914765551
C -3.0510707232 0.6857436553 1.3976031541
H -5.6486031174 -0.8437150288 2.9703421116
H -2.7949309985 1.6496612185 0.9678585414
C -3.8468729373 -1.8289263631 2.3036522094
C -2.1862936022 -0.415714074 1.2151904594
H -4.1676778088 -2.8149439376 2.6346262199
C -2.6276767039 -1.6802745005 1.6659305047
H -1.997606621 -2.5489765753 1.4782956676
H -4.9427226017 1.3835799802 2.1280637111
C 5.2064694435 -0.2700073552 -0.4243549006

C 6.1226573258 -0.2816719651 0.6239823237
C 4.0214846329 -0.9860908421 -0.3008981272
H 7.052657241 0.2787947877 0.5306872729
C 5.8705857673 -0.9961760062 1.7972169263
C 3.7458598747 -1.7078222639 0.8609025598
H 2.8089885854 -2.255422447 0.9494690004
C 4.6660724238 -1.7063681417 1.8985838914
H 4.4509337609 -2.2650983975 2.809500877
H 5.3983422115 0.2882639429 -1.338316949
C 6.8587800499 -1.0109730473 2.9231922694
H 7.7064003049 -0.3461540224 2.7273101883
H 6.3990522807 -0.6969753888 3.8685168944
H 7.2614017703 -2.018326961 3.09036144
H -0.5865508848 -1.3047800603 0.1124448517
H -4.7242399121 1.8514706499 -1.58613002
C -2.8703746291 0.8222139006 -1.9551887771
C -2.814925099 -1.6259831861 -1.5875019092
C -4.2346194524 -1.5410597858 -1.2493499174
C -4.9083335209 -0.2861837717 -1.2405098912
C -6.2652194176 -0.2584351021 -0.8514149138
C -6.9321421577 -1.4167216428 -0.4988329699
C -6.2650821552 -2.6521483818 -0.5204837776
C -4.9325286447 -2.7031833552 -0.8862774304
H -2.3309791073 1.7308459763 -2.2265403472
H -1.1266136097 -0.4155398231 -2.2365058909
O -2.1954752365 -2.7350755934 -1.5189328511
H -4.3801425563 -3.6414770013 -0.8965467281
H -6.7955064354 -3.5619670427 -0.2430161956
H -7.9802237347 -1.3700123881 -0.2048016317
H -6.7839933574 0.7012111993 -0.8320191539
K 0.1917225707 -2.9211859112 -2.355006024

TS_A-B_ortho_neutral_toluene

C -1.044212584 -0.415091042 0.3659847809
C -1.9565746477 -0.3593801016 -1.6059180781
C -3.9423212685 1.0458081645 -1.5539911981
C 2.0576694808 1.58939151 -0.4072683077
C 1.7772121126 2.6996609471 0.3449979128

C 0.6268816251 2.8163166418 1.1251772182
C -0.3069564668 1.8243162013 1.1580511624
C -0.0853607935 0.6471543226 0.386987841
C 1.1317950033 0.5091846224 -0.3903599404
C 1.7955651442 4.699296498 1.3271284126
H 2.9492235783 1.5555189498 -1.0227781426
H -1.1726070771 1.9071843945 1.8039798656
H 2.4243566533 5.0035768879 2.1733622761
O 2.5397062637 3.8188883045 0.4854374991
O 0.6522283796 4.0103353025 1.8056056667
H 1.4856441156 5.5783352972 0.7411038422
N 1.3260776305 -0.6754701138 -1.0646711931
S 2.7834745479 -0.987089981 -1.7112935398
O 3.266298137 -0.0179761944 -2.7046303014
O 2.7008870071 -2.3977697129 -2.1579149548
C -4.2574880149 0.2452792998 2.2895809251
C -4.5145700464 -1.0251445017 2.7956589058
C -3.1236172666 0.4843133477 1.5204095396
H -5.4065256505 -1.2075802822 3.39222861
H -2.9770852499 1.467989224 1.084674951
C -3.6308355223 -2.0666331742 2.5164639562
C -2.2151931142 -0.5514247122 1.2358677325
H -3.828481598 -3.068298983 2.8942571411
C -2.5046156286 -1.8327831062 1.7408676997
H -1.8321627792 -2.6545225761 1.4953910534
H -4.9560872004 1.058846286 2.4782487827
C 5.1996867368 -0.3639690988 -0.5384143447
C 6.1231724128 -0.3790152307 0.503223865
C 3.9556511011 -0.9575473937 -0.356796258
H 7.0981184524 0.0880688545 0.3638628414
C 5.8214766482 -0.9756825705 1.7295220935
C 3.6302541763 -1.5574540249 0.8595936239
H 2.6434667707 -1.9976121474 0.9976954733
C 4.5582009715 -1.5607681983 1.8910281549
H 4.3010599907 -2.0207785061 2.8454371774
H 5.427286888 0.1113085588 -1.4902693456
C 6.8169055512 -0.9924831901 2.8494117025
H 7.7172673959 -0.4215683397 2.599493295

H 6.3973308361 -0.5680613676 3.7700444849
H 7.1323335376 -2.015662318 3.0917426846
H -0.619074801 -1.3801999142 0.0845204327
H -4.4174328479 2.0251785074 -1.6082313786
C -2.5970575032 0.9115716941 -1.7229302204
C -2.7482927364 -1.5680779908 -1.4859083377
C -4.1852879783 -1.3969378572 -1.2711959962
C -4.7691084669 -0.1023529438 -1.3107604657
C -6.1547761208 0.014495378 -1.0963669405
C -6.9336418459 -1.1024220257 -0.8466490288
C -6.3521337882 -2.3774135033 -0.8080797924
C -4.991853919 -2.5161101292 -1.0220474047
H -1.9752419546 1.78891947 -1.9024517263
H -0.9521997707 -0.4631907672 -2.0202584494
O -2.2202079362 -2.7125982138 -1.4896205976
H -4.5072581169 -3.490347425 -0.9918338926
H -6.9688468373 -3.25133057 -0.6056513018
H -8.0040954329 -0.9889053217 -0.680874523
H -6.6095302915 1.0050939253 -1.1251369867
K 0.2222501076 -3.0750090609 -2.1857919574

InterB_ortho_neutral_toluene

C -1.199022 -0.4458 0.299955
C -1.845685 -0.421577 -1.171184
C -3.783908 1.021714 -1.737087
C 1.986271 1.586997 -0.230323
C 1.680892 2.684455 0.537202
C 0.503144 2.786968 1.258972
C -0.438468 1.787885 1.223024
C -0.175353 0.64425 0.444637
C 1.051937 0.517145 -0.275444
C 1.623735 4.685322 1.500353
H 2.906482 1.558285 -0.803451
H -1.348856 1.868007 1.808482
H 2.191199 5.076936 2.353148
O 2.45 3.799667 0.752322
O 0.494672 3.965847 1.973453
H 1.291782 5.511494 0.847596

N 1.26313 -0.677314 -0.966461
S 2.674447 -0.930177 -1.697335
O 3.093832 0.076444 -2.682177
O 2.608873 -2.332483 -2.184653
C -4.339686 0.168253 2.4183
C -4.417635 -1.02437 3.131915
C -3.287195 0.394942 1.536093
H -5.243955 -1.201073 3.818078
H -3.258257 1.324157 0.972496
C -3.42892 -1.988586 2.959147
C -2.28795 -0.56892 1.341385
H -3.475419 -2.926548 3.510111
C -2.38198 -1.759063 2.071948
H -1.622169 -2.527704 1.922897
H -5.107634 0.930013 2.542786
C 5.211818 -0.457183 -0.720466
C 6.20715 -0.50454 0.251542
C 3.937195 -0.92605 -0.420272
H 7.205755 -0.135252 0.017138
C 5.949306 -1.008409 1.528655
C 3.656562 -1.429229 0.849464
H 2.645231 -1.760939 1.083332
C 4.655933 -1.465812 1.812212
H 4.43119 -1.847635 2.808648
H 5.406996 -0.047756 -1.709373
C 7.019525 -1.050125 2.57703
H 7.969369 -0.655337 2.201251
H 6.74244 -0.460826 3.460515
H 7.202119 -2.073886 2.928297
H -0.656388 -1.402549 0.301669
H -4.204792 1.99569 -1.987072
C -2.464121 0.880862 -1.521743
C -2.755175 -1.617523 -1.275932
C -4.199485 -1.391098 -1.338763
C -4.700427 -0.089471 -1.587586
C -6.088511 0.088337 -1.660109
C -6.952973 -0.981536 -1.470991
C -6.452503 -2.261806 -1.213203

C -5.082046 -2.462025 -1.158096
H -1.794682 1.738811 -1.580441
H -0.962173 -0.58147 -1.812292
O -2.276904 -2.763594 -1.23904
H -4.659021 -3.445249 -0.962162
H -7.135991 -3.093937 -1.057614
H -8.028523 -0.820453 -1.5226
H -6.484793 1.083727 -1.85889
K 0.280363 -3.227529 -1.630351

InterA_pre-para_neutral_toluene
C 12.0205830185 -16.7687937929 -1.0294883044
C 14.5453825304 -17.3098068044 -0.431948908
C 11.1960742891 -13.9400763679 1.26927222
C 11.5100983991 -12.8900596396 0.4460954945
C 12.0210100895 -13.0696615662 -0.8479394375
C 12.2374395035 -14.3072923174 -1.3711371818
C 11.9375942942 -15.448574612 -0.5602769275
C 11.4167982189 -15.2536451001 0.7890497986
C 11.8121522756 -10.8811492832 -0.474210689
H 10.7742842151 -13.7905376491 2.2562930502
H 12.5895862619 -14.4153095428 -2.3898338045
H 10.9626591327 -10.3109939048 -0.8749885678
O 11.3989912982 -11.5630779702 0.709103379
O 12.2340648127 -11.8469361199 -1.4251668338
H 12.6475799768 -10.2121150401 -0.230508308
N 11.1128716019 -16.3480759204 1.5689432375
S 9.5636118164 -16.539273575 1.9835968223
O 8.834291595 -15.3024148465 2.2885856267
O 9.5403735941 -17.6263496369 2.9870567642
C 12.9498912641 -17.3071694 -4.6771255152
C 12.2004499766 -18.4516711125 -4.9485523913
C 12.9245237137 -16.7290398486 -3.4150713315
H 12.2230677424 -18.8982267025 -5.9406491754
H 13.5634595591 -15.8709328365 -3.2192619535
C 11.4431677258 -19.0307154635 -3.9318856087
C 12.1470716146 -17.2836732726 -2.3786890709
H 10.8662183931 -19.9342619683 -4.1236452016

C 11.4300706911 -18.4640922017 -2.6650131128
H 10.8391150857 -18.9179691826 -1.8693440436
H 13.5707645717 -16.8665476879 -5.4550411572
C 8.5799002972 -16.3447278637 -0.5744541656
C 8.141424334 -16.8654190565 -1.7845411923
C 8.8350806283 -17.2062594167 0.4951044424
H 7.9527815499 -16.1931881524 -2.6217749938
C 7.939748707 -18.2414826722 -1.9514792049
C 8.6445959041 -18.5778009501 0.3511373212
H 8.8412435435 -19.2337644645 1.1962965365
C 8.1877171501 -19.0832313568 -0.8630893555
H 8.0260951656 -20.1561492237 -0.9723512118
H 8.7222742485 -15.2719835407 -0.448906805
C 7.474144333 -18.789377665 -3.2659199344
H 7.5285509137 -19.8834286076 -3.2905760662
H 6.4335437296 -18.5088814492 -3.4757041241
H 8.0783203947 -18.4044688368 -4.097096006
H 11.6693163074 -17.5018946546 -0.3030748292
H 14.667224247 -16.7714054652 -1.3689571306
C 14.4466532862 -18.7072841776 -0.4237635182
C 14.4127169041 -19.4308219411 0.7537666654
C 14.4791700288 -18.789380204 2.0308335305
C 14.6958090735 -17.3323515095 2.0166683554
C 14.7062331885 -16.6084089055 0.7908137938
C 14.8361889455 -15.202451496 0.839651073
C 14.954329925 -14.5365814422 2.0447919707
C 14.9737442172 -15.2554817502 3.2502324537
C 14.8501257737 -16.6350838656 3.2253511736
H 14.3587180211 -19.2292235565 -1.37808632
H 14.2886607449 -20.5122231417 0.7448933815
O 14.2831673683 -19.3958784435 3.1229592848
H 14.8954963523 -17.2226934263 4.1439573951
H 15.1011402928 -14.730771752 4.1957429189
H 15.0424679206 -13.4509443429 2.0582941549
H 14.8383980037 -14.6446547601 -0.0974458929
K 12.0487542384 -18.0637815434 3.6030374301

TS_A-B_para_neutral_toluene

C 12.213581 -16.798758 -0.94137
C 14.43337 -17.21712 -0.433417
C 11.183248 -13.929956 1.244344
C 11.471358 -12.890531 0.396599
C 12.005389 -13.08826 -0.880502
C 12.278386 -14.336511 -1.359159
C 12.007782 -15.456386 -0.519283
C 11.453717 -15.247771 0.802141
C 11.688447 -10.901954 -0.587554
H 10.738257 -13.769753 2.219607
H 12.662823 -14.465505 -2.364573
H 10.804805 -10.412248 -1.022406
O 11.312059 -11.557586 0.621482
O 12.19141 -11.87551 -1.491349
H 12.470402 -10.163349 -0.369261
N 11.165152 -16.336162 1.611919
S 9.610729 -16.56749 1.96506
O 8.845256 -15.351582 2.270531
O 9.577758 -17.66979 2.954214
C 12.883005 -17.344345 -4.653026
C 12.061848 -18.445595 -4.889332
C 12.961671 -16.782544 -3.384192
H 12.003622 -18.881675 -5.884749
H 13.647067 -15.953301 -3.219156
C 11.33423 -18.994299 -3.835657
C 12.217943 -17.30814 -2.312979
H 10.698707 -19.863543 -3.999871
C 11.422859 -18.440744 -2.56453
H 10.851266 -18.869655 -1.741344
H 13.476921 -16.924049 -5.46266
C 8.675553 -16.361774 -0.61377
C 8.219437 -16.873166 -1.821189
C 8.909351 -17.226912 0.458258
H 8.047023 -16.197868 -2.659522
C 7.973452 -18.242647 -1.982838
C 8.681317 -18.593036 0.316791
H 8.859436 -19.251206 1.164343
C 8.204811 -19.088741 -0.894549

H 8.010973 -20.156834 -0.999827
H 8.841458 -15.292176 -0.491105
C 7.47364 -18.776977 -3.290385
H 7.505189 -19.871907 -3.319464
H 6.43489 -18.475401 -3.479625
H 8.069175 -18.400915 -4.131845
H 11.847878 -17.519331 -0.209704
H 14.671533 -16.671585 -1.344491
C 14.404473 -18.632054 -0.463424
C 14.395798 -19.387992 0.683206
C 14.453199 -18.778675 1.982539
C 14.682667 -17.321604 2.013306
C 14.66166 -16.559285 0.817024
C 14.820886 -15.161994 0.906115
C 14.994985 -14.539765 2.129384
C 15.04184 -15.298144 3.307489
C 14.891147 -16.673839 3.240605
H 14.319872 -19.124137 -1.433581
H 14.294633 -20.470875 0.645497
O 14.252612 -19.414532 3.050016
H 14.960299 -17.293969 4.135822
H 15.212296 -14.808609 4.264883
H 15.105697 -13.45743 2.174996
H 14.79758 -14.571194 -0.009678
K 12.059155 -18.021237 3.669725

InterB_para_neutral_toluene
C 12.788865 -16.910766 -0.751691
C 14.343463 -16.989272 -0.40635
C 11.038036 -14.002898 1.012163
C 11.341975 -13.000304 0.123512
C 12.063611 -13.22908 -1.041679
C 12.521892 -14.485324 -1.356269
C 12.225129 -15.5425 -0.472381
C 11.472422 -15.320917 0.710105
C 11.432746 -11.102056 -1.027654
H 10.460464 -13.816922 1.910929
H 13.083478 -14.665398 -2.271668

H 10.531576 -10.882603 -1.625525
O 11.047982 -11.662233 0.220962
O 12.243586 -12.045367 -1.717994
H 12.009306 -10.18599 -0.853307
N 11.207264 -16.39721 1.575849
S 9.665984 -16.668708 1.92822
O 8.88283 -15.492709 2.337772
O 9.665297 -17.830714 2.854177
C 12.982301 -17.598204 -4.525579
C 11.800632 -18.305983 -4.725547
C 13.324579 -17.151619 -3.250824
H 11.535395 -18.659698 -5.720282
H 14.259644 -16.605143 -3.118478
C 10.964627 -18.560678 -3.640483
C 12.49346 -17.400531 -2.153034
H 10.039981 -19.120495 -3.776961
C 11.309029 -18.111952 -2.370116
H 10.646968 -18.308737 -1.526249
H 13.645684 -17.393095 -5.364282
C 8.627167 -16.334079 -0.603726
C 8.082065 -16.783283 -1.799294
C 8.893437 -17.247228 0.420198
H 7.88353 -16.070158 -2.599785
C 7.775184 -18.135433 -1.995942
C 8.610384 -18.598735 0.239735
H 8.814707 -19.294364 1.050656
C 8.049065 -19.032396 -0.959299
H 7.81481 -20.089265 -1.093112
H 8.838627 -15.276494 -0.454176
C 7.149638 -18.597501 -3.277521
H 7.253964 -19.680121 -3.413919
H 6.074943 -18.371993 -3.304253
H 7.596821 -18.102002 -4.148134
H 12.29852 -17.591772 -0.042818
H 14.858336 -16.364909 -1.152844
C 14.748877 -18.413838 -0.557813
C 14.878686 -19.25442 0.48716
C 14.689406 -18.776007 1.849939

C 14.778994 -17.314099 2.048499
C 14.632523 -16.441227 0.958396
C 14.709313 -15.064283 1.191871
C 14.917555 -14.569999 2.472799
C 15.067949 -15.442134 3.554569
C 15.001295 -16.809246 3.338965
H 14.803419 -18.801694 -1.575294
H 15.04179 -20.321745 0.352545
O 14.432421 -19.534125 2.799485
H 15.150976 -17.520362 4.152259
H 15.254066 -15.049904 4.552534
H 14.969419 -13.494503 2.630515
H 14.588646 -14.374428 0.358334
K 12.132242 -18.16107 3.526663

TS_A-B_ortho_neutral_toluene
C -1.044212584 -0.415091042 0.3659847809
C -1.9565746477 -0.3593801016 -1.6059180781
C -3.9423212685 1.0458081645 -1.5539911981
C 2.0576694808 1.58939151 -0.4072683077
C 1.7772121126 2.6996609471 0.3449979128
C 0.6268816251 2.8163166418 1.1251772182
C -0.3069564668 1.8243162013 1.1580511624
C -0.0853607935 0.6471543226 0.386987841
C 1.1317950033 0.5091846224 -0.3903599404
C 1.7955651442 4.699296498 1.3271284126
H 2.9492235783 1.5555189498 -1.0227781426
H -1.1726070771 1.9071843945 1.8039798656
H 2.4243566533 5.0035768879 2.1733622761
O 2.5397062637 3.8188883045 0.4854374991
O 0.6522283796 4.0103353025 1.8056056667
H 1.4856441156 5.5783352972 0.7411038422
N 1.3260776305 -0.6754701138 -1.0646711931
S 2.7834745479 -0.987089981 -1.7112935398
O 3.266298137 -0.0179761944 -2.7046303014
O 2.7008870071 -2.3977697129 -2.1579149548
C -4.2574880149 0.2452792998 2.2895809251
C -4.5145700464 -1.0251445017 2.7956589058

C -3.1236172666 0.4843133477 1.5204095396
H -5.4065256505 -1.2075802822 3.39222861
H -2.9770852499 1.467989224 1.084674951
C -3.6308355223 -2.0666331742 2.5164639562
C -2.2151931142 -0.5514247122 1.2358677325
H -3.828481598 -3.068298983 2.8942571411
C -2.5046156286 -1.8327831062 1.7408676997
H -1.8321627792 -2.6545225761 1.4953910534
H -4.9560872004 1.058846286 2.4782487827
C 5.1996867368 -0.3639690988 -0.5384143447
C 6.1231724128 -0.3790152307 0.503223865
C 3.9556511011 -0.9575473937 -0.356796258
H 7.0981184524 0.0880688545 0.3638628414
C 5.8214766482 -0.9756825705 1.7295220935
C 3.6302541763 -1.5574540249 0.8595936239
H 2.6434667707 -1.9976121474 0.9976954733
C 4.5582009715 -1.5607681983 1.8910281549
H 4.3010599907 -2.0207785061 2.8454371774
H 5.427286888 0.1113085588 -1.4902693456
C 6.8169055512 -0.9924831901 2.8494117025
H 7.7172673959 -0.4215683397 2.599493295
H 6.3973308361 -0.5680613676 3.7700444849
H 7.1323335376 -2.015662318 3.0917426846
H -0.619074801 -1.3801999142 0.0845204327
H -4.4174328479 2.0251785074 -1.6082313786
C -2.5970575032 0.9115716941 -1.7229302204
C -2.7482927364 -1.5680779908 -1.4859083377
C -4.1852879783 -1.3969378572 -1.2711959962
C -4.7691084669 -0.1023529438 -1.3107604657
C -6.1547761208 0.014495378 -1.0963669405
C -6.9336418459 -1.1024220257 -0.8466490288
C -6.3521337882 -2.3774135033 -0.8080797924
C -4.991853919 -2.5161101292 -1.0220474047
H -1.9752419546 1.78891947 -1.9024517263
H -0.9521997707 -0.4631907672 -2.0202584494
O -2.2202079362 -2.7125982138 -1.4896205976
H -4.5072581169 -3.490347425 -0.9918338926
H -6.9688468373 -3.25133057 -0.6056513018

H -8.0040954329 -0.9889053217 -0.680874523
H -6.6095302915 1.0050939253 -1.1251369867
K 0.2222501076 -3.0750090609 -2.1857919574

TS_A-B_ortho_neutral_toluene_conf2
C -10.480045 17.12583 -1.771509
C -11.269741 17.987538 0.13377
C -9.418035 19.267583 1.051455
C -12.932494 17.976918 -4.510173
C -13.675927 16.827119 -4.479627
C -13.419676 15.776067 -3.592654
C -12.38901 15.835421 -2.704948
C -11.578825 17.006377 -2.662713
C -11.840897 18.107907 -3.583824
C -15.033488 15.137153 -4.974607
H -13.082172 18.714702 -5.294606
H -12.17371 14.990994 -2.060311
H -14.682895 14.523249 -5.819927
O -14.75182 16.511118 -5.257751
O -14.348037 14.777112 -3.79126
H -16.111959 15.015456 -4.827526
N -11.013738 19.174125 -3.52661
S -11.243105 20.426559 -4.524457
O -11.310141 20.092185 -5.956985
O -12.374043 21.276755 -4.045194
C -9.529212 13.961284 0.132386
C -8.145671 13.974596 -0.034756
C -10.315618 14.963919 -0.422739
H -7.532609 13.189147 0.4031
H -11.386108 14.962939 -0.225297
C -7.555036 15.013567 -0.748848
C -9.740436 16.007742 -1.167079
H -6.474074 15.047022 -0.873523
C -8.342135 16.020239 -1.296329
H -7.875596 16.838478 -1.844054
H -10.00052 13.170245 0.713308
C -9.248097 22.065811 -5.358689
C -8.101328 22.834189 -5.203023

C -9.732515 21.330633 -4.277773
H -7.720613 23.407873 -6.048284
C -7.420876 22.878307 -3.981237
C -9.077377 21.360691 -3.049917
H -9.458647 20.77766 -2.214943
C -7.927933 22.130974 -2.913868
H -7.416627 22.153475 -1.951006
H -9.765968 22.014306 -6.313661
C -6.165076 23.682307 -3.831515
H -5.979987 23.955072 -2.786623
H -6.200358 24.605721 -4.421133
H -5.286638 23.119948 -4.176496
H -9.851572 17.991521 -1.981947
H -8.490096 19.326058 1.619216
C -10.09014 18.087586 0.927924
C -11.921817 19.172709 -0.367615
C -11.176329 20.433095 -0.23751
C -9.936863 20.465064 0.464356
C -9.261748 21.697911 0.586342
C -9.784544 22.857852 0.046436
C -11.012087 22.826959 -0.631233
C -11.6923 21.629831 -0.754343
H -9.694658 17.183408 1.390659
H -11.912775 17.115632 0.242747
O -13.064836 19.119458 -0.890459
H -12.640875 21.596716 -1.282496
H -11.418853 23.73611 -1.069647
H -9.241759 23.796918 0.144962
H -8.313222 21.721518 1.124219
K -14.358764 20.082973 -2.839011

TS_A-B_ortho_neutral_toluene_conf3
C 0.888418718 0.1367526169 0.5297143514
C 1.9913011364 -0.2131596639 -1.3099661641
C 3.8072541726 1.396003674 -1.4939639961
C -2.3270054433 1.4267794357 -0.9643015695
C -2.2453893655 2.7261596399 -0.5375350806
C -1.1929878633 3.2030184082 0.2438916211

C -0.1605682999 2.3915751069 0.608503811
C -0.1755969441 1.0306914607 0.1874905922
C -1.2895089058 0.5267105727 -0.5931687056
C -2.5823669215 4.8951769005 -0.1556641308
H -3.1472527604 1.1071168675 -1.5968412066
H 0.6257863895 2.7618387777 1.2549503338
H -3.317268075 5.3253428334 0.536555416
O -3.1387389866 3.7288377588 -0.7614806042
O -1.4152289089 4.5225095193 0.5583509024
H -2.3245526633 5.6197746002 -0.943411696
N -1.2838532635 -0.8104247632 -0.9234001495
S -2.6438342757 -1.4833985385 -1.5027603383
O -3.1760070369 -0.8995956407 -2.7419758292
O -2.3580938872 -2.9371768558 -1.5361208958
C 3.7905277277 1.6940266418 2.4328416432
C 4.1197250233 0.6486477805 3.2900855803
C 2.7289083377 1.5716086022 1.5425397662
H 4.955390791 0.7480957683 3.9804018864
H 2.5317372218 2.3761417551 0.8405648842
C 3.3822493094 -0.533593738 3.2449253036
C 1.9678345036 0.3897108519 1.4877008287
H 3.6383136451 -1.3638374502 3.9008743005
C 2.3296269888 -0.6618450828 2.3503814102
H 1.7742209519 -1.59780299 2.294223521
H 4.3773173277 2.6111872758 2.4409321986
C -5.1937681161 -0.9142118898 -0.6208730117
C -6.174625952 -0.7746340385 0.3572619912
C -3.8970299152 -1.2457954935 -0.2450221808
H -7.1916328872 -0.5131496543 0.0649931546
C -5.8789388986 -0.9563585583 1.7101494469
C -3.5768637103 -1.4303007679 1.1000451918
H -2.5519938481 -1.666799227 1.3836153163
C -4.5629305479 -1.2827985364 2.0647992312
H -4.3113789702 -1.4179074104 3.1169909574
H -5.4189032933 -0.7609709229 -1.6741888249
C -6.9347154028 -0.802565545 2.7622393322
H -7.0875931114 -1.735953768 3.3191261761
H -7.8985713077 -0.5147475108 2.3295850103

H -6.6604853131 -0.0389073142 3.5010192708
H 0.5982041418 -0.9150324413 0.4960675185
H 4.1834350751 2.3789813737 -1.7769717854
C 2.508412112 1.0529111714 -1.7211916741
C 2.8849014534 -1.2388514229 -0.80823199
C 4.265091434 -0.8358098769 -0.5375495452
C 4.7152100577 0.4665745521 -0.8833791006
C 6.0498967044 0.8110013916 -0.6011669944
C 6.905772211 -0.0924897011 0.0051355348
C 6.4559402549 -1.3759112515 0.3452294684
C 5.1488551718 -1.7383026869 0.0702863314
H 1.8235306678 1.7667733201 -2.1793493155
H 1.0517603106 -0.5474047523 -1.753462187
O 2.4810398995 -2.4032458386 -0.5443261941
H 4.7648034353 -2.7236803648 0.3279829161
H 7.1319526288 -2.0799315028 0.8272813204
H 7.9342690753 0.1961236159 0.2176055087
H 6.4021357943 1.8090160104 -0.8635482976
K 0.1833952022 -3.2449690838 -1.3196895602

TS_A-B_ortho_neutral_toluene_conf4

C -1.5502975714 1.3390741395 -0.4179240899
C -0.9071799946 1.8778717473 1.6838361919
C 1.0302034694 3.3153471087 1.4016491216
C -1.805813544 -2.4190137234 -0.6223402476
C -3.1587693896 -2.540484848 -0.4449351518
C -4.0006685775 -1.4432739168 -0.2320508169
C -3.5161742065 -0.1706258607 -0.2118986701
C -2.1167982278 0.0388986781 -0.3813835804
C -1.2312780236 -1.1024494063 -0.5916322533
C -5.2623080595 -3.2575413602 -0.4222121109
H -1.2020112381 -3.2810686874 -0.8961709852
H -4.1928367398 0.6698560834 -0.1077746853
H -5.6826654238 -3.3977685253 -1.4312322812
O -3.8975657831 -3.6880977338 -0.4302582159
O -5.293936819 -1.8895851462 -0.0648997647
H -5.8189159127 -3.8444890284 0.3158662317
N 0.0809348156 -0.8421001714 -0.769016863

S 1.1064638348 -2.0617356996 -1.0317798407
O 0.8664440016 -2.7958565385 -2.2829098521
O 1.2521283499 -2.9388553844 0.1694250415
C -4.0262707306 4.2314753621 -0.2961907915
C -3.4967197817 5.0583848582 -1.2854285404
C -3.4236331512 3.0153374785 0.0023326415
H -3.9698714831 6.0111710555 -1.515162126
H -3.8197862648 2.4166420331 0.8206757747
C -2.3450504837 4.663079496 -1.9606726646
C -2.2747566529 2.5875461494 -0.6849240371
H -1.9098789852 5.3061838185 -2.7236850758
C -1.7388003448 3.4501014554 -1.6552484572
H -0.8297802101 3.1498814659 -2.1759925408
H -4.9091449728 4.5430187656 0.2595024099
C 3.8145203918 -1.8940340359 -0.987131664
C 5.0408280678 -1.2573581298 -1.1416350725
C 2.6384574629 -1.1733906278 -1.1812371132
H 5.9621808746 -1.8174907252 -0.9802556764
C 5.1138664701 0.094505865 -1.4882522466
C 2.6851187792 0.1724976246 -1.5377480057
H 1.7580903697 0.724047865 -1.668638519
C 3.9178584405 0.7936291571 -1.6842684614
H 3.954444512 1.8540698926 -1.9357264922
H 3.76233829 -2.9418321883 -0.6978662635
C 6.4331215907 0.7896978402 -1.6341930918
H 7.2678279338 0.1446731202 -1.339078882
H 6.6121695985 1.1056200028 -2.6702791705
H 6.4776417556 1.6976754023 -1.0184962574
H -0.520534829 1.3506949856 -0.7730330582
H 1.4695887031 4.2964273538 1.223400902
C -0.3222233326 3.1623425473 1.4969299987
C -0.0875148409 0.7322639447 1.9915195293
C 1.3652889382 0.9076282843 1.8462345843
C 1.9059835056 2.1957153205 1.5714020395
C 3.3069761977 2.3413495706 1.506838519
C 4.148215067 1.2628579507 1.7050389811
C 3.6135063737 -0.0054504456 1.9717315419
C 2.2422933091 -0.1673735355 2.0445329037

H -0.9794864129 4.0240976191 1.3794688557
H -1.9477960805 1.8037382048 1.9954886249
O -0.6004476893 -0.3513900862 2.3735782571
H 1.8187560154 -1.1520740657 2.2294630603
H 4.2742140133 -0.8607284099 2.1016858415
H 5.2282743729 1.3951861737 1.6479967596
H 3.7199740591 3.3293907517 1.2995340331
K -0.4869668064 -2.8495988914 2.0953197725

TS_A-B_ortho_neutral_toluene_conf5
C 12.408311 -14.658544 -1.12897
C 13.429101 -16.358262 -2.21947
C 12.819922 -16.052446 -4.551041
C 10.37179 -16.130509 1.684079
C 11.257627 -15.920497 2.707677
C 12.516675 -15.340091 2.518455
C 12.928062 -14.923093 1.289045
C 12.065752 -15.113263 0.170445
C 10.756813 -15.731792 0.358355
C 12.206596 -15.627064 4.696198
H 9.362352 -16.480563 1.88671
H 13.880665 -14.418766 1.173609
H 11.855354 -14.721452 5.216692
O 11.095321 -16.231112 4.026734
O 13.178439 -15.28821 3.72642
H 12.629547 -16.34461 5.407165
N 9.975351 -15.869344 -0.73348
S 8.503232 -16.516923 -0.583223
O 7.578191 -15.728956 0.244448
O 8.563211 -17.964947 -0.219807
C 15.649959 -12.67519 -1.392164
C 15.240858 -11.632768 -2.222122
C 14.752956 -13.666263 -1.01221
H 15.946304 -10.85936 -2.519812
H 15.111943 -14.502656 -0.415013
C 13.92744 -11.602906 -2.683248
C 13.416911 -13.6407 -1.447741
H 13.598088 -10.804367 -3.345825

C 13.033555 -12.599398 -2.30887
H 12.01006 -12.580902 -2.682783
H 16.681975 -12.725832 -1.049123
C 6.917317 -17.322923 -2.632314
C 6.407043 -17.276434 -3.9246
C 7.946074 -16.456766 -2.269715
H 5.609139 -17.961067 -4.213303
C 6.909163 -16.374554 -4.866837
C 8.457565 -15.542682 -3.187989
H 9.273568 -14.888101 -2.893208
C 7.938882 -15.511976 -4.475372
H 8.35773 -14.816326 -5.203206
H 6.538079 -18.040414 -1.907295
C 6.372179 -16.332497 -6.265051
H 5.707445 -17.178482 -6.47111
H 5.79974 -15.414053 -6.451012
H 7.181046 -16.352446 -7.006852
H 11.570484 -14.671395 -1.825037
H 12.976358 -15.583204 -5.521974
C 13.654743 -15.788265 -3.504596
C 12.445944 -17.396944 -2.035874
C 11.537397 -17.645854 -3.164855
C 11.741667 -16.983125 -4.408179
C 10.888021 -17.291674 -5.487059
C 9.870864 -18.217633 -5.352615
C 9.671904 -18.869548 -4.127412
C 10.502882 -18.585055 -3.059655
H 14.48201 -15.089198 -3.628361
H 14.22225 -16.329672 -1.47411
O 12.38499 -18.054985 -0.964833
H 10.347279 -19.075288 -2.101274
H 8.855871 -19.580903 -4.012943
H 9.218491 -18.438565 -6.19687
H 11.046808 -16.78466 -6.439735
K 10.757384 -18.992019 0.713717

TS_A-B_ortho_neutral_toluene_conf6

C 11.9179931236 -15.7703670272 -1.3255781517

C 13.549223333 -16.9335735028 -2.1667551247
C 14.0937569257 -16.0946415308 -4.3852137829
C 13.8367293589 -13.9915448568 1.3927785556
C 13.7807952175 -12.7200489103 0.8854822111
C 13.1162631465 -12.405187664 -0.2998011969
C 12.4996599434 -13.3685219384 -1.040745297
C 12.5312411818 -14.7154164479 -0.5779289923
C 13.1875508864 -15.035095142 0.675769329
C 14.0266218215 -10.5392989912 0.5012789163
H 14.3913640578 -14.1955211711 2.3015016784
H 11.9479453437 -13.1038357216 -1.9346021896
H 13.5152190481 -9.7235489327 1.0277446704
O 14.3086966318 -11.5880932438 1.4273872424
O 13.1852987698 -11.0500522105 -0.519398536
H 14.9723976324 -10.1831056807 0.0644428673
N 13.1334110059 -16.341157813 1.1089360352
S 13.5815026354 -16.686374341 2.6315987103
O 14.9635339572 -16.3379857498 2.9898684818
O 13.1933578079 -18.1036372196 2.8246923859
C 10.41088429 -14.7582192641 -4.6682357201
C 9.2907390431 -15.5830227107 -4.6994455685
C 11.2698616465 -14.7723286468 -3.574239131
H 8.6252076525 -15.5716021892 -5.5606471877
H 12.1711066009 -14.1672772572 -3.6011739979
C 9.037954502 -16.4374537165 -3.627195038
C 11.0271057704 -15.6213950321 -2.4792926773
H 8.1728506161 -17.0980423686 -3.6459251191
C 9.899209677 -16.4609963444 -2.5398878042
H 9.7189820607 -17.1530338589 -1.7175124675
H 10.6327212654 -14.1077407015 -5.5127380157
C 13.0501292515 -15.1203315542 4.8369632892
C 12.2124529424 -14.407002545 5.6900066542
C 12.5171483942 -15.7229882839 3.7031714414
H 12.628441363 -13.93203095 6.5785220352
C 10.8469508638 -14.2808125014 5.4249594642
C 11.1567648217 -15.61013184 3.4164980656
H 10.7556156443 -16.0674992941 2.512890028
C 10.3344235372 -14.8915498309 4.2723507717

H 9.2727213052 -14.7949519876 4.0440960255
H 14.1163662372 -15.2089276775 5.0342356462
C 9.9472888577 -13.5081063513 6.34090163
H 10.5000970202 -13.0758516563 7.1814911427
H 9.4458003271 -12.6857921287 5.814968933
H 9.1545241981 -14.1427868211 6.7572081617
H 11.6736883537 -16.6459121301 -0.7214049316
H 14.6191492178 -15.396344011 -5.0365247287
C 14.269559389 -16.0574087675 -3.0345135399
C 12.7512628558 -18.0119113153 -2.7167499126
C 12.5494275546 -18.0106352438 -4.1656374831
C 13.2187409754 -17.0621996542 -4.984630156
C 12.9896001031 -17.0955670735 -6.3724523959
C 12.1288701901 -18.0253814988 -6.9301692312
C 11.4707312603 -18.9587053825 -6.1170778303
C 11.6859725003 -18.9476618818 -4.7500685224
H 14.9317239063 -15.3159522972 -2.5868693099
H 13.9205362708 -17.0614094157 -1.1483828381
O 12.1919881948 -18.8684872622 -1.9805083571
H 11.1852328307 -19.652863329 -4.0891840474
H 10.7906660996 -19.6837244186 -6.5608174048
H 11.9637792621 -18.0317139407 -8.0067095408
H 13.4995027477 -16.3701881605 -7.0071020915
K 12.768715571 -19.1233746102 0.5042839476

TS_A-B_ortho_neutral_toluene_conf7
C 11.933615 -15.028817 -1.265444
C 13.292997 -16.684073 -1.630191
C 14.081799 -16.612834 -3.931378
C 14.139526 -13.010044 1.035574
C 14.367692 -11.938149 0.21323
C 13.80563 -11.82492 -1.058348
C 13.008643 -12.806168 -1.567522
C 12.742049 -13.956495 -0.770619
C 13.290659 -14.052332 0.568896
C 15.080424 -10.035872 -0.701872
H 14.622312 -13.069828 2.004211
H 12.541742 -12.685692 -2.537552

H 14.743351 -9.024529 -0.441281
O 15.118547 -10.834293 0.480532
O 14.168577 -10.622158 -1.615864
H 16.087541 -10.004098 -1.145419
N 12.9452 -15.152416 1.321562
S 13.311944 -15.174288 2.903729
O 14.738462 -15.047884 3.232701
O 12.622049 -16.374681 3.432628
C 10.782761 -14.681084 -4.873597
C 9.511547 -15.240351 -4.788384
C 11.584426 -14.570555 -3.742324
H 8.890902 -15.329874 -5.678074
H 12.595939 -14.189643 -3.845844
C 9.046643 -15.702341 -3.557898
C 11.129593 -15.022984 -2.490376
H 8.059558 -16.154993 -3.48071
C 9.848365 -15.60214 -2.430085
H 9.496705 -15.993293 -1.475628
H 11.16649 -14.34304 -5.834849
C 13.147216 -13.026952 4.621277
C 12.493322 -11.957228 5.226142
C 12.491915 -13.761308 3.639212
H 13.006165 -11.378666 5.99449
C 11.192046 -11.604439 4.861141
C 11.193207 -13.426989 3.256036
H 10.699339 -13.994973 2.468628
C 10.555384 -12.354534 3.863231
H 9.544041 -12.086138 3.556982
H 14.165724 -13.294963 4.894016
C 10.488397 -10.453755 5.513958
H 11.147768 -9.912705 6.200659
H 10.115098 -9.735167 4.77372
H 9.617003 -10.79024 6.090709
H 11.487939 -15.637622 -0.476735
H 14.770008 -16.242085 -4.690833
C 14.216574 -16.243863 -2.626681
C 12.294465 -17.6847 -1.951854
C 12.14515 -18.037998 -3.363498

C 13.035211 -17.508489 -4.335971
C 12.851834 -17.875104 -5.681929
C 11.825665 -18.726193 -6.054891
C 10.949019 -19.244064 -5.091201
C 11.115148 -18.902028 -3.760598
H 15.011288 -15.559674 -2.328435
H 13.595322 -16.603349 -0.584536
O 11.536044 -18.175238 -1.072687
H 10.448398 -19.280128 -2.987736
H 10.140085 -19.907752 -5.391569
H 11.700201 -18.994983 -7.102864
H 13.531501 -17.472816 -6.4337
K 11.966874 -17.851517 1.434461

TS_A-B_para_neutral_toluene
C 12.213581 -16.798758 -0.94137
C 14.43337 -17.21712 -0.433417
C 11.183248 -13.929956 1.244344
C 11.471358 -12.890531 0.396599
C 12.005389 -13.08826 -0.880502
C 12.278386 -14.336511 -1.359159
C 12.007782 -15.456386 -0.519283
C 11.453717 -15.247771 0.802141
C 11.688447 -10.901954 -0.587554
H 10.738257 -13.769753 2.219607
H 12.662823 -14.465505 -2.364573
H 10.804805 -10.412248 -1.022406
O 11.312059 -11.557586 0.621482
O 12.19141 -11.87551 -1.491349
H 12.470402 -10.163349 -0.369261
N 11.165152 -16.336162 1.611919
S 9.610729 -16.56749 1.96506
O 8.845256 -15.351582 2.270531
O 9.577758 -17.66979 2.954214
C 12.883005 -17.344345 -4.653026
C 12.061848 -18.445595 -4.889332
C 12.961671 -16.782544 -3.384192
H 12.003622 -18.881675 -5.884749

H 13.647067 -15.953301 -3.219156
C 11.33423 -18.994299 -3.835657
C 12.217943 -17.30814 -2.312979
H 10.698707 -19.863543 -3.999871
C 11.422859 -18.440744 -2.56453
H 10.851266 -18.869655 -1.741344
H 13.476921 -16.924049 -5.46266
C 8.675553 -16.361774 -0.61377
C 8.219437 -16.873166 -1.821189
C 8.909351 -17.226912 0.458258
H 8.047023 -16.197868 -2.659522
C 7.973452 -18.242647 -1.982838
C 8.681317 -18.593036 0.316791
H 8.859436 -19.251206 1.164343
C 8.204811 -19.088741 -0.894549
H 8.010973 -20.156834 -0.999827
H 8.841458 -15.292176 -0.491105
C 7.47364 -18.776977 -3.290385
H 7.505189 -19.871907 -3.319464
H 6.43489 -18.475401 -3.479625
H 8.069175 -18.400915 -4.131845
H 11.847878 -17.519331 -0.209704
H 14.671533 -16.671585 -1.344491
C 14.404473 -18.632054 -0.463424
C 14.395798 -19.387992 0.683206
C 14.453199 -18.778675 1.982539
C 14.682667 -17.321604 2.013306
C 14.66166 -16.559285 0.817024
C 14.820886 -15.161994 0.906115
C 14.994985 -14.539765 2.129384
C 15.04184 -15.298144 3.307489
C 14.891147 -16.673839 3.240605
H 14.319872 -19.124137 -1.433581
H 14.294633 -20.470875 0.645497
O 14.252612 -19.414532 3.050016
H 14.960299 -17.293969 4.135822
H 15.212296 -14.808609 4.264883
H 15.105697 -13.45743 2.174996

H 14.79758 -14.571194 -0.009678
K 12.059155 -18.021237 3.669725

TS_A-B_para_neutral_toluene_conf2
C -12.96485 16.008814 0.739989
C -14.65365 17.597033 0.71641
C -11.161613 16.400247 -2.525174
C -11.868721 15.497911 -3.278617
C -12.935353 14.757452 -2.759214
C -13.341283 14.894526 -1.463798
C -12.649523 15.825117 -0.634065
C -11.545139 16.589669 -1.175136
C -12.626307 14.142818 -4.878967
H -10.313785 16.944388 -2.925178
H -14.149761 14.285327 -1.075899
H -12.082485 13.215153 -5.109839
O -11.686885 15.175578 -4.588467
O -13.448171 13.946026 -3.737374
H -13.246749 14.448639 -5.731155
N -10.853317 17.476141 -0.363341
S -9.308973 17.117852 -0.077027
O -8.551138 16.623494 -1.234078
O -8.754426 18.271332 0.669038
C -15.298166 13.447443 2.326112
C -14.639789 13.235154 3.536289
C -14.787238 14.341431 1.392586
H -15.044204 12.534218 4.263989
H -15.348468 14.522988 0.477904
C -13.472768 13.943818 3.813297
C -13.598865 15.049619 1.644341
H -12.953994 13.800445 4.760301
C -12.9689 14.84683 2.88562
H -12.054463 15.399235 3.102404
H -16.225448 12.919545 2.110752
C -9.739173 14.491314 0.618232
C -9.893892 13.447904 1.520699
C -9.381217 15.759497 1.083514
H -10.183123 12.461726 1.156802

C -9.682925 13.637371 2.892576
C -9.174687 15.972368 2.443742
H -8.886102 16.963043 2.787994
C -9.313988 14.910824 3.334865
H -9.136273 15.074319 4.398408
H -9.883341 14.328002 -0.449084
C -9.853301 12.49962 3.852504
H -9.086655 11.727591 3.705473
H -10.825387 12.007185 3.721264
H -9.785135 12.834226 4.893647
H -12.261833 16.677245 1.237254
H -15.318052 16.780836 0.439028
C -14.590162 18.006975 2.069599
C -14.001233 19.188359 2.449169
C -13.448109 20.093988 1.481434
C -13.692406 19.766686 0.063789
C -14.276161 18.52684 -0.303779
C -14.423602 18.23194 -1.674004
C -14.013696 19.127859 -2.645175
C -13.464664 20.364722 -2.279072
C -13.313664 20.674471 -0.937056
H -14.964907 17.322651 2.832344
H -13.889529 19.45259 3.498867
O -12.731607 21.080847 1.792519
H -12.923187 21.6434 -0.62152
H -13.175629 21.081572 -3.045808
H -14.128191 18.87441 -3.698
H -14.86285 17.276679 -1.961857
K -10.577688 20.128053 0.522101

TS_A-B_para_neutral_toluene_conf3

C -12.62546 17.086859 1.385446
C -14.721157 17.837646 1.8651
C -9.928746 19.642397 1.905422
C -9.771992 20.066444 0.610048
C -10.569482 19.591034 -0.434258
C -11.537639 18.652876 -0.226903
C -11.737188 18.168433 1.098308

C -10.948959 18.700389 2.183325
C -9.093313 21.029202 -1.282491
H -9.289333 19.999709 2.705321
H -12.105174 18.258976 -1.062543
H -8.191091 20.662485 -1.793543
O -8.883519 20.977483 0.12795
O -10.199316 20.200484 -1.605704
H -9.309315 22.064544 -1.576674
N -11.211006 18.319469 3.488919
S -10.070647 17.561521 4.323572
O -8.688235 17.819595 3.895643
O -10.402839 17.777636 5.753128
C -13.932076 15.228405 -1.67131
C -13.642514 13.914065 -1.305268
C -13.622454 16.280286 -0.818223
H -13.884215 13.093119 -1.977634
H -13.902933 17.294814 -1.099066
C -13.06209 13.661278 -0.065341
C -13.011104 16.045164 0.425256
H -12.842821 12.63891 0.240036
C -12.763694 14.713085 0.794274
H -12.311993 14.515559 1.765894
H -14.413162 15.434543 -2.625826
C -9.508688 15.082605 3.228801
C -9.787933 13.740253 2.978597
C -10.368604 15.821029 4.034694
H -9.114849 13.160269 2.346808
C -10.916929 13.121547 3.521067
C -11.514144 15.229884 4.571594
H -12.193309 15.826121 5.180237
C -11.777443 13.891225 4.318003
H -12.673216 13.430382 4.736981
H -8.623537 15.563034 2.817756
C -11.212413 11.676474 3.255231
H -12.27747 11.512359 3.048064
H -10.960874 11.047118 4.119569
H -10.640488 11.296368 2.401487
H -12.516232 16.721081 2.406427

H -15.117159 17.119287 1.152137
C -14.66892 19.202252 1.494526
C -14.507828 20.203759 2.415674
C -14.478589 19.929482 3.827396
C -14.725878 18.534238 4.233809
C -14.837634 17.509409 3.261355
C -15.040747 16.184614 3.695902
C -15.146609 15.885034 5.04227
C -15.05785 16.903558 6.003267
C -14.848777 18.209674 5.594054
H -14.685005 19.452146 0.432177
H -14.390663 21.241873 2.111066
O -14.222337 20.81333 4.68471
H -14.820608 19.026638 6.316749
H -15.166548 16.667656 7.060368
H -15.310251 14.855195 5.357353
H -15.111224 15.392166 2.948936
K -12.022391 19.827616 5.70211

TS_A-B_para_neutral_toluene_conf4
C -12.732451 16.29173 0.505243
C -14.782209 17.421084 0.75987
C -11.629383 17.39075 -2.905408
C -12.210185 16.400894 -3.660096
C -12.917216 15.34366 -3.081958
C -13.094023 15.247688 -1.731268
C -12.543314 16.269793 -0.90428
C -11.77459 17.336382 -1.500451
C -12.834376 15.007322 -5.282969
H -11.034082 18.182808 -3.350916
H -13.623566 14.403192 -1.303504
H -12.09604 14.301016 -5.691283
O -12.189468 16.251907 -5.013121
O -13.363794 14.500941 -4.067383
H -13.64871 15.171397 -5.999806
N -11.137416 18.317087 -0.747488
S -9.748203 17.908853 -0.058964
O -9.249074 19.13984 0.59814

O -9.797268 16.679067 0.751598
C -14.21348 13.169288 2.097869
C -13.368686 13.033836 3.197861
C -14.03374 14.213385 1.198405
H -13.511898 12.214534 3.89985
H -14.736242 14.326579 0.37436
C -12.350278 13.96298 3.399258
C -13.004498 15.153116 1.380569
H -11.68748 13.870115 4.257701
C -12.177037 15.016463 2.510415
H -11.370798 15.734458 2.652098
H -15.02683 12.462348 1.943903
C -7.876847 18.566975 -1.983894
C -7.086167 18.290524 -3.092041
C -8.616272 17.544232 -1.39266
H -6.498142 19.088165 -3.547279
C -7.026682 17.001227 -3.635461
C -8.572301 16.253468 -1.915608
H -9.150787 15.463216 -1.439557
C -7.782485 15.992643 -3.030636
H -7.746253 14.982638 -3.439647
H -7.915621 19.56727 -1.557204
C -6.152321 16.708369 -4.816816
H -5.101917 16.590477 -4.517963
H -6.180458 17.518391 -5.555174
H -6.448669 15.782246 -5.321286
H -12.206155 17.10463 0.996696
H -15.260108 16.492538 0.453748
C -14.66506 17.723317 2.135724
C -14.301828 18.971778 2.582249
C -14.052013 20.050529 1.668782
C -14.362328 19.791245 0.250501
C -14.714105 18.487974 -0.189171
C -14.939969 18.280598 -1.564858
C -14.828765 19.319405 -2.471344
C -14.504623 20.610495 -2.031281
C -14.277613 20.835242 -0.683231
H -14.802834 16.914398 2.854738

H -14.139574 19.167026 3.640383
O -13.532068 21.143496 2.017439
H -14.056754 21.834889 -0.305601
H -14.447779 21.431545 -2.744142
H -14.99955 19.133406 -3.53082
H -15.19806 17.278494 -1.908881
K -11.318093 20.763268 0.569095

TS_A-B_para_neutral_toluene_conf5
C -12.732451 16.29173 0.505243
C -14.782209 17.421084 0.75987
C -11.629383 17.39075 -2.905408
C -12.210185 16.400894 -3.660096
C -12.917216 15.34366 -3.081958
C -13.094023 15.247688 -1.731268
C -12.543314 16.269793 -0.90428
C -11.77459 17.336382 -1.500451
C -12.834375 15.007322 -5.282969
H -11.034082 18.182808 -3.350916
H -13.623566 14.403192 -1.303504
H -12.09604 14.301016 -5.691283
O -12.189468 16.251907 -5.013121
O -13.363794 14.500941 -4.067383
H -13.64871 15.171396 -5.999806
N -11.137417 18.317088 -0.747488
S -9.748203 17.908853 -0.058964
O -9.249074 19.13984 0.59814
O -9.797268 16.679067 0.751598
C -14.21348 13.169288 2.097869
C -13.368686 13.033836 3.197862
C -14.03374 14.213385 1.198405
H -13.511897 12.214534 3.89985
H -14.736242 14.326579 0.374361
C -12.350278 13.96298 3.399258
C -13.004498 15.153116 1.380569
H -11.68748 13.870115 4.257701
C -12.177037 15.016464 2.510415
H -11.370798 15.734458 2.652098

H -15.02683 12.462348 1.943903
C -7.876847 18.566975 -1.983894
C -7.086167 18.290524 -3.092041
C -8.616272 17.544233 -1.39266
H -6.498142 19.088165 -3.547279
C -7.026682 17.001227 -3.635461
C -8.572301 16.253468 -1.915608
H -9.150787 15.463216 -1.439557
C -7.782485 15.992643 -3.030636
H -7.746252 14.982638 -3.439646
H -7.915621 19.56727 -1.557204
C -6.152321 16.708369 -4.816816
H -5.101917 16.590476 -4.517963
H -6.180457 17.518391 -5.555174
H -6.44867 15.782247 -5.321287
H -12.206155 17.10463 0.996696
H -15.260108 16.492538 0.453748
C -14.66506 17.723317 2.135723
C -14.301829 18.971778 2.582249
C -14.052013 20.050529 1.668782
C -14.362328 19.791245 0.250501
C -14.714105 18.487974 -0.189171
C -14.939969 18.280598 -1.564858
C -14.828765 19.319405 -2.471344
C -14.504623 20.610495 -2.031281
C -14.277613 20.835242 -0.68323
H -14.802834 16.914398 2.854738
H -14.139574 19.167026 3.640383
O -13.532068 21.143496 2.017439
H -14.056754 21.834889 -0.305601
H -14.447779 21.431545 -2.744142
H -14.99955 19.133406 -3.53082
H -15.19806 17.278494 -1.908881
K -11.318093 20.763268 0.569094

TS_A-B_para_neutral_toluene_conf6

C 1.5663733827 0.2947351011 0.5020372013
C 3.0318982822 -0.3381827124 -1.1209694923

C -1.552643143 1.2205439228 -1.3684852334
C -1.295420182 2.5514598133 -1.5915389181
C -0.1631834615 3.183424088 -1.0754011131
C 0.7712207568 2.5032105011 -0.3465896778
C 0.576347891 1.1107461172 -0.1320315421
C -0.6188376288 0.47184633 -0.6157473039
C -1.3873315993 4.7092793749 -2.1451897703
H -2.4648894893 0.744267343 -1.7163270516
H 1.6358233344 3.0182858153 0.0585593789
H -2.0366485949 5.4101900435 -1.6008337408
O -2.0557279172 3.4548659502 -2.2705697294
O -0.1824446032 4.5104702147 -1.4210118312
H -1.1566306794 5.0970921774 -3.1466070858
N -0.9222258618 -0.8663942847 -0.3718969319
S -1.4601008464 -1.2527186782 1.0845502912
O -1.7985824153 -2.6949254471 1.0113473997
O -0.6133279889 -0.810856187 2.207014227
C 4.3243005417 2.1179867086 2.3870213775
C 4.3158015157 1.4034144342 3.5843811421
C 3.4430684946 1.7848259003 1.3661713762
H 5.0078157621 1.6661437764 4.3824064486
H 3.5021251426 2.3176337575 0.417768637
C 3.4277168099 0.3428758466 3.7447233064
C 2.5297412017 0.7270396907 1.517385368
H 3.4177627317 -0.2259891272 4.6727895231
C 2.5519205385 -0.0003320423 2.7205979771
H 1.8406265148 -0.8148755679 2.8468364204
H 5.0315192783 2.9328707775 2.2425227234
C -4.2059317939 -0.960248743 0.9087139351
C -5.3831544255 -0.2239481777 0.9763227726
C -3.0031557154 -0.3749516955 1.2965885469
H -6.3264139209 -0.6867801126 0.6842262296
C -5.3817390624 1.1039754571 1.4171910233
C -2.9782630605 0.9469371992 1.7407229908
H -2.032507929 1.3914512382 2.0473667937
C -4.1614080659 1.6746584618 1.7945020698
H -4.1413856249 2.7081455089 2.1415491265
H -4.2104469925 -1.9963895338 0.5759961355

C -6.6577330896 1.8841796446 1.5130867992
H -6.495248507 2.9530601899 1.3336114181
H -7.107452348 1.7946414134 2.5114295636
H -7.4055302208 1.5309687776 0.7941551314
H 1.2360433932 -0.7305183594 0.6372744563
H 3.8183272464 0.2654292394 -0.6741592868
C 2.4193273382 0.1183660271 -2.3136827491
C 1.6453154808 -0.696400537 -3.0959139853
C 1.4760424876 -2.0927207552 -2.7872963063
C 2.2789652186 -2.6221504233 -1.6702187893
C 3.052395339 -1.7525512467 -0.8594790542
C 3.7831722708 -2.3005415682 0.213447663
C 3.7555462204 -3.6593234451 0.471363215
C 3.0053712024 -4.5212709281 -0.3428859482
C 2.2793683572 -4.0001412171 -1.4001093631
H 2.4972043237 1.1783452376 -2.5608519498
H 1.1062634078 -0.3105510161 -3.9590185345
O 0.6602680861 -2.8283981236 -3.3975037845
H 1.7221745405 -4.6501901026 -2.0766408166
H 3.0084007734 -5.5927848863 -0.1508074516
H 4.3287684143 -4.0633002689 1.3041390919
H 4.3720582833 -1.6329216423 0.8434559694
K -0.969127395 -3.3939852498 -1.3996232888

Table S9. Computed energies for the neutral transition state conformational analysis in acetonitrile.

	M06-L/BS1							TPSS0-D4/BS2
	1st frequency (cm-1)	Electronic energy	ZPE corr	Thermal corr	Enthalpy corr:	Gibbs corr	Electronic energy in acetonitrile	Electronic energy
pre-ortho TS								
TS_A-B_ortho_2Kcation_acetonitrile	-370.6	-2081.14380	0.48085	0.51632	0.51727	0.40941	-2081.22768	-3225.19162
TS_A-B_ortho_2Kcation_acetonitrile_conf2	-398.5	-2081.10508	0.48016	0.51576	0.51670	0.40968	-2081.20435	-3225.15198
TS_A-B_ortho_2Kcation_acetonitrile_conf3	-352.5	-2081.11886	0.48041	0.51594	0.51688	0.40852	-2081.21139	-3225.16819
TS_A-B_ortho_2Kcation_acetonitrile_conf4	-399.2	-2081.10504	0.48021	0.51582	0.51676	0.40924	-2081.20445	-3225.15199
TS_A-B_ortho_2Kcation_acetonitrile_conf5	-352.6	-2081.11887	0.48036	0.51588	0.51683	0.40860	-2081.21140	-3225.16818
TS_A-B_ortho_2Kcation_acetonitrile_conf6	-317.5	-2081.12382	0.48051	0.51611	0.51705	0.40900	-2081.21213	-3225.17040
TS_A-B_ortho_2Kcation_acetonitrile_conf7	-371.0	-2081.14380	0.48083	0.51627	0.51721	0.41004	-2081.22775	-3225.19155
TS_A-B_ortho_2Kcation_acetonitrile_conf8	-314.7	-2081.14371	0.48058	0.51609	0.51704	0.41010	-2081.22732	-3225.18954
TS_A-B_ortho_2Kcation_acetonitrile_conf9	-358.7	-2081.10983	0.48081	0.51617	0.51712	0.41004	-2081.20974	-3225.16136
TS_A-B_ortho_2Kcation_acetonitrile_conf10	-372.2	-2081.14382	0.48077	0.51625	0.51720	0.40947	-2081.22773	-3225.19152
TS_A-B_ortho_2Kcation_acetonitrile_conf11	-316.7	-2081.14370	0.48057	0.51607	0.51701	0.41001	-2081.22724	-3225.18957
TS_A-B_ortho_2Kcation_acetonitrile_conf12	-350.0	-2081.10171	0.48029	0.51599	0.51694	0.40712	-2081.20095	-3225.15247
TS_A-B_ortho_2Kcation_acetonitrile_conf13	-369.3	-2081.14384	0.48112	0.51648	0.51742	0.41059	-2081.22780	-3225.19164
TS_A-B_ortho_2Kcation_acetonitrile_conf14	-314.9	-2081.14374	0.48050	0.51600	0.51695	0.41032	-2081.22734	-3225.18960
TS_A-B_ortho_2Kcation_acetonitrile_conf15	-359.3	-2081.10976	0.48048	0.51592	0.51686	0.40895	-2081.20960	-3225.16137
TS_A-B_ortho_2Kcation_acetonitrile_conf16	-86.2	-2081.12895	0.47990	0.51567	0.51662	0.40861	-2081.21860	-3225.17255
TS_A-B_ortho_2Kcation_acetonitrile_conf17	-181.2	-2081.12897	0.48036	0.51585	0.51679	0.40970	-2081.21604	-3225.17519
TS_A-B_ortho_2Kcation_acetonitrile_conf18	-370.5	-2081.14383	0.48110	0.51650	0.51745	0.41024	-2081.22776	-3225.19155
TS_A-B_ortho_2Kcation_acetonitrile_conf19	-361.3	-2081.10977	0.48074	0.51613	0.51707	0.40979	-2081.20975	-3225.16135
TS_A-B_ortho_2Kcation_acetonitrile_conf20	-312.5	-2081.14372	0.48040	0.51594	0.51689	0.40974	-2081.22724	-3225.18947
pre-para TS								
TS_A-B_para_2Kcation_acetonitrile	-260.1	-2081.11516	0.48060	0.51575	0.51669	0.41103	-2081.21196	-3225.16542
TS_A-B_para_2Kcation_acetonitrile_conf2	-365.1	-2081.10804	0.48069	0.51613	0.51708	0.40984	-2081.20900	-3225.15672
TS_A-B_para_2Kcation_acetonitrile_conf3	-294.9	-2081.11434	0.48063	0.51610	0.51705	0.41011	-2081.20972	-3225.16049
TS_A-B_para_2Kcation_acetonitrile_conf4	-354.0	-2081.10376	0.48041	0.51595	0.51689	0.40948	-2081.20671	-3225.15231
TS_A-B_para_2Kcation_acetonitrile_conf5	-283.8	-2081.11704	0.48076	0.51556	0.51651	0.41269	-2081.21128	-3225.16768
TS_A-B_para_2Kcation_acetonitrile_conf6	-316.0	-2081.11026	0.48042	0.51600	0.51695	0.40876	-2081.20779	-3225.16058
TS_A-B_para_2Kcation_acetonitrile_conf7	-364.5	-2081.10798	0.48069	0.51615	0.51710	0.40918	-2081.20873	-3225.15665
TS_A-B_para_2Kcation_acetonitrile_conf8	-298.8	-2081.11437	0.48082	0.51620	0.51715	0.41073	-2081.20986	-3225.16058
TS_A-B_para_2Kcation_acetonitrile_conf9	-260.5	-2081.11521	0.48077	0.51576	0.51671	0.41221	-2081.21208	-3225.16547
TS_A-B_para_2Kcation_acetonitrile_conf10	-337.8	-2081.10218	0.48060	0.51608	0.51703	0.40977	-2081.20572	-3225.15017

TS_A-B_para_2Kcation_acetonitrile_conf11	-317.8	-2081.11022	0.48045	0.51604	0.51698	0.40865	-2081.20809	-3225.16083
TS_A-B_para_2Kcation_acetonitrile_conf12	-372.2	-2081.10424	0.48038	0.51597	0.51692	0.40937	-2081.20106	-3225.15315
TS_A-B_para_2Kcation_acetonitrile_conf13	-365.4	-2081.10597	0.48072	0.51641	0.51736	0.40803	-2081.20538	-3225.15607
TS_A-B_para_2Kcation_acetonitrile_conf14	-220.0	-2081.11064	0.48076	0.51645	0.51739	0.40853	-2081.20383	-3225.15834
TS_A-B_para_2Kcation_acetonitrile_conf15	-282.3	-2081.10804	0.48055	0.51598	0.51693	0.40818	-2081.20654	-3225.15990
TS_A-B_para_2Kcation_acetonitrile_conf16	-365.6	-2081.10595	0.48057	0.51632	0.51726	0.40735	-2081.20541	-3225.15609
TS_A-B_para_2Kcation_acetonitrile_conf17	-218.9	-2081.11065	0.48078	0.51645	0.51740	0.40858	-2081.20381	-3225.15833
TS_A-B_para_2Kcation_acetonitrile_conf18	-282.1	-2081.10807	0.48078	0.51615	0.51710	0.40863	-2081.20657	-3225.15995
TS_A-B_para_2Kcation_acetonitrile_conf19	-359.4	-2081.10185	0.48043	0.51617	0.51712	0.40757	-2081.20079	-3225.15252
TS_A-B_para_2Kcation_acetonitrile_conf20	-302.7	-2081.10694	0.48056	0.51635	0.51729	0.40729	-2081.20180	-3225.15755
TS_A-B_para_2Kcation_acetonitrile_conf21	-248.2	-2081.10969	0.48025	0.51564	0.51659	0.40838	-2081.20444	-3225.16128

Table S10. Computed energies for the neutral path in acetonitrile.

	M06-L/BS1							TPSS0-D4/BS2
	1st frequency (cm-1)	Electronic energy	ZPE corr	Thermal corr	Enthalpy corr:	Gibbs corr	Electronic energy in acetonitrile	Electronic energy
Path for ortho product								
InterA_pre-ortho_2Kcation_acetonitrile	8.0	-2081.15403	0.48291	0.51869	0.51963	0.40885	-2081.24202	- 3225.20586
TS_A-B_ortho_2Kcation_acetonitrile	-370.6	-2081.14380	0.48085	0.51632	0.51727	0.40941	-2081.22768	- 3225.19162
InterB_ortho_2Kcation_acetonitrile	17.2	-2081.15456	0.48012	0.51644	0.51738	0.40843	-2081.23760	- 3225.19565
Path for para product								
InterA_pre-para_2Kcation_acetonitrile	18.1	-2081.13249	0.48311	0.51809	0.51904	0.41371	-2081.23049	- 3225.18722
TS_A-B_para_2Kcation_acetonitrile	-260.1	-2081.11516	0.48060	0.51575	0.51669	0.41103	-2081.21196	- 3225.16542
InterB_para_2Kcation_acetonitrile	13.6	-2081.11816	0.47946	0.51576	0.51670	0.40755	-2081.21784	- 3225.16420

InterA_pre-ortho_2Kcation_acetonitrile

C -0.548781 1.066891 -0.32385

C -0.360355 1.666813 1.141502

C 0.687326 3.857744 1.639508

C -4.306456 0.476197 -0.553713

C -4.652371 1.462396 -1.450037
C -3.706821 2.304791 -2.018803
C -2.370543 2.205551 -1.707119
C -1.980853 1.228129 -0.771565
C -2.945426 0.360623 -0.199274
C -5.699764 2.825783 -2.869382
H -5.049733 -0.209285 -0.156087
H -1.64214 2.847047 -2.193912
H -6.013319 2.497834 -3.869856
O -5.889394 1.756407 -1.941383
O -4.315882 3.164779 -2.89005
H -6.286073 3.69597 -2.545506
N -2.489993 -0.683439 0.642416
S -2.977683 -0.796715 2.132736
O -2.902728 0.460453 2.935712
O -2.16462 -1.906871 2.736901
C 1.856999 3.091899 -2.531668
C 2.682704 2.081704 -3.016903
C 0.806492 2.792135 -1.66877
H 3.501565 2.319631 -3.692264
H 0.183411 3.60136 -1.295068
C 2.451765 0.764954 -2.630363
C 0.553107 1.472332 -1.27251
H 3.088975 -0.035525 -3.001111
C 1.400594 0.474039 -1.767306
H 1.223551 -0.562857 -1.468718
H 2.029162 4.124895 -2.827183
C -5.650049 -0.419953 2.69599
C -6.987024 -0.807057 2.685837
C -4.680097 -1.308611 2.240788
H -7.747776 -0.112197 3.039607
C -7.374852 -2.068333 2.225942
C -5.042569 -2.572165 1.769764
H -4.278693 -3.258635 1.40851
C -6.37835 -2.944112 1.770173
H -6.6616 -3.931851 1.407576
H -5.351545 0.56533 3.045065
C -8.812515 -2.48563 2.225391

H -9.479312 -1.647112 2.448653
H -9.110896 -2.90468 1.257297
H -9.001197 -3.264261 2.975592
H -0.416646 -0.003398 -0.139026
H 0.662741 4.946762 1.64392
C -0.356657 3.147654 1.175922
C 0.807412 0.992873 1.810863
C 1.957002 1.793682 2.203178
C 1.887263 3.210876 2.123008
C 2.999991 3.955811 2.540602
C 4.143193 3.326068 3.010073
C 4.209291 1.928498 3.085309
C 3.119272 1.17321 2.690488
H -1.245516 3.646885 0.788712
H -1.260204 1.300722 1.66107
O 0.749228 -0.238765 2.037441
H 3.144528 0.085824 2.731322
H 5.113321 1.4441 3.446834
H 4.996779 3.924342 3.322393
H 2.957695 5.042685 2.489315
K -0.823593 -0.248623 4.443992
K -0.387335 -2.427251 0.795042

TS_A-B_ortho_2Kcation_acetonitrile
C -0.629168 1.074476 -0.266902
C -0.474154 1.98163 1.625647
C 0.922341 3.947388 1.335824
C -4.380596 0.743619 -0.27875
C -4.682303 1.660172 -1.259202
C -3.697688 2.351588 -1.966201
C -2.369284 2.174137 -1.699301
C -2.003225 1.270113 -0.665509
C -3.021019 0.526221 0.029034
C -5.687094 2.961395 -2.764293
H -5.169473 0.186264 0.216278
H -1.618867 2.679543 -2.295539
H -6.1154 2.583937 -3.700719
O -5.906467 1.99629 -1.72862

O -4.284662 3.141035 -2.913874
H -6.154797 3.910974 -2.4734
N -2.605201 -0.429736 0.956207
S -3.312456 -0.5655 2.36928
O -3.490565 0.718445 3.094644
O -2.482266 -1.555239 3.124999
C 1.93197 3.127304 -2.193262
C 2.971899 2.207761 -2.285216
C 0.743431 2.787965 -1.554098
H 3.899499 2.478397 -2.784863
H -0.031934 3.540762 -1.44584
C 2.823895 0.940413 -1.721536
C 0.562774 1.508515 -0.99899
H 3.636209 0.218578 -1.773788
C 1.640331 0.607153 -1.081481
H 1.53581 -0.377354 -0.618317
H 2.047621 4.124672 -2.61253
C -6.019851 -0.700177 2.882427
C -7.280012 -1.277122 2.755373
C -4.936177 -1.285853 2.234555
H -8.131135 -0.817741 3.25649
C -7.478142 -2.430645 1.991148
C -5.109219 -2.431378 1.455989
H -4.261303 -2.862917 0.925161
C -6.371078 -2.994956 1.340913
H -6.508665 -3.885867 0.729065
H -5.870623 0.20609 3.463876
C -8.832418 -3.05682 1.871286
H -9.624902 -2.379236 2.203672
H -9.05165 -3.354311 0.839617
H -8.903169 -3.965822 2.48266
H -0.483435 0.109337 0.213663
H 1.05075 5.00153 1.093103
C -0.315607 3.386199 1.383703
C 0.646598 1.205851 2.123483
C 1.966471 1.803047 2.005995
C 2.095415 3.160804 1.597277
C 3.387505 3.700623 1.467491

C 4.509325 2.932745 1.727902
C 4.377686 1.599193 2.143154
C 3.117677 1.048305 2.286521
H -1.203006 3.978474 1.161945
H -1.460478 1.611914 1.906079
O 0.476642 0.02834 2.575588
H 2.988391 0.015614 2.605965
H 5.264858 1.004132 2.349108
H 5.500124 3.368734 1.615912
H 3.495078 4.738284 1.153261
K -1.27507 0.470096 4.619377
K -0.495735 -2.199034 1.491576

InterB_ortho_2Kcation_acetonitrile
C -0.681146 0.858999 -0.477301
C -0.495873 2.372262 2.03973
C 1.052936 3.99419 1.125386
C -4.36863 1.088691 0.047062
C -4.670191 1.898457 -1.019311
C -3.719914 2.2981 -1.978357
C -2.415497 1.937907 -1.872719
C -2.020432 1.139655 -0.748811
C -3.034247 0.637632 0.183719
C -5.703167 3.131057 -2.56471
H -5.149189 0.786938 0.737019
H -1.694163 2.209359 -2.635305
H -6.344837 2.702488 -3.342798
O -5.880351 2.38117 -1.354441
O -4.341282 3.026659 -2.948438
H -5.959983 4.179793 -2.371969
N -2.613063 -0.233821 1.148545
S -3.432131 -0.407937 2.522499
O -3.763022 0.874987 3.183146
O -2.592904 -1.312514 3.353746
C 1.874091 3.033397 -2.207557
C 3.000804 2.223592 -2.091587
C 0.651781 2.617364 -1.69601
H 3.956712 2.558463 -2.48905

H -0.198407 3.292882 -1.728207
C 2.909843 0.989814 -1.439167
C 0.525657 1.360078 -1.06241
H 3.794518 0.369508 -1.315499
C 1.698298 0.580127 -0.919793
H 1.631561 -0.368268 -0.38262
H 1.952812 4.010985 -2.678733
C -6.114302 -0.865609 2.91188
C -7.30345 -1.551598 2.68906
C -4.969131 -1.25077 2.219067
H -8.202607 -1.250001 3.224625
C -7.370079 -2.616713 1.784967
C -5.009716 -2.302255 1.303238
H -4.116237 -2.571929 0.741553
C -6.205087 -2.974586 1.092473
H -6.242619 -3.789797 0.370845
H -6.070224 -0.026617 3.602265
C -8.647921 -3.364369 1.565982
H -9.518063 -2.78982 1.898324
H -8.792686 -3.621435 0.510955
H -8.651334 -4.309378 2.124678
H -0.523164 0.111962 0.295151
H 1.254881 4.953626 0.651019
C -0.233135 3.614433 1.441745
C 0.540603 1.467754 2.396179
C 1.895102 1.868839 2.048948
C 2.138875 3.121561 1.407832
C 3.46642 3.448149 1.051917
C 4.510118 2.581069 1.308975
C 4.266494 1.349646 1.940659
C 2.977483 1.008559 2.305311
H -1.069281 4.270626 1.198053
H -1.530204 2.068145 2.216754
O 0.305038 0.344652 2.985975
H 2.767934 0.066179 2.810063
H 5.093964 0.673895 2.149776
H 5.52522 2.853446 1.025096
H 3.654713 4.404185 0.562622

K -1.538564 1.012934 4.773827
K -0.395618 -1.91845 1.997149

InterA_pre-para_2Kcation_acetonitrile
C 12.632552 -16.907168 -1.018347
C 14.20741 -17.063252 -0.73849
C 10.533108 -19.617199 0.693672
C 10.357776 -19.022344 1.919535
C 10.913638 -17.78672 2.229882
C 11.669529 -17.085665 1.320401
C 11.836052 -17.637208 0.032665
C 11.287129 -18.91221 -0.281533
C 9.54136 -18.348771 3.838667
H 10.078786 -20.569172 0.436733
H 12.1062 -16.123826 1.58093
H 8.615036 -17.84899 3.51598
O 9.742803 -19.529959 3.06409
O 10.660746 -17.504242 3.576297
H 9.512311 -18.60467 4.900684
N 11.516915 -19.478933 -1.539746
S 10.210875 -19.601484 -2.48308
O 9.042915 -20.194358 -1.815927
O 10.702232 -20.257619 -3.720172
C 10.876976 -13.483144 -0.995172
C 11.602437 -12.796075 -1.960577
C 11.197881 -14.800388 -0.669955
H 11.351556 -11.767282 -2.211391
H 10.581986 -15.324699 0.055615
C 12.6397 -13.450708 -2.622985
C 12.260216 -15.459601 -1.294167
H 13.201078 -12.939961 -3.403155
C 12.956412 -14.762898 -2.293683
H 13.754231 -15.262055 -2.845884
H 10.042474 -12.998331 -0.490635
C 8.782949 -17.271675 -2.169274
C 8.476716 -15.948457 -2.469731
C 9.747451 -17.937679 -2.923455
H 7.716717 -15.42742 -1.88667

C 9.121216 -15.271206 -3.50996
C 10.41248 -17.279919 -3.95772
H 11.155381 -17.816907 -4.545167
C 10.095762 -15.958239 -4.244081
H 10.609449 -15.444802 -5.057114
H 8.264191 -17.803328 -1.373755
C 8.756058 -13.860706 -3.85537
H 9.615502 -13.304801 -4.246599
H 7.975065 -13.831871 -4.626986
H 8.368924 -13.316539 -2.986603
H 12.471428 -17.463534 -1.952652
H 14.699821 -16.453207 -1.507081
C 14.556589 -18.488488 -0.954716
C 14.754554 -19.353715 0.055656
C 14.678956 -18.911467 1.441709
C 14.765105 -17.460295 1.681598
C 14.58776 -16.5567 0.616726
C 14.671747 -15.185285 0.874484
C 14.926851 -14.722022 2.160436
C 15.102673 -15.620915 3.216617
C 15.020848 -16.982772 2.97446
H 14.540127 -18.8352 -1.989435
H 14.921112 -20.41864 -0.102655
O 14.553464 -19.726177 2.374194
H 15.200617 -17.705663 3.770176
H 15.320608 -15.251696 4.21631
H 14.99945 -13.651858 2.342048
H 14.528739 -14.477869 0.058596
K 12.781256 -21.511827 -2.88325
K 12.567843 -19.650217 4.029648
TS_A-B_para_2Kcation_acetonitrile
C 12.267273 -16.852093 -1.108616
C 14.493382 -17.026145 -0.781032
C 10.626639 -19.721715 0.684723
C 10.473166 -19.144467 1.919923
C 10.972247 -17.877282 2.225147
C 11.604981 -17.103403 1.291736
C 11.717418 -17.620224 -0.028882

C 11.282742 -18.964042 -0.318432
C 9.706639 -18.529482 3.881005
H 10.227094 -20.701407 0.442991
H 12.032522 -16.141239 1.559231
H 8.743357 -18.071324 3.610265
O 9.9225 -19.69113 3.071853
O 10.775329 -17.640394 3.587198
H 9.732404 -18.80726 4.938278
N 11.483038 -19.495044 -1.581201
S 10.161582 -19.687336 -2.493303
O 9.020888 -20.270894 -1.778104
O 10.644813 -20.365345 -3.719168
C 11.3506 -13.182236 -0.767
C 11.962563 -12.662186 -1.903937
C 11.433493 -14.540501 -0.478029
H 11.897702 -11.598645 -2.123998
H 10.891927 -14.928446 0.379741
C 12.636006 -13.520421 -2.775911
C 12.139409 -15.417234 -1.32213
H 13.093563 -13.13016 -3.682789
C 12.71253 -14.873626 -2.490654
H 13.226522 -15.543992 -3.18108
H 10.792103 -12.52704 -0.101357
C 8.909331 -17.285195 -2.068319
C 8.661348 -15.945063 -2.340696
C 9.693703 -18.030701 -2.949763
H 8.047389 -15.361609 -1.65417
C 9.185901 -15.326792 -3.481666
C 10.230427 -17.436245 -4.090599
H 10.830801 -18.034155 -4.773578
C 9.968548 -16.096225 -4.350754
H 10.37901 -15.631583 -5.247617
H 8.48767 -17.765708 -1.186715
C 8.897293 -13.887238 -3.774829
H 9.656065 -13.446813 -4.430886
H 7.92813 -13.77043 -4.277724
H 8.855464 -13.288552 -2.857616
H 12.349344 -17.429443 -2.028468

H 14.70201 -16.354615 -1.610929
C 14.631467 -18.421617 -0.976184
C 14.578646 -19.301073 0.072581
C 14.480919 -18.843014 1.432714
C 14.595939 -17.393244 1.651887
C 14.610297 -16.50083 0.549865
C 14.647097 -15.113957 0.796267
C 14.688065 -14.627617 2.090949
C 14.703782 -15.51315 3.179803
C 14.656191 -16.876896 2.955072
H 14.699321 -18.787136 -2.002861
H 14.598047 -20.381027 -0.074176
O 14.303657 -19.638571 2.389706
H 14.690351 -17.581029 3.786155
H 14.761395 -15.124262 4.194343
H 14.722847 -13.554152 2.265218
H 14.64379 -14.426385 -0.04977
K 12.94458 -21.31462 -2.97105
K 12.616473 -19.916363 4.227987

InterB_para_2Kcation_acetonitrile
C 12.1057023318 -16.753639233 -1.3016527623
C 14.8222234797 -17.1705993094 -0.714734865
C 10.7512257826 -19.6130461701 0.6825901945
C 10.6530678912 -18.9884709608 1.902296521
C 11.0507222643 -17.6653087224 2.1176891452
C 11.5592543374 -16.9000147072 1.1137846272
C 11.6556341922 -17.4781236076 -0.1922277455
C 11.2939284041 -18.8745222664 -0.3951932279
C 10.2029761705 -18.4264656227 4.0159057848
H 10.3812676279 -20.6189151681 0.4986893608
H 11.9588327025 -15.9109895816 1.3168837617
H 9.1480712794 -18.1438240408 4.1460885256
O 10.2619773701 -19.5410426159 3.0968694301
O 10.9397145488 -17.3675034173 3.4512119175
H 10.6527832299 -18.7231020065 4.9683132822
N 11.4243396938 -19.439651248 -1.6441917918
S 10.0476844581 -19.7601042894 -2.4356408107

O 9.0198402216 -20.4176950668 -1.6226424191
O 10.4792074359 -20.4118514742 -3.6937104276
C 11.6697100822 -13.0122608006 -0.9856993933
C 12.450586006 -12.5789403161 -2.0551973003
C 11.5394776231 -14.3675386072 -0.7108039759
H 12.5519539021 -11.5159540433 -2.2631986329
H 10.8699732928 -14.6853647701 0.0837103178
C 13.0799872767 -13.5177962053 -2.8769989049
C 12.1996860094 -15.3325211359 -1.5012850505
H 13.6716353492 -13.1884922199 -3.7285499095
C 12.9436879849 -14.8689590284 -2.6096750875
H 13.4310210794 -15.6041891894 -3.2514874832
H 11.1438886536 -12.2873150327 -0.3678271932
C 8.7044811685 -17.4313183478 -1.9023513058
C 8.4459050357 -16.0787498399 -2.1023048973
C 9.4522233117 -18.1326832857 -2.8465575154
H 7.8642667767 -15.5282339773 -1.3628437703
C 8.9257394216 -15.4063633503 -3.2304033603
C 9.9279408173 -17.4857275814 -3.9881276367
H 10.4981165705 -18.0495968645 -4.7245484024
C 9.6558836496 -16.1378890894 -4.1768762283
H 10.0257656598 -15.6333494462 -5.0697489337
H 8.3331927266 -17.9476920985 -1.0183438406
C 8.6715487473 -13.9445054051 -3.4296268093
H 9.5933446709 -13.4109538347 -3.6931145594
H 7.9587129694 -13.7674404017 -4.2452415448
H 8.2606313054 -13.4778719428 -2.5281409205
H 12.2931917604 -17.3621741902 -2.185632732
H 15.1115739578 -16.5435800055 -1.5566611262
C 14.7002307847 -18.5489829173 -0.858491566
C 14.3197400253 -19.3586051787 0.2020823397
C 14.1011811964 -18.8282622437 1.5102309819
C 14.3876867814 -17.4035656798 1.6932139553
C 14.7004053277 -16.5819134978 0.5717500512
C 14.829427971 -15.1874575769 0.7676064691
C 14.6939798064 -14.6352444487 2.0256002338
C 14.436370429 -15.4581732717 3.1368599589
C 14.281760563 -16.8204377564 2.9664345535

H 14.8870489921 -18.9875528767 -1.8425060921
H 14.1586916719 -20.4297399569 0.0631617752
O 13.6577081828 -19.5349768067 2.4663790148
H 14.0476032579 -17.4714319525 3.8060664121
H 14.3501479698 -15.0171930111 4.1279679002
H 14.7983746304 -13.5602140298 2.1597936791
H 15.0395243877 -14.5526466291 -0.0941106451
K 13.0474032572 -20.6556795505 -3.4370122261
K 12.2598615343 -21.5056150977 3.1028169004

TS_A-B_ortho_2Kcation_acetonitrile
C -0.629168 1.074476 -0.266902
C -0.474154 1.98163 1.625647
C 0.922341 3.947388 1.335824
C -4.380596 0.743619 -0.27875
C -4.682303 1.660172 -1.259202
C -3.697688 2.351588 -1.966201
C -2.369284 2.174137 -1.699301
C -2.003225 1.270113 -0.665509
C -3.021019 0.526221 0.029034
C -5.687094 2.961395 -2.764293
H -5.169473 0.186264 0.216278
H -1.618867 2.679543 -2.295539
H -6.1154 2.583937 -3.700719
O -5.906467 1.99629 -1.72862
O -4.284662 3.141035 -2.913874
H -6.154797 3.910974 -2.4734
N -2.605201 -0.429736 0.956207
S -3.312456 -0.5655 2.36928
O -3.490565 0.718445 3.094644
O -2.482266 -1.555239 3.124999
C 1.93197 3.127304 -2.193262
C 2.971899 2.207761 -2.285216
C 0.743431 2.787965 -1.554098
H 3.899499 2.478397 -2.784863
H -0.031934 3.540762 -1.44584
C 2.823895 0.940413 -1.721536
C 0.562774 1.508515 -0.99899

H 3.636209 0.218578 -1.773788
C 1.640331 0.607153 -1.081481
H 1.53581 -0.377354 -0.618317
H 2.047621 4.124672 -2.61253
C -6.019851 -0.700177 2.882427
C -7.280012 -1.277122 2.755373
C -4.936177 -1.285853 2.234555
H -8.131135 -0.817741 3.25649
C -7.478142 -2.430645 1.991148
C -5.109219 -2.431378 1.455989
H -4.261303 -2.862917 0.925161
C -6.371078 -2.994956 1.340913
H -6.508665 -3.885867 0.729065
H -5.870623 0.20609 3.463876
C -8.832418 -3.05682 1.871286
H -9.624902 -2.379236 2.203672
H -9.05165 -3.354311 0.839617
H -8.903169 -3.965822 2.48266
H -0.483435 0.109337 0.213663
H 1.05075 5.00153 1.093103
C -0.315607 3.386199 1.383703
C 0.646598 1.205851 2.123483
C 1.966471 1.803047 2.005995
C 2.095415 3.160804 1.597277
C 3.387505 3.700623 1.467491
C 4.509325 2.932745 1.727902
C 4.377686 1.599193 2.143154
C 3.117677 1.048305 2.286521
H -1.203006 3.978474 1.161945
H -1.460478 1.611914 1.906079
O 0.476642 0.02834 2.575588
H 2.988391 0.015614 2.605965
H 5.264858 1.004132 2.349108
H 5.500124 3.368734 1.615912
H 3.495078 4.738284 1.153261
K -1.27507 0.470096 4.619377
K -0.495735 -2.199034 1.491576

TS_A-B_ortho_2Kcation_acetonitrile_conf2

C -1.526345 1.328245 -0.253308
C -0.859197 1.750393 1.658744
C 1.009794 3.315176 1.384085
C -1.860289 -2.398646 -0.772948
C -3.226259 -2.492839 -0.694563
C -4.053028 -1.397538 -0.406172
C -3.537339 -0.148438 -0.226788
C -2.127395 0.029684 -0.314349
C -1.259601 -1.109902 -0.567169
C -5.348415 -3.148221 -0.8446
H -1.258716 -3.245416 -1.093624
H -4.196142 0.700679 -0.081277
H -5.739324 -3.186319 -1.871491
O -3.988508 -3.604369 -0.852387
O -5.358403 -1.812992 -0.365948
H -5.935599 -3.784473 -0.175518
N 0.071114 -0.865744 -0.57164
S 1.115747 -2.092116 -0.770756
O 0.873106 -2.935563 -1.946927
O 1.26386 -2.845685 0.512949
C -4.056489 4.178049 -0.141836
C -3.612072 4.952464 -1.212173
C -3.401488 2.996577 0.187315
H -4.126621 5.875722 -1.46925
H -3.739586 2.42301 1.050469
C -2.497933 4.543805 -1.940012
C -2.288616 2.561724 -0.548125
H -2.136019 5.14623 -2.770577
C -1.836576 3.367966 -1.601522
H -0.958259 3.055895 -2.166008
H -4.914887 4.499666 0.444586
C 3.817645 -1.937758 -0.840211
C 5.038821 -1.317692 -1.071102
C 2.638055 -1.214606 -1.016746
H 5.961576 -1.880158 -0.929953
C 5.105817 0.018558 -1.483246
C 2.676234 0.113475 -1.432567

H 1.747604 0.664392 -1.557133
C 3.907105 0.717923 -1.658202
H 3.940055 1.762607 -1.968114
H 3.773753 -2.975114 -0.513905
C 6.424079 0.679541 -1.744518
H 7.228498 0.237909 -1.145993
H 6.718332 0.576321 -2.79725
H 6.389371 1.75398 -1.530082
H -0.522448 1.337734 -0.679602
H 1.385286 4.321694 1.206991
C -0.325613 3.081819 1.49363
C 0.053645 0.703741 2.059504
C 1.481568 0.933131 1.825272
C 1.952149 2.244662 1.525524
C 3.344103 2.459881 1.446602
C 4.241376 1.429157 1.656394
C 3.77628 0.134724 1.945123
C 2.415969 -0.102607 2.031631
H -1.030459 3.904863 1.388629
H -1.856019 1.661711 2.09407
O -0.335229 -0.318859 2.71681
H 2.040354 -1.114756 2.185763
H 4.482038 -0.686811 2.051532
H 5.311472 1.615838 1.581324
H 3.70554 3.462035 1.217
K -0.852101 -2.784266 2.035204
K 1.22108 0.643443 4.779386

TS_A-B_ortho_2Kcation_acetonitrile_conf3
C -1.3077120453 1.4589362892 -0.0144325562
C -0.5162327122 2.2934169067 1.735366448
C 1.5087849476 3.2649223905 0.8447617878
C 1.0518635033 2.7767744572 -2.6296176511
C 1.8083351849 1.6557235026 -2.8451971951
C 1.5236665557 0.4322611006 -2.234269725
C 0.4933675326 0.2982046178 -1.3510505356
C -0.2888565363 1.441912094 -1.0350945161
C -0.0365169097 2.6905268723 -1.719490979

C 3.362993715 0.187721614 -3.4478594502
H 1.2396675608 3.692718445 -3.1799229414
H 0.2947824836 -0.6686332123 -0.9063446497
H 3.5330198066 -0.3090915996 -4.4080796573
O 2.8690887922 1.5114564539 -3.6847254895
O 2.3882957451 -0.5228465736 -2.7073039236
H 4.2983807849 0.2585614355 -2.8683989875
N -0.8962195778 3.7225778804 -1.4531127234
S -0.5518791954 5.2080928962 -1.864190056
O -0.5631958814 5.490877412 -3.3334045618
O 0.685046528 5.7733222542 -1.2355368952
C -2.3839780924 -2.0503679423 1.073727247
C -3.7691682107 -1.8848486829 1.1299758173
C -1.5512151353 -0.9816135024 0.7458719238
H -4.417151923 -2.7305380962 1.3534054398
H -0.4760833611 -1.1320647924 0.7664047832
C -4.3133638533 -0.6231453727 0.8739115448
C -2.0817111368 0.2849416037 0.4257415495
H -5.3919128162 -0.4755576098 0.901356768
C -3.4788395535 0.4402191136 0.5375893733
H -3.914053468 1.4125234201 0.3051846841
H -1.9430855404 -3.0246014888 1.2793516298
C -1.7455959684 6.9052742497 -0.0658733809
C -2.8363329905 7.5916910038 0.4622526844
C -1.9345792833 6.0679909819 -1.160018738
H -2.6935508616 8.2478931175 1.3204617018
C -4.1125098582 7.4573597923 -0.090568112
C -3.1978680498 5.9149079468 -1.7320470103
H -3.3261745256 5.261264992 -2.5923053853
C -4.2735228966 6.6060662127 -1.1933508376
H -5.2619738145 6.4911684556 -1.6378331929
H -0.7508008127 7.0107186979 0.3621166651
C -5.2794406973 8.216536565 0.4621361727
H -5.0503821918 8.6639663249 1.4346908907
H -5.5779902096 9.0319410206 -0.2094492324
H -6.1600674712 7.5750189458 0.5842053751
H -1.9474664075 2.3340933786 -0.129474103
H 2.0468549157 4.0977097282 0.3953515368

C 0.2290592068 3.4289459018 1.276380313
C 0.2059675628 1.1279076956 2.2251057009
C 1.5366554251 0.929004472 1.6614794596
C 2.1602711656 1.9895835197 0.9470293979
C 3.4203503384 1.7493196366 0.3690882536
C 4.0323672363 0.511363242 0.4834202887
C 3.4150148992 -0.5270869402 1.1940054857
C 2.1837309801 -0.3077853162 1.7888182574
H -0.2723985388 4.3868167145 1.1608557368
H -1.4754998822 2.4784164861 2.2273538932
O -0.3267537638 0.2957383245 3.0011713252
H 1.683302367 -1.0914782195 2.3562588475
H 3.9031907527 -1.4953905786 1.2788477627
H 5.0068802901 0.3457906474 0.0258806588
H 3.9101145537 2.555751279 -0.1779013533
K 1.6807201971 6.8812518563 -3.4562283585
K -2.6958498584 -0.2556310211 3.6973457941

TS_A-B_ortho_2Kcation_acetonitrile_conf4
C 12.478656 -14.768619 -1.139634
C 13.31565 -16.374161 -2.138534
C 12.842863 -16.012912 -4.51796
C 10.325429 -15.943785 1.734505
C 11.177263 -15.636907 2.76425
C 12.466502 -15.123409 2.563019
C 12.935104 -14.856527 1.311117
C 12.097629 -15.130788 0.192544
C 10.776934 -15.707606 0.390764
C 12.068878 -15.163535 4.748135
H 9.29988 -16.247537 1.928639
H 13.903553 -14.38714 1.177418
H 11.741467 -14.203424 5.172164
O 10.952512 -15.783177 4.094736
O 13.080073 -14.950271 3.776403
H 12.442976 -15.83224 5.529214
N 10.068948 -15.996899 -0.72528
S 8.628432 -16.734713 -0.587969
O 7.668191 -16.069297 0.300321

O 8.828606 -18.190012 -0.305777
C 15.750047 -12.819583 -1.314107
C 15.341211 -11.701289 -2.038604
C 14.840011 -13.823966 -1.004262
H 16.053998 -10.915689 -2.279529
H 15.185726 -14.708554 -0.469169
C 14.018936 -11.603534 -2.463073
C 13.5 -13.732293 -1.410144
H 13.69153 -10.739811 -3.038013
C 13.112779 -12.615307 -2.162728
H 12.081277 -12.541448 -2.506152
H 16.785871 -12.91327 -0.994028
C 6.984244 -17.492871 -2.598548
C 6.424795 -17.412962 -3.867251
C 8.018422 -16.624205 -2.249994
H 5.618914 -18.093107 -4.142522
C 6.877134 -16.471995 -4.79978
C 8.482666 -15.674577 -3.155912
H 9.293797 -15.010012 -2.870001
C 7.911669 -15.609702 -4.42136
H 8.284152 -14.877572 -5.138094
H 6.631521 -18.230988 -1.880702
C 6.261799 -16.378972 -6.162037
H 5.841992 -17.337243 -6.487281
H 5.440425 -15.650541 -6.177809
H 6.988493 -16.051561 -6.914612
H 11.622402 -14.709318 -1.812756
H 13.061485 -15.52002 -5.463869
C 13.619422 -15.790824 -3.423753
C 12.377973 -17.473214 -2.100213
C 11.47763 -17.628127 -3.245204
C 11.737719 -16.923263 -4.456528
C 10.936096 -17.20078 -5.583544
C 9.9212 -18.137925 -5.527587
C 9.663318 -18.830259 -4.331969
C 10.435643 -18.577615 -3.212199
H 14.460604 -15.102462 -3.486003
H 14.136152 -16.465943 -1.425023

O 12.416239 -18.362203 -1.185036
H 10.202753 -19.055118 -2.25986
H 8.828469 -19.525974 -4.274539
H 9.309212 -18.330087 -6.40742
H 11.132635 -16.661475 -6.509931
K 11.019335 -18.77035 0.980905
K 13.105948 -20.125313 -3.19212

TS_A-B_ortho_2Kcation_acetonitrile_conf5
C 12.545597 -14.973026 -1.440478
C 13.445786 -16.289465 -2.797756
C 12.18539 -15.747368 -4.786833
C 10.337105 -12.931968 -3.698286
C 9.125322 -13.504903 -3.417124
C 8.971889 -14.48616 -2.434802
C 10.036288 -14.959053 -1.725454
C 11.331172 -14.452113 -2.018212
C 11.479522 -13.38871 -2.986749
C 7.005059 -14.167337 -3.406712
H 10.418909 -12.11208 -4.404205
H 9.877284 -15.710364 -0.962231
H 6.103123 -13.652653 -3.061398
O 7.913089 -13.205617 -3.957387
O 7.641883 -14.802494 -2.313869
H 6.763128 -14.909876 -4.185284
N 12.736768 -12.869347 -3.141462
S 13.106403 -11.960544 -4.379249
O 12.491242 -10.596959 -4.384787
O 12.942414 -12.610072 -5.719274
C 11.909365 -17.153026 1.641605
C 12.910758 -16.684787 2.494461
C 11.783742 -16.65162 0.346991
H 12.983398 -17.055439 3.515428
H 11.02975 -17.078312 -0.307786
C 13.804336 -15.717326 2.026326
C 12.637296 -15.634387 -0.12699
H 14.584953 -15.330419 2.67958
C 13.670317 -15.212165 0.735129

H 14.350081 -14.432959 0.389403
H 11.210416 -17.912872 1.987908
C 15.759246 -12.330348 -4.983256
C 17.122358 -12.136737 -4.772279
C 14.846884 -11.745574 -4.111807
H 17.841078 -12.594553 -5.451485
C 17.586862 -11.363191 -3.705543
C 15.282714 -10.96755 -3.03869
H 14.555078 -10.511807 -2.370409
C 16.644093 -10.785337 -2.842966
H 16.98915 -10.17731 -2.006881
H 15.396067 -12.932115 -5.813842
C 19.052266 -11.136266 -3.494184
H 19.660924 -11.80637 -4.109809
H 19.337321 -10.107969 -3.751693
H 19.339304 -11.28614 -2.446706
H 13.368921 -14.274418 -1.590348
H 12.04514 -15.163024 -5.694554
C 13.330847 -15.624579 -4.06277
C 12.620797 -17.461157 -2.541091
C 11.3509 -17.504604 -3.257591
C 11.133214 -16.622362 -4.352296
C 9.877413 -16.648703 -4.986204
C 8.876517 -17.501566 -4.549035
C 9.100043 -18.372837 -3.474148
C 10.33559 -18.379916 -2.848432
H 14.108521 -14.931835 -4.375195
H 14.429102 -16.306611 -2.319037
O 12.937038 -18.319951 -1.680255
H 10.543985 -19.052209 -2.017
H 8.308489 -19.039201 -3.138696
H 7.909888 -17.502731 -5.051164
H 9.699619 -15.979144 -5.828546
K 11.710234 -10.561025 -6.911978
K 14.52261 -18.395883 0.289599

TS_A-B_ortho_2Kcation_acetonitrile_conf6

C 12.303448 -14.576871 -1.235801

C 13.517791 -16.111843 -2.254805
C 12.869754 -15.993157 -4.591297
C 15.194024 -12.63369 0.210973
C 15.095633 -11.48841 -0.547213
C 14.110309 -11.314851 -1.51604
C 13.174762 -12.281202 -1.763931
C 13.23896 -13.499365 -1.035685
C 14.249263 -13.668147 -0.023729
C 15.247245 -9.41341 -1.337061
H 15.856111 -12.685911 1.070097
H 12.37508 -12.095703 -2.470757
H 14.784213 -8.638799 -0.708448
O 15.880811 -10.380571 -0.48339
O 14.257039 -10.082598 -2.099404
H 15.997993 -8.980142 -2.00565
N 14.203389 -14.826746 0.723112
S 15.465524 -15.484534 1.364413
O 16.431238 -14.637412 2.122565
O 16.24934 -16.310926 0.354308
C 9.726555 -13.994482 -3.966698
C 8.638391 -14.669732 -3.423248
C 10.93243 -13.929531 -3.276453
H 7.697554 -14.717819 -3.967705
H 11.783328 -13.449036 -3.749388
C 8.762725 -15.29523 -2.181764
C 11.074523 -14.540607 -2.015423
H 7.920459 -15.835467 -1.75453
C 9.965436 -15.239907 -1.496491
H 10.071853 -15.749279 -0.538117
H 9.643098 -13.523439 -4.944264
C 15.484911 -17.019055 3.616015
C 14.971122 -17.975299 4.484473
C 14.737364 -16.634377 2.50297
H 15.548112 -18.265547 5.361759
C 13.718136 -18.560408 4.263539
C 13.481989 -17.196823 2.258397
H 12.905537 -16.888902 1.386996
C 12.990434 -18.158557 3.135393

H 12.008132 -18.592619 2.950201
H 16.447657 -16.549863 3.806576
C 13.155079 -19.557023 5.227822
H 13.940286 -20.149462 5.709107
H 12.601094 -19.052571 6.030175
H 12.454829 -20.244934 4.742757
H 12.254848 -15.255178 -0.383117
H 12.972897 -15.56744 -5.588931
C 13.697046 -15.609562 -3.578194
C 12.621039 -17.22432 -2.011373
C 11.709334 -17.58256 -3.093513
C 11.836482 -16.965341 -4.367581
C 10.931355 -17.327906 -5.381066
C 9.935663 -18.260809 -5.144556
C 9.817636 -18.869971 -3.887555
C 10.701956 -18.534448 -2.877487
H 14.460944 -14.850968 -3.753427
H 14.298698 -15.958906 -1.508161
O 12.597386 -17.821447 -0.898326
H 10.627727 -18.981271 -1.887713
H 9.029677 -19.598574 -3.708284
H 9.241853 -18.523552 -5.941029
H 11.019846 -16.857376 -6.36017
K 18.141903 -14.318949 0.089421
K 14.919979 -18.616961 -0.127012

TS_A-B_ortho_2Kcation_acetonitrile_conf7
C 11.496009 -15.406472 -1.878568
C 13.240844 -16.579803 -1.907532
C 14.175062 -16.36509 -4.139035
C 12.313712 -13.527178 1.280112
C 12.538351 -12.279402 0.745907
C 12.374949 -12.006441 -0.612786
C 12.008982 -12.980892 -1.498285
C 11.806875 -14.298515 -1.005991
C 11.930468 -14.563653 0.403095
C 12.961846 -10.11666 0.412251
H 12.403482 -13.693472 2.348946

H 11.836542 -12.73736 -2.539928
H 12.261613 -9.313976 0.673213
O 12.880634 -11.147243 1.403969
O 12.603286 -10.67954 -0.843194
H 13.992359 -9.740447 0.370727
N 11.614638 -15.847551 0.849433
S 12.528138 -16.590073 1.912228
O 13.982436 -16.546507 1.613341
O 11.988552 -17.984519 1.983934
C 11.222527 -14.541987 -5.574736
C 10.171767 -15.373495 -5.9491
C 11.670171 -14.518749 -4.257407
H 9.826824 -15.389032 -6.980676
H 12.527915 -13.903521 -4.001639
C 9.570355 -16.196564 -4.996936
C 11.064971 -15.323785 -3.275812
H 8.757382 -16.861205 -5.281442
C 10.021863 -16.174479 -3.686564
H 9.566596 -16.838639 -2.947474
H 11.709341 -13.911558 -6.316009
C 13.452303 -15.669821 4.342508
C 13.276875 -15.142084 5.618294
C 12.336068 -15.903702 3.54489
H 14.148936 -14.952098 6.242753
C 12.004203 -14.842757 6.112958
C 11.055262 -15.601405 4.010087
H 10.191928 -15.751532 3.362518
C 10.897365 -15.077061 5.284321
H 9.898868 -14.83401 5.64623
H 14.444166 -15.885642 3.953498
C 11.819427 -14.290137 7.491737
H 12.76472 -13.945468 7.922037
H 11.117111 -13.448825 7.500252
H 11.409059 -15.049013 8.170447
H 10.994595 -16.210122 -1.343157
H 14.802264 -15.852911 -4.867676
C 14.128129 -15.952907 -2.84383
C 12.620022 -17.848119 -2.242878

C 12.629415 -18.236688 -3.64353
C 13.390926 -17.484076 -4.582109
C 13.357762 -17.870639 -5.93378
C 12.60299 -18.955404 -6.345728
C 11.864571 -19.702328 -5.415699
C 11.887708 -19.346996 -4.079699
H 14.704959 -15.089025 -2.514597
H 13.401706 -16.404192 -0.843613
O 12.035655 -18.545279 -1.353191
H 11.322756 -19.909249 -3.338143
H 11.276665 -20.555359 -5.747818
H 12.587348 -19.234261 -7.397531
H 13.935848 -17.298717 -6.658911
K 9.97784 -18.049421 0.261796
K 14.068671 -18.970239 0.421833

TS_A-B_ortho_2Kcation_acetonitrile_conf8
C 11.670667 -15.375224 -2.036144
C 13.61527 -16.419066 -1.777687
C 14.788455 -16.196231 -3.899627
C 9.147374 -17.825948 -0.678152
C 9.068497 -17.340877 0.603533
C 9.788341 -16.224547 1.039257
C 10.622272 -15.555998 0.196096
C 10.771406 -16.021333 -1.143102
C 10.005734 -17.165055 -1.609262
C 8.466577 -16.888463 2.701172
H 8.504576 -18.647081 -0.980319
H 11.141075 -14.665245 0.530499
H 7.518081 -16.348788 2.829934
O 8.319086 -17.828036 1.623799
O 9.501846 -15.983141 2.355163
H 8.737645 -17.430368 3.612973
N 10.100543 -17.503372 -2.937617
S 9.37067 -18.841669 -3.474865
O 9.646209 -20.052902 -2.660067
O 9.729996 -18.945947 -4.902357
C 13.266804 -12.148344 -0.792208

C 13.082569 -11.330676 -1.906048
C 12.815837 -13.462544 -0.800879
H 13.438188 -10.302951 -1.895453
H 13.024109 -14.097172 0.058702
C 12.458537 -11.845952 -3.03856
C 12.162038 -13.993916 -1.925243
H 12.320505 -11.222295 -3.919311
C 12.017596 -13.164215 -3.047984
H 11.529697 -13.563283 -3.939012
H 13.778655 -11.763315 0.087179
C 6.770003 -19.711676 -3.335953
C 5.393335 -19.530752 -3.335261
C 7.605368 -18.595845 -3.395299
H 4.739205 -20.400823 -3.288676
C 4.828231 -18.249858 -3.39047
C 7.069364 -17.311489 -3.448437
H 7.730921 -16.447081 -3.4717
C 5.688522 -17.148216 -3.440707
H 5.267153 -16.144083 -3.470344
H 7.202205 -20.70867 -3.285821
C 3.341949 -18.071891 -3.405249
H 2.851949 -18.693044 -2.646832
H 3.054033 -17.031115 -3.227187
H 2.918284 -18.367098 -4.373782
H 11.542003 -15.689213 -3.070788
H 15.451168 -15.646211 -4.566167
C 14.496066 -15.720148 -2.654618
C 13.187299 -17.761341 -2.08682
C 13.440733 -18.225604 -3.462045
C 14.263713 -17.45516 -4.337444
C 14.523873 -17.958055 -5.633828
C 13.984596 -19.162317 -6.056411
C 13.161224 -19.90874 -5.195838
C 12.913766 -19.445415 -3.91437
H 14.909775 -14.767122 -2.32736
H 13.607553 -16.158153 -0.720738
O 12.605319 -18.478429 -1.239092
H 12.257867 -20.000974 -3.247124

H 12.716884 -20.841684 -5.534952
H 14.206494 -19.535366 -7.054783
H 15.1755 -17.38727 -6.29594
K 11.40004 -17.090611 -5.547483
K 11.047459 -20.321213 -0.456722

TS_A-B_ortho_2Kcation_acetonitrile_conf9
C 12.191082 -15.364629 -1.879728
C 13.473169 -16.987403 -1.968795
C 14.202782 -16.852836 -4.267865
C 12.535082 -14.056431 -5.385665
C 11.421634 -14.623411 -5.949178
C 10.459358 -15.29753 -5.192855
C 10.617344 -15.498692 -3.8533
C 11.788265 -14.998566 -3.219239
C 12.719592 -14.195469 -3.984399
C 9.879933 -15.430534 -7.326104
H 13.249389 -13.516626 -5.998767
H 9.868286 -16.063154 -3.314185
H 9.099173 -14.920481 -7.898093
O 11.034188 -14.591178 -7.250516
O 9.423413 -15.675054 -6.004948
H 10.165023 -16.38352 -7.801839
N 13.716827 -13.545797 -3.28373
S 15.104555 -13.134651 -3.999957
O 15.68551 -14.141261 -4.89112
O 15.947855 -12.636647 -2.883588
C 9.33211 -16.592086 0.363771
C 9.739401 -16.082574 1.59796
C 10.112757 -16.416606 -0.777944
H 9.109382 -16.194307 2.478518
H 9.783633 -16.880401 -1.700332
C 10.961154 -15.408409 1.679115
C 11.313843 -15.678792 -0.738147
H 11.301169 -15.00223 2.630755
C 11.726833 -15.222066 0.531827
H 12.675252 -14.685075 0.60422
H 8.388759 -17.130017 0.282228

C 15.801037 -11.256192 -5.88134
C 15.626255 -10.086641 -6.607656
C 14.803147 -11.675148 -4.999702
H 16.405685 -9.763723 -7.297371
C 14.463047 -9.314727 -6.478809
C 13.635884 -10.927589 -4.86085
H 12.83313 -11.29321 -4.218728
C 13.471653 -9.757784 -5.599391
H 12.548927 -9.185381 -5.504876
H 16.701173 -11.856319 -5.998148
C 14.287377 -8.061803 -7.279832
H 14.28836 -8.272315 -8.356444
H 13.346239 -7.555313 -7.043279
H 15.103779 -7.351627 -7.100398
H 13.025673 -14.741331 -1.557569
H 14.877066 -16.467196 -5.02923
C 14.442875 -16.603125 -2.95326
C 12.504397 -18.031363 -2.287569
C 12.218201 -18.225803 -3.703225
C 13.050267 -17.607441 -4.676239
C 12.717322 -17.762422 -6.033053
C 11.604196 -18.498749 -6.411298
C 10.793486 -19.113898 -5.447668
C 11.110452 -18.984405 -4.105849
H 15.303592 -16.005911 -2.658928
H 13.772396 -16.953346 -0.916658
O 11.889001 -18.654095 -1.385523
H 10.500064 -19.451974 -3.334775
H 9.9232 -19.68983 -5.754438
H 11.368432 -18.614618 -7.468551
H 13.348482 -17.292105 -6.787023
K 14.152551 -11.665454 -1.287763
K 11.708664 -18.603241 1.127682

TS_A-B_ortho_2Kcation_acetonitrile_conf10
C -1.541111 -0.222346 0.91149
C -1.658524 -0.235458 -1.189158
C -3.739943 0.872645 -1.771054

C 1.951906 1.168736 1.124901
C 1.666384 2.410459 1.643731
C 0.370451 2.798665 1.985827
C -0.697973 1.968503 1.794183
C -0.464604 0.68949 1.220194
C 0.879454 0.276974 0.913015
C 1.755236 4.470056 2.491178
H 2.977318 0.881278 0.915204
H -1.688407 2.267995 2.115733
H 2.105999 4.693216 3.506022
O 2.538449 3.398577 1.951987
O 0.397407 4.050295 2.532193
H 1.853728 5.348204 1.839798
N 1.067103 -1.027996 0.455648
S 2.033411 -1.336375 -0.763381
O 1.835133 -0.459236 -1.945521
O 1.821726 -2.786013 -1.070465
C -5.029723 1.038891 1.76048
C -5.620672 -0.139418 2.205528
C -3.702282 1.049673 1.343298
H -6.659233 -0.142753 2.529193
H -3.280316 1.968761 0.947146
C -4.878029 -1.320052 2.222188
C -2.927435 -0.123542 1.373378
H -5.33426 -2.2504 2.553743
C -3.557164 -1.306866 1.801801
H -2.984333 -2.237656 1.791771
H -5.608633 1.959508 1.725476
C 4.636129 -0.508671 -1.14732
C 5.971503 -0.396601 -0.771239
C 3.747502 -1.172994 -0.307268
H 6.668384 0.127987 -1.423699
C 6.436216 -0.936277 0.431423
C 4.183718 -1.711172 0.904668
H 3.471942 -2.196039 1.572102
C 5.517658 -1.591893 1.264337
H 5.858211 -2.005639 2.212964
H 4.27335 -0.07281 -2.074743

C 7.875329 -0.822842 0.827171
H 8.423907 -0.136871 0.174546
H 7.984131 -0.467036 1.858272
H 8.378576 -1.796899 0.7767
H -1.196755 -1.253786 0.871826
H -4.315653 1.770667 -1.991831
C -2.39858 0.936809 -1.556333
C -2.263626 -1.545917 -1.340553
C -3.707146 -1.590349 -1.505527
C -4.434858 -0.38263 -1.703281
C -5.833001 -0.457799 -1.833915
C -6.489683 -1.674648 -1.769663
C -5.767838 -2.863543 -1.586624
C -4.391396 -2.816515 -1.464534
H -1.876716 1.893034 -1.583538
H -0.571664 -0.2078 -1.270838
O -1.560759 -2.603681 -1.25971
H -3.807855 -3.724241 -1.32039
H -6.29194 -3.815773 -1.54012
H -7.572937 -1.709603 -1.86777
H -6.39763 0.461864 -1.983923
K -0.008304 -3.609567 0.655766
K 0.234497 -2.185103 -3.277344

TS_A-B_ortho_2Kcation_acetonitrile_conf11
C -1.669317 -0.071463 0.809771
C -1.470314 0.088595 -1.39426
C -3.421308 1.32833 -2.160455
C -0.968572 -3.71043 1.502664
C 0.372044 -3.606333 1.778041
C 1.056362 -2.387223 1.774635
C 0.412198 -1.223147 1.485961
C -0.976337 -1.261721 1.166051
C -1.699165 -2.52198 1.195827
C 2.46887 -3.972595 2.437926
H -1.461661 -4.672901 1.598191
H 0.941606 -0.278521 1.528586
H 2.624167 -4.076398 3.520857

O 1.229863 -4.610649 2.087459
O 2.371046 -2.602254 2.08662
H 3.283436 -4.441588 1.876743
N -3.057779 -2.487837 0.993514
S -3.886113 -3.872183 0.896119
O -3.300606 -4.864338 -0.041754
O -5.281386 -3.482858 0.614416
C 0.252023 3.194467 1.200302
C -0.706026 3.974102 1.846478
C -0.026942 1.87755 0.856169
H -0.483994 5.00489 2.112849
H 0.716403 1.309279 0.29989
C -1.95528 3.429377 2.129618
C -1.273409 1.301988 1.154701
H -2.716384 4.031272 2.621732
C -2.234907 2.114773 1.774976
H -3.216546 1.69318 1.998364
H 1.221352 3.61993 0.949448
C -4.071327 -6.033657 2.579452
C -4.11908 -6.642151 3.826971
C -3.873437 -4.655266 2.499218
H -4.272366 -7.718879 3.891714
C -3.970419 -5.898678 5.004729
C -3.720191 -3.893009 3.655039
H -3.542286 -2.821502 3.57968
C -3.764555 -4.519199 4.895418
H -3.631973 -3.926848 5.800041
H -4.179912 -6.61842 1.668686
C -4.04475 -6.564156 6.343557
H -3.534005 -7.53339 6.344437
H -3.601874 -5.946409 7.131216
H -5.086753 -6.756917 6.630354
H -2.750437 -0.199325 0.7903
H -3.922855 2.269252 -2.381993
C -2.133933 1.311322 -1.708353
C -2.055768 -1.177294 -1.762792
C -3.471144 -1.146042 -2.171157
C -4.124398 0.10279 -2.395861

C -5.469909 0.090312 -2.832806
C -6.149053 -1.101644 -3.02647
C -5.505406 -2.328467 -2.788681
C -4.180982 -2.338121 -2.383797
H -1.604557 2.248897 -1.543373
H -0.386564 0.08921 -1.290668
O -1.402759 -2.246002 -1.707576
H -3.677989 -3.27955 -2.173183
H -6.044536 -3.264206 -2.917378
H -7.181121 -1.087926 -3.3726
H -5.964235 1.041232 -3.033261
K -5.4449 -0.93433 0.245311
K -1.096885 -4.753175 -1.466948

TS_A-B_ortho_2Kcation_acetonitrile_conf12
C -1.4134253783 0.2537588991 0.4506271047
C -1.5336581323 -0.3568675796 -1.4886059483
C -3.7059480601 0.5363422302 -2.1217429759
C -4.6970484526 2.0549215533 0.8371215817
C -5.4068613111 1.0751389094 1.4812738825
C -4.8193074579 -0.1104455487 1.9302395818
C -3.5074825127 -0.3916627204 1.6855407393
C -2.7341397704 0.5526857479 0.9568343957
C -3.3130834113 1.8315625114 0.6031584809
C -6.9839814237 -0.1793951819 2.4236604921
H -5.1820918571 2.9762685529 0.5319375468
H -3.0778831122 -1.3228295044 2.034663524
H -7.4778970818 -0.0355746794 3.3906761031
O -6.718665063 1.0949034641 1.8363294929
O -5.7490603476 -0.8524238315 2.6117210585
H -7.624096639 -0.7608080129 1.740102229
N -2.4574123799 2.7894973648 0.1059911603
S -3.006590988 4.0319171968 -0.7714592544
O -3.9639918175 3.6799631807 -1.824857835
O -1.7704609127 4.7442407942 -1.181735838
C 0.2987912821 -2.7457432907 2.0907734431
C 1.5865112693 -2.2427377701 2.252373624
C -0.6958375112 -1.9762831905 1.4967800956

H 2.3591674474 -2.84963374 2.7192017599
H -1.674269649 -2.4188422866 1.3374618783
C 1.8807423848 -0.9602295155 1.7952845001
C -0.4344008634 -0.6656825361 1.0642295552
H 2.8870595813 -0.5586332773 1.8972826568
C 0.8830406869 -0.1964770411 1.2054745723
H 1.1215546127 0.8050204028 0.8364884282
H 0.0647454051 -3.7548206548 2.4245284529
C -4.7819339864 6.0553071623 -0.2144036386
C -5.3992917151 6.9837534807 0.6132216878
C -3.8556547265 5.1636183715 0.3262186166
H -6.1242500835 7.6789788829 0.1905203066
C -5.1150705421 7.0403658631 1.9839862692
C -3.5626877623 5.1963806443 1.687210076
H -2.8778773745 4.4609594549 2.1111206009
C -4.1930211837 6.128234606 2.5059843892
H -3.9758847373 6.1424521176 3.5738583123
H -5.0182863617 6.0040816932 -1.2750480609
C -5.7933431746 8.0458595605 2.862482105
H -6.884737089 7.945189063 2.8202782039
H -5.4903208718 7.9423058025 3.9092269992
H -5.5622331167 9.0717008218 2.5496496841
H -0.923521308 1.1700366192 0.120535126
H -4.3230729465 1.3506497822 -2.4941707028
C -2.349892587 0.6456550291 -2.1235250158
C -2.0952131514 -1.6884139181 -1.3323628357
C -3.5510687261 -1.7467902731 -1.1849866634
C -4.3392730154 -0.6256362719 -1.5686832515
C -5.7347546928 -0.7033449412 -1.3892713666
C -6.3239035794 -1.8294194767 -0.8388708973
C -5.5421770756 -2.9277295367 -0.4460567575
C -4.1701251821 -2.8808400442 -0.6311607607
H -1.8819968545 1.5517030431 -2.5043228465
H -0.4607077201 -0.3522461617 -1.6881861247
O -1.3871777257 -2.7272036112 -1.2821787546
H -3.5312183379 -3.688562878 -0.2639321854
H -6.0084567579 -3.7884859711 0.0288871378
H -7.4042685212 -1.8615740182 -0.7052787955

H -6.3474226232 0.15114475 -1.6757597773
K 0.0457720268 3.9135975953 0.453668262
K -2.4322960335 -4.449148687 -2.8628358296

TS_A-B_ortho_2Kcation_acetonitrile_conf13
C 11.645393 -14.739864 -1.91517
C 13.06578 -16.238812 -1.5033
C 14.069328 -16.838403 -3.632402
C 12.79085 -12.318152 0.731815
C 13.307383 -11.3319 -0.076345
C 13.248788 -11.398368 -1.468997
C 12.695389 -12.470762 -2.110298
C 12.184717 -13.538078 -1.32315
C 12.206891 -13.44329 0.112795
C 14.209182 -9.468484 -0.902908
H 12.80763 -12.210785 1.811752
H 12.610816 -12.478701 -3.190524
H 13.687519 -8.50359 -0.912243
O 13.878724 -10.16542 0.304289
O 13.77976 -10.259179 -2.003838
H 15.296675 -9.32708 -0.948348
N 11.591176 -14.463686 0.838311
S 12.282071 -15.092203 2.119846
O 13.70596 -15.470344 1.929463
O 11.4144 -16.25107 2.500236
C 11.674392 -14.827955 -5.71955
C 10.465275 -15.47116 -5.96451
C 12.083422 -14.565482 -4.415712
H 10.150706 -15.675128 -6.98569
H 13.056465 -14.112901 -4.247982
C 9.664126 -15.865415 -4.892787
C 11.280857 -14.931974 -3.32048
H 8.724305 -16.383352 -5.071947
C 10.076618 -15.606292 -3.59499
H 9.45965 -15.938002 -2.75596
H 12.314304 -14.535909 -6.54968
C 13.404213 -13.752121 4.253148
C 13.355764 -12.875516 5.33344

C 12.250165 -13.977695 3.509127
H 14.258506 -12.693882 5.915331
C 12.17354 -12.217934 5.683779
C 11.05886 -13.325042 3.83157
H 10.168789 -13.483476 3.223557
C 11.026917 -12.456003 4.911478
H 10.098889 -11.943044 5.161942
H 14.328386 -14.251103 3.972886
C 12.122782 -11.281175 6.850175
H 13.114238 -11.115772 7.282518
H 11.713275 -10.304731 6.565365
H 11.476277 -11.671619 7.646222
H 10.955905 -15.244316 -1.241482
H 14.814609 -16.69086 -4.413039
C 14.094253 -16.096923 -2.492436
C 12.168641 -17.378088 -1.557025
C 12.115794 -18.118308 -2.806886
C 13.053169 -17.832233 -3.83965
C 12.958521 -18.548615 -5.046104
C 11.977822 -19.508256 -5.229222
C 11.064131 -19.796221 -4.204361
C 11.142104 -19.111113 -3.005937
H 14.852022 -15.327461 -2.347009
H 13.242219 -15.82889 -0.508808
O 11.415264 -17.664363 -0.572095
H 10.4447 -19.314889 -2.195153
H 10.298785 -20.554402 -4.355983
H 11.919476 -20.047367 -6.172747
H 13.670869 -18.334766 -5.842295
K 9.492237 -16.312748 0.674784
K 13.217623 -18.07898 1.429688

TS_A-B_ortho_2Kcation_acetonitrile_conf14

C 11.817046 -14.782667 -2.051421
C 13.451331 -16.129912 -1.381413
C 14.692301 -16.715423 -3.392162
C 8.762224 -16.161901 -0.324979
C 8.778778 -15.353817 0.78456

C 9.733822 -14.352196 0.981245
C 10.716327 -14.13153 0.065152
C 10.773806 -14.951926 -1.099151
C 9.76645 -15.975013 -1.32332
C 8.264044 -14.245389 2.648161
H 7.94937 -16.868489 -0.461828
H 11.425505 -13.32424 0.205674
H 7.46764 -13.49322 2.561361
O 7.918834 -15.377586 1.83332
O 9.4901 -13.717649 2.168609
H 8.382981 -14.574701 3.68539
N 9.798028 -16.656826 -2.515885
S 8.7836 -17.891312 -2.756923
O 8.759512 -18.882117 -1.650299
O 9.126646 -18.435524 -4.084988
C 14.105467 -11.780523 -1.542677
C 14.141895 -11.269445 -2.839104
C 13.357301 -12.915031 -1.253489
H 14.72956 -10.381703 -3.061248
H 13.393209 -13.327702 -0.246831
C 13.437099 -11.91592 -3.850413
C 12.619056 -13.567446 -2.255134
H 13.468135 -11.535325 -4.869184
C 12.697744 -13.056668 -3.560019
H 12.147992 -13.560466 -4.35704
H 14.676084 -11.298962 -0.751442
C 6.051691 -18.104027 -2.595434
C 4.753488 -17.632406 -2.737626
C 7.121772 -17.25135 -2.867634
H 3.916431 -18.296669 -2.525157
C 4.498596 -16.316857 -3.146555
C 6.896059 -15.938297 -3.273404
H 7.738658 -15.274836 -3.461135
C 5.589664 -15.480529 -3.404577
H 5.410718 -14.45024 -3.709965
H 6.242629 -19.124485 -2.270601
C 3.093466 -15.828037 -3.31254
H 2.454655 -16.13906 -2.478248

H 3.047186 -14.736984 -3.385787
H 2.63893 -16.235549 -4.224791
H 11.637036 -15.322559 -2.979725
H 15.48168 -16.513813 -4.11476
C 14.494555 -15.890416 -2.323417
C 12.724272 -17.375747 -1.39507
C 12.889632 -18.213617 -2.59578
C 13.891466 -17.89097 -3.559787
C 14.055336 -18.74713 -4.673842
C 13.257674 -19.867758 -4.839263
C 12.26172 -20.171992 -3.894948
C 12.099739 -19.358672 -2.785407
H 15.116438 -15.004806 -2.199328
H 13.479076 -15.615849 -0.422037
O 11.971875 -17.708752 -0.449477
H 11.315913 -19.568819 -2.060216
H 11.618842 -21.037854 -4.035992
H 13.40873 -20.519375 -5.698188
H 14.838501 -18.520469 -5.397746
K 11.202939 -17.240581 -5.046033
K 10.017391 -18.885449 0.652457

TS_A-B_ortho_2Kcation_acetonitrile_conf15

C 12.299262 -14.821166 -1.890159
C 13.168 -16.638121 -1.414623
C 13.989741 -17.321023 -3.581337
C 13.073836 -14.667095 -5.564244
C 11.879257 -15.114192 -6.066136
C 10.756658 -15.318104 -5.259655
C 10.812544 -15.160106 -3.906251
C 12.044943 -14.770324 -3.312765
C 13.167958 -14.43976 -4.165396
C 10.241619 -15.921777 -7.32716
H 13.918508 -14.49401 -6.223041
H 9.930212 -15.367389 -3.315653
H 9.627012 -15.436723 -8.091371
O 11.559509 -15.369804 -7.361165
O 9.691432 -15.67546 -6.04253

H 10.307921 -17.008839 -7.499333
N 14.264283 -13.852527 -3.564778
S 15.732768 -13.963051 -4.22701
O 16.093131 -15.275647 -4.767236
O 16.631591 -13.370007 -3.204412
C 9.142816 -14.726709 0.286818
C 9.604003 -13.981827 1.373609
C 9.99088 -15.053766 -0.770304
H 8.928507 -13.70128 2.179706
H 9.603706 -15.683792 -1.562398
C 10.944252 -13.585824 1.399544
C 11.327203 -14.604575 -0.803893
H 11.328309 -13.005069 2.237049
C 11.780595 -13.901054 0.332465
H 12.823474 -13.577313 0.35758
H 8.104928 -15.05485 0.253622
C 16.910892 -12.850431 -6.450037
C 17.030071 -11.913163 -7.466685
C 15.808668 -12.802576 -5.594746
H 17.890176 -11.955168 -8.134477
C 16.062378 -10.916926 -7.658373
C 14.831661 -11.825034 -5.770827
H 13.941333 -11.830917 -5.140294
C 14.961853 -10.892787 -6.797837
H 14.185871 -10.141879 -6.946328
H 17.660389 -13.628135 -6.317553
C 16.200291 -9.924679 -8.771344
H 16.15233 -10.415578 -9.751391
H 15.409766 -9.16803 -8.743333
H 17.164675 -9.404167 -8.730334
H 13.243279 -14.325863 -1.661397
H 14.76274 -17.323166 -4.346656
C 14.235238 -16.767605 -2.364097
C 11.992441 -17.494029 -1.538228
C 11.717545 -18.011717 -2.872539
C 12.706271 -17.890027 -3.886908
C 12.394182 -18.345749 -5.179542
C 11.151605 -18.896243 -5.45745

C 10.18422 -19.021001 -4.451093
C 10.475849 -18.591368 -3.167304
H 15.201703 -16.31332 -2.155138
H 13.429346 -16.373661 -0.385374
O 11.21689 -17.689044 -0.568437
H 9.74516 -18.677084 -2.36453
H 9.212937 -19.454393 -4.679076
H 10.932398 -19.251606 -6.463662
H 13.145212 -18.257423 -5.964479
K 15.056123 -11.602458 -2.140965
K 10.951727 -16.89308 1.808689

TS_A-B_ortho_2Kcation_acetonitrile_conf16
C -10.40144 16.956016 -1.898836
C -11.305955 18.106791 -0.010185
C -9.415846 19.29319 0.929149
C -12.910106 17.873645 -4.552923
C -13.733791 16.77682 -4.456055
C -13.473971 15.712638 -3.586723
C -12.384044 15.713263 -2.767484
C -11.514722 16.846078 -2.769244
C -11.770961 17.944652 -3.690896
C -15.332312 15.240599 -4.72688
H -13.093641 18.634829 -5.307353
H -12.170646 14.852202 -2.145677
H -15.388263 14.555879 -5.581295
O -14.848791 16.51452 -5.182959
O -14.420226 14.739506 -3.764057
H -16.322013 15.378191 -4.271483
N -10.819313 18.925366 -3.756151
S -11.027632 20.160676 -4.762056
O -10.758286 19.734224 -6.16516
O -12.307915 20.894483 -4.602873
C -9.667298 13.984236 0.358758
C -8.274872 14.048978 0.406059
C -10.392493 14.904482 -0.386518
H -7.712928 13.32676 0.993947
H -11.478469 14.875576 -0.33862

C -7.613297 15.06431 -0.28004
C -9.742569 15.918389 -1.116596
H -6.529346 15.143925 -0.227985
C -8.339536 15.990185 -1.018112
H -7.820596 16.802621 -1.528946
H -10.195053 13.219627 0.925021
C -9.752994 22.544418 -4.7976
C -8.734888 23.433846 -4.477457
C -9.719952 21.251891 -4.275004
H -8.765345 24.447354 -4.875595
C -7.67633 23.055442 -3.644305
C -8.675528 20.847385 -3.444874
H -8.689912 19.855649 -2.999635
C -7.666995 21.751012 -3.137092
H -6.868388 21.447374 -2.460514
H -10.577997 22.852619 -5.436805
C -6.582474 24.016909 -3.297978
H -5.687041 23.838764 -3.907802
H -6.274922 23.917108 -2.250567
H -6.884306 25.055727 -3.465633
H -9.758096 17.808662 -2.111671
H -8.546291 19.328319 1.584155
C -10.189454 18.16713 0.856664
C -11.762139 19.267731 -0.728559
C -10.907253 20.461562 -0.655906
C -9.75734 20.46516 0.184234
C -8.998365 21.649327 0.291638
C -9.361196 22.794701 -0.391695
C -10.493086 22.789717 -1.219152
C -11.247659 21.636927 -1.339889
H -9.93035 17.287991 1.445826
H -12.011647 17.281549 0.064954
O -12.846316 19.253082 -1.371167
H -12.108897 21.617335 -2.004479
H -10.76707 23.684938 -1.774402
H -8.767915 23.702168 -0.288629
H -8.123428 21.651921 0.941756
K -14.382974 20.325748 -3.074073

K -9.605759 17.362591 -5.848374

TS_A-B_ortho_2Kcation_acetonitrile_conf17
C -10.76224 16.861891 -1.31953
C -10.757077 17.905582 0.762729
C -9.106432 19.633092 0.40425
C -10.771298 13.33336 0.000999
C -9.479958 12.963276 -0.306821
C -8.59492 13.829619 -0.953489
C -8.958956 15.104808 -1.299307
C -10.272209 15.552397 -0.974391
C -11.189024 14.650924 -0.322882
C -7.504919 11.96229 -0.461917
H -11.454139 12.655187 0.502006
H -8.2669 15.739541 -1.840526
H -7.155189 11.127642 -1.074716
O -8.881787 11.755175 -0.121734
O -7.421893 13.17171 -1.208659
H -6.916867 12.052215 0.463283
N -12.457771 15.098368 0.027004
S -13.737323 14.325148 -0.530015
O -13.460707 12.962439 -1.05317
O -14.819073 14.483665 0.458609
C -8.295257 19.170544 -3.048063
C -9.168522 19.901814 -3.846281
C -8.775835 18.164764 -2.215062
H -8.788241 20.692591 -4.489683
H -8.082424 17.657596 -1.550637
C -10.538523 19.6259 -3.809641
C -10.151972 17.853521 -2.176199
H -11.229423 20.206177 -4.41783
C -11.018941 18.62794 -2.981216
H -12.090317 18.425665 -2.930196
H -7.230937 19.39733 -3.054501
C -14.73144 14.544648 -3.108534
C -15.182809 15.25169 -4.222433
C -14.273697 15.235878 -1.986306
H -15.551529 14.706675 -5.091298

C -15.202019 16.649066 -4.234649
C -14.297793 16.632861 -1.973231
H -13.944282 17.185604 -1.099548
C -14.757271 17.322484 -3.087404
H -14.774656 18.413094 -3.066811
H -14.783189 13.456364 -3.083341
C -15.696093 17.41082 -5.424697
H -14.926821 18.086199 -5.819014
H -16.558281 18.037404 -5.166046
H -16.003276 16.744841 -6.23681
H -11.84866 16.912801 -1.287835
H -8.080275 19.996129 0.450156
C -9.4335 18.394911 0.883567
C -11.848087 18.788437 0.401747
C -11.468945 20.078559 -0.169787
C -10.106077 20.482505 -0.176951
C -9.773609 21.712452 -0.771622
C -10.75004 22.510108 -1.347124
C -12.091948 22.108361 -1.333541
C -12.443896 20.906197 -0.741259
H -8.664106 17.745342 1.300266
H -11.031992 16.965919 1.241085
O -13.053723 18.443643 0.517847
H -13.478579 20.567714 -0.715361
H -12.853261 22.741554 -1.784637
H -10.472223 23.456485 -1.807387
H -8.730817 22.029251 -0.78056
K -13.996684 16.501979 1.989029
K -11.639123 13.123199 -3.008845

TS_A-B_ortho_2Kcation_acetonitrile_conf18
C 1.227488 0.592486 0.99956
C 1.506206 -0.15595 -0.947638
C 3.489896 0.93478 -1.827611
C -2.414146 1.46574 0.606177
C -2.320149 2.837744 0.643474
C -1.109014 3.499921 0.849119
C 0.063806 2.814314 0.997516

C 0.029746 1.395474 0.924697
C -1.22834 0.716615 0.757247
C -2.720678 5.030168 0.683442
H -3.379431 0.982079 0.494642
H 0.984713 3.344468 1.209748
H -3.165981 5.546968 1.542219
O -3.326126 3.738572 0.551066
O -1.32754 4.847269 0.900561
H -2.881595 5.597334 -0.24252
N -1.221963 -0.677745 0.804282
S -2.049909 -1.538914 -0.238991
O -1.865036 -1.134551 -1.656217
O -1.648798 -2.95687 0.023076
C 4.469995 2.538275 1.426475
C 5.160058 1.68942 2.286055
C 3.185175 2.218255 0.998644
H 6.164905 1.943392 2.616409
H 2.688804 2.867872 0.283531
C 4.561361 0.503864 2.71244
C 2.553104 1.039291 1.432778
H 5.097299 -0.174555 3.372854
C 3.283243 0.185683 2.280486
H 2.826009 -0.755828 2.595202
H 4.938164 3.45497 1.073805
C -4.686318 -1.182092 -0.967615
C -6.050052 -1.111892 -0.698935
C -3.802223 -1.456752 0.071681
H -6.743809 -0.891338 -1.50927
C -6.548254 -1.311372 0.591757
C -4.272994 -1.648282 1.371676
H -3.570949 -1.830081 2.184722
C -5.63504 -1.57707 1.622506
H -6.003485 -1.721447 2.637596
H -4.299282 -1.011042 -1.968857
C -8.017291 -1.251613 0.872499
H -8.433461 -2.257077 1.017033
H -8.570039 -0.786371 0.050511
H -8.232608 -0.688026 1.787331

H 1.010762 -0.42212 1.32725
H 3.977158 1.76188 -2.342494
C 2.136979 0.893428 -1.694147
C 2.263679 -1.341211 -0.591107
C 3.711366 -1.252459 -0.685816
C 4.313165 -0.110068 -1.285769
C 5.716925 -0.042999 -1.337499
C 6.497977 -1.06053 -0.817247
C 5.900922 -2.190502 -0.238635
C 4.522527 -2.283482 -0.183508
H 1.515582 1.698386 -2.085671
H 0.432623 -0.303707 -1.066391
O 1.679447 -2.381699 -0.148693
H 4.034405 -3.149126 0.261086
H 6.522504 -2.987067 0.164727
H 7.582717 -0.986042 -0.863039
H 6.185639 0.828706 -1.792996
K 0.141645 -2.823773 1.978704
K 0.008442 -3.003864 -2.210166

TS_A-B_ortho_2Kcation_acetonitrile_conf19
C 0.871022 -0.129007 0.68279
C 1.577458 -0.275337 -1.256248
C 3.294435 1.424057 -1.23908
C 3.068614 2.630107 1.976399
C 4.026172 1.879733 2.607734
C 3.907206 0.497213 2.772315
C 2.862459 -0.197525 2.237781
C 1.869995 0.512844 1.50766
C 1.933561 1.958865 1.448975
C 5.862937 1.126082 3.59951
H 3.17935 3.706081 1.890294
H 2.834044 -1.273136 2.350099
H 6.210091 1.237052 4.631101
O 5.166962 2.310564 3.205831
O 4.961642 0.033525 3.512575
H 6.707632 0.965026 2.909395
N 0.835436 2.617865 0.932781

S 0.975116 4.086437 0.275397
O 2.128097 4.280705 -0.60675
O -0.373188 4.353358 -0.287134
C 0.100352 -3.76309 1.639635
C -1.26332 -3.820397 1.346785
C 0.83675 -2.602622 1.40518
H -1.839115 -4.718437 1.562995
H 1.903455 -2.62559 1.594091
C -1.880944 -2.696984 0.78981
C 0.22359 -1.432853 0.911191
H -2.943575 -2.716807 0.551816
C -1.143334 -1.536369 0.574927
H -1.637861 -0.660989 0.147746
H 0.602257 -4.631395 2.06401
C 1.516579 6.586171 1.278949
C 1.576222 7.549411 2.276325
C 1.139079 5.282068 1.602909
H 1.874586 8.565935 2.021118
C 1.268723 7.240109 3.608582
C 0.835325 4.950432 2.92134
H 0.602317 3.91617 3.179114
C 0.904176 5.925959 3.913295
H 0.686603 5.659658 4.947563
H 1.7717 6.831543 0.250071
C 1.348427 8.290647 4.672726
H 0.685794 9.136655 4.452608
H 2.362147 8.701702 4.754263
H 1.070897 7.896968 5.655686
H 0.138327 0.604729 0.345788
H 3.608044 2.458349 -1.361139
C 2.025739 1.053393 -1.556966
C 2.560003 -1.337869 -1.066404
C 3.893019 -0.910642 -0.661177
C 4.233637 0.468432 -0.720234
C 5.504842 0.860772 -0.266772
C 6.403217 -0.074859 0.22506
C 6.06556 -1.434369 0.270209
C 4.822161 -1.844033 -0.18127

H 1.316085 1.793821 -1.920952
H 0.631273 -0.606483 -1.695337
O 2.241516 -2.550661 -1.156733
H 4.531332 -2.893099 -0.157977
H 6.777269 -2.160461 0.656854
H 7.387355 0.248446 0.562317
H 5.775114 1.915914 -0.306092
K -1.885511 2.976879 1.304173
K 0.150269 -3.896008 -1.567923

TS_A-B_ortho_2Kcation_acetonitrile_conf20
C 1.226304 0.814422 0.618718
C 1.386698 -0.551762 -1.129912
C 2.855818 0.557459 -2.723088
C 1.798092 -1.447498 3.580073
C 0.486749 -1.729779 3.871537
C -0.584575 -1.189149 3.154132
C -0.372213 -0.344207 2.107988
C 0.966792 -0.027572 1.735179
C 2.083641 -0.565091 2.493871
C -1.411144 -2.377336 4.843366
H 2.58506 -1.833488 4.220633
H -1.209821 0.103963 1.586377
H -1.708609 -1.819067 5.741977
O 0.016847 -2.538867 4.853714
O -1.761662 -1.650725 3.677206
H -1.882118 -3.365226 4.819872
N 3.34764 -0.138459 2.165265
S 4.629469 -0.809384 2.885501
O 4.615507 -2.294838 2.886042
O 5.81101 -0.188317 2.256285
C -1.79866 2.483654 -0.984666
C -1.331516 3.791936 -1.101087
C -0.995767 1.495908 -0.427621
H -1.963123 4.561684 -1.53853
H -1.362063 0.471137 -0.400724
C -0.04394 4.10051 -0.671751
C 0.297654 1.792492 0.033739

H 0.337483 5.114817 -0.768776
C 0.760808 3.107945 -0.124646
H 1.769709 3.355353 0.210186
H -2.79318 2.22684 -1.343178
C 5.329029 -1.115167 5.521974
C 5.394364 -0.713535 6.849525
C 4.638118 -0.325262 4.602717
H 5.933392 -1.329082 7.568996
C 4.777982 0.467619 7.282959
C 4.013982 0.852019 5.007872
H 3.458185 1.448692 4.286405
C 4.08412 1.236068 6.342442
H 3.586655 2.150276 6.664147
H 5.804251 -2.036922 5.193568
C 4.87284 0.897165 8.71381
H 5.870818 1.293417 8.941589
H 4.703876 0.060115 9.400584
H 4.148921 1.682496 8.952728
H 2.263538 1.140722 0.564201
H 3.011254 1.242583 -3.555206
C 1.603959 0.328477 -2.230139
C 2.449721 -1.400474 -0.649853
C 3.805901 -1.081818 -1.129928
C 3.987194 -0.135177 -2.182797
C 5.299031 0.106365 -2.653872
C 6.389689 -0.544657 -2.100652
C 6.207322 -1.461511 -1.051004
C 4.92864 -1.730532 -0.591594
H 0.748983 0.852816 -2.655168
H 0.377761 -0.91005 -0.932574
O 2.231243 -2.338088 0.152964
H 4.779449 -2.417481 0.239327
H 7.065 -1.952907 -0.597423
H 7.389502 -0.351185 -2.485436
H 5.438733 0.806441 -3.477992
K 5.090354 1.366886 0.325954
K 2.802169 -3.977369 2.000588

TS_A-B_para_2Kcation_acetonitrile

C 12.267273 -16.852093 -1.108616
C 14.493382 -17.026145 -0.781032
C 10.626639 -19.721715 0.684723
C 10.473166 -19.144467 1.919923
C 10.972247 -17.877282 2.225147
C 11.604981 -17.103403 1.291736
C 11.717418 -17.620224 -0.028882
C 11.282742 -18.964042 -0.318432
C 9.706639 -18.529482 3.881005
H 10.227094 -20.701407 0.442991
H 12.032522 -16.141239 1.559231
H 8.743357 -18.071324 3.610265
O 9.9225 -19.69113 3.071853
O 10.775329 -17.640394 3.587198
H 9.732404 -18.80726 4.938278
N 11.483038 -19.495044 -1.581201
S 10.161582 -19.687336 -2.493303
O 9.020888 -20.270894 -1.778104
O 10.644813 -20.365345 -3.719168
C 11.3506 -13.182236 -0.767
C 11.962563 -12.662186 -1.903937
C 11.433493 -14.540501 -0.478029
H 11.897702 -11.598645 -2.123998
H 10.891927 -14.928446 0.379741
C 12.636006 -13.520421 -2.775911
C 12.139409 -15.417234 -1.32213
H 13.093563 -13.13016 -3.682789
C 12.71253 -14.873626 -2.490654
H 13.226522 -15.543992 -3.18108
H 10.792103 -12.52704 -0.101357
C 8.909331 -17.285195 -2.068319
C 8.661348 -15.945063 -2.340696
C 9.693703 -18.030701 -2.949763
H 8.047389 -15.361609 -1.65417
C 9.185901 -15.326792 -3.481666
C 10.230427 -17.436245 -4.090599
H 10.830801 -18.034155 -4.773578

C 9.968548 -16.096225 -4.350754
H 10.37901 -15.631583 -5.247617
H 8.48767 -17.765708 -1.186715
C 8.897293 -13.887238 -3.774829
H 9.656065 -13.446813 -4.430886
H 7.92813 -13.77043 -4.277724
H 8.855464 -13.288552 -2.857616
H 12.349344 -17.429443 -2.028468
H 14.70201 -16.354615 -1.610929
C 14.631467 -18.421617 -0.976184
C 14.578646 -19.301073 0.072581
C 14.480919 -18.843014 1.432714
C 14.595939 -17.393244 1.651887
C 14.610297 -16.50083 0.549865
C 14.647097 -15.113957 0.796267
C 14.688065 -14.627617 2.090949
C 14.703782 -15.51315 3.179803
C 14.656191 -16.876896 2.955072
H 14.699321 -18.787136 -2.002861
H 14.598047 -20.381027 -0.074176
O 14.303657 -19.638571 2.389706
H 14.690351 -17.581029 3.786155
H 14.761395 -15.124262 4.194343
H 14.722847 -13.554152 2.265218
H 14.64379 -14.426385 -0.04977
K 12.94458 -21.31462 -2.97105
K 12.616473 -19.916363 4.227987

TS_A-B_para_2Kcation_acetonitrile_conf2
C 12.390381 -16.807412 -0.914913
C 14.335296 -17.166525 -0.419782
C 11.114368 -13.951805 1.17013
C 11.389512 -12.914576 0.313817
C 12.00295 -13.103916 -0.931425
C 12.36338 -14.346328 -1.369239
C 12.095557 -15.456219 -0.521806
C 11.478528 -15.260612 0.768158
C 11.548789 -10.934728 -0.699807

H 10.612093 -13.794536 2.117809
H 12.803861 -14.478855 -2.352108
H 10.663391 -10.508473 -1.190567
O 11.145882 -11.594051 0.50177
O 12.155266 -11.898227 -1.553429
H 12.275308 -10.151051 -0.455062
N 11.242173 -16.358199 1.584127
S 9.691193 -16.659649 1.936114
O 8.89541 -15.480655 2.295574
O 9.718849 -17.791734 2.893053
C 12.958174 -17.303335 -4.660013
C 12.010424 -18.290685 -4.92278
C 13.11316 -16.804346 -3.370927
H 11.891667 -18.678484 -5.932326
H 13.878323 -16.05042 -3.187355
C 11.227295 -18.784962 -3.882735
C 12.32114 -17.280653 -2.316005
H 10.489903 -19.563659 -4.071857
C 11.390613 -18.293328 -2.592219
H 10.773143 -18.676983 -1.779272
H 13.584865 -16.921385 -5.463304
C 8.71925 -16.414527 -0.625776
C 8.204526 -16.904719 -1.818303
C 8.986528 -17.297653 0.423514
H 7.998465 -16.215429 -2.636889
C 7.930333 -18.268577 -1.98556
C 8.739118 -18.659865 0.274553
H 8.936819 -19.329902 1.108165
C 8.205097 -19.133462 -0.921793
H 7.98841 -20.196547 -1.030189
H 8.893801 -15.347317 -0.496082
C 7.341643 -18.774842 -3.266536
H 7.422375 -19.864236 -3.349782
H 6.275827 -18.523122 -3.342456
H 7.832299 -18.329553 -4.140516
H 11.940934 -17.512529 -0.213521
H 14.693255 -16.639459 -1.30447
C 14.345248 -18.58766 -0.46016

C 14.361317 -19.351068 0.685637
C 14.485269 -18.762752 1.979524
C 14.706517 -17.31811 2.031031
C 14.63817 -16.538686 0.849562
C 14.830751 -15.149562 0.949357
C 15.075769 -14.54803 2.170759
C 15.163302 -15.322375 3.33704
C 14.987312 -16.691852 3.260524
H 14.223064 -19.073201 -1.428642
H 14.194914 -20.428964 0.622777
O 14.391759 -19.444759 3.058759
H 15.087572 -17.318973 4.147522
H 15.387003 -14.847978 4.290389
H 15.213954 -13.470145 2.224822
H 14.776137 -14.544071 0.045631
K 12.138309 -17.841045 3.771439
K 15.931438 -21.485252 3.011022

TS_A-B_para_2Kcation_acetonitrile_conf3
C 12.13492 -17.217268 -0.982081
C 14.301718 -17.037599 -0.61571
C 11.946862 -16.850578 -4.698766
C 11.459937 -18.095391 -5.024321
C 11.256282 -19.100201 -4.069668
C 11.505653 -18.894771 -2.74224
C 11.982623 -17.61299 -2.347814
C 12.243596 -16.598659 -3.342114
C 10.665292 -19.900707 -6.065514
H 12.094789 -16.076299 -5.444108
H 11.277058 -19.667043 -2.015483
H 9.617133 -19.972542 -6.381575
O 11.117826 -18.556436 -6.249176
O 10.774262 -20.219336 -4.682747
H 11.299515 -20.576641 -6.652148
N 12.839139 -15.4116 -2.931202
S 12.026582 -14.03457 -3.158184
O 11.376041 -13.904307 -4.464597
O 12.970573 -12.972284 -2.728894

C 11.194197 -19.923885 1.518303
C 10.407689 -19.180975 2.401528
C 11.803891 -19.30617 0.428661
H 9.869349 -19.680042 3.2079
H 12.417001 -19.898434 -0.246176
C 10.291249 -17.798223 2.22225
C 11.633781 -17.930015 0.189851
H 9.67685 -17.205159 2.900122
C 10.917625 -17.180077 1.144291
H 10.806312 -16.105444 0.990655
H 11.307406 -20.99757 1.657845
C 9.632796 -14.92213 -2.14746
C 8.685347 -15.076401 -1.143666
C 10.730491 -14.085722 -1.932454
H 7.829902 -15.730813 -1.30948
C 8.804019 -14.399321 0.077281
C 10.868885 -13.400533 -0.728327
H 11.719927 -12.737142 -0.588986
C 9.905277 -13.556698 0.264137
H 10.005573 -13.00837 1.201588
H 9.521955 -15.437222 -3.100416
C 7.768856 -14.568901 1.146811
H 8.133232 -14.23026 2.123286
H 6.863879 -13.989153 0.92366
H 7.454069 -15.61525 1.242633
H 12.104486 -16.134916 -0.866285
H 14.292658 -16.547008 -1.585954
C 14.800701 -18.356637 -0.506258
C 15.113064 -18.919726 0.70612
C 14.991221 -18.179869 1.931831
C 14.637051 -16.753385 1.811623
C 14.299665 -16.197865 0.552116
C 13.894737 -14.846812 0.492026
C 13.860996 -14.063638 1.632288
C 14.249447 -14.599706 2.8707
C 14.627649 -15.928911 2.95095
H 14.87633 -18.957573 -1.414131
H 15.440594 -19.954602 0.782631

O 15.123153 -18.713375 3.064057
H 14.98217 -16.354253 3.89129
H 14.26513 -13.967471 3.75627
H 13.540017 -13.024923 1.568188
H 13.55954 -14.438225 -0.466551
K 15.307266 -14.095716 -2.867391
K 12.850934 -18.396618 4.188708

TS_A-B_para_2Kcation_acetonitrile_conf4
C 11.646045 -15.273279 -1.373212
C 13.552951 -15.216591 -0.787056
C 9.145884 -16.409913 1.201315
C 9.024573 -15.253057 1.929631
C 9.792326 -14.114422 1.664659
C 10.683043 -14.079232 0.625581
C 10.799559 -15.230769 -0.194293
C 10.078524 -16.432431 0.133037
C 8.471047 -13.672824 3.401368
H 8.558637 -17.294116 1.423224
H 11.298918 -13.199073 0.466323
H 7.555034 -13.089693 3.232766
O 8.244184 -15.028236 3.019106
O 9.524569 -13.152719 2.594554
H 8.767469 -13.63864 4.456035
N 10.322649 -17.596865 -0.582437
S 9.083701 -18.25627 -1.381775
O 7.815141 -18.282659 -0.64666
O 9.608902 -19.537355 -1.913833
C 11.26108 -12.086233 -3.437937
C 11.423145 -12.604408 -4.723148
C 11.334104 -12.917629 -2.326961
H 11.359573 -11.948372 -5.589301
H 11.179953 -12.485928 -1.342901
C 11.653285 -13.967053 -4.889098
C 11.574177 -14.293791 -2.474019
H 11.75935 -14.390816 -5.886751
C 11.733735 -14.79586 -3.777226
H 11.902515 -15.865259 -3.910561

H 11.068507 -11.024212 -3.300063
C 8.258784 -15.928252 -2.587552
C 8.122036 -15.058518 -3.660666
C 8.834833 -17.18591 -2.788801
H 7.678496 -14.07529 -3.502342
C 8.537095 -15.423686 -4.947842
C 9.264854 -17.566067 -4.057236
H 9.692846 -18.556034 -4.199085
C 9.105591 -16.688595 -5.126165
H 9.423763 -16.992475 -6.12419
H 7.90555 -15.645436 -1.596923
C 8.356786 -14.484657 -6.100412
H 7.302875 -14.417395 -6.400277
H 8.677676 -13.467181 -5.844459
H 8.923965 -14.806919 -6.980795
H 11.669643 -16.293719 -1.764876
H 13.581357 -14.147579 -0.566714
C 13.530584 -16.100874 0.335227
C 13.890287 -17.416906 0.233511
C 14.386725 -17.969152 -0.990497
C 14.621185 -17.036939 -2.091106
C 14.310252 -15.657488 -1.941786
C 14.67029 -14.772862 -2.974772
C 15.281528 -15.230923 -4.128367
C 15.517311 -16.603462 -4.30962
C 15.187952 -17.489622 -3.300949
H 13.110977 -15.732369 1.27074
H 13.798617 -18.085459 1.090407
O 14.643078 -19.215431 -1.106764
H 15.303219 -18.565543 -3.446261
H 15.938685 -16.964033 -5.245884
H 15.54833 -14.526819 -4.913695
H 14.441577 -13.713167 -2.864328
K 11.90839 -19.842938 -0.752385
K 17.228977 -19.201324 -1.449097

TS_A-B_para_2Kcation_acetonitrile_conf5

C 11.482216 -15.273532 -1.40253

C 13.62963 -15.085591 -0.797716
C 11.045981 -18.275086 -3.637212
C 10.861033 -19.234303 -2.674424
C 10.933857 -18.954849 -1.309481
C 11.175395 -17.691757 -0.843446
C 11.321836 -16.64563 -1.796911
C 11.294794 -16.946214 -3.206898
C 10.164527 -20.985091 -1.550432
H 10.954322 -18.486242 -4.697549
H 11.266711 -17.495657 0.220064
H 9.07853 -20.810859 -1.517918
O 10.668131 -20.600433 -2.8341
O 10.824314 -20.154438 -0.603777
H 10.406785 -22.033765 -1.358593
N 11.458833 -15.933061 -4.131514
S 10.130001 -15.516179 -4.954427
O 9.339352 -16.658093 -5.429107
O 10.58994 -14.518955 -5.947036
C 9.182293 -14.502496 1.518869
C 9.452788 -13.149922 1.70833
C 9.837901 -15.221053 0.525347
H 8.937209 -12.591452 2.486903
H 9.558568 -16.257143 0.353267
C 10.36662 -12.507921 0.871412
C 10.791051 -14.603203 -0.303732
H 10.56417 -11.4445 0.990469
C 11.016237 -13.224233 -0.121119
H 11.714186 -12.714538 -0.787596
H 8.441208 -15.001847 2.140231
C 8.241777 -15.380607 -2.964488
C 7.52177 -14.724878 -1.972144
C 9.117282 -14.652121 -3.769903
H 6.830275 -15.2902 -1.34667
C 7.660922 -13.34826 -1.762681
C 9.284726 -13.282293 -3.570273
H 9.965067 -12.727 -4.213141
C 8.555407 -12.64182 -2.576387
H 8.67481 -11.568331 -2.427635

H 8.113364 -16.447834 -3.139176
C 6.849651 -12.641954 -0.721463
H 5.906094 -12.26891 -1.14184
H 6.589858 -13.305387 0.111038
H 7.381311 -11.776084 -0.311052
H 11.689695 -14.622313 -2.25216
H 13.490531 -14.076985 -0.413692
C 13.706934 -16.153618 0.127616
C 14.082447 -17.410094 -0.268874
C 14.47303 -17.68876 -1.626305
C 14.723243 -16.518303 -2.481438
C 14.33301 -15.227233 -2.047723
C 14.683306 -14.113298 -2.835704
C 15.450446 -14.267913 -3.979051
C 15.825473 -15.55336 -4.41244
C 15.439323 -16.664974 -3.678904
H 13.361834 -15.983653 1.148001
H 14.054858 -18.248857 0.426704
O 14.636301 -18.857526 -2.0569
H 15.736835 -17.669096 -3.97529
H 16.459941 -15.670388 -5.291082
H 15.791752 -13.387258 -4.524292
H 14.40025 -13.116153 -2.496568
K 13.156022 -14.917355 -5.993531
K 13.4122 -21.055612 -1.871764

TS_A-B_para_2Kcation_acetonitrile_conf6
C 11.303028 -15.672279 -1.887563
C 13.119871 -15.222321 -0.747985
C 10.441808 -12.250442 -3.136944
C 10.646993 -12.442629 -4.484418
C 11.135102 -13.644573 -5.007482
C 11.41187 -14.719074 -4.209453
C 11.185264 -14.583236 -2.810549
C 10.737552 -13.32297 -2.269266
C 10.780909 -12.237655 -6.700862
H 10.055278 -11.31737 -2.740893
H 11.726243 -15.659101 -4.648911

H 9.88684 -12.312188 -7.333217
O 10.440148 -11.558849 -5.48871
O 11.244356 -13.540156 -6.364785
H 11.576785 -11.682106 -7.211879
N 10.678856 -13.181257 -0.886668
S 9.246067 -12.850965 -0.218874
O 8.422074 -11.891032 -0.959026
O 9.547892 -12.568831 1.205358
C 12.363993 -18.924815 -3.550175
C 11.456311 -19.811689 -2.967113
C 12.358944 -17.577196 -3.197617
H 11.407232 -20.847429 -3.305563
H 13.074073 -16.903695 -3.664585
C 10.586942 -19.349866 -1.973236
C 11.444532 -17.082222 -2.249084
H 9.865961 -20.026813 -1.514044
C 10.60253 -18.008039 -1.603568
H 9.908937 -17.643655 -0.84394
H 13.058815 -19.27433 -4.312111
C 7.784182 -14.814683 -1.456635
C 7.239744 -16.089178 -1.55216
C 8.38669 -14.413462 -0.2628
H 6.770739 -16.403836 -2.484178
C 7.273967 -16.975985 -0.468431
C 8.438216 -15.279041 0.827455
H 8.897646 -14.942285 1.754543
C 7.874765 -16.547544 0.720897
H 7.892625 -17.218272 1.580539
H 7.737247 -14.123345 -2.296405
C 6.674773 -18.344437 -0.583633
H 5.579731 -18.310189 -0.516208
H 6.914266 -18.810239 -1.547547
H 7.02388 -19.010154 0.214002
H 10.712565 -15.511842 -0.987333
H 12.720461 -14.211877 -0.696424
C 12.91975 -16.082512 0.359066
C 13.602801 -17.265827 0.484136
C 14.622477 -17.655361 -0.457689

C 14.948557 -16.691848 -1.516028
C 14.215781 -15.4834 -1.640095
C 14.536333 -14.601329 -2.691423
C 15.545442 -14.908581 -3.58757
C 16.272384 -16.101954 -3.455272
C 15.974315 -16.980431 -2.42888
H 12.136246 -15.826712 1.078281
H 13.417998 -17.929947 1.33028
O 15.182828 -18.781926 -0.400586
H 16.523999 -17.910549 -2.299792
H 17.071158 -16.330333 -4.157776
H 15.780114 -14.218393 -4.39573
H 13.96924 -13.675722 -2.798011
K 12.070745 -11.963376 1.179574
K 13.360924 -20.530879 -0.593706

TS_A-B_para_2Kcation_acetonitrile_conf7
C -13.084608 16.138181 0.740822
C -14.514019 17.589156 0.709637
C -11.13774 16.308047 -2.470669
C -11.857079 15.403253 -3.211321
C -12.982763 14.738033 -2.707951
C -13.431634 14.954066 -1.436309
C -12.718463 15.878936 -0.625201
C -11.567713 16.575973 -1.147455
C -12.612753 14.012232 -4.782276
H -10.251517 16.793679 -2.862845
H -14.283069 14.402977 -1.050276
H -12.105708 13.051577 -4.944489
O -11.636371 15.013578 -4.491148
O -13.491194 13.910723 -3.667444
H -13.180315 14.309738 -5.671918
N -10.896985 17.483835 -0.339897
S -9.354219 17.124849 -0.003546
O -8.559194 16.666401 -1.148126
O -8.842579 18.279035 0.77366
C -15.381417 13.521935 2.313298
C -14.623484 13.168207 3.427935

C -14.91555 14.485309 1.424702
H -14.990721 12.414093 4.120842
H -15.538458 14.761072 0.574088
C -13.400772 13.795241 3.654079
C -13.679453 15.115234 1.630728
H -12.802767 13.535634 4.526312
C -12.941108 14.766612 2.771375
H -11.980911 15.253386 2.946393
H -16.344531 13.047695 2.135951
C -9.772721 14.484405 0.645175
C -9.875207 13.416008 1.525733
C -9.436182 15.749593 1.133728
H -10.139737 12.430111 1.144023
C -9.630418 13.577337 2.895899
C -9.205495 15.938093 2.49397
H -8.927698 16.926134 2.853891
C -9.294151 14.852024 3.361901
H -9.089972 14.994272 4.423455
H -9.931028 14.338307 -0.422427
C -9.711887 12.406329 3.826404
H -8.841292 11.747005 3.714972
H -10.598263 11.792153 3.626267
H -9.746231 12.72082 4.87526
H -12.307838 16.722923 1.236874
H -15.286419 16.868336 0.440556
C -14.469222 18.01885 2.064368
C -13.88606 19.210151 2.43464
C -13.393186 20.13794 1.46891
C -13.634286 19.819447 0.062076
C -14.190949 18.569491 -0.306283
C -14.380819 18.295396 -1.672189
C -14.027988 19.216905 -2.64175
C -13.494905 20.461633 -2.275725
C -13.309821 20.756567 -0.937457
H -14.80983 17.324636 2.833
H -13.715398 19.427183 3.491685
O -12.752437 21.19703 1.794866
H -12.929745 21.730842 -0.626942

H -13.247315 21.194524 -3.040917
H -14.176254 18.979514 -3.6932
H -14.80385 17.334579 -1.96261
K -10.521277 20.173501 0.319193
K -14.099864 22.548352 3.497569

TS_A-B_para_2Kcation_acetonitrile_conf8
C -12.881288 16.087454 1.191193
C -14.578825 17.377734 0.647362
C -14.588224 12.857652 1.991941
C -14.269918 12.758301 3.326454
C -13.569903 13.759063 4.012581
C -13.133475 14.892156 3.386353
C -13.41034 15.030868 1.997079
C -14.180453 14.022576 1.307092
C -14.013786 12.107878 5.444423
H -15.124287 12.073974 1.467185
H -12.536123 15.620053 3.924839
H -13.259502 11.365607 5.733918
O -14.562545 11.745654 4.174391
O -13.404991 13.387746 5.314779
H -14.821978 12.155763 6.184531
N -14.548443 14.26226 -0.011861
S -14.03357 13.232275 -1.1446
O -14.119827 11.815181 -0.782736
O -14.701072 13.686501 -2.390126
C -10.682755 18.531802 3.104024
C -9.604329 18.634717 2.22243
C -11.784314 17.742996 2.779865
H -8.711114 19.189119 2.512283
H -12.617203 17.678654 3.475775
C -9.66758 17.993007 0.980762
C -11.829214 17.027316 1.569203
H -8.832421 18.060402 0.283205
C -10.77719 17.220622 0.651491
H -10.816106 16.70096 -0.30725
H -10.653199 19.041218 4.065685
C -11.406474 13.192734 -0.344441

C -10.081654 13.606137 -0.400649
C -12.301318 13.620667 -1.327766
H -9.383627 13.274835 0.367836
C -9.622785 14.43639 -1.431884
C -11.86758 14.442145 -2.364904
H -12.574674 14.745857 -3.133982
C -10.534527 14.840004 -2.413347
H -10.191807 15.471907 -3.233571
H -11.74953 12.532816 0.450913
C -8.188874 14.867177 -1.48285
H -7.54309 14.065176 -1.863232
H -7.809635 15.128489 -0.487352
H -8.046795 15.731713 -2.141147
H -12.847939 15.815981 0.137158
H -15.078352 16.427684 0.473677
C -14.897539 18.131015 1.801758
C -14.540268 19.449878 1.933279
C -13.858732 20.15227 0.881158
C -13.673565 19.42545 -0.388587
C -14.034172 18.059193 -0.496357
C -13.774654 17.381629 -1.707237
C -13.211732 18.042058 -2.785018
C -12.904891 19.409352 -2.694984
C -13.132611 20.083542 -1.507611
H -15.387327 17.620668 2.632832
H -14.740132 20.002366 2.849196
O -13.388927 21.310254 1.030443
H -12.950327 21.156679 -1.429952
H -12.503684 19.934864 -3.559324
H -13.013041 17.501879 -3.709548
H -13.980802 16.308259 -1.765588
K -16.714533 15.104936 -1.569668
K -10.874967 21.040772 0.684039

TS_A-B_para_2Kcation_acetonitrile_conf9
C -13.04973 15.941324 0.858621
C -14.829736 17.320242 0.720713
C -10.55713 17.548511 3.170786

C -9.875228 18.310712 2.256278
C -10.25412 18.395472 0.915718
C -11.306608 17.680827 0.413767
C -11.998474 16.810993 1.301841
C -11.662327 16.792865 2.703605
C -8.311124 19.36693 1.138653
H -10.254518 17.472314 4.21019
H -11.621312 17.798484 -0.619354
H -7.627144 18.544102 0.881326
O -8.806772 19.172752 2.468118
O -9.448435 19.347354 0.287235
H -7.810799 20.33682 1.070396
N -12.402764 16.017489 3.57946
S -11.667968 14.703242 4.167796
O -10.282307 14.931967 4.592778
O -12.62402 14.14726 5.154216
C -12.331632 14.628433 -2.631579
C -13.448884 13.823534 -2.834253
C -12.175195 15.33426 -1.443142
H -13.568878 13.27613 -3.766816
H -11.263023 15.90275 -1.28703
C -14.401045 13.700584 -1.819941
C -13.140103 15.254254 -0.422456
H -15.264098 13.051811 -1.955489
C -14.240162 14.397273 -0.633567
H -14.976993 14.28688 0.163389
H -11.565947 14.700857 -3.401411
C -10.554401 13.671316 1.882736
C -10.571935 12.892892 0.73084
C -11.607596 13.573147 2.792559
H -9.748509 12.966138 0.020099
C -11.627936 12.014685 0.463817
C -12.67372 12.709082 2.545794
H -13.482148 12.635915 3.27066
C -12.673711 11.935127 1.391924
H -13.501265 11.250979 1.202457
H -9.722703 14.34175 2.093928
C -11.627276 11.162443 -0.76713

H -11.134325 10.198731 -0.582064
H -11.091335 11.643264 -1.593076
H -12.644944 10.9409 -1.107715
H -13.518825 15.404175 1.681849
H -15.457426 16.527463 0.319806
C -14.943436 17.659119 2.090689
C -14.331504 18.770255 2.607397
C -13.628046 19.700537 1.765478
C -13.730109 19.485903 0.31387
C -14.334436 18.30832 -0.195523
C -14.348728 18.097202 -1.588627
C -13.800206 19.030622 -2.450078
C -13.229481 20.209728 -1.945984
C -13.197023 20.426302 -0.580487
H -15.465875 16.959401 2.746146
H -14.338962 18.993667 3.674383
O -12.9554 20.649405 2.242129
H -12.778699 21.345888 -0.171814
H -12.822766 20.951099 -2.630688
H -13.824336 18.856362 -3.523825
H -14.800072 17.185432 -1.980536
K -14.208209 16.149211 5.610506
K -10.587811 21.471331 2.069897

TS_A-B_para_2Kcation_acetonitrile_conf10
C -12.824269 17.090038 1.450991
C -14.633505 17.825442 1.906117
C -9.948309 19.459141 1.998843
C -9.717689 19.830744 0.697585
C -10.522079 19.395972 -0.361229
C -11.569674 18.541298 -0.16097
C -11.829979 18.103934 1.164612
C -11.048413 18.604188 2.261855
C -8.886382 20.652836 -1.199414
H -9.305545 19.784522 2.80978
H -12.139956 18.163982 -1.003775
H -8.01753 20.147009 -1.643729
O -8.749048 20.656065 0.222703

O -10.076061 19.947703 -1.529981
H -8.960533 21.686317 -1.556903
N -11.424199 18.286261 3.558122
S -10.369542 17.532041 4.514016
O -8.964997 17.921918 4.319382
O -10.932951 17.659684 5.879635
C -14.04155 15.264087 -1.670647
C -13.667633 13.95748 -1.358617
C -13.787625 16.296304 -0.775003
H -13.865685 13.151048 -2.061391
H -14.110293 17.309027 -1.020544
C -13.05612 13.690207 -0.137181
C -13.152456 16.04453 0.449382
H -12.773227 12.671494 0.122537
C -12.813591 14.72261 0.764375
H -12.340761 14.512931 1.723964
H -14.538126 15.478367 -2.614897
C -9.625839 15.284072 3.108573
C -9.759476 13.952535 2.726977
C -10.477897 15.804872 4.080464
H -9.090693 13.541568 1.971017
C -10.73434 13.127373 3.298445
C -11.465626 15.006665 4.656325
H -12.11248 15.431823 5.420917
C -11.586536 13.678641 4.265305
H -12.351199 13.049708 4.723302
H -8.852906 15.916944 2.67679
C -10.859602 11.689594 2.89633
H -11.905257 11.35955 2.884861
H -10.329655 11.031468 3.597562
H -10.433747 11.506859 1.903635
H -12.657127 16.670599 2.44517
H -15.144454 17.09605 1.279283
C -14.662175 19.171953 1.443044
C -14.551772 20.237398 2.299869
C -14.555819 20.064895 3.722528
C -14.747752 18.711949 4.231735
C -14.786083 17.614887 3.337599

C -14.936122 16.320752 3.863986
C -15.05692 16.116645 5.226522
C -15.043723 17.205658 6.111829
C -14.893965 18.485438 5.613968
H -14.659991 19.350451 0.366953
H -14.402829 21.246944 1.909197
O -14.410664 21.054116 4.520738
H -14.909959 19.347516 6.28219
H -15.157287 17.039837 7.180819
H -15.168615 15.105691 5.614415
H -14.945261 15.471454 3.179709
K -12.091342 20.003575 5.654696
K -16.330035 22.659753 3.909415

TS_A-B_para_2Kcation_acetonitrile_conf11
C -12.693359 16.735635 1.621584
C -14.682909 16.956944 2.509613
C -11.66164 13.476333 3.153635
C -10.625909 13.916048 3.946544
C -10.285785 15.267797 4.05659
C -10.948596 16.237675 3.357952
C -12.009701 15.832384 2.499535
C -12.391313 14.443189 2.430292
C -8.884639 14.08875 5.328746
H -11.912633 12.424576 3.066133
H -10.620383 17.269498 3.412332
H -7.867462 13.847895 4.993891
O -9.803775 13.17373 4.725103
O -9.230748 15.404594 4.913142
H -8.966026 14.011961 6.419965
N -13.526652 14.100684 1.701756
S -13.343211 13.108197 0.441345
O -12.398656 12.00714 0.650855
O -14.724329 12.778586 0.01286
C -11.493506 20.330501 1.94183
C -11.395541 20.684292 0.594744
C -11.93525 19.060838 2.30663
H -10.970205 21.647819 0.310067

H -12.006447 18.80758 3.361759
C -11.807516 19.775695 -0.385868
C -12.29044 18.110874 1.330927
H -11.728748 20.032477 -1.442524
C -12.270399 18.516283 -0.0173
H -12.572607 17.800604 -0.783634
H -11.192634 21.033477 2.717025
C -11.277549 14.425351 -0.791807
C -10.74481 15.357388 -1.673544
C -12.646705 14.154081 -0.824337
H -9.676539 15.571354 -1.647407
C -11.554738 16.022345 -2.602939
C -13.47414 14.806527 -1.735435
H -14.53622 14.570581 -1.755377
C -12.922647 15.726519 -2.622754
H -13.563931 16.221277 -3.352956
H -10.639869 13.898517 -0.083869
C -10.964736 17.019893 -3.552551
H -11.735076 17.651299 -4.009891
H -10.427005 16.525391 -4.371784
H -10.239598 17.673454 -3.052021
H -13.142003 16.224319 0.771499
H -14.666274 15.870704 2.570984
C -15.364466 17.560266 1.424731
C -15.647745 18.902215 1.41039
C -15.341892 19.754602 2.532049
C -14.790437 19.103159 3.726128
C -14.485663 17.717424 3.71311
C -13.921177 17.137503 4.866643
C -13.668601 17.904024 5.99112
C -13.980455 19.272249 6.001204
C -14.535087 19.861197 4.878752
H -15.58649 16.947115 0.5469
H -16.150831 19.355152 0.553982
O -15.508897 21.00186 2.483335
H -14.788328 20.919214 4.859146
H -13.788113 19.865187 6.892899
H -13.230307 17.442024 6.873716

H -13.673087 16.075325 4.858142
K -16.143619 13.292858 2.121945
K -14.279836 21.928917 0.474804

TS_A-B_para_2Kcation_acetonitrile_conf12
C -12.931358 16.795573 1.54287
C -14.80488 17.450507 2.050299
C -11.120933 18.990953 -0.900568
C -11.181907 18.187178 -2.016146
C -11.883543 16.979912 -2.034059
C -12.520822 16.504242 -0.921025
C -12.463779 17.28641 0.262023
C -11.809083 18.564951 0.25923
C -10.945343 17.26965 -4.034754
H -10.553118 19.916217 -0.892881
H -12.996862 15.529234 -0.934464
H -10.015477 16.744601 -4.292709
O -10.628476 18.412656 -3.234367
O -11.789099 16.414128 -3.276034
H -11.470558 17.59915 -4.93953
N -11.941473 19.377279 1.380007
S -10.634325 19.90065 2.147939
O -9.552403 20.375795 1.262965
O -11.147923 20.879927 3.136929
C -13.936916 13.122972 1.456198
C -13.109761 12.611411 2.456308
C -13.902893 14.477061 1.147147
H -13.137382 11.550802 2.696906
H -14.575366 14.871329 0.383905
C -12.261515 13.466924 3.153516
C -13.038765 15.348369 1.827745
H -11.622847 13.077395 3.944419
C -12.234578 14.825315 2.850288
H -11.576099 15.498613 3.400211
H -14.615489 12.463151 0.919468
C -9.095264 17.641689 2.449878
C -8.684202 16.500861 3.129733
C -9.999621 18.510515 3.059634

H -7.975444 15.821241 2.65735
C -9.15929 16.209973 4.414376
C -10.500433 18.236044 4.330952
H -11.209456 18.925841 4.784818
C -10.073014 17.094956 5.00032
H -10.455702 16.882422 5.999605
H -8.710234 17.874029 1.458715
C -8.693854 14.987271 5.143952
H -9.408984 14.675552 5.913583
H -7.73614 15.16514 5.650721
H -8.537341 14.143309 4.462328
H -12.451373 17.344026 2.353376
H -15.25555 16.461488 1.985839
C -15.208957 18.386866 1.053992
C -15.172101 19.738398 1.258922
C -14.781072 20.293547 2.519422
C -14.449746 19.359101 3.593859
C -14.570873 17.961429 3.386925
C -14.35517 17.097292 4.477181
C -13.993192 17.591773 5.717862
C -13.795801 18.97022 5.902499
C -14.019499 19.837161 4.847848
H -15.506418 18.000986 0.078176
H -15.458231 20.433636 0.469192
O -14.76521 21.553552 2.719304
H -13.7785 20.897909 4.937625
H -13.434826 19.345655 6.858402
H -13.829164 16.90549 6.546524
H -14.458905 16.023137 4.325246
K -12.553 22.161877 1.250822
K -16.452349 21.761008 4.712289

TS_A-B_para_2Kcation_acetonitrile_conf13
C -12.879526 16.403878 0.44009
C -14.649678 17.444687 0.692273
C -11.603346 17.302764 -2.981485
C -12.17875 16.28846 -3.709809
C -12.964173 15.296012 -3.114855

C -13.208983 15.285377 -1.770005
C -12.647986 16.325203 -0.978903
C -11.835937 17.34384 -1.588041
C -12.792437 14.840071 -5.289772
H -10.961288 18.047853 -3.443041
H -13.78927 14.486057 -1.319663
H -12.080893 14.079606 -5.638923
O -12.087955 16.060593 -5.043366
O -13.390479 14.417982 -4.070993
H -13.571365 15.019666 -6.040395
N -11.275833 18.392317 -0.86069
S -9.892263 18.105453 -0.095916
O -9.472306 19.416378 0.458271
O -9.939623 16.967752 0.839484
C -14.239146 13.212806 2.034223
C -13.261228 12.987011 3.000433
C -14.146407 14.313291 1.188559
H -13.333884 12.123705 3.658657
H -14.934842 14.482119 0.455082
C -12.19625 13.876202 3.122278
C -13.074879 15.212138 1.294986
H -11.427322 13.707624 3.873742
C -12.108269 14.985388 2.287971
H -11.267734 15.673439 2.363943
H -15.081662 12.530723 1.939076
C -7.969766 18.63376 -2.005452
C -7.119339 18.271123 -3.042875
C -8.692973 17.650311 -1.333947
H -6.541744 19.037995 -3.559041
C -6.98359 16.933765 -3.434151
C -8.574091 16.311652 -1.704676
H -9.137624 15.552774 -1.16471
C -7.725232 15.964967 -2.74953
H -7.6287 14.918346 -3.037796
H -8.060048 19.672695 -1.694218
C -6.04298 16.546785 -4.533715
H -5.043569 16.320691 -4.138723
H -5.920597 17.350805 -5.267549

H -6.384087 15.651471 -5.064767
H -12.24989 17.15505 0.908503
H -15.243364 16.567905 0.435386
C -14.513619 17.771085 2.067059
C -14.156115 19.033424 2.483749
C -14.019073 20.118865 1.567637
C -14.397882 19.858836 0.179213
C -14.696696 18.541003 -0.248962
C -15.003978 18.328584 -1.605048
C -15.025595 19.378305 -2.505177
C -14.763892 20.687398 -2.073738
C -14.458861 20.918957 -0.744863
H -14.579246 16.962715 2.79538
H -13.882362 19.196713 3.528775
O -13.569349 21.264873 1.914165
H -14.286907 21.93183 -0.377809
H -14.821569 21.514792 -2.77828
H -15.262255 19.190183 -3.550494
H -15.220498 17.315408 -1.942284
K -11.455082 21.004026 -0.042307
K -14.441244 22.059669 4.187785

TS_A-B_para_2Kcation_acetonitrile_conf14
C -12.549042 16.533842 0.922731
C -14.69173 17.20485 0.712659
C -13.122277 12.946921 1.823829
C -12.612053 12.946581 3.103193
C -12.108409 14.103885 3.706418
C -12.084206 15.304257 3.054032
C -12.590755 15.352924 1.724296
C -13.129974 14.163301 1.111821
C -11.885981 12.412655 5.142776
H -13.503512 12.041922 1.358392
H -11.635185 16.174057 3.52204
H -10.929097 11.899653 5.300901
O -12.498944 11.901081 3.954012
O -11.662324 13.805335 4.961015
H -12.562142 12.253228 5.991703

N -13.754681 14.208078 -0.134621
S -12.881289 13.983556 -1.465278
O -13.874009 14.027168 -2.569726
O -11.715582 14.876562 -1.574886
C -11.033572 19.546246 2.675237
C -10.17992 20.006941 1.670069
C -11.850695 18.440514 2.451405
H -9.486042 20.823388 1.872546
H -12.513365 18.096048 3.241581
C -10.197891 19.390827 0.413784
C -11.825032 17.763241 1.217785
H -9.530712 19.734405 -0.375976
C -11.027706 18.297912 0.185179
H -11.018948 17.792566 -0.780761
H -11.039231 20.028052 3.651618
C -13.000299 11.255438 -1.850664
C -12.527684 9.959398 -1.690482
C -12.229824 12.327839 -1.403649
H -13.121769 9.121004 -2.053706
C -11.29361 9.708913 -1.076796
C -10.998083 12.104881 -0.791245
H -10.402854 12.952376 -0.457106
C -10.54166 10.801108 -0.631687
H -9.576846 10.625027 -0.156863
H -13.951096 11.444933 -2.345222
C -10.780467 8.309493 -0.932775
H -10.275835 7.977403 -1.84966
H -11.5898 7.59624 -0.741526
H -10.05331 8.224359 -0.118536
H -12.61322 16.322564 -0.139361
H -14.832446 16.143215 0.51997
C -15.050319 17.743647 1.967106
C -15.136968 19.098664 2.180818
C -14.903586 20.041592 1.12301
C -14.692777 19.483355 -0.225183
C -14.575881 18.083466 -0.417411
C -14.278866 17.599922 -1.710423
C -14.152733 18.463908 -2.783161

C -14.333384 19.844998 -2.602888
C -14.593506 20.340041 -1.336613
H -15.203488 17.056769 2.801205
H -15.35929 19.503165 3.166222
O -14.820756 21.282283 1.320566
H -14.781625 21.403484 -1.178086
H -14.277401 20.516993 -3.457068
H -13.91432 18.068979 -3.76873
H -14.095127 16.531535 -1.852648
K -16.169475 14.488196 -1.44158
K -12.437043 21.825001 0.543169

TS_A-B_para_2Kcation_acetonitrile_conf15

C -12.905536 16.277173 0.45406
C -14.903255 17.265477 0.630433
C -10.620469 18.186701 2.766263
C -10.229869 19.188197 1.913319
C -10.721038 19.291608 0.614008
C -11.605643 18.38324 0.098632
C -11.991167 17.286684 0.918113
C -11.5203 17.207131 2.275709
C -9.052052 20.679166 0.822435
H -10.223426 18.089726 3.773222
H -12.031659 18.52285 -0.890552
H -8.219657 20.065548 0.445114
O -9.379807 20.261795 2.153974
O -10.217484 20.46351 0.043016
H -8.79383 21.742023 0.822574
N -11.905537 16.197746 3.147632
S -11.20535 14.753058 2.979428
O -11.76028 13.939996 4.088135
O -11.270478 14.201915 1.619307
C -12.387011 15.549247 -3.238131
C -13.33246 14.546599 -3.429795
C -12.218282 16.131472 -1.986097
H -13.461694 14.095532 -4.411236
H -11.430621 16.866709 -1.85404
C -14.093044 14.101124 -2.346626

C -12.999963 15.724303 -0.890403
H -14.811321 13.294738 -2.477442
C -13.920645 14.676956 -1.099475
H -14.503576 14.316302 -0.25133
H -11.762262 15.874116 -4.067503
C -9.000526 14.952508 4.632767
C -7.66859 15.249725 4.891904
C -9.473831 15.002834 3.322814
H -7.293109 15.19246 5.913582
C -6.792397 15.609378 3.859958
C -8.621816 15.358082 2.278757
H -9.001433 15.375897 1.258475
C -7.292604 15.660112 2.554733
H -6.62203 15.927761 1.738198
H -9.671943 14.651801 5.434805
C -5.35002 15.894469 4.145567
H -4.770225 14.964902 4.217787
H -5.21863 16.422029 5.09703
H -4.889537 16.497721 3.356033
H -13.188237 15.571943 1.228932
H -15.398214 16.404304 0.187142
C -14.943543 17.427286 2.037508
C -14.53223 18.586071 2.639915
C -14.139394 19.733014 1.866813
C -14.339069 19.652424 0.412107
C -14.728416 18.429806 -0.1914
C -14.843737 18.36805 -1.594543
C -14.597103 19.484902 -2.373043
C -14.23909 20.701921 -1.772303
C -14.113066 20.775938 -0.397296
H -15.238304 16.565497 2.640188
H -14.475597 18.687863 3.723474
O -13.646086 20.757433 2.403638
H -13.861751 21.718128 0.089602
H -14.071349 21.582491 -2.38906
H -14.695417 19.422917 -3.454906
H -15.131186 17.425289 -2.06031
K -13.533047 15.484856 5.179024

K -11.599586 22.171917 2.131715

TS_A-B_para_2Kcation_acetonitrile_conf16
C -12.861741 16.350686 0.4685
C -14.626112 17.392492 0.755711
C -11.612067 17.315985 -2.944863
C -12.195612 16.317504 -3.688523
C -12.978151 15.314889 -3.106987
C -13.211914 15.277874 -1.760676
C -12.641965 16.300447 -0.953635
C -11.832723 17.329382 -1.548938
C -12.822572 14.90024 -5.291217
H -10.972399 18.068948 -3.396882
H -13.789865 14.47072 -1.321464
H -12.113048 14.145798 -5.657316
O -12.116286 16.116031 -5.027091
O -13.414421 14.45711 -4.076929
H -13.605311 15.09317 -6.034436
N -11.263276 18.360881 -0.80459
S -9.873186 18.0522 -0.060039
O -9.445 19.347988 0.522602
O -9.914483 16.891344 0.846697
C -14.223951 13.132641 2.004413
C -13.238833 12.880309 2.956622
C -14.13249 14.251116 1.182631
H -13.310296 12.003037 3.596239
H -14.926203 14.440219 0.459873
C -12.168018 13.761023 3.088716
C -13.05526 15.141834 1.299434
H -11.393502 13.571801 3.829458
C -12.081208 14.888089 2.278518
H -11.236294 15.569882 2.362293
H -15.070922 12.457222 1.901627
C -7.973022 18.626659 -1.978385
C -7.134267 18.289513 -3.033604
C -8.686315 17.62669 -1.320616
H -6.564563 19.069195 -3.539275
C -7.000255 16.96148 -3.456325

C -8.568873 16.297062 -1.72252
H -9.124597 15.525121 -1.193115
C -7.731498 15.975899 -2.784814
H -7.636069 14.936216 -3.097498
H -8.061823 19.658226 -1.643098
C -6.071955 16.602035 -4.575516
H -5.062708 16.387434 -4.199574
H -5.974058 17.416462 -5.30159
H -6.407861 15.70783 -5.111622
H -12.225756 17.089783 0.947338
H -15.224825 16.523409 0.484819
C -14.479391 17.689545 2.136126
C -14.1148 18.941755 2.576704
C -13.980016 20.045276 1.682193
C -14.369197 19.815703 0.291341
C -14.675904 18.508268 -0.162157
C -14.993962 18.325433 -1.520082
C -15.018375 19.393944 -2.397788
C -14.748644 20.692747 -1.940903
C -14.432849 20.895183 -0.609787
H -14.543136 16.866308 2.847779
H -13.834119 19.083149 3.623103
O -13.523966 21.182381 2.049396
H -14.254457 21.899522 -0.222971
H -14.808511 21.534961 -2.627472
H -15.263557 19.228641 -3.445002
H -15.217148 17.320435 -1.876841
K -11.423843 20.954378 0.074425
K -14.389967 21.945286 4.334299

TS_A-B_para_2Kcation_acetonitrile_conf17
C -12.533999 16.465947 0.953293
C -14.674347 17.154455 0.767767
C -13.123859 12.865124 1.785557
C -12.608097 12.836631 3.062386
C -12.09523 13.978958 3.686128
C -12.067045 15.191839 3.057491
C -12.579108 15.269597 1.731266

C -13.127831 14.095412 1.097717
C -11.878233 12.258863 5.088635
H -13.512629 11.971659 1.304371
H -11.611244 16.049774 3.540628
H -10.92468 11.736399 5.235149
O -12.497117 11.773873 3.891888
O -11.645172 13.653116 4.932429
H -12.553849 12.088267 5.935877
N -13.757998 14.168454 -0.144614
S -12.892208 13.967171 -1.483992
O -13.890371 14.036464 -2.582189
O -11.723736 14.857972 -1.582084
C -10.990352 19.433015 2.757973
C -10.136996 19.90681 1.758698
C -11.816362 18.337742 2.515972
H -9.436369 20.714066 1.974343
H -12.478517 17.982835 3.301938
C -10.164107 19.315247 0.490808
C -11.800431 17.684456 1.269318
H -9.497182 19.669103 -0.294614
C -11.002989 18.23321 0.244198
H -11.00179 17.746877 -0.731472
H -10.988844 19.89577 3.743538
C -13.022422 11.247739 -1.923787
C -12.552235 9.947417 -1.794007
C -12.24617 12.308466 -1.459301
H -13.150774 9.118396 -2.171142
C -11.31488 9.680948 -1.193817
C -11.011235 12.069525 -0.859491
H -10.411565 12.908267 -0.511536
C -10.557276 10.761539 -0.730394
H -9.589914 10.573014 -0.265651
H -13.975783 11.45009 -2.4082
C -10.804411 8.277513 -1.083237
H -10.306825 7.963934 -2.010438
H -11.614311 7.562329 -0.90186
H -10.072015 8.173145 -0.275969
H -12.605358 16.276483 -0.112446

H -14.822628 16.097417 0.556238
C -15.022344 17.672164 2.033945
C -15.098378 19.023564 2.273283
C -14.864208 19.984439 1.231896
C -14.665341 19.45001 -0.127692
C -14.55877 18.053138 -0.346431
C -14.2721 17.591827 -1.649843
C -14.146136 18.474702 -2.707067
C -14.317107 19.853326 -2.500335
C -14.567056 20.326473 -1.223698
H -15.175795 16.970939 2.855972
H -15.312505 19.411133 3.267286
O -14.771086 21.220601 1.452069
H -14.748012 21.387845 -1.044643
H -14.261687 20.540639 -3.342275
H -13.915487 18.096578 -3.701041
H -14.096007 16.525185 -1.812859
K -16.17761 14.486429 -1.43389
K -12.386164 21.758878 0.674292

TS_A-B_para_2Kcation_acetonitrile_conf18
C -12.885438 16.225785 0.477054
C -14.881555 17.213147 0.677319
C -10.589369 18.081622 2.821694
C -10.20067 19.101116 1.989378
C -10.69716 19.233968 0.694752
C -11.584413 18.337788 0.162747
C -11.968385 17.224154 0.959613
C -11.492742 17.11452 2.313382
C -9.02658 20.615678 0.926886
H -10.188472 17.961847 3.824664
H -12.014034 18.499626 -0.821519
H -8.195904 20.010674 0.532251
O -9.348331 20.168164 2.250366
O -10.195341 20.417905 0.148013
H -8.768108 21.678213 0.949937
N -11.876464 16.086833 3.164464
S -11.177486 14.645906 2.962523

O -11.730952 13.808444 4.053679
O -11.245693 14.12562 1.590482
C -12.375994 15.573894 -3.23035
C -13.32259 14.576002 -3.440455
C -12.204003 16.130069 -1.966963
H -13.454344 14.145285 -4.430661
H -11.415749 16.862174 -1.821363
C -14.081201 14.108897 -2.365034
C -12.98354 15.700854 -0.878228
H -14.800505 13.306006 -2.510874
C -13.905557 14.658761 -1.106664
H -14.487034 14.281346 -0.264814
H -11.752933 15.915332 -4.054305
C -8.969918 14.807211 4.616164
C -7.637009 15.096393 4.879418
C -9.445081 14.885948 3.308292
H -7.260072 15.017035 5.89908
C -6.761689 15.47587 3.853876
C -8.594028 15.261563 2.270596
H -8.975295 15.301519 1.251553
C -7.263813 15.555098 2.550767
H -6.593958 15.838505 1.738972
H -9.640714 14.490982 5.41274
C -5.318175 15.751572 4.142927
H -4.739387 14.819678 4.188396
H -5.183449 16.253214 5.107826
H -4.859121 16.375193 3.368511
H -13.167214 15.504962 1.237797
H -15.3787 16.362788 0.215943
C -14.919961 17.344456 2.087665
C -14.505328 18.488967 2.714603
C -14.110461 19.651355 1.965963
C -14.31219 19.602914 0.510191
C -14.705275 18.394709 -0.119211
C -14.821834 18.363578 -1.523272
C -14.572821 19.496372 -2.277616
C -14.210912 20.699124 -1.651
C -14.083599 20.74307 -0.274825

H -15.216286 16.470569 2.671928
H -14.447508 18.567174 3.800054
O -13.613503 20.662483 2.524196
H -13.828351 21.673492 0.232168
H -14.040838 21.592296 -2.248709
H -14.672114 19.458064 -3.360485
H -15.111938 17.43183 -2.009105
K -13.504109 15.324733 5.178309
K -11.564814 22.079754 2.297323

TS_A-B_para_2Kcation_acetonitrile_conf19
C 1.471219 0.211626 0.137531
C 2.732581 -0.380499 -1.338384
C -1.723057 1.606156 -1.295842
C -1.373397 2.933731 -1.388048
C -0.120526 3.399017 -0.985307
C 0.828874 2.554984 -0.477376
C 0.516138 1.17424 -0.375839
C -0.770569 0.691383 -0.793027
C -1.329188 5.145765 -1.657722
H -2.713137 1.25417 -1.572731
H 1.781936 2.946399 -0.138441
H -1.8157 5.795342 -0.917278
O -2.123207 3.969859 -1.840555
O -0.04846 4.749197 -1.183439
H -1.222861 5.661596 -2.619544
N -1.12225 -0.659969 -0.752841
S -1.665466 -1.241345 0.641939
O -2.07317 -2.637742 0.34941
O -0.753525 -1.029136 1.779933
C 4.273574 1.719408 2.243945
C 4.139247 0.96426 3.408552
C 3.419011 1.503751 1.168988
H 4.808676 1.134758 4.249215
H 3.571098 2.077085 0.254959
C 3.150274 -0.012403 3.484669
C 2.412482 0.527853 1.234514
H 3.03901 -0.608829 4.388025

C 2.301847 -0.23877 2.405149
H 1.517231 -0.99115 2.463075
H 5.052189 2.475992 2.170071
C -4.383245 -0.763873 0.558533
C -5.514836 0.008712 0.78769
C -3.14944 -0.339884 1.048665
H -6.482612 -0.330132 0.417719
C -5.437118 1.214732 1.495086
C -3.047223 0.856365 1.756899
H -2.079026 1.17123 2.142264
C -4.187174 1.622915 1.972035
H -4.108962 2.556575 2.528761
H -4.45116 -1.709278 0.023738
C -6.666831 2.027136 1.762304
H -6.429685 3.084772 1.919355
H -7.184013 1.677646 2.665806
H -7.386946 1.959449 0.93928
H 1.008374 -0.764765 0.26388
H 3.415699 0.460475 -1.218599
C 1.921416 -0.373342 -2.509639
C 1.383957 -1.515524 -3.03949
C 1.646315 -2.80098 -2.472375
C 2.572465 -2.855572 -1.343382
C 3.193636 -1.670549 -0.86314
C 4.177175 -1.788863 0.137976
C 4.497504 -3.018745 0.68348
C 3.823226 -4.178884 0.2686
C 2.877264 -4.091752 -0.73478
H 1.664805 0.590724 -2.948862
H 0.732464 -1.470056 -3.91297
O 1.110412 -3.86322 -2.940547
H 2.290521 -4.965792 -1.024024
H 4.029485 -5.131339 0.752818
H 5.253868 -3.081203 1.462967
H 4.675103 -0.889534 0.497602
K -1.403432 -3.01543 -2.12816
K 3.240337 -5.20956 -3.655063

TS_A-B_para_2Kcation_acetonitrile_conf20

C 1.260866 -0.230059 0.714461
C 2.852785 -0.123313 -0.812274
C 3.016064 1.640464 3.443333
C 3.131002 0.636974 4.380494
C 2.6902 -0.665179 4.133662
C 2.101732 -1.0167 2.949696
C 1.944488 -0.010029 1.957207
C 2.429223 1.325241 2.201325
C 3.506775 -0.58523 6.207084
H 3.357685 2.653027 3.641452
H 1.708369 -2.018104 2.813131
H 2.852742 -0.51823 7.085938
O 3.647799 0.716977 5.629775
O 2.920999 -1.441295 5.233834
H 4.498273 -0.963894 6.484782
N 2.446475 2.282334 1.187022
S 1.187116 3.252536 0.962702
O 1.609736 4.158938 -0.134381
O -0.096379 2.548637 0.797258
C -0.028306 -3.791323 0.357552
C -1.311227 -3.535952 -0.137937
C 0.854196 -2.742516 0.59795
H -2.029589 -4.34937 -0.248855
H 1.853509 -2.962135 0.966855
C -1.677399 -2.225354 -0.454722
C 0.469091 -1.407314 0.367336
H -2.671923 -2.011122 -0.842804
C -0.790239 -1.178305 -0.219514
H -1.089355 -0.150925 -0.426508
H 0.274835 -4.810215 0.597372
C 1.7274 5.430772 2.569138
C 1.663387 6.134955 3.76492
C 1.011212 4.242916 2.432346
H 2.209696 7.072444 3.867461
C 0.899696 5.666906 4.841448
C 0.242956 3.757047 3.48895
H -0.320658 2.834626 3.363606

C 0.194752 4.469091 4.682021
H -0.408856 4.092684 5.507491
H 2.30567 5.809554 1.728543
C 0.813532 6.447473 6.116593
H 0.585336 5.804291 6.973055
H 0.019169 7.203761 6.064051
H 1.745587 6.981562 6.331385
H 0.847577 0.685528 0.307993
H 3.3839 0.517588 -0.112692
C 2.091971 0.484811 -1.84581
C 1.622883 -0.226356 -2.919117
C 1.937298 -1.619175 -3.087972
C 2.861292 -2.217625 -2.109071
C 3.328348 -1.463691 -1.001702
C 4.206482 -2.076211 -0.084274
C 4.612124 -3.387302 -0.257598
C 4.164244 -4.126353 -1.364614
C 3.302511 -3.54154 -2.275543
H 1.776099 1.524223 -1.7099
H 0.978353 0.232599 -3.66585
O 1.426117 -2.316778 -4.002805
H 2.988927 -4.076189 -3.173021
H 4.514416 -5.145763 -1.513985
H 5.294193 -3.843245 0.457391
H 4.556052 -1.500883 0.774138
K 3.99153 3.379517 -0.788442
K -0.112485 -4.025908 -2.953873

TS_A-B_para_2Kcation_acetonitrile_conf21
C 1.473133 0.267021 0.227279
C 2.804903 -0.422851 -1.468001
C -0.032823 -2.942074 1.508868
C -1.175446 -2.97204 0.752611
C -1.497477 -1.955516 -0.144744
C -0.676228 -0.882989 -0.353933
C 0.529367 -0.800413 0.398428
C 0.827938 -1.82521 1.37226
C -3.276806 -3.177311 0.174305

H 0.181911 -3.689975 2.266866
H -0.958205 -0.104241 -1.052877
H -3.776013 -2.613905 0.97765
O -2.186528 -3.92285 0.72845
O -2.704061 -2.284512 -0.769685
H -3.971652 -3.856722 -0.32735
N 1.899631 -1.753329 2.246989
S 1.756514 -0.719658 3.480624
O 2.942232 -0.974289 4.330113
O 1.478795 0.670711 3.091266
C 0.197361 3.423356 -1.491428
C 0.80968 4.390673 -0.695941
C 0.391669 2.073288 -1.230546
H 0.653237 5.447126 -0.902973
H -0.061656 1.345473 -1.898881
C 1.631371 3.994299 0.355793
C 1.207131 1.652575 -0.164664
H 2.116317 4.739423 0.982713
C 1.837402 2.644565 0.610601
H 2.45226 2.33428 1.45302
H -0.429772 3.723246 -2.328509
C 0.437331 -2.235292 5.377508
C -0.705212 -2.723041 5.999103
C 0.319828 -1.257487 4.391679
H -0.612498 -3.473794 6.783836
C -1.977772 -2.257187 5.644185
C -0.934832 -0.776568 4.022062
H -1.01052 -0.000755 3.261528
C -2.070706 -1.281187 4.646727
H -3.05234 -0.897779 4.367436
H 1.425336 -2.585469 5.670561
C -3.200397 -2.767927 6.342689
H -4.113016 -2.552698 5.77699
H -3.316377 -2.301242 7.329569
H -3.152323 -3.849916 6.511195
H 2.314118 0.179956 0.909578
H 3.360255 0.51365 -1.47862
C 1.806261 -0.63569 -2.445513

C 1.184929 -1.851252 -2.572942
C 1.574451 -2.985755 -1.779343
C 2.829208 -2.848144 -1.026299
C 3.450572 -1.579356 -0.905662
C 4.715554 -1.499941 -0.28848
C 5.364427 -2.644676 0.142136
C 4.730653 -3.898771 0.047412
C 3.465789 -3.987639 -0.5109
H 1.478258 0.209428 -3.048814
H 0.359399 -1.985864 -3.272805
O 0.917948 -4.058861 -1.766742
H 2.96226 -4.946104 -0.62271
H 5.255422 -4.801871 0.359791
H 6.383971 -2.576083 0.524725
H 5.207989 -0.529927 -0.218267
K 4.271665 -2.786737 3.029885
K -1.487749 -4.730567 -2.020122

Table S11. Computed energies for the neutral transition state conformational analysis in acetonitrile.

	M06-L/BS1							TPSS0-D4/BS2
	1st frequency (cm-1)	Electronic energy	ZPE corr	Thermal corr	Enthalpy corr:	Gibbs corr	Electronic energy in acetonitrile	Electronic energy
pre-ortho TS								
TS_A-B_ortho_2Kcation_acetonitrile	-370.6	-2081.14380	0.48085	0.51632	0.51727	0.40941	-2081.20221	-3223.11125
TS_A-B_ortho_2Kcation_acetonitrile_conf2	-86.2	-2081.12895	0.47990	0.51567	0.51662	0.40861	-2081.18860	-3223.09073
TS_A-B_ortho_2Kcation_acetonitrile_conf3	-181.2	-2081.12897	0.48036	0.51585	0.51679	0.40970	-2081.18812	-3223.09050
TS_A-B_ortho_2Kcation_acetonitrile_conf4	-7.4	-2081.12396	0.47958	0.51521	0.51615	0.40826	-2081.18593	-3223.07999
TS_A-B_ortho_2Kcation_acetonitrile_conf5	-370.5	-2081.14383	0.48110	0.51650	0.51745	0.41024	-2081.20230	-3223.11123
TS_A-B_ortho_2Kcation_acetonitrile_conf6	-361.3	-2081.10977	0.48074	0.51613	0.51707	0.40979	-2081.17457	-3223.07754
TS_A-B_ortho_2Kcation_acetonitrile_conf7	-312.5	-2081.14372	0.48040	0.51594	0.51689	0.40968	-2081.20176	-3223.10833
TS_A-B_ortho_2Kcation_acetonitrile_conf8	-398.5	-2081.10508	0.48016	0.51576	0.51670	0.40968	-2081.16951	-3223.06719
TS_A-B_ortho_2Kcation_acetonitrile_conf9	-352.5	-2081.11886	0.48042	0.51594	0.51688	0.40855	-2081.18000	-3223.08565
TS_A-B_ortho_2Kcation_acetonitrile_conf10	-399.2	-2081.10504	0.48021	0.51582	0.51676	0.40924	-2081.16954	-3223.06722
TS_A-B_ortho_2Kcation_acetonitrile_conf11	-352.6	-2081.11887	0.48036	0.51588	0.51683	0.40860	-2081.18004	-3223.08562
TS_A-B_ortho_2Kcation_acetonitrile_conf12	-317.5	-2081.12382	0.48051	0.51611	0.51705	0.40900	-2081.18356	-3223.08838
TS_A-B_ortho_2Kcation_acetonitrile_conf13	-371.0	-2081.14380	0.48083	0.51627	0.51721	0.41003	-2081.20228	-3223.11123
TS_A-B_ortho_2Kcation_acetonitrile_conf14	-314.7	-2081.14371	0.48058	0.51609	0.51704	0.41010	-2081.20181	-3223.10843
TS_A-B_ortho_2Kcation_acetonitrile_conf15	-358.7	-2081.10983	0.48081	0.51617	0.51712	0.41004	-2081.17448	-3223.07750
TS_A-B_ortho_2Kcation_acetonitrile_conf16	-372.2	-2081.14382	0.48077	0.51625	0.51720	0.40947	-2081.20234	-3223.11118
TS_A-B_ortho_2Kcation_acetonitrile_conf17	-316.7	-2081.14370	0.48057	0.51607	0.51701	0.41001	-2081.20170	-3223.10840
TS_A-B_ortho_2Kcation_acetonitrile_conf18	-350.0	-2081.10171	0.48029	0.51599	0.51694	0.40708	-2081.16635	-3223.06693
TS_A-B_ortho_2Kcation_acetonitrile_conf19	-369.3	-2081.14384	0.48112	0.51648	0.51742	0.41059	-2081.20232	-3223.11133
TS_A-B_ortho_2Kcation_acetonitrile_conf20	-314.9	-2081.14374	0.48050	0.51600	0.51695	0.41032	-2081.20179	-3223.10846
TS_A-B_ortho_2Kcation_acetonitrile_conf21	-359.3	-2081.10976	0.48048	0.51592	0.51686	0.40895	-2081.17433	-3223.07744
pre-para TS								
TS_A-B_para_2Kcation_acetonitrile	-260.1	-2081.11516	0.48060	0.51575	0.51669	0.41103	-2081.17784	-3223.08080
TS_A-B_para_2Kcation_acetonitrile_conf2	-298.8	-2081.11437	0.48082	0.51620	0.51715	0.41073	-2081.17767	-3223.07261
TS_A-B_para_2Kcation_acetonitrile_conf3	-260.5	-2081.11521	0.48077	0.51576	0.51671	0.41221	-2081.17767	-3223.08077
TS_A-B_para_2Kcation_acetonitrile_conf4	-337.8	-2081.10218	0.48060	0.51608	0.51703	0.40977	-2081.16927	-3223.06236
TS_A-B_para_2Kcation_acetonitrile_conf5	-317.8	-2081.11022	0.48045	0.51604	0.51698	0.40865	-2081.17452	-3223.07283
TS_A-B_para_2Kcation_acetonitrile_conf6	-372.2	-2081.10424	0.48038	0.51597	0.51692	0.40937	-2081.16790	-3223.06625
TS_A-B_para_2Kcation_acetonitrile_conf7	-365.4	-2081.10597	0.48072	0.51641	0.51736	0.40803	-2081.17156	-3223.06914
TS_A-B_para_2Kcation_acetonitrile_conf8	-220.0	-2081.11064	0.48076	0.51645	0.51739	0.40853	-2081.17353	-3223.07051
TS_A-B_para_2Kcation_acetonitrile_conf9	-282.3	-2081.10804	0.48055	0.51598	0.51693	0.40818	-2081.17229	-3223.07432

TS_A-B_para_2Kcation_acetonitrile_conf10	-365.6	-2081.10595	0.48057	0.51632	0.51726	0.40735	-2081.17156	-3223.06916
TS_A-B_para_2Kcation_acetonitrile_conf11	-218.9	-2081.11065	0.48078	0.51645	0.51740	0.40858	-2081.17353	-3223.07049
TS_A-B_para_2Kcation_acetonitrile_conf12	-282.1	-2081.10807	0.48078	0.51615	0.51710	0.40863	-2081.17227	-3223.07438
TS_A-B_para_2Kcation_acetonitrile_conf13	-359.4	-2081.10185	0.48043	0.51617	0.51712	0.40757	-2081.16692	-3223.06539
TS_A-B_para_2Kcation_acetonitrile_conf14	-302.7	-2081.10694	0.48056	0.51635	0.51729	0.40729	-2081.17050	-3223.06941
TS_A-B_para_2Kcation_acetonitrile_conf15	-248.2	-2081.10969	0.48025	0.51564	0.51659	0.40838	-2081.17183	-3223.07739
TS_A-B_para_2Kcation_acetonitrile_conf16	-364.5	-2081.10798	0.48069	0.51615	0.51710	0.40918	-2081.17376	-3223.06923
TS_A-B_para_2Kcation_acetonitrile_conf15	-365.1	-2081.10804	0.48069	0.51613	0.51708	0.40984	-2081.17387	-3223.06931
TS_A-B_para_2Kcation_acetonitrile_conf18	-294.9	-2081.11434	0.48063	0.51610	0.51705	0.41011	-2081.17765	-3223.07250
TS_A-B_para_2Kcation_acetonitrile_conf19	-354.0	-2081.10376	0.48041	0.51595	0.51689	0.40948	-2081.17002	-3223.06551
TS_A-B_para_2Kcation_acetonitrile_conf20	-283.8	-2081.11704	0.48076	0.51556	0.51651	0.41269	-2081.17816	-3223.08356
TS_A-B_para_2Kcation_acetonitrile_conf21	-316.0	-2081.11026	0.48042	0.51600	0.51695	0.40876	-2081.17448	-3223.07248

Table S12. Computed energies for the neutral path in acetonitrile.

	M06-L/BS1							TPSS0-D4/BS2
	1st frequency (cm-1)	Electronic energy	ZPE corr	Thermal corr	Enthalpy corr:	Gibbs corr	Electronic energy in acetonitrile	Electronic energy
Path for ortho product								
InterA_pre-ortho_2Kcation_acetonitrile	8.0	-2081.15403	0.48291	0.51869	0.51963	0.40885	-2081.21509	-3223.12731
TS_A-B_ortho_2Kcation_acetonitrile	-370.6	-2081.14380	0.48085	0.51632	0.51727	0.40941	-2081.20221	-3223.11125
InterB_ortho_2Kcation_acetonitrile	17.2	-2081.15456	0.48012	0.51644	0.51738	0.40843	-2081.21221	-3223.11366
Path for para product								
InterA_pre-para_2Kcation_acetonitrile	18.1	-2081.13249	0.48311	0.51809	0.51904	0.41371	-2081.19569	-3223.10466
TS_A-B_para_2Kcation_acetonitrile	-260.1	-2081.11516	0.48060	0.51575	0.51669	0.41103	-2081.17784	-3223.08080
InterB_para_2Kcation_acetonitrile	17.3	-2081.11815	0.47970	0.51588	0.51683	0.40824	-2081.18183	-3223.08011

InterA_pre-ortho_2Kcation_acetonitrile

C -0.548781 1.066891 -0.32385
C -0.360355 1.666813 1.141502
C 0.687326 3.857744 1.639508
C -4.306456 0.476197 -0.553713
C -4.652371 1.462396 -1.450037
C -3.706821 2.304791 -2.018803
C -2.370543 2.205551 -1.707119
C -1.980853 1.228129 -0.771565
C -2.945426 0.360623 -0.199274
C -5.699764 2.825783 -2.869382
H -5.049733 -0.209285 -0.156087
H -1.64214 2.847047 -2.193912
H -6.013319 2.497834 -3.869856
O -5.889394 1.756407 -1.941383
O -4.315882 3.164779 -2.89005
H -6.286073 3.69597 -2.545506
N -2.489993 -0.683439 0.642416
S -2.977683 -0.796715 2.132736
O -2.902728 0.460453 2.935712
O -2.16462 -1.906871 2.736901
C 1.856999 3.091899 -2.531668
C 2.682704 2.081704 -3.016903
C 0.806492 2.792135 -1.66877
H 3.501565 2.319631 -3.692264
H 0.183411 3.60136 -1.295068
C 2.451765 0.764954 -2.630363
C 0.553107 1.472332 -1.27251
H 3.088975 -0.035525 -3.001111
C 1.400594 0.474039 -1.767306
H 1.223551 -0.562857 -1.468718
H 2.029162 4.124895 -2.827183
C -5.650049 -0.419953 2.69599
C -6.987024 -0.807057 2.685837
C -4.680097 -1.308611 2.240788
H -7.747776 -0.112197 3.039607
C -7.374852 -2.068333 2.225942
C -5.042569 -2.572165 1.769764

H -4.278693 -3.258635 1.40851
C -6.37835 -2.944112 1.770173
H -6.6616 -3.931851 1.407576
H -5.351545 0.56533 3.045065
C -8.812515 -2.48563 2.225391
H -9.479312 -1.647112 2.448653
H -9.110896 -2.90468 1.257297
H -9.001197 -3.264261 2.975592
H -0.416646 -0.003398 -0.139026
H 0.662741 4.946762 1.64392
C -0.356657 3.147654 1.175922
C 0.807412 0.992873 1.810863
C 1.957002 1.793682 2.203178
C 1.887263 3.210876 2.123008
C 2.999991 3.955811 2.540602
C 4.143193 3.326068 3.010073
C 4.209291 1.928498 3.085309
C 3.119272 1.17321 2.690488
H -1.245516 3.646885 0.788712
H -1.260204 1.300722 1.66107
O 0.749228 -0.238765 2.037441
H 3.144528 0.085824 2.731322
H 5.113321 1.4441 3.446834
H 4.996779 3.924342 3.322393
H 2.957695 5.042685 2.489315
K -0.823593 -0.248623 4.443992
K -0.387335 -2.427251 0.795042

TS_A-B_ortho_2Kcation_acetonitrile
C -0.629168 1.074476 -0.266902
C -0.474154 1.98163 1.625647
C 0.922341 3.947388 1.335824
C -4.380596 0.743619 -0.27875
C -4.682303 1.660172 -1.259202
C -3.697688 2.351588 -1.966201
C -2.369284 2.174137 -1.699301
C -2.003225 1.270113 -0.665509
C -3.021019 0.526221 0.029034

C -5.687094 2.961395 -2.764293
H -5.169473 0.186264 0.216278
H -1.618867 2.679543 -2.295539
H -6.1154 2.583937 -3.700719
O -5.906467 1.99629 -1.72862
O -4.284662 3.141035 -2.913874
H -6.154797 3.910974 -2.4734
N -2.605201 -0.429736 0.956207
S -3.312456 -0.5655 2.36928
O -3.490565 0.718445 3.094644
O -2.482266 -1.555239 3.124999
C 1.93197 3.127304 -2.193262
C 2.971899 2.207761 -2.285216
C 0.743431 2.787965 -1.554098
H 3.899499 2.478397 -2.784863
H -0.031934 3.540762 -1.44584
C 2.823895 0.940413 -1.721536
C 0.562774 1.508515 -0.99899
H 3.636209 0.218578 -1.773788
C 1.640331 0.607153 -1.081481
H 1.53581 -0.377354 -0.618317
H 2.047621 4.124672 -2.61253
C -6.019851 -0.700177 2.882427
C -7.280012 -1.277122 2.755373
C -4.936177 -1.285853 2.234555
H -8.131135 -0.817741 3.25649
C -7.478142 -2.430645 1.991148
C -5.109219 -2.431378 1.455989
H -4.261303 -2.862917 0.925161
C -6.371078 -2.994956 1.340913
H -6.508665 -3.885867 0.729065
H -5.870623 0.20609 3.463876
C -8.832418 -3.05682 1.871286
H -9.624902 -2.379236 2.203672
H -9.05165 -3.354311 0.839617
H -8.903169 -3.965822 2.48266
H -0.483435 0.109337 0.213663
H 1.05075 5.00153 1.093103

C -0.315607 3.386199 1.383703
C 0.646598 1.205851 2.123483
C 1.966471 1.803047 2.005995
C 2.095415 3.160804 1.597277
C 3.387505 3.700623 1.467491
C 4.509325 2.932745 1.727902
C 4.377686 1.599193 2.143154
C 3.117677 1.048305 2.286521
H -1.203006 3.978474 1.161945
H -1.460478 1.611914 1.906079
O 0.476642 0.02834 2.575588
H 2.988391 0.015614 2.605965
H 5.264858 1.004132 2.349108
H 5.500124 3.368734 1.615912
H 3.495078 4.738284 1.153261
K -1.27507 0.470096 4.619377
K -0.495735 -2.199034 1.491576

InterB_ortho_2Kcation_acetonitrile
C -0.681146 0.858999 -0.477301
C -0.495873 2.372262 2.03973
C 1.052936 3.99419 1.125386
C -4.36863 1.088691 0.047062
C -4.670191 1.898457 -1.019311
C -3.719914 2.2981 -1.978357
C -2.415497 1.937907 -1.872719
C -2.020432 1.139655 -0.748811
C -3.034247 0.637632 0.183719
C -5.703167 3.131057 -2.56471
H -5.149189 0.786938 0.737019
H -1.694163 2.209359 -2.635305
H -6.344837 2.702488 -3.342798
O -5.880351 2.38117 -1.354441
O -4.341282 3.026659 -2.948438
H -5.959983 4.179793 -2.371969
N -2.613063 -0.233821 1.148545
S -3.432131 -0.407937 2.522499
O -3.763022 0.874987 3.183146

O -2.592904 -1.312514 3.353746
C 1.874091 3.033397 -2.207557
C 3.000804 2.223592 -2.091587
C 0.651781 2.617364 -1.69601
H 3.956712 2.558463 -2.48905
H -0.198407 3.292882 -1.728207
C 2.909843 0.989814 -1.439167
C 0.525657 1.360078 -1.06241
H 3.794518 0.369508 -1.315499
C 1.698298 0.580127 -0.919793
H 1.631561 -0.368268 -0.38262
H 1.952812 4.010985 -2.678733
C -6.114302 -0.865609 2.91188
C -7.30345 -1.551598 2.68906
C -4.969131 -1.25077 2.219067
H -8.202607 -1.250001 3.224625
C -7.370079 -2.616713 1.784967
C -5.009716 -2.302255 1.303238
H -4.116237 -2.571929 0.741553
C -6.205087 -2.974586 1.092473
H -6.242619 -3.789797 0.370845
H -6.070224 -0.026617 3.602265
C -8.647921 -3.364369 1.565982
H -9.518063 -2.78982 1.898324
H -8.792686 -3.621435 0.510955
H -8.651334 -4.309378 2.124678
H -0.523164 0.111962 0.295151
H 1.254881 4.953626 0.651019
C -0.233135 3.614433 1.441745
C 0.540603 1.467754 2.396179
C 1.895102 1.868839 2.048948
C 2.138875 3.121561 1.407832
C 3.46642 3.448149 1.051917
C 4.510118 2.581069 1.308975
C 4.266494 1.349646 1.940659
C 2.977483 1.008559 2.305311
H -1.069281 4.270626 1.198053
H -1.530204 2.068145 2.216754

O 0.305038 0.344652 2.985975
H 2.767934 0.066179 2.810063
H 5.093964 0.673895 2.149776
H 5.52522 2.853446 1.025096
H 3.654713 4.404185 0.562622
K -1.538564 1.012934 4.773827
K -0.395618 -1.91845 1.997149

InterA_pre-para_2Kcation_acetonitrile
C 12.632552 -16.907168 -1.018347
C 14.20741 -17.063252 -0.73849
C 10.533108 -19.617199 0.693672
C 10.357776 -19.022344 1.919535
C 10.913638 -17.78672 2.229882
C 11.669529 -17.085665 1.320401
C 11.836052 -17.637208 0.032665
C 11.287129 -18.91221 -0.281533
C 9.54136 -18.348771 3.838667
H 10.078786 -20.569172 0.436733
H 12.1062 -16.123826 1.58093
H 8.615036 -17.84899 3.51598
O 9.742803 -19.529959 3.06409
O 10.660746 -17.504242 3.576297
H 9.512311 -18.60467 4.900684
N 11.516915 -19.478933 -1.539746
S 10.210875 -19.601484 -2.48308
O 9.042915 -20.194358 -1.815927
O 10.702232 -20.257619 -3.720172
C 10.876976 -13.483144 -0.995172
C 11.602437 -12.796075 -1.960577
C 11.197881 -14.800388 -0.669955
H 11.351556 -11.767282 -2.211391
H 10.581986 -15.324699 0.055615
C 12.6397 -13.450708 -2.622985
C 12.260216 -15.459601 -1.294167
H 13.201078 -12.939961 -3.403155
C 12.956412 -14.762898 -2.293683
H 13.754231 -15.262055 -2.845884

H 10.042474 -12.998331 -0.490635
C 8.782949 -17.271675 -2.169274
C 8.476716 -15.948457 -2.469731
C 9.747451 -17.937679 -2.923455
H 7.716717 -15.42742 -1.88667
C 9.121216 -15.271206 -3.50996
C 10.41248 -17.279919 -3.95772
H 11.155381 -17.816907 -4.545167
C 10.095762 -15.958239 -4.244081
H 10.609449 -15.444802 -5.057114
H 8.264191 -17.803328 -1.373755
C 8.756058 -13.860706 -3.85537
H 9.615502 -13.304801 -4.246599
H 7.975065 -13.831871 -4.626986
H 8.368924 -13.316539 -2.986603
H 12.471428 -17.463534 -1.952652
H 14.699821 -16.453207 -1.507081
C 14.556589 -18.488488 -0.954716
C 14.754554 -19.353715 0.055656
C 14.678956 -18.911467 1.441709
C 14.765105 -17.460295 1.681598
C 14.58776 -16.5567 0.616726
C 14.671747 -15.185285 0.874484
C 14.926851 -14.722022 2.160436
C 15.102673 -15.620915 3.216617
C 15.020848 -16.982772 2.97446
H 14.540127 -18.8352 -1.989435
H 14.921112 -20.41864 -0.102655
O 14.553464 -19.726177 2.374194
H 15.200617 -17.705663 3.770176
H 15.320608 -15.251696 4.21631
H 14.99945 -13.651858 2.342048
H 14.528739 -14.477869 0.058596
K 12.781256 -21.511827 -2.88325
K 12.567843 -19.650217 4.029648

TS_A-B_para_2Kcation_acetonitrile

C 12.267273 -16.852093 -1.108616

C 14.493382 -17.026145 -0.781032
C 10.626639 -19.721715 0.684723
C 10.473166 -19.144467 1.919923
C 10.972247 -17.877282 2.225147
C 11.604981 -17.103403 1.291736
C 11.717418 -17.620224 -0.028882
C 11.282742 -18.964042 -0.318432
C 9.706639 -18.529482 3.881005
H 10.227094 -20.701407 0.442991
H 12.032522 -16.141239 1.559231
H 8.743357 -18.071324 3.610265
O 9.9225 -19.69113 3.071853
O 10.775329 -17.640394 3.587198
H 9.732404 -18.80726 4.938278
N 11.483038 -19.495044 -1.581201
S 10.161582 -19.687336 -2.493303
O 9.020888 -20.270894 -1.778104
O 10.644813 -20.365345 -3.719168
C 11.3506 -13.182236 -0.767
C 11.962563 -12.662186 -1.903937
C 11.433493 -14.540501 -0.478029
H 11.897702 -11.598645 -2.123998
H 10.891927 -14.928446 0.379741
C 12.636006 -13.520421 -2.775911
C 12.139409 -15.417234 -1.32213
H 13.093563 -13.13016 -3.682789
C 12.71253 -14.873626 -2.490654
H 13.226522 -15.543992 -3.18108
H 10.792103 -12.52704 -0.101357
C 8.909331 -17.285195 -2.068319
C 8.661348 -15.945063 -2.340696
C 9.693703 -18.030701 -2.949763
H 8.047389 -15.361609 -1.65417
C 9.185901 -15.326792 -3.481666
C 10.230427 -17.436245 -4.090599
H 10.830801 -18.034155 -4.773578
C 9.968548 -16.096225 -4.350754
H 10.37901 -15.631583 -5.247617

H 8.48767 -17.765708 -1.186715
C 8.897293 -13.887238 -3.774829
H 9.656065 -13.446813 -4.430886
H 7.92813 -13.77043 -4.277724
H 8.855464 -13.288552 -2.857616
H 12.349344 -17.429443 -2.028468
H 14.70201 -16.354615 -1.610929
C 14.631467 -18.421617 -0.976184
C 14.578646 -19.301073 0.072581
C 14.480919 -18.843014 1.432714
C 14.595939 -17.393244 1.651887
C 14.610297 -16.50083 0.549865
C 14.647097 -15.113957 0.796267
C 14.688065 -14.627617 2.090949
C 14.703782 -15.51315 3.179803
C 14.656191 -16.876896 2.955072
H 14.699321 -18.787136 -2.002861
H 14.598047 -20.381027 -0.074176
O 14.303657 -19.638571 2.389706
H 14.690351 -17.581029 3.786155
H 14.761395 -15.124262 4.194343
H 14.722847 -13.554152 2.265218
H 14.64379 -14.426385 -0.04977
K 12.94458 -21.31462 -2.97105
K 12.616473 -19.916363 4.227987

InterB_para_2Kcation_acetonitrile

C 12.2672701793 -16.8520925313 -1.1086140874
C 14.4933795457 -17.0261427352 -0.7810327855
C 10.6266374739 -19.7217152853 0.6847249931
C 10.4731658444 -19.1444680084 1.919925674
C 10.9722477655 -17.8772829535 2.2251496152
C 11.6049804372 -17.1034040852 1.2917391108
C 11.717416346 -17.6202236833 -0.0288791927
C 11.2827404704 -18.9640417175 -0.3184300628
C 9.706642518 -18.5294839639 3.8810091488
H 10.2270921028 -20.7014068795 0.442992465
H 12.0325222009 -16.1412401644 1.5592341351

H 8.7433600215 -18.0713260848 3.6102716905
O 9.9225010804 -19.691131251 3.0718558836
O 10.7753318591 -17.6403957214 3.5872012873
H 9.7324087735 -18.8072624336 4.9382823373
N 11.4830361801 -19.4950436481 -1.5811988492
S 10.1615809793 -19.6873360281 -2.4933011692
O 9.0208859926 -20.2708916001 -1.7781023192
O 10.6448120873 -20.3653468618 -3.7191658365
C 11.3506006369 -13.1822346149 -0.7669975275
C 11.9625577129 -12.6621860809 -1.9039394418
C 11.4334948421 -14.5404986161 -0.4780252401
H 11.8976951638 -11.5986453412 -2.1240014415
H 10.8919337264 -14.9284416306 0.3797489973
C 12.6359946096 -13.5204233689 -2.7759160025
C 12.1394058074 -15.4172333255 -1.3221283527
H 13.0935463095 -13.1301635627 -3.6827975122
C 12.7125199743 -14.873627435 -2.4906569153
H 13.2265087343 -15.543995464 -3.1810843699
H 10.7921088584 -12.5270370992 -0.1013518367
C 8.9093284118 -17.2851938771 -2.0683244848
C 8.6613467087 -15.945062739 -2.3407022459
C 9.6937041575 -18.0307012051 -2.9497643256
H 8.0473843572 -15.3616075887 -1.6541798346
C 9.1859071089 -15.3267911815 -3.4816696948
C 10.2304331226 -17.4362451663 -4.0905980155
H 10.8308103233 -18.0341564745 -4.7735743335
C 9.9685570106 -16.0962254842 -4.3507539628
H 10.3790235603 -15.6315838146 -5.2476149509
H 8.4876624954 -17.7657073724 -1.1867224811
C 8.8973033117 -13.8872367358 -3.7748326782
H 9.6560619692 -13.4468224836 -4.4309115356
H 7.9281274853 -13.7704222176 -4.2777010097
H 8.8555058087 -13.2885458678 -2.8576212887
H 12.3493402117 -17.4294421317 -2.0284659246
H 14.7020063656 -16.3546117983 -1.6109289293
C 14.6314653006 -18.4216140979 -0.9761853111
C 14.5786464548 -19.30107081 0.0725791115
C 14.4809195777 -18.8430134026 1.4327127917

C 14.5959390131 -17.3932437973 1.651886493
C 14.6102957054 -16.5008285365 0.5498652249
C 14.6470945685 -15.1139557461 0.7962673399
C 14.6880635005 -14.6276161346 2.0909499923
C 14.7037813919 -15.5131506385 3.1798037514
C 14.6561912266 -16.8768961698 2.9550719886
H 14.6993192598 -18.7871326134 -2.0028628223
H 14.5980490834 -20.381025322 -0.074178997
O 14.3036592635 -19.6385713559 2.3897037309
H 14.690351079 -17.5810295854 3.7861540405
H 14.7613939876 -15.1242630792 4.1943434478
H 14.7228444418 -13.5541520032 2.2652195858
H 14.6437872018 -14.4263832954 -0.0497689671
K 12.9445793346 -21.3146186897 -2.9710479383
K 12.6164739673 -19.9163804092 4.2279808375

TS_A-B_ortho_2Kcation_acetonitrile

C -0.629168 1.074476 -0.266902
C -0.474154 1.98163 1.625647
C 0.922341 3.947388 1.335824
C -4.380596 0.743619 -0.27875
C -4.682303 1.660172 -1.259202
C -3.697688 2.351588 -1.966201
C -2.369284 2.174137 -1.699301
C -2.003225 1.270113 -0.665509
C -3.021019 0.526221 0.029034
C -5.687094 2.961395 -2.764293
H -5.169473 0.186264 0.216278
H -1.618867 2.679543 -2.295539
H -6.1154 2.583937 -3.700719
O -5.906467 1.99629 -1.72862
O -4.284662 3.141035 -2.913874
H -6.154797 3.910974 -2.4734
N -2.605201 -0.429736 0.956207
S -3.312456 -0.5655 2.36928
O -3.490565 0.718445 3.094644
O -2.482266 -1.555239 3.124999
C 1.93197 3.127304 -2.193262

C 2.971899 2.207761 -2.285216
C 0.743431 2.787965 -1.554098
H 3.899499 2.478397 -2.784863
H -0.031934 3.540762 -1.44584
C 2.823895 0.940413 -1.721536
C 0.562774 1.508515 -0.99899
H 3.636209 0.218578 -1.773788
C 1.640331 0.607153 -1.081481
H 1.53581 -0.377354 -0.618317
H 2.047621 4.124672 -2.61253
C -6.019851 -0.700177 2.882427
C -7.280012 -1.277122 2.755373
C -4.936177 -1.285853 2.234555
H -8.131135 -0.817741 3.25649
C -7.478142 -2.430645 1.991148
C -5.109219 -2.431378 1.455989
H -4.261303 -2.862917 0.925161
C -6.371078 -2.994956 1.340913
H -6.508665 -3.885867 0.729065
H -5.870623 0.20609 3.463876
C -8.832418 -3.05682 1.871286
H -9.624902 -2.379236 2.203672
H -9.05165 -3.354311 0.839617
H -8.903169 -3.965822 2.48266
H -0.483435 0.109337 0.213663
H 1.05075 5.00153 1.093103
C -0.315607 3.386199 1.383703
C 0.646598 1.205851 2.123483
C 1.966471 1.803047 2.005995
C 2.095415 3.160804 1.597277
C 3.387505 3.700623 1.467491
C 4.509325 2.932745 1.727902
C 4.377686 1.599193 2.143154
C 3.117677 1.048305 2.286521
H -1.203006 3.978474 1.161945
H -1.460478 1.611914 1.906079
O 0.476642 0.02834 2.575588
H 2.988391 0.015614 2.605965

H 5.264858 1.004132 2.349108
H 5.500124 3.368734 1.615912
H 3.495078 4.738284 1.153261
K -1.27507 0.470096 4.619377
K -0.495735 -2.199034 1.491576

TS_A-B_ortho_2Kcation_acetonitrile_conf8
C -1.526345 1.328245 -0.253308
C -0.859197 1.750393 1.658744
C 1.009794 3.315176 1.384085
C -1.860289 -2.398646 -0.772948
C -3.226259 -2.492839 -0.694563
C -4.053028 -1.397538 -0.406172
C -3.537339 -0.148438 -0.226788
C -2.127395 0.029684 -0.314349
C -1.259601 -1.109902 -0.567169
C -5.348415 -3.148221 -0.8446
H -1.258716 -3.245416 -1.093624
H -4.196142 0.700679 -0.081277
H -5.739324 -3.186319 -1.871491
O -3.988508 -3.604369 -0.852387
O -5.358403 -1.812992 -0.365948
H -5.935599 -3.784473 -0.175518
N 0.071114 -0.865744 -0.57164
S 1.115747 -2.092116 -0.770756
O 0.873106 -2.935563 -1.946927
O 1.26386 -2.845685 0.512949
C -4.056489 4.178049 -0.141836
C -3.612072 4.952464 -1.212173
C -3.401488 2.996577 0.187315
H -4.126621 5.875722 -1.46925
H -3.739586 2.42301 1.050469
C -2.497933 4.543805 -1.940012
C -2.288616 2.561724 -0.548125
H -2.136019 5.14623 -2.770577
C -1.836576 3.367966 -1.601522
H -0.958259 3.055895 -2.166008
H -4.914887 4.499666 0.444586

C 3.817645 -1.937758 -0.840211
C 5.038821 -1.317692 -1.071102
C 2.638055 -1.214606 -1.016746
H 5.961576 -1.880158 -0.929953
C 5.105817 0.018558 -1.483246
C 2.676234 0.113475 -1.432567
H 1.747604 0.664392 -1.557133
C 3.907105 0.717923 -1.658202
H 3.940055 1.762607 -1.968114
H 3.773753 -2.975114 -0.513905
C 6.424079 0.679541 -1.744518
H 7.228498 0.237909 -1.145993
H 6.718332 0.576321 -2.79725
H 6.389371 1.75398 -1.530082
H -0.522448 1.337734 -0.679602
H 1.385286 4.321694 1.206991
C -0.325613 3.081819 1.49363
C 0.053645 0.703741 2.059504
C 1.481568 0.933131 1.825272
C 1.952149 2.244662 1.525524
C 3.344103 2.459881 1.446602
C 4.241376 1.429157 1.656394
C 3.77628 0.134724 1.945123
C 2.415969 -0.102607 2.031631
H -1.030459 3.904863 1.388629
H -1.856019 1.661711 2.09407
O -0.335229 -0.318859 2.71681
H 2.040354 -1.114756 2.185763
H 4.482038 -0.686811 2.051532
H 5.311472 1.615838 1.581324
H 3.70554 3.462035 1.217
K -0.852101 -2.784266 2.035204
K 1.22108 0.643443 4.779386

TS_A-B_ortho_2Kcation_acetonitrile_conf9

C -1.3077120453 1.4589362892 -0.0144325562
C -0.5162327122 2.2934169067 1.735366448
C 1.5087849476 3.2649223905 0.8447617878

C 1.0518635033 2.7767744572 -2.6296176511
C 1.8083351849 1.6557235026 -2.8451971951
C 1.5236665557 0.4322611006 -2.234269725
C 0.4933675326 0.2982046178 -1.3510505356
C -0.2888565363 1.441912094 -1.0350945161
C -0.0365169097 2.6905268723 -1.719490979
C 3.362993715 0.187721614 -3.4478594502
H 1.2396675608 3.692718445 -3.1799229414
H 0.2947824836 -0.6686332123 -0.9063446497
H 3.5330198066 -0.3090915996 -4.4080796573
O 2.8690887922 1.5114564539 -3.6847254895
O 2.3882957451 -0.5228465736 -2.7073039236
H 4.2983807849 0.2585614355 -2.8683989875
N -0.8962195778 3.7225778804 -1.4531127234
S -0.5518791954 5.2080928962 -1.864190056
O -0.5631958814 5.490877412 -3.3334045618
O 0.685046528 5.7733222542 -1.2355368952
C -2.3839780924 -2.0503679423 1.073727247
C -3.7691682107 -1.8848486829 1.1299758173
C -1.5512151353 -0.9816135024 0.7458719238
H -4.417151923 -2.7305380962 1.3534054398
H -0.4760833611 -1.1320647924 0.7664047832
C -4.3133638533 -0.6231453727 0.8739115448
C -2.0817111368 0.2849416037 0.4257415495
H -5.3919128162 -0.4755576098 0.901356768
C -3.4788395535 0.4402191136 0.5375893733
H -3.914053468 1.4125234201 0.3051846841
H -1.9430855404 -3.0246014888 1.2793516298
C -1.7455959684 6.9052742497 -0.0658733809
C -2.8363329905 7.5916910038 0.4622526844
C -1.9345792833 6.0679909819 -1.160018738
H -2.6935508616 8.2478931175 1.3204617018
C -4.1125098582 7.4573597923 -0.090568112
C -3.1978680498 5.9149079468 -1.7320470103
H -3.3261745256 5.261264992 -2.5923053853
C -4.2735228966 6.6060662127 -1.1933508376
H -5.2619738145 6.4911684556 -1.6378331929
H -0.7508008127 7.0107186979 0.3621166651

C -5.2794406973 8.216536565 0.4621361727
H -5.0503821918 8.6639663249 1.4346908907
H -5.5779902096 9.0319410206 -0.2094492324
H -6.1600674712 7.5750189458 0.5842053751
H -1.9474664075 2.3340933786 -0.129474103
H 2.0468549157 4.0977097282 0.3953515368
C 0.2290592068 3.4289459018 1.276380313
C 0.2059675628 1.1279076956 2.2251057009
C 1.5366554251 0.929004472 1.6614794596
C 2.1602711656 1.9895835197 0.9470293979
C 3.4203503384 1.7493196366 0.3690882536
C 4.0323672363 0.511363242 0.4834202887
C 3.4150148992 -0.5270869402 1.1940054857
C 2.1837309801 -0.3077853162 1.7888182574
H -0.2723985388 4.3868167145 1.1608557368
H -1.4754998822 2.4784164861 2.2273538932
O -0.3267537638 0.2957383245 3.0011713252
H 1.683302367 -1.0914782195 2.3562588475
H 3.9031907527 -1.4953905786 1.2788477627
H 5.0068802901 0.3457906474 0.0258806588
H 3.9101145537 2.555751279 -0.1779013533
K 1.6807201971 6.8812518563 -3.4562283585
K -2.6958498584 -0.2556310211 3.6973457941

TS_A-B_ortho_2Kcation_acetonitrile_conf10
C 12.478656 -14.768619 -1.139634
C 13.31565 -16.374161 -2.138534
C 12.842863 -16.012912 -4.51796
C 10.325429 -15.943785 1.734505
C 11.177263 -15.636907 2.76425
C 12.466502 -15.123409 2.563019
C 12.935104 -14.856527 1.311117
C 12.097629 -15.130788 0.192544
C 10.776934 -15.707606 0.390764
C 12.068878 -15.163535 4.748135
H 9.29988 -16.247537 1.928639
H 13.903553 -14.38714 1.177418
H 11.741467 -14.203424 5.172164

O 10.952512 -15.783177 4.094736
O 13.080073 -14.950271 3.776403
H 12.442976 -15.83224 5.529214
N 10.068948 -15.996899 -0.72528
S 8.628432 -16.734713 -0.587969
O 7.668191 -16.069297 0.300321
O 8.828606 -18.190012 -0.305777
C 15.750047 -12.819583 -1.314107
C 15.341211 -11.701289 -2.038604
C 14.840011 -13.823966 -1.004262
H 16.053998 -10.915689 -2.279529
H 15.185726 -14.708554 -0.469169
C 14.018936 -11.603534 -2.463073
C 13.5 -13.732293 -1.410144
H 13.69153 -10.739811 -3.038013
C 13.112779 -12.615307 -2.162728
H 12.081277 -12.541448 -2.506152
H 16.785871 -12.91327 -0.994028
C 6.984244 -17.492871 -2.598548
C 6.424795 -17.412962 -3.867251
C 8.018422 -16.624205 -2.249994
H 5.618914 -18.093107 -4.142522
C 6.877134 -16.471995 -4.79978
C 8.482666 -15.674577 -3.155912
H 9.293797 -15.010012 -2.870001
C 7.911669 -15.609702 -4.42136
H 8.284152 -14.877572 -5.138094
H 6.631521 -18.230988 -1.880702
C 6.261799 -16.378972 -6.162037
H 5.841992 -17.337243 -6.487281
H 5.440425 -15.650541 -6.177809
H 6.988493 -16.051561 -6.914612
H 11.622402 -14.709318 -1.812756
H 13.061485 -15.52002 -5.463869
C 13.619422 -15.790824 -3.423753
C 12.377973 -17.473214 -2.100213
C 11.47763 -17.628127 -3.245204
C 11.737719 -16.923263 -4.456528

C 10.936096 -17.20078 -5.583544
C 9.9212 -18.137925 -5.527587
C 9.663318 -18.830259 -4.331969
C 10.435643 -18.577615 -3.212199
H 14.460604 -15.102462 -3.486003
H 14.136152 -16.465943 -1.425023
O 12.416239 -18.362203 -1.185036
H 10.202753 -19.055118 -2.25986
H 8.828469 -19.525974 -4.274539
H 9.309212 -18.330087 -6.40742
H 11.132635 -16.661475 -6.509931
K 11.019335 -18.77035 0.980905
K 13.105948 -20.125313 -3.19212

TS_A-B_ortho_2Kcation_acetonitrile_conf11
C 12.545597 -14.973026 -1.440478
C 13.445786 -16.289465 -2.797756
C 12.18539 -15.747368 -4.786833
C 10.337105 -12.931968 -3.698286
C 9.125322 -13.504903 -3.417124
C 8.971889 -14.48616 -2.434802
C 10.036288 -14.959053 -1.725454
C 11.331172 -14.452113 -2.018212
C 11.479522 -13.38871 -2.986749
C 7.005059 -14.167337 -3.406712
H 10.418909 -12.11208 -4.404205
H 9.877284 -15.710364 -0.962231
H 6.103123 -13.652653 -3.061398
O 7.913089 -13.205617 -3.957387
O 7.641883 -14.802494 -2.313869
H 6.763128 -14.909876 -4.185284
N 12.736768 -12.869347 -3.141462
S 13.106403 -11.960544 -4.379249
O 12.491242 -10.596959 -4.384787
O 12.942414 -12.610072 -5.719274
C 11.909365 -17.153026 1.641605
C 12.910758 -16.684787 2.494461
C 11.783742 -16.65162 0.346991

H 12.983398 -17.055439 3.515428
H 11.02975 -17.078312 -0.307786
C 13.804336 -15.717326 2.026326
C 12.637296 -15.634387 -0.12699
H 14.584953 -15.330419 2.67958
C 13.670317 -15.212165 0.735129
H 14.350081 -14.432959 0.389403
H 11.210416 -17.912872 1.987908
C 15.759246 -12.330348 -4.983256
C 17.122358 -12.136737 -4.772279
C 14.846884 -11.745574 -4.111807
H 17.841078 -12.594553 -5.451485
C 17.586862 -11.363191 -3.705543
C 15.282714 -10.96755 -3.03869
H 14.555078 -10.511807 -2.370409
C 16.644093 -10.785337 -2.842966
H 16.98915 -10.17731 -2.006881
H 15.396067 -12.932115 -5.813842
C 19.052266 -11.136266 -3.494184
H 19.660924 -11.80637 -4.109809
H 19.337321 -10.107969 -3.751693
H 19.339304 -11.28614 -2.446706
H 13.368921 -14.274418 -1.590348
H 12.04514 -15.163024 -5.694554
C 13.330847 -15.624579 -4.06277
C 12.620797 -17.461157 -2.541091
C 11.3509 -17.504604 -3.257591
C 11.133214 -16.622362 -4.352296
C 9.877413 -16.648703 -4.986204
C 8.876517 -17.501566 -4.549035
C 9.100043 -18.372837 -3.474148
C 10.33559 -18.379916 -2.848432
H 14.108521 -14.931835 -4.375195
H 14.429102 -16.306611 -2.319037
O 12.937038 -18.319951 -1.680255
H 10.543985 -19.052209 -2.017
H 8.308489 -19.039201 -3.138696
H 7.909888 -17.502731 -5.051164

H 9.699619 -15.979144 -5.828546
K 11.710234 -10.561025 -6.911978
K 14.52261 -18.395883 0.289599

TS_A-B_ortho_2Kcation_acetonitrile_conf12
C 12.303448 -14.576871 -1.235801
C 13.517791 -16.111843 -2.254805
C 12.869754 -15.993157 -4.591297
C 15.194024 -12.63369 0.210973
C 15.095633 -11.48841 -0.547213
C 14.110309 -11.314851 -1.51604
C 13.174762 -12.281202 -1.763931
C 13.23896 -13.499365 -1.035685
C 14.249263 -13.668147 -0.023729
C 15.247245 -9.41341 -1.337061
H 15.856111 -12.685911 1.070097
H 12.37508 -12.095703 -2.470757
H 14.784213 -8.638799 -0.708448
O 15.880811 -10.380571 -0.48339
O 14.257039 -10.082598 -2.099404
H 15.997993 -8.980142 -2.00565
N 14.203389 -14.826746 0.723112
S 15.465524 -15.484534 1.364413
O 16.431238 -14.637412 2.122565
O 16.24934 -16.310926 0.354308
C 9.726555 -13.994482 -3.966698
C 8.638391 -14.669732 -3.423248
C 10.93243 -13.929531 -3.276453
H 7.697554 -14.717819 -3.967705
H 11.783328 -13.449036 -3.749388
C 8.762725 -15.29523 -2.181764
C 11.074523 -14.540607 -2.015423
H 7.920459 -15.835467 -1.75453
C 9.965436 -15.239907 -1.496491
H 10.071853 -15.749279 -0.538117
H 9.643098 -13.523439 -4.944264
C 15.484911 -17.019055 3.616015
C 14.971122 -17.975299 4.484473

C 14.737364 -16.634377 2.50297
H 15.548112 -18.265547 5.361759
C 13.718136 -18.560408 4.263539
C 13.481989 -17.196823 2.258397
H 12.905537 -16.888902 1.386996
C 12.990434 -18.158557 3.135393
H 12.008132 -18.592619 2.950201
H 16.447657 -16.549863 3.806576
C 13.155079 -19.557023 5.227822
H 13.940286 -20.149462 5.709107
H 12.601094 -19.052571 6.030175
H 12.454829 -20.244934 4.742757
H 12.254848 -15.255178 -0.383117
H 12.972897 -15.56744 -5.588931
C 13.697046 -15.609562 -3.578194
C 12.621039 -17.22432 -2.011373
C 11.709334 -17.58256 -3.093513
C 11.836482 -16.965341 -4.367581
C 10.931355 -17.327906 -5.381066
C 9.935663 -18.260809 -5.144556
C 9.817636 -18.869971 -3.887555
C 10.701956 -18.534448 -2.877487
H 14.460944 -14.850968 -3.753427
H 14.298698 -15.958906 -1.508161
O 12.597386 -17.821447 -0.898326
H 10.627727 -18.981271 -1.887713
H 9.029677 -19.598574 -3.708284
H 9.241853 -18.523552 -5.941029
H 11.019846 -16.857376 -6.36017
K 18.141903 -14.318949 0.089421
K 14.919979 -18.616961 -0.127012

TS_A-B_ortho_2Kcation_acetonitrile_conf13
C 11.496009 -15.406472 -1.878568
C 13.240844 -16.579803 -1.907532
C 14.175062 -16.36509 -4.139035
C 12.313712 -13.527178 1.280112
C 12.538351 -12.279402 0.745907

C 12.374949 -12.006441 -0.612786
C 12.008982 -12.980892 -1.498285
C 11.806875 -14.298515 -1.005991
C 11.930468 -14.563653 0.403095
C 12.961846 -10.11666 0.412251
H 12.403482 -13.693472 2.348946
H 11.836542 -12.73736 -2.539928
H 12.261613 -9.313976 0.673213
O 12.880634 -11.147243 1.403969
O 12.603286 -10.67954 -0.843194
H 13.992359 -9.740447 0.370727
N 11.614638 -15.847551 0.849433
S 12.528138 -16.590073 1.912228
O 13.982436 -16.546507 1.613341
O 11.988552 -17.984519 1.983934
C 11.222527 -14.541987 -5.574736
C 10.171767 -15.373495 -5.9491
C 11.670171 -14.518749 -4.257407
H 9.826824 -15.389032 -6.980676
H 12.527915 -13.903521 -4.001639
C 9.570355 -16.196564 -4.996936
C 11.064971 -15.323785 -3.275812
H 8.757382 -16.861205 -5.281442
C 10.021863 -16.174479 -3.686564
H 9.566596 -16.838639 -2.947474
H 11.709341 -13.911558 -6.316009
C 13.452303 -15.669821 4.342508
C 13.276875 -15.142084 5.618294
C 12.336068 -15.903702 3.54489
H 14.148936 -14.952098 6.242753
C 12.004203 -14.842757 6.112958
C 11.055262 -15.601405 4.010087
H 10.191928 -15.751532 3.362518
C 10.897365 -15.077061 5.284321
H 9.898868 -14.83401 5.64623
H 14.444166 -15.885642 3.953498
C 11.819427 -14.290137 7.491737
H 12.76472 -13.945468 7.922037

H 11.117111 -13.448825 7.500252
H 11.409059 -15.049013 8.170447
H 10.994595 -16.210122 -1.343157
H 14.802264 -15.852911 -4.867676
C 14.128129 -15.952907 -2.84383
C 12.620022 -17.848119 -2.242878
C 12.629415 -18.236688 -3.64353
C 13.390926 -17.484076 -4.582109
C 13.357762 -17.870639 -5.93378
C 12.60299 -18.955404 -6.345728
C 11.864571 -19.702328 -5.415699
C 11.887708 -19.346996 -4.079699
H 14.704959 -15.089025 -2.514597
H 13.401706 -16.404192 -0.843613
O 12.035655 -18.545279 -1.353191
H 11.322756 -19.909249 -3.338143
H 11.276665 -20.555359 -5.747818
H 12.587348 -19.234261 -7.397531
H 13.935848 -17.298717 -6.658911
K 9.97784 -18.049421 0.261796
K 14.068671 -18.970239 0.421833

TS_A-B_ortho_2Kcation_acetonitrile_conf14
C 11.670667 -15.375224 -2.036144
C 13.61527 -16.419066 -1.777687
C 14.788455 -16.196231 -3.899627
C 9.147374 -17.825948 -0.678152
C 9.068497 -17.340877 0.603533
C 9.788341 -16.224547 1.039257
C 10.622272 -15.555998 0.196096
C 10.771406 -16.021333 -1.143102
C 10.005734 -17.165055 -1.609262
C 8.466577 -16.888463 2.701172
H 8.504576 -18.647081 -0.980319
H 11.141075 -14.665245 0.530499
H 7.518081 -16.348788 2.829934
O 8.319086 -17.828036 1.623799
O 9.501846 -15.983141 2.355163

H 8.737645 -17.430368 3.612973
N 10.100543 -17.503372 -2.937617
S 9.37067 -18.841669 -3.474865
O 9.646209 -20.052902 -2.660067
O 9.729996 -18.945947 -4.902357
C 13.266804 -12.148344 -0.792208
C 13.082569 -11.330676 -1.906048
C 12.815837 -13.462544 -0.800879
H 13.438188 -10.302951 -1.895453
H 13.024109 -14.097172 0.058702
C 12.458537 -11.845952 -3.03856
C 12.162038 -13.993916 -1.925243
H 12.320505 -11.222295 -3.919311
C 12.017596 -13.164215 -3.047984
H 11.529697 -13.563283 -3.939012
H 13.778655 -11.763315 0.087179
C 6.770003 -19.711676 -3.335953
C 5.393335 -19.530752 -3.335261
C 7.605368 -18.595845 -3.395299
H 4.739205 -20.400823 -3.288676
C 4.828231 -18.249858 -3.39047
C 7.069364 -17.311489 -3.448437
H 7.730921 -16.447081 -3.4717
C 5.688522 -17.148216 -3.440707
H 5.267153 -16.144083 -3.470344
H 7.202205 -20.70867 -3.285821
C 3.341949 -18.071891 -3.405249
H 2.851949 -18.693044 -2.646832
H 3.054033 -17.031115 -3.227187
H 2.918284 -18.367098 -4.373782
H 11.542003 -15.689213 -3.070788
H 15.451168 -15.646211 -4.566167
C 14.496066 -15.720148 -2.654618
C 13.187299 -17.761341 -2.08682
C 13.440733 -18.225604 -3.462045
C 14.263713 -17.45516 -4.337444
C 14.523873 -17.958055 -5.633828
C 13.984596 -19.162317 -6.056411

C 13.161224 -19.90874 -5.195838
C 12.913766 -19.445415 -3.91437
H 14.909775 -14.767122 -2.32736
H 13.607553 -16.158153 -0.720738
O 12.605319 -18.478429 -1.239092
H 12.257867 -20.000974 -3.247124
H 12.716884 -20.841684 -5.534952
H 14.206494 -19.535366 -7.054783
H 15.1755 -17.38727 -6.29594
K 11.40004 -17.090611 -5.547483
K 11.047459 -20.321213 -0.456722

TS_A-B_ortho_2Kcation_acetonitrile_conf15
C 12.191082 -15.364629 -1.879728
C 13.473169 -16.987403 -1.968795
C 14.202782 -16.852836 -4.267865
C 12.535082 -14.056431 -5.385665
C 11.421634 -14.623411 -5.949178
C 10.459358 -15.29753 -5.192855
C 10.617344 -15.498692 -3.8533
C 11.788265 -14.998566 -3.219239
C 12.719592 -14.195469 -3.984399
C 9.879933 -15.430534 -7.326104
H 13.249389 -13.516626 -5.998767
H 9.868286 -16.063154 -3.314185
H 9.099173 -14.920481 -7.898093
O 11.034188 -14.591178 -7.250516
O 9.423413 -15.675054 -6.004948
H 10.165023 -16.38352 -7.801839
N 13.716827 -13.545797 -3.28373
S 15.104555 -13.134651 -3.999957
O 15.68551 -14.141261 -4.89112
O 15.947855 -12.636647 -2.883588
C 9.33211 -16.592086 0.363771
C 9.739401 -16.082574 1.59796
C 10.112757 -16.416606 -0.777944
H 9.109382 -16.194307 2.478518
H 9.783633 -16.880401 -1.700332

C 10.961154 -15.408409 1.679115
C 11.313843 -15.678792 -0.738147
H 11.301169 -15.00223 2.630755
C 11.726833 -15.222066 0.531827
H 12.675252 -14.685075 0.60422
H 8.388759 -17.130017 0.282228
C 15.801037 -11.256192 -5.88134
C 15.626255 -10.086641 -6.607656
C 14.803147 -11.675148 -4.999702
H 16.405685 -9.763723 -7.297371
C 14.463047 -9.314727 -6.478809
C 13.635884 -10.927589 -4.86085
H 12.83313 -11.29321 -4.218728
C 13.471653 -9.757784 -5.599391
H 12.548927 -9.185381 -5.504876
H 16.701173 -11.856319 -5.998148
C 14.287377 -8.061803 -7.279832
H 14.28836 -8.272315 -8.356444
H 13.346239 -7.555313 -7.043279
H 15.103779 -7.351627 -7.100398
H 13.025673 -14.741331 -1.557569
H 14.877066 -16.467196 -5.02923
C 14.442875 -16.603125 -2.95326
C 12.504397 -18.031363 -2.287569
C 12.218201 -18.225803 -3.703225
C 13.050267 -17.607441 -4.676239
C 12.717322 -17.762422 -6.033053
C 11.604196 -18.498749 -6.411298
C 10.793486 -19.113898 -5.447668
C 11.110452 -18.984405 -4.105849
H 15.303592 -16.005911 -2.658928
H 13.772396 -16.953346 -0.916658
O 11.889001 -18.654095 -1.385523
H 10.500064 -19.451974 -3.334775
H 9.9232 -19.68983 -5.754438
H 11.368432 -18.614618 -7.468551
H 13.348482 -17.292105 -6.787023
K 14.152551 -11.665454 -1.287763

K 11.708664 -18.603241 1.127682

TS_A-B_ortho_2Kcation_acetonitrile_conf16
C -1.541111 -0.222346 0.91149
C -1.658524 -0.235458 -1.189158
C -3.739943 0.872645 -1.771054
C 1.951906 1.168736 1.124901
C 1.666384 2.410459 1.643731
C 0.370451 2.798665 1.985827
C -0.697973 1.968503 1.794183
C -0.464604 0.68949 1.220194
C 0.879454 0.276974 0.913015
C 1.755236 4.470056 2.491178
H 2.977318 0.881278 0.915204
H -1.688407 2.267995 2.115733
H 2.105999 4.693216 3.506022
O 2.538449 3.398577 1.951987
O 0.397407 4.050295 2.532193
H 1.853728 5.348204 1.839798
N 1.067103 -1.027996 0.455648
S 2.033411 -1.336375 -0.763381
O 1.835133 -0.459236 -1.945521
O 1.821726 -2.786013 -1.070465
C -5.029723 1.038891 1.76048
C -5.620672 -0.139418 2.205528
C -3.702282 1.049673 1.343298
H -6.659233 -0.142753 2.529193
H -3.280316 1.968761 0.947146
C -4.878029 -1.320052 2.222188
C -2.927435 -0.123542 1.373378
H -5.33426 -2.2504 2.553743
C -3.557164 -1.306866 1.801801
H -2.984333 -2.237656 1.791771
H -5.608633 1.959508 1.725476
C 4.636129 -0.508671 -1.14732
C 5.971503 -0.396601 -0.771239
C 3.747502 -1.172994 -0.307268
H 6.668384 0.127987 -1.423699

C 6.436216 -0.936277 0.431423
C 4.183718 -1.711172 0.904668
H 3.471942 -2.196039 1.572102
C 5.517658 -1.591893 1.264337
H 5.858211 -2.005639 2.212964
H 4.27335 -0.07281 -2.074743
C 7.875329 -0.822842 0.827171
H 8.423907 -0.136871 0.174546
H 7.984131 -0.467036 1.858272
H 8.378576 -1.796899 0.7767
H -1.196755 -1.253786 0.871826
H -4.315653 1.770667 -1.991831
C -2.39858 0.936809 -1.556333
C -2.263626 -1.545917 -1.340553
C -3.707146 -1.590349 -1.505527
C -4.434858 -0.38263 -1.703281
C -5.833001 -0.457799 -1.833915
C -6.489683 -1.674648 -1.769663
C -5.767838 -2.863543 -1.586624
C -4.391396 -2.816515 -1.464534
H -1.876716 1.893034 -1.583538
H -0.571664 -0.2078 -1.270838
O -1.560759 -2.603681 -1.25971
H -3.807855 -3.724241 -1.32039
H -6.29194 -3.815773 -1.54012
H -7.572937 -1.709603 -1.86777
H -6.39763 0.461864 -1.983923
K -0.008304 -3.609567 0.655766
K 0.234497 -2.185103 -3.277344

TS_A-B_ortho_2Kcation_acetonitrile_conf17
C -1.669317 -0.071463 0.809771
C -1.470314 0.088595 -1.39426
C -3.421308 1.32833 -2.160455
C -0.968572 -3.71043 1.502664
C 0.372044 -3.606333 1.778041
C 1.056362 -2.387223 1.774635
C 0.412198 -1.223147 1.485961

C -0.976337 -1.261721 1.166051
C -1.699165 -2.52198 1.195827
C 2.46887 -3.972595 2.437926
H -1.461661 -4.672901 1.598191
H 0.941606 -0.278521 1.528586
H 2.624167 -4.076398 3.520857
O 1.229863 -4.610649 2.087459
O 2.371046 -2.602254 2.08662
H 3.283436 -4.441588 1.876743
N -3.057779 -2.487837 0.993514
S -3.886113 -3.872183 0.896119
O -3.300606 -4.864338 -0.041754
O -5.281386 -3.482858 0.614416
C 0.252023 3.194467 1.200302
C -0.706026 3.974102 1.846478
C -0.026942 1.87755 0.856169
H -0.483994 5.00489 2.112849
H 0.716403 1.309279 0.29989
C -1.95528 3.429377 2.129618
C -1.273409 1.301988 1.154701
H -2.716384 4.031272 2.621732
C -2.234907 2.114773 1.774976
H -3.216546 1.69318 1.998364
H 1.221352 3.61993 0.949448
C -4.071327 -6.033657 2.579452
C -4.11908 -6.642151 3.826971
C -3.873437 -4.655266 2.499218
H -4.272366 -7.718879 3.891714
C -3.970419 -5.898678 5.004729
C -3.720191 -3.893009 3.655039
H -3.542286 -2.821502 3.57968
C -3.764555 -4.519199 4.895418
H -3.631973 -3.926848 5.800041
H -4.179912 -6.61842 1.668686
C -4.04475 -6.564156 6.343557
H -3.534005 -7.53339 6.344437
H -3.601874 -5.946409 7.131216
H -5.086753 -6.756917 6.630354

H -2.750437 -0.199325 0.7903
H -3.922855 2.269252 -2.381993
C -2.133933 1.311322 -1.708353
C -2.055768 -1.177294 -1.762792
C -3.471144 -1.146042 -2.171157
C -4.124398 0.10279 -2.395861
C -5.469909 0.090312 -2.832806
C -6.149053 -1.101644 -3.02647
C -5.505406 -2.328467 -2.788681
C -4.180982 -2.338121 -2.383797
H -1.604557 2.248897 -1.543373
H -0.386564 0.08921 -1.290668
O -1.402759 -2.246002 -1.707576
H -3.677989 -3.27955 -2.173183
H -6.044536 -3.264206 -2.917378
H -7.181121 -1.087926 -3.3726
H -5.964235 1.041232 -3.033261
K -5.4449 -0.93433 0.245311
K -1.096885 -4.753175 -1.466948

TS_A-B_ortho_2Kcation_acetonitrile_conf18
C -1.4134253783 0.2537588991 0.4506271047
C -1.5336581323 -0.3568675796 -1.4886059483
C -3.7059480601 0.5363422302 -2.1217429759
C -4.6970484526 2.0549215533 0.8371215817
C -5.4068613111 1.0751389094 1.4812738825
C -4.8193074579 -0.1104455487 1.9302395818
C -3.5074825127 -0.3916627204 1.6855407393
C -2.7341397704 0.5526857479 0.9568343957
C -3.3130834113 1.8315625114 0.6031584809
C -6.9839814237 -0.1793951819 2.4236604921
H -5.1820918571 2.9762685529 0.5319375468
H -3.0778831122 -1.3228295044 2.034663524
H -7.4778970818 -0.0355746794 3.3906761031
O -6.718665063 1.0949034641 1.8363294929
O -5.7490603476 -0.8524238315 2.6117210585
H -7.624096639 -0.7608080129 1.740102229
N -2.4574123799 2.7894973648 0.1059911603

S -3.006590988 4.0319171968 -0.7714592544
O -3.9639918175 3.6799631807 -1.824857835
O -1.7704609127 4.7442407942 -1.181735838
C 0.2987912821 -2.7457432907 2.0907734431
C 1.5865112693 -2.2427377701 2.252373624
C -0.6958375112 -1.9762831905 1.4967800956
H 2.3591674474 -2.84963374 2.7192017599
H -1.674269649 -2.4188422866 1.3374618783
C 1.8807423848 -0.9602295155 1.7952845001
C -0.4344008634 -0.6656825361 1.0642295552
H 2.8870595813 -0.5586332773 1.8972826568
C 0.8830406869 -0.1964770411 1.2054745723
H 1.1215546127 0.8050204028 0.8364884282
H 0.0647454051 -3.7548206548 2.4245284529
C -4.7819339864 6.0553071623 -0.2144036386
C -5.3992917151 6.9837534807 0.6132216878
C -3.8556547265 5.1636183715 0.3262186166
H -6.1242500835 7.6789788829 0.1905203066
C -5.1150705421 7.0403658631 1.9839862692
C -3.5626877623 5.1963806443 1.687210076
H -2.8778773745 4.4609594549 2.1111206009
C -4.1930211837 6.128234606 2.5059843892
H -3.9758847373 6.1424521176 3.5738583123
H -5.0182863617 6.0040816932 -1.2750480609
C -5.7933431746 8.0458595605 2.862482105
H -6.884737089 7.945189063 2.8202782039
H -5.4903208718 7.9423058025 3.9092269992
H -5.5622331167 9.0717008218 2.5496496841
H -0.923521308 1.1700366192 0.120535126
H -4.3230729465 1.3506497822 -2.4941707028
C -2.349892587 0.6456550291 -2.1235250158
C -2.0952131514 -1.6884139181 -1.3323628357
C -3.5510687261 -1.7467902731 -1.1849866634
C -4.3392730154 -0.6256362719 -1.5686832515
C -5.7347546928 -0.7033449412 -1.3892713666
C -6.3239035794 -1.8294194767 -0.8388708973
C -5.5421770756 -2.9277295367 -0.4460567575
C -4.1701251821 -2.8808400442 -0.6311607607

H -1.8819968545 1.5517030431 -2.5043228465
H -0.4607077201 -0.3522461617 -1.6881861247
O -1.3871777257 -2.7272036112 -1.2821787546
H -3.5312183379 -3.688562878 -0.2639321854
H -6.0084567579 -3.7884859711 0.0288871378
H -7.4042685212 -1.8615740182 -0.7052787955
H -6.3474226232 0.15114475 -1.6757597773
K 0.0457720268 3.9135975953 0.453668262
K -2.4322960335 -4.449148687 -2.8628358296

TS_A-B_ortho_2Kcation_acetonitrile_conf19
C 11.645393 -14.739864 -1.91517
C 13.06578 -16.238812 -1.5033
C 14.069328 -16.838403 -3.632402
C 12.79085 -12.318152 0.731815
C 13.307383 -11.3319 -0.076345
C 13.248788 -11.398368 -1.468997
C 12.695389 -12.470762 -2.110298
C 12.184717 -13.538078 -1.32315
C 12.206891 -13.44329 0.112795
C 14.209182 -9.468484 -0.902908
H 12.80763 -12.210785 1.811752
H 12.610816 -12.478701 -3.190524
H 13.687519 -8.50359 -0.912243
O 13.878724 -10.16542 0.304289
O 13.77976 -10.259179 -2.003838
H 15.296675 -9.32708 -0.948348
N 11.591176 -14.463686 0.838311
S 12.282071 -15.092203 2.119846
O 13.70596 -15.470344 1.929463
O 11.4144 -16.25107 2.500236
C 11.674392 -14.827955 -5.71955
C 10.465275 -15.47116 -5.96451
C 12.083422 -14.565482 -4.415712
H 10.150706 -15.675128 -6.98569
H 13.056465 -14.112901 -4.247982
C 9.664126 -15.865415 -4.892787
C 11.280857 -14.931974 -3.32048

H 8.724305 -16.383352 -5.071947
C 10.076618 -15.606292 -3.59499
H 9.45965 -15.938002 -2.75596
H 12.314304 -14.535909 -6.54968
C 13.404213 -13.752121 4.253148
C 13.355764 -12.875516 5.33344
C 12.250165 -13.977695 3.509127
H 14.258506 -12.693882 5.915331
C 12.17354 -12.217934 5.683779
C 11.05886 -13.325042 3.83157
H 10.168789 -13.483476 3.223557
C 11.026917 -12.456003 4.911478
H 10.098889 -11.943044 5.161942
H 14.328386 -14.251103 3.972886
C 12.122782 -11.281175 6.850175
H 13.114238 -11.115772 7.282518
H 11.713275 -10.304731 6.565365
H 11.476277 -11.671619 7.646222
H 10.955905 -15.244316 -1.241482
H 14.814609 -16.69086 -4.413039
C 14.094253 -16.096923 -2.492436
C 12.168641 -17.378088 -1.557025
C 12.115794 -18.118308 -2.806886
C 13.053169 -17.832233 -3.83965
C 12.958521 -18.548615 -5.046104
C 11.977822 -19.508256 -5.229222
C 11.064131 -19.796221 -4.204361
C 11.142104 -19.111113 -3.005937
H 14.852022 -15.327461 -2.347009
H 13.242219 -15.82889 -0.508808
O 11.415264 -17.664363 -0.572095
H 10.4447 -19.314889 -2.195153
H 10.298785 -20.554402 -4.355983
H 11.919476 -20.047367 -6.172747
H 13.670869 -18.334766 -5.842295
K 9.492237 -16.312748 0.674784
K 13.217623 -18.07898 1.429688

TS_A-B_ortho_2Kcation_acetonitrile_conf20
C 11.817046 -14.782667 -2.051421
C 13.451331 -16.129912 -1.381413
C 14.692301 -16.715423 -3.392162
C 8.762224 -16.161901 -0.324979
C 8.778778 -15.353817 0.78456
C 9.733822 -14.352196 0.981245
C 10.716327 -14.13153 0.065152
C 10.773806 -14.951926 -1.099151
C 9.76645 -15.975013 -1.32332
C 8.264044 -14.245389 2.648161
H 7.94937 -16.868489 -0.461828
H 11.425505 -13.32424 0.205674
H 7.46764 -13.49322 2.561361
O 7.918834 -15.377586 1.83332
O 9.4901 -13.717649 2.168609
H 8.382981 -14.574701 3.68539
N 9.798028 -16.656826 -2.515885
S 8.7836 -17.891312 -2.756923
O 8.759512 -18.882117 -1.650299
O 9.126646 -18.435524 -4.084988
C 14.105467 -11.780523 -1.542677
C 14.141895 -11.269445 -2.839104
C 13.357301 -12.915031 -1.253489
H 14.72956 -10.381703 -3.061248
H 13.393209 -13.327702 -0.246831
C 13.437099 -11.91592 -3.850413
C 12.619056 -13.567446 -2.255134
H 13.468135 -11.535325 -4.869184
C 12.697744 -13.056668 -3.560019
H 12.147992 -13.560466 -4.35704
H 14.676084 -11.298962 -0.751442
C 6.051691 -18.104027 -2.595434
C 4.753488 -17.632406 -2.737626
C 7.121772 -17.25135 -2.867634
H 3.916431 -18.296669 -2.525157
C 4.498596 -16.316857 -3.146555
C 6.896059 -15.938297 -3.273404

H 7.738658 -15.274836 -3.461135
C 5.589664 -15.480529 -3.404577
H 5.410718 -14.45024 -3.709965
H 6.242629 -19.124485 -2.270601
C 3.093466 -15.828037 -3.31254
H 2.454655 -16.13906 -2.478248
H 3.047186 -14.736984 -3.385787
H 2.63893 -16.235549 -4.224791
H 11.637036 -15.322559 -2.979725
H 15.48168 -16.513813 -4.11476
C 14.494555 -15.890416 -2.323417
C 12.724272 -17.375747 -1.39507
C 12.889632 -18.213617 -2.59578
C 13.891466 -17.89097 -3.559787
C 14.055336 -18.74713 -4.673842
C 13.257674 -19.867758 -4.839263
C 12.26172 -20.171992 -3.894948
C 12.099739 -19.358672 -2.785407
H 15.116438 -15.004806 -2.199328
H 13.479076 -15.615849 -0.422037
O 11.971875 -17.708752 -0.449477
H 11.315913 -19.568819 -2.060216
H 11.618842 -21.037854 -4.035992
H 13.40873 -20.519375 -5.698188
H 14.838501 -18.520469 -5.397746
K 11.202939 -17.240581 -5.046033
K 10.017391 -18.885449 0.652457

TS_A-B_ortho_2Kcation_acetonitrile_conf21
C 12.299262 -14.821166 -1.890159
C 13.168 -16.638121 -1.414623
C 13.989741 -17.321023 -3.581337
C 13.073836 -14.667095 -5.564244
C 11.879257 -15.114192 -6.066136
C 10.756658 -15.318104 -5.259655
C 10.812544 -15.160106 -3.906251
C 12.044943 -14.770324 -3.312765
C 13.167958 -14.43976 -4.165396

C 10.241619 -15.921777 -7.32716
H 13.918508 -14.49401 -6.223041
H 9.930212 -15.367389 -3.315653
H 9.627012 -15.436723 -8.091371
O 11.559509 -15.369804 -7.361165
O 9.691432 -15.67546 -6.04253
H 10.307921 -17.008839 -7.499333
N 14.264283 -13.852527 -3.564778
S 15.732768 -13.963051 -4.22701
O 16.093131 -15.275647 -4.767236
O 16.631591 -13.370007 -3.204412
C 9.142816 -14.726709 0.286818
C 9.604003 -13.981827 1.373609
C 9.99088 -15.053766 -0.770304
H 8.928507 -13.70128 2.179706
H 9.603706 -15.683792 -1.562398
C 10.944252 -13.585824 1.399544
C 11.327203 -14.604575 -0.803893
H 11.328309 -13.005069 2.237049
C 11.780595 -13.901054 0.332465
H 12.823474 -13.577313 0.35758
H 8.104928 -15.05485 0.253622
C 16.910892 -12.850431 -6.450037
C 17.030071 -11.913163 -7.466685
C 15.808668 -12.802576 -5.594746
H 17.890176 -11.955168 -8.134477
C 16.062378 -10.916926 -7.658373
C 14.831661 -11.825034 -5.770827
H 13.941333 -11.830917 -5.140294
C 14.961853 -10.892787 -6.797837
H 14.185871 -10.141879 -6.946328
H 17.660389 -13.628135 -6.317553
C 16.200291 -9.924679 -8.771344
H 16.15233 -10.415578 -9.751391
H 15.409766 -9.16803 -8.743333
H 17.164675 -9.404167 -8.730334
H 13.243279 -14.325863 -1.661397
H 14.76274 -17.323166 -4.346656

C 14.235238 -16.767605 -2.364097
C 11.992441 -17.494029 -1.538228
C 11.717545 -18.011717 -2.872539
C 12.706271 -17.890027 -3.886908
C 12.394182 -18.345749 -5.179542
C 11.151605 -18.896243 -5.45745
C 10.18422 -19.021001 -4.451093
C 10.475849 -18.591368 -3.167304
H 15.201703 -16.31332 -2.155138
H 13.429346 -16.373661 -0.385374
O 11.21689 -17.689044 -0.568437
H 9.74516 -18.677084 -2.36453
H 9.212937 -19.454393 -4.679076
H 10.932398 -19.251606 -6.463662
H 13.145212 -18.257423 -5.964479
K 15.056123 -11.602458 -2.140965
K 10.951727 -16.89308 1.808689

TS_A-B_ortho_2Kcation_acetonitrile_conf2
C -10.40144 16.956016 -1.898836
C -11.305955 18.106791 -0.010185
C -9.415846 19.29319 0.929149
C -12.910106 17.873645 -4.552923
C -13.733791 16.77682 -4.456055
C -13.473971 15.712638 -3.586723
C -12.384044 15.713263 -2.767484
C -11.514722 16.846078 -2.769244
C -11.770961 17.944652 -3.690896
C -15.332312 15.240599 -4.72688
H -13.093641 18.634829 -5.307353
H -12.170646 14.852202 -2.145677
H -15.388263 14.555879 -5.581295
O -14.848791 16.51452 -5.182959
O -14.420226 14.739506 -3.764057
H -16.322013 15.378191 -4.271483
N -10.819313 18.925366 -3.756151
S -11.027632 20.160676 -4.762056
O -10.758286 19.734224 -6.16516

O -12.307915 20.894483 -4.602873
C -9.667298 13.984236 0.358758
C -8.274872 14.048978 0.406059
C -10.392493 14.904482 -0.386518
H -7.712928 13.32676 0.993947
H -11.478469 14.875576 -0.33862
C -7.613297 15.06431 -0.28004
C -9.742569 15.918389 -1.116596
H -6.529346 15.143925 -0.227985
C -8.339536 15.990185 -1.018112
H -7.820596 16.802621 -1.528946
H -10.195053 13.219627 0.925021
C -9.752994 22.544418 -4.7976
C -8.734888 23.433846 -4.477457
C -9.719952 21.251891 -4.275004
H -8.765345 24.447354 -4.875595
C -7.67633 23.055442 -3.644305
C -8.675528 20.847385 -3.444874
H -8.689912 19.855649 -2.999635
C -7.666995 21.751012 -3.137092
H -6.868388 21.447374 -2.460514
H -10.577997 22.852619 -5.436805
C -6.582474 24.016909 -3.297978
H -5.687041 23.838764 -3.907802
H -6.274922 23.917108 -2.250567
H -6.884306 25.055727 -3.465633
H -9.758096 17.808662 -2.111671
H -8.546291 19.328319 1.584155
C -10.189454 18.16713 0.856664
C -11.762139 19.267731 -0.728559
C -10.907253 20.461562 -0.655906
C -9.75734 20.46516 0.184234
C -8.998365 21.649327 0.291638
C -9.361196 22.794701 -0.391695
C -10.493086 22.789717 -1.219152
C -11.247659 21.636927 -1.339889
H -9.93035 17.287991 1.445826
H -12.011647 17.281549 0.064954

O -12.846316 19.253082 -1.371167
H -12.108897 21.617335 -2.004479
H -10.76707 23.684938 -1.774402
H -8.767915 23.702168 -0.288629
H -8.123428 21.651921 0.941756
K -14.382974 20.325748 -3.074073
K -9.605759 17.362591 -5.848374

TS_A-B_ortho_2Kcation_acetonitrile_conf3
C -10.76224 16.861891 -1.31953
C -10.757077 17.905582 0.762729
C -9.106432 19.633092 0.40425
C -10.771298 13.33336 0.000999
C -9.479958 12.963276 -0.306821
C -8.59492 13.829619 -0.953489
C -8.958956 15.104808 -1.299307
C -10.272209 15.552397 -0.974391
C -11.189024 14.650924 -0.322882
C -7.504919 11.96229 -0.461917
H -11.454139 12.655187 0.502006
H -8.2669 15.739541 -1.840526
H -7.155189 11.127642 -1.074716
O -8.881787 11.755175 -0.121734
O -7.421893 13.17171 -1.208659
H -6.916867 12.052215 0.463283
N -12.457771 15.098368 0.027004
S -13.737323 14.325148 -0.530015
O -13.460707 12.962439 -1.05317
O -14.819073 14.483665 0.458609
C -8.295257 19.170544 -3.048063
C -9.168522 19.901814 -3.846281
C -8.775835 18.164764 -2.215062
H -8.788241 20.692591 -4.489683
H -8.082424 17.657596 -1.550637
C -10.538523 19.6259 -3.809641
C -10.151972 17.853521 -2.176199
H -11.229423 20.206177 -4.41783
C -11.018941 18.62794 -2.981216

H -12.090317 18.425665 -2.930196
H -7.230937 19.39733 -3.054501
C -14.73144 14.544648 -3.108534
C -15.182809 15.25169 -4.222433
C -14.273697 15.235878 -1.986306
H -15.551529 14.706675 -5.091298
C -15.202019 16.649066 -4.234649
C -14.297793 16.632861 -1.973231
H -13.944282 17.185604 -1.099548
C -14.757271 17.322484 -3.087404
H -14.774656 18.413094 -3.066811
H -14.783189 13.456364 -3.083341
C -15.696093 17.41082 -5.424697
H -14.926821 18.086199 -5.819014
H -16.558281 18.037404 -5.166046
H -16.003276 16.744841 -6.23681
H -11.84866 16.912801 -1.287835
H -8.080275 19.996129 0.450156
C -9.4335 18.394911 0.883567
C -11.848087 18.788437 0.401747
C -11.468945 20.078559 -0.169787
C -10.106077 20.482505 -0.176951
C -9.773609 21.712452 -0.771622
C -10.75004 22.510108 -1.347124
C -12.091948 22.108361 -1.333541
C -12.443896 20.906197 -0.741259
H -8.664106 17.745342 1.300266
H -11.031992 16.965919 1.241085
O -13.053723 18.443643 0.517847
H -13.478579 20.567714 -0.715361
H -12.853261 22.741554 -1.784637
H -10.472223 23.456485 -1.807387
H -8.730817 22.029251 -0.78056
K -13.996684 16.501979 1.989029
K -11.639123 13.123199 -3.008845

TS_A-B_ortho_2Kcation_acetonitrile_conf5

C 1.227488 0.592486 0.99956

C 1.506206 -0.15595 -0.947638
C 3.489896 0.93478 -1.827611
C -2.414146 1.46574 0.606177
C -2.320149 2.837744 0.643474
C -1.109014 3.499921 0.849119
C 0.063806 2.814314 0.997516
C 0.029746 1.395474 0.924697
C -1.22834 0.716615 0.757247
C -2.720678 5.030168 0.683442
H -3.379431 0.982079 0.494642
H 0.984713 3.344468 1.209748
H -3.165981 5.546968 1.542219
O -3.326126 3.738572 0.551066
O -1.32754 4.847269 0.900561
H -2.881595 5.597334 -0.24252
N -1.221963 -0.677745 0.804282
S -2.049909 -1.538914 -0.238991
O -1.865036 -1.134551 -1.656217
O -1.648798 -2.95687 0.023076
C 4.469995 2.538275 1.426475
C 5.160058 1.68942 2.286055
C 3.185175 2.218255 0.998644
H 6.164905 1.943392 2.616409
H 2.688804 2.867872 0.283531
C 4.561361 0.503864 2.71244
C 2.553104 1.039291 1.432778
H 5.097299 -0.174555 3.372854
C 3.283243 0.185683 2.280486
H 2.826009 -0.755828 2.595202
H 4.938164 3.45497 1.073805
C -4.686318 -1.182092 -0.967615
C -6.050052 -1.111892 -0.698935
C -3.802223 -1.456752 0.071681
H -6.743809 -0.891338 -1.50927
C -6.548254 -1.311372 0.591757
C -4.272994 -1.648282 1.371676
H -3.570949 -1.830081 2.184722
C -5.63504 -1.57707 1.622506

H -6.003485 -1.721447 2.637596
H -4.299282 -1.011042 -1.968857
C -8.017291 -1.251613 0.872499
H -8.433461 -2.257077 1.017033
H -8.570039 -0.786371 0.050511
H -8.232608 -0.688026 1.787331
H 1.010762 -0.42212 1.32725
H 3.977158 1.76188 -2.342494
C 2.136979 0.893428 -1.694147
C 2.263679 -1.341211 -0.591107
C 3.711366 -1.252459 -0.685816
C 4.313165 -0.110068 -1.285769
C 5.716925 -0.042999 -1.337499
C 6.497977 -1.06053 -0.817247
C 5.900922 -2.190502 -0.238635
C 4.522527 -2.283482 -0.183508
H 1.515582 1.698386 -2.085671
H 0.432623 -0.303707 -1.066391
O 1.679447 -2.381699 -0.148693
H 4.034405 -3.149126 0.261086
H 6.522504 -2.987067 0.164727
H 7.582717 -0.986042 -0.863039
H 6.185639 0.828706 -1.792996
K 0.141645 -2.823773 1.978704
K 0.008442 -3.003864 -2.210166

TS_A-B_ortho_2Kcation_acetonitrile_conf6
C 0.871022 -0.129007 0.68279
C 1.577458 -0.275337 -1.256248
C 3.294435 1.424057 -1.23908
C 3.068614 2.630107 1.976399
C 4.026172 1.879733 2.607734
C 3.907206 0.497213 2.772315
C 2.862459 -0.197525 2.237781
C 1.869995 0.512844 1.50766
C 1.933561 1.958865 1.448975
C 5.862937 1.126082 3.59951
H 3.17935 3.706081 1.890294

H 2.834044 -1.273136 2.350099
H 6.210091 1.237052 4.631101
O 5.166962 2.310564 3.205831
O 4.961642 0.033525 3.512575
H 6.707632 0.965026 2.909395
N 0.835436 2.617865 0.932781
S 0.975116 4.086437 0.275397
O 2.128097 4.280705 -0.60675
O -0.373188 4.353358 -0.287134
C 0.100352 -3.76309 1.639635
C -1.26332 -3.820397 1.346785
C 0.83675 -2.602622 1.40518
H -1.839115 -4.718437 1.562995
H 1.903455 -2.62559 1.594091
C -1.880944 -2.696984 0.78981
C 0.22359 -1.432853 0.911191
H -2.943575 -2.716807 0.551816
C -1.143334 -1.536369 0.574927
H -1.637861 -0.660989 0.147746
H 0.602257 -4.631395 2.06401
C 1.516579 6.586171 1.278949
C 1.576222 7.549411 2.276325
C 1.139079 5.282068 1.602909
H 1.874586 8.565935 2.021118
C 1.268723 7.240109 3.608582
C 0.835325 4.950432 2.92134
H 0.602317 3.91617 3.179114
C 0.904176 5.925959 3.913295
H 0.686603 5.659658 4.947563
H 1.7717 6.831543 0.250071
C 1.348427 8.290647 4.672726
H 0.685794 9.136655 4.452608
H 2.362147 8.701702 4.754263
H 1.070897 7.896968 5.655686
H 0.138327 0.604729 0.345788
H 3.608044 2.458349 -1.361139
C 2.025739 1.053393 -1.556966
C 2.560003 -1.337869 -1.066404

C 3.893019 -0.910642 -0.661177
C 4.233637 0.468432 -0.720234
C 5.504842 0.860772 -0.266772
C 6.403217 -0.074859 0.22506
C 6.06556 -1.434369 0.270209
C 4.822161 -1.844033 -0.18127
H 1.316085 1.793821 -1.920952
H 0.631273 -0.606483 -1.695337
O 2.241516 -2.550661 -1.156733
H 4.531332 -2.893099 -0.157977
H 6.777269 -2.160461 0.656854
H 7.387355 0.248446 0.562317
H 5.775114 1.915914 -0.306092
K -1.885511 2.976879 1.304173
K 0.150269 -3.896008 -1.567923

TS_A-B_ortho_2Kcation_acetonitrile_conf7
C 1.226304 0.814422 0.618718
C 1.386698 -0.551762 -1.129912
C 2.855818 0.557459 -2.723088
C 1.798092 -1.447498 3.580073
C 0.486749 -1.729779 3.871537
C -0.584575 -1.189149 3.154132
C -0.372213 -0.344207 2.107988
C 0.966792 -0.027572 1.735179
C 2.083641 -0.565091 2.493871
C -1.411144 -2.377336 4.843366
H 2.58506 -1.833488 4.220633
H -1.209821 0.103963 1.586377
H -1.708609 -1.819067 5.741977
O 0.016847 -2.538867 4.853714
O -1.761662 -1.650725 3.677206
H -1.882118 -3.365226 4.819872
N 3.34764 -0.138459 2.165265
S 4.629469 -0.809384 2.885501
O 4.615507 -2.294838 2.886042
O 5.81101 -0.188317 2.256285
C -1.79866 2.483654 -0.984666

C -1.331516 3.791936 -1.101087
C -0.995767 1.495908 -0.427621
H -1.963123 4.561684 -1.53853
H -1.362063 0.471137 -0.400724
C -0.04394 4.10051 -0.671751
C 0.297654 1.792492 0.033739
H 0.337483 5.114817 -0.768776
C 0.760808 3.107945 -0.124646
H 1.769709 3.355353 0.210186
H -2.79318 2.22684 -1.343178
C 5.329029 -1.115167 5.521974
C 5.394364 -0.713535 6.849525
C 4.638118 -0.325262 4.602717
H 5.933392 -1.329082 7.568996
C 4.777982 0.467619 7.282959
C 4.013982 0.852019 5.007872
H 3.458185 1.448692 4.286405
C 4.08412 1.236068 6.342442
H 3.586655 2.150276 6.664147
H 5.804251 -2.036922 5.193568
C 4.87284 0.897165 8.71381
H 5.870818 1.293417 8.941589
H 4.703876 0.060115 9.400584
H 4.148921 1.682496 8.952728
H 2.263538 1.140722 0.564201
H 3.011254 1.242583 -3.555206
C 1.603959 0.328477 -2.230139
C 2.449721 -1.400474 -0.649853
C 3.805901 -1.081818 -1.129928
C 3.987194 -0.135177 -2.182797
C 5.299031 0.106365 -2.653872
C 6.389689 -0.544657 -2.100652
C 6.207322 -1.461511 -1.051004
C 4.92864 -1.730532 -0.591594
H 0.748983 0.852816 -2.655168
H 0.377761 -0.91005 -0.932574
O 2.231243 -2.338088 0.152964
H 4.779449 -2.417481 0.239327

H 7.065 -1.952907 -0.597423
H 7.389502 -0.351185 -2.485436
H 5.438733 0.806441 -3.477992
K 5.090354 1.366886 0.325954
K 2.802169 -3.977369 2.000588

TS_A-B_para_2Kcation_acetonitrile
C 12.267273 -16.852093 -1.108616
C 14.493382 -17.026145 -0.781032
C 10.626639 -19.721715 0.684723
C 10.473166 -19.144467 1.919923
C 10.972247 -17.877282 2.225147
C 11.604981 -17.103403 1.291736
C 11.717418 -17.620224 -0.028882
C 11.282742 -18.964042 -0.318432
C 9.706639 -18.529482 3.881005
H 10.227094 -20.701407 0.442991
H 12.032522 -16.141239 1.559231
H 8.743357 -18.071324 3.610265
O 9.9225 -19.69113 3.071853
O 10.775329 -17.640394 3.587198
H 9.732404 -18.80726 4.938278
N 11.483038 -19.495044 -1.581201
S 10.161582 -19.687336 -2.493303
O 9.020888 -20.270894 -1.778104
O 10.644813 -20.365345 -3.719168
C 11.3506 -13.182236 -0.767
C 11.962563 -12.662186 -1.903937
C 11.433493 -14.540501 -0.478029
H 11.897702 -11.598645 -2.123998
H 10.891927 -14.928446 0.379741
C 12.636006 -13.520421 -2.775911
C 12.139409 -15.417234 -1.32213
H 13.093563 -13.13016 -3.682789
C 12.71253 -14.873626 -2.490654
H 13.226522 -15.543992 -3.18108
H 10.792103 -12.52704 -0.101357
C 8.909331 -17.285195 -2.068319

C 8.661348 -15.945063 -2.340696
C 9.693703 -18.030701 -2.949763
H 8.047389 -15.361609 -1.65417
C 9.185901 -15.326792 -3.481666
C 10.230427 -17.436245 -4.090599
H 10.830801 -18.034155 -4.773578
C 9.968548 -16.096225 -4.350754
H 10.37901 -15.631583 -5.247617
H 8.48767 -17.765708 -1.186715
C 8.897293 -13.887238 -3.774829
H 9.656065 -13.446813 -4.430886
H 7.92813 -13.77043 -4.277724
H 8.855464 -13.288552 -2.857616
H 12.349344 -17.429443 -2.028468
H 14.70201 -16.354615 -1.610929
C 14.631467 -18.421617 -0.976184
C 14.578646 -19.301073 0.072581
C 14.480919 -18.843014 1.432714
C 14.595939 -17.393244 1.651887
C 14.610297 -16.50083 0.549865
C 14.647097 -15.113957 0.796267
C 14.688065 -14.627617 2.090949
C 14.703782 -15.51315 3.179803
C 14.656191 -16.876896 2.955072
H 14.699321 -18.787136 -2.002861
H 14.598047 -20.381027 -0.074176
O 14.303657 -19.638571 2.389706
H 14.690351 -17.581029 3.786155
H 14.761395 -15.124262 4.194343
H 14.722847 -13.554152 2.265218
H 14.64379 -14.426385 -0.04977
K 12.94458 -21.31462 -2.97105
K 12.616473 -19.916363 4.227987

TS_A-B_para_2Kcation_acetonitrile_conf15

C 12.390381 -16.807412 -0.914913
C 14.335296 -17.166525 -0.419782
C 11.114368 -13.951805 1.17013

C 11.389512 -12.914576 0.313817
C 12.00295 -13.103916 -0.931425
C 12.36338 -14.346328 -1.369239
C 12.095557 -15.456219 -0.521806
C 11.478528 -15.260612 0.768158
C 11.548789 -10.934728 -0.699807
H 10.612093 -13.794536 2.117809
H 12.803861 -14.478855 -2.352108
H 10.663391 -10.508473 -1.190567
O 11.145882 -11.594051 0.50177
O 12.155266 -11.898227 -1.553429
H 12.275308 -10.151051 -0.455062
N 11.242173 -16.358199 1.584127
S 9.691193 -16.659649 1.936114
O 8.89541 -15.480655 2.295574
O 9.718849 -17.791734 2.893053
C 12.958174 -17.303335 -4.660013
C 12.010424 -18.290685 -4.92278
C 13.11316 -16.804346 -3.370927
H 11.891667 -18.678484 -5.932326
H 13.878323 -16.05042 -3.187355
C 11.227295 -18.784962 -3.882735
C 12.32114 -17.280653 -2.316005
H 10.489903 -19.563659 -4.071857
C 11.390613 -18.293328 -2.592219
H 10.773143 -18.676983 -1.779272
H 13.584865 -16.921385 -5.463304
C 8.71925 -16.414527 -0.625776
C 8.204526 -16.904719 -1.818303
C 8.986528 -17.297653 0.423514
H 7.998465 -16.215429 -2.636889
C 7.930333 -18.268577 -1.98556
C 8.739118 -18.659865 0.274553
H 8.936819 -19.329902 1.108165
C 8.205097 -19.133462 -0.921793
H 7.98841 -20.196547 -1.030189
H 8.893801 -15.347317 -0.496082
C 7.341643 -18.774842 -3.266536

H 7.422375 -19.864236 -3.349782
H 6.275827 -18.523122 -3.342456
H 7.832299 -18.329553 -4.140516
H 11.940934 -17.512529 -0.213521
H 14.693255 -16.639459 -1.30447
C 14.345248 -18.58766 -0.46016
C 14.361317 -19.351068 0.685637
C 14.485269 -18.762752 1.979524
C 14.706517 -17.31811 2.031031
C 14.63817 -16.538686 0.849562
C 14.830751 -15.149562 0.949357
C 15.075769 -14.54803 2.170759
C 15.163302 -15.322375 3.33704
C 14.987312 -16.691852 3.260524
H 14.223064 -19.073201 -1.428642
H 14.194914 -20.428964 0.622777
O 14.391759 -19.444759 3.058759
H 15.087572 -17.318973 4.147522
H 15.387003 -14.847978 4.290389
H 15.213954 -13.470145 2.224822
H 14.776137 -14.544071 0.045631
K 12.138309 -17.841045 3.771439
K 15.931438 -21.485252 3.011022

TS_A-B_para_2Kcation_acetonitrile_conf18

C 12.13492 -17.217268 -0.982081
C 14.301718 -17.037599 -0.61571
C 11.946862 -16.850578 -4.698766
C 11.459937 -18.095391 -5.024321
C 11.256282 -19.100201 -4.069668
C 11.505653 -18.894771 -2.74224
C 11.982623 -17.61299 -2.347814
C 12.243596 -16.598659 -3.342114
C 10.665292 -19.900707 -6.065514
H 12.094789 -16.076299 -5.444108
H 11.277058 -19.667043 -2.015483
H 9.617133 -19.972542 -6.381575
O 11.117826 -18.556436 -6.249176

O 10.774262 -20.219336 -4.682747
H 11.299515 -20.576641 -6.652148
N 12.839139 -15.4116 -2.931202
S 12.026582 -14.03457 -3.158184
O 11.376041 -13.904307 -4.464597
O 12.970573 -12.972284 -2.728894
C 11.194197 -19.923885 1.518303
C 10.407689 -19.180975 2.401528
C 11.803891 -19.30617 0.428661
H 9.869349 -19.680042 3.2079
H 12.417001 -19.898434 -0.246176
C 10.291249 -17.798223 2.22225
C 11.633781 -17.930015 0.189851
H 9.67685 -17.205159 2.900122
C 10.917625 -17.180077 1.144291
H 10.806312 -16.105444 0.990655
H 11.307406 -20.99757 1.657845
C 9.632796 -14.92213 -2.14746
C 8.685347 -15.076401 -1.143666
C 10.730491 -14.085722 -1.932454
H 7.829902 -15.730813 -1.30948
C 8.804019 -14.399321 0.077281
C 10.868885 -13.400533 -0.728327
H 11.719927 -12.737142 -0.588986
C 9.905277 -13.556698 0.264137
H 10.005573 -13.00837 1.201588
H 9.521955 -15.437222 -3.100416
C 7.768856 -14.568901 1.146811
H 8.133232 -14.23026 2.123286
H 6.863879 -13.989153 0.92366
H 7.454069 -15.61525 1.242633
H 12.104486 -16.134916 -0.866285
H 14.292658 -16.547008 -1.585954
C 14.800701 -18.356637 -0.506258
C 15.113064 -18.919726 0.70612
C 14.991221 -18.179869 1.931831
C 14.637051 -16.753385 1.811623
C 14.299665 -16.197865 0.552116

C 13.894737 -14.846812 0.492026
C 13.860996 -14.063638 1.632288
C 14.249447 -14.599706 2.8707
C 14.627649 -15.928911 2.95095
H 14.87633 -18.957573 -1.414131
H 15.440594 -19.954602 0.782631
O 15.123153 -18.713375 3.064057
H 14.98217 -16.354253 3.89129
H 14.26513 -13.967471 3.75627
H 13.540017 -13.024923 1.568188
H 13.55954 -14.438225 -0.466551
K 15.307266 -14.095716 -2.867391
K 12.850934 -18.396618 4.188708

TS_A-B_para_2Kcation_acetonitrile_conf19
C 11.646045 -15.273279 -1.373212
C 13.552951 -15.216591 -0.787056
C 9.145884 -16.409913 1.201315
C 9.024573 -15.253057 1.929631
C 9.792326 -14.114422 1.664659
C 10.683043 -14.079232 0.625581
C 10.799559 -15.230769 -0.194293
C 10.078524 -16.432431 0.133037
C 8.471047 -13.672824 3.401368
H 8.558637 -17.294116 1.423224
H 11.298918 -13.199073 0.466323
H 7.555034 -13.089693 3.232766
O 8.244184 -15.028236 3.019106
O 9.524569 -13.152719 2.594554
H 8.767469 -13.63864 4.456035
N 10.322649 -17.596865 -0.582437
S 9.083701 -18.25627 -1.381775
O 7.815141 -18.282659 -0.64666
O 9.608902 -19.537355 -1.913833
C 11.26108 -12.086233 -3.437937
C 11.423145 -12.604408 -4.723148
C 11.334104 -12.917629 -2.326961
H 11.359573 -11.948372 -5.589301

H 11.179953 -12.485928 -1.342901
C 11.653285 -13.967053 -4.889098
C 11.574177 -14.293791 -2.474019
H 11.75935 -14.390816 -5.886751
C 11.733735 -14.79586 -3.777226
H 11.902515 -15.865259 -3.910561
H 11.068507 -11.024212 -3.300063
C 8.258784 -15.928252 -2.587552
C 8.122036 -15.058518 -3.660666
C 8.834833 -17.18591 -2.788801
H 7.678496 -14.07529 -3.502342
C 8.537095 -15.423686 -4.947842
C 9.264854 -17.566067 -4.057236
H 9.692846 -18.556034 -4.199085
C 9.105591 -16.688595 -5.126165
H 9.423763 -16.992475 -6.12419
H 7.90555 -15.645436 -1.596923
C 8.356786 -14.484657 -6.100412
H 7.302875 -14.417395 -6.400277
H 8.677676 -13.467181 -5.844459
H 8.923965 -14.806919 -6.980795
H 11.669643 -16.293719 -1.764876
H 13.581357 -14.147579 -0.566714
C 13.530584 -16.100874 0.335227
C 13.890287 -17.416906 0.233511
C 14.386725 -17.969152 -0.990497
C 14.621185 -17.036939 -2.091106
C 14.310252 -15.657488 -1.941786
C 14.67029 -14.772862 -2.974772
C 15.281528 -15.230923 -4.128367
C 15.517311 -16.603462 -4.30962
C 15.187952 -17.489622 -3.300949
H 13.110977 -15.732369 1.27074
H 13.798617 -18.085459 1.090407
O 14.643078 -19.215431 -1.106764
H 15.303219 -18.565543 -3.446261
H 15.938685 -16.964033 -5.245884
H 15.54833 -14.526819 -4.913695

H 14.441577 -13.713167 -2.864328
K 11.90839 -19.842938 -0.752385
K 17.228977 -19.201324 -1.449097

TS_A-B_para_2Kcation_acetonitrile_conf20
C 11.482216 -15.273532 -1.40253
C 13.62963 -15.085591 -0.797716
C 11.045981 -18.275086 -3.637212
C 10.861033 -19.234303 -2.674424
C 10.933857 -18.954849 -1.309481
C 11.175395 -17.691757 -0.843446
C 11.321836 -16.64563 -1.796911
C 11.294794 -16.946214 -3.206898
C 10.164527 -20.985091 -1.550432
H 10.954322 -18.486242 -4.697549
H 11.266711 -17.495657 0.220064
H 9.07853 -20.810859 -1.517918
O 10.668131 -20.600433 -2.8341
O 10.824314 -20.154438 -0.603777
H 10.406785 -22.033765 -1.358593
N 11.458833 -15.933061 -4.131514
S 10.130001 -15.516179 -4.954427
O 9.339352 -16.658093 -5.429107
O 10.58994 -14.518955 -5.947036
C 9.182293 -14.502496 1.518869
C 9.452788 -13.149922 1.70833
C 9.837901 -15.221053 0.525347
H 8.937209 -12.591452 2.486903
H 9.558568 -16.257143 0.353267
C 10.36662 -12.507921 0.871412
C 10.791051 -14.603203 -0.303732
H 10.56417 -11.4445 0.990469
C 11.016237 -13.224233 -0.121119
H 11.714186 -12.714538 -0.787596
H 8.441208 -15.001847 2.140231
C 8.241777 -15.380607 -2.964488
C 7.52177 -14.724878 -1.972144
C 9.117282 -14.652121 -3.769903

H 6.830275 -15.2902 -1.34667
C 7.660922 -13.34826 -1.762681
C 9.284726 -13.282293 -3.570273
H 9.965067 -12.727 -4.213141
C 8.555407 -12.64182 -2.576387
H 8.67481 -11.568331 -2.427635
H 8.113364 -16.447834 -3.139176
C 6.849651 -12.641954 -0.721463
H 5.906094 -12.26891 -1.14184
H 6.589858 -13.305387 0.111038
H 7.381311 -11.776084 -0.311052
H 11.689695 -14.622313 -2.25216
H 13.490531 -14.076985 -0.413692
C 13.706934 -16.153618 0.127616
C 14.082447 -17.410094 -0.268874
C 14.47303 -17.68876 -1.626305
C 14.723243 -16.518303 -2.481438
C 14.33301 -15.227233 -2.047723
C 14.683306 -14.113298 -2.835704
C 15.450446 -14.267913 -3.979051
C 15.825473 -15.55336 -4.41244
C 15.439323 -16.664974 -3.678904
H 13.361834 -15.983653 1.148001
H 14.054858 -18.248857 0.426704
O 14.636301 -18.857526 -2.0569
H 15.736835 -17.669096 -3.97529
H 16.459941 -15.670388 -5.291082
H 15.791752 -13.387258 -4.524292
H 14.40025 -13.116153 -2.496568
K 13.156022 -14.917355 -5.993531
K 13.4122 -21.055612 -1.871764

TS_A-B_para_2Kcation_acetonitrile_conf21
C 11.303028 -15.672279 -1.887563
C 13.119871 -15.222321 -0.747985
C 10.441808 -12.250442 -3.136944
C 10.646993 -12.442629 -4.484418
C 11.135102 -13.644573 -5.007482

C 11.41187 -14.719074 -4.209453
C 11.185264 -14.583236 -2.810549
C 10.737552 -13.32297 -2.269266
C 10.780909 -12.237655 -6.700862
H 10.055278 -11.31737 -2.740893
H 11.726243 -15.659101 -4.648911
H 9.88684 -12.312188 -7.333217
O 10.440148 -11.558849 -5.48871
O 11.244356 -13.540156 -6.364785
H 11.576785 -11.682106 -7.211879
N 10.678856 -13.181257 -0.886668
S 9.246067 -12.850965 -0.218874
O 8.422074 -11.891032 -0.959026
O 9.547892 -12.568831 1.205358
C 12.363993 -18.924815 -3.550175
C 11.456311 -19.811689 -2.967113
C 12.358944 -17.577196 -3.197617
H 11.407232 -20.847429 -3.305563
H 13.074073 -16.903695 -3.664585
C 10.586942 -19.349866 -1.973236
C 11.444532 -17.082222 -2.249084
H 9.865961 -20.026813 -1.514044
C 10.60253 -18.008039 -1.603568
H 9.908937 -17.643655 -0.84394
H 13.058815 -19.27433 -4.312111
C 7.784182 -14.814683 -1.456635
C 7.239744 -16.089178 -1.55216
C 8.38669 -14.413462 -0.2628
H 6.770739 -16.403836 -2.484178
C 7.273967 -16.975985 -0.468431
C 8.438216 -15.279041 0.827455
H 8.897646 -14.942285 1.754543
C 7.874765 -16.547544 0.720897
H 7.892625 -17.218272 1.580539
H 7.737247 -14.123345 -2.296405
C 6.674773 -18.344437 -0.583633
H 5.579731 -18.310189 -0.516208
H 6.914266 -18.810239 -1.547547

H 7.02388 -19.010154 0.214002
H 10.712565 -15.511842 -0.987333
H 12.720461 -14.211877 -0.696424
C 12.91975 -16.082512 0.359066
C 13.602801 -17.265827 0.484136
C 14.622477 -17.655361 -0.457689
C 14.948557 -16.691848 -1.516028
C 14.215781 -15.4834 -1.640095
C 14.536333 -14.601329 -2.691423
C 15.545442 -14.908581 -3.58757
C 16.272384 -16.101954 -3.455272
C 15.974315 -16.980431 -2.42888
H 12.136246 -15.826712 1.078281
H 13.417998 -17.929947 1.33028
O 15.182828 -18.781926 -0.400586
H 16.523999 -17.910549 -2.299792
H 17.071158 -16.330333 -4.157776
H 15.780114 -14.218393 -4.39573
H 13.96924 -13.675722 -2.798011
K 12.070745 -11.963376 1.179574
K 13.360924 -20.530879 -0.593706

TS_A-B_para_2Kcation_acetonitrile_conf16
C -13.084608 16.138181 0.740822
C -14.514019 17.589156 0.709637
C -11.13774 16.308047 -2.470669
C -11.857079 15.403253 -3.211321
C -12.982763 14.738033 -2.707951
C -13.431634 14.954066 -1.436309
C -12.718463 15.878936 -0.625201
C -11.567713 16.575973 -1.147455
C -12.612753 14.012232 -4.782276
H -10.251517 16.793679 -2.862845
H -14.283069 14.402977 -1.050276
H -12.105708 13.051577 -4.944489
O -11.636371 15.013578 -4.491148
O -13.491194 13.910723 -3.667444
H -13.180315 14.309738 -5.671918

N -10.896985 17.483835 -0.339897
S -9.354219 17.124849 -0.003546
O -8.559194 16.666401 -1.148126
O -8.842579 18.279035 0.77366
C -15.381417 13.521935 2.313298
C -14.623484 13.168207 3.427935
C -14.91555 14.485309 1.424702
H -14.990721 12.414093 4.120842
H -15.538458 14.761072 0.574088
C -13.400772 13.795241 3.654079
C -13.679453 15.115234 1.630728
H -12.802767 13.535634 4.526312
C -12.941108 14.766612 2.771375
H -11.980911 15.253386 2.946393
H -16.344531 13.047695 2.135951
C -9.772721 14.484405 0.645175
C -9.875207 13.416008 1.525733
C -9.436182 15.749593 1.133728
H -10.139737 12.430111 1.144023
C -9.630418 13.577337 2.895899
C -9.205495 15.938093 2.49397
H -8.927698 16.926134 2.853891
C -9.294151 14.852024 3.361901
H -9.089972 14.994272 4.423455
H -9.931028 14.338307 -0.422427
C -9.711887 12.406329 3.826404
H -8.841292 11.747005 3.714972
H -10.598263 11.792153 3.626267
H -9.746231 12.72082 4.87526
H -12.307838 16.722923 1.236874
H -15.286419 16.868336 0.440556
C -14.469222 18.01885 2.064368
C -13.88606 19.210151 2.43464
C -13.393186 20.13794 1.46891
C -13.634286 19.819447 0.062076
C -14.190949 18.569491 -0.306283
C -14.380819 18.295396 -1.672189
C -14.027988 19.216905 -2.64175

C -13.494905 20.461633 -2.275725
C -13.309821 20.756567 -0.937457
H -14.80983 17.324636 2.833
H -13.715398 19.427183 3.491685
O -12.752437 21.19703 1.794866
H -12.929745 21.730842 -0.626942
H -13.247315 21.194524 -3.040917
H -14.176254 18.979514 -3.6932
H -14.80385 17.334579 -1.96261
K -10.521277 20.173501 0.319193
K -14.099864 22.548352 3.497569

TS_A-B_para_2Kcation_acetonitrile_conf2
C -12.881288 16.087454 1.191193
C -14.578825 17.377734 0.647362
C -14.588224 12.857652 1.991941
C -14.269918 12.758301 3.326454
C -13.569903 13.759063 4.012581
C -13.133475 14.892156 3.386353
C -13.41034 15.030868 1.997079
C -14.180453 14.022576 1.307092
C -14.013786 12.107878 5.444423
H -15.124287 12.073974 1.467185
H -12.536123 15.620053 3.924839
H -13.259502 11.365607 5.733918
O -14.562545 11.745654 4.174391
O -13.404991 13.387746 5.314779
H -14.821978 12.155763 6.184531
N -14.548443 14.26226 -0.011861
S -14.03357 13.232275 -1.1446
O -14.119827 11.815181 -0.782736
O -14.701072 13.686501 -2.390126
C -10.682755 18.531802 3.104024
C -9.604329 18.634717 2.22243
C -11.784314 17.742996 2.779865
H -8.711114 19.189119 2.512283
H -12.617203 17.678654 3.475775
C -9.66758 17.993007 0.980762

C -11.829214 17.027316 1.569203
H -8.832421 18.060402 0.283205
C -10.77719 17.220622 0.651491
H -10.816106 16.70096 -0.30725
H -10.653199 19.041218 4.065685
C -11.406474 13.192734 -0.344441
C -10.081654 13.606137 -0.400649
C -12.301318 13.620667 -1.327766
H -9.383627 13.274835 0.367836
C -9.622785 14.43639 -1.431884
C -11.86758 14.442145 -2.364904
H -12.574674 14.745857 -3.133982
C -10.534527 14.840004 -2.413347
H -10.191807 15.471907 -3.233571
H -11.74953 12.532816 0.450913
C -8.188874 14.867177 -1.48285
H -7.54309 14.065176 -1.863232
H -7.809635 15.128489 -0.487352
H -8.046795 15.731713 -2.141147
H -12.847939 15.815981 0.137158
H -15.078352 16.427684 0.473677
C -14.897539 18.131015 1.801758
C -14.540268 19.449878 1.933279
C -13.858732 20.15227 0.881158
C -13.673565 19.42545 -0.388587
C -14.034172 18.059193 -0.496357
C -13.774654 17.381629 -1.707237
C -13.211732 18.042058 -2.785018
C -12.904891 19.409352 -2.694984
C -13.132611 20.083542 -1.507611
H -15.387327 17.620668 2.632832
H -14.740132 20.002366 2.849196
O -13.388927 21.310254 1.030443
H -12.950327 21.156679 -1.429952
H -12.503684 19.934864 -3.559324
H -13.013041 17.501879 -3.709548
H -13.980802 16.308259 -1.765588
K -16.714533 15.104936 -1.569668

K -10.874967 21.040772 0.684039

TS_A-B_para_2Kcation_acetonitrile_conf3
C -13.04973 15.941324 0.858621
C -14.829736 17.320242 0.720713
C -10.55713 17.548511 3.170786
C -9.875228 18.310712 2.256278
C -10.25412 18.395472 0.915718
C -11.306608 17.680827 0.413767
C -11.998474 16.810993 1.301841
C -11.662327 16.792865 2.703605
C -8.311124 19.36693 1.138653
H -10.254518 17.472314 4.21019
H -11.621312 17.798484 -0.619354
H -7.627144 18.544102 0.881326
O -8.806772 19.172752 2.468118
O -9.448435 19.347354 0.287235
H -7.810799 20.33682 1.070396
N -12.402764 16.017489 3.57946
S -11.667968 14.703242 4.167796
O -10.282307 14.931967 4.592778
O -12.62402 14.14726 5.154216
C -12.331632 14.628433 -2.631579
C -13.448884 13.823534 -2.834253
C -12.175195 15.33426 -1.443142
H -13.568878 13.27613 -3.766816
H -11.263023 15.90275 -1.28703
C -14.401045 13.700584 -1.819941
C -13.140103 15.254254 -0.422456
H -15.264098 13.051811 -1.955489
C -14.240162 14.397273 -0.633567
H -14.976993 14.28688 0.163389
H -11.565947 14.700857 -3.401411
C -10.554401 13.671316 1.882736
C -10.571935 12.892892 0.73084
C -11.607596 13.573147 2.792559
H -9.748509 12.966138 0.020099
C -11.627936 12.014685 0.463817

C -12.67372 12.709082 2.545794
H -13.482148 12.635915 3.27066
C -12.673711 11.935127 1.391924
H -13.501265 11.250979 1.202457
H -9.722703 14.34175 2.093928
C -11.627276 11.162443 -0.76713
H -11.134325 10.198731 -0.582064
H -11.091335 11.643264 -1.593076
H -12.644944 10.9409 -1.107715
H -13.518825 15.404175 1.681849
H -15.457426 16.527463 0.319806
C -14.943436 17.659119 2.090689
C -14.331504 18.770255 2.607397
C -13.628046 19.700537 1.765478
C -13.730109 19.485903 0.31387
C -14.334436 18.30832 -0.195523
C -14.348728 18.097202 -1.588627
C -13.800206 19.030622 -2.450078
C -13.229481 20.209728 -1.945984
C -13.197023 20.426302 -0.580487
H -15.465875 16.959401 2.746146
H -14.338962 18.993667 3.674383
O -12.9554 20.649405 2.242129
H -12.778699 21.345888 -0.171814
H -12.822766 20.951099 -2.630688
H -13.824336 18.856362 -3.523825
H -14.800072 17.185432 -1.980536
K -14.208209 16.149211 5.610506
K -10.587811 21.471331 2.069897

TS_A-B_para_2Kcation_acetonitrile_conf4

C -12.824269 17.090038 1.450991
C -14.633505 17.825442 1.906117
C -9.948309 19.459141 1.998843
C -9.717689 19.830744 0.697585
C -10.522079 19.395972 -0.361229
C -11.569674 18.541298 -0.16097
C -11.829979 18.103934 1.164612

C -11.048413 18.604188 2.261855
C -8.886382 20.652836 -1.199414
H -9.305545 19.784522 2.80978
H -12.139956 18.163982 -1.003775
H -8.01753 20.147009 -1.643729
O -8.749048 20.656065 0.222703
O -10.076061 19.947703 -1.529981
H -8.960533 21.686317 -1.556903
N -11.424199 18.286261 3.558122
S -10.369542 17.532041 4.514016
O -8.964997 17.921918 4.319382
O -10.932951 17.659684 5.879635
C -14.04155 15.264087 -1.670647
C -13.667633 13.95748 -1.358617
C -13.787625 16.296304 -0.775003
H -13.865685 13.151048 -2.061391
H -14.110293 17.309027 -1.020544
C -13.05612 13.690207 -0.137181
C -13.152456 16.04453 0.449382
H -12.773227 12.671494 0.122537
C -12.813591 14.72261 0.764375
H -12.340761 14.512931 1.723964
H -14.538126 15.478367 -2.614897
C -9.625839 15.284072 3.108573
C -9.759476 13.952535 2.726977
C -10.477897 15.804872 4.080464
H -9.090693 13.541568 1.971017
C -10.73434 13.127373 3.298445
C -11.465626 15.006665 4.656325
H -12.11248 15.431823 5.420917
C -11.586536 13.678641 4.265305
H -12.351199 13.049708 4.723302
H -8.852906 15.916944 2.67679
C -10.859602 11.689594 2.89633
H -11.905257 11.35955 2.884861
H -10.329655 11.031468 3.597562
H -10.433747 11.506859 1.903635
H -12.657127 16.670599 2.44517

H -15.144454 17.09605 1.279283
C -14.662175 19.171953 1.443044
C -14.551772 20.237398 2.299869
C -14.555819 20.064895 3.722528
C -14.747752 18.711949 4.231735
C -14.786083 17.614887 3.337599
C -14.936122 16.320752 3.863986
C -15.05692 16.116645 5.226522
C -15.043723 17.205658 6.111829
C -14.893965 18.485438 5.613968
H -14.659991 19.350451 0.366953
H -14.402829 21.246944 1.909197
O -14.410664 21.054116 4.520738
H -14.909959 19.347516 6.28219
H -15.157287 17.039837 7.180819
H -15.168615 15.105691 5.614415
H -14.945261 15.471454 3.179709
K -12.091342 20.003575 5.654696
K -16.330035 22.659753 3.909415

TS_A-B_para_2Kcation_acetonitrile_conf5
C -12.693359 16.735635 1.621584
C -14.682909 16.956944 2.509613
C -11.66164 13.476333 3.153635
C -10.625909 13.916048 3.946544
C -10.285785 15.267797 4.05659
C -10.948596 16.237675 3.357952
C -12.009701 15.832384 2.499535
C -12.391313 14.443189 2.430292
C -8.884639 14.08875 5.328746
H -11.912633 12.424576 3.066133
H -10.620383 17.269498 3.412332
H -7.867462 13.847895 4.993891
O -9.803775 13.17373 4.725103
O -9.230748 15.404594 4.913142
H -8.966026 14.011961 6.419965
N -13.526652 14.100684 1.701756
S -13.343211 13.108197 0.441345

O -12.398656 12.00714 0.650855
O -14.724329 12.778586 0.01286
C -11.493506 20.330501 1.94183
C -11.395541 20.684292 0.594744
C -11.93525 19.060838 2.30663
H -10.970205 21.647819 0.310067
H -12.006447 18.80758 3.361759
C -11.807516 19.775695 -0.385868
C -12.29044 18.110874 1.330927
H -11.728748 20.032477 -1.442524
C -12.270399 18.516283 -0.0173
H -12.572607 17.800604 -0.783634
H -11.192634 21.033477 2.717025
C -11.277549 14.425351 -0.791807
C -10.74481 15.357388 -1.673544
C -12.646705 14.154081 -0.824337
H -9.676539 15.571354 -1.647407
C -11.554738 16.022345 -2.602939
C -13.47414 14.806527 -1.735435
H -14.53622 14.570581 -1.755377
C -12.922647 15.726519 -2.622754
H -13.563931 16.221277 -3.352956
H -10.639869 13.898517 -0.083869
C -10.964736 17.019893 -3.552551
H -11.735076 17.651299 -4.009891
H -10.427005 16.525391 -4.371784
H -10.239598 17.673454 -3.052021
H -13.142003 16.224319 0.771499
H -14.666274 15.870704 2.570984
C -15.364466 17.560266 1.424731
C -15.647745 18.902215 1.41039
C -15.341892 19.754602 2.532049
C -14.790437 19.103159 3.726128
C -14.485663 17.717424 3.71311
C -13.921177 17.137503 4.866643
C -13.668601 17.904024 5.99112
C -13.980455 19.272249 6.001204
C -14.535087 19.861197 4.878752

H -15.58649 16.947115 0.5469
H -16.150831 19.355152 0.553982
O -15.508897 21.00186 2.483335
H -14.788328 20.919214 4.859146
H -13.788113 19.865187 6.892899
H -13.230307 17.442024 6.873716
H -13.673087 16.075325 4.858142
K -16.143619 13.292858 2.121945
K -14.279836 21.928917 0.474804

TS_A-B_para_2Kcation_acetonitrile_conf6
C -12.931358 16.795573 1.54287
C -14.80488 17.450507 2.050299
C -11.120933 18.990953 -0.900568
C -11.181907 18.187178 -2.016146
C -11.883543 16.979912 -2.034059
C -12.520822 16.504242 -0.921025
C -12.463779 17.28641 0.262023
C -11.809083 18.564951 0.25923
C -10.945343 17.26965 -4.034754
H -10.553118 19.916217 -0.892881
H -12.996862 15.529234 -0.934464
H -10.015477 16.744601 -4.292709
O -10.628476 18.412656 -3.234367
O -11.789099 16.414128 -3.276034
H -11.470558 17.59915 -4.93953
N -11.941473 19.377279 1.380007
S -10.634325 19.90065 2.147939
O -9.552403 20.375795 1.262965
O -11.147923 20.879927 3.136929
C -13.936916 13.122972 1.456198
C -13.109761 12.611411 2.456308
C -13.902893 14.477061 1.147147
H -13.137382 11.550802 2.696906
H -14.575366 14.871329 0.383905
C -12.261515 13.466924 3.153516
C -13.038765 15.348369 1.827745
H -11.622847 13.077395 3.944419

C -12.234578 14.825315 2.850288
H -11.576099 15.498613 3.400211
H -14.615489 12.463151 0.919468
C -9.095264 17.641689 2.449878
C -8.684202 16.500861 3.129733
C -9.999621 18.510515 3.059634
H -7.975444 15.821241 2.65735
C -9.15929 16.209973 4.414376
C -10.500433 18.236044 4.330952
H -11.209456 18.925841 4.784818
C -10.073014 17.094956 5.00032
H -10.455702 16.882422 5.999605
H -8.710234 17.874029 1.458715
C -8.693854 14.987271 5.143952
H -9.408984 14.675552 5.913583
H -7.73614 15.16514 5.650721
H -8.537341 14.143309 4.462328
H -12.451373 17.344026 2.353376
H -15.25555 16.461488 1.985839
C -15.208957 18.386866 1.053992
C -15.172101 19.738398 1.258922
C -14.781072 20.293547 2.519422
C -14.449746 19.359101 3.593859
C -14.570873 17.961429 3.386925
C -14.35517 17.097292 4.477181
C -13.993192 17.591773 5.717862
C -13.795801 18.97022 5.902499
C -14.019499 19.837161 4.847848
H -15.506418 18.000986 0.078176
H -15.458231 20.433636 0.469192
O -14.76521 21.553552 2.719304
H -13.7785 20.897909 4.937625
H -13.434826 19.345655 6.858402
H -13.829164 16.90549 6.546524
H -14.458905 16.023137 4.325246
K -12.553 22.161877 1.250822
K -16.452349 21.761008 4.712289

TS_A-B_para_2Kcation_acetonitrile_conf7

C -12.879526 16.403878 0.44009
C -14.649678 17.444687 0.692273
C -11.603346 17.302764 -2.981485
C -12.17875 16.28846 -3.709809
C -12.964173 15.296012 -3.114855
C -13.208983 15.285377 -1.770005
C -12.647986 16.325203 -0.978903
C -11.835937 17.34384 -1.588041
C -12.792437 14.840071 -5.289772
H -10.961288 18.047853 -3.443041
H -13.78927 14.486057 -1.319663
H -12.080893 14.079606 -5.638923
O -12.087955 16.060593 -5.043366
O -13.390479 14.417982 -4.070993
H -13.571365 15.019666 -6.040395
N -11.275833 18.392317 -0.86069
S -9.892263 18.105453 -0.095916
O -9.472306 19.416378 0.458271
O -9.939623 16.967752 0.839484
C -14.239146 13.212806 2.034223
C -13.261228 12.987011 3.000433
C -14.146407 14.313291 1.188559
H -13.333884 12.123705 3.658657
H -14.934842 14.482119 0.455082
C -12.19625 13.876202 3.122278
C -13.074879 15.212138 1.294986
H -11.427322 13.707624 3.873742
C -12.108269 14.985388 2.287971
H -11.267734 15.673439 2.363943
H -15.081662 12.530723 1.939076
C -7.969766 18.63376 -2.005452
C -7.119339 18.271123 -3.042875
C -8.692973 17.650311 -1.333947
H -6.541744 19.037995 -3.559041
C -6.98359 16.933765 -3.434151
C -8.574091 16.311652 -1.704676
H -9.137624 15.552774 -1.16471

C -7.725232 15.964967 -2.74953
H -7.6287 14.918346 -3.037796
H -8.060048 19.672695 -1.694218
C -6.04298 16.546785 -4.533715
H -5.043569 16.320691 -4.138723
H -5.920597 17.350805 -5.267549
H -6.384087 15.651471 -5.064767
H -12.24989 17.15505 0.908503
H -15.243364 16.567905 0.435386
C -14.513619 17.771085 2.067059
C -14.156115 19.033424 2.483749
C -14.019073 20.118865 1.567637
C -14.397882 19.858836 0.179213
C -14.696696 18.541003 -0.248962
C -15.003978 18.328584 -1.605048
C -15.025595 19.378305 -2.505177
C -14.763892 20.687398 -2.073738
C -14.458861 20.918957 -0.744863
H -14.579246 16.962715 2.79538
H -13.882362 19.196713 3.528775
O -13.569349 21.264873 1.914165
H -14.286907 21.93183 -0.377809
H -14.821569 21.514792 -2.77828
H -15.262255 19.190183 -3.550494
H -15.220498 17.315408 -1.942284
K -11.455082 21.004026 -0.042307
K -14.441244 22.059669 4.187785

TS_A-B_para_2Kcation_acetonitrile_conf8

C -12.549042 16.533842 0.922731
C -14.69173 17.20485 0.712659
C -13.122277 12.946921 1.823829
C -12.612053 12.946581 3.103193
C -12.108409 14.103885 3.706418
C -12.084206 15.304257 3.054032
C -12.590755 15.352924 1.724296
C -13.129974 14.163301 1.111821
C -11.885981 12.412655 5.142776

H -13.503512 12.041922 1.358392
H -11.635185 16.174057 3.52204
H -10.929097 11.899653 5.300901
O -12.498944 11.901081 3.954012
O -11.662324 13.805335 4.961015
H -12.562142 12.253228 5.991703
N -13.754681 14.208078 -0.134621
S -12.881289 13.983556 -1.465278
O -13.874009 14.027168 -2.569726
O -11.715582 14.876562 -1.574886
C -11.033572 19.546246 2.675237
C -10.17992 20.006941 1.670069
C -11.850695 18.440514 2.451405
H -9.486042 20.823388 1.872546
H -12.513365 18.096048 3.241581
C -10.197891 19.390827 0.413784
C -11.825032 17.763241 1.217785
H -9.530712 19.734405 -0.375976
C -11.027706 18.297912 0.185179
H -11.018948 17.792566 -0.780761
H -11.039231 20.028052 3.651618
C -13.000299 11.255438 -1.850664
C -12.527684 9.959398 -1.690482
C -12.229824 12.327839 -1.403649
H -13.121769 9.121004 -2.053706
C -11.29361 9.708913 -1.076796
C -10.998083 12.104881 -0.791245
H -10.402854 12.952376 -0.457106
C -10.54166 10.801108 -0.631687
H -9.576846 10.625027 -0.156863
H -13.951096 11.444933 -2.345222
C -10.780467 8.309493 -0.932775
H -10.275835 7.977403 -1.84966
H -11.5898 7.59624 -0.741526
H -10.05331 8.224359 -0.118536
H -12.61322 16.322564 -0.139361
H -14.832446 16.143215 0.51997
C -15.050319 17.743647 1.967106

C -15.136968 19.098664 2.180818
C -14.903586 20.041592 1.12301
C -14.692777 19.483355 -0.225183
C -14.575881 18.083466 -0.417411
C -14.278866 17.599922 -1.710423
C -14.152733 18.463908 -2.783161
C -14.333384 19.844998 -2.602888
C -14.593506 20.340041 -1.336613
H -15.203488 17.056769 2.801205
H -15.35929 19.503165 3.166222
O -14.820756 21.282283 1.320566
H -14.781625 21.403484 -1.178086
H -14.277401 20.516993 -3.457068
H -13.91432 18.068979 -3.76873
H -14.095127 16.531535 -1.852648
K -16.169475 14.488196 -1.44158
K -12.437043 21.825001 0.543169

TS_A-B_para_2Kcation_acetonitrile_conf9
C -12.905536 16.277173 0.45406
C -14.903255 17.265477 0.630433
C -10.620469 18.186701 2.766263
C -10.229869 19.188197 1.913319
C -10.721038 19.291608 0.614008
C -11.605643 18.38324 0.098632
C -11.991167 17.286684 0.918113
C -11.5203 17.207131 2.275709
C -9.052052 20.679166 0.822435
H -10.223426 18.089726 3.773222
H -12.031659 18.52285 -0.890552
H -8.219657 20.065548 0.445114
O -9.379807 20.261795 2.153974
O -10.217484 20.46351 0.043016
H -8.79383 21.742023 0.822574
N -11.905537 16.197746 3.147632
S -11.20535 14.753058 2.979428
O -11.76028 13.939996 4.088135
O -11.270478 14.201915 1.619307

C -12.387011 15.549247 -3.238131
C -13.33246 14.546599 -3.429795
C -12.218282 16.131472 -1.986097
H -13.461694 14.095532 -4.411236
H -11.430621 16.866709 -1.85404
C -14.093044 14.101124 -2.346626
C -12.999963 15.724303 -0.890403
H -14.811321 13.294738 -2.477442
C -13.920645 14.676956 -1.099475
H -14.503576 14.316302 -0.25133
H -11.762262 15.874116 -4.067503
C -9.000526 14.952508 4.632767
C -7.66859 15.249725 4.891904
C -9.473831 15.002834 3.322814
H -7.293109 15.19246 5.913582
C -6.792397 15.609378 3.859958
C -8.621816 15.358082 2.278757
H -9.001433 15.375897 1.258475
C -7.292604 15.660112 2.554733
H -6.62203 15.927761 1.738198
H -9.671943 14.651801 5.434805
C -5.35002 15.894469 4.145567
H -4.770225 14.964902 4.217787
H -5.21863 16.422029 5.09703
H -4.889537 16.497721 3.356033
H -13.188237 15.571943 1.228932
H -15.398214 16.404304 0.187142
C -14.943543 17.427286 2.037508
C -14.53223 18.586071 2.639915
C -14.139394 19.733014 1.866813
C -14.339069 19.652424 0.412107
C -14.728416 18.429806 -0.1914
C -14.843737 18.36805 -1.594543
C -14.597103 19.484902 -2.373043
C -14.23909 20.701921 -1.772303
C -14.113066 20.775938 -0.397296
H -15.238304 16.565497 2.640188
H -14.475597 18.687863 3.723474

O -13.646086 20.757433 2.403638
H -13.861751 21.718128 0.089602
H -14.071349 21.582491 -2.38906
H -14.695417 19.422917 -3.454906
H -15.131186 17.425289 -2.06031
K -13.533047 15.484856 5.179024
K -11.599586 22.171917 2.131715

TS_A-B_para_2Kcation_acetonitrile_conf10
C -12.861741 16.350686 0.4685
C -14.626112 17.392492 0.755711
C -11.612067 17.315985 -2.944863
C -12.195612 16.317504 -3.688523
C -12.978151 15.314889 -3.106987
C -13.211914 15.277874 -1.760676
C -12.641965 16.300447 -0.953635
C -11.832723 17.329382 -1.548938
C -12.822572 14.90024 -5.291217
H -10.972399 18.068948 -3.396882
H -13.789865 14.47072 -1.321464
H -12.113048 14.145798 -5.657316
O -12.116286 16.116031 -5.027091
O -13.414421 14.45711 -4.076929
H -13.605311 15.09317 -6.034436
N -11.263276 18.360881 -0.80459
S -9.873186 18.0522 -0.060039
O -9.445 19.347988 0.522602
O -9.914483 16.891344 0.846697
C -14.223951 13.132641 2.004413
C -13.238833 12.880309 2.956622
C -14.13249 14.251116 1.182631
H -13.310296 12.003037 3.596239
H -14.926203 14.440219 0.459873
C -12.168018 13.761023 3.088716
C -13.05526 15.141834 1.299434
H -11.393502 13.571801 3.829458
C -12.081208 14.888089 2.278518
H -11.236294 15.569882 2.362293

H -15.070922 12.457222 1.901627
C -7.973022 18.626659 -1.978385
C -7.134267 18.289513 -3.033604
C -8.686315 17.62669 -1.320616
H -6.564563 19.069195 -3.539275
C -7.000255 16.96148 -3.456325
C -8.568873 16.297062 -1.72252
H -9.124597 15.525121 -1.193115
C -7.731498 15.975899 -2.784814
H -7.636069 14.936216 -3.097498
H -8.061823 19.658226 -1.643098
C -6.071955 16.602035 -4.575516
H -5.062708 16.387434 -4.199574
H -5.974058 17.416462 -5.30159
H -6.407861 15.70783 -5.111622
H -12.225756 17.089783 0.947338
H -15.224825 16.523409 0.484819
C -14.479391 17.689545 2.136126
C -14.1148 18.941755 2.576704
C -13.980016 20.045276 1.682193
C -14.369197 19.815703 0.291341
C -14.675904 18.508268 -0.162157
C -14.993962 18.325433 -1.520082
C -15.018375 19.393944 -2.397788
C -14.748644 20.692747 -1.940903
C -14.432849 20.895183 -0.609787
H -14.543136 16.866308 2.847779
H -13.834119 19.083149 3.623103
O -13.523966 21.182381 2.049396
H -14.254457 21.899522 -0.222971
H -14.808511 21.534961 -2.627472
H -15.263557 19.228641 -3.445002
H -15.217148 17.320435 -1.876841
K -11.423843 20.954378 0.074425
K -14.389967 21.945286 4.334299

TS_A-B_para_2Kcation_acetonitrile_conf11

C -12.533999 16.465947 0.953293

C -14.674347 17.154455 0.767767
C -13.123859 12.865124 1.785557
C -12.608097 12.836631 3.062386
C -12.09523 13.978958 3.686128
C -12.067045 15.191839 3.057491
C -12.579108 15.269597 1.731266
C -13.127831 14.095412 1.097717
C -11.878233 12.258863 5.088635
H -13.512629 11.971659 1.304371
H -11.611244 16.049774 3.540628
H -10.92468 11.736399 5.235149
O -12.497117 11.773873 3.891888
O -11.645172 13.653116 4.932429
H -12.553849 12.088267 5.935877
N -13.757998 14.168454 -0.144614
S -12.892208 13.967171 -1.483992
O -13.890371 14.036464 -2.582189
O -11.723736 14.857972 -1.582084
C -10.990352 19.433015 2.757973
C -10.136996 19.90681 1.758698
C -11.816362 18.337742 2.515972
H -9.436369 20.714066 1.974343
H -12.478517 17.982835 3.301938
C -10.164107 19.315247 0.490808
C -11.800431 17.684456 1.269318
H -9.497182 19.669103 -0.294614
C -11.002989 18.23321 0.244198
H -11.00179 17.746877 -0.731472
H -10.988844 19.89577 3.743538
C -13.022422 11.247739 -1.923787
C -12.552235 9.947417 -1.794007
C -12.24617 12.308466 -1.459301
H -13.150774 9.118396 -2.171142
C -11.31488 9.680948 -1.193817
C -11.011235 12.069525 -0.859491
H -10.411565 12.908267 -0.511536
C -10.557276 10.761539 -0.730394
H -9.589914 10.573014 -0.265651

H -13.975783 11.45009 -2.4082
C -10.804411 8.277513 -1.083237
H -10.306825 7.963934 -2.010438
H -11.614311 7.562329 -0.90186
H -10.072015 8.173145 -0.275969
H -12.605358 16.276483 -0.112446
H -14.822628 16.097417 0.556238
C -15.022344 17.672164 2.033945
C -15.098378 19.023564 2.273283
C -14.864208 19.984439 1.231896
C -14.665341 19.45001 -0.127692
C -14.55877 18.053138 -0.346431
C -14.2721 17.591827 -1.649843
C -14.146136 18.474702 -2.707067
C -14.317107 19.853326 -2.500335
C -14.567056 20.326473 -1.223698
H -15.175795 16.970939 2.855972
H -15.312505 19.411133 3.267286
O -14.771086 21.220601 1.452069
H -14.748012 21.387845 -1.044643
H -14.261687 20.540639 -3.342275
H -13.915487 18.096578 -3.701041
H -14.096007 16.525185 -1.812859
K -16.17761 14.486429 -1.43389
K -12.386164 21.758878 0.674292

TS_A-B_para_2Kcation_acetonitrile_conf12
C -12.885438 16.225785 0.477054
C -14.881555 17.213147 0.677319
C -10.589369 18.081622 2.821694
C -10.20067 19.101116 1.989378
C -10.69716 19.233968 0.694752
C -11.584413 18.337788 0.162747
C -11.968385 17.224154 0.959613
C -11.492742 17.11452 2.313382
C -9.02658 20.615678 0.926886
H -10.188472 17.961847 3.824664
H -12.014034 18.499626 -0.821519

H -8.195904 20.010674 0.532251
O -9.348331 20.168164 2.250366
O -10.195341 20.417905 0.148013
H -8.768108 21.678213 0.949937
N -11.876464 16.086833 3.164464
S -11.177486 14.645906 2.962523
O -11.730952 13.808444 4.053679
O -11.245693 14.12562 1.590482
C -12.375994 15.573894 -3.23035
C -13.32259 14.576002 -3.440455
C -12.204003 16.130069 -1.966963
H -13.454344 14.145285 -4.430661
H -11.415749 16.862174 -1.821363
C -14.081201 14.108897 -2.365034
C -12.98354 15.700854 -0.878228
H -14.800505 13.306006 -2.510874
C -13.905557 14.658761 -1.106664
H -14.487034 14.281346 -0.264814
H -11.752933 15.915332 -4.054305
C -8.969918 14.807211 4.616164
C -7.637009 15.096393 4.879418
C -9.445081 14.885948 3.308292
H -7.260072 15.017035 5.89908
C -6.761689 15.47587 3.853876
C -8.594028 15.261563 2.270596
H -8.975295 15.301519 1.251553
C -7.263813 15.555098 2.550767
H -6.593958 15.838505 1.738972
H -9.640714 14.490982 5.41274
C -5.318175 15.751572 4.142927
H -4.739387 14.819678 4.188396
H -5.183449 16.253214 5.107826
H -4.859121 16.375193 3.368511
H -13.167214 15.504962 1.237797
H -15.3787 16.362788 0.215943
C -14.919961 17.344456 2.087665
C -14.505328 18.488967 2.714603
C -14.110461 19.651355 1.965963

C -14.31219 19.602914 0.510191
C -14.705275 18.394709 -0.119211
C -14.821834 18.363578 -1.523272
C -14.572821 19.496372 -2.277616
C -14.210912 20.699124 -1.651
C -14.083599 20.74307 -0.274825
H -15.216286 16.470569 2.671928
H -14.447508 18.567174 3.800054
O -13.613503 20.662483 2.524196
H -13.828351 21.673492 0.232168
H -14.040838 21.592296 -2.248709
H -14.672114 19.458064 -3.360485
H -15.111938 17.43183 -2.009105
K -13.504109 15.324733 5.178309
K -11.564814 22.079754 2.297323

TS_A-B_para_2Kcation_acetonitrile_conf13
C 1.471219 0.211626 0.137531
C 2.732581 -0.380499 -1.338384
C -1.723057 1.606156 -1.295842
C -1.373397 2.933731 -1.388048
C -0.120526 3.399017 -0.985307
C 0.828874 2.554984 -0.477376
C 0.516138 1.17424 -0.375839
C -0.770569 0.691383 -0.793027
C -1.329188 5.145765 -1.657722
H -2.713137 1.25417 -1.572731
H 1.781936 2.946399 -0.138441
H -1.8157 5.795342 -0.917278
O -2.123207 3.969859 -1.840555
O -0.04846 4.749197 -1.183439
H -1.222861 5.661596 -2.619544
N -1.12225 -0.659969 -0.752841
S -1.665466 -1.241345 0.641939
O -2.07317 -2.637742 0.34941
O -0.753525 -1.029136 1.779933
C 4.273574 1.719408 2.243945
C 4.139247 0.96426 3.408552

C 3.419011 1.503751 1.168988
H 4.808676 1.134758 4.249215
H 3.571098 2.077085 0.254959
C 3.150274 -0.012403 3.484669
C 2.412482 0.527853 1.234514
H 3.03901 -0.608829 4.388025
C 2.301847 -0.23877 2.405149
H 1.517231 -0.99115 2.463075
H 5.052189 2.475992 2.170071
C -4.383245 -0.763873 0.558533
C -5.514836 0.008712 0.78769
C -3.14944 -0.339884 1.048665
H -6.482612 -0.330132 0.417719
C -5.437118 1.214732 1.495086
C -3.047223 0.856365 1.756899
H -2.079026 1.17123 2.142264
C -4.187174 1.622915 1.972035
H -4.108962 2.556575 2.528761
H -4.45116 -1.709278 0.023738
C -6.666831 2.027136 1.762304
H -6.429685 3.084772 1.919355
H -7.184013 1.677646 2.665806
H -7.386946 1.959449 0.93928
H 1.008374 -0.764765 0.26388
H 3.415699 0.460475 -1.218599
C 1.921416 -0.373342 -2.509639
C 1.383957 -1.515524 -3.03949
C 1.646315 -2.80098 -2.472375
C 2.572465 -2.855572 -1.343382
C 3.193636 -1.670549 -0.86314
C 4.177175 -1.788863 0.137976
C 4.497504 -3.018745 0.68348
C 3.823226 -4.178884 0.2686
C 2.877264 -4.091752 -0.73478
H 1.664805 0.590724 -2.948862
H 0.732464 -1.470056 -3.91297
O 1.110412 -3.86322 -2.940547
H 2.290521 -4.965792 -1.024024

H 4.029485 -5.131339 0.752818
H 5.253868 -3.081203 1.462967
H 4.675103 -0.889534 0.497602
K -1.403432 -3.01543 -2.12816
K 3.240337 -5.20956 -3.655063

TS_A-B_para_2Kcation_acetonitrile_conf14
C 1.260866 -0.230059 0.714461
C 2.852785 -0.123313 -0.812274
C 3.016064 1.640464 3.443333
C 3.131002 0.636974 4.380494
C 2.6902 -0.665179 4.133662
C 2.101732 -1.0167 2.949696
C 1.944488 -0.010029 1.957207
C 2.429223 1.325241 2.201325
C 3.506775 -0.58523 6.207084
H 3.357685 2.653027 3.641452
H 1.708369 -2.018104 2.813131
H 2.852742 -0.51823 7.085938
O 3.647799 0.716977 5.629775
O 2.920999 -1.441295 5.233834
H 4.498273 -0.963894 6.484782
N 2.446475 2.282334 1.187022
S 1.187116 3.252536 0.962702
O 1.609736 4.158938 -0.134381
O -0.096379 2.548637 0.797258
C -0.028306 -3.791323 0.357552
C -1.311227 -3.535952 -0.137937
C 0.854196 -2.742516 0.59795
H -2.029589 -4.34937 -0.248855
H 1.853509 -2.962135 0.966855
C -1.677399 -2.225354 -0.454722
C 0.469091 -1.407314 0.367336
H -2.671923 -2.011122 -0.842804
C -0.790239 -1.178305 -0.219514
H -1.089355 -0.150925 -0.426508
H 0.274835 -4.810215 0.597372
C 1.7274 5.430772 2.569138

C 1.663387 6.134955 3.76492
C 1.011212 4.242916 2.432346
H 2.209696 7.072444 3.867461
C 0.899696 5.666906 4.841448
C 0.242956 3.757047 3.48895
H -0.320658 2.834626 3.363606
C 0.194752 4.469091 4.682021
H -0.408856 4.092684 5.507491
H 2.30567 5.809554 1.728543
C 0.813532 6.447473 6.116593
H 0.585336 5.804291 6.973055
H 0.019169 7.203761 6.064051
H 1.745587 6.981562 6.331385
H 0.847577 0.685528 0.307993
H 3.3839 0.517588 -0.112692
C 2.091971 0.484811 -1.84581
C 1.622883 -0.226356 -2.919117
C 1.937298 -1.619175 -3.087972
C 2.861292 -2.217625 -2.109071
C 3.328348 -1.463691 -1.001702
C 4.206482 -2.076211 -0.084274
C 4.612124 -3.387302 -0.257598
C 4.164244 -4.126353 -1.364614
C 3.302511 -3.54154 -2.275543
H 1.776099 1.524223 -1.7099
H 0.978353 0.232599 -3.66585
O 1.426117 -2.316778 -4.002805
H 2.988927 -4.076189 -3.173021
H 4.514416 -5.145763 -1.513985
H 5.294193 -3.843245 0.457391
H 4.556052 -1.500883 0.774138
K 3.99153 3.379517 -0.788442
K -0.112485 -4.025908 -2.953873

TS_A-B_para_2Kcation_acetonitrile_conf15
C 1.473133 0.267021 0.227279
C 2.804903 -0.422851 -1.468001
C -0.032823 -2.942074 1.508868

C -1.175446 -2.97204 0.752611
C -1.497477 -1.955516 -0.144744
C -0.676228 -0.882989 -0.353933
C 0.529367 -0.800413 0.398428
C 0.827938 -1.82521 1.37226
C -3.276806 -3.177311 0.174305
H 0.181911 -3.689975 2.266866
H -0.958205 -0.104241 -1.052877
H -3.776013 -2.613905 0.97765
O -2.186528 -3.92285 0.72845
O -2.704061 -2.284512 -0.769685
H -3.971652 -3.856722 -0.32735
N 1.899631 -1.753329 2.246989
S 1.756514 -0.719658 3.480624
O 2.942232 -0.974289 4.330113
O 1.478795 0.670711 3.091266
C 0.197361 3.423356 -1.491428
C 0.80968 4.390673 -0.695941
C 0.391669 2.073288 -1.230546
H 0.653237 5.447126 -0.902973
H -0.061656 1.345473 -1.898881
C 1.631371 3.994299 0.355793
C 1.207131 1.652575 -0.164664
H 2.116317 4.739423 0.982713
C 1.837402 2.644565 0.610601
H 2.45226 2.33428 1.45302
H -0.429772 3.723246 -2.328509
C 0.437331 -2.235292 5.377508
C -0.705212 -2.723041 5.999103
C 0.319828 -1.257487 4.391679
H -0.612498 -3.473794 6.783836
C -1.977772 -2.257187 5.644185
C -0.934832 -0.776568 4.022062
H -1.01052 -0.000755 3.261528
C -2.070706 -1.281187 4.646727
H -3.05234 -0.897779 4.367436
H 1.425336 -2.585469 5.670561
C -3.200397 -2.767927 6.342689

H -4.113016 -2.552698 5.77699
H -3.316377 -2.301242 7.329569
H -3.152323 -3.849916 6.511195
H 2.314118 0.179956 0.909578
H 3.360255 0.51365 -1.47862
C 1.806261 -0.63569 -2.445513
C 1.184929 -1.851252 -2.572942
C 1.574451 -2.985755 -1.779343
C 2.829208 -2.848144 -1.026299
C 3.450572 -1.579356 -0.905662
C 4.715554 -1.499941 -0.28848
C 5.364427 -2.644676 0.142136
C 4.730653 -3.898771 0.047412
C 3.465789 -3.987639 -0.5109
H 1.478258 0.209428 -3.048814
H 0.359399 -1.985864 -3.272805
O 0.917948 -4.058861 -1.766742
H 2.96226 -4.946104 -0.62271
H 5.255422 -4.801871 0.359791
H 6.383971 -2.576083 0.524725
H 5.207989 -0.529927 -0.218267
K 4.271665 -2.786737 3.029885
K -1.487749 -4.730567 -2.020122

Table S13. Computed energies for the neutral transition state conformational analysis in acetonitrile.

	M06-L/BS1							TPSS0-D4/BS2
	1st frequency (cm-1)	Electronic energy	ZPE corr	Thermal corr	Enthalpy corr:	Gibbs corr	Electronic energy in acetonitrile	Electronic energy
pre-ortho TS								
TS_A-B_ortho_2Kcation_MicroSolvation_acetonitrile	-351.6	-3408.67877	0.94897	1.03688	1.03783	0.80059	-3408.78471	-4553.27130
TS_A-B_ortho_2Kcation_MicroSolvation_acetonitrile_conf2	-312.1	-3408.71295	0.95054	1.03674	1.03769	0.81683	-3408.80871	-4553.28563
TS_A-B_ortho_2Kcation_MicroSolvation_acetonitrile_conf3	-387.8	-3408.69899	0.94933	1.03607	1.03701	0.81192	-3408.80666	-4553.26874
TS_A-B_ortho_2Kcation_MicroSolvation_acetonitrile_conf4	-361.8	-3408.68694	0.95025	1.03701	1.03795	0.81033	-3408.78414	-4553.26759
TS_A-B_ortho_2Kcation_MicroSolvation_acetonitrile_conf5	-359.8	-3408.70095	0.95021	1.03669	1.03763	0.81443	-3408.80021	-4553.28089
TS_A-B_ortho_2Kcation_MicroSolvation_acetonitrile_conf6	-389.8	-3408.70101	0.95156	1.03691	1.03786	0.82075	-3408.79997	-4553.27265
TS_A-B_ortho_2Kcation_MicroSolvation_acetonitrile_conf7	-338.9	-3408.68086	0.94994	1.03672	1.03766	0.81147	-3408.77989	-4553.26091
TS_A-B_ortho_2Kcation_MicroSolvation_acetonitrile_conf8	-334.3	-3408.69226	0.94965	1.03672	1.03766	0.80983	-3408.79662	-4553.27293
TS_A-B_ortho_2Kcation_MicroSolvation_acetonitrile_conf9	-300.0	-3408.70295	0.95081	1.03750	1.03844	0.81311	-3408.80705	-4553.28196
TS_A-B_ortho_2Kcation_MicroSolvation_acetonitrile_conf10	-327.8	-3408.70127	0.94996	1.03683	1.03778	0.81337	-3408.80075	-4553.27733
TS_A-B_ortho_2Kcation_MicroSolvation_acetonitrile_conf11	-308.4	-3408.71151	0.95022	1.03707	1.03802	0.81314	-3408.80928	-4553.28534
TS_A-B_ortho_2Kcation_MicroSolvation_acetonitrile_conf12	-265.2	-3408.70411	0.95027	1.03684	1.03778	0.81429	-3408.79889	-4553.28057
TS_A-B_ortho_2Kcation_MicroSolvation_acetonitrile_conf13	-367.4	-3408.69759	0.94986	1.03644	1.03738	0.81323	-3408.79290	-4553.27477
TS_A-B_ortho_2Kcation_MicroSolvation_acetonitrile_conf14	-353.5	-3408.69708	0.95058	1.03736	1.03830	0.81215	-3408.79634	-4553.27578
TS_A-B_ortho_2Kcation_MicroSolvation_acetonitrile_conf15	-344.4	-3408.69399	0.95043	1.03701	1.03795	0.81157	-3408.79274	-4553.27778
TS_A-B_ortho_2Kcation_MicroSolvation_acetonitrile_conf16	-312.7	-3408.70986	0.95096	1.03730	1.03825	0.81631	-3408.81045	-4553.28353
TS_A-B_ortho_2Kcation_MicroSolvation_acetonitrile_conf17	-344.1	-3408.69624	0.95029	1.03715	1.03810	0.81181	-3408.79369	-4553.27654
TS_A-B_ortho_2Kcation_MicroSolvation_acetonitrile_conf18	-351.3	-3408.69575	0.95019	1.03709	1.03804	0.80980	-3408.80056	-4553.27397
pre-para TS								
TS_A-B_para_2Kcation_MicroSolvation_acetonitrile	-261.4	-3408.67752	0.94747	1.03639	1.03734	0.79660	-3408.78567	-4553.26597
TS_A-B_para_2Kcation_MicroSolvation_acetonitrile_conf1	-203.2	-3408.68688	0.94951	1.03653	1.03748	0.80989	-3408.78840	-4553.26659
TS_A-B_para_2Kcation_MicroSolvation_acetonitrile_conf2	-208.8	-3408.68725	0.94902	1.03645	1.03739	0.80872	-3408.78551	-4553.26693
TS_A-B_para_2Kcation_MicroSolvation_acetonitrile_conf3	-189.3	-3408.67160	0.94808	1.03638	1.03733	0.79924	-3408.77982	-4553.26109
TS_A-B_para_2Kcation_MicroSolvation_acetonitrile_conf4	-363.9	-3408.69832	0.95054	1.03719	1.03813	0.81095	-3408.79712	-4553.28366
TS_A-B_para_2Kcation_MicroSolvation_acetonitrile_conf5	-229.1	-3408.68883	0.94958	1.03632	1.03726	0.81267	-3408.78845	-4553.26519
TS_A-B_para_2Kcation_MicroSolvation_acetonitrile_conf6	-137.4	-3408.67491	0.94810	1.03626	1.03721	0.79948	-3408.78664	-4553.26003
TS_A-B_para_2Kcation_MicroSolvation_acetonitrile_conf7	-335.3	-3408.68691	0.94951	1.03678	1.03772	0.80518	-3408.78961	-4553.27585
TS_A-B_para_2Kcation_MicroSolvation_acetonitrile_conf8	-361.0	-3408.67942	0.95010	1.03678	1.03773	0.81231	-3408.78115	-4553.26035
TS_A-B_para_2Kcation_MicroSolvation_acetonitrile_conf9	-331.6	-3408.69134	0.95005	1.03644	1.03738	0.81216	-3408.78676	-4553.27389
TS_A-B_para_2Kcation_MicroSolvation_acetonitrile_conf10	-249.9	-3408.69735	0.95068	1.03698	1.03793	0.81560	-3408.79456	-4553.27273
TS_A-B_para_2Kcation_MicroSolvation_acetonitrile_conf11	-275.5	-3408.69297	0.94919	1.03660	1.03754	0.80744	-3408.79406	-4553.27697

TS_A-B_para_2Kcation_MicroSolvation_acetonitrile_conf12	-346.8	-3408.69884	0.95042	1.03697	1.03791	0.81158	-3408.79343	-4553.28716
TS_A-B_para_2Kcation_MicroSolvation_acetonitrile_conf13	-310.2	-3408.69313	0.94918	1.03614	1.03708	0.81224	-3408.79430	-4553.26864
TS_A-B_para_2Kcation_MicroSolvation_acetonitrile_conf14	-337.5	-3408.69226	0.95037	1.03663	1.03758	0.81583	-3408.78960	-4553.26991
TS_A-B_para_2Kcation_MicroSolvation_acetonitrile_conf15	-342.2	-3408.68898	0.94977	1.03710	1.03805	0.80571	-3408.79365	-4553.27644
TS_A-B_para_2Kcation_MicroSolvation_acetonitrile_conf16	-210.5	-3408.69541	0.94973	1.03664	1.03758	0.81034	-3408.79482	-4553.27619
TS_A-B_para_2Kcation_MicroSolvation_acetonitrile_conf17	-339.2	-3408.69019	0.95017	1.03668	1.03762	0.81433	-3408.78821	-4553.27244
TS_A-B_para_2Kcation_MicroSolvation_acetonitrile_conf18	-316.3	-3408.69251	0.95040	1.03697	1.03791	0.81288	-3408.79242	-4553.27150
TS_A-B_para_2Kcation_MicroSolvation_acetonitrile_conf19	-308.9	-3408.68461	0.94899	1.03666	1.03761	0.80295	-3408.78966	-4553.26871

Table S14. Computed energies for the neutral path in acetonitrile.

	M06-L/BS1							TPSS0-D4/BS2
	1st frequency (cm-1)	Electronic energy	ZPE corr	Thermal corr	Enthalpy corr:	Gibbs corr	Electronic energy in acetonitrile	Electronic energy
Path for ortho product								
InterA_pre-ortho_2Kcation_MicroSolvation_acetonitrile	9.3	-3408.70140	0.94983	1.03752	1.03847	0.80626	-3408.80178	**-4553.28308**
TS_A-B_ortho_2Kcation_MicroSolvation_acetonitrile	-351.6	-3408.67877	0.94897	1.03688	1.03783	0.80059	-3408.78471	-4553.27130
InterB_ortho_2Kcation_MicroSolvation_acetonitrile	13.0	-3408.70547	0.95237	1.03893	1.03987	0.81563	-3408.80531	-4553.29457
Path for para product								
InterA_pre-para_2Kcation_MicroSolvation_acetonitrile	6.0	-3408.67939	0.94719	1.03711	1.03805	0.79279	-3408.78724	**-4553.26410**
TS_A-B_para_2Kcation_MicroSolvation_acetonitrile	-261.4	-3408.67752	0.94747	1.03639	1.03734	0.79660	-3408.78567	-4553.26597
InterB_para_2Kcation_MicroSolvation_acetonitrile	8.1	-3408.70401	0.95105	1.03908	1.04003	0.80449	-3408.81144	-4553.29396

InterA_pre-ortho_2Kcation_MicroSolvation_acetonitrile

C 12.0985518722 -15.0093081829 -1.2712248941
C 15.0419581142 -16.9145723137 -1.4285593379
C 14.9547992127 -16.7636980329 -3.8548449917
C 12.6364223889 -14.63171962 -4.9533925196
C 11.668062614 -15.4994883365 -5.3751816898
C 10.8399558267 -16.2364026121 -4.496005401
C 10.9622253537 -16.1428043037 -3.1518200273

C 11.9630730185 -15.2556764362 -2.6279430396
C 12.8269069981 -14.4916685646 -3.5520096821
C 10.3164577147 -16.7895602026 -6.583585152
H 13.2679369577 -14.1101130992 -5.6644674898
H 10.289586454 -16.6729605668 -2.4870119196
H 9.4526960207 -16.4855325848 -7.1832734529
O 11.3135433853 -15.7657923651 -6.6497972532
O 9.9323385619 -16.9420324957 -5.2307095401
H 10.7528863083 -17.7277647793 -6.9574461656
N 13.7397714275 -13.6881740518 -2.965118697
S 14.7140260616 -12.723880296 -3.8134121932
O 15.433532805 -13.3437733837 -4.940328785
O 15.5678338586 -12.081309323 -2.7855690625
C 10.4724445511 -17.4905838155 1.0666514013
C 10.2344618171 -16.669149933 2.1669043994
C 11.0770405729 -16.9857349698 -0.0774587492
H 9.761149678 -17.0773288453 3.0586365748
H 11.2996897987 -17.655024489 -0.9045036222
C 10.6342823142 -15.3302015737 2.1342010792
C 11.4689461336 -15.6315728785 -0.137001417
H 10.4616908689 -14.6857465057 2.9953298982
C 11.2619396467 -14.827215982 1.0064577813
H 11.5861831276 -13.7865150929 0.9709372263
H 10.2050362273 -18.5446174544 1.1022061529
C 13.9333784924 -10.9557592171 -5.7816180214
C 13.1491515748 -9.9243000952 -6.2903095849
C 13.6723733611 -11.4464968662 -4.5048443525
H 13.3499655985 -9.5405158637 -7.290157781
C 12.0980119959 -9.378692183 -5.5469552104
C 12.6242028107 -10.9238563927 -3.744689205
H 12.4131616502 -11.3370052706 -2.7582927547
C 11.8495198245 -9.8969751339 -4.2683627855
H 11.026264281 -9.4919654979 -3.6800680474
H 14.7354946085 -11.3965502917 -6.3712300747
C 11.2351768481 -8.2929697611 -6.1118036173
H 11.722565558 -7.777380945 -6.9455059194
H 10.2882946779 -8.6984097179 -6.4917800644
H 10.9752781835 -7.5444680199 -5.3548623948

H 12.6773543495 -14.1168267281 -1.0308743113
H 15.3014526244 -16.3116789794 -4.7830423648
C 15.5067452304 -16.3918736361 -2.6396154511
C 14.0281344969 -17.9181630139 -1.3768569692
C 13.4691518462 -18.3290609659 -2.6679476236
C 13.9250656027 -17.7371163778 -3.8873698766
C 13.3495521473 -18.1793905277 -5.1047745605
C 12.382779248 -19.1684887666 -5.1198953836
C 11.9276021749 -19.7371533603 -3.9172122339
C 12.4674718683 -19.314645733 -2.7138411102
H 16.3234898914 -15.6654129977 -2.6350911034
H 15.5092217725 -16.6384188799 -0.4792134321
O 13.634291301 -18.4471930631 -0.2933371281
H 12.133091303 -19.7486609138 -1.7705530051
H 11.1589037905 -20.5072314653 -3.9331816325
H 11.9744077378 -19.5155268102 -6.0696362493
H 13.703381256 -17.7392796808 -6.039274147
K 15.3743582159 -13.7775191714 -0.6173474663
K 13.8901488493 -20.7988055603 0.8738251494
N 11.071768054 -21.114554608 0.3058225675
C 10.5595472837 -21.2979239986 1.337103386
C 9.9457856662 -21.4977577856 2.6306610009
H 10.5652319444 -21.0036590488 3.3880356121
H 8.939044644 -21.0680962982 2.6494354338
H 9.8747276595 -22.5653872989 2.8606995684
N 13.404696881 -23.1131078567 2.4990681109
C 13.4331922849 -23.9494608755 3.3082941836
C 13.4725425603 -24.9887839576 4.313788319
H 13.0611042508 -24.6224023892 5.2593562273
H 12.8891600507 -25.8568725403 3.9917863096
H 14.5040698048 -25.3103620454 4.4873336536
N 15.5355265807 -21.1367794775 -1.4027416533
C 15.6258879826 -20.8370816662 -2.5251825157
C 15.7481112988 -20.4710068375 -3.9175889515
H 14.7622894756 -20.4946125323 -4.3974113664
H 16.1366834473 -19.4497293158 -4.0091112534
H 16.4189361619 -21.1616763864 -4.4385389547
N 16.2350245455 -19.2809997595 1.6142762176

C 16.9441381062 -18.8928850422 0.7747121324
C 17.8298194617 -18.4225968665 -0.2681331026
H 18.8178623056 -18.882861994 -0.1643539244
H 17.4148481155 -18.6837435132 -1.2473182338
H 17.9352611778 -17.3320791816 -0.22854592
N 12.4979839691 -19.5068804653 3.243183046
C 13.0256814998 -18.4983613152 2.9826891658
C 13.682098812 -17.2709608018 2.6028282839
H 13.8457060119 -17.3053878626 1.5140636324
H 13.0548532519 -16.4072906127 2.8440627776
H 14.6475388712 -17.1726266722 3.1077004975
N 14.5401211826 -14.2274031075 2.0466504277
C 14.1362109958 -13.2568031774 2.5495061431
C 13.6324568581 -12.04916525 3.1636825378
H 13.523392255 -12.1798753513 4.2446814679
H 14.3241066727 -11.2225288232 2.9721192056
H 12.6620994012 -11.7854117632 2.7313791703
N 16.8734077706 -11.7034258097 0.7465105584
C 17.585195509 -10.7868519552 0.8541178582
C 18.4705536281 -9.6481690109 0.9562381413
H 17.9214586833 -8.7588248473 1.2822422712
H 19.2704305153 -9.8414897693 1.6783111811
H 18.9104870192 -9.4577179591 -0.0291045014
N 18.0525552613 -14.8070185743 -0.6121171028
C 18.9327503606 -14.0443189197 -0.5616173971
C 20.018567076 -13.0929203807 -0.4960429296
H 20.2675471391 -12.8768527166 0.5482848438
H 20.9091168728 -13.4936743527 -0.9902676674
H 19.7229256545 -12.1617669143 -0.9969409275
N 18.5247962405 -10.5754034173 -2.1751484942
C 18.0835170296 -10.6503564033 -3.2516581104
C 17.5502867911 -10.7173395833 -4.5931111162
H 16.6217686568 -10.1378415766 -4.6500944343
H 18.2693114653 -10.3105273796 -5.3114850094
H 17.307993779 -11.7533107488 -4.8555584024
N 13.5467326563 -11.4754217546 -0.0742054769
C 14.1531200358 -10.6351914708 -0.6107733783
C 14.9057201555 -9.5886008293 -1.2679460091

H 14.8923129553 -8.671527085 -0.6691452476
H 15.9390110314 -9.9201420788 -1.4195904591
H 14.4703583273 -9.3791760067 -2.2518341114

TS_A-B_ortho_2Kcation_MicroSolvation_acetonitrile
C 12.2123622777 -15.5538598244 -1.2775955183
C 13.9152065176 -16.8098775041 -1.2448749306
C 14.5528831762 -16.7315582277 -3.5876882816
C 12.3769208855 -14.33296487 -4.8327619891
C 11.3687110677 -15.0918051099 -5.3690842299
C 10.5573613596 -15.9247258478 -4.5962643237
C 10.7577705742 -16.0669276486 -3.2549794894
C 11.8274791483 -15.3544370838 -2.6506195859
C 12.6178035951 -14.4348726057 -3.4365054502
C 9.981363637 -16.1751598168 -6.719469427
H 12.97752291 -13.6906119459 -5.4680041949
H 10.1143891361 -16.7132478585 -2.6699530671
H 9.113113325 -15.8267287372 -7.2877281877
O 10.9581997637 -15.1345574259 -6.6671852627
O 9.591019346 -16.486909178 -5.3937555894
H 10.4331717728 -17.0628096509 -7.1949810413
N 13.5586574872 -13.6827595701 -2.7588067078
S 14.6982424849 -12.8960973212 -3.5465011394
O 15.379451384 -13.6338062036 -4.6309151821
O 15.6013727532 -12.3421450489 -2.5015954695
C 9.7506977257 -17.5147789317 0.8847978735
C 9.812331047 -16.7726854692 2.0606372886
C 10.5214890596 -17.1655955603 -0.2192742669
H 9.2120340884 -17.0585530701 2.9219551664
H 10.4908176546 -17.7985349545 -1.101556441
C 10.6660842664 -15.673597976 2.1312412813
C 11.3663988104 -16.0433490807 -0.1804422826
H 10.7424902887 -15.0881895677 3.0471434423
C 11.4314137334 -15.3259977128 1.0276872084
H 12.0890924234 -14.4573578336 1.0899283805
H 9.1025328647 -18.3872212861 0.8259047388
C 14.4695296106 -10.9687958267 -5.5180119444
C 13.9515349587 -9.7954345399 -6.0581133863

C 13.9544969791 -11.4597957611 -4.3196516385
H 14.3514584125 -9.4130724767 -6.997250791
C 12.9138221664 -9.1026765684 -5.4245379517
C 12.9114697869 -10.7933451354 -3.6771214135
H 12.5031824209 -11.2001105875 -2.7535497386
C 12.4026947528 -9.6236005557 -4.2288335506
H 11.5851273079 -9.1050632448 -3.7280045591
H 15.256311749 -11.5228174585 -6.0276414494
C 12.3353292435 -7.8608192732 -6.0300244133
H 13.0632911035 -7.3354377694 -6.6574174178
H 11.474663647 -8.0974862752 -6.6694993417
H 11.978258597 -7.1624162141 -5.2652094543
H 12.8275190029 -14.7250600618 -0.9408795466
H 15.1078894332 -16.2725035791 -4.4042737428
C 14.7552001863 -16.3264081589 -2.3006425663
C 13.2231188778 -18.0806799514 -1.4326371451
C 12.9322762822 -18.4367628713 -2.8287309612
C 13.5721785962 -17.7353675681 -3.8884345551
C 13.2301393372 -18.074774097 -5.2134913795
C 12.2884579228 -19.058792498 -5.4756151679
C 11.6669943212 -19.7500466687 -4.425844146
C 12.0027424138 -19.4442764813 -3.1150620974
H 15.5008999147 -15.5625645699 -2.084344792
H 14.2025119816 -16.6033055673 -0.2113503026
O 12.8266026323 -18.7739171809 -0.47838077
H 11.5434791262 -19.9624789717 -2.2730899666
H 10.9210767321 -20.5129944389 -4.6405912082
H 12.0317254157 -19.2981891601 -6.5071132678
H 13.7085974659 -17.5383569831 -6.0337731242
K 14.7545680468 -13.1843480093 -0.0985027359
K 13.5651596923 -20.4989236657 1.3668025056
N 11.3741449959 -22.2620652609 1.9406122637
C 10.46396678 -22.8770670474 2.3252001185
C 9.3322399031 -23.6408429548 2.8039033084
H 8.5188004556 -22.9721488944 3.1017399932
H 8.96255447 -24.3098190567 2.0207204783
H 9.6178544064 -24.2454449965 3.6701503059
N 15.2005812366 -22.411667981 2.7379389745

C 15.9066372359 -23.1282286721 3.3230371256
C 16.7843892667 -24.0187841997 4.0510101709
H 16.3673357588 -24.2452310643 5.0370972974
H 16.9149471854 -24.9584065673 3.5054534532
H 17.7674398504 -23.5581175798 4.18864116
N 14.7579507029 -21.2425839181 -1.2050913689
C 14.7843502178 -21.2292861601 -2.3701378841
C 14.8328670998 -21.2077335533 -3.8160104387
H 13.9745278221 -21.7438760006 -4.2325965021
H 14.786320553 -20.1743791784 -4.1819588013
H 15.7527856957 -21.6779652459 -4.1781263729
N 15.8955803036 -18.7281691319 1.1078489579
C 16.3952960029 -18.6796229493 0.0558948095
C 17.0220934606 -18.6263964383 -1.246173412
H 17.5817085493 -19.5480853769 -1.4356409496
H 16.2615754677 -18.4976730919 -2.0254418627
H 17.7034298285 -17.7712536375 -1.3026175035
N 13.013819671 -18.3680556608 3.1941501256
C 13.6448023136 -17.401211018 3.0359718946
C 14.440496408 -16.2076472824 2.8534488518
H 13.8017230914 -15.3440754098 2.6340723776
H 15.0100446179 -15.9879428282 3.7625207866
H 15.1518131589 -16.3609157927 2.0332540862
N 13.4152067164 -12.768993353 2.3957773082
C 12.926191043 -11.7228823847 2.5539595786
C 12.3238271869 -10.421693998 2.7287379285
H 11.2485591013 -10.5172437962 2.9080970898
H 12.7760900986 -9.8991142335 3.5770795802
H 12.4856037315 -9.8360886197 1.8173777346
N 16.9014115514 -11.6121627602 0.9326849046
C 17.795465018 -10.8654574189 0.9169585992
C 18.9146158467 -9.9513395315 0.8725956275
H 18.565801187 -8.9136228441 0.8895193039
H 19.5753103387 -10.1052820534 1.7317049928
H 19.474181415 -10.126639229 -0.0530714676
N 16.8830024101 -15.0924623194 0.1906632638
C 17.9473456982 -14.616101429 0.213632622
C 19.2653589216 -14.0237092099 0.2430212253

H 19.485824108 -13.6518579041 1.2490873703
H 20.0246636314 -14.7636587917 -0.029152286
H 19.3122598863 -13.1855006894 -0.4651611965
N 18.9352458592 -11.5234397325 -1.9834200449
C 18.500526851 -11.6113513083 -3.0618446064
C 17.9786435725 -11.7000506523 -4.4053010493
H 17.2927347914 -10.8654580437 -4.5914450037
H 18.7931150488 -11.657432499 -5.1356524058
H 17.4052579765 -12.6263046497 -4.5351033091
N 13.8627542293 -10.3558364794 -0.252238458
C 14.6758623013 -9.9245230035 -0.9685578313
C 15.6950055122 -9.3831628874 -1.8397006441
H 15.9640022153 -8.3679303393 -1.5290906793
H 16.5793453921 -10.0296977388 -1.8083082114
H 15.3263281695 -9.3566540619 -2.871655681

InterB_ortho_2Kcation_MicroSolvation_acetonitrile
C 12.7238226838 -16.2429869706 -0.823895663
C 13.8371328499 -17.3411501236 -1.1826187478
C 14.7502911445 -16.9712376784 -3.4547672383
C 11.9339858706 -14.4457302765 -4.1021983148
C 10.9151710572 -15.2553309194 -4.5419208115
C 10.411728301 -16.3031834776 -3.7829207609
C 10.932610102 -16.5855680567 -2.5463740527
C 11.9995040837 -15.7971356604 -2.0653197478
C 12.5049926502 -14.7112239277 -2.8272118794
C 9.4055091839 -16.3405502066 -5.7575192985
H 12.3041228345 -13.6476937668 -4.7371074566
H 10.5578213006 -17.4237968428 -1.9592831422
H 8.3870006106 -16.0571942441 -6.0442339691
O 10.2270485423 -15.1766888203 -5.7221865241
O 9.3902011644 -16.9227324903 -4.4596764902
H 9.8268727036 -17.0615258574 -6.4790416308
N 13.5421738985 -13.9546218124 -2.2659054824
S 14.4829060183 -13.0993027232 -3.2200137076
O 14.9490105937 -13.7659593194 -4.4554566593
O 15.5675418543 -12.549631818 -2.360236966
C 11.5722904987 -17.5674506455 2.5878376308

C 10.2911919653 -17.0295830912 2.6870920449
C 12.327329872 -17.3693132697 1.4357635578
H 9.6976920944 -17.1815225813 3.5864963521
H 13.3227973035 -17.8052567009 1.3921617666
C 9.7805457049 -16.2996203854 1.6178428911
C 11.8408977399 -16.6269144625 0.3531037491
H 8.7784115796 -15.8766095805 1.6726810476
C 10.543899189 -16.1047729056 0.4680539977
H 10.130452554 -15.5264500546 -0.3558689784
H 11.9912728465 -18.1572227755 3.4020923269
C 13.8717712827 -11.0779589741 -5.0159351583
C 13.2343863595 -9.9022559052 -5.4007410886
C 13.5803207354 -11.6479539296 -3.7773218367
H 13.4619624509 -9.459668375 -6.3705415886
C 12.2925069243 -9.282935409 -4.5711490049
C 12.6313953626 -11.0572816696 -2.9445154905
H 12.3974715698 -11.52499393 -1.9898615292
C 12.0005707327 -9.8833903408 -3.340012479
H 11.2592766609 -9.4237009938 -2.6860603719
H 14.577818399 -11.5755863004 -5.6787144226
C 11.5842659805 -8.0369901966 -5.0062964854
H 12.1955586489 -7.4380335001 -5.6898215341
H 10.6539150906 -8.2763787215 -5.5381220808
H 11.3089124708 -7.4057237819 -4.1544459423
H 13.3268286693 -15.3885651618 -0.4843354992
H 15.3927863066 -16.4233477954 -4.1427943498
C 14.8217877231 -16.7483888446 -2.130738208
C 13.1611715849 -18.5906800622 -1.6975291989
C 13.0247840888 -18.7303664844 -3.1552672287
C 13.7754237144 -17.8882455501 -4.0126131219
C 13.5745846065 -17.9966597122 -5.3960625235
C 12.6615770979 -18.9089311346 -5.9123459654
C 11.9435396636 -19.7572267887 -5.0605970603
C 12.1360763704 -19.6693322505 -3.6883748691
H 15.5455264367 -16.0362433713 -1.7320834774
H 14.3371818355 -17.5877733228 -0.2385931698
O 12.6968842046 -19.4234105867 -0.911585546
H 11.580645185 -20.3017493699 -2.9972785814

H 11.2299027239 -20.4684460898 -5.4709564301
H 12.5080425877 -18.966385851 -6.9888314336
H 14.1339373782 -17.3411703539 -6.0629885626
K 15.3652772437 -13.5252035633 0.0979544529
K 14.4613991851 -20.5762833617 0.9557492409
N 11.7908539346 -21.0416359387 1.9447889616
C 10.9608199525 -20.5244305111 1.3100362421
C 9.9367927324 -19.8768355002 0.5222126255
H 10.2467158327 -18.8495509502 0.2969490903
H 9.7896410579 -20.4119289734 -0.4212480133
H 8.9892747763 -19.8502723417 1.0693143272
N 14.9753442101 -22.6419650848 2.8080801117
C 14.302149828 -22.3945306893 3.7269486313
C 13.453497578 -22.0639446787 4.8485856069
H 12.5371094359 -21.5970890297 4.4721348109
H 13.1934087283 -22.9621417641 5.4167258449
H 13.9618529441 -21.3545713137 5.5080933247
N 15.5458001656 -20.7224092348 -1.7279907382
C 15.5577004324 -20.7516697468 -2.8929106692
C 15.5756321212 -20.7853045702 -4.3381336738
H 14.5502218598 -20.7695268655 -4.7269534876
H 16.1019714452 -19.9093720518 -4.7322526923
H 16.0774280685 -21.690408843 -4.6945346848
N 16.4538745834 -18.4652717452 0.6447689621
C 17.1558383015 -18.3919596844 -0.2825125766
C 18.0109815015 -18.3160889318 -1.4447803756
H 18.3535171567 -19.3175076726 -1.7239880524
H 17.4492706771 -17.8825145977 -2.280539238
H 18.8779672656 -17.6816679117 -1.2398626468
N 14.5935667367 -19.0931372433 3.5094456738
C 14.9419420284 -17.9844454004 3.4093781707
C 15.3473035485 -16.6071909288 3.2406294747
H 14.4720278283 -15.9630971984 3.0850262198
H 15.8936691078 -16.2518187952 4.120006829
H 16.0028197124 -16.53625187 2.3636406942
N 13.2674749784 -14.048962552 1.9981287731
C 12.4255327344 -13.5305728231 1.3790869894
C 11.4222309812 -12.8873088015 0.5619343569

H 11.4518417717 -13.3170633905 -0.4492552654
H 10.4242846655 -13.0370367102 0.9852347368
H 11.6419336432 -11.8163812901 0.4972017566
N 17.4958901789 -11.7890774887 0.7987002292
C 18.2559084374 -10.9189459527 0.6503496326
C 19.2049847429 -9.8498479881 0.437960751
H 18.7398001147 -8.8772390839 0.6281166474
H 20.063632097 -9.9557880121 1.1087076604
H 19.5518425388 -9.8887090916 -0.600641002
N 17.7002131057 -15.1471525016 -0.3000722939
C 18.7075211056 -14.5599029213 -0.3318721111
C 19.9573946417 -13.8357261132 -0.3706298788
H 20.227568752 -13.5014466301 0.6363165556
H 20.7593888585 -14.474462225 -0.7542001073
H 19.8509609791 -12.9599808634 -1.0242755784
N 18.8583563465 -11.4530410345 -2.4019349183
C 18.230762821 -11.5619396631 -3.3786599202
C 17.469045741 -11.6666979624 -4.6008712465
H 16.7063625523 -10.8796707092 -4.6258949508
H 18.1240840478 -11.5522991297 -5.4706684289
H 16.9429239359 -12.6279608942 -4.6474799099
N 14.076272923 -10.9017971444 0.1799906464
C 14.7370591695 -10.3170896237 -0.5826118545
C 15.5651594038 -9.5959753814 -1.5233443517
H 15.8133110943 -8.6020423306 -1.1361637802
H 16.4838880397 -10.1648824127 -1.7066644874
H 15.0339589775 -9.4859320802 -2.4761067011

InterA_pre-para_2Kcation_MicroSolvation_acetonitrile
C -13.7793672608 17.2193756043 1.856958869
C -16.2587083965 16.7275631639 2.7825152012
C -11.998680978 14.9289901192 4.2044337939
C -11.3935465024 15.8710656733 4.9975983074
C -11.5958641082 17.2508275955 4.8331077398
C -12.4010828388 17.7496636241 3.8564333805
C -13.0515887942 16.8183393655 2.9853876846
C -12.8735507975 15.389482985 3.1918796592
C -10.1664956666 16.9445867464 6.5215728764

H -11.8472780805 13.8657870254 4.3533374863
H -12.4856844755 18.8180226876 3.688552363
H -9.0846188806 17.0752913505 6.3860051692
O -10.5461546286 15.6594204542 6.0346557414
O -10.8675274059 17.9316310597 5.7742048548
H -10.4381705732 17.0222732662 7.5819517564
N -13.6222080127 14.5284788808 2.4299722779
S -12.8654310354 13.4352962444 1.5289844905
O -11.5540071977 12.985184716 2.0265348755
O -13.8583912926 12.3919985131 1.1952202275
C -14.5421061446 20.917106387 1.6579916854
C -14.4568565614 21.1148153075 0.2810893092
C -14.3157015498 19.6577839393 2.2000354205
H -14.6378648219 22.1052275515 -0.1372649541
H -14.430499163 19.5100817833 3.2725010733
C -14.1924294987 20.0297588466 -0.5593627192
C -14.0230254397 18.5562788573 1.3719982833
H -14.1436203042 20.1727565372 -1.6387155348
C -14.0086064661 18.7632389999 -0.0236543921
H -13.8169122713 17.9088780396 -0.6747308015
H -14.8001742555 21.7434796487 2.3186270811
C -11.2986546013 14.8946396453 -0.2114677567
C -11.1087779986 15.7140913634 -1.3201702838
C -12.5548027038 14.3408583882 0.0211915601
H -10.1247465398 16.1436155659 -1.5075977778
C -12.1567517027 15.9942879685 -2.2028247436
C -13.6200223901 14.6095239874 -0.8401740896
H -14.6048670958 14.195457233 -0.6254174698
C -13.4104407639 15.4217140131 -1.9479791403
H -14.236892915 15.6240866366 -2.6303423785
H -10.4816776569 14.6703946724 0.4713542152
C -11.9562450317 16.9149144258 -3.3679553272
H -12.6564713608 16.7003077013 -4.1826823245
H -10.9395383178 16.8488327908 -3.7703234253
H -12.1131613256 17.9637683292 -3.0770182179
H -14.0082844422 16.4160276045 1.1566193678
H -15.8929580785 15.7384247069 2.5109844347
C -16.9932731691 17.4984179515 1.87592061

C -17.5173457517 18.7333146806 2.2175354351
C -17.3384495724 19.3052579968 3.5223061643
C -16.620666059 18.4605215046 4.4953694348
C -16.0913149858 17.1907262643 4.1151516814
C -15.4084343185 16.4288169275 5.0945006177
C -15.2673656585 16.8916610456 6.3900533859
C -15.8157171008 18.1315939961 6.763856409
C -16.4771129435 18.9019118169 5.8200994444
H -17.1255862656 17.1191283212 0.858595704
H -18.0915889026 19.3201970455 1.5025337891
O -17.7621801436 20.4432917716 3.8397802311
H -16.9111076101 19.8689280643 6.0709562317
H -15.7191690486 18.4828871241 7.7908816873
H -14.7396295294 16.2835456526 7.124668038
H -15.0113052614 15.4514206369 4.8160121868
K -15.9897404413 12.6839052227 2.8765103649
K -17.3256490082 23.0363030287 3.6567995676
N -17.8416735103 14.1158559628 4.4717361835
C -18.2189706184 14.9181642341 5.2269175818
C -18.6719753623 15.9289763768 6.1544363114
H -18.1608278454 15.8153886717 7.1155171961
H -18.4337229178 16.9252870751 5.7617804725
H -19.7518801085 15.8537448629 6.3154003943
N -18.8026547566 11.5903942241 2.3613384479
C -18.8573751171 10.4297067891 2.4521367033
C -18.8999705295 8.9884239569 2.557339491
H -19.1743329401 8.6827056969 3.5716751853
H -19.632113691 8.5731081005 1.8579525137
H -17.9099199622 8.5869902285 2.3166774151
N -17.0512663386 13.9852337968 0.432364893
C -18.0605245455 14.342910921 0.896079852
C -19.3025401912 14.7792806551 1.4924035625
H -19.6732847577 13.9867760199 2.1509097862
H -19.1311901273 15.6884906112 2.0801863401
H -20.0481008463 14.9868942551 0.71838461
N -15.9995594816 9.9917844069 1.3536960308
C -15.7737804356 10.475761983 0.3165646965
C -15.4962638372 11.0954676473 -0.9597386038

H -15.8184719526 10.4497500608 -1.7824920858
H -16.0266016971 12.0527121686 -1.0188439568
H -14.4241927513 11.2965991032 -1.0411425759
N -13.9448693008 11.599781963 4.546460414
C -13.2730952415 10.9219360955 3.8751974817
C -12.4494941553 10.0898952144 3.0278372278
H -11.991111502 9.2817731351 3.6061017345
H -13.0668197354 9.6601002932 2.2323342435
H -11.6736488856 10.7102071018 2.5668388101
N -15.1140781888 24.3848197665 2.338426377
C -14.0511472862 24.4748788678 1.8717267949
C -12.7297068001 24.563679831 1.2886404816
H -12.2630902076 23.5731964125 1.2632104217
H -12.7858239409 24.947003494 0.2649055117
H -12.0932837173 25.2338391879 1.8745919697
N -16.9681250011 25.0633488754 5.6773405221
C -16.7857865371 25.9774408731 6.374237663
C -16.5597185763 27.1145976461 7.2403661888
H -15.5757457254 27.0459837969 7.714381508
H -16.6038190671 28.0461517558 6.6677229919
H -17.320413491 27.1548941887 8.0260905881
N -17.7097806644 22.4347624362 0.8620840534
C -17.6812187099 21.6030148461 0.0475641224
C -17.6384056535 20.5598033694 -0.9547551725
H -17.2466846497 20.949076554 -1.9003574466
H -16.9829258042 19.7495798397 -0.6141287495
H -18.6408483754 20.1569671803 -1.132730102
N -19.9483831376 24.2334121784 3.4343126986
C -21.008240765 24.6343948234 3.1691730565
C -22.3260008032 25.1325553331 2.838628099
H -22.9457106862 24.3300076752 2.4269900054
H -22.82108233 25.5293829192 3.7302722925
H -22.25707729 25.9327045168 2.0952292476
N -14.8986224282 21.9322024208 4.8934610821
C -14.150430328 21.2295171238 5.4452665941
C -13.2251556629 20.348053419 6.1244932061
H -13.4985697053 19.3018415169 5.9402523659
H -12.1997816779 20.5097825421 5.7763916797

H -13.2557725517 20.523116942 7.2049687614

TS_A-B_para_2Kcation_MicroSolvation_acetonitrile
C -13.9397760582 17.1633600625 1.9738233483
C -15.9830108912 16.8160674036 2.8634127491
C -11.959211323 14.8703840853 4.1898459206
C -11.2857672817 15.8117220677 4.9318285203
C -11.4760851119 17.1839805387 4.7537950678
C -12.3397155038 17.6746716707 3.816481594
C -13.0587595143 16.7430344776 3.016069359
C -12.8811691197 15.3288811434 3.2209204076
C -9.9022312215 16.892365727 6.3043142002
H -11.8064804804 13.8060046641 4.3323198342
H -12.4268290545 18.7415973334 3.6390156888
H -8.8506073199 16.9934228973 5.9979419008
O -10.3791146134 15.6037913556 5.9249416885
O -10.6923132396 17.8760401508 5.6437343869
H -10.0029385013 17.0097955368 7.3897270593
N -13.6763268426 14.442131581 2.5114203489
S -12.9499174643 13.4411364845 1.4997629743
O -11.588462208 13.0250385281 1.884135305
O -13.9180063462 12.3667376982 1.1830314274
C -14.3093145788 20.9252131431 1.6442474217
C -14.1484103049 21.0771261922 0.2680733749
C -14.2292618908 19.6634637393 2.2224169883
H -14.2188482673 22.0669706644 -0.1834714036
H -14.3866048112 19.5566181099 3.2942802434
C -13.9413922696 19.951212997 -0.5298852968
C -14.0148135309 18.5190544823 1.4356603186
H -13.8231594693 20.0565913418 -1.6083053661
C -13.9045733772 18.6854690291 0.0438611872
H -13.7601288834 17.8027548682 -0.5801544681
H -14.5124658379 21.7892222557 2.2760463704
C -11.5562175144 15.0143686476 -0.2946622456
C -11.4637457631 15.8497830411 -1.4034314909
C -12.7668986923 14.3876773164 -0.0073470329
H -10.5177414088 16.3436578333 -1.6260295786
C -12.5640790105 16.0707304187 -2.2387485886

C -13.88336623 14.6032839749 -0.8169291748
H -14.8333824139 14.1363723004 -0.5579736811
C -13.7715017221 15.4318283032 -1.9276934999
H -14.6411437549 15.5989864571 -2.5645319786
H -10.6982742776 14.8364513767 0.3505260399
C -12.4554759731 16.9783754135 -3.4263994749
H -13.4407051081 17.3091074268 -3.7749307283
H -11.9653418567 16.4805027711 -4.2735449563
H -11.8593136737 17.8707498645 -3.199272712
H -14.1538730649 16.3787715229 1.2481333769
H -15.724280269 15.7632747619 2.747978258
C -16.8172457332 17.4332542699 1.8977663463
C -17.4405686732 18.6349991397 2.124883714
C -17.3246820013 19.3320489484 3.3836088619
C -16.5971755385 18.6149170809 4.4512130595
C -15.9497821651 17.3779774862 4.1866513143
C -15.2810723639 16.7317758745 5.2497677435
C -15.2684373277 17.278802273 6.522357752
C -15.9269163566 18.4919933409 6.779954092
C -16.5759330062 19.1511236232 5.7478340981
H -16.9070430726 16.951193496 0.9201791848
H -18.0493470835 19.1126211539 1.3587649702
O -17.8169648561 20.4623797265 3.5818339379
H -17.091091463 20.0967380405 5.9081362744
H -15.9256396872 18.9115709406 7.7853255649
H -14.7521121487 16.7563667161 7.3271384177
H -14.7935203095 15.7756334894 5.0601358989
K -15.9110404439 12.5262079597 3.0374329684
K -17.2630120325 23.0700734072 3.531480085
N -17.4762390475 14.2282055633 4.664077734
C -17.9592598558 15.0217369949 5.3660657746
C -18.5513345311 16.024042304 6.2220614061
H -18.1544590496 15.9385019336 7.2384452889
H -18.3072848088 17.0251615329 5.8452011071
H -19.6392631781 15.9090527985 6.2582992656
N -18.8284776502 11.6428779207 2.8955767169
C -18.9013735426 10.4800092932 2.8599759465
C -18.9700475904 9.0369605592 2.807952204

H -19.1059436233 8.6200868624 3.8106034171
H -19.8056940681 8.7142915936 2.1792808612
H -18.0354535938 8.6559401106 2.3830869167
N -17.147250005 13.9182716561 0.7106862503
C -18.105296733 14.3181279249 1.24316814
C -19.287411169 14.8111560474 1.9135512624
H -19.5951426508 14.0791398805 2.6676572847
H -19.0674424517 15.7685131391 2.4008900864
H -20.1019326197 14.9552218005 1.1966352815
N -16.2396435443 10.0614781913 1.3115702969
C -15.9482658513 10.5274485006 0.2829563281
C -15.5838510707 11.1139218159 -0.9867152072
H -15.7723478939 10.4133707188 -1.8060827802
H -16.1693594347 12.0251115139 -1.1476806914
H -14.5255914387 11.3907074107 -0.9631799595
N -13.8905731511 11.0592199704 4.390124083
C -13.1713982078 10.5769058743 3.6086861644
C -12.282411441 9.9899552312 2.6323315464
H -11.6833485922 9.1935576694 3.0845932436
H -12.8687769368 9.574722828 1.8064823792
H -11.628854631 10.7730414894 2.2314963695
N -14.9987094871 24.4746236484 2.3852876469
C -13.9268995747 24.5691607805 1.9405071489
C -12.5938692654 24.6653337901 1.3857299864
H -12.1218074283 23.6772108747 1.3666681599
H -12.6301521603 25.0523652673 0.3625341635
H -11.9732305302 25.3362133248 1.9875729982
N -17.3791319049 24.6597473085 5.9431021642
C -17.3938725055 25.4159663281 6.8276508414
C -17.4126594158 26.3569631879 7.9268457156
H -16.6183576271 26.1258559812 8.6432503811
H -17.2613092537 27.3766594372 7.5594875186
H -18.3732312657 26.3163311607 8.4495982672
N -17.424976451 22.4204031226 0.7292694829
C -17.3843107372 21.5560779516 -0.0499138109
C -17.32856605 20.478463341 -1.0143068023
H -16.8656701191 20.8202297883 -1.9459019393
H -16.7283935869 19.653674276 -0.6125694334

H -18.3355595348 20.112291311 -1.2398412372
N -19.7886618022 24.3798201323 2.9997729358
C -20.7664632399 24.85390162 2.5831622325
C -21.9817783841 25.4431409226 2.0638495352
H -22.6091595699 24.677049718 1.5979320454
H -22.5532856818 25.9165099365 2.8681468531
H -21.7470933449 26.2028092695 1.3118876068
N -14.8873857399 21.9687052378 4.8484772826
C -14.1168534315 21.2589864522 5.3591407855
C -13.1649493438 20.3626053432 5.9794602096
H -13.4429931851 19.3199804793 5.7793045701
H -12.1548694383 20.5349979595 5.5931943826
H -13.1511892662 20.5092932935 7.064359625

InterB_para_2Kcation_MicroSolvation_acetonitrile
C -14.2680176612 17.0923934723 2.5834595261
C -15.69542456 16.7712482113 3.1801344882
C -11.8489149314 14.611684303 4.1842258586
C -11.1465697688 15.4695102876 4.9991846275
C -11.421399266 16.8303650314 5.0549748521
C -12.4139313708 17.3922985078 4.2868581877
C -13.1491939967 16.5439335152 3.434942798
C -12.8781821101 15.1568850767 3.3806050661
C -9.6542128078 16.4284811624 6.3381453498
H -11.6218120201 13.551919619 4.1292884635
H -12.6103679983 18.4627649206 4.3129884844
H -8.6800097386 16.6455691342 5.8700640466
O -10.1344068655 15.1722895259 5.8735879371
O -10.594381945 17.434560953 5.9696666497
H -9.5604997433 16.4011876165 7.4295868936
N -13.6881594074 14.3267882246 2.5797066527
S -12.9924696244 13.6406457708 1.324765865
O -11.6036126214 13.1823601495 1.5219226108
O -13.9567185089 12.6354310122 0.8099642986
C -14.4464950119 20.9003834916 2.0236642322
C -13.3691064519 21.0844237768 1.1642315715
C -14.7584304137 19.625642112 2.5034054692
H -13.1303784125 22.0766917874 0.7825678507

H -15.6188894514 19.515334178 3.1577826673
C -12.6036973379 19.9783693162 0.7856584026
C -14.0086898323 18.5105468849 2.129450505
H -11.7539910474 20.1016449286 0.1148658717
C -12.9228689118 18.7130303526 1.2616225612
H -12.3241880466 17.8511543803 0.9649614988
H -15.0870178561 21.7359487802 2.3062285526
C -11.6964035089 15.4544208007 -0.3438214274
C -11.6832281062 16.4851114419 -1.283041382
C -12.9093671439 14.8858740469 0.0342900881
H -10.731954916 16.9266698545 -1.582264089
C -12.8684252899 16.9701849493 -1.8447333285
C -14.1045140002 15.3430734271 -0.5265555173
H -15.0498650643 14.889781702 -0.2223976575
C -14.0763854696 16.373951798 -1.4569433274
H -15.0099813826 16.720279788 -1.9042688759
H -10.775189856 15.074746444 0.0934678203
C -12.8548140887 18.1205725876 -2.8045413641
H -13.7514711425 18.1361601374 -3.435131483
H -11.9805834372 18.0949879633 -3.4645481247
H -12.8191253365 19.0817114492 -2.2704583405
H -14.2675772633 16.4757543217 1.6746878665
H -15.6461446066 15.6681177255 3.2394775225
C -16.7310921953 17.0920914322 2.1670516037
C -17.7937212307 17.8908638296 2.3810311322
C -17.9871359507 18.5755274503 3.6535849788
C -17.0946864313 18.1695502323 4.7676091261
C -16.0177195396 17.2826005923 4.555632204
C -15.2995169998 16.8363590401 5.6779550111
C -15.5959103779 17.3092333297 6.9517399738
C -16.6259866726 18.2362049102 7.1443383746
C -17.376261502 18.6517255069 6.0552053886
H -16.5728803846 16.6460324897 1.1813744087
H -18.5067607593 18.1223295673 1.591535721
O -18.8562771367 19.4428078933 3.8075732402
H -18.2043451682 19.3502579056 6.1621868556
H -16.8501812934 18.6089765649 8.1424390466
H -15.0196991195 16.9493190536 7.8026897297

H -14.5073199626 16.1024587062 5.5462195629
K -15.9385447259 12.5501024422 2.6161729923
K -18.8888962492 22.1005303715 3.0833914976
N -17.5552336381 14.2568431347 4.2574325156
C -17.9761661612 14.6478406779 5.2707815352
C -18.4968114287 15.1326794457 6.5288700862
H -17.8607624244 14.7986949657 7.354691592
H -18.5149901823 16.2285707662 6.5322212039
H -19.5113136271 14.7571541828 6.694829995
N -18.4769201361 11.1316380035 3.0703629595
C -18.1098320522 10.0368628764 2.9062080328
C -17.6330543765 8.6909181933 2.6877400802
H -16.8644004262 8.4426273098 3.425800866
H -18.4545767937 7.9718623383 2.7611970545
H -17.1845902724 8.6403603993 1.6901713979
N -17.3777572413 14.1601348215 0.5569031734
C -18.4155431622 14.104674014 1.0871112107
C -19.6892992075 14.0284767714 1.7654919328
H -19.6842723357 13.1597406577 2.4348580549
H -19.8484369609 14.9357786623 2.3576304505
H -20.5042423897 13.9229426561 1.0427353524
N -16.51599595 10.6308314401 0.2657027026
C -16.1258605203 11.2925311841 -0.6114597968
C -15.6376889011 12.1207104309 -1.6910027189
H -15.696174637 11.5890658277 -2.6458102998
H -16.236229734 13.0357858817 -1.7512265538
H -14.6010968562 12.4022459632 -1.4796376331
N -14.5716330999 10.1297538895 3.2640780367
C -13.6611238891 10.0678322324 2.5370217866
C -12.5347286454 9.9915351342 1.6351580395
H -11.8981486121 9.1380742481 1.8889429668
H -12.8961550721 9.881537615 0.60782107
H -11.9531353766 10.9208973306 1.683095154
N -16.8945405591 24.056718755 2.2939197149
C -15.8743035891 24.2409673268 2.8251808818
C -14.6065685567 24.4540734892 3.4903672043
H -13.9307972275 23.6123702732 3.2981428666
H -14.1296778356 25.3720998828 3.1333889693

H -14.7586211876 24.5347533763 4.5714728077
N -19.6308283164 24.0841740812 5.0046519302
C -19.9344183372 24.9822690033 5.6798361577
C -20.312673863 26.0992040255 6.5182545413
H -19.7391506382 26.0909669918 7.4502186467
H -20.1232180507 27.045659656 6.0025370469
H -21.377122598 26.0475439575 6.7669825535
N -17.6764628839 20.8589509797 0.8295981966
C -16.9238323904 20.3366908504 0.1103648717
C -15.98647412 19.6765080759 -0.7713836115
H -15.344255939 20.4124862695 -1.2659609648
H -15.3425144168 18.9972596879 -0.1965081304
H -16.5174562266 19.1017234058 -1.5376121837
N -21.381782291 22.6963673775 1.8048206207
C -22.3103537109 22.9919267354 1.1685051196
C -23.4638193946 23.3597260758 0.3765822551
H -23.7673405844 22.5276083555 -0.2660452898
H -24.3057096967 23.6222743464 1.0245066053
H -23.2322095276 24.2208120891 -0.2578160075
N -16.5210836538 21.7704546446 4.7500558984
C -15.5885214726 21.2320979921 5.1968836193
C -14.4230147663 20.554334466 5.7201655469
H -14.4852086703 19.4807196314 5.4985066907
H -13.5126450781 20.9476540269 5.253795428
H -14.3524122768 20.6795558799 6.8052717006

TS_A-B_para_2Kcation_MicroSolvation_acetonitrile
C -13.9397760582 17.1633600625 1.9738233483
C -15.9830108912 16.8160674036 2.8634127491
C -11.959211323 14.8703840853 4.1898459206
C -11.2857672817 15.8117220677 4.9318285203
C -11.4760851119 17.1839805387 4.7537950678
C -12.3397155038 17.6746716707 3.816481594
C -13.0587595143 16.7430344776 3.016069359
C -12.8811691197 15.3288811434 3.2209204076
C -9.9022312215 16.892365727 6.3043142002
H -11.8064804804 13.8060046641 4.3323198342
H -12.4268290545 18.7415973334 3.6390156888

H -8.8506073199 16.9934228973 5.9979419008
O -10.3791146134 15.6037913556 5.9249416885
O -10.6923132396 17.8760401508 5.6437343869
H -10.0029385013 17.0097955368 7.3897270593
N -13.6763268426 14.442131581 2.5114203489
S -12.9499174643 13.4411364845 1.4997629743
O -11.588462208 13.0250385281 1.884135305
O -13.9180063462 12.3667376982 1.1830314274
C -14.3093145788 20.9252131431 1.6442474217
C -14.1484103049 21.0771261922 0.2680733749
C -14.2292618908 19.6634637393 2.2224169883
H -14.2188482673 22.0669706644 -0.1834714036
H -14.3866048112 19.5566181099 3.2942802434
C -13.9413922696 19.951212997 -0.5298852968
C -14.0148135309 18.5190544823 1.4356603186
H -13.8231594693 20.0565913418 -1.6083053661
C -13.9045733772 18.6854690291 0.0438611872
H -13.7601288834 17.8027548682 -0.5801544681
H -14.5124658379 21.7892222557 2.2760463704
C -11.5562175144 15.0143686476 -0.2946622456
C -11.4637457631 15.8497830411 -1.4034314909
C -12.7668986923 14.3876773164 -0.0073470329
H -10.5177414088 16.3436578333 -1.6260295786
C -12.5640790105 16.0707304187 -2.2387485886
C -13.88336623 14.6032839749 -0.8169291748
H -14.8333824139 14.1363723004 -0.5579736811
C -13.7715017221 15.4318283032 -1.9276934999
H -14.6411437549 15.5989864571 -2.5645319786
H -10.6982742776 14.8364513767 0.3505260399
C -12.4554759731 16.9783754135 -3.4263994749
H -13.4407051081 17.3091074268 -3.7749307283
H -11.9653418567 16.4805027711 -4.2735449563
H -11.8593136737 17.8707498645 -3.199272712
H -14.1538730649 16.3787715229 1.2481333769
H -15.724280269 15.7632747619 2.747978258
C -16.8172457332 17.4332542699 1.8977663463
C -17.4405686732 18.6349991397 2.124883714
C -17.3246820013 19.3320489484 3.3836088619

C -16.5971755385 18.6149170809 4.4512130595
C -15.9497821651 17.3779774862 4.1866513143
C -15.2810723639 16.7317758745 5.2497677435
C -15.2684373277 17.278802273 6.522357752
C -15.9269163566 18.4919933409 6.779954092
C -16.5759330062 19.1511236232 5.7478340981
H -16.9070430726 16.951193496 0.9201791848
H -18.0493470835 19.1126211539 1.3587649702
O -17.8169648561 20.4623797265 3.5818339379
H -17.091091463 20.0967380405 5.9081362744
H -15.9256396872 18.9115709406 7.7853255649
H -14.7521121487 16.7563667161 7.3271384177
H -14.7935203095 15.7756334894 5.0601358989
K -15.9110404439 12.5262079597 3.0374329684
K -17.2630120325 23.0700734072 3.531480085
N -17.4762390475 14.2282055633 4.664077734
C -17.9592598558 15.0217369949 5.3660657746
C -18.5513345311 16.024042304 6.2220614061
H -18.1544590496 15.9385019336 7.2384452889
H -18.3072848088 17.0251615329 5.8452011071
H -19.6392631781 15.9090527985 6.2582992656
N -18.8284776502 11.6428779207 2.8955767169
C -18.9013735426 10.4800092932 2.8599759465
C -18.9700475904 9.0369605592 2.807952204
H -19.1059436233 8.6200868624 3.8106034171
H -19.8056940681 8.7142915936 2.1792808612
H -18.0354535938 8.6559401106 2.3830869167
N -17.147250005 13.9182716561 0.7106862503
C -18.105296733 14.3181279249 1.24316814
C -19.287411169 14.8111560474 1.9135512624
H -19.5951426508 14.0791398805 2.6676572847
H -19.0674424517 15.7685131391 2.4008900864
H -20.1019326197 14.9552218005 1.1966352815
N -16.2396435443 10.0614781913 1.3115702969
C -15.9482658513 10.5274485006 0.2829563281
C -15.5838510707 11.1139218159 -0.9867152072
H -15.7723478939 10.4133707188 -1.8060827802
H -16.1693594347 12.0251115139 -1.1476806914

H -14.5255914387 11.3907074107 -0.9631799595
N -13.8905731511 11.0592199704 4.390124083
C -13.1713982078 10.5769058743 3.6086861644
C -12.282411441 9.9899552312 2.6323315464
H -11.6833485922 9.1935576694 3.0845932436
H -12.8687769368 9.574722828 1.8064823792
H -11.628854631 10.7730414894 2.2314963695
N -14.9987094871 24.4746236484 2.3852876469
C -13.9268995747 24.5691607805 1.9405071489
C -12.5938692654 24.6653337901 1.3857299864
H -12.1218074283 23.6772108747 1.3666681599
H -12.6301521603 25.0523652673 0.3625341635
H -11.9732305302 25.3362133248 1.9875729982
N -17.3791319049 24.6597473085 5.9431021642
C -17.3938725055 25.4159663281 6.8276508414
C -17.4126594158 26.3569631879 7.9268457156
H -16.6183576271 26.1258559812 8.6432503811
H -17.2613092537 27.3766594372 7.5594875186
H -18.3732312657 26.3163311607 8.4495982672
N -17.424976451 22.4204031226 0.7292694829
C -17.3843107372 21.5560779516 -0.0499138109
C -17.32856605 20.478463341 -1.0143068023
H -16.8656701191 20.8202297883 -1.9459019393
H -16.7283935869 19.653674276 -0.6125694334
H -18.3355595348 20.112291311 -1.2398412372
N -19.7886618022 24.3798201323 2.9997729358
C -20.7664632399 24.85390162 2.5831622325
C -21.9817783841 25.4431409226 2.0638495352
H -22.6091595699 24.677049718 1.5979320454
H -22.5532856818 25.9165099365 2.8681468531
H -21.7470933449 26.2028092695 1.3118876068
N -14.8873857399 21.9687052378 4.8484772826
C -14.1168534315 21.2589864522 5.3591407855
C -13.1649493438 20.3626053432 5.9794602096
H -13.4429931851 19.3199804793 5.7793045701
H -12.1548694383 20.5349979595 5.5931943826
H -13.1511892662 20.5092932935 7.064359625

TS_A-B_para_2Kcation_MicroSolvation_acetonitrile_conf1

C -12.0745740156 17.6185224694 1.4493973663
C -14.1068124667 17.5808741871 0.3510477225
C -9.7053538237 19.5038444472 -0.7745228216
C -9.273024201 18.475775474 -1.5776218052
C -9.7945304467 17.1860849379 -1.4727539851
C -10.736354906 16.8699102451 -0.53002411
C -11.1538051479 17.8846128951 0.3755331917
C -10.6760649625 19.2267552839 0.2162182294
C -8.2329831739 17.1739497597 -3.0386930831
H -9.3652359271 20.5261944359 -0.9157933664
H -11.1834777866 15.8801312665 -0.504385427
H -7.2737890716 16.816738296 -2.6317840236
O -8.426982846 18.5375311964 -2.6504342153
O -9.3033841261 16.4140947269 -2.4969675326
H -8.2498207915 17.1038601093 -4.1298430974
N -11.2152785566 20.3008198713 0.9141544439
S -10.6377011125 20.8048604079 2.2966150581
O -9.2527671387 20.3832668767 2.5915570561
O -10.9176547786 22.2676057656 2.3547266548
C -11.3119498985 14.2550708916 3.0325959926
C -12.1478296652 14.309577375 4.1481181652
C -11.2719021878 15.3065621467 2.1277373367
H -12.1695880765 13.4830510122 4.8557441907
H -10.5593222506 15.2627513171 1.3101581628
C -12.9365038055 15.4381658414 4.3595302018
C -12.0830230247 16.4473578309 2.3054411998
H -13.5844464211 15.501978966 5.2326712545
C -12.9061985462 16.4853189123 3.4515046512
H -13.5267422692 17.3656996575 3.6166735621
H -10.6674149396 13.391981218 2.8781834903
C -11.0722584513 19.4321564561 4.6741355159
C -11.8875272526 18.9478282649 5.6984779703
C -11.6424336104 20.1525901093 3.6309528697
H -11.4421877519 18.3765910832 6.5131464123
C -13.268192956 19.1552235973 5.685137841
C -13.0222263252 20.3780646497 3.5993377871
H -13.4659867184 20.9433138291 2.7792432532

C -13.8218416829 19.8749879435 4.6146488765
H -14.9021571736 20.0218473843 4.5681507273
H -9.9989197061 19.2554970013 4.674383006
C -14.1582941871 18.6011660786 6.7541973762
H -14.9392908042 17.9582765714 6.3241878926
H -14.67789328 19.3999997374 7.299784445
H -13.5999035213 18.0066300121 7.4849064282
H -12.438333311 18.5161343793 1.9401048111
H -13.6742256057 16.8836906156 -0.3658955589
C -14.1354740586 18.9675260788 0.0447754551
C -14.920044093 19.8516199908 0.7387277283
C -15.7867246461 19.4236690951 1.8037038463
C -15.8395383804 17.9785532103 2.0653010277
C -15.0524102594 17.0736618134 1.3005301931
C -15.1774763647 15.6939068565 1.5549449258
C -16.0371944415 15.2242301298 2.5324076849
C -16.7924227437 16.1214770958 3.2999003555
C -16.6915268135 17.4822305294 3.0621429335
H -13.4615750587 19.3494413684 -0.724607643
H -14.8897587539 20.9199600113 0.5247592281
O -16.4678261427 20.2380696794 2.4794086929
H -17.2751244158 18.2029910155 3.6329145226
H -17.4598673483 15.7471097654 4.0739789558
H -16.1139432019 14.1537631995 2.7150246571
H -14.5664156793 14.9952187658 0.9819421382
K -12.2952650577 22.7719819826 0.0040507956
K -16.7102123586 22.5704890358 3.6772777558
N -17.7399505844 23.2443715387 1.1003288086
C -17.8070213773 22.2855488533 0.4400993654
C -17.905186779 21.0991731222 -0.3789653108
H -18.2637589884 20.2585392724 0.2231054476
H -18.595838287 21.2650844156 -1.2118034094
H -16.9164984078 20.8458256894 -0.7761955686
N -16.540636434 25.4839529656 4.0169935801
C -16.5788294613 25.6287248259 5.1728870452
C -16.6188829675 25.7812753518 6.6102190292
H -17.6422175862 25.9733522994 6.9472049969
H -16.2541486 24.8596787303 7.0764316385

H -15.9846704558 26.6150376363 6.9269290371
N -18.728241619 22.9247158528 5.6843922904
C -19.686524969 22.9447109224 6.3455063054
C -20.8787838124 22.9683622084 7.1645486438
H -21.5077615149 23.8239359534 6.8999559073
H -21.4601822095 22.053225129 7.0159151477
H -20.6157650899 23.0425431171 8.2242061133
N -14.8380664175 23.0729099829 5.8065176368
C -13.7151886676 23.1398437689 5.4968509591
C -12.3253770488 23.2236328956 5.114315374
H -11.9165084968 24.2017030915 5.3906089881
H -11.7473908823 22.4405742027 5.6178910309
H -12.2132320488 23.0763667025 4.0317216364
N -14.3535432824 23.3240291726 2.1638936178
C -14.7435400467 24.263755971 1.5912000604
C -15.2139199224 25.4208662853 0.8700926991
H -15.9783064158 25.9278746848 1.4674734398
H -14.374131176 26.0975803607 0.6810012359
H -15.6524484737 25.1033776366 -0.0812596982
N -11.7853721528 25.502364227 1.4856068976
C -10.7815591611 25.2838096415 2.0376639941
C -9.5373862186 25.0032301952 2.7190460785
H -9.4833906317 25.5482022234 3.6670185873
H -9.484160822 23.924382308 2.9095503955
H -8.6886223402 25.3046464241 2.0955874572
N -12.6029182843 25.0948391097 -1.8330369571
C -11.6995900188 25.7982201242 -1.6138036027
C -10.5748462244 26.6612467617 -1.3343638387
H -10.8239129447 27.348387673 -0.5201128092
H -9.722712489 26.0437237434 -1.0302542892
H -10.3010596423 27.2397147795 -2.2220738938
N -9.4557769185 23.572963777 -0.4367921468
C -8.5796444 22.9753221953 0.0498521019
C -7.5084083614 22.2283486713 0.6686929227
H -6.8118844411 22.9065650128 1.1731683421
H -7.9289962363 21.5271818676 1.4063206865
H -6.9529610878 21.6611582676 -0.0854040201
N -12.1322878522 21.3347330194 -2.5110885894

C -11.9760709052 20.2860837303 -2.9949545743
C -11.7935576429 18.978826654 -3.5869570973
H -12.4432597434 18.8544271552 -4.4589985637
H -10.75215592 18.8461325313 -3.8993036638
H -12.032522917 18.1990773969 -2.8536857123
N -15.0679777241 22.6612137248 -1.3258415735
C -14.8368572648 22.9504263372 -2.4316470299
C -14.5330038132 23.3156481582 -3.7959448028
H -15.3635629248 23.8713750612 -4.2420791101
H -13.6386029172 23.9466796186 -3.7964987782
H -14.3387284585 22.4218018138 -4.3961098286

TS_A-B_para_2Kcation_MicroSolvation_acetonitrile_conf2
C -12.956043 16.425705 1.358389
C -15.073131 17.325177 1.595033
C -12.091268 17.59289 -2.093721
C -12.40689 16.461093 -2.806698
C -12.871313 15.301464 -2.185232
C -13.055141 15.236878 -0.831974
C -12.757314 16.392119 -0.052594
C -12.250288 17.576153 -0.69463
C -12.731741 14.91071 -4.377371
H -11.740761 18.501651 -2.567021
H -13.388376 14.316982 -0.362046
H -11.902379 14.351255 -4.829695
O -12.31192 16.262549 -4.147567
O -13.079273 14.331743 -3.128992
H -13.605715 14.915781 -5.041697
N -11.945113 18.742676 0.004769
S -10.540612 18.861274 0.71646
O -10.50194 20.184143 1.38426
O -10.143228 17.705337 1.561127
C -14.022172 13.144493 2.956275
C -13.107324 13.071561 4.005253
C -13.998339 14.221536 2.078395
H -13.128248 12.226854 4.69043
H -14.740847 14.278174 1.282892
C -12.171897 14.089842 4.174391

C -13.061344 15.256731 2.233066
H -11.450306 14.038414 4.987542
C -12.156738 15.175152 3.306317
H -11.405902 15.957683 3.413488
H -14.764754 12.360085 2.825002
C -8.804363 20.150862 -1.006224
C -7.81147 20.194513 -1.98086
C -9.267502 18.920851 -0.546192
H -7.433217 21.16186 -2.316864
C -7.27977 19.021297 -2.527243
C -8.758824 17.735488 -1.082629
H -9.110525 16.777896 -0.701562
C -7.776015 17.793058 -2.065373
H -7.369728 16.867183 -2.473065
H -9.195329 21.068818 -0.571956
C -6.228505 19.069075 -3.593853
H -5.798695 20.070866 -3.696898
H -6.63663 18.786122 -4.574268
H -5.407887 18.371861 -3.388971
H -12.51811 17.298004 1.83334
H -15.471507 16.314616 1.54034
C -14.883034 17.919568 2.864594
C -14.660643 19.269249 3.013406
C -14.650954 20.153859 1.881899
C -15.051059 19.576887 0.590268
C -15.254687 18.179651 0.458126
C -15.588029 17.665054 -0.809444
C -15.719418 18.499703 -1.906417
C -15.540488 19.88254 -1.766556
C -15.219217 20.409562 -0.526015
H -14.857894 17.2672 3.740047
H -14.453087 19.701895 3.991743
O -14.320475 21.368401 1.963965
H -15.109535 21.486499 -0.396067
H -15.662272 20.543718 -2.623052
H -15.972596 18.079356 -2.87865
H -15.738338 16.590366 -0.917619
K -12.339274 21.697201 -0.001846

K -13.868831 23.137377 3.961589
N -11.213698 24.418912 4.601369
C -10.59184 23.814143 3.821285
C -9.843688 23.053947 2.847234
H -10.130067 23.364973 1.836082
H -8.765436 23.198461 2.968522
H -10.058617 21.983291 2.939641
N -14.110153 24.025242 6.704764
C -13.034013 23.950465 7.146876
C -11.697743 23.849733 7.68851
H -11.198729 22.970325 7.269361
H -11.116803 24.733125 7.409135
H -11.729011 23.761575 8.778882
N -15.547564 25.503997 3.675676
C -16.178913 25.426087 4.652895
C -16.958833 25.303609 5.862602
H -16.422938 25.754042 6.702541
H -17.930753 25.792684 5.745317
H -17.107922 24.24146 6.081124
N -16.606328 22.222182 4.354889
C -17.1373 21.60718 3.519348
C -17.794911 20.848644 2.478149
H -18.876738 21.012072 2.507455
H -17.415559 21.153567 1.496758
H -17.591919 19.779489 2.602902
N -12.072345 21.106279 4.85896
C -11.774432 19.993648 4.679673
C -11.42711 18.610635 4.457055
H -10.789163 18.237089 5.264908
H -12.339356 18.002906 4.421653
H -10.90046 18.494516 3.499809
N -14.007488 23.568705 -1.6525
C -13.257846 23.87149 -2.492851
C -12.318489 24.248709 -3.524722
H -12.452409 23.61525 -4.406387
H -12.461198 25.295037 -3.812468
H -11.300042 24.110242 -3.147457
N -10.140529 23.530065 -0.857093

C -9.058036 23.904482 -0.635522
C -7.716309 24.346497 -0.331256
H -7.124517 24.444705 -1.246904
H -7.740321 25.317848 0.173281
H -7.240136 23.610633 0.330913
N -12.704785 24.201994 1.460487
C -13.425971 25.035556 1.08007
C -14.335056 26.05662 0.616583
H -14.915059 25.668866 -0.227099
H -15.011818 26.326288 1.434529
H -13.784468 26.945948 0.29469
N -7.154225 21.726789 1.566233
C -7.110847 20.574503 1.733439
C -7.037669 19.144686 1.935117
H -6.405222 18.912995 2.798266
H -8.037048 18.720787 2.096827
H -6.609027 18.664497 1.048474
N -11.575719 21.227804 -2.871915
C -10.962027 20.539937 -3.585268
C -10.225015 19.641356 -4.448232
H -10.906855 19.130174 -5.136526
H -9.474276 20.185929 -5.030225
H -9.710555 18.885979 -3.840967

TS_A-B_para_2Kcation_MicroSolvation_acetonitrile_conf3
C -12.2889446303 16.7072636238 0.5972691354
C -14.3543203643 17.1114260173 -0.3681489505
C -13.2433553029 13.6670512901 2.6938528178
C -12.585590814 14.0024861485 3.8529281724
C -11.8280664425 15.1648545604 3.9508783685
C -11.7347313324 16.047112334 2.9092930786
C -12.4028752489 15.75079906 1.6844150808
C -13.1322150669 14.5162175953 1.5649209995
C -11.5773963366 14.0126754446 5.8279966955
H -13.8408587316 12.7640210808 2.6132929352
H -11.1375363585 16.948801929 3.0018531963
H -10.6578887073 13.4058161219 5.8414703093
O -12.5689323321 13.3398275577 5.0462440638

O -11.3205045656 15.2719636194 5.2250005705
H -11.9604450094 14.1641957612 6.8420195041
N -13.8960043994 14.165016569 0.462582155
S -13.2625690268 13.7519853486 -0.9362403398
O -14.3326490322 13.9857097442 -1.9398172982
O -11.9248812703 14.2966382143 -1.2255651042
C -12.1448281621 20.1961039573 2.0089162004
C -11.395553867 20.8479758208 1.0330244069
C -12.3954168222 18.8343010225 1.8999451238
H -11.2162467399 21.9204893513 1.1046644181
H -13.0099758324 18.3419528255 2.6499216959
C -10.8916294855 20.1249991318 -0.0517423715
C -11.9085669351 18.0875725732 0.80732055
H -10.3096078693 20.6298701806 -0.8206264768
C -11.1535649123 18.7687969874 -0.1696411208
H -10.7826828408 18.2119740349 -1.0291295718
H -12.5736375633 20.7495018573 2.8435003079
C -14.0244679164 11.129839915 -1.4185462169
C -13.8468980712 9.7499301621 -1.3560622374
C -13.0457167839 11.9737754333 -0.8983599162
H -14.607232179 9.0918736102 -1.7782425116
C -12.7080542347 9.1901028671 -0.7686137116
C -11.8993663684 11.4376881183 -0.307658466
H -11.128103801 12.1063173837 0.0713624331
C -11.7396540442 10.0580717365 -0.2450205125
H -10.8381397906 9.6421180232 0.2052134421
H -14.9140191457 11.5568454262 -1.8790662077
C -12.532653084 7.7047177367 -0.6794570372
H -11.5293715408 7.394481272 -0.9939471164
H -13.258254612 7.171726564 -1.3026315715
H -12.6600533332 7.3450113425 0.3511426962
H -12.0554428005 16.289737274 -0.3799342886
H -14.3255984243 16.1218637967 -0.8116839289
C -15.1355284569 17.326599955 0.7871905518
C -15.4837437517 18.5854456197 1.2075912353
C -15.0943640972 19.7698210677 0.4746622477
C -14.3900721383 19.5427282307 -0.800964775
C -14.0647657504 18.2278739552 -1.2206555238

C -13.4135373274 18.04945149 -2.4571289175
C -13.0924452551 19.1401251133 -3.2495313791
C -13.4129308551 20.4395927709 -2.8288218513
C -14.0534905798 20.6337519296 -1.6146921354
H -15.4227144911 16.4611633882 1.3905533761
H -16.0723238957 18.7309076256 2.1128522853
O -15.3413992665 20.9249289401 0.8839385408
H -14.3108064932 21.6293193093 -1.2566420845
H -13.1525730943 21.292097422 -3.4539284503
H -12.5862523599 18.9876876639 -4.2018006764
H -13.1580206506 17.0355519273 -2.7722042355
K -16.6009637196 14.3562386838 -0.3658973028
K -15.895781554 21.7025960662 3.3974995801
N -13.3794649936 22.8399041822 4.2610780978
C -12.2291089312 22.9402857462 4.4112580597
C -10.796067897 23.0403477959 4.5822838004
H -10.3054395493 22.1596200126 4.1540102403
H -10.4097402858 23.9323620777 4.0794843178
H -10.5378703192 23.1002243913 5.6439509306
N -17.1860308056 21.9486520962 5.9889547434
C -17.541246412 22.4143995902 6.9951890746
C -17.9812526342 22.9962640408 8.2449530654
H -17.538541904 23.988011956 8.3793049837
H -19.0706842888 23.0991857129 8.2582869325
H -17.6811233011 22.367227292 9.0886755836
N -16.8587885836 24.36544319 2.9478667428
C -17.2092602353 25.4011326648 2.5488618174
C -17.6445909631 26.687921537 2.0506578255
H -17.1022245916 27.4962832018 2.550615923
H -17.4611000014 26.7616120656 0.97432675
H -18.7154235347 26.8256259482 2.2292928185
N -18.2157955229 19.9149998772 3.1694507151
C -18.282974873 19.2586694888 4.1311515762
C -18.3560387439 18.437926595 5.3189557483
H -17.5632547483 18.7288525375 6.0160078628
H -19.3250066937 18.560491113 5.8129648819
H -18.2304152939 17.3841697383 5.044439766
N -15.0023974803 19.1976888347 4.6304736025

C -14.9441625057 18.0365710785 4.5349455542
C -14.8850006184 16.6007665962 4.3806286844
H -14.3914873019 16.1339063481 5.2402233974
H -15.8937215984 16.1890086866 4.2570208723
H -14.3119987088 16.3519729312 3.4777526717
N -17.0688998935 16.5012647806 -2.189597824
C -16.8744458318 17.4776576431 -2.7949488951
C -16.6375211205 18.6916354291 -3.5442423033
H -16.135643445 19.434211929 -2.9116374337
H -15.9874404202 18.4885171531 -4.4009147625
H -17.5819901915 19.1095563872 -3.9069511003
N -19.4820962465 14.363832084 -0.0651685339
C -19.7220256491 13.2441021388 -0.2836783295
C -19.9895366006 11.8525266324 -0.5654900849
H -21.0358070998 11.7101885906 -0.8525091957
H -19.3391603328 11.5311889776 -1.3860070405
H -19.7721122986 11.2421796669 0.3160373669
N -17.4990863761 12.7210142485 -2.6588571169
C -16.7938188342 13.0838279606 -3.513750156
C -15.8996350695 13.5377253871 -4.5542934489
H -16.2928851463 14.4462615995 -5.0213315208
H -14.9263720519 13.7582650718 -4.1013666349
H -15.7817380879 12.7688635891 -5.3240595369
N -16.7466887769 11.7936799685 0.9071199206
C -15.8944970155 11.1104490085 1.3135661666
C -14.8415198861 10.2640768343 1.8303956748
H -14.9914406166 9.2285082678 1.5080843591
H -13.8683710412 10.6004279483 1.4501656377
H -14.8297049287 10.2939950605 2.9249996182
N -17.7654058531 16.2444803702 2.7351353556
C -18.2874311019 16.8468300199 1.8824958637
C -18.9135186482 17.588984941 0.8122025299
H -19.3860958038 16.8915164909 0.1122962671
H -19.6623609336 18.2769980801 1.2162376424
H -18.1523955092 18.1786138317 0.2879515892

TS_A-B_para_2Kcation_MicroSolvation_acetonitrile_conf4

C -13.141041 16.665637 1.095684

C -15.102365 17.54582 1.309644
C -11.18631 18.614843 3.644581
C -10.962952 19.762983 2.920609
C -11.312395 19.863711 1.576525
C -11.941269 18.846647 0.914497
C -12.25908 17.662829 1.638288
C -11.864167 17.550275 3.013557
C -10.725938 21.865216 2.27899
H -10.886712 18.512201 4.684173
H -12.287448 18.989571 -0.105117
H -9.869224 22.526033 2.113032
O -10.406631 20.942522 3.334691
O -10.977105 21.111641 1.110159
H -11.631273 22.425573 2.566891
N -12.210892 16.446185 3.788558
S -11.416678 15.090265 3.596672
O -12.155342 14.067393 4.388716
O -11.077137 14.713632 2.209502
C -12.330526 16.134656 -2.578189
C -13.302854 15.204629 -2.931416
C -12.258968 16.625612 -1.277786
H -13.356674 14.82527 -3.949732
H -11.455518 17.306395 -1.011772
C -14.193842 14.74347 -1.96114
C -13.168184 16.202049 -0.295819
H -14.944341 13.997928 -2.217872
C -14.120244 15.232732 -0.665997
H -14.819079 14.868053 0.089174
H -11.608391 16.475156 -3.317661
C -9.791923 15.96834 5.647925
C -8.593831 16.037308 6.351167
C -9.843893 15.276044 4.438339
H -8.554617 16.575717 7.298754
C -7.431935 15.42671 5.862601
C -8.699378 14.664585 3.931255
H -8.755244 14.138984 2.980536
C -7.50729 14.741489 4.643863
H -6.612216 14.263034 4.246155

H -10.695229 16.4416 6.034663
C -6.13515 15.528287 6.606209
H -5.566003 14.592833 6.562326
H -6.288394 15.781867 7.660694
H -5.491044 16.30747 6.177429
H -13.39794 15.887683 1.812234
H -15.611319 16.708613 0.833954
C -15.169826 17.64232 2.727444
C -14.756427 18.769719 3.383921
C -14.34533 19.946589 2.662817
C -14.6097 19.958038 1.214416
C -15.000628 18.769206 0.550162
C -15.170514 18.797042 -0.847077
C -14.977882 19.969847 -1.558147
C -14.601048 21.14693 -0.893323
C -14.413376 21.135105 0.478369
H -15.471998 16.757191 3.285653
H -14.717809 18.805282 4.473794
O -13.795521 20.929682 3.223048
H -14.086994 22.025701 1.012992
H -14.439103 22.063792 -1.459186
H -15.11445 19.975941 -2.638057
H -15.44621 17.878023 -1.365461
K -14.076904 15.592385 5.6548
K -14.812943 23.093207 4.425902
N -14.963896 24.485573 1.918867
C -15.860492 24.16588 1.245949
C -16.967495 23.751796 0.413086
H -17.03073 24.374463 -0.48478
H -16.82832 22.70672 0.114084
H -17.905735 23.828822 0.970496
N -15.565201 25.585555 5.589035
C -15.783187 26.70611 5.815641
C -16.053653 28.099285 6.095664
H -15.756023 28.722848 5.247055
H -17.12124 28.252022 6.28127
H -15.497592 28.427162 6.979294
N -17.086814 21.859876 3.123147

C -17.528111 20.780968 3.134711
C -18.0872 19.447684 3.147956
H -19.179473 19.494228 3.210464
H -17.805086 18.913566 2.234002
H -17.700205 18.874803 3.999734
N -15.665567 21.015743 6.253104
C -16.015653 20.149875 6.950422
C -16.439858 19.075007 7.818841
H -17.388077 19.327003 8.30419
H -16.570072 18.15638 7.234792
H -15.682167 18.894559 8.588047
N -12.057981 23.47307 5.21151
C -10.901627 23.39068 5.323315
C -9.466261 23.26671 5.453351
H -9.14047 22.3126 5.025077
H -8.961381 24.081253 4.924041
H -9.169714 23.306064 6.506163
N -17.020101 16.896204 5.342963
C -17.45747 15.819768 5.448767
C -17.961168 14.474481 5.59549
H -19.052692 14.456113 5.520507
H -17.534469 13.840903 4.810777
H -17.661354 14.087643 6.575302
N -15.760337 15.303878 8.039848
C -15.004132 15.639955 8.861424
C -14.058204 16.072665 9.864319
H -14.57743 16.501546 10.727323
H -13.447904 15.225931 10.189945
H -13.391048 16.821912 9.42525
N -15.378365 14.200341 3.40745
C -14.614059 13.574433 2.785179
C -13.678671 12.797434 2.004562
H -14.174642 12.366181 1.128824
H -12.837664 13.428566 1.686481
H -13.265328 11.993922 2.621309
N -12.313592 14.476363 7.751226
C -11.495857 13.755881 7.335769
C -10.490798 12.86011 6.809816

H -10.5218 11.896762 7.328525
H -10.685663 12.71303 5.741842
H -9.493231 13.29842 6.924545
N -13.378617 18.062083 6.972025
C -12.982522 19.095878 6.601385
C -12.522533 20.380524 6.129688
H -12.874177 21.175336 6.795511
H -11.428317 20.408987 6.088151
H -12.918905 20.56669 5.115327

TS_A-B_para_2Kcation_MicroSolvation_acetonitrile_conf5
C -12.741105609 16.2018176005 0.9266839498
C -14.8998409232 16.7252553365 1.5400229302
C -12.4631033318 18.1553788416 -2.2625095203
C -12.8526285074 17.1878582108 -3.1561950433
C -13.1152720817 15.8762940735 -2.7573749904
C -13.049877135 15.5011801572 -1.4452205664
C -12.6884900057 16.4793827454 -0.4717809313
C -12.3154493176 17.8056695064 -0.9014324002
C -13.3801958206 15.9997969633 -4.9690359928
H -12.2558670773 19.1771376538 -2.5660528921
H -13.2582088116 14.4772624469 -1.1528961153
H -12.627220912 15.6442283157 -5.6844258664
O -13.0011559197 17.3011833633 -4.5015252346
O -13.4376310132 15.123063887 -3.8543627267
H -14.3679438015 16.0642415678 -5.4439265468
N -11.8304312645 18.8138452012 -0.0773629553
S -10.4124347553 18.7353236813 0.6230118175
O -10.1254125953 20.1067881331 1.1189818874
O -10.2163079124 17.6631015873 1.6280083462
C -13.2255183751 12.5595477152 1.9159851248
C -12.2279375493 12.4327676788 2.8809179743
C -13.4184935285 13.7698382856 1.260839812
H -12.0786880862 11.4835854453 3.3911939014
H -14.2338128535 13.8613077474 0.5443869755
C -11.4335270594 13.5327591526 3.1961758056
C -12.6181930676 14.8858836797 1.5580094883
H -10.6534199766 13.4439529121 3.9500009507

C -11.6334012666 14.7485877273 2.5529021619
H -10.9993143296 15.6055441168 2.7766701469
H -13.8656229751 11.712160545 1.6786296803
C -8.2056228446 19.3088645369 -0.9558651709
C -7.2395731836 19.0010510468 -1.910131446
C -9.1775153237 18.3686440432 -0.6249921191
H -6.4690333684 19.7340580178 -2.1515429896
C -7.2318345226 17.7629518226 -2.5601271557
C -9.1753393675 17.116322767 -1.2459657724
H -9.9048745226 16.3607087683 -0.9572774802
C -8.2152663829 16.8280051176 -2.209471622
H -8.2141037321 15.8481531818 -2.6866395883
H -8.2059615642 20.271179924 -0.449759736
C -6.2159784985 17.4499794717 -3.6154274701
H -5.3312163545 18.0895154254 -3.5309135138
H -6.6270020301 17.5992785251 -4.6238589373
H -5.8842666952 16.4071490458 -3.5645161751
H -12.3723603511 17.016403676 1.5419595459
H -15.263608912 15.7256406527 1.3088200582
C -14.5943389654 17.0536737822 2.8840779373
C -14.3109315441 18.3438424166 3.2637650962
C -14.3039772253 19.4109936806 2.3005340647
C -14.9495640481 19.1266617296 1.0091451014
C -15.2354794776 17.7883216115 0.6385758157
C -15.8023136963 17.5462545454 -0.6270061978
C -16.112791959 18.5932458421 -1.4787456115
C -15.8681639682 19.918535312 -1.0913217625
C -15.2881336667 20.1752272455 0.141135446
H -14.4996407111 16.2415531729 3.6070867633
H -13.9995170246 18.5827771327 4.2789657717
O -13.7760322512 20.5276897311 2.5233066223
H -15.1249237467 21.2009225809 0.4745512001
H -16.1282970611 20.742793026 -1.7521264457
H -16.559788374 18.3859620115 -2.4500226993
H -16.0049394052 16.5169202973 -0.9250434698
K -12.3913926118 21.5642904889 0.4130720122
K -13.9157769995 22.4784176717 4.316761393
N -15.1586071175 26.6333703469 3.3782794143

C -16.0349700905 25.8629950401 3.3743622367
C -17.1124822581 24.8985059651 3.3564014202
H -16.9172211858 24.1309986705 2.5999650698
H -17.1803047496 24.3969386362 4.3278115581
H -18.0656550641 25.3889711682 3.1345121883
N -15.517082783 24.0869698649 6.1228664123
C -15.0855303864 25.1085099865 6.4828181057
C -14.5479409517 26.3800701625 6.9098771058
H -14.4992904695 27.0601600077 6.0526397522
H -15.176197816 26.8249850451 7.6876438397
H -13.5350590872 26.2407887549 7.2990162413
N -16.6014784753 21.4743925398 3.5037709505
C -17.1726195516 20.5377520897 3.1104859645
C -17.8718307315 19.3672320551 2.6239581495
H -18.8688718081 19.2987621929 3.070653405
H -17.9702772415 19.4090793969 1.5337621153
H -17.3044067896 18.4647052921 2.8789329224
N -12.0931015643 20.5054713326 5.4450885408
C -11.6753491805 19.469564689 5.110333086
C -11.1470054368 18.1955223223 4.6781934534
H -10.1935247253 17.9876537804 5.1761818944
H -11.8473971293 17.3888951303 4.9216629588
H -10.9790246128 18.1910631055 3.5908195974
N -12.4179885716 24.8996615048 5.0371887764
C -12.1698963509 25.4956071528 4.0662800207
C -11.8655431799 26.2261875256 2.8579981475
H -11.6825368876 25.521798643 2.0371442282
H -12.7146167833 26.8710971042 2.6055589319
H -10.9744695995 26.8468369403 2.9967028183
N -13.8341790587 22.930293639 -1.7478942072
C -13.116476733 23.541660295 -2.4343280173
C -12.2223156735 24.3019469123 -3.2785040872
H -12.5863707279 24.3222119568 -4.3104032733
H -12.1337363512 25.3285833762 -2.9097772457
H -11.2289414496 23.8425483962 -3.2542517039
N -11.1053083649 24.5454914321 -0.2301632685
C -10.1394661891 23.9186913335 -0.0418636661
C -8.9591332585 23.1186248269 0.1986193401

H -8.3518135254 23.0614904563 -0.7103778001
H -8.3597832424 23.5607366944 1.0015398715
H -9.2513701612 22.0987695268 0.4873628535
N -14.1763068417 23.7310855941 1.6759000973
C -14.2186951991 24.636649845 0.9430022436
C -14.2667783967 25.7812618313 0.0628637963
H -14.9152294828 25.5744785748 -0.7943640162
H -14.6530508533 26.6493084573 0.6086851683
H -13.2553844952 25.9976511219 -0.2990337211
N -11.1249061032 22.7650329729 2.7833647646
C -10.2734295741 22.134569726 3.2709231397
C -9.2029439889 21.3515063491 3.8446590295
H -9.1597079718 20.387252849 3.3249040862
H -8.2430670516 21.8630893882 3.7161990262
H -9.3781250095 21.1967895472 4.9136913434
N -10.567550328 21.5511212577 -2.1271928515
C -10.2621443375 20.7428643402 -2.9110008675
C -9.9062051838 19.7289241923 -3.8802259127
H -10.6884805601 19.6363020116 -4.6417669693
H -8.9592264521 19.978541921 -4.3697249509
H -9.7930156049 18.7608891495 -3.3752001426

TS_A-B_para_2Kcation_MicroSolvation_acetonitrile_conf6
C -12.1886597931 16.432935148 1.1628125115
C -14.3630290777 17.2203725787 1.399098941
C -12.8142335971 12.7406510651 0.9322385062
C -12.0650377646 12.3066196157 2.0003896836
C -11.4069308396 13.1948806911 2.8490298504
C -11.4580485327 14.551928144 2.6570540811
C -12.2030924456 15.0484493635 1.5500279613
C -12.9033382367 14.1302613375 0.6924827394
C -10.8962240789 11.1253444041 3.4752145597
H -13.3268991575 12.0536037007 0.2651163146
H -10.8805318978 15.2177611834 3.2898195582
H -9.9344722345 10.7602710855 3.0832773918
O -11.8871935813 11.0273294069 2.4448517201
O -10.7814632889 12.4902753114 3.8473980529
H -11.2165988875 10.5346074482 4.3392338036

N -13.7501736949 14.5947948581 -0.305079558
S -13.2521343772 14.6558169007 -1.8140483704
O -14.3958483652 15.2207289306 -2.5741747048
O -11.9310645384 15.2743938603 -1.9913671408
C -10.6645196205 18.8306088858 3.6686226382
C -10.1626580272 19.800332166 2.803683642
C -11.3219135783 17.7148997007 3.1647907005
H -9.6473692764 20.6726695657 3.2026988426
H -11.761245923 16.9992221043 3.8562025432
C -10.3333226897 19.6554901741 1.4263185911
C -11.4744751148 17.5292843692 1.7744018483
H -9.9617109266 20.4176969546 0.743868275
C -10.988206858 18.5428905363 0.9215255929
H -11.1355993676 18.4323224282 -0.1530019089
H -10.5630258964 18.9537201916 4.7451148794
C -14.2204701578 12.2598201503 -2.7924894066
C -14.1055615168 10.9283407094 -3.1738517837
C -13.0829680005 12.9742109045 -2.4137443652
H -14.9959963282 10.3767069339 -3.4806514029
C -12.8626491156 10.2784880246 -3.1685979972
C -11.8386565581 12.3478503408 -2.4014628609
H -10.9594796417 12.9192723052 -2.1102400214
C -11.7376523692 11.0097833997 -2.7722890618
H -10.7628284702 10.5224313301 -2.7637675841
H -15.19312473 12.7517725771 -2.7909156562
C -12.7443848847 8.8479661247 -3.5987973289
H -12.6740598627 8.7650137696 -4.6914277421
H -13.6165999879 8.2561783863 -3.2956891663
H -11.8500063177 8.3704504192 -3.1848587313
H -12.3824532967 16.5678527902 0.1002483056
H -14.6571317215 16.2977030695 0.9057823265
C -14.4932586976 17.3447868529 2.7967781325
C -14.3197198558 18.5456983781 3.436520522
C -14.053750477 19.7615366869 2.7078354697
C -14.0200343894 19.6578881698 1.2392322669
C -14.2098559884 18.4052100602 0.6018466284
C -14.1445584334 18.3413973922 -0.8070329183
C -13.9001778436 19.481186316 -1.5527700569

C -13.7252437538 20.7225741199 -0.9176980325
C -13.7934872493 20.804874794 0.4617673736
H -14.6889002761 16.447164214 3.386547153
H -14.3796152385 18.6233278711 4.5219629707
O -13.8731143118 20.8553306412 3.2924045367
H -13.6722977752 21.7531836275 0.9840231502
H -13.5338082003 21.6145715031 -1.5124137918
H -13.84146178 19.4107985316 -2.6374986015
H -14.2665922368 17.3761857288 -1.3034621233
K -16.5136895679 14.9907276036 -0.8161947412
K -16.0889397969 21.5715231067 4.7830653951
N -17.6003249676 19.1056806962 4.3467048104
C -17.6711819178 18.5884413521 3.30454255
C -17.7501267969 17.9638452166 2.0018420242
H -16.922989525 18.3103720062 1.3699773069
H -17.6721006552 16.8725761546 2.0833323025
H -18.6944315725 18.2122722725 1.5053455681
N -17.9156502767 23.3678356123 6.0483257524
C -18.4348722652 24.3463975886 6.4045811547
C -19.0784272474 25.564496219 6.845937784
H -19.9037032721 25.8251818191 6.1762298218
H -19.4782697364 25.4425038138 7.8572791978
H -18.3621962102 26.3918710393 6.852256171
N -14.632800373 20.8605285534 7.1417068793
C -14.0432941686 21.0045462063 8.134860162
C -13.3123979266 21.1864059502 9.3703075234
H -13.9168527825 20.8638566178 10.2236622404
H -12.3875477165 20.6015299831 9.3596162587
H -13.0539511437 22.2409385248 9.5074997005
N -14.3148516594 23.8787803889 4.7685507044
C -13.356861187 23.6811234727 4.134493993
C -12.1723445931 23.4141731918 3.3488665979
H -12.1411359729 24.0621258713 2.4669567318
H -11.2684527444 23.5903506152 3.9408091503
H -12.2065549498 22.3659493756 3.0286399926
N -16.921134924 21.5643437977 2.0462797101
C -17.0063031821 21.2186447658 0.9369781469
C -17.1201604301 20.7712787341 -0.4331567241

H -16.5392214173 19.8536758936 -0.5892012352
H -18.1651635567 20.5587308533 -0.6816059279
H -16.7406932565 21.5364959954 -1.1174622326
N -17.7229762497 17.586659562 -1.3741580263
C -18.6909036468 17.2523457638 -1.9331950662
C -19.8810434605 16.8101460437 -2.6231917344
H -20.1817817467 17.5363502661 -3.3845819765
H -19.6732761435 15.847404113 -3.1025561188
H -20.6972612443 16.671345644 -1.9085709686
N -19.3738506058 14.8469102812 -0.0761158022
C -19.4049720489 13.9848738031 0.707895755
C -19.425172158 12.9110359472 1.6755288305
H -19.4026472437 13.3160242519 2.6918543462
H -20.325749054 12.300967219 1.5560476078
H -18.5420942341 12.2800935189 1.5284907221
N -17.5461345532 14.5213849084 -3.4927393253
C -16.7152195289 14.5148598101 -4.311352617
C -15.6670913074 14.5059858953 -5.3064414664
H -15.9335409224 15.1430962905 -6.155274249
H -14.7487943542 14.877894275 -4.8382555819
H -15.4942127975 13.4882451703 -5.6710911728
N -16.4694990531 12.0730818302 -0.1023061638
C -15.8107147475 11.1172530866 0.0055446044
C -14.9807917907 9.9374968762 0.129680185
H -14.4534452938 9.9314007824 1.090225051
H -15.5874999975 9.029263919 0.0560818649
H -14.2349310149 9.9264222412 -0.6756000963
N -16.558036552 14.5908996975 2.0718428825
C -15.8082751574 14.0254573993 2.762085721
C -14.8756777656 13.3444988945 3.6304303415
H -13.8983606469 13.8436295343 3.5953340137
H -15.2398321507 13.3494747347 4.6628937598
H -14.7371196042 12.3071457171 3.306128162

TS_A-B_para_2Kcation_MicroSolvation_acetonitrile_conf7

C -13.3479861917 16.7802808238 1.296424868
C -15.2687855893 17.8461356962 1.000453508
C -11.6973867453 18.8227201113 3.9692770484

C -11.1003975937 19.7847729172 3.1914574182
C -11.1896735476 19.7770712638 1.8016859724
C -11.8491198201 18.7892915769 1.1286945517
C -12.5148899321 17.7841064791 1.8859238979
C -12.4415986586 17.8140854774 3.3247356752
C -10.2567911196 21.6774153448 2.441428796
H -11.6124383905 18.8220559347 5.0533171892
H -11.9035949107 18.8149606373 0.0467575258
H -9.2488413188 22.09510425 2.3624399499
O -10.3290658813 20.8368606185 3.5896709259
O -10.5299578168 20.8749663558 1.2987618014
H -11.0237796966 22.4644311149 2.5377162044
N -13.0927241818 16.8565110748 4.116372812
S -12.2221790203 15.5638635482 4.4505697623
O -13.0595977218 14.6666623714 5.2802099777
O -11.5580398858 14.9556942187 3.2799446754
C -12.6327953607 15.8795833179 -2.3464050219
C -12.8309627873 14.5019407744 -2.2976761008
C -12.7887162086 16.6602408664 -1.2058348349
H -12.6984200217 13.8962339553 -3.19184943
H -12.6470463898 17.7320659482 -1.291882125
C -13.1897236729 13.9071372083 -1.0893470338
C -13.1599013957 16.0821754424 0.0212481548
H -13.3270340066 12.8281575289 -1.0300607571
C -13.3591122296 14.6871802991 0.0474470747
H -13.6062749327 14.2184594158 1.0004838931
H -12.349208675 16.3555621223 -3.2833479445
C -11.2021061132 16.5081341372 6.8296703603
C -10.2131738646 17.0697550719 7.6296046988
C -10.8968853553 16.1160418379 5.525398556
H -10.4492687604 17.3649079371 8.6535083359
C -8.9118873171 17.2625791519 7.1443514887
C -9.6111936651 16.3003781788 5.0228984675
H -9.3954933023 16.0012146598 3.9989451494
C -8.6315619913 16.8713191874 5.8308630164
H -7.6267187637 17.0179001224 5.434305563
H -12.2182206905 16.3831370784 7.2061630458
C -7.8476370044 17.8606967999 8.0136977684

H -7.2128592817 17.0844168995 8.4607636463
H -8.2741985613 18.4378051087 8.8422897763
H -7.1810202044 18.521134977 7.4463997374
H -13.7685265813 16.1221023768 2.0548552679
H -15.7641767085 16.9936746802 0.5349329521
C -15.5581973099 18.1363784702 2.3631147868
C -15.090598899 19.2756755319 2.9615118951
C -14.3580928682 20.2723203355 2.2154743898
C -14.4361322998 20.1488439232 0.7463335089
C -14.9148286739 18.9550740744 0.1494162319
C -15.0150914275 18.8988220278 -1.2539130793
C -14.6580630124 19.9859626933 -2.0355839217
C -14.1822293746 21.1625175879 -1.4372043757
C -14.0713501035 21.2355117228 -0.0584342393
H -16.0729595393 17.3802500857 2.9538882144
H -15.2162264959 19.4438929799 4.0286392286
O -13.7298062106 21.2097971759 2.7517144478
H -13.7225858342 22.1340335711 0.4500529523
H -13.9138525336 22.0145679842 -2.0603472439
H -14.7556034346 19.9291607234 -3.1182915296
H -15.3830800241 17.9850422403 -1.7209872395
K -15.5763515494 15.6366309138 5.1490468366
K -14.2945901996 23.215115723 4.4492200634
N -16.2232555749 14.8123927805 2.4695823638
C -16.4292619867 14.3290172024 1.4300348409
C -16.6820731325 13.7332118613 0.1362791203
H -16.1611916318 14.2951061179 -0.646856178
H -16.3145059437 12.7021223107 0.11528039
H -17.7549949926 13.7251937081 -0.0795410561
N -16.3041974967 13.0250853899 5.9569540229
C -16.3027868814 11.877783956 6.1576335236
C -16.2811482344 10.4549641856 6.4097887255
H -16.9025588032 9.926509316 5.680206917
H -15.2469271295 10.1026611873 6.3269283215
H -16.661686448 10.2367393053 7.4125074574
N -13.1396581593 11.0815493192 5.8318679722
C -12.1777454101 11.583598066 5.4062829029
C -10.9750565754 12.1990264409 4.8933367021

H -10.271199636 11.4342886191 4.5493906233
H -11.2099745941 12.8823745082 4.0683783887
H -10.4912557435 12.7888608698 5.6804474424
N -14.777945138 15.8088659155 7.9619189301
C -14.1671320568 14.8188468211 8.0498126981
C -13.4012881737 13.5982163554 8.1457288602
H -13.4944271396 13.1594341011 9.1440184457
H -13.7459360605 12.8784982155 7.3946856606
H -12.3462857991 13.8088738704 7.938431077
N -17.160379169 17.9627404341 5.4172660099
C -17.6765210859 19.0036777336 5.3349176915
C -18.2990966159 20.3021434637 5.2225707993
H -19.0098062296 20.3158867286 4.3896806855
H -18.835995078 20.5479573804 6.1441310119
H -17.5294280108 21.0636140523 5.0551421223
N -12.9822546391 24.4347483063 2.16190854
C -12.3757679139 24.2648285453 1.1819326237
C -11.6167239718 24.0205052434 -0.0264155752
H -11.3721826043 22.9542164111 -0.1055546132
H -12.195407963 24.313316958 -0.9084412393
H -10.6858081984 24.5967082298 -0.0198178345
N -14.8773017876 25.7752326161 5.5840705334
C -15.0923598062 26.8481581019 5.9802078428
C -15.3597658379 28.1818319597 6.4724694484
H -15.3001198172 28.205218558 7.5649111903
H -14.6301306723 28.8909804464 6.0696907286
H -16.3608942996 28.5062407731 6.172418343
N -16.6494110377 22.5855895202 2.8609552627
C -17.0906378583 21.9088215219 2.0213860987
C -17.6429067845 21.0483067845 0.9973561795
H -17.1589371654 21.24092441 0.0338586253
H -17.4606463009 19.9994846723 1.2635413121
H -18.7202393566 21.2137549195 0.8927672854
N -15.2591431475 21.0719537107 6.129199402
C -14.7216597809 20.0780369114 6.4193405953
C -14.0651735612 18.8374228035 6.7578950665
H -13.8280461119 18.2570356186 5.8488338833
H -13.127522198 19.0384821598 7.2898390221

H -14.6995808867 18.2189317857 7.4033429837
N -12.0423034254 22.2648917448 5.9687348495
C -11.1556580794 21.6050197692 6.3353915529
C -10.0512316972 20.778919622 6.7735000237
H -9.4125025853 20.5364356933 5.91733945
H -9.454163523 21.2982810232 7.5300874332
H -10.4117113997 19.8359983014 7.2026753219

TS_A-B_para_2Kcation_MicroSolvation_acetonitrile_conf8
C 1.796494 0.351617 -0.298316
C 3.360011 -0.478579 -1.584511
C -1.307442 0.744894 -2.383407
C -1.229415 2.086555 -2.658682
C -0.209204 2.887836 -2.15375
C 0.780676 2.362729 -1.370219
C 0.759101 0.969948 -1.079581
C -0.314403 0.144036 -1.572151
C -1.601136 4.190018 -3.312327
H -2.092937 0.115608 -2.786679
H 1.555415 3.002371 -0.95998
H -2.353376 4.80262 -2.795394
O -2.086264 2.84161 -3.406872
O -0.392114 4.18169 -2.573436
H -1.416223 4.574964 -4.323067
N -0.429745 -1.22694 -1.417638
S -0.717311 -2.007027 -0.066414
O -0.879449 -3.422252 -0.471756
O 0.189085 -1.753144 1.0717
C 4.200142 2.740531 1.434272
C 4.12392 2.256207 2.740229
C 3.456037 2.144803 0.423534
H 4.706273 2.725938 3.53036
H 3.557593 2.503877 -0.600969
C 3.312747 1.160393 3.023398
C 2.619608 1.050059 0.696671
H 3.257539 0.769604 4.037695
C 2.577157 0.552611 2.010716
H 1.94306 -0.308444 2.220697

H 4.850057 3.581991 1.201844
C -3.458729 -2.242594 0.29979
C -4.690064 -1.817944 0.795647
C -2.32131 -1.475462 0.536898
H -5.57631 -2.430154 0.624166
C -4.810952 -0.628085 1.519665
C -2.418975 -0.279353 1.252975
H -1.519466 0.2999 1.459401
C -3.655275 0.136927 1.733233
H -3.72612 1.064382 2.302055
H -3.373892 -3.17502 -0.256714
C -6.137686 -0.170464 2.045039
H -6.425702 0.803947 1.627663
H -6.120011 -0.045816 3.134729
H -6.93547 -0.882157 1.807855
H 1.533238 -0.649116 0.020175
H 4.103527 0.250558 -1.266386
C 2.831185 -0.370273 -2.899014
C 2.181611 -1.405963 -3.513266
C 2.031475 -2.683695 -2.867564
C 2.662196 -2.851919 -1.553063
C 3.39849 -1.786346 -0.969493
C 4.030618 -2.001234 0.271235
C 3.891567 -3.207805 0.93768
C 3.118833 -4.239346 0.385276
C 2.51825 -4.057529 -0.848395
H 2.888326 0.598435 -3.399499
H 1.706883 -1.283352 -4.485716
O 1.394724 -3.628669 -3.405365
H 1.880441 -4.832648 -1.273243
H 2.980168 -5.172438 0.928201
H 4.369492 -3.346795 1.906287
H 4.600252 -1.187731 0.721394
K -1.335266 -3.328951 -3.182748
K 2.366392 -6.134442 -3.816967
N 4.553229 -6.776558 -2.029838
C 5.195291 -5.839601 -1.766834
C 5.989428 -4.674306 -1.449004

H 5.356499 -3.778151 -1.477424
H 6.410487 -4.759498 -0.44243
H 6.808197 -4.56549 -2.168174
N 2.909382 -8.788554 -4.886189
C 3.0081 -8.899399 -6.042677
C 3.109909 -9.016117 -7.479072
H 4.158162 -9.006613 -7.793065
H 2.651703 -9.947991 -7.824738
H 2.591743 -8.16546 -7.934744
N 1.229891 -8.073317 -1.920719
C 2.116185 -8.814965 -1.764522
C 3.221458 -9.729237 -1.597298
H 3.726724 -9.851155 -2.560769
H 3.93949 -9.322937 -0.879644
H 2.868449 -10.703513 -1.245684
N 1.456984 -6.238994 -6.760326
C 0.991699 -5.171617 -6.669836
C 0.426988 -3.857178 -6.48509
H 0.735997 -3.180495 -7.287872
H -0.669295 -3.90779 -6.462909
H 0.77448 -3.454414 -5.521076
N 4.370927 -4.224614 -4.515829
C 4.897582 -3.191352 -4.403667
C 5.574501 -1.922346 -4.249441
H 6.644938 -2.031033 -4.453132
H 5.156613 -1.181803 -4.938049
H 5.43772 -1.54619 -3.228509
N -0.70016 -6.062536 -3.99593
C -0.721179 -7.138618 -4.442704
C -0.702501 -8.469406 -5.005407
H -0.282745 -8.431667 -6.016713
H -0.07664 -9.123011 -4.388358
H -1.714142 -8.88377 -5.053087
N -2.940749 -4.004544 -5.727381
C -3.969031 -4.218446 -5.220773
C -5.233157 -4.484082 -4.572286
H -5.782354 -5.26731 -5.103919
H -5.038352 -4.808171 -3.543409

H -5.840935 -3.574938 -4.545626
N -3.318635 -5.340151 -1.960882
C -2.509659 -5.82057 -1.270816
C -1.487518 -6.39828 -0.429049
H -0.731798 -6.905928 -1.041838
H -1.003283 -5.592096 0.133286
H -1.924951 -7.122263 0.265709
N -3.977336 -1.777866 -3.168459
C -4.312833 -0.851181 -2.544196
C -4.680761 0.330576 -1.796296
H -4.589721 1.220802 -2.429813
H -5.708942 0.255368 -1.428235
H -4.009959 0.442988 -0.934744
N -0.870531 -1.253679 -5.216868
C -1.814831 -0.627999 -5.490125
C -2.991404 0.148345 -5.812971
H -2.976989 0.451039 -6.864638
H -3.88882 -0.451978 -5.630057
H -3.031775 1.05075 -5.191219

TS_A-B_para_2Kcation_MicroSolvation_acetonitrile_conf9
C 2.6355627402 0.1700564488 0.5465032626
C 4.3276422648 0.1325229645 -0.8711297315
C 1.5223638942 -3.4018360007 0.228472939
C 0.5938499152 -3.2610844596 -0.7747784629
C 0.2093755997 -2.0096475996 -1.2517074131
C 0.8134095302 -0.8600557052 -0.8263600441
C 1.8267379605 -0.9519301884 0.1705649338
C 2.1101770517 -2.2358161182 0.7648027526
C -1.1478917508 -3.5193966964 -2.1218015795
H 1.8154992191 -4.3698007981 0.6221581251
H 0.523162505 0.0977192596 -1.243161228
H -2.0578461655 -3.5714296575 -1.5022351514
O -0.0973971503 -4.2360653313 -1.4487843368
O -0.7280188282 -2.1707668906 -2.2488158416
H -1.3009136844 -3.9585850651 -3.110448728
N 2.9524788506 -2.3991426428 1.8603527922
S 2.4204361053 -1.8856712969 3.272122075

O 3.4737521528 -2.1958656249 4.2637675869
O 1.8996352829 -0.4984140873 3.3002498025
C 1.2800282311 3.6059018818 -0.4228615796
C 1.4966345826 4.2990055444 0.766078949
C 1.6411888693 2.2684366247 -0.534478377
H 1.2135113535 5.3461362847 0.8492915237
H 1.4970339636 1.7662080191 -1.487676702
C 2.0855051999 3.6410547559 1.8423323179
C 2.2304777513 1.5844232877 0.5421595123
H 2.2587466553 4.1692457588 2.777924109
C 2.4542406499 2.3062171485 1.7279930452
H 2.8775215155 1.7816673067 2.5824606405
H 0.8329148346 4.1129778063 -1.2756992433
C 1.1694906279 -3.9331045601 4.646429877
C 0.0728855535 -4.7176527272 4.9923915963
C 1.0139711879 -2.8995998393 3.7239817643
H 0.1921611057 -5.5113378442 5.7305686115
C -1.1835715379 -4.5022740832 4.4152182478
C -0.2310937715 -2.662718616 3.138197582
H -0.3536457107 -1.8332009061 2.4424605262
C -1.3143731136 -3.4647317121 3.4824738124
H -2.289248274 -3.2706518015 3.0349912822
H 2.1451353 -4.1003555539 5.1002813545
C -2.3507074001 -5.3753399729 4.7614296971
H -3.2946396099 -4.8197661249 4.7455819389
H -2.2413842916 -5.8268061032 5.7531063077
H -2.4593198036 -6.2012570381 4.0438696697
H 3.3125479819 -0.0682512223 1.3635808383
H 4.8187849824 1.0019409011 -0.4327880052
C 3.6365199047 0.2980131063 -2.0995234369
C 3.1460084195 -0.7807191057 -2.7856395819
C 3.3772263821 -2.1291408182 -2.3354022198
C 4.3964736238 -2.2958320162 -1.2867426025
C 4.909879344 -1.1614071889 -0.6090110682
C 5.9729951134 -1.3365322348 0.2956962601
C 6.5331662007 -2.5843920393 0.5003831939
C 5.9904538669 -3.7140479545 -0.1362085408
C 4.9193310102 -3.5678229338 -1.0004549983

H 3.4265584571 1.3073892701 -2.4492799464
H 2.533260343 -0.6508945594 -3.6773506402
O 2.7917834499 -3.1147560775 -2.8540669809
H 4.4767338255 -4.427347859 -1.502100368
H 6.4269734473 -4.694869823 0.050199602
H 7.387012619 -2.6907167347 1.1692108157
H 6.3844438894 -0.465473272 0.806550083
K 5.1244654526 -3.8930205097 2.9872286678
K 1.8609199221 -5.566245727 -3.2367707268
N 2.5080191981 -4.2213509626 -6.0171578464
C 1.7030554131 -3.4308736119 -5.7200180817
C 0.6949220084 -2.4780362106 -5.3141618994
H 0.7514992529 -2.3378425435 -4.22763097
H 0.8538914056 -1.51192083 -5.8039693417
H -0.3020478517 -2.8481117331 -5.5736214861
N 2.0115462929 -7.6375252087 -5.2940713631
C 2.9402133129 -7.4113709183 -5.9620169441
C 4.0883204117 -7.1138213195 -6.7871257174
H 4.0912000411 -6.0419549268 -7.0133812578
H 5.0086636937 -7.3688191651 -6.2531185397
H 4.0498476016 -7.6806121307 -7.7224758961
N 4.7400883483 -5.9328963677 -3.7130965939
C 5.0864540145 -4.8827975143 -4.0866074808
C 5.5119871681 -3.5800354969 -4.5442850509
H 4.6721334804 -3.0782079562 -5.0358720277
H 5.8365016651 -2.9730782266 -3.6912321547
H 6.3424097524 -3.6717185168 -5.2514450376
N 2.5486688269 -6.7491605567 -0.7860104937
C 3.660331751 -7.0098008192 -0.5487859031
C 5.0394715207 -7.323544568 -0.2624433292
H 5.6729108298 -6.9838754554 -1.089450489
H 5.361819813 -6.8327899144 0.6641799146
H 5.1687869936 -8.4043911131 -0.1474722787
N -0.607747123 -5.5148577648 -4.7574011567
C -0.8269230756 -6.3957908229 -5.4882245105
C -1.0852415245 -7.4973612252 -6.3859223742
H -1.0017427969 -7.1692486131 -7.4266046043
H -0.3456049328 -8.2840944379 -6.2056176513

H -2.0898901522 -7.8998911113 -6.2247201661
N 6.8429311652 -6.1668740089 2.4174628429
C 6.7585620183 -6.8279861869 3.3745609041
C 6.6310805528 -7.629566288 4.5696242945
H 5.8050352918 -8.3397290383 4.4619872441
H 7.5533315223 -8.1852291587 4.7644976477
H 6.4198884298 -6.9665506112 5.4158059101
N 6.0619233293 -4.5476852745 5.6229696789
C 6.6088381658 -3.6547732144 6.1374353239
C 7.2943200547 -2.5552645892 6.7744468247
H 7.6610298373 -2.8554965964 7.7611511627
H 8.1395551207 -2.2404603638 6.1549621814
H 6.6096209794 -1.7043265515 6.8857734189
N 6.7508137318 -1.600607322 3.7228548075
C 6.0837307841 -0.644150454 3.750152229
C 5.259131835 0.5425186789 3.7506777894
H 5.4812992071 1.155773951 4.629192648
H 5.4347456969 1.1321850908 2.8427803879
H 4.2022283527 0.2483871534 3.7931284197
N 4.8384154749 -0.3408862437 6.8107798344
C 3.6830724651 -0.4730348465 6.7308117195
C 2.2467716755 -0.6108530474 6.636718503
H 1.9672589852 -1.6670094592 6.7046166782
H 1.7548405484 -0.0587209699 7.4439026928
H 1.8955536454 -0.2352739165 5.6680563956
N 3.2952753483 -6.0730881061 2.6413960245
C 2.1623497133 -6.2041307843 2.3987435444
C 0.7647560999 -6.3555747467 2.0593191297
H 0.6776736742 -6.6694158737 1.0124043967
H 0.2882672347 -7.1017090187 2.7037487941
H 0.2439281239 -5.3984664102 2.190967742

TS_A-B_para_2Kcation_MicroSolvation_acetonitrile_conf10

C -13.29752 16.123353 0.032472
C -15.199032 17.340648 -0.235368
C -10.382835 17.560806 -1.800334
C -10.267492 16.625692 -2.80089
C -11.204691 15.608656 -2.992698

C -12.267369 15.443509 -2.149296
C -12.408681 16.355987 -1.064737
C -11.501093 17.471733 -0.939796
C -9.589421 15.354969 -4.497709
H -9.668738 18.368484 -1.683897
H -12.933858 14.596971 -2.269211
H -8.819878 14.613377 -4.235658
O -9.325825 16.569652 -3.785321
O -10.871063 14.886193 -4.109731
H -9.583251 15.560885 -5.573293
N -11.784748 18.457022 -0.007839
S -10.730575 18.799366 1.128885
O -9.303031 18.736656 0.729202
O -11.167587 20.069897 1.757532
C -15.215462 12.843735 -0.136889
C -15.061018 12.39641 1.175087
C -14.659441 14.052222 -0.535762
H -15.49729 11.448841 1.4839
H -14.833679 14.408636 -1.550449
C -14.364691 13.181273 2.0907
C -13.92689 14.843732 0.366636
H -14.255619 12.850342 3.122263
C -13.816038 14.395426 1.693879
H -13.275011 15.013478 2.411226
H -15.78324 12.250722 -0.851028
C -10.170552 16.366789 2.303036
C -10.388842 15.350217 3.22621
C -10.924148 17.538445 2.380473
H -9.803638 14.433048 3.162629
C -11.352146 15.479252 4.234213
C -11.899281 17.683703 3.366313
H -12.493053 18.594285 3.411851
C -12.100154 16.662311 4.288007
H -12.864763 16.779383 5.057167
H -9.414366 16.264904 1.526247
C -11.579256 14.37528 5.221133
H -10.837641 14.398841 6.030713
H -11.497217 13.389206 4.749204

H -12.567895 14.445599 5.688242
H -13.031873 16.719533 0.902257
H -15.708461 16.421446 -0.513791
C -15.256013 17.775946 1.109972
C -14.92565 19.058439 1.47354
C -14.589052 20.048911 0.495623
C -14.716865 19.665742 -0.916025
C -14.996377 18.319422 -1.265377
C -15.072155 17.977301 -2.628553
C -14.914769 18.938279 -3.61339
C -14.661513 20.272133 -3.263836
C -14.559068 20.62531 -1.92772
H -15.487883 17.038686 1.882328
H -14.869074 19.339292 2.526013
O -14.186577 21.20423 0.816439
H -14.378845 21.664001 -1.647652
H -14.552525 21.030928 -4.037352
H -15.004394 18.658166 -4.66258
H -15.283504 16.942429 -2.901008
K -11.793588 21.635641 -0.400172
K -13.03359 21.670439 3.201286
N -13.168182 23.978061 -1.342771
C -12.577471 24.871317 -0.881307
C -11.839002 25.963967 -0.291764
H -12.132307 26.91648 -0.743884
H -12.045778 25.997559 0.784381
H -10.768273 25.805549 -0.459215
N -9.321938 23.353403 -0.780656
C -8.621852 22.41939 -0.77263
C -7.783321 21.242732 -0.767368
H -7.575582 20.926592 -1.794933
H -6.838877 21.455229 -0.256888
H -8.284322 20.421542 -0.234036
N -11.038089 23.355995 2.081872
C -10.078017 24.015051 2.045525
C -8.883581 24.827638 2.013883
H -8.76327 25.278407 1.024103
H -8.932372 25.619813 2.768584

H -8.018522 24.183358 2.206842
N -7.919875 21.794557 2.631548
C -8.046891 20.707529 3.034536
C -8.18833 19.36907 3.561173
H -7.511231 19.220308 4.408805
H -9.219791 19.217224 3.898903
H -7.97052 18.63655 2.77868
N -10.875569 20.724874 -2.899081
C -11.210943 19.941694 -3.693751
C -11.619617 18.958656 -4.672069
H -12.106755 19.443807 -5.524297
H -10.7487 18.398595 -5.029019
H -12.328869 18.259763 -4.212553
N -13.965565 24.819977 2.175624
C -14.80791 24.40369 1.4829
C -15.846921 23.880292 0.625374
H -15.89205 24.447978 -0.309075
H -15.610583 22.834292 0.395875
H -16.816168 23.930237 1.131108
N -13.064065 23.481384 5.476491
C -13.956293 24.217606 5.326074
C -15.068706 25.120518 5.140954
H -14.758711 25.971755 4.528324
H -15.869428 24.590681 4.61432
H -15.440101 25.478366 6.106226
N -16.015405 22.231 3.569215
C -16.627872 21.240785 3.632155
C -17.374575 20.00577 3.722819
H -17.573085 19.606177 2.723222
H -16.783275 19.271454 4.280725
H -18.326022 20.167091 4.239259
N -14.273388 19.6957 5.061068
C -13.665257 19.808317 6.049821
C -12.909226 19.943623 7.273685
H -12.67869 20.999271 7.448506
H -13.478708 19.55367 8.12305
H -11.96466 19.398438 7.184732
N -10.831002 20.845705 5.102119

C -10.293212 21.875797 5.203831
C -9.63314 23.15575 5.326933
H -8.801196 23.208807 4.615639
H -10.352772 23.95397 5.11592
H -9.245825 23.291884 6.341605

TS_A-B_para_2Kcation_MicroSolvation_acetonitrile_conf11
C -12.9652234695 16.4934465475 0.6703103764
C -14.5719992761 18.0285630811 0.8130523907
C -14.7455856144 13.2346639543 1.1322082939
C -14.1661400037 12.8203148167 2.306349012
C -13.2738690114 13.6182790179 3.0253919402
C -12.8781861177 14.847288117 2.5730019466
C -13.4211734831 15.3103767115 1.3439755697
C -14.4066039827 14.52022088 0.6513097188
C -13.4554197234 11.6643439793 4.0670880176
H -15.4604853554 12.6232782756 0.5923200931
H -12.1293443467 15.4161313514 3.1127762608
H -12.6460595187 10.9538514672 3.8377763279
O -14.3975252738 11.6627039676 2.991081023
O -12.9299090698 12.9770004023 4.1908788623
H -13.9676459113 11.3857010687 4.9940622769
N -15.0621634681 15.1007242521 -0.417900333
S -15.0851159163 14.4221529522 -1.8556712545
O -15.333307705 12.9593024884 -1.8793482941
O -15.9935032423 15.2524548123 -2.6774000862
C -10.1790145593 18.4374859324 2.3728376949
C -9.4503093576 18.845585118 1.258552862
C -11.3366483324 17.6802021396 2.2235647814
H -8.5481578071 19.4413884246 1.3833215721
H -11.9235698343 17.4235774242 3.1029097158
C -9.8880225361 18.4909774877 -0.0170219305
C -11.7721433222 17.2789725728 0.9468138717
H -9.336380094 18.8179662123 -0.8968222649
C -11.0324516445 17.7224485633 -0.1683335323
H -11.3798708445 17.445919963 -1.1651591659
H -9.8422909166 18.7145677552 3.3706962412
C -12.3888981927 13.8997831212 -1.9409125102

C -11.0766404718 14.2035712002 -2.2766278307
C -13.4298523666 14.6623057875 -2.4768925136
H -10.264110814 13.612999982 -1.8538216169
C -10.7756618467 15.258510347 -3.1488224939
C -13.1566812194 15.7066367434 -3.3555749722
H -13.9819228063 16.2826423473 -3.7697953182
C -11.8356640338 15.9915190239 -3.6908370758
H -11.6201559047 16.8052239864 -4.3840899572
H -12.612952093 13.0752948691 -1.2657421017
C -9.352873903 15.598482558 -3.4704666263
H -8.7968299739 14.723984689 -3.8293675062
H -8.8208903404 15.9635311436 -2.5815281066
H -9.2843068711 16.3755650565 -4.2388559317
H -13.2121676613 16.466068871 -0.3904040185
H -15.259160558 17.2422301829 0.5085818318
C -14.5429292834 18.4379386916 2.1656389083
C -13.9425711642 19.6091211526 2.5550184025
C -13.3695376624 20.5269486738 1.603620042
C -13.4386663149 20.1346670803 0.1872447634
C -14.0842170791 18.93243984 -0.1943380105
C -14.1428097181 18.5954790661 -1.5631040552
C -13.559509071 19.411208944 -2.5165262589
C -12.9092030791 20.5981759007 -2.1324742415
C -12.8628046647 20.9563401959 -0.7950317449
H -14.9838512547 17.7749581189 2.9123136092
H -13.9156084862 19.897508297 3.6073210242
O -12.8601496036 21.6222344963 1.9550997184
H -12.3605387309 21.8637410231 -0.4605621522
H -12.4252926694 21.2193117543 -2.8863614299
H -13.6015583582 19.1317593554 -3.5688894017
H -14.6456924153 17.668202274 -1.8493135201
K -17.7516055943 16.0323919561 -0.4820105491
K -15.0428898888 22.8790709587 3.0654538305
N -13.2378034573 22.5701026704 5.3450633539
C -12.3290710366 21.9299804503 4.9923403464
C -11.2136883985 21.1293601204 4.543047787
H -11.1975283008 21.1495660347 3.4469495802
H -10.2704875943 21.515946611 4.9416190164

H -11.339327481 20.0924285924 4.8730819587
N -16.2926419654 24.8217402535 4.7783036656
C -16.7357618808 25.8046698593 5.2178705592
C -17.2859135566 27.0272491255 5.7613544288
H -16.7056073046 27.3568623665 6.6286353739
H -17.2637064277 27.8211091184 5.0084268524
H -18.3239424199 26.8754941477 6.0728891449
N -14.8354269979 24.0194126388 0.4667965033
C -14.5878058885 24.0588428852 -0.6698957709
C -14.2818367894 24.0925473706 -2.0831840596
H -13.4708558733 24.7994866848 -2.284449409
H -13.9676181663 23.0960661059 -2.4170746865
H -15.1598522273 24.3986428139 -2.6611343919
N -16.9933580292 21.388592641 1.6300105249
C -17.4699862334 20.3714442835 1.9430375387
C -18.0566081238 19.1161728741 2.3456328627
H -18.9958456208 18.9334480074 1.8137942222
H -17.3743503551 18.2859796613 2.1267584358
H -18.2452990997 19.1291609792 3.424080244
N -16.3180809148 20.9888724535 5.0273768568
C -16.1438217305 21.5468332248 6.0364842738
C -15.9116237052 22.2510662315 7.2778018464
H -14.983204273 22.8252271357 7.1902177549
H -16.738094401 22.9369806951 7.4875022203
H -15.8195758222 21.5464441772 8.1100707628
N -19.190149381 14.2125275419 -2.3366844794
C -18.5034432117 13.8303710389 -3.1990601405
C -17.6611810439 13.3271716158 -4.2602900496
H -17.308033887 14.1568029919 -4.8785904584
H -16.7806991901 12.8423480712 -3.8232647454
H -18.2132884588 12.6166762691 -4.884105127
N -20.3077069786 16.3784681613 0.82017482
C -20.8744969482 15.3970916199 0.5457807397
C -21.5591694551 14.1755631965 0.1853667477
H -21.1566287599 13.8122425762 -0.7669874804
H -21.3957296725 13.4114692005 0.9512784801
H -22.6339086583 14.3519314808 0.0788698897
N -18.33185457 13.2682869888 0.7800387728

C -18.1568681202 12.3999142742 0.0207629028
C -17.917940323 11.330346868 -0.921533472
H -18.7568058185 11.2495976643 -1.6208294465
H -16.9989053658 11.5530756312 -1.4783217755
H -17.8011567987 10.3755753866 -0.3990285458
N -16.838348006 15.9851340041 2.3183366541
C -16.8932372754 15.0277662305 2.9797204021
C -16.9696318466 13.8388959171 3.7985158216
H -17.8597030754 13.8721718274 4.4348668891
H -17.0308049178 12.9552893296 3.1551852588
H -16.0822696027 13.7531315421 4.435184085
N -17.5910210593 18.803421301 -0.9801101072
C -17.1614417185 19.8527166397 -1.2476704485
C -16.6174794283 21.152612916 -1.5649015758
H -15.5799907545 21.0402084298 -1.9045048641
H -17.2054200995 21.6329055795 -2.354172319
H -16.6306756666 21.777728068 -0.6640853991

TS_A-B_para_2Kcation_MicroSolvation_acetonitrile_conf12
C -13.4604685074 16.6565096654 1.5360330328
C -15.2336333973 17.9263308937 1.4469489674
C -11.1594927311 18.6268008822 3.7656351654
C -10.657476198 19.5081315495 2.8355038262
C -10.9808400963 19.4265222832 1.4837774564
C -11.8383998287 18.4763275638 1.0054748846
C -12.4238530676 17.5669818418 1.931402965
C -12.0836975688 17.656242002 3.3242454598
C -9.8573542663 21.2969795401 1.806936435
H -10.8650776113 18.6584623989 4.8106443403
H -12.1324182734 18.4766460209 -0.0396559981
H -8.8497934785 21.6343776074 1.5482704774
O -9.8003145986 20.5530449282 3.0320899769
O -10.34209674 20.4285357621 0.7985471921
H -10.554322611 22.1392743287 1.9429006063
N -12.6779382705 16.7692594422 4.2337993545
S -11.7043000364 15.6465936397 4.8151426722
O -10.4777948657 16.1429856556 5.4750003981
O -12.5524958311 14.7513667446 5.6444005611

C -12.6161650327 15.2028669869 -1.8716818341
C -13.7299812503 14.3995096087 -2.0972849425
C -12.5108429001 15.9558945192 -0.7060296202
H -13.8098997291 13.8147594029 -3.0117009359
H -11.6005569673 16.5206348819 -0.5227510639
C -14.7331190021 14.3339753156 -1.1288224547
C -13.5274123006 15.9309226218 0.2632443983
H -15.5971923852 13.6893422752 -1.2810607449
C -14.6288043163 15.0831092965 0.0341176181
H -15.4029326322 15.0158796434 0.7996346073
H -11.8098756993 15.2345287193 -2.6027684243
C -9.9383812623 14.9097696273 2.8238581218
C -9.6200864519 14.2810817212 1.6239143343
C -11.187998794 14.693019488 3.3982757347
H -8.6435347894 14.4527075463 1.171120612
C -10.5335024735 13.439688828 0.9799767749
C -12.1206142191 13.8679259726 2.7667297606
H -13.1156383798 13.7428685699 3.1998442684
C -11.7862915873 13.2412302601 1.573516041
H -12.5212824665 12.6129342975 1.0695257726
H -9.2303852622 15.5698443698 3.3210794909
C -10.1824488629 12.7686021063 -0.3121761407
H -9.6249061341 11.8376621774 -0.142470664
H -9.5493800899 13.4068249253 -0.9395107545
H -11.0776355465 12.5109171847 -0.8886906928
H -13.8873292866 16.1003492328 2.3695032907
H -15.8915627121 17.1070246793 1.1616808467
C -15.2707586594 18.3704278801 2.7976150382
C -14.6035356867 19.4981704472 3.1908593384
C -13.9539121323 20.3648314143 2.2365855144
C -14.2319445354 20.0911539938 0.8157293115
C -14.8723205205 18.8856877802 0.4312591572
C -15.0714188441 18.6345612641 -0.9397664102
C -14.6790428122 19.5615938232 -1.8916132592
C -14.0645563444 20.7626226822 -1.5034675467
C -13.8356332755 21.0155474582 -0.1613119815
H -15.7441786721 17.7251087775 3.5374243892
H -14.4989854641 19.7568449217 4.2443740198

O -13.2154040715 21.3156739207 2.5825606279
H -13.3323087705 21.9221738262 0.1717228517
H -13.7569303894 21.4835246541 -2.2586717807
H -14.8474708805 19.355944269 -2.9472189221
H -15.5377122032 17.6967450479 -1.2437100747
K -15.0578105718 15.7430791517 5.5273683954
K -13.9753081951 23.1475317109 4.3407571322
N -16.469697695 15.1483719448 3.0642252364
C -17.2361293599 14.3537856616 3.4416747501
C -18.1706194116 13.366171332 3.9284010641
H -18.6462603652 12.8383330242 3.0962038468
H -17.6293429295 12.6508154274 4.5564396653
H -18.9351030776 13.8513419552 4.541836273
N -17.7721277742 15.3485137403 6.558098383
C -17.6597958525 15.8112167615 7.6224681824
C -17.4873771632 16.3934264243 8.9343684256
H -17.9144361341 17.4005810081 8.9626495465
H -17.9776606649 15.7798817164 9.6963339736
H -16.4158713966 16.4533185846 9.1570989836
N -15.1982980902 12.807658783 5.3838379036
C -14.1594604286 12.28176697 5.4429171355
C -12.8630522154 11.6455471805 5.5090713602
H -12.6491109048 11.1137540538 4.5763029694
H -12.1045960057 12.4228616871 5.6519195442
H -12.8234629843 10.9310635717 6.3372319225
N -14.1521863878 15.809361525 8.3536401708
C -13.036082196 15.4744620075 8.4119581273
C -11.647387943 15.0805954293 8.4903557389
H -11.2201338744 15.3731338847 9.4549588395
H -11.5627020418 13.9950271911 8.3800342494
H -11.0821131155 15.5386719851 7.6684244571
N -16.0063413374 18.3742914599 6.2892156841
C -16.3569275564 19.4701180503 6.0989018961
C -16.7702578815 20.8312984758 5.847386118
H -16.6675708505 21.0652543335 4.7797547902
H -17.8160411607 20.9801723107 6.1342508875
H -16.1519321752 21.5365542732 6.4129883001
N -15.9585650262 24.0655310838 6.2150180652

C -16.209335757 25.2021449133 6.160438496
C -16.5053599242 26.6144055804 6.079983886
H -17.5077771486 26.7750425856 5.671684592
H -16.4531771703 27.0758076686 7.0708696294
H -15.7724859833 27.0955558817 5.4240660115
N -13.6040717448 26.0389309552 4.3557156571
C -12.5128408465 26.4030854817 4.1680510231
C -11.1544789057 26.8370282372 3.934350303
H -11.0745508231 27.3515072014 2.9718756947
H -10.8279318293 27.5206054049 4.724050891
H -10.4980314278 25.9607026777 3.9286486928
N -13.3501786148 21.1322055191 6.265206663
C -13.1193746164 20.0326156594 6.5807098301
C -12.8335663511 18.667027046 6.9466512625
H -13.5652547561 18.3042764642 7.6757658066
H -12.8769736379 18.0226492045 6.0491542231
H -11.8272136752 18.5813068344 7.3741723261
N -11.0759620721 23.5151220435 4.579743482
C -10.504291308 22.7124386925 5.2029123522
C -9.7880130094 21.7332601383 5.988815523
H -9.0910555424 22.2268159105 6.673305412
H -10.4988734536 21.1449639841 6.5781298372
H -9.2333463089 21.0619893577 5.3266501931
N -16.2771440479 22.5724300616 2.694716071
C -16.7483919995 22.0301336976 1.7769279554
C -17.3348827626 21.3512239926 0.642617812
H -18.3216830495 21.7657986417 0.4136174101
H -16.687177714 21.4554528765 -0.2353417202
H -17.4369659803 20.2823794211 0.8591432201

TS_A-B_para_2Kcation_MicroSolvation_acetonitrile_conf13
C -12.7028234018 16.9985630964 1.1951134711
C -14.9228640292 17.1531070196 1.4122321981
C -10.884573475 20.2251636688 1.8482420887
C -10.8158061616 20.699828662 0.5649076636
C -11.4167158782 20.0422967733 -0.5132723559
C -12.0785818146 18.8601993883 -0.3524250866
C -12.1563933522 18.300276898 0.9550597645

C -11.5939211172 19.0155570402 2.0737363184
C -10.3407195592 21.8154209501 -1.3004241871
H -10.4172090325 20.7399316477 2.6848690119
H -12.5091950138 18.35276636 -1.2087075422
H -9.3437643054 21.5668626053 -1.6944289949
O -10.2718423043 21.8767195357 0.1303599997
O -11.2711070463 20.8049047481 -1.6474236923
H -10.6869345723 22.7801351461 -1.6849039877
N -11.7706999642 18.5613297783 3.3624001907
S -10.516220081 18.12028695 4.2380446444
O -9.1971843859 18.5598290387 3.746374318
O -10.8417750248 18.4418154058 5.6525312897
C -13.0392216875 14.7458753292 -1.8584715635
C -12.4171403904 13.593068391 -1.3787439784
C -13.1587840549 15.8684502023 -1.0492667705
H -12.3224196751 12.7178262408 -2.017931922
H -13.692022261 16.740338538 -1.4263166727
C -11.9372897676 13.5653553492 -0.0721507994
C -12.6564665533 15.866196406 0.2625585064
H -11.4593138913 12.6682196858 0.3185136028
C -12.0705120472 14.6833065151 0.7433796658
H -11.6951880539 14.6621080436 1.7666098312
H -13.4422409471 14.7660119975 -2.8691009862
C -9.4362856205 15.6342288884 3.6434650747
C -9.4958700003 14.242627067 3.5687548171
C -10.5208904101 16.3346122559 4.1576063039
H -8.6478518724 13.6914137408 3.1628351993
C -10.6232334441 13.5386149219 3.9992910038
C -11.6660561632 15.6547236022 4.5806961595
H -12.5229385813 16.22292224 4.9456065703
C -11.7098652163 14.2705063042 4.5048809655
H -12.6076407211 13.7412299999 4.8285160943
H -8.5585141807 16.1817843992 3.3078972039
C -10.684722128 12.0441753961 3.9153036029
H -11.6213163385 11.7031996584 3.456555123
H -10.6384018938 11.5827234931 4.9103587438
H -9.8548589506 11.6360378993 3.3291348024
H -12.5768426158 16.6953750674 2.2353143938

H -15.107629178 16.219855214 0.8843376012
C -15.1887242021 18.3737868563 0.7486681401
C -15.1551177536 19.5702434805 1.4205161555
C -14.9343076472 19.6289326326 2.8429916257
C -15.0443185397 18.3539684184 3.5656787182
C -15.0340971473 17.1304897966 2.8501440456
C -15.1407520445 15.9231210672 3.5633429038
C -15.3056530571 15.9261113968 4.9386253265
C -15.3426457269 17.1382262424 5.6431914039
C -15.1958089598 18.3353902637 4.9601467386
H -15.3187132623 18.3677478998 -0.3348072935
H -15.2927894834 20.5143506396 0.8970640589
O -14.699370914 20.7064247999 3.4498708474
H -15.2412915867 19.283842262 5.4972559001
H -15.4855748701 17.1438941838 6.7216594288
H -15.4174706919 14.9834208284 5.4732581561
H -15.1082648481 14.9817388718 3.0127464884
K -12.6669854132 20.4790289 5.3599856254
K -15.9984148798 22.8213004989 4.4574594074
N -17.9612212797 21.0956710618 3.2068849976
C -18.1345180009 19.9492551147 3.0872247575
C -18.3479522967 18.5268896145 2.9303804346
H -17.716238237 18.1440830804 2.1199713964
H -18.0788476663 17.9980797538 3.8509897287
H -19.3969997476 18.3197035434 2.6961285017
N -17.9537294066 25.0816046118 3.8380679934
C -18.4017450304 24.6164449827 2.8669818492
C -18.9410302012 24.0246616426 1.6644966495
H -18.855269541 22.9347547547 1.7340744851
H -19.9932499162 24.297043452 1.5378705707
H -18.3704905716 24.3639498229 0.7950503737
N -17.8690679678 22.9791546895 6.6829969767
C -18.0840509296 24.1039066026 6.9032353257
C -18.3483799953 25.502219734 7.1588930218
H -18.4717828543 26.020769301 6.2023233032
H -19.2586879455 25.6220035923 7.7543645178
H -17.5066526776 25.9529188328 7.6932587009
N -15.1345638467 25.4133145919 6.0499754194

C -15.1384910803 25.9547063806 5.0165582505
C -15.1437467628 26.5973040069 3.720397218
H -15.1940992558 25.8391136197 2.9290235689
H -16.0208115284 27.2439487159 3.6238691716
H -14.2381986662 27.19756643 3.585608326
N -15.6561852995 23.7129781043 1.6072665984
C -14.6967472736 23.1738639122 1.2209499137
C -13.5024095283 22.5081316816 0.7541309769
H -12.6839791637 23.2259277371 0.628049942
H -13.2056147346 21.7499214348 1.4894455684
H -13.6785152043 22.0128445611 -0.2073834473
N -10.0628450361 21.8377391245 4.870283805
C -9.3179176216 21.1767251762 5.4786134922
C -8.4074839364 20.3633768222 6.2477430571
H -8.9311768502 19.9714107942 7.1249231646
H -8.0794596212 19.5216982551 5.6295413779
H -7.5442606809 20.9534291081 6.5711008292
N -11.459190043 21.6980771666 7.9480063323
C -11.7103851041 22.8040613958 7.6742418698
C -12.0308863468 24.1667875439 7.3097530209
H -11.3659902292 24.5014179794 6.506046143
H -13.0669766125 24.2348749543 6.9554982294
H -11.9063802902 24.8352902449 8.1675682931
N -13.0772464058 23.4953616142 3.9549151575
C -12.0457023582 23.7582654232 3.4776082084
C -10.7801564787 24.1029871862 2.8676266102
H -9.99055631 24.099904627 3.6240715721
H -10.5085777885 23.3649379498 2.1034465186
H -10.8373169385 25.0917847096 2.4003096164
N -14.6745515636 21.9505360071 6.7730096127
C -14.9181236949 21.6262193726 7.8645846863
C -15.2104381886 21.2351021951 9.2215115193
H -14.2888648706 21.2425119466 9.8125419752
H -15.9396996548 21.9194610194 9.6661950664
H -15.6110325097 20.2177519633 9.2329421315
N -13.5711669981 18.8781856489 7.8607173458
C -12.8692883431 17.9501368528 7.9369978976
C -12.0106882 16.7903620048 8.0172331144

H -11.1559188226 16.9394238248 7.348100107
H -11.6579656684 16.6400412994 9.0422550955
H -12.554507033 15.8941028286 7.6971746978

TS_A-B_para_2Kcation_MicroSolvation_acetonitrile_conf14
C 12.5213988229 -16.4392906075 -0.9720097292
C 14.3176697324 -17.7212033853 -1.0324998108
C 12.4134016683 -14.6692747785 2.3283343017
C 12.8703533578 -13.4329805825 1.9396434978
C 13.2743576426 -13.1610598911 0.6318030021
C 13.2174596485 -14.1091726325 -0.3506659605
C 12.7465479434 -15.406331595 -0.00305746
C 12.3814807509 -15.6965859899 1.3598198308
C 13.4227221047 -11.2748969101 1.8053487336
H 12.0785681063 -14.8767555381 3.3417524539
H 13.475414614 -13.8543833596 -1.3727180526
H 12.5968208753 -10.5543331078 1.7116719449
O 13.0273309301 -12.3167769192 2.7066766329
O 13.7026443364 -11.8607421438 0.5458873464
H 14.3236013176 -10.7868036517 2.1943573408
N 12.062499736 -16.9921055827 1.7370809151
S 10.5775484222 -17.3256949474 2.201557486
O 9.7946277755 -16.181155332 2.7087787421
O 10.6686029344 -18.5200354708 3.0797363489
C 12.9179565364 -15.3438164443 -4.596235639
C 11.7597377698 -15.8904915333 -5.148156179
C 13.1980380462 -15.5116547786 -3.2459931347
H 11.5415395592 -15.7539233473 -6.2052782748
H 14.1288330018 -15.1137395803 -2.8426122959
C 10.8963332319 -16.6271235531 -4.3414347974
C 12.3231804603 -16.2278792978 -2.4110689054
H 9.9956320537 -17.0695362047 -4.7642335269
C 11.1834199269 -16.8056444923 -2.9928921558
H 10.5103436112 -17.3853436412 -2.3614178295
H 13.6114040152 -14.788868308 -5.2250992367
C 8.7291417547 -17.0903171474 0.1545221428
C 8.0847861671 -17.5341433459 -0.9988343702
C 9.6995152729 -17.8917800718 0.747226587

H 7.322337414 -16.9079647298 -1.4620598821
C 8.3991607557 -18.7666903275 -1.5776099242
C 10.0398436865 -19.1214293892 0.179665599
H 10.8167338204 -19.7263960197 0.645834538
C 9.3926957218 -19.5503383729 -0.9706767627
H 9.659071598 -20.5110836888 -1.4133019909
H 8.4784133985 -16.1347033519 0.6093325969
C 7.7044941095 -19.2414864376 -2.8172789863
H 8.4135277032 -19.6537114524 -3.5460507186
H 6.9847803996 -20.0402934939 -2.594776979
H 7.150088263 -18.4333835598 -3.3061784049
H 11.9151631587 -17.2472670162 -0.5678262782
H 14.6047276953 -17.2896384105 -1.9893896365
C 13.6859655417 -18.9943886466 -1.0292367681
C 13.5965623124 -19.7654544996 0.1031439248
C 14.1767092725 -19.3332975027 1.3385025145
C 15.0365084423 -18.1417924149 1.2888671947
C 15.1010112238 -17.3522483464 0.1144028804
C 15.9077250395 -16.199370026 0.1211075416
C 16.6534085437 -15.8557892657 1.2371667561
C 16.6149624266 -16.6599428413 2.3853549192
C 15.8063085502 -17.7845165851 2.404413239
H 13.1885403364 -19.322556557 -1.9441773844
H 13.0325857389 -20.6975751451 0.112792638
O 13.9822683851 -19.9279107638 2.4347097766
H 15.7677190762 -18.4316661159 3.282265519
H 17.2024133076 -16.39092159 3.2610842516
H 17.2745716514 -14.9619261861 1.22026539
H 15.9445901651 -15.5762734884 -0.7732991984
K 13.093395895 -18.1033803722 4.3752227799
K 15.4310045725 -21.8525446605 3.4912692434
N 17.1155472969 -21.2449936071 1.2223596826
C 17.4658458088 -20.3093991448 0.621163228
C 17.9091307146 -19.1575713221 -0.1304210717
H 17.2364328754 -18.9785365226 -0.9757669279
H 17.8969331805 -18.2651893087 0.507411291
H 18.9242577307 -19.3142055382 -0.5091419695
N 17.2491598503 -24.1794675502 3.1845381294

C 18.2511352461 -23.5828233699 3.1546378581
C 19.4759289314 -22.8164683568 3.1236431913
H 19.8285105544 -22.7087163651 2.0932851269
H 19.2822287749 -21.8204766103 3.5388016868
H 20.2531587455 -23.3092629695 3.7156689673
N 17.6437210413 -20.3162634209 4.6346351717
C 17.3126502432 -19.4371218893 5.326805908
C 16.8824426181 -18.3491511003 6.1746791159
H 16.4210312283 -17.5468035301 5.5839547113
H 16.121912804 -18.6941091488 6.8836969696
H 17.7308558839 -17.933975229 6.7280023482
N 14.0416799323 -23.8313910558 5.1770297779
C 14.1975275935 -24.8261668481 4.5885768668
C 14.4058460348 -26.050970708 3.8502282656
H 15.4149030109 -26.0422497412 3.4238834094
H 14.3006896779 -26.9199167906 4.5069866184
H 13.6778062857 -26.1262343045 3.0367530621
N 14.0793149474 -23.5629732146 1.5814674166
C 14.9420533477 -23.8884403269 0.8670145553
C 16.0253158652 -24.2892968074 -0.0026812514
H 16.7858300914 -23.5009994316 -0.0157771906
H 16.4857045289 -25.210328176 0.3690717944
H 15.6631287269 -24.4572937614 -1.0214765598
N 11.2598021027 -16.2724029342 5.7511094356
C 10.1846400391 -16.6843726613 5.559499663
C 8.8556286216 -17.2129906992 5.3604847447
H 8.2725988416 -16.5187005559 4.7500117248
H 8.9357126126 -18.1589478792 4.815634657
H 8.3590537677 -17.3784461108 6.3227059537
N 13.6577414599 -18.0269204824 7.5924893731
C 12.5731610318 -18.0228433456 8.0212471741
C 11.2277420282 -18.0410514953 8.5523676734
H 10.7964486899 -17.0357820604 8.5200070289
H 10.613336088 -18.7137785229 7.943019154
H 11.2278453568 -18.392511386 9.5892988947
N 10.9868929602 -20.1493605268 5.8787798169
C 11.1121554899 -20.7266044977 4.8718027211
C 11.3047547569 -21.4301091735 3.623389723

H 10.4076061686 -21.3321738508 3.0044295493
H 12.1477667487 -20.9901580294 3.0680117786
H 11.5111756189 -22.4897967779 3.8079808871
N 14.4121562544 -20.4296876089 5.6653554228
C 13.9435827992 -20.959121944 6.5914858944
C 13.3424513203 -21.6069473638 7.7312505475
H 13.8490421358 -21.3081916731 8.65446556
H 12.2867916629 -21.3192988079 7.7884933992
H 13.4105593262 -22.6928991932 7.6105699165
N 14.8174998758 -15.8656733856 5.0679418507
C 14.2229830919 -14.9217518306 5.4058217401
C 13.4667661123 -13.7625274673 5.8200961061
H 13.3925777172 -13.0401401934 4.9997241663
H 12.4583388854 -14.0850259698 6.1014665066
H 13.9430145328 -13.2799109942 6.6791994186

TS_A-B_para_2Kcation_MicroSolvation_acetonitrile_conf15
C 13.1023 -17.676886 -1.256446
C 15.271999 -17.928868 -1.437365
C 12.042112 -20.709952 0.679895
C 12.360795 -20.290113 1.951131
C 12.824146 -19.001843 2.213452
C 13.018791 -18.08698 1.215673
C 12.761421 -18.490776 -0.126161
C 12.27311 -19.817526 -0.385084
C 12.999077 -20.203418 4.067491
H 11.646598 -21.699555 0.474837
H 13.447507 -17.113115 1.434071
H 12.448061 -20.209639 5.012754
O 12.299111 -21.000904 3.109852
O 13.058258 -18.871144 3.560442
H 14.018472 -20.599576 4.188768
N 12.047402 -20.225528 -1.698246
S 10.531011 -20.295227 -2.167414
O 9.604083 -20.942946 -1.21856
O 10.547929 -20.843562 -3.548042
C 12.261347 -14.039488 -0.543838
C 12.65044 -13.441856 -1.739078

C 12.408135 -15.410779 -0.355594
H 12.53403 -12.369192 -1.881131
H 12.040377 -15.862643 0.56184
C 13.171596 -14.236181 -2.762134
C 12.95678 -16.22416 -1.360554
H 13.465083 -13.785132 -3.709199
C 13.317253 -15.602115 -2.571592
H 13.733062 -16.222077 -3.367415
H 11.825325 -13.434614 0.249567
C 9.556807 -17.919936 -1.172972
C 9.27798 -16.559256 -1.25212
C 9.984222 -18.601725 -2.311467
H 8.94789 -16.025607 -0.360466
C 9.416257 -15.85678 -2.452692
C 10.13397 -17.921007 -3.520161
H 10.462724 -18.473799 -4.401132
C 9.840276 -16.56424 -3.585103
H 9.927282 -16.03404 -4.535897
H 9.437241 -18.461501 -0.23581
C 9.106855 -14.393829 -2.536037
H 9.877559 -13.850684 -3.096663
H 8.151519 -14.212678 -3.046387
H 9.037326 -13.939129 -1.542044
H 12.946198 -18.192836 -2.204039
H 15.336937 -17.313356 -2.333881
C 15.366794 -19.340607 -1.588257
C 15.503102 -20.170724 -0.509624
C 15.687554 -19.647812 0.823679
C 15.916546 -18.192654 0.92732
C 15.728397 -17.351044 -0.198178
C 15.923736 -15.963741 -0.047094
C 16.318169 -15.431348 1.169298
C 16.518395 -16.271383 2.276707
C 16.306425 -17.63434 2.152572
H 15.232087 -19.754359 -2.587092
H 15.469152 -21.25251 -0.630916
O 15.673982 -20.376886 1.842651
H 16.43926 -18.306837 2.997219

H 16.845644 -15.851063 3.227525
H 16.476817 -14.358478 1.264221
H 15.76085 -15.310367 -0.904814
K 13.206037 -21.386876 -3.90882
K 17.273092 -21.19743 3.91296
N 15.022913 -22.993715 4.023372
C 14.660224 -23.141688 2.924881
C 14.219287 -23.301926 1.558482
H 13.212591 -23.730654 1.526513
H 14.208118 -22.319698 1.072479
H 14.902333 -23.953361 1.004087
N 18.419632 -21.869544 6.442098
C 18.594017 -22.134595 7.561759
C 18.809022 -22.46373 8.95408
H 18.332085 -23.417803 9.198757
H 19.879094 -22.544814 9.168455
H 18.38394 -21.688749 9.599252
N 18.643876 -23.51284 2.908849
C 19.384458 -24.38474 2.694949
C 20.306869 -25.468039 2.431284
H 19.939129 -26.400136 2.871291
H 20.426652 -25.616122 1.35366
H 21.288629 -25.245785 2.860674
N 19.02369 -19.735158 2.175932
C 19.090979 -19.007242 1.269268
C 19.161039 -18.098593 0.145794
H 20.200166 -17.940948 -0.159985
H 18.598138 -18.503853 -0.702048
H 18.718042 -17.133302 0.418193
N 15.904713 -19.121043 5.411556
C 15.279467 -18.137942 5.424707
C 14.510144 -16.913699 5.413982
H 13.475415 -17.110986 5.709166
H 14.944428 -16.17643 6.09639
H 14.498296 -16.503305 4.397877
N 11.89525 -20.424605 -6.634057
C 10.88049 -20.983189 -6.495253
C 9.620655 -21.666981 -6.313476

H 9.25939 -21.458688 -5.300329
H 8.887157 -21.323764 -7.049555
H 9.759781 -22.746739 -6.42912
N 15.046773 -22.272043 -5.925748
C 15.060039 -21.470701 -6.772408
C 15.056158 -20.468546 -7.814141
H 14.038416 -20.077248 -7.922368
H 15.72914 -19.646643 -7.550431
H 15.380196 -20.899813 -8.766205
N 14.173093 -18.763255 -4.720663
C 13.46983 -18.036545 -5.300929
C 12.599167 -17.13253 -6.01694
H 11.876019 -17.711658 -6.601765
H 12.058433 -16.496108 -5.305988
H 13.17615 -16.495123 -6.694647
N 14.75431 -23.280255 -2.291134
C 14.826223 -24.123321 -3.094272
C 14.89092 -25.151922 -4.108004
H 13.906512 -25.249478 -4.578383
H 15.61565 -24.87063 -4.877477
H 15.179364 -26.112125 -3.669584
N 11.723005 -23.870196 -4.207196
C 10.744928 -23.855408 -3.57102
C 9.531353 -23.834213 -2.787077
H 9.491514 -22.919303 -2.178761
H 8.656256 -23.848339 -3.445539
H 9.484279 -24.709771 -2.131907

TS_A-B_para_2Kcation_MicroSolvation_acetonitrile_conf16
C 12.256691 -17.449407 -1.187822
C 14.487521 -17.797022 -0.902338
C 12.260185 -15.841752 -4.574433
C 11.522952 -16.768989 -5.271733
C 11.071513 -17.954772 -4.688928
C 11.3245 -18.258379 -3.377591
C 12.059921 -17.318336 -2.603701
C 12.55015 -16.110193 -3.217002
C 10.317166 -17.874995 -6.77952

H 12.62312 -14.926973 -5.030874
H 10.912574 -19.157671 -2.932384
H 9.281709 -17.52052 -6.894188
O 11.179391 -16.748233 -6.590792
O 10.42106 -18.707016 -5.633144
H 10.640312 -18.430172 -7.666408
N 13.365629 -15.274114 -2.472667
S 12.912888 -13.776108 -2.175845
O 12.362817 -13.025171 -3.328862
O 14.048798 -13.14604 -1.463333
C 11.069876 -20.690157 0.388321
C 10.676152 -20.221194 1.6395
C 11.588053 -19.813503 -0.558313
H 10.264564 -20.910726 2.375216
H 11.939167 -20.207017 -1.509301
C 10.826165 -18.869018 1.950534
C 11.705886 -18.436723 -0.281832
H 10.543526 -18.4969 2.934538
C 11.342996 -17.993306 1.007338
H 11.471717 -16.936997 1.249838
H 10.986425 -21.748782 0.151208
C 10.337585 -14.392635 -1.439102
C 9.361487 -14.721346 -0.506834
C 11.584534 -13.94524 -0.998025
H 8.38936 -15.074746 -0.849764
C 9.603631 -14.610076 0.868299
C 11.843377 -13.810737 0.36381
H 12.817497 -13.449841 0.687051
C 10.851597 -14.133916 1.284381
H 11.051665 -14.022492 2.350725
H 10.138676 -14.479334 -2.506341
C 8.564745 -15.025157 1.863892
H 8.756907 -14.600251 2.854751
H 7.55819 -14.720952 1.55556
H 8.543213 -16.118316 1.975897
H 12.452617 -16.489677 -0.711613
H 14.618085 -17.009145 -1.638727
C 14.655999 -19.144988 -1.285259

C 14.735953 -20.15675 -0.362996
C 14.739689 -19.891152 1.053172
C 14.648945 -18.479252 1.464103
C 14.566113 -17.451497 0.489619
C 14.464144 -16.11135 0.919699
C 14.448399 -15.801457 2.268623
C 14.545761 -16.819736 3.230778
C 14.650252 -18.139783 2.826193
H 14.66335 -19.383331 -2.351193
H 14.822253 -21.198353 -0.667302
O 14.832648 -20.811175 1.902
H 14.750153 -18.944123 3.554231
H 14.544684 -16.570423 4.290804
H 14.365543 -14.761595 2.583445
H 14.366558 -15.321961 0.169376
K 16.159404 -14.69396 -2.284275
K 17.285796 -21.417459 2.868282
N 17.82038 -18.848367 4.130524
C 17.839143 -17.924013 3.420139
C 17.864181 -16.783674 2.531688
H 17.071253 -16.875095 1.780128
H 17.693594 -15.853881 3.083246
H 18.830015 -16.716621 2.020319
N 19.114736 -22.147555 5.067834
C 18.696315 -21.48536 5.931545
C 18.170575 -20.652855 6.988484
H 17.869831 -19.690787 6.56106
H 17.289003 -21.125169 7.430878
H 18.925657 -20.489018 7.763422
N 15.529994 -21.402747 5.212689
C 14.420665 -21.274925 4.876452
C 13.056694 -21.108439 4.427997
H 12.61628 -20.203202 4.859663
H 13.07171 -21.011149 3.335805
H 12.445161 -21.969712 4.715082
N 18.353323 -23.958882 2.196575
C 19.161005 -24.45783 2.871928
C 20.162042 -25.054079 3.726655

H 20.371753 -24.366543 4.553017
H 19.804557 -26.004442 4.134647
H 21.085859 -25.238333 3.169848
N 18.001895 -19.61363 0.724801
C 18.033029 -18.810036 -0.118683
C 18.077942 -17.787416 -1.139857
H 17.955691 -16.796296 -0.681641
H 19.038231 -17.799672 -1.665173
H 17.278682 -17.937565 -1.875711
N 15.929099 -17.047749 -3.89851
C 15.285998 -17.633683 -4.673314
C 14.491485 -18.381974 -5.621955
H 15.087812 -19.17995 -6.075707
H 13.627192 -18.828558 -5.115386
H 14.12059 -17.723888 -6.414658
N 18.953953 -14.831071 -3.131402
C 18.950681 -14.044563 -3.992865
C 18.931968 -13.057237 -5.048557
H 19.948923 -12.824563 -5.379016
H 18.354694 -13.429151 -5.900092
H 18.452274 -12.145394 -4.677215
N 15.536547 -13.63725 -4.915225
C 14.662064 -13.114961 -5.482714
C 13.566579 -12.468684 -6.171461
H 13.27191 -13.045299 -7.054281
H 12.715967 -12.399347 -5.483751
H 13.853304 -11.462911 -6.49565
N 17.024517 -11.805946 -2.47519
C 16.009602 -11.253801 -2.637025
C 14.75738 -10.56693 -2.856658
H 14.889167 -9.751422 -3.575645
H 14.002535 -11.276124 -3.217524
H 14.387033 -10.153991 -1.913616
N 17.812507 -14.525205 0.119914
C 18.608189 -13.714019 -0.14358
C 19.58204 -12.705059 -0.491193
H 19.929031 -12.176465 0.401861
H 20.439218 -13.169595 -0.988512

H 19.117085 -11.989775 -1.179171

TS_A-B_para_2Kcation_MicroSolvation_acetonitrile_conf17
C 11.74013082 -15.423403555 -1.3162950537
C 13.641434734 -14.8375425249 -0.3812649076
C 9.4882777819 -16.993975201 1.2568461524
C 9.0725620721 -15.8616264417 1.9137540093
C 9.5348819343 -14.5878530118 1.577195848
C 10.4097316046 -14.3961031675 0.5408013565
C 10.8363656063 -15.5333927677 -0.1953773488
C 10.4225495804 -16.8475966894 0.2036104792
C 8.0777689595 -14.3797028734 3.240499633
H 9.144320831 -17.9868942641 1.5323161965
H 10.7997274319 -13.4055974138 0.3213657799
H 7.0602189181 -14.0965065533 2.9307364094
O 8.2675492702 -15.7766574788 3.0126389694
O 9.0329822134 -13.6700247175 2.4607316016
H 8.2375660168 -14.1616664998 4.3015278122
N 10.9559358929 -17.9730707041 -0.4154106085
S 9.9580791516 -18.8470910102 -1.2978165862
O 8.5861926411 -18.9778923708 -0.7753912117
O 10.6791985532 -20.1100343138 -1.6073389375
C 10.632972062 -12.580435587 -3.5730083426
C 11.4133907364 -12.8361079481 -4.7013578408
C 10.7233100255 -13.3965668608 -2.4536311991
H 11.3379120002 -12.1921636625 -5.5757615438
H 10.0676812981 -13.2084391312 -1.6082292181
C 12.2692872726 -13.934860874 -4.7105755572
C 11.6052796649 -14.4948497387 -2.4299234393
H 12.8671963589 -14.1594022553 -5.5927037965
C 12.3586477313 -14.7532604093 -3.5935947953
H 13.0194809499 -15.6199681701 -3.6079812273
H 9.9382008882 -11.7428072405 -3.5708370273
C 8.845766217 -16.9581421717 -2.9547252632
C 8.799930945 -16.1795339073 -4.1054803514
C 9.8071403674 -17.9628945679 -2.8413423643
H 8.0497483139 -15.3933604549 -4.1928565347
C 9.7002753542 -16.3840785223 -5.156093861

C 10.7270163077 -18.1730236512 -3.8688593163
H 11.4790530095 -18.9554059205 -3.7640787599
C 10.6623125828 -17.3923511963 -5.0167479559
H 11.3746914614 -17.5614995173 -5.8254728666
H 8.1326118558 -16.8059588866 -2.1464234197
C 9.6194029679 -15.5632996121 -6.4060348001
H 8.9263912862 -16.0095413172 -7.1321250952
H 9.2585032584 -14.549374056 -6.1998029968
H 10.5934067198 -15.4798767132 -6.9016840303
H 12.151522982 -16.39075883 -1.611772712
H 13.2841666363 -13.8429536877 -0.113229753
C 13.6566372083 -15.8470446675 0.6213188479
C 14.3159758485 -17.0327592968 0.4426920004
C 15.033017239 -17.3115253414 -0.772841112
C 15.2786507132 -16.1726570443 -1.6621118588
C 14.6188295623 -14.9339241884 -1.4311720788
C 14.9168527725 -13.8460481864 -2.2739672339
C 15.8447463124 -13.9718537994 -3.2947102486
C 16.4918275697 -15.1960583953 -3.5188400078
C 16.2021475708 -16.2852658104 -2.7142792919
H 13.042360608 -15.7047792678 1.5122968457
H 14.2663687149 -17.8183585578 1.1945457927
O 15.4248747516 -18.4781305223 -1.0585596237
H 16.7138132289 -17.2361471322 -2.8591398636
H 17.2232403456 -15.2888166473 -4.3196839849
H 16.0688182749 -13.1149663452 -3.9278712489
H 14.4016555456 -12.8983725414 -2.1144051399
K 13.0678333132 -19.8926719532 -0.2959455091
K 17.6456445232 -19.7121480244 -0.3162521723
N 17.0500041793 -21.6920306597 -2.5499716965
C 16.4814659629 -20.9073923938 -3.2012022681
C 15.7895427053 -19.9095012221 -3.9821804881
H 15.3726840633 -19.166062224 -3.2903628673
H 16.4936183023 -19.4107961544 -4.6548828914
H 14.9752864945 -20.3681737348 -4.5496808453
N 20.1404188881 -20.9858945915 -0.8980242774
C 20.1544405319 -21.5466750326 -1.9200054887
C 20.166343359 -22.223954656 -3.1966650276

H 19.4038071579 -23.0079535146 -3.2060651845
H 19.9264746875 -21.5102548258 -3.991874537
H 21.1477538969 -22.6664536262 -3.392790286
N 18.7194721519 -18.7452020654 -3.1791958173
C 19.726636333 -18.2988079778 -2.7959310156
C 20.9789116076 -17.7688897556 -2.3051840914
H 21.7426623869 -17.7911303525 -3.0888292001
H 20.8413086503 -16.739223834 -1.9633530655
H 21.3195956038 -18.3675538391 -1.4540531149
N 18.7439053758 -17.0648249583 -0.1592378961
C 18.1425136852 -16.1479182784 0.2354338632
C 17.3941129884 -15.0147898734 0.729553702
H 17.9685301576 -14.479310755 1.4919790542
H 17.1583889657 -14.3284446496 -0.0912435369
H 16.4515963039 -15.361573595 1.1703428325
N 16.699834886 -18.7858072519 2.26128506
C 16.189298759 -19.5807460404 2.9444150545
C 15.5714869906 -20.5855481876 3.7808875677
H 15.0619553289 -21.3129674355 3.1402600301
H 16.3274256022 -21.0991022368 4.383416123
H 14.8331920867 -20.1291042302 4.4462715238
N 13.4855903111 -21.6196753756 -2.6272436195
C 12.4450870656 -22.1270409095 -2.7708169417
C 11.1563469248 -22.7585749723 -2.931825496
H 10.3967205904 -21.9841784694 -3.0769545722
H 11.16135994 -23.4461344157 -3.7830433376
H 10.9123630763 -23.3129947546 -2.0190951858
N 12.1145638416 -22.7036228828 0.5169475733
C 11.9730252213 -22.6864510378 1.6748767797
C 11.8047047254 -22.6689691448 3.1110877523
H 10.787475206 -22.9697559013 3.3790106981
H 12.5167692803 -23.3495642634 3.5890809276
H 11.9626955612 -21.650638569 3.4808157887
N 15.4564248833 -21.5978770635 0.5581183913
C 15.2979982827 -22.5715254506 -0.0654803991
C 15.0926839284 -23.7774147139 -0.8323476413
H 14.0295132776 -24.0392662494 -0.8149782519
H 15.4066383543 -23.6006827294 -1.8657485091

H 15.6772874353 -24.6020584426 -0.4125924
N 12.8631842678 -19.3852693722 2.5532708373
C 12.0816789764 -18.7840851495 3.1769096385
C 11.1176477786 -18.0577569801 3.9709869317
H 10.128897941 -18.5037326869 3.8174162924
H 11.3793626006 -18.1097630488 5.0328486017
H 11.0827773332 -17.0076384762 3.6638109457
N 9.6190183274 -20.4535809982 2.3282908201
C 9.1300505513 -21.0076377903 1.4255109236
C 8.5428107649 -21.7362887544 0.3238047465
H 9.1959122974 -21.6412490165 -0.5499542101
H 8.4163735621 -22.7916850496 0.5894484065
H 7.575020232 -21.3010622093 0.0613876555

TS_A-B_para_2Kcation_MicroSolvation_acetonitrile_conf18
C 12.0743787635 -14.9298098826 -1.1619118613
C 13.8527716012 -14.1508509965 -0.0936499799
C 13.4264954115 -17.8666720197 -3.0672887135
C 13.3461791079 -18.8329834672 -2.0879820918
C 12.8079518822 -18.5734530064 -0.8317666516
C 12.3574368342 -17.3265430881 -0.4929640555
C 12.4298404538 -16.2845168697 -1.4638036852
C 12.9585551291 -16.5736576218 -2.7703742122
C 13.2407676068 -20.734805232 -0.9564039933
H 13.8005839858 -18.07909399 -4.0618648389
H 12.01253785 -17.1229409753 0.514657035
H 12.3184759738 -21.2713931328 -1.234348422
O 13.7843123959 -20.1256041519 -2.1398246328
O 12.9346599349 -19.6918617707 -0.0431694387
H 13.9901152353 -21.4002077263 -0.5254864586
N 12.934435456 -15.5859544086 -3.7621328141
S 11.8374556177 -15.8223720625 -4.8903287773
O 12.0051268224 -17.0604342716 -5.6976886973
O 11.7596138778 -14.5716875653 -5.6846976626
C 8.9846030152 -14.8189239758 1.0511135976
C 8.8040460455 -13.443676734 1.1732844732
C 10.0479655389 -15.3308774926 0.3146750876
H 7.9719351603 -13.0478302408 1.7518744625

H 10.121383251 -16.4060260627 0.1728035929
C 9.689462933 -12.5778227375 0.5303541214
C 10.9690654835 -14.4752897325 -0.3131638218
H 9.5468295506 -11.5009777487 0.597979269
C 10.7500832193 -13.0882485651 -0.2036209256
H 11.4277760523 -12.4083766915 -0.7228843163
H 8.2808230725 -15.5043421119 1.5205506429
C 9.7417236986 -17.2713416005 -3.8127946988
C 8.5995938438 -17.399943572 -3.0301601861
C 10.2748288329 -16.0049074444 -4.0455480926
H 8.1826483054 -18.3904139847 -2.8469978752
C 7.9745872537 -16.2799902405 -2.4685466187
C 9.6783233358 -14.8787197411 -3.4809527015
H 10.1351211664 -13.8985810022 -3.6195961103
C 8.5311313759 -15.0197225106 -2.7102382469
H 8.0768337592 -14.1395672755 -2.2546607205
H 10.2269602787 -18.1433236358 -4.2480924969
C 6.7489955141 -16.4310272349 -1.6208597033
H 5.874504504 -16.7103670959 -2.2227276627
H 6.8748283378 -17.2183239952 -0.8670530844
H 6.5037125842 -15.5011927817 -1.0968246303
H 12.2883179434 -14.2487841235 -1.9875928109
H 13.4134642391 -13.1684794356 0.073633826
C 13.8977781106 -15.0626533109 0.9926777026
C 14.5938990357 -16.2386080576 0.899357737
C 15.3389732121 -16.5783926533 -0.2914762703
C 15.6001981299 -15.4667648871 -1.2208874889
C 14.8858077653 -14.2511152538 -1.0958983068
C 15.2127425285 -13.1813227661 -1.9509205693
C 16.258974532 -13.2927187479 -2.853643442
C 16.9613853651 -14.5024009738 -2.9735948041
C 16.6156397807 -15.5820886468 -2.1806731791
H 13.2663426745 -14.8702213636 1.860788959
H 14.521565634 -16.999074076 1.6765525064
O 15.7749741963 -17.7339470082 -0.5075287626
H 17.1616132748 -16.5214399616 -2.25732613
H 17.777492379 -14.5850952426 -3.6885701774
H 16.5336128821 -12.4414695855 -3.4762044922

H 14.6528214823 -12.2473345299 -1.8729405672
K 14.1954501335 -13.4687946102 -5.2121438533
K 16.8029837768 -19.4628817503 -2.4824945013
N 18.9140856951 -18.4145434823 -0.7380287547
C 18.8257437229 -17.4061094474 -0.1604437921
C 18.7074212677 -16.1539217094 0.5561534584
H 18.8038870507 -15.3088782692 -0.1335716865
H 17.7249027537 -16.0833884013 1.0373650161
H 19.4834298657 -16.0720517374 1.3237310779
N 18.6732113085 -21.4689870165 -3.19751668
C 19.4426194326 -22.3411322986 -3.2352717853
C 20.3986075651 -23.4262213273 -3.2803908307
H 21.3859341664 -23.0794565708 -2.9602485463
H 20.0850254034 -24.2379828971 -2.6168504775
H 20.4821319325 -23.8222514027 -4.297143654
N 16.3317888508 -21.0743252778 -0.1333650648
C 16.3004531485 -20.4016058682 0.8179752587
C 16.2551726609 -19.5767732394 2.0033245032
H 16.64950571 -18.5852213339 1.7633961883
H 15.2183500066 -19.4622049305 2.3363564178
H 16.841896169 -20.0251551949 2.8110063949
N 15.4412216253 -20.0555829239 -5.0010879142
C 14.337295583 -20.1540986092 -5.3632455393
C 12.9519195284 -20.2226447859 -5.7719260731
H 12.8544759015 -20.7265176343 -6.7384857236
H 12.3747493016 -20.7810032952 -5.0266042472
H 12.5364685771 -19.2076355179 -5.8479652797
N 18.3420866886 -17.6569782754 -4.1755305981
C 19.0813570726 -16.9844135572 -4.7745509044
C 19.9855452969 -16.1372972728 -5.5210950607
H 20.7494327809 -15.716799528 -4.8595078009
H 20.4858856308 -16.7111705503 -6.3072642744
H 19.4247336223 -15.3177126735 -5.985329654
N 15.4617203247 -10.8654330627 -5.092220131
C 14.6752873794 -10.0058840074 -5.0525908175
C 13.687036652 -8.9519951541 -5.0289314311
H 13.1162807652 -8.9955864723 -4.096541568
H 14.1642910617 -7.970987818 -5.1143270959

H 12.9947530386 -9.0864306156 -5.867404119
N 13.1356547928 -11.5693159654 -7.4981402279
C 14.1804909439 -11.7401694348 -7.9885530771
C 15.4929789199 -11.9819666724 -8.5448730425
H 16.2611284755 -11.6009537461 -7.8629297346
H 15.6386532231 -13.0612034376 -8.6668513603
H 15.6013541042 -11.4888482395 -9.515766375
N 16.9689880457 -14.6507872055 -6.2945355375
C 16.3965673863 -15.6489408957 -6.0951781586
C 15.6623555749 -16.8566369159 -5.7948436784
H 16.1438767474 -17.7302527617 -6.2446127002
H 15.6458061778 -17.0043141075 -4.7080295357
H 14.6289828307 -16.7811532731 -6.1592776614
N 14.065078614 -14.8855655083 -7.9561316394
C 12.9652286242 -15.16895482 -8.2202641636
C 11.6014677162 -15.5143090769 -8.5460334904
H 11.5176836315 -15.8213086523 -9.5932519221
H 11.276056659 -16.3269419115 -7.888014296
H 10.9543792276 -14.6501988876 -8.3661434146
N 12.1853520802 -11.7117552171 -3.9125807499
C 11.3440042403 -11.7762779468 -4.719618996
C 10.3128807585 -11.8510793308 -5.730128598
H 9.4985661111 -11.1545492742 -5.5074612454
H 10.7541599211 -11.6053520354 -6.702303819
H 9.9246395254 -12.8736120942 -5.7766751673

TS_A-B_para_2Kcation_MicroSolvation_acetonitrile_conf19
C 11.3851569086 -15.5462633371 -1.8212779118
C 13.3633977732 -14.9993207219 -1.0222871917
C 10.0440758165 -12.063686886 -2.1667153515
C 9.9881456527 -11.9767664881 -3.5384588112
C 10.4114165068 -13.0103949384 -4.37633228
C 10.9020195671 -14.1869867536 -3.8794853981
C 10.9689719922 -14.3344863985 -2.466787587
C 10.5588486863 -13.2540007922 -1.6051352143
C 9.5811770951 -11.3825463358 -5.6436681775
H 9.7030476388 -11.2635875978 -1.5165203788
H 11.1781079354 -14.9952549871 -4.5476913492

H 8.5406042689 -11.5171469571 -5.9759519025
O 9.5820864303 -10.9161396764 -4.2915343653
O 10.2681318105 -12.6245223326 -5.6847464538
H 10.100456584 -10.6540566322 -6.2756293448
N 10.7532032254 -13.3858416511 -0.2395302136
S 9.471756528 -13.318687112 0.7217978509
O 8.4569151095 -12.3131398463 0.3574426412
O 9.996441807 -13.2797852371 2.1045042242
C 12.511759197 -18.3299039294 -4.1488622227
C 11.9750508173 -19.4346678949 -3.4917732083
C 12.3305749973 -17.0491774012 -3.6375222794
H 12.1051094604 -20.4337944767 -3.904881585
H 12.7814204779 -16.2040793497 -4.1535740903
C 11.2820606714 -19.2543663786 -2.2943608542
C 11.6157940538 -16.8446070572 -2.4431455367
H 10.8672069365 -20.112318223 -1.7660753274
C 11.12758394 -17.9789737836 -1.7663154337
H 10.593526543 -17.8353302546 -0.8254080145
H 13.0781285181 -18.4625067966 -5.0688690671
C 7.9436297911 -15.1505067482 -0.6437598607
C 7.5067348665 -16.4387102835 -0.9245215663
C 8.7153912099 -14.919639157 0.4968535918
H 6.907620999 -16.6197081053 -1.8168686006
C 7.8202808883 -17.5117432052 -0.0803827709
C 9.0372270438 -15.9696662346 1.3535675157
H 9.6303268753 -15.7680347213 2.2434543641
C 8.5781776332 -17.2521905306 1.0664298184
H 8.8124978544 -18.0725151958 1.7457201626
H 7.6867021546 -14.319101394 -1.2983651315
C 7.3737590227 -18.9017568859 -0.4163268147
H 6.2938903457 -18.9493988197 -0.6013369542
H 7.8637699821 -19.2660166626 -1.3296899674
H 7.6048747497 -19.6084173246 0.3879523597
H 11.0028969818 -15.6151237021 -0.8039055064
H 12.9070703862 -14.0654157603 -0.7062587202
C 13.5224265199 -16.038493834 -0.0721375232
C 14.3600879715 -17.1020762034 -0.2895505689
C 15.1969462089 -17.1868377375 -1.4621274468

C 15.0896738923 -16.0758586836 -2.4247593175
C 14.221564579 -14.9809261876 -2.1742606134
C 14.1533337064 -13.9411024112 -3.1279390335
C 14.8860060693 -14.0067355903 -4.3006621069
C 15.7346981365 -15.0988929857 -4.5473135755
C 15.8411548819 -16.1118922622 -3.6102213251
H 12.9043070834 -16.007239166 0.8298646124
H 14.4430614693 -17.9107188634 0.4365802383
O 15.979938962 -18.1455226049 -1.6632435324
H 16.4940829135 -16.9687344134 -3.7668506845
H 16.3053534156 -15.1450371764 -5.4734833605
H 14.8027709132 -13.2075439944 -5.035806056
H 13.4914860413 -13.0974690248 -2.9349749885
K 12.5978053618 -12.6534211208 1.7936827525
K 18.2758754746 -18.6034163351 -0.320855048
N 14.5821598964 -14.5558079569 2.8386502818
C 15.3681244071 -14.3671338474 1.9961096206
C 16.3086996585 -14.1104702792 0.9309177676
H 17.3290123745 -14.3198140621 1.2673636095
H 16.0874121081 -14.7751934586 0.085277866
H 16.2182231807 -13.0659316173 0.6115532103
N 15.1314794166 -11.0953090458 2.5386490931
C 14.8155706149 -10.0352303957 2.1691061998
C 14.4006129002 -8.7327357309 1.6984029209
H 14.5713396212 -7.9726975181 2.4670440804
H 14.9641965224 -8.455472212 0.8013938336
H 13.3303382981 -8.7627354335 1.4589605767
N 13.971527502 -11.5975832819 -0.5810167619
C 13.3326598144 -10.7765559788 -1.1094302014
C 12.5510981186 -9.7452954443 -1.754374664
H 12.1005505794 -10.1220303973 -2.6786292472
H 11.7506473737 -9.4323486997 -1.0748092588
H 13.1790432419 -8.8802995841 -1.992751748
N 11.3088943206 -10.0483853527 1.3123863968
C 10.3658840836 -10.1881122769 1.9863421413
C 9.2079801061 -10.3689048134 2.8282053811
H 9.4845183461 -10.9889562393 3.6864719818
H 8.834346558 -9.4013447539 3.1777470605

H 8.4325907247 -10.8910549328 2.2573910686
N 12.3451677471 -11.6510686803 4.49598246
C 13.3343779615 -11.9840653563 5.0159667624
C 14.5761579655 -12.393942149 5.6331272605
H 14.5756169485 -13.4749584897 5.8013526297
H 15.4061463491 -12.1479943442 4.9621577421
H 14.719574714 -11.8831270086 6.5901614352
N 18.5763091718 -15.9428139366 -1.3408414649
C 18.2231195277 -14.905841895 -1.7367092236
C 17.7763344815 -13.6126132465 -2.2071685164
H 16.6832163582 -13.5366164392 -2.1314057829
H 18.2316667667 -12.812041076 -1.6145478269
H 18.0507264038 -13.4723366604 -3.2571865241
N 19.857008122 -19.8309566144 1.7064767616
C 20.2874760337 -20.3978657322 2.6271073492
C 20.8218368265 -21.1029153079 3.7718180407
H 21.7296420713 -21.6484651698 3.4965017216
H 21.0704833132 -20.3995757946 4.5724836158
H 20.0889254167 -21.8199937046 4.1542108958
N 17.2726977581 -17.0022662338 1.8465284582
C 16.3915132652 -17.3985036353 2.4990222426
C 15.2883919015 -17.8894773473 3.2931413925
H 15.5551186302 -17.9052737128 4.3544579562
H 14.4190150817 -17.2352125559 3.1651160416
H 15.0392800945 -18.9076636314 2.975140961
N 16.0673485303 -20.2746201255 0.9723322562
C 15.3221433841 -20.4083157586 0.0839412758
C 14.3820379752 -20.5744324385 -1.0022352521
H 13.8572080715 -21.5320128898 -0.9187114312
H 13.6477432449 -19.7597530186 -0.9902410981
H 14.9061188772 -20.5203303976 -1.960269833
N 19.6255676401 -20.3666318957 -2.1321867633
C 20.2507273316 -21.0471791076 -2.8397839673
C 21.0286166675 -21.8925437432 -3.7190474038
H 22.083522336 -21.6026678171 -3.6925198457
H 20.9488364827 -22.939913168 -3.412185626
H 20.6709094476 -21.8046730526 -4.7494994163

TS_A-B_ortho_2Kcation_MicroSolvation_acetonitrile

C 12.2123622777 -15.5538598244 -1.2775955183
C 13.9152065176 -16.8098775041 -1.2448749306
C 14.5528831762 -16.7315582277 -3.5876882816
C 12.3769208855 -14.33296487 -4.8327619891
C 11.3687110677 -15.0918051099 -5.3690842299
C 10.5573613596 -15.9247258478 -4.5962643237
C 10.7577705742 -16.0669276486 -3.2549794894
C 11.8274791483 -15.3544370838 -2.6506195859
C 12.6178035951 -14.4348726057 -3.4365054502
C 9.981363637 -16.1751598168 -6.719469427
H 12.97752291 -13.6906119459 -5.4680041949
H 10.1143891361 -16.7132478585 -2.6699530671
H 9.113113325 -15.8267287372 -7.2877281877
O 10.9581997637 -15.1345574259 -6.6671852627
O 9.591019346 -16.486909178 -5.3937555894
H 10.4331717728 -17.0628096509 -7.1949810413
N 13.5586574872 -13.6827595701 -2.7588067078
S 14.6982424849 -12.8960973212 -3.5465011394
O 15.379451384 -13.6338062036 -4.6309151821
O 15.6013727532 -12.3421450489 -2.5015954695
C 9.7506977257 -17.5147789317 0.8847978735
C 9.812331047 -16.7726854692 2.0606372886
C 10.5214890596 -17.1655955603 -0.2192742669
H 9.2120340884 -17.0585530701 2.9219551664
H 10.4908176546 -17.7985349545 -1.101556441
C 10.6660842664 -15.673597976 2.1312412813
C 11.3663988104 -16.0433490807 -0.1804422826
H 10.7424902887 -15.0881895677 3.0471434423
C 11.4314137334 -15.3259977128 1.0276872084
H 12.0890924234 -14.4573578336 1.0899283805
H 9.1025328647 -18.3872212861 0.8259047388
C 14.4695296106 -10.9687958267 -5.5180119444
C 13.9515349587 -9.7954345399 -6.0581133863
C 13.9544969791 -11.4597957611 -4.3196516385
H 14.3514584125 -9.4130724767 -6.997250791
C 12.9138221664 -9.1026765684 -5.4245379517
C 12.9114697869 -10.7933451354 -3.6771214135

H 12.5031824209 -11.2001105875 -2.7535497386
C 12.4026947528 -9.6236005557 -4.2288335506
H 11.5851273079 -9.1050632448 -3.7280045591
H 15.256311749 -11.5228174585 -6.0276414494
C 12.3353292435 -7.8608192732 -6.0300244133
H 13.0632911035 -7.3354377694 -6.6574174178
H 11.474663647 -8.0974862752 -6.6694993417
H 11.978258597 -7.1624162141 -5.2652094543
H 12.8275190029 -14.7250600618 -0.9408795466
H 15.1078894332 -16.2725035791 -4.4042737428
C 14.7552001863 -16.3264081589 -2.3006425663
C 13.2231188778 -18.0806799514 -1.4326371451
C 12.9322762822 -18.4367628713 -2.8287309612
C 13.5721785962 -17.7353675681 -3.8884345551
C 13.2301393372 -18.074774097 -5.2134913795
C 12.2884579228 -19.058792498 -5.4756151679
C 11.6669943212 -19.7500466687 -4.425844146
C 12.0027424138 -19.4442764813 -3.1150620974
H 15.5008999147 -15.5625645699 -2.084344792
H 14.2025119816 -16.6033055673 -0.2113503026
O 12.8266026323 -18.7739171809 -0.47838077
H 11.5434791262 -19.9624789717 -2.2730899666
H 10.9210767321 -20.5129944389 -4.6405912082
H 12.0317254157 -19.2981891601 -6.5071132678
H 13.7085974659 -17.5383569831 -6.0337731242
K 14.7545680468 -13.1843480093 -0.0985027359
K 13.5651596923 -20.4989236657 1.3668025056
N 11.3741449959 -22.2620652609 1.9406122637
C 10.46396678 -22.8770670474 2.3252001185
C 9.3322399031 -23.6408429548 2.8039033084
H 8.5188004556 -22.9721488944 3.1017399932
H 8.96255447 -24.3098190567 2.0207204783
H 9.6178544064 -24.2454449965 3.6701503059
N 15.2005812366 -22.411667981 2.7379389745
C 15.9066372359 -23.1282286721 3.3230371256
C 16.7843892667 -24.0187841997 4.0510101709
H 16.3673357588 -24.2452310643 5.0370972974
H 16.9149471854 -24.9584065673 3.5054534532

H 17.7674398504 -23.5581175798 4.18864116
N 14.7579507029 -21.2425839181 -1.2050913689
C 14.7843502178 -21.2292861601 -2.3701378841
C 14.8328670998 -21.2077335533 -3.8160104387
H 13.9745278221 -21.7438760006 -4.2325965021
H 14.786320553 -20.1743791784 -4.1819588013
H 15.7527856957 -21.6779652459 -4.1781263729
N 15.8955803036 -18.7281691319 1.1078489579
C 16.3952960029 -18.6796229493 0.0558948095
C 17.0220934606 -18.6263964383 -1.246173412
H 17.5817085493 -19.5480853769 -1.4356409496
H 16.2615754677 -18.4976730919 -2.0254418627
H 17.7034298285 -17.7712536375 -1.3026175035
N 13.013819671 -18.3680556608 3.1941501256
C 13.6448023136 -17.401211018 3.0359718946
C 14.440496408 -16.2076472824 2.8534488518
H 13.8017230914 -15.3440754098 2.6340723776
H 15.0100446179 -15.9879428282 3.7625207866
H 15.1518131589 -16.3609157927 2.0332540862
N 13.4152067164 -12.768993353 2.3957773082
C 12.926191043 -11.7228823847 2.5539595786
C 12.3238271869 -10.421693998 2.7287379285
H 11.2485591013 -10.5172437962 2.9080970898
H 12.7760900986 -9.8991142335 3.5770795802
H 12.4856037315 -9.8360886197 1.8173777346
N 16.9014115514 -11.6121627602 0.9326849046
C 17.795465018 -10.8654574189 0.9169585992
C 18.9146158467 -9.9513395315 0.8725956275
H 18.565801187 -8.9136228441 0.8895193039
H 19.5753103387 -10.1052820534 1.7317049928
H 19.474181415 -10.126639229 -0.0530714676
N 16.8830024101 -15.0924623194 0.1906632638
C 17.9473456982 -14.616101429 0.213632622
C 19.2653589216 -14.0237092099 0.2430212253
H 19.485824108 -13.6518579041 1.2490873703
H 20.0246636314 -14.7636587917 -0.029152286
H 19.3122598863 -13.1855006894 -0.4651611965
N 18.9352458592 -11.5234397325 -1.9834200449

C 18.500526851 -11.6113513083 -3.0618446064
C 17.9786435725 -11.7000506523 -4.4053010493
H 17.2927347914 -10.8654580437 -4.5914450037
H 18.7931150488 -11.657432499 -5.1356524058
H 17.4052579765 -12.6263046497 -4.5351033091
N 13.8627542293 -10.3558364794 -0.252238458
C 14.6758623013 -9.9245230035 -0.9685578313
C 15.6950055122 -9.3831628874 -1.8397006441
H 15.9640022153 -8.3679303393 -1.5290906793
H 16.5793453921 -10.0296977388 -1.8083082114
H 15.3263281695 -9.3566540619 -2.871655681

TS_A-B_ortho_2Kcation_MicroSolvation_acetonitrile_conf2
C 11.6719402985 -14.4859665279 -1.783925311
C 13.300488092 -15.883354037 -1.2524301081
C 14.2630674461 -16.5401684244 -3.3865423354
C 12.6091056965 -11.8943801491 0.7705150375
C 13.0757373022 -10.9089357465 -0.0687858028
C 13.038823426 -11.029503641 -1.4574578655
C 12.5647221441 -12.1601160847 -2.0631575148
C 12.1210062652 -13.2320892041 -1.2417052713
C 12.1095109122 -13.0847632112 0.1918131136
C 13.9130575075 -9.0434711983 -0.9551991734
H 12.5918217737 -11.7394641041 1.8448252766
H 12.4970994194 -12.2181836078 -3.1436222428
H 13.4066443191 -8.0726380042 -1.0120083476
O 13.5568998267 -9.6854017418 0.2734569739
O 13.4885824152 -9.8694349249 -2.0300891826
H 15.0045611812 -8.9161000557 -0.9900895216
N 11.4974816775 -14.0797582461 0.9458279626
S 12.1852600072 -14.729578775 2.2144173923
O 13.5504819415 -15.2492410409 1.9768579135
O 11.2159706008 -15.7300836348 2.7407821575
C 11.8113654715 -14.8324846247 -5.5674403928
C 10.6961185032 -15.6367004905 -5.7800166295
C 12.1561338568 -14.4272289618 -4.2810122954
H 10.4376085059 -15.9578505001 -6.7871447807
H 13.0693517896 -13.8570412056 -4.1335396228

C 9.9230902354 -16.0447325156 -4.6914814901
C 11.3736658145 -14.8037145985 -3.1732850804
H 9.0667807524 -16.7014069931 -4.8404105974
C 10.263076469 -15.6373426404 -3.4123845684
H 9.6782269638 -15.9784568908 -2.5580526995
H 12.4338594251 -14.5312805309 -6.4079074552
C 13.628307205 -13.2464887642 4.0566737985
C 13.7476111694 -12.3258781436 5.0954379534
C 12.3685100428 -13.5418417775 3.5440543651
H 14.7335021315 -12.096600281 5.4995444698
C 12.6246546938 -11.6862752305 5.6303287028
C 11.2318938697 -12.9149220201 4.059442448
H 10.2480856756 -13.1588080542 3.6571766856
C 11.3661660404 -11.9998325031 5.0950637063
H 10.4786619287 -11.5153699065 5.5024443173
H 14.500278935 -13.7485767096 3.6434291463
C 12.7584916766 -10.6757505857 6.7275412494
H 13.7263251011 -10.749724008 7.2338124034
H 12.6756860849 -9.653271575 6.3363316209
H 11.9714567871 -10.7871215601 7.4820969003
H 11.04945831 -15.0305881591 -1.0783876415
H 14.9778089568 -16.3920197779 -4.1956724654
C 14.2774685544 -15.7412245818 -2.2797264806
C 12.4398211307 -17.047724741 -1.22429189
C 12.3975460354 -17.8572751214 -2.4422465357
C 13.2974847971 -17.5948556132 -3.5110444859
C 13.2181757939 -18.4011864405 -4.6627268584
C 12.2839864788 -19.4211304774 -4.7563653006
C 11.3960297408 -19.6710094814 -3.6984466134
C 11.4604900532 -18.8930269797 -2.5548882464
H 15.0030924607 -14.9307395142 -2.2005097771
H 13.4510857652 -15.3923769191 -0.2942865978
O 11.7014926453 -17.308961357 -0.2392001323
H 10.7831562218 -19.0464125777 -1.7182691913
H 10.6560377411 -20.465774792 -3.7822882938
H 12.2374815277 -20.028792814 -5.6586572445
H 13.9018398152 -18.2078831663 -5.4895286844
K 9.4277481313 -16.2959679775 0.7154114731

K 12.5175072785 -18.1668556 2.2736475512
N 14.1260724418 -16.8813496341 4.6728507624
C 13.2775349806 -16.3483627761 5.2702310261
C 12.211526022 -15.7004118927 6.0038093405
H 11.3500898661 -15.5783396558 5.3377200339
H 11.9145147433 -16.3064318044 6.8655424705
H 12.5302681032 -14.7096992121 6.3472781515
N 13.8669582652 -20.4098281464 3.6090823312
C 14.6452444794 -20.8798040421 2.8802006832
C 15.6099720812 -21.4501099801 1.96734792
H 15.9711291381 -20.6619990124 1.2969991766
H 16.4587340264 -21.8720098996 2.5144756084
H 15.1433242892 -22.2417564347 1.3722992571
N 11.0526698607 -18.5330287826 4.9824434978
C 11.7017020567 -19.2658383709 5.6157031034
C 12.5216640829 -20.1466705027 6.4166541026
H 13.5549273659 -19.7853859816 6.4138525825
H 12.1516878523 -20.1870249952 7.4460319885
H 12.5186736435 -21.1544393256 5.9926702797
N 12.7309365193 -20.5096078039 0.2974507025
C 13.5268897549 -20.3681829617 -0.5437544538
C 14.5304325253 -20.196140437 -1.5706693128
H 15.2802253781 -20.9927384237 -1.5110222399
H 14.0682036703 -20.2172916262 -2.5645304926
H 15.0292506889 -19.2306045214 -1.4303181106
N 15.3030840775 -18.234048874 1.0431096544
C 15.7718696942 -17.2886180961 1.5407112221
C 16.3560394359 -16.1270674041 2.1695464767
H 16.0934326594 -15.228088789 1.604634086
H 15.9426360337 -16.0314682769 3.179531616
H 17.4446404535 -16.2274705579 2.2222836485
N 8.1574464525 -14.2182891126 2.9680504689
C 8.2660177041 -15.2220916355 3.55448756
C 8.4022041223 -16.4911283158 4.2330406466
H 8.1296479931 -16.3971487597 5.2892392054
H 7.7456590368 -17.2348975562 3.767095908
H 9.4332077057 -16.8591745374 4.1656538018
N 6.5173587694 -16.559889608 1.0622987539

C 5.9898881187 -15.5197935786 1.0723290363
C 5.3593162315 -14.2189023517 1.0931800248
H 5.9354464906 -13.5565879598 1.7493811315
H 5.3433290082 -13.7896091932 0.0859173366
H 4.3334471197 -14.2891716808 1.4676144438
N 8.2292120048 -14.0676950987 -0.7306185721
C 8.6017270834 -13.1079120626 -0.1814046896
C 9.0447441864 -11.9204645311 0.5119431064
H 9.6493279832 -12.2195187895 1.3747762098
H 9.6583830143 -11.2989387086 -0.1486637107
H 8.1839490292 -11.3396262711 0.8591316369
N 8.2619310286 -18.0505120769 -1.5198825007
C 7.4249785136 -17.3485757769 -1.9283245234
C 6.4076752562 -16.4516867049 -2.4297056335
H 5.4643017571 -16.6199224053 -1.9003752009
H 6.7308363234 -15.4169389088 -2.2649787074
H 6.2453432093 -16.6100829399 -3.5004792817
N 9.6283817603 -18.9460627907 1.7644865087
C 9.5157522001 -19.8079594213 0.9873696883
C 9.3796315767 -20.8689400817 0.0169452733
H 8.7951752839 -21.6969116309 0.4298684274
H 8.8733672694 -20.4784561769 -0.8726762148
H 10.3753247593 -21.2338935927 -0.2585241365

TS_A-B_ortho_2Kcation_MicroSolvation_acetonitrile_conf3
C -1.7550458263 1.3793740227 -0.4525055706
C -0.9652299863 2.1224084644 1.3823828834
C 1.1695516385 3.1925152264 0.8793665494
C -2.4913858495 -2.2774192678 0.0822741072
C -3.8450223221 -2.1959813839 0.26517484
C -4.5421852991 -0.9802265838 0.2360002382
C -3.9027711774 0.1982558291 -0.0031670574
C -2.4882268585 0.1885493772 -0.1845479761
C -1.7591819327 -1.0682365006 -0.1355747914
C -6.0075749835 -2.6187739089 0.6178546519
H -2.001394883 -3.2442501738 0.0449205883
H -4.4675114205 1.1193576685 -0.101641429
H -6.6656685422 -3.0198660551 -0.163589562

O -4.7134744444 -3.2130005278 0.4834872992
O -5.873966662 -1.2123739231 0.4562137241
H -6.3955410066 -2.8386506125 1.6207646962
N -0.4169523051 -1.0096858074 -0.3149249606
S 0.4435470667 -2.362681397 -0.2024691553
O 0.1233011386 -3.3823130714 -1.2238204388
O 0.4720292855 -2.9148170961 1.1870898456
C -3.8906320044 4.5189958959 -0.7876491687
C -3.3688037796 5.0588518912 -1.9618604138
C -3.3930895886 3.321810285 -0.2854477246
H -3.7594158793 5.9950489007 -2.3549547352
H -3.7896007985 2.9338100432 0.6534892872
C -2.3364049067 4.3960981137 -2.6200414632
C -2.3637937879 2.6301948252 -0.9451512512
H -1.9138274659 4.8125536107 -3.5322760391
C -1.8342203931 3.2035676425 -2.1100829925
H -1.0181264712 2.6942134103 -2.6217749952
H -4.688164684 5.0366440419 -0.2573677282
C 3.1412336151 -2.6016243103 -0.2054752486
C 4.442405683 -2.2155157647 -0.5025812693
C 2.0799125032 -1.7658024886 -0.5480528728
H 5.2733073635 -2.865878598 -0.2292394707
C 4.705490874 -1.0015710772 -1.1450757787
C 2.3153298456 -0.5546680232 -1.1935595047
H 1.4768617234 0.090774311 -1.4409944608
C 3.6220381285 -0.1835321794 -1.4834043661
H 3.8083475305 0.772832214 -1.9728742126
H 2.9449938215 -3.5438082411 0.3052670272
C 6.1070317234 -0.5793542088 -1.463630873
H 6.8476717128 -1.2251023154 -0.9801188121
H 6.3013100957 -0.6122123765 -2.5434401159
H 6.2992509447 0.452779881 -1.1435618209
H -0.7662091523 1.186597418 -0.8688780885
H 1.7435465486 4.0373574926 0.499047609
C -0.1887577654 3.2523215589 0.9588596524
C -0.308503262 0.9872264635 1.9857685989
C 1.1511205239 0.9227330085 1.8578341138
C 1.8744768938 2.0266400528 1.3213601057

C 3.2817325952 1.957800456 1.2900103017
C 3.958042698 0.8549878939 1.784052073
C 3.242012508 -0.2234127309 2.3236767881
C 1.8589068332 -0.1831579392 2.3510014428
H -0.7164744712 4.1478646208 0.6277067888
H -1.9782938843 2.3009112555 1.7449344696
O -0.9628866349 0.1174724816 2.6333159543
H 1.2957383724 -1.0369033942 2.7205099992
H 3.7660195835 -1.1034390459 2.6940188838
H 5.0466260723 0.8182339819 1.7387424805
H 3.8366508028 2.7955795738 0.8638347572
K -1.560251016 -2.5344065966 3.0159593433
K 0.3148358639 0.3040974747 5.1295099093
N -1.8406284848 -5.210436109 1.7281017783
C -1.121998258 -5.6907821977 0.945158949
C -0.2219361092 -6.2526934788 -0.0346893754
H 0.1570591794 -5.4357965955 -0.6646891775
H 0.616015433 -6.7577520233 0.4574613685
H -0.7447640612 -6.9795337903 -0.6640698872
N -1.9042805072 -4.7155183318 4.9947751721
C -2.9233540536 -5.0835322503 4.5621671858
C -4.1758657289 -5.515648208 3.9834793795
H -3.9834042497 -6.2609731641 3.2062334111
H -4.8313040541 -5.9448703875 4.747420501
H -4.6750307078 -4.6537211866 3.525164953
N 1.0630194659 -2.4573862411 4.7595371269
C 1.1988450781 -3.5126350325 4.2832308835
C 1.3114038572 -4.8044515273 3.6469811003
H 0.446248135 -5.4191726397 3.9221056961
H 1.3166554942 -4.6540411369 2.5602045142
H 2.2290161175 -5.3142485686 3.9560160102
N -2.1206215821 -1.2020058624 5.5455276542
C -2.6108207092 -0.711470854 6.4816532795
C -3.2239823411 -0.0732833657 7.6227577848
H -4.2907418872 -0.3144398595 7.6689887991
H -2.7449200547 -0.4044785682 8.54861269
H -3.0970688597 1.0103455033 7.5298017744
N -4.5933199226 -2.1349748152 3.4382870847

C -4.4223056583 -0.9806780408 3.4142939035
C -4.1467935683 0.4373923139 3.3825881447
H -4.8349076166 0.9398492889 2.6940059409
H -4.2489094479 0.8702773109 4.3835412955
H -3.1071860514 0.5834868881 3.0440294866
N 1.1428760741 3.0278224066 4.5732946792
C 2.2840403379 3.2424561929 4.4716814664
C 3.6985206926 3.5161323964 4.3679728693
H 4.2238775464 2.6114216885 4.0437697616
H 3.8821845798 4.3033330806 3.6294463674
H 4.0826728214 3.8390626484 5.3425101171
N 2.8386306358 3.4675234834 7.8205786799
C 1.7336450984 3.8064071253 7.6643286621
C 0.3622967714 4.2251756736 7.4732096586
H 0.3258894218 5.1176219674 6.8396155825
H -0.2094516356 3.4275261195 6.9860678772
H -0.103009729 4.4584066406 8.4362985237
N -2.08936872 2.2927592717 5.4208367341
C -1.9657232184 3.2104430454 4.7111832318
C -1.7945044314 4.356479234 3.8463522263
H -0.8192344656 4.2908082416 3.3497729376
H -2.5751609786 4.3863737545 3.077870349
H -1.843538764 5.2840495594 4.4265391263
N 0.0604252126 0.8223200079 7.9744879058
C 0.9898372404 0.7030388142 8.6683088273
C 2.146589819 0.5636831728 9.5241630622
H 2.784394477 1.4491192148 9.421039633
H 1.8431752953 0.4631007277 10.5708366849
H 2.7184929887 -0.3251337988 9.2383154104
N 3.0376039528 0.5168085176 6.1156109616
C 4.046187698 0.8468564118 6.5966776325
C 5.282626343 1.2843761515 7.2067072086
H 6.060248752 1.4248603393 6.4488219614
H 5.1080050231 2.2353076701 7.7225565311
H 5.6416197691 0.5444843837 7.9295222416

TS_A-B_ortho_2Kcation_MicroSolvation_acetonitrile_conf4

C -1.0933726564 1.5460060082 0.9118819323

C -0.553215074 2.7848676424 2.5555551895
C 1.5736791459 3.6496943175 1.7674315925
C 1.5648210715 2.2681980866 -1.6513038454
C 2.3549570696 1.1633077873 -1.4867961293
C 2.0331810914 0.1392991787 -0.5931924949
C 0.8976681703 0.190382493 0.1622073858
C 0.0656112375 1.3418513959 0.0830655582
C 0.3950304699 2.3977771754 -0.8478551617
C 4.026993431 -0.2845270626 -1.4549505525
H 1.7898839914 3.0111768787 -2.409194239
H 0.6490743739 -0.6345575924 0.819248585
H 4.3966334103 -1.0129448495 -2.1815464799
O 3.4971092585 0.8497309938 -2.1591466683
O 2.973003272 -0.8591448609 -0.699633431
H 4.827485024 0.070359605 -0.7866147209
N -0.4458145386 3.4701892223 -0.9191974465
S 0.0669287851 4.7925637206 -1.6497929734
O -0.0055351361 4.7404699559 -3.1336249663
O 1.3793204573 5.2915172937 -1.1432663868
C -2.3435030874 -1.6462719218 2.5968394703
C -3.7118144539 -1.5239809804 2.368521603
C -1.4635720536 -0.6527447262 2.1810573607
H -4.3936739131 -2.3106193426 2.6867926694
H -0.4083306148 -0.7428179589 2.4264491481
C -4.2004049145 -0.3771399414 1.7451608171
C -1.9285249286 0.4845807225 1.4947549762
H -5.2686967342 -0.2585266819 1.5737734093
C -3.3200806058 0.6121675488 1.3258125063
H -3.7040159238 1.4980118677 0.8198375072
H -1.9597021887 -2.5213199749 3.1193750204
C -0.8075986086 6.964860452 -0.2122116632
C -1.7695854481 7.891726484 0.1830191338
C -1.1558421363 5.9638011991 -1.1134119174
H -1.5013360097 8.6778606805 0.8885337114
C -3.0760936022 7.8324031678 -0.3083615518
C -2.451299303 5.8864021336 -1.624811772
H -2.7037307753 5.1046153244 -2.3380743376
C -3.3986657026 6.8147119815 -1.2175208358

H -4.411926745 6.7568879485 -1.6147874891
H 0.21170182 7.0088923961 0.1685272959
C -4.110617315 8.8291168194 0.1158060003
H -3.7089331555 9.5519030322 0.8331707663
H -4.5010435726 9.3929029634 -0.7406369904
H -4.972585459 8.3388339726 0.585115082
H -1.6982100206 2.3673347144 0.5276347082
H 2.1578698647 4.4124452961 1.2517811917
C 0.2344100301 3.8233052273 1.9615838041
C 0.114841358 1.7795755565 3.3608810851
C 1.5131947858 1.5193690669 3.0128415352
C 2.2275194301 2.4540465156 2.2152545239
C 3.5742866291 2.1738091939 1.9128914801
C 4.1794480045 1.0062996075 2.3503097086
C 3.466929737 0.0849059743 3.1307479285
C 2.151402303 0.3590108736 3.4664566066
H -0.2646052261 4.713272838 1.5825308709
H -1.5757729926 3.0143571886 2.8582712259
O -0.4752296772 1.1197419703 4.2486334065
H 1.5773448483 -0.3341536045 4.0822624151
H 3.9447646529 -0.8297746659 3.4769088192
H 5.2173494187 0.8091188991 2.0835573616
H 4.1521135096 2.8963977199 1.3368869255
K 3.9442018606 4.3888460596 -1.2472350539
K 0.6323443902 0.7081779648 6.6687610916
N -2.1432305486 0.9862748002 7.3979128007
C -2.7283483452 0.8003742085 6.4057006873
C -3.4194461443 0.5586945915 5.1615149144
H -4.2961929851 1.2070367211 5.0671170916
H -2.7192336464 0.7544344223 4.3383723392
H -3.7405746443 -0.4860901666 5.1005881097
N 0.5128233741 -0.847841383 9.117185901
C -0.5853148161 -1.2397372649 9.1237113989
C -1.9453105313 -1.7265716207 9.1144962533
H -2.6314844944 -0.9165910736 9.378287686
H -2.1907409298 -2.0757620506 8.1060056013
H -2.0649173378 -2.5508206686 9.8243915096
N -0.6497639241 -1.8881600555 5.8815134109

C 0.0801516862 -2.4179651489 5.1422995838
C 0.9932475922 -3.0727427727 4.2299585505
H 0.8155435892 -4.1527466025 4.2155261673
H 0.8703437505 -2.6850398762 3.2121134018
H 2.0225566488 -2.8831564169 4.5535145351
N 2.7452150431 -1.3361045913 6.5059993371
C 3.164535436 -1.5676735916 7.5691159211
C 3.6655761385 -1.8428655643 8.8965206376
H 2.9004334237 -1.5608336675 9.6275424321
H 3.8909329907 -2.9077884942 9.0094991097
H 4.5776355646 -1.2703422821 9.090956322
N 2.4278854281 2.8105486664 6.0612217498
C 2.9421222918 3.5925578703 5.3682497566
C 3.5731095806 4.5734415233 4.5130850981
H 2.8515764752 4.9442930443 3.7763106538
H 3.9452587552 5.4149268605 5.1060859226
H 4.4068268033 4.1250687754 3.9638239948
N 3.8440990613 7.3715969892 -1.9272723774
C 2.8148594404 7.4868364175 -2.4648490415
C 1.5508560091 7.6382793763 -3.1460952581
H 1.1984487015 6.6639182382 -3.5079140105
H 0.7948719504 8.0246263557 -2.4533876432
H 1.6490598373 8.3338728324 -3.9858670811
N 6.7101014126 5.4912044693 -2.0212664169
C 6.4304203156 6.0320057515 -3.0159310822
C 6.0669919416 6.7000411066 -4.2441995628
H 5.8510081855 7.7543336893 -4.0461668743
H 6.8763099199 6.6286144062 -4.9772425796
H 5.166706509 6.2273441731 -4.6516740301
N 5.114241167 5.5049598606 1.1826369174
C 5.6258227454 6.4927141399 0.8302991203
C 6.2505743512 7.7144626374 0.3775350222
H 5.7032190988 8.0804673874 -0.4984796007
H 6.2297016173 8.4747201391 1.164359233
H 7.2891317607 7.5238080938 0.089304627
N 5.9668961314 2.4447910629 -0.4592519885
C 6.9291718748 2.9856360635 -0.0838854295
C 8.1144409583 3.6753618382 0.3713725855

H 8.3011080485 4.5257501486 -0.2931655221
H 7.9677320269 4.0447862506 1.3913784184
H 8.9821447355 3.0088461956 0.3566916888
N 3.4999778598 4.4410283633 -4.1044701215
C 2.6614557731 3.8165360491 -4.6219514718
C 1.6076015346 3.0316884814 -5.2218152141
H 0.6577913404 3.3108641606 -4.7494800277
H 1.5514594272 3.21571992 -6.2990569869
H 1.7947337108 1.9655062586 -5.054513472

TS_A-B_ortho_2Kcation_MicroSolvation_acetonitrile_conf5
C -1.161567 1.293637 0.17376
C -0.728521 2.417571 1.968955
C 1.136288 3.928947 1.541196
C -4.606687 2.721651 -0.320983
C -4.385896 3.44093 -1.466607
C -3.1623 3.431546 -2.133192
C -2.094391 2.727327 -1.655382
C -2.248117 1.993382 -0.446466
C -3.534313 1.954217 0.213784
C -4.575626 4.730776 -3.278349
H -5.585082 2.686095 0.142291
H -1.161068 2.69377 -2.207608
H -5.08004 4.370526 -4.184953
O -5.26953 4.235477 -2.12952
O -3.240488 4.241924 -3.243723
H -4.567857 5.828353 -3.245824
N -3.642059 1.172442 1.331524
S -4.847013 1.413659 2.350608
O -6.228835 1.228829 1.839814
O -4.666565 2.709448 3.078368
C 2.203336 1.715062 -1.54505
C 2.738958 0.443681 -1.362707
C 0.928326 2.015376 -1.077551
H 3.73697 0.212467 -1.729407
H 0.549634 3.028832 -1.178134
C 1.993759 -0.527958 -0.695303
C 0.156459 1.046875 -0.411554

H 2.40855 -1.521542 -0.536514
C 0.727219 -0.225097 -0.217598
H 0.158537 -0.973012 0.335382
H 2.788199 2.485616 -2.044359
C -5.571101 -0.783591 3.828201
C -5.350803 -1.756981 4.801901
C -4.560327 0.126498 3.538022
H -6.132318 -2.484449 5.019711
C -4.142896 -1.819314 5.502999
C -3.345215 0.088941 4.222678
H -2.540748 0.771501 3.951622
C -3.152481 -0.871269 5.205845
H -2.212871 -0.911323 5.757467
H -6.510109 -0.730961 3.281617
C -3.879743 -2.881298 6.524831
H -4.764541 -3.497691 6.713342
H -3.070232 -3.546604 6.19335
H -3.548745 -2.451421 7.477617
H -1.49086 0.496736 0.84208
H 1.525474 4.893478 1.212527
C -0.20595 3.68369 1.562591
C 0.152172 1.424644 2.546428
C 1.592918 1.669984 2.431836
C 2.07197 2.924446 1.965996
C 3.463402 3.135724 1.923194
C 4.348656 2.134082 2.285576
C 3.870591 0.881612 2.696141
C 2.506508 0.662186 2.772823
H -0.907736 4.453618 1.235738
H -1.7767 2.340878 2.262831
O -0.290956 0.364571 3.057245
H 2.11286 -0.314514 3.05787
H 4.568476 0.080404 2.935273
H 5.420889 2.315645 2.231345
H 3.840331 4.099161 1.578749
K -4.217304 5.176781 2.178655
K 0.617763 -0.167464 5.540717
N -1.105657 2.053296 6.04593

C -2.216427 2.3987 6.118451
C -3.605659 2.788979 6.182067
H -4.002569 2.895444 5.163149
H -4.181213 2.019265 6.708982
H -3.721724 3.742207 6.707906
N -0.494388 -1.817981 7.600781
C 0.013812 -2.849724 7.40644
C 0.645526 -4.120064 7.133451
H 0.283863 -4.890928 7.820642
H 0.422436 -4.411571 6.10181
H 1.730978 -4.027572 7.238153
N -0.341964 -2.708252 4.267238
C -1.080272 -2.502111 3.388027
C -2.005892 -2.234542 2.309955
H -3.013103 -2.570929 2.579348
H -2.040015 -1.152592 2.129072
H -1.694944 -2.749242 1.394966
N 3.120511 -1.434135 6.028142
C 4.027727 -0.704206 6.087211
C 5.144443 0.210981 6.157363
H 5.458249 0.349108 7.196627
H 5.994036 -0.170635 5.582512
H 4.834716 1.181003 5.754208
N 2.432613 2.113787 5.857563
C 1.841931 3.052638 5.497393
C 1.099615 4.208753 5.049177
H 0.341227 3.913548 4.314705
H 0.585729 4.679789 5.892798
H 1.77095 4.936439 4.581673
N -5.943349 5.132593 4.888323
C -6.560364 4.153783 4.738573
C -7.323084 2.937447 4.568725
H -7.265211 2.599172 3.527488
H -6.903733 2.142342 5.195852
H -8.368311 3.09459 4.85397
N -4.86429 7.842169 3.060678
C -6.010256 7.835833 3.276092
C -7.430235 7.804323 3.542933

H -7.607248 7.624507 4.607801
H -7.901007 8.749032 3.254518
H -7.882688 6.986526 2.971927
N -2.30551 5.349552 4.349543
C -2.779304 6.147844 5.056697
C -3.392792 7.120164 5.933261
H -4.454079 6.871049 6.044799
H -2.910033 7.116404 6.915712
H -3.313978 8.120991 5.498439
N -2.770986 6.069111 -0.08347
C -1.918086 6.081219 -0.876459
C -0.855319 6.077116 -1.856286
H -0.554822 7.0996 -2.105467
H 0.015795 5.544356 -1.458928
H -1.195084 5.572853 -2.767591
N -7.035084 4.946216 1.504973
C -7.578618 4.126562 0.878235
C -8.223555 3.090815 0.10291
H -7.876514 2.112794 0.457816
H -9.311696 3.150932 0.203544
H -7.962125 3.198834 -0.955346

TS_A-B_ortho_2Kcation_MicroSolvation_acetonitrile_conf6
C 12.7390892822 -15.2008969504 -1.5287860678
C 13.0487382005 -16.7023718483 -3.0272638384
C 11.7276283863 -16.1432736059 -4.9992102022
C 10.8217896027 -16.9068377665 1.2183538565
C 11.81650399 -16.9811635158 2.1535355373
C 13.1214964366 -16.5345010665 1.9174464418
C 13.470663801 -15.945227505 0.7407212108
C 12.4857692026 -15.8365073556 -0.281983747
C 11.1428435648 -16.3457176686 -0.0579012212
C 13.0346618249 -17.3654564026 3.986420673
H 9.8081134549 -17.2119288841 1.4611485141
H 14.4641745286 -15.5298443107 0.6042372929
H 12.9882609705 -16.7420971626 4.8902068968
O 11.7238212594 -17.458412555 3.4226936419
O 13.8916489986 -16.7619205543 3.0309834892

H 13.3887423204 -18.3777732244 4.2265412688
N 10.2850405165 -16.2463396884 -1.099654445
S 8.796717093 -16.8424916926 -0.9418083895
O 7.992760663 -16.1790780022 0.1079100773
O 8.7828394735 -18.3306910071 -0.8430860594
C 16.3110572616 -13.9425778564 -1.937662936
C 16.0787636244 -12.6653999282 -2.4459031047
C 15.2428293897 -14.7778780756 -1.6265140549
H 16.9141137988 -12.0119321169 -2.6879754193
H 15.4416675127 -15.7862887239 -1.2605375927
C 14.7692359114 -12.2378473802 -2.6493803959
C 13.9143079207 -14.3569702955 -1.8068630931
H 14.5751984275 -11.2467315459 -3.0544146482
C 13.7036166504 -13.0777531788 -2.3429391049
H 12.6807212322 -12.7421466262 -2.5122767426
H 17.3314032519 -14.2913004109 -1.7841548131
C 7.0215938728 -17.1834305587 -2.9707435777
C 6.3644832771 -16.8154567421 -4.1374101419
C 8.0890501173 -16.4139711513 -2.5120370506
H 5.5344423707 -17.4214484483 -4.5012335782
C 6.7611032522 -15.6884098798 -4.8655575816
C 8.4879119664 -15.2743250493 -3.2059597902
H 9.3213545845 -14.6841387592 -2.8337537749
C 7.8265077234 -14.9252790094 -4.3776246846
H 8.1472963023 -14.0419577772 -4.930418584
H 6.7275743571 -18.0764451754 -2.4218845805
C 6.091958669 -15.3397362414 -6.1591953224
H 5.0202117683 -15.5665419603 -6.1400140226
H 6.2090230363 -14.2796044447 -6.4076440191
H 6.5216322834 -15.9142609092 -6.9927506264
H 11.8289418703 -14.8595008475 -2.019828358
H 11.5287144998 -15.5077342927 -5.8621582622
C 12.8286398944 -15.9436803545 -4.2237535761
C 12.1985734514 -17.8307596813 -2.7213094027
C 11.081354313 -18.0965397538 -3.6290922859
C 10.8392930614 -17.2364414967 -4.7379513903
C 9.7522150614 -17.5191043358 -5.5880636122
C 8.9613337222 -18.6365045954 -5.3913978764

C 9.2456668142 -19.5216265216 -4.3403985411
C 10.2903952056 -19.2467699373 -3.4755783733
H 13.523603689 -15.1351276995 -4.4575181438
H 14.0658942555 -16.8005806188 -2.6479777816
O 12.4607492199 -18.5964825827 -1.7421020294
H 10.5192830192 -19.9318592414 -2.6614778706
H 8.6389124481 -20.4141444751 -4.1974812301
H 8.1227655066 -18.8329696038 -6.0586160492
H 9.5474993364 -16.8406401437 -6.4176919015
K 13.7872299713 -20.539958909 -3.1244537042
K 10.8451685952 -19.8203931511 0.1484975331
N 12.1217958468 -22.015835067 -1.3123714414
C 12.7292375049 -22.7933810039 -0.690480908
C 13.4867421467 -23.7482381195 0.081249979
H 13.6090877535 -24.6824852393 -0.476224054
H 14.477437754 -23.3263773101 0.285850222
H 12.9593688873 -23.9479294753 1.0198239992
N 10.9555075081 -22.5026792401 1.8929232195
C 10.2169205167 -22.5146473155 2.7952544347
C 9.2955130412 -22.5364552795 3.9085877092
H 8.69805202 -21.6184946753 3.8935936926
H 8.6271890264 -23.4003287197 3.8382701079
H 9.8476940983 -22.5817487451 4.8521883884
N 13.0795931612 -19.8771544655 1.8741515912
C 13.5030331238 -20.7047217416 2.5766362128
C 14.0458682457 -21.7352616161 3.4304483364
H 13.3109959548 -22.538816997 3.5415897004
H 14.9691016066 -22.1371017938 2.9988744625
H 14.2502827522 -21.3329104847 4.4268383003
N 11.6279696708 -20.4597222643 5.010280511
C 10.7694642315 -19.682227337 5.1470988194
C 9.692563937 -18.732664789 5.3234501894
H 8.9470760268 -19.1244464429 6.0233748634
H 10.0760305941 -17.7851592812 5.7137106803
H 9.2158497529 -18.5398979676 4.356824315
N 8.6751986472 -19.6814475516 2.2046989611
C 7.6907950632 -19.2430556716 1.7540569492
C 6.470708321 -18.692418255 1.2133878537

H 6.166414734 -19.2622085839 0.3300742812
H 5.6698641071 -18.729477162 1.9587218818
H 6.6560197528 -17.6539933441 0.906028923
N 15.5507273281 -21.1801641832 -0.2266714354
C 15.4347327764 -20.0230888445 -0.1241330134
C 15.2639203862 -18.5941615939 -0.0096994656
H 14.3311050232 -18.2993629217 -0.5188409483
H 15.183794605 -18.3051782061 1.0432604882
H 16.1137573996 -18.080604818 -0.4712108991
N 15.1081582981 -23.1663018163 -2.976705318
C 16.1931937646 -22.8454626021 -2.6918167997
C 17.5306902386 -22.4248109427 -2.3416066843
H 17.7398428022 -22.6791913162 -1.298684586
H 18.2689935676 -22.9059082841 -2.9904182498
H 17.606284594 -21.3370015933 -2.4491939767
N 12.1920530449 -22.1198729727 -4.9551068701
C 12.1506506472 -23.2000965867 -4.5188772995
C 12.1193295407 -24.5356018581 -3.9671418129
H 13.1098158135 -24.776762656 -3.5666040513
H 11.3819636931 -24.5941474006 -3.1600400271
H 11.8571924026 -25.2667364921 -4.7379499589
N 13.9517498982 -19.061980401 -5.5740815283
C 13.0564995526 -19.3312743207 -6.271000788
C 11.947798124 -19.6766295725 -7.128591811
H 12.2235658732 -20.5088032031 -7.7835703277
H 11.0959234579 -19.9833462093 -6.5122908123
H 11.6531565258 -18.8164706967 -7.7377675743
N 16.3004422103 -19.0779159184 -3.0496144729
C 16.2841204473 -18.121643724 -3.7180027789
C 16.2409867529 -16.9420213231 -4.5504621394
H 16.1876849423 -16.0357208365 -3.9338049595
H 17.129542139 -16.8877769543 -5.1872313545
H 15.3473312976 -16.9968005598 -5.183011561

TS_A-B_ortho_2Kcation_MicroSolvation_acetonitrile_conf7
C 12.5871536749 -14.7985107141 -1.2923959293
C 13.7639129063 -16.4292049759 -2.1272853351
C 12.8937516411 -16.6420946376 -4.3893481848

C 10.2617373011 -13.8175526971 -4.0943443424
C 9.1099645776 -14.4172080229 -3.6465969294
C 9.0555479679 -15.1380721964 -2.4518207693
C 10.1486739653 -15.2628359879 -1.6459271498
C 11.3784032947 -14.6830180648 -2.0575371702
C 11.4434095767 -13.9369167768 -3.2988242035
C 7.0909221679 -15.3172994367 -3.452149969
H 10.2470969148 -13.1899956444 -4.9834589397
H 10.0643770949 -15.7845282576 -0.701007691
H 6.109856737 -14.8794156614 -3.2435278599
O 7.8691008003 -14.3818988183 -4.2108254919
O 7.7686661554 -15.5702667628 -2.2385786644
H 6.9946139076 -16.2499727945 -4.0346704371
N 12.6350285708 -13.3521822795 -3.6154692193
S 12.8785072515 -12.7376423677 -5.0652816095
O 12.3349605485 -11.3700208624 -5.2441591045
O 12.5323521799 -13.6657740526 -6.1848481302
C 12.1776189812 -16.1060898324 2.2720644244
C 13.1071940483 -15.3062966984 2.9312562512
C 11.9885954424 -15.988930417 0.8987690495
H 13.24432701 -15.3980311609 4.0070252576
H 11.300476973 -16.6718727205 0.4123108995
C 13.8699415662 -14.3963004038 2.1994647846
C 12.7183697442 -15.0490089038 0.1460229217
H 14.6069219608 -13.7707222709 2.6996537847
C 13.6836361782 -14.2818579526 0.8287705494
H 14.2760756613 -13.5642445391 0.2605611705
H 11.5973288853 -16.8395078453 2.8283513169
C 15.4142299926 -13.6255656927 -5.6473651917
C 16.8009940718 -13.5104430514 -5.6699691211
C 14.6513843215 -12.606701576 -5.0821668539
H 17.4027583985 -14.3003017353 -6.1217143623
C 17.4427103741 -12.3900357307 -5.1324053292
C 15.2663296328 -11.4778525048 -4.5453434269
H 14.6548844859 -10.6815952714 -4.1266456886
C 16.6515028087 -11.3797333577 -4.5700415185
H 17.133880779 -10.4961869954 -4.1529306503
H 14.9132521516 -14.4968956539 -6.0705653988

C 18.934216905 -12.2593177544 -5.1680244398
H 19.4135412774 -13.1632362544 -5.5575942694
H 19.2472944651 -11.4206452949 -5.8025071901
H 19.3458809907 -12.0657512335 -4.1702302451
H 13.3479730888 -14.1093345148 -1.6582414975
H 12.9389840679 -16.3739075067 -5.445100578
C 13.8051860079 -16.1174926314 -3.5174580565
C 12.9765003007 -17.5607668915 -1.6830786944
C 11.9105653866 -18.0080516494 -2.5870897146
C 11.8929477497 -17.56363844 -3.9376271151
C 10.8996798682 -18.0897985192 -4.7896370671
C 9.9388636539 -18.9713346543 -4.3210916221
C 9.9460139982 -19.3809888693 -2.980936321
C 10.9397054626 -18.9145956134 -2.1358745098
H 14.5447820773 -15.3981888266 -3.8682151719
H 14.6309550642 -16.1905227567 -1.5107798282
O 13.1683386989 -18.1307542227 -0.5815760884
H 10.9811095013 -19.2338365251 -1.094229014
H 9.1925436196 -20.072945637 -2.6116566583
H 9.1898251882 -19.361296175 -5.010586368
H 10.9223928401 -17.8613385451 -5.8557317374
K 10.6786719789 -15.4384091543 -6.7527200017
K 13.8536870597 -20.6011345311 -1.6195726276
N 16.0181057626 -19.8340565152 0.1782785736
C 15.8074377511 -18.8162860394 0.7072325277
C 15.5192188861 -17.5596105221 1.3599872545
H 14.5294271491 -17.2226276652 1.0261688881
H 15.5160707523 -17.6786689497 2.4480452423
H 16.2652906415 -16.8037194038 1.0932223833
N 15.5821126721 -22.8380883201 -2.2907360998
C 16.6317887542 -22.3677201621 -2.099898388
C 17.917671953 -21.7583664227 -1.847590237
H 17.7884702383 -20.9629020934 -1.1049664295
H 18.6268511236 -22.4985528445 -1.4648380019
H 18.3189060396 -21.3228159376 -2.7676651528
N 11.8631152877 -22.6324055854 -1.4538111493
C 12.2065781768 -23.4434393281 -2.2177583964
C 12.6616636956 -24.4313374705 -3.1696706764

H 13.751386008 -24.3605113968 -3.2569865188
H 12.3913299929 -25.4394445974 -2.8411936377
H 12.2149945903 -24.2459942983 -4.1513569129
N 13.1047954808 -20.8999977111 -4.4317998868
C 12.8322200485 -20.5461882694 -5.5081447303
C 12.4956459647 -20.1108390153 -6.8477824662
H 12.9981932365 -20.7370550688 -7.592259156
H 11.4144353688 -20.1722523385 -7.0110118212
H 12.7931725006 -19.0674149646 -7.0107547274
N 15.7088032579 -19.1714841729 -3.2904858344
C 15.8006565535 -18.5730818237 -4.2857486344
C 15.9262488396 -17.8384042305 -5.5266590227
H 16.2503232481 -16.8114029171 -5.3243122003
H 16.6601867163 -18.3163722725 -6.1839008193
H 14.9610144454 -17.7894249238 -6.0439216855
N 8.1373120148 -14.5158118556 -7.5052624551
C 7.227658684 -13.8583686954 -7.1917303356
C 6.1112652761 -13.0370310877 -6.7871246955
H 5.4236282111 -13.6134558521 -6.1600010591
H 6.4953491624 -12.1850716618 -6.2134731206
H 5.5642860446 -12.6693658134 -7.6606937845
N 11.000814951 -13.8620135477 -9.2064034524
C 10.8021051759 -12.831004224 -8.6954866483
C 10.5505434975 -11.5755904706 -8.0291244552
H 10.6224443645 -10.7427445785 -8.7358839749
H 9.5474678483 -11.574288684 -7.5846606707
H 11.2752032693 -11.4254357491 -7.2159983083
N 8.3979239574 -11.1365636266 -5.4064233461
C 9.0480941186 -10.842558389 -4.4843440949
C 9.8754643447 -10.5018782918 -3.3487428628
H 9.6394255154 -11.1526536068 -2.4998964377
H 10.9285636094 -10.6442956343 -3.6219962206
H 9.7141829156 -9.4607305927 -3.0520607653
N 13.2823469386 -16.5599562097 -7.8392404415
C 13.6810784354 -15.5798235155 -8.3322044668
C 14.1728865168 -14.3544150108 -8.9203415619
H 15.2618618236 -14.3891028168 -9.0294106455
H 13.7138112934 -14.1947286917 -9.9002127064

H 13.9012427964 -13.5203874257 -8.2631983827
N 9.897921742 -17.746788119 -8.2693960603
C 9.499552713 -17.1925009891 -9.2153670947
C 9.0134592081 -16.4763115242 -10.3730589316
H 9.1821506841 -17.0570180419 -11.2849894552
H 7.942561474 -16.2771553501 -10.2697035197
H 9.5413623264 -15.5188051579 -10.4515232535

TS_A-B_ortho_2Kcation_MicroSolvation_acetonitrile_conf8
C 12.7884349127 -14.9949347917 -1.0593547673
C 14.0378942979 -16.2024537366 -2.4057929203
C 13.5678213298 -15.5092209158 -4.6966114453
C 15.717145505 -12.9731177883 0.1611038439
C 15.3930659341 -11.7553388793 -0.3795904621
C 14.2093905294 -11.5428872513 -1.0834796314
C 13.3129564527 -12.5513115926 -1.3039774849
C 13.6076962131 -13.8469930819 -0.7973864634
C 14.8058938224 -14.0492496201 -0.0156621033
C 15.323282149 -9.6084889633 -0.9816237898
H 16.6054605025 -13.1126788496 0.7650122179
H 12.376711301 -12.3557345244 -1.8160965226
H 15.0308622319 -8.8428889485 -0.2495980416
O 16.1142604251 -10.6034174288 -0.3242616714
O 14.1628875183 -10.235104229 -1.5135792317
H 15.9101638101 -9.1662877652 -1.7964905423
N 14.9868243324 -15.2895038054 0.5232938255
S 16.4209231275 -15.824645891 0.9244367953
O 17.2226671372 -15.0422489641 1.9028980818
O 17.2224176186 -16.1697856917 -0.301778394
C 9.8861230266 -14.3570163087 -3.4276816393
C 8.9336784214 -15.2186850078 -2.8912686107
C 11.1420286734 -14.2450351639 -2.8420622745
H 7.9502828845 -15.3005731035 -3.3500891879
H 11.889696669 -13.6081889195 -3.3074293296
C 9.252992737 -15.9878046378 -1.772471776
C 11.4790755197 -15.0017797529 -1.7048954615
H 8.5200861812 -16.6743376074 -1.3532356271
C 10.511180933 -15.889630283 -1.1967531051

H 10.769632541 -16.5093793251 -0.3387028749
H 9.6536102998 -13.7737729882 -4.3170748078
C 16.6566158219 -17.7226880031 2.8749300876
C 16.3906363092 -18.9642781344 3.4472500296
C 15.9906844626 -17.3549806951 1.709700587
H 16.9027174221 -19.2545783204 4.3644301483
C 15.4635621079 -19.8409429581 2.8737233718
C 15.064904915 -18.2094918697 1.1094151318
H 14.5434664634 -17.9252863541 0.1927719541
C 14.8081792958 -19.4387984008 1.7018039327
H 14.0822370461 -20.1080833987 1.2383238454
H 17.3618201284 -17.0308028254 3.3304310002
C 15.1592862796 -21.1665686226 3.5008162396
H 15.8871459783 -21.4282813396 4.275722247
H 14.1679523388 -21.1639344834 3.9720206085
H 15.1503806416 -21.9725355802 2.7572006507
H 12.9190878228 -15.7968709596 -0.33148951
H 13.75766175 -14.8597348242 -5.5526377852
C 14.2801135802 -15.373100391 -3.5407561814
C 13.1899937879 -17.3687475981 -2.5348835439
C 12.351985131 -17.4374503073 -3.7429217231
C 12.5534549353 -16.5207805632 -4.8116387771
C 11.7284450899 -16.6205751012 -5.9477276346
C 10.7301688857 -17.5794701185 -6.0231959957
C 10.5346327685 -18.4778029102 -4.9662872124
C 11.342981923 -18.4033420525 -3.8421921805
H 15.0327896952 -14.5853722908 -3.4599291267
H 14.7843149165 -16.2504754998 -1.6121211507
O 13.1370446938 -18.2536988036 -1.6475553804
H 11.1825502896 -19.0717067145 -2.9960730865
H 9.7442577207 -19.2255904628 -5.0220786162
H 10.0953654699 -17.6313110842 -6.9067224019
H 11.8760127436 -15.9191307441 -6.7695541015
K 17.9982533676 -14.3567574677 -2.1996103759
K 13.4961806309 -20.8613205896 -2.3603941361
N 19.5400048967 -13.1852363814 -0.0205730255
C 19.1477262072 -12.7420282477 0.9842661926
C 18.6379988794 -12.2077261299 2.2267741539

H 18.0314652721 -11.3166579296 2.0317537556
H 18.0123245577 -12.9683805564 2.7078613904
H 19.4587824797 -11.9330596149 2.8966175076
N 20.29313682 -13.1994516508 -3.5854825072
C 21.1935781206 -13.4460139232 -2.8877596753
C 22.2988947374 -13.7551181328 -2.0096240265
H 21.9861602475 -13.5932939337 -0.9723898111
H 22.5908206618 -14.8027232747 -2.1308182077
H 23.1582066585 -13.1142078802 -2.2294301447
N 20.1378059124 -16.3277564472 -1.6479435626
C 20.1602950663 -16.327174602 -0.4811197632
C 20.1862725952 -16.3140936774 0.9645068029
H 19.291999408 -15.800594886 1.3437561052
H 20.2023054561 -17.336350809 1.3586630607
H 21.0792075151 -15.7909848906 1.3236415706
N 17.6108841021 -15.0185052134 -5.0070912826
C 18.0507593976 -14.1513770837 -5.6489908921
C 18.6009553071 -13.058686623 -6.4168681917
H 19.2833786687 -12.4922095566 -5.7744057713
H 19.154247391 -13.434951981 -7.2826690406
H 17.8041079955 -12.3958604111 -6.7683993193
N 16.5498686575 -12.2120275978 -3.5007734492
C 15.4955882583 -11.9791981128 -3.9409247407
C 14.1796506848 -11.715978418 -4.4796560129
H 13.6913123743 -10.9254526855 -3.8996109958
H 14.2416273803 -11.4052222993 -5.5275950929
H 13.5746320427 -12.6299737486 -4.4152390621
N 16.1320955591 -20.66105522 -1.2887237591
C 16.9880922469 -20.1228439836 -0.7099472976
C 18.0384188759 -19.4313267412 0.0023723692
H 17.9544663652 -18.3489894377 -0.1721723189
H 17.9375299113 -19.6120577625 1.079843964
H 19.0230894818 -19.7772522551 -0.3312090082
N 13.7960681126 -23.6840529779 -2.0488175004
C 13.9926560953 -24.8310480811 -2.0641887254
C 14.2383897248 -26.2567232915 -2.0838794577
H 13.5416403088 -26.7750381111 -1.4179345861
H 14.1107823715 -26.6524642316 -3.096146657

H 15.2590526395 -26.4730916831 -1.7537577299
N 10.7107852975 -21.421716347 -1.9463785118
C 10.1435393314 -22.0117525796 -2.775983872
C 9.4576421616 -22.7432788415 -3.8172048766
H 9.18778147 -23.7456152348 -3.4708045402
H 8.543126608 -22.2222198715 -4.1174890524
H 10.1225189509 -22.8297411086 -4.6831791725
N 12.3797978346 -21.6524901946 -5.0041881744
C 12.7762238525 -20.8561654466 -5.7586256725
C 13.2663540174 -19.8678587736 -6.6916687585
H 14.2703634223 -20.1375434023 -7.0323388065
H 12.5970775367 -19.7908368936 -7.5544384011
H 13.3237007987 -18.8934417896 -6.1943300353
N 15.1375355574 -19.3540980636 -4.2414273875
C 15.976842428 -18.6864145583 -3.7838717894
C 17.0170976921 -17.8586553877 -3.2189389236
H 16.7311499591 -17.5137384043 -2.2151346669
H 17.9557606435 -18.41905534 -3.141773928
H 17.1880685439 -16.9858520027 -3.862543201

TS_A-B_ortho_2Kcation_MicroSolvation_acetonitrile_conf9
C 12.1157969794 -15.581908909 -1.6224992406
C 13.7700419999 -17.0487413762 -1.7545023765
C 14.4501590051 -16.964350349 -4.0825555545
C 13.2195444005 -13.7679969278 1.487553013
C 13.5104151429 -12.5420431954 0.9387103793
C 13.3658519477 -12.2736844971 -0.4237597682
C 12.9309727969 -13.2313458598 -1.2971291643
C 12.6273804599 -14.5267543188 -0.7927302387
C 12.7640518076 -14.7994391363 0.621845226
C 14.1228979591 -10.4292817843 0.5716607255
H 13.3514142226 -13.9372809726 2.5517869482
H 12.7720399965 -12.9870186584 -2.3413466167
H 13.5308900293 -9.5424546543 0.8276479612
O 13.9351629685 -11.4245828077 1.5833707203
O 13.6787578496 -10.9616039239 -0.6676258446
H 15.1915106653 -10.1788604894 0.5101846753
N 12.4100005785 -16.0610357245 1.0304152358

S 12.823561677 -16.6212049494 2.4636241533
O 14.2443701378 -16.4215908166 2.8141752132
O 12.3482303166 -18.0241574599 2.4926895703
C 11.7833445152 -14.7312646127 -5.3160971044
C 10.6289538406 -15.4442742619 -5.6261080113
C 12.2960238712 -14.7438519543 -4.0219048774
H 10.2394596404 -15.4374089659 -6.6423284908
H 13.2348586443 -14.2346896048 -3.8209590659
C 9.9872097587 -16.1835899335 -4.6311161963
C 11.6484694591 -15.4620472719 -2.9984287528
H 9.1018598934 -16.7730813048 -4.8662379768
C 10.4953298521 -16.1942009863 -3.3421150933
H 10.0174799397 -16.8014174259 -2.5742623053
H 12.3039006667 -14.1735355379 -6.0924203388
C 12.4935941574 -15.4058222128 4.9282440031
C 11.7390769513 -14.7957961445 5.9265421085
C 11.8797144806 -15.7748147898 3.7316733746
H 12.2201924628 -14.5022804263 6.8593729587
C 10.37282967 -14.5430463927 5.7512255061
C 10.5239047419 -15.5224757463 3.5295947484
H 10.0569735501 -15.8134502918 2.5885072804
C 9.7827749853 -14.9115512166 4.5354777071
H 8.7222383958 -14.7092421927 4.3777088035
H 13.5588242988 -15.5895264435 5.0575024
C 9.5631297408 -13.9151997592 6.8438578998
H 10.1161677094 -13.118479872 7.3532207766
H 8.6284412335 -13.4884120259 6.4656569239
H 9.2943841919 -14.6507331102 7.6146406778
H 11.5753717953 -16.3342562573 -1.046772763
H 15.0559197245 -16.5422006119 -4.8840823561
C 14.6139478529 -16.5577994452 -2.7904330799
C 12.9274628019 -18.2060292688 -1.9872076997
C 12.7140222633 -18.5820856975 -3.3857251645
C 13.4614657439 -17.9498535624 -4.417710815
C 13.2140381052 -18.3280514786 -5.7511614401
C 12.2682585952 -19.2941556479 -6.0535999536
C 11.5436137583 -19.9221585276 -5.0294243
C 11.771298467 -19.5686589645 -3.7085437101

H 15.3530373586 -15.792766355 -2.5487052799
H 14.0282017355 -16.8514903323 -0.7147049402
O 12.3679393022 -18.8246733913 -1.0459936724
H 11.2052238227 -20.0217423409 -2.8958120608
H 10.7972726647 -20.6756744213 -5.2742087624
H 12.087402648 -19.5670470038 -7.0920378849
H 13.7767085007 -17.8427612041 -6.549209115
K 10.2842514111 -18.8778799885 0.6207505423
K 14.379999947 -19.261333313 0.8557309269
N 8.8602469006 -16.1994536261 0.3035407725
C 9.1456523495 -15.08748014 0.0953823008
C 9.4893599946 -13.7031020591 -0.1430767428
H 10.2688713777 -13.3845203827 0.5591717796
H 9.8776471276 -13.5777482219 -1.1597100433
H 8.6096706352 -13.0637601109 -0.0160070288
N 6.8532817194 -19.2661603704 0.7161384535
C 6.6871345089 -18.4390788632 1.5201307828
C 6.4720827833 -17.4015081815 2.5044250878
H 7.371495428 -16.782858899 2.582927414
H 5.6236151296 -16.7711971956 2.2178360612
H 6.2678169117 -17.8445100347 3.4838265921
N 8.6514690188 -18.7251267459 -2.1351232064
C 7.6367994286 -18.1585496492 -2.0411110916
C 6.3870113245 -17.440746246 -1.923034812
H 6.4795618618 -16.6777231664 -1.1425631929
H 6.1290240996 -16.9526431042 -2.8684723694
H 5.5804233209 -18.1253101792 -1.6415723417
N 9.6049311089 -21.4798890216 -0.5818142507
C 8.5287246084 -21.5969622427 -1.0127267217
C 7.1907561368 -21.7215054067 -1.5441079548
H 6.5204974745 -21.0819836685 -0.9595783701
H 7.17102637 -21.3993278635 -2.590086776
H 6.8432359682 -22.7575035381 -1.4848362091
N 9.2139449757 -18.6641981758 3.3169549421
C 9.5870638516 -18.4681470892 4.402768066
C 10.0502699995 -18.2090870376 5.7476911922
H 10.9014349285 -17.5175296318 5.7207012595
H 9.2559659485 -17.7496945997 6.3455839818

H 10.3584729412 -19.1394682877 6.2355577305
N 15.2967432945 -20.2211676278 -1.684744055
C 15.2904414529 -20.4966831912 -2.8164408547
C 15.265459141 -20.8365222805 -4.2219689303
H 14.228438914 -20.8831039273 -4.5770051482
H 15.7901750209 -20.0735333209 -4.8058243264
H 15.7457554008 -21.8050133763 -4.393604968
N 15.5292159807 -22.166485805 1.0025842248
C 16.5725146712 -21.682111601 1.1928010131
C 17.8436901262 -21.0348620685 1.4277060124
H 18.6480116412 -21.7726977944 1.5060636114
H 18.0675128061 -20.344921526 0.6070963604
H 17.7849193505 -20.4587925325 2.3572442793
N 16.7120371727 -17.665617733 0.4946298081
C 16.5951642102 -16.52045684 0.6783655645
C 16.4355339474 -15.1017256007 0.904458025
H 17.4099878123 -14.6053309244 0.9480762667
H 15.844504166 -14.6519510716 0.0976434981
H 15.8975764033 -14.9493214647 1.8458239289
N 15.4974441662 -19.8121725658 3.4796104569
C 14.500111331 -19.843271561 4.0821233398
C 13.2537461244 -19.8819363438 4.8129223148
H 13.3184125409 -20.5947184305 5.6414656299
H 13.0291972096 -18.8849900705 5.2064738186
H 12.4507775011 -20.1793054111 4.1287995357
N 12.1389695243 -21.1051397846 1.7353401153
C 12.3274728767 -21.7049320581 0.7520126153
C 12.5999019453 -22.4059661913 -0.4807393484
H 11.8764683283 -23.213033796 -0.6252376106
H 12.5091138192 -21.7014358075 -1.3156548005
H 13.6187407996 -22.8058644488 -0.445404127

TS_A-B_ortho_2Kcation_MicroSolvation_acetonitrile_conf10

C 11.796165 -15.774582 -1.476997
C 13.552858 -17.051711 -1.942595
C 14.090429 -17.029525 -4.321527
C 9.321652 -18.430598 -0.481817
C 9.334828 -18.22591 0.869795

C 10.090478 -17.220043 1.480818
C 10.880132 -16.387406 0.749781
C 10.946513 -16.569346 -0.663217
C 10.150316 -17.6076 -1.300007
C 9.093744 -18.410492 3.073606
H 8.736962 -19.234991 -0.908649
H 11.420621 -15.575217 1.221885
H 8.229952 -18.174107 3.703639
O 8.632915 -18.90705 1.816386
O 9.843087 -17.224971 2.832552
H 9.740712 -19.168946 3.538597
N 10.247075 -17.68834 -2.654275
S 9.52803 -18.876097 -3.490856
O 9.536825 -20.199902 -2.84594
O 10.090438 -18.763096 -4.848611
C 13.988907 -13.379449 0.504739
C 13.890377 -12.227963 -0.277192
C 13.314919 -14.538857 0.136149
H 14.418685 -11.321104 0.011273
H 13.449316 -15.439887 0.734211
C 13.118077 -12.254937 -1.437185
C 12.511316 -14.575051 -1.018051
H 13.041248 -11.367051 -2.063041
C 12.440788 -13.413706 -1.804297
H 11.846539 -13.432346 -2.716936
H 14.611807 -13.386103 1.399556
C 6.827985 -18.955087 -2.84262
C 5.511048 -18.515356 -2.94707
C 7.808203 -18.404077 -3.666381
H 4.747319 -18.953615 -2.305125
C 5.14584 -17.531326 -3.870962
C 7.459348 -17.434207 -4.609706
H 8.215893 -17.027531 -5.279424
C 6.142475 -17.003733 -4.703517
H 5.877455 -16.249191 -5.444217
H 7.092423 -19.749604 -2.148208
C 3.722963 -17.081969 -3.999344
H 3.145109 -17.294652 -3.094016

H 3.651355 -16.007841 -4.204313
H 3.219812 -17.594639 -4.82972
H 11.504939 -15.737923 -2.524005
H 14.476398 -16.527287 -5.209846
C 14.123485 -16.431712 -3.092742
C 13.05047 -18.409021 -2.018207
C 13.053306 -19.049695 -3.341422
C 13.561643 -18.355979 -4.475176
C 13.554292 -19.004438 -5.728215
C 13.100478 -20.306814 -5.848135
C 12.648349 -21.004587 -4.717779
C 12.629724 -20.378065 -3.482659
H 14.537845 -15.426007 -2.98448
H 13.910117 -16.747552 -0.961869
O 12.654664 -19.007553 -0.988961
H 12.270099 -20.896199 -2.596892
H 12.300565 -22.03208 -4.812884
H 13.10624 -20.79324 -6.823265
H 13.920201 -18.463145 -6.601797
K 11.187937 -16.348422 -5.435236
K 14.345008 -18.891719 1.143414
N 11.762894 -20.132234 2.149624
C 11.309817 -20.675254 1.22095
C 10.743517 -21.338978 0.069268
H 10.85226 -20.69744 -0.813383
H 11.25249 -22.2916 -0.111291
H 9.678412 -21.536246 0.231766
N 15.039026 -20.353846 3.499312
C 14.193181 -20.242919 4.292865
C 13.126291 -20.093824 5.256654
H 12.194276 -19.894769 4.716835
H 13.009097 -21.006411 5.849038
H 13.335832 -19.259814 5.933673
N 15.845679 -19.677813 -1.180226
C 16.034996 -19.958358 -2.294736
C 16.263786 -20.310703 -3.678931
H 15.365344 -20.778163 -4.100418
H 16.486176 -19.414655 -4.267157

H 17.103369 -21.0079 -3.76391
N 16.017427 -16.602729 0.635314
C 16.440611 -15.811235 -0.106536
C 16.941988 -14.823054 -1.036097
H 17.834262 -14.332353 -0.634733
H 17.20258 -15.297897 -1.987733
H 16.173636 -14.061013 -1.216326
N 13.366614 -17.025204 3.089232
C 12.771305 -16.175414 3.61823
C 12.013292 -15.115578 4.246481
H 10.946651 -15.360597 4.216307
H 12.320086 -14.981599 5.288377
H 12.171643 -14.170081 3.716224
N 9.349012 -14.585283 -3.792964
C 8.680794 -14.905169 -2.892332
C 7.847725 -15.302308 -1.778915
H 7.591721 -16.365413 -1.866381
H 8.381716 -15.15458 -0.833551
H 6.921861 -14.717944 -1.76482
N 9.946605 -14.442763 -7.278291
C 9.822453 -13.454245 -6.672723
C 9.683876 -12.233356 -5.911951
H 8.700559 -12.193632 -5.434707
H 10.450269 -12.216544 -5.13039
H 9.803263 -11.359318 -6.559806
N 12.577601 -13.753232 -5.091675
C 13.678346 -13.689197 -5.469535
C 15.042393 -13.635233 -5.944743
H 15.271003 -12.647533 -6.35691
H 15.7427 -13.842657 -5.128433
H 15.17262 -14.388783 -6.729046
N 13.341437 -15.902671 -7.598454
C 12.663439 -15.870384 -8.547304
C 11.818667 -15.81842 -9.717796
H 10.814029 -15.509234 -9.41229
H 12.217352 -15.106428 -10.447349
H 11.750491 -16.808949 -10.175565
N 10.53623 -17.986793 -7.890964

C 10.042275 -19.007709 -7.62151
C 9.432621 -20.274634 -7.284431
H 8.449771 -20.106579 -6.832967
H 9.317273 -20.899417 -8.175716
H 10.05192 -20.790483 -6.544115

TS_A-B_ortho_2Kcation_MicroSolvation_acetonitrile_conf11
C -1.141318 0.184785 0.778701
C -1.770621 0.194815 -1.335668
C -3.979033 1.199577 -1.355522
C 2.312516 1.590528 0.239825
C 2.161303 2.794643 0.884923
C 0.972678 3.171367 1.510198
C -0.127118 2.358718 1.49382
C -0.039971 1.104275 0.829461
C 1.20689 0.697683 0.21052
C 2.443696 4.826634 1.76125
H 3.243318 1.346151 -0.258327
H -1.031172 2.646373 2.018559
H 3.013191 5.041456 2.673843
O 3.092773 3.771271 1.043206
O 1.13385 4.397608 2.10206
H 2.390516 5.716555 1.118413
N 1.212943 -0.541656 -0.38149
S 2.511617 -1.184259 -1.060981
O 3.238523 -0.293834 -1.996977
O 2.051959 -2.482027 -1.607139
C -4.399651 1.376133 2.333787
C -4.883207 0.173206 2.838994
C -3.178661 1.422372 1.666635
H -5.842603 0.142179 3.351702
H -2.852972 2.360313 1.224159
C -4.13972 -0.995879 2.667269
C -2.401486 0.260351 1.505704
H -4.52018 -1.949268 3.031857
C -2.923906 -0.949481 2.004691
H -2.354351 -1.865833 1.848295
H -4.985488 2.287057 2.441632

C 4.764873 -0.748757 0.492154
C 5.59237 -1.00712 1.582803
C 3.676634 -1.582866 0.238428
H 6.445387 -0.356217 1.772599
C 5.353108 -2.08864 2.435617
C 3.442579 -2.687838 1.061223
H 2.637516 -3.38511 0.844988
C 4.270029 -2.931586 2.148026
H 4.064471 -3.794324 2.780861
H 4.978334 0.079581 -0.179449
C 6.229106 -2.351636 3.621199
H 7.048298 -1.629167 3.691776
H 5.660107 -2.303016 4.558678
H 6.670828 -3.354592 3.579439
H -0.808878 -0.835021 0.605495
H -4.629619 2.071892 -1.410692
C -2.625549 1.324104 -1.481063
C -2.321696 -1.142395 -1.32648
C -3.765582 -1.254938 -1.132467
C -4.578212 -0.086947 -1.138704
C -5.963474 -0.236175 -0.939492
C -6.521769 -1.487505 -0.737897
C -5.714963 -2.634692 -0.737332
C -4.350119 -2.515414 -0.937785
H -2.180693 2.311235 -1.619508
H -0.725622 0.245078 -1.625098
O -1.587613 -2.161833 -1.416434
H -3.696281 -3.387001 -0.941893
H -6.16216 -3.613369 -0.574638
H -7.595051 -1.58036 -0.580777
H -6.594891 0.65239 -0.939771
K 0.004497 -3.4671 0.317601
K 0.012936 -3.182281 -3.378199
N -1.206351 -5.333292 -1.426947
C -1.873743 -6.101448 -1.99251
C -2.705311 -7.041595 -2.707687
H -3.303612 -6.490546 -3.441276
H -2.084552 -7.780805 -3.224568

H -3.375951 -7.565466 -2.019534
N 1.404961 -5.082781 2.711265
C 0.876184 -6.019643 2.25937
C 0.199353 -7.153252 1.669508
H -0.882326 -6.978221 1.671649
H 0.536096 -7.266143 0.633485
H 0.416427 -8.070291 2.225932
N 1.842944 -5.563352 -0.681234
C 2.475996 -5.431853 -1.650995
C 3.252641 -5.237213 -2.852896
H 4.074205 -5.95865 -2.902799
H 2.617585 -5.359426 -3.737778
H 3.658941 -4.22095 -2.853669
N 0.349225 -1.902022 2.727225
C 1.18605 -1.134574 2.990915
C 2.219259 -0.177617 3.316398
H 2.696112 -0.434977 4.268202
H 2.984621 -0.17197 2.530265
H 1.790388 0.828471 3.388882
N -2.055759 -4.546781 2.114611
C -1.81919 -4.174981 3.194843
C -1.513999 -3.705202 4.526915
H -2.092324 -4.25701 5.274436
H -0.444761 -3.838627 4.720303
H -1.747021 -2.638179 4.604872
N -3.127433 -4.065957 -4.014293
C -3.282178 -2.927762 -4.214053
C -3.453701 -1.517476 -4.486339
H -4.320661 -1.125087 -3.943891
H -2.564199 -0.958311 -4.169889
H -3.594051 -1.36083 -5.560545
N -0.951926 -2.878011 -6.309354
C -0.214687 -2.134824 -6.8219
C 0.695354 -1.206169 -7.453225
H 0.241088 -0.76462 -8.345678
H 0.93914 -0.409059 -6.742856
H 1.618298 -1.719453 -7.74033
N 0.278452 -0.325846 -4.198267

C 0.674933 0.646422 -3.690121
C 1.160851 1.848374 -3.051689
H 0.512136 2.116908 -2.208702
H 2.16498 1.661521 -2.655215
H 1.183009 2.683628 -3.758883
N 2.387347 -2.806598 -5.055264
C 3.146234 -1.922158 -5.023263
C 4.076452 -0.816901 -4.979999
H 5.059654 -1.12324 -5.35063
H 3.713742 0.00983 -5.601286
H 4.164356 -0.469859 -3.943583
N 0.154734 -5.820034 -4.59355
C -0.661002 -5.897508 -5.423395
C -1.687137 -5.99685 -6.43666
H -2.52593 -5.350329 -6.158407
H -1.299337 -5.66317 -7.403343
H -2.036845 -7.030208 -6.530343

TS_A-B_ortho_2Kcation_MicroSolvation_acetonitrile_conf12
C -1.7208422157 0.1314954292 -0.0883859417
C -2.0501554261 0.3082315001 -2.3082964192
C -4.1254479278 1.5441529814 -2.5142357172
C -1.4312925635 -3.5869992934 0.438297618
C -0.0671863809 -3.6981841817 0.4166832126
C 0.7800319879 -2.5948362066 0.2433692998
C 0.2828550383 -1.3312335453 0.1142114236
C -1.1332894219 -1.1440399606 0.1171107311
C -2.0152436241 -2.2916339797 0.2972763642
C 2.0220657973 -4.4454391329 0.2775506836
H -2.0450680508 -4.4736680565 0.5547734098
H 0.9552260961 -0.4854001022 0.0287186685
H 2.6825947484 -4.8284379482 1.0617149054
O 0.6788070224 -4.8193274694 0.577555449
O 2.087610616 -3.0161019074 0.2606653738
H 2.2897078092 -4.8376115951 -0.7139387611
N -3.3510734835 -2.0331445603 0.3778742663
S -4.4140646063 -3.2420891836 0.4870079881
O -4.285164589 -4.3050771542 -0.5237328561

O -5.7363036943 -2.5790913965 0.5883850076
C 0.6597795737 3.1026885752 -0.2341362657
C -0.0859510055 4.0577250542 0.455254866
C 0.1643785012 1.8168727486 -0.42273335
H 0.306028084 5.062578624 0.5977738046
H 0.7456499191 1.1177403695 -1.0223250694
C -1.3485147178 3.7196472462 0.9361156453
C -1.0948134212 1.4465440282 0.0862310658
H -1.9561761788 4.4606176653 1.4514507644
C -1.8480720852 2.4366658025 0.7441824037
H -2.8456292623 2.1885958759 1.1075977683
H 1.6362554414 3.3633320803 -0.6412102603
C -4.276695218 -5.4304787076 2.1677356001
C -4.1624496028 -6.0433411477 3.4117389653
C -4.155865628 -4.0459043863 2.0712497489
H -4.2586128589 -7.1262701235 3.4880819841
C -3.9217470696 -5.2940693862 4.570055574
C -3.9137507477 -3.2780295424 3.2089679569
H -3.8295260633 -2.195234334 3.1266742257
C -3.8007278064 -3.9036503206 4.4451714355
H -3.6197675212 -3.3014929899 5.3362640849
H -4.4585296258 -6.0138319897 1.2671294644
C -3.7671300006 -5.9639361986 5.9007913076
H -4.3562281554 -6.8850309708 5.9647117545
H -2.7209978733 -6.2435699702 6.0830778253
H -4.0693559552 -5.3089534589 6.7250489122
H -2.7901308447 0.1447793821 0.1037554347
H -4.678468005 2.4798549114 -2.523941485
C -2.7651305621 1.5339275313 -2.4186718095
C -2.7050132396 -0.9550338854 -2.5357230302
C -4.1686494027 -0.9282617008 -2.6240333004
C -4.8592911519 0.3170557494 -2.5990780816
C -6.2669047785 0.3101603202 -2.6940848203
C -6.9629959016 -0.8751560855 -2.8444560983
C -6.2762017594 -2.0976997493 -2.8964223369
C -4.8973552794 -2.1163922797 -2.7866395512
H -2.2174797294 2.4743289618 -2.3492356417
H -0.9673142567 0.3102994136 -2.4308341969

O -2.0421257424 -2.0251803803 -2.6362995988
H -4.3580861045 -3.0606927882 -2.782142687
H -6.8247841985 -3.0318429521 -3.0097907325
H -8.0487640895 -0.8523975028 -2.9351566673
H -6.7974841948 1.2608734321 -2.6532949013
K -5.4550181764 0.0913214897 1.1286165939
K 0.6112393258 -2.2637640482 -3.0564518683
N -5.9937392989 2.6447870564 -0.1509124463
C -7.001340764 2.9903015763 0.323069506
C -8.2490694087 3.3922935163 0.9291915113
H -8.767826421 4.1282140833 0.3072967213
H -8.8833740762 2.5081720175 1.0517589813
H -8.055151191 3.81998083 1.917065327
N -6.2669546451 2.1106577878 3.1591701673
C -5.9888155289 1.6460167542 4.191008875
C -5.6374726569 1.0625706326 5.4656408987
H -5.3259735426 1.8371439353 6.1733731009
H -6.4925562802 0.5190258356 5.8771716964
H -4.8169775985 0.3521963292 5.3227147467
N -8.3027672111 -0.025616089 0.5191805639
C -8.5249550068 -1.1165512895 0.1727536012
C -8.7799632539 -2.4743797605 -0.2548795952
H -9.4327271751 -2.9856834587 0.4604957627
H -9.2685627405 -2.4840694501 -1.234508067
H -7.8233199819 -3.0059652626 -0.3225873887
N -6.6509833872 -1.3039220344 3.6391953878
C -6.9528759386 -2.4146824226 3.4555738862
C -7.3078138453 -3.7964334187 3.2201908523
H -8.3532013892 -3.9828923345 3.4866918216
H -7.1490698825 -4.0303313221 2.1618210997
H -6.6654689555 -4.4567271353 3.8152479418
N -3.2116864811 0.169743644 3.0153880271
C -2.0658389292 0.0019416652 3.1484061008
C -0.6459570381 -0.2157449841 3.3086252473
H -0.0931423972 0.4099771409 2.5979506038
H -0.3283761037 0.0354118259 4.3256834706
H -0.4026556192 -1.2648331066 3.1051436284
N 0.2285319706 -0.0963727982 -4.9134710631

C 0.6177514078 0.9416245665 -4.5529333693
C 1.0968496054 2.2182315959 -4.0733210109
H 1.3416812777 2.8810995201 -4.9087629096
H 0.3335313219 2.7000833734 -3.4519164773
H 1.992389376 2.0615345703 -3.4623966903
N 3.2021894736 -3.5945801314 -3.3225108903
C 2.867537704 -4.5014654617 -3.9755716312
C 2.4193494521 -5.6119135415 -4.7832972818
H 1.4641725886 -5.3482798649 -5.2519364638
H 3.1534631128 -5.8469454557 -5.5599585383
H 2.2626581968 -6.4907749387 -4.1510940433
N 2.5065678609 -0.2506746873 -2.116294824
C 3.4649530226 -0.7867824422 -1.7223903148
C 4.6447339038 -1.4651531624 -1.2398335348
H 5.5421439548 -0.875139331 -1.449085398
H 4.5667812905 -1.6336028924 -0.1616753666
H 4.7197542331 -2.4328260612 -1.7474374797
N 0.3489859453 -5.1516654234 -2.4196174912
C -0.8085291058 -5.2069176565 -2.5587679299
C -2.2404715103 -5.2109069696 -2.7293137634
H -2.5061233331 -5.6338293341 -3.7038590283
H -2.7328199398 -5.7783160818 -1.9346827878
H -2.5882819593 -4.1699853893 -2.6655672199
N -0.4853860105 -3.6744297366 -5.3531275957
C -1.4169055227 -2.9969574971 -5.5381502748
C -2.5653371235 -2.148865855 -5.7601003566
H -2.2707922938 -1.1014096222 -5.6327907447
H -2.9582840784 -2.2891097793 -6.7717667981
H -3.3503318351 -2.3739896743 -5.0288498917

TS_A-B_ortho_2Kcation_MicroSolvation_acetonitrile_conf13
C -0.805856 0.654171 -0.687444
C -1.380778 0.207324 -2.702681
C -3.64583 1.073777 -2.674505
C -4.077641 1.868015 0.760799
C -4.516801 0.676136 1.273962
C -3.751591 -0.493269 1.2425
C -2.522455 -0.509246 0.658454

C -2.011627 0.69556 0.09095
C -2.787603 1.913239 0.156962
C -5.74125 -0.992023 2.083693
H -4.724079 2.737471 0.789668
H -1.966631 -1.437945 0.558327
H -6.043985 -1.20361 3.114275
O -5.704561 0.421665 1.884177
O -4.440506 -1.509252 1.851722
H -6.448633 -1.433914 1.362741
N -2.201476 3.06498 -0.329448
S -3.124058 4.352887 -0.59722
O -4.392902 4.084837 -1.305756
O -2.253233 5.366463 -1.239179
C 1.616708 -1.492778 1.30502
C 2.621915 -1.739062 0.375999
C 0.488841 -0.756749 0.94732
H 3.498942 -2.323303 0.645638
H -0.265956 -0.544591 1.701793
C 2.493458 -1.214928 -0.910375
C 0.326566 -0.24068 -0.35098
H 3.287355 -1.371887 -1.642627
C 1.368769 -0.480774 -1.265321
H 1.305691 -0.068326 -2.269613
H 1.709338 -1.867286 2.323241
C -4.653362 5.968557 1.015781
C -4.983362 6.591651 2.213081
C -3.595881 5.059202 0.982134
H -5.808482 7.303157 2.238828
C -4.280875 6.314295 3.393926
C -2.886568 4.764247 2.143196
H -2.067663 4.048686 2.101294
C -3.23072 5.390613 3.337265
H -2.677442 5.15681 4.246853
H -5.214432 6.173378 0.1059
C -4.669379 6.96304 4.686808
H -4.983795 8.002687 4.542955
H -5.515048 6.43974 5.152091
H -3.848384 6.953234 5.411326

H -0.481321 1.628075 -1.053751
H -4.351445 1.900744 -2.740706
C -2.327298 1.2496 -2.961036
C -1.834631 -1.156925 -2.505429
C -3.251772 -1.322495 -2.178478
C -4.128006 -0.202684 -2.229608
C -5.464459 -0.382151 -1.826192
C -5.920625 -1.618858 -1.399103
C -5.057304 -2.724434 -1.364808
C -3.738349 -2.569306 -1.75456
H -1.948228 2.226768 -3.266342
H -0.382021 0.320653 -3.120116
O -1.045746 -2.137054 -2.53754
H -3.038215 -3.404566 -1.720178
H -5.421083 -3.690769 -1.021287
H -6.962025 -1.735269 -1.100807
H -6.137638 0.474897 -1.846801
K 0.218869 4.439761 -1.626184
K 1.287546 -3.007269 -3.416408
N 0.23285 5.744782 0.9972
C -0.540073 6.618208 1.008074
C -1.50967 7.690071 1.001169
H -2.157028 7.618672 1.883282
H -1.012384 8.664977 0.999685
H -2.135162 7.593869 0.107145
N 2.470004 5.479323 -3.103796
C 2.226977 5.959784 -4.137682
C 1.906273 6.548914 -5.417989
H 1.98251 7.63953 -5.369984
H 2.589913 6.186634 -6.191876
H 0.880747 6.272777 -5.684906
N 1.997851 2.744855 -0.02954
C 1.347678 2.663263 0.934871
C 0.511766 2.567418 2.109327
H 0.369715 3.56657 2.534634
H -0.466939 2.155706 1.826287
H 0.970691 1.912763 2.857055
N 1.138389 2.346482 -3.375773

C 2.272872 2.252364 -3.120509
C 3.670689 2.118559 -2.788463
H 4.200477 3.048373 -3.0185
H 3.762039 1.924964 -1.714565
H 4.115244 1.292592 -3.353859
N -0.895023 5.02092 -4.351171
C -2.051736 4.868855 -4.369884
C -3.482688 4.670425 -4.38653
H -3.978292 5.513885 -4.877605
H -3.732423 3.752952 -4.930379
H -3.861114 4.585192 -3.357583
N -0.447947 -5.433797 -2.578555
C -0.408243 -4.915935 -1.533345
C -0.304061 -4.219378 -0.270817
H -0.405938 -3.142335 -0.461038
H -1.093082 -4.534448 0.419894
H 0.672837 -4.410196 0.186273
N 2.484977 -4.604753 -5.47517
C 2.380077 -4.014144 -6.474892
C 2.233335 -3.266133 -7.702875
H 2.303205 -3.930265 -8.569743
H 1.253981 -2.775165 -7.703351
H 3.015567 -2.504325 -7.778806
N -0.510326 -2.702753 -5.832509
C -1.432401 -3.251996 -5.37416
C -2.563551 -3.93961 -4.797666
H -2.205069 -4.688241 -4.081198
H -3.204835 -3.229447 -4.263775
H -3.148097 -4.436919 -5.577923
N 2.115714 -0.660561 -4.784484
C 1.256671 -0.092649 -5.333216
C 0.186756 0.614596 -5.998586
H 0.463479 0.846005 -7.03224
H -0.711484 -0.01205 -6.0062
H -0.020589 1.548368 -5.462313
N 2.881947 -4.76403 -1.803436
C 2.885561 -5.788537 -2.36095
C 2.862011 -7.046731 -3.070394

H 1.868591 -7.499354 -2.986631
H 3.072851 -6.867268 -4.1294
H 3.606241 -7.737766 -2.66341

TS_A-B_ortho_2Kcation_MicroSolvation_acetonitrile_conf14
C 12.2117533686 -15.1996444724 -1.2678592978
C 13.2743949622 -17.0705165238 -1.6870693664
C 13.5868024016 -17.4744357613 -4.0749567912
C 8.9957394134 -16.5956621659 0.0982237906
C 9.1983034791 -16.3263481262 1.4238737071
C 10.330350518 -15.6538436954 1.8976286685
C 11.3154919319 -15.2438542456 1.053541259
C 11.1910416259 -15.5333823253 -0.3359896716
C 10.0084338671 -16.2151066161 -0.8276795004
C 9.0980269047 -16.2597565605 3.6433126189
H 8.1007809558 -17.1168492702 -0.2231210693
H 12.1552161948 -14.6658082621 1.4197885277
H 8.4456741783 -15.6813960775 4.3049078921
O 8.3697712572 -16.6113241128 2.4628538612
O 10.2043937549 -15.4555248487 3.2498045826
H 9.4612348016 -17.179374815 4.1234720084
N 9.9534908921 -16.4321687777 -2.1766432631
S 8.7244740219 -17.2554200237 -2.8004826075
O 8.4119693965 -18.5530231016 -2.1711702367
O 8.9755798091 -17.3069650498 -4.2611588482
C 15.332978046 -13.7782577006 0.3791129328
C 15.6700753035 -12.7683000709 -0.5221535949
C 14.2115453094 -14.5697392444 0.1577678702
H 16.5481456307 -12.1486406806 -0.3503495357
H 14.0016488115 -15.3849162681 0.8491882737
C 14.8817238565 -12.5733180757 -1.6544544134
C 13.3867783943 -14.3671983529 -0.9643196224
H 15.1439714145 -11.8006799363 -2.3750821276
C 13.76107762 -13.3678603143 -1.8764053795
H 13.1623105894 -13.2232958106 -2.7749802274
H 15.9555310468 -13.970842674 1.2529108042
C 6.0045613621 -16.892393773 -2.7214224816
C 4.840070097 -16.1349739857 -2.6778878133

C 7.2428569245 -16.2598798932 -2.6104117579
H 3.8723970253 -16.6291750491 -2.7645263794
C 4.8865450352 -14.7443368489 -2.5120415235
C 7.3150629085 -14.8788519984 -2.443451995
H 8.28621846 -14.3949932215 -2.3589017852
C 6.1407232692 -14.1334777225 -2.3949585536
H 6.1968730049 -13.0534031929 -2.2598133143
H 5.9645874384 -17.9752794232 -2.8298676171
C 3.6262530277 -13.939334114 -2.4248593485
H 2.8269502055 -14.3639638414 -3.0421095732
H 3.2480377501 -13.9090386161 -1.3944645733
H 3.7826879312 -12.9012565105 -2.7369994955
H 11.8636444208 -15.1195913089 -2.2972088339
H 14.0623507923 -17.2326227562 -5.0257571458
C 13.9225280562 -16.8069661125 -2.931472362
C 12.3035378951 -18.1429162888 -1.5820906861
C 11.9803239615 -18.8732464439 -2.8176663326
C 12.6250981992 -18.5407579234 -4.0423110192
C 12.318821081 -19.2916313787 -5.1979613462
C 11.4417595421 -20.3610516059 -5.1345787056
C 10.8418273836 -20.7130982829 -3.9150095461
C 11.1108406431 -19.9720285401 -2.776071626
H 14.6653354649 -16.0073963715 -2.9722962494
H 13.8170744888 -16.8600243085 -0.7673215329
O 11.7938971473 -18.4475935295 -0.4786386123
H 10.650694915 -20.2195007883 -1.8225983516
H 10.1593073409 -21.5607375733 -3.8664438812
H 11.2293180461 -20.9386058673 -6.0343019281
H 12.7945681868 -19.0208647874 -6.1413327097
K 11.0673792972 -16.0972765413 -5.3808987705
K 13.4058136768 -18.7376740799 1.7205321175
N 10.692966308 -19.0197949587 2.7480840799
C 9.9905452325 -19.3735713871 1.8860296099
C 9.1240930183 -19.7975414975 0.8104625952
H 8.0934902101 -19.4823571974 1.0057370998
H 9.4563952367 -19.3381886493 -0.1282898217
H 9.1436290915 -20.8877374818 0.7087512372
N 13.8802197747 -21.0863154377 3.2808940939

C 14.0377285909 -21.9402102313 4.0557227988
C 14.2334960399 -23.0005222097 5.0203279641
H 13.4600609539 -22.9606691389 5.7934760331
H 14.1850042189 -23.9779519893 4.5306260753
H 15.211127823 -22.9024232316 5.5022163342
N 14.5627417958 -20.1337054271 -0.598298666
C 14.5709271934 -20.5765224364 -1.6760313793
C 14.5749220249 -21.1245408042 -3.0154959762
H 13.5491507846 -21.347168495 -3.3340376784
H 14.9955577255 -20.3998991511 -3.7204235199
H 15.1688484517 -22.0432698828 -3.0538755778
N 15.9265897522 -17.4325076913 1.1266721602
C 16.4077791858 -17.142016953 0.1062162878
C 16.9793235581 -16.7745205398 -1.1699354089
H 18.0651473785 -16.9115775822 -1.1637553417
H 16.5469898154 -17.3958296237 -1.9628688943
H 16.7559068186 -15.7233559271 -1.3898476977
N 13.4900372544 -16.4442251218 3.4211957548
C 13.3057746379 -15.3753004905 3.8439299909
C 13.05103496 -14.0392936114 4.3354144896
H 11.9712412375 -13.8592973374 4.3576789061
H 13.4543254643 -13.9114897318 5.3446037768
H 13.5181128038 -13.2976805092 3.6779380136
N 13.1987588982 -16.6495458538 -7.3995438335
C 13.689224486 -15.6400071876 -7.7159139655
C 14.2740657769 -14.3792395044 -8.1135476572
H 14.7445381819 -13.8974269476 -7.2511528707
H 15.0234938987 -14.5286600379 -8.8969387602
H 13.4858444065 -13.7184303634 -8.4910577185
N 10.771742404 -14.3905274151 -8.2492938458
C 10.6632623498 -15.282142801 -8.9930697753
C 10.5651924305 -16.3889186753 -9.9179988378
H 10.9016531757 -16.0864888789 -10.914811687
H 11.1835900297 -17.2170608405 -9.5584564053
H 9.5326954801 -16.7433087443 -9.9783214282
N 9.5038076602 -17.7428514182 -7.3078929885
C 8.9053903021 -18.5724205208 -6.747562941
C 8.1549310793 -19.5868687401 -6.0406496355

H 7.0856219467 -19.3496184308 -6.0616732722
H 8.3037799733 -20.5703879831 -6.4979949847
H 8.4842772607 -19.6080024045 -4.9960937077
N 9.0698389447 -13.9704366593 -5.2775608069
C 8.0970183416 -14.5647201194 -5.5238938471
C 6.8994543322 -15.3183844972 -5.8188865976
H 7.080221019 -16.3692411444 -5.5672313214
H 6.0642018214 -14.9553236185 -5.2082804213
H 6.6397072012 -15.2333034852 -6.8788522863
N 12.6974560382 -13.707305047 -5.2906858867
C 12.0176152897 -12.8434187667 -5.6833696921
C 11.1779393449 -11.7833686371 -6.1907886694
H 11.7725283099 -10.8925246161 -6.4164286925
H 10.6815448761 -12.1396029723 -7.0996159619
H 10.4086866644 -11.5331753247 -5.4550953524

TS_A-B_ortho_2Kcation_MicroSolvation_acetonitrile_conf15
C 12.574931 -14.629703 -1.426867
C 13.363988 -16.511787 -0.834674
C 13.597447 -17.556624 -3.007089
C 13.172772 -14.818344 -5.141824
C 11.885154 -15.04148 -5.556387
C 10.796565 -15.052836 -4.676795
C 10.963094 -14.882344 -3.333691
C 12.281944 -14.693849 -2.835218
C 13.398503 -14.618746 -3.75236
C 10.055351 -15.559642 -6.703961
H 13.993453 -14.806261 -5.85276
H 10.108047 -14.904979 -2.665131
H 9.481255 -14.979268 -7.433149
O 11.439999 -15.233616 -6.828376
O 9.636428 -15.226061 -5.390869
H 9.926841 -16.641742 -6.87457
N 14.619591 -14.325847 -3.203837
S 15.986561 -14.74373 -3.931918
O 15.988706 -16.06097 -4.583434
O 17.03197 -14.508582 -2.897268
C 9.458098 -14.196593 0.739533

C 9.814913 -13.067302 1.472193
C 10.335515 -14.74346 -0.191907
H 9.122218 -12.641345 2.195356
H 10.040681 -15.638526 -0.733503
C 11.067861 -12.489998 1.27185
C 11.601285 -14.174529 -0.415185
H 11.361929 -11.604172 1.832715
C 11.949138 -13.044572 0.350863
H 12.923109 -12.58085 0.196773
H 8.486905 -14.662485 0.894789
C 17.136162 -13.914448 -6.288602
C 17.517203 -12.955067 -7.219603
C 16.34115 -13.53989 -5.206474
H 18.139245 -13.2453 -8.066256
C 17.114611 -11.620049 -7.09234
C 15.919554 -12.219545 -5.062203
H 15.281362 -11.937087 -4.224382
C 16.309798 -11.271778 -6.001786
H 15.987834 -10.23697 -5.88343
H 17.443228 -14.953404 -6.391851
C 17.508096 -10.598899 -8.115161
H 18.483239 -10.823855 -8.560618
H 16.783288 -10.563268 -8.939449
H 17.554605 -9.591495 -7.687612
H 13.56064 -14.204462 -1.248052
H 14.183965 -17.772927 -3.898395
C 14.172744 -16.941765 -1.9359
C 12.11516 -17.209604 -0.582804
C 11.459984 -17.760298 -1.774824
C 12.19912 -17.880781 -2.986866
C 11.526568 -18.353926 -4.131849
C 10.183037 -18.689529 -4.081837
C 9.468356 -18.594003 -2.877951
C 10.113553 -18.145432 -1.734055
H 15.227935 -16.677144 -1.960391
H 13.877143 -16.152595 0.056204
O 11.608008 -17.277016 0.562414
H 9.586525 -18.05643 -0.784105

H 8.413814 -18.860134 -2.84682
H 9.678139 -19.038079 -4.982081
H 12.081817 -18.440074 -5.067209
K 15.871184 -12.623938 -1.26515
K 13.429004 -18.625513 2.122552
N 13.55517 -11.247481 -2.223857
C 12.435139 -11.378459 -2.519583
C 11.041414 -11.526351 -2.873439
H 10.944734 -12.097664 -3.802863
H 10.513835 -12.071532 -2.081277
H 10.571903 -10.545862 -3.002565
N 15.890826 -10.550664 0.813126
C 16.700641 -11.031288 1.501105
C 17.706572 -11.657583 2.329073
H 18.152032 -10.930724 3.0151
H 17.258037 -12.468532 2.911982
H 18.488873 -12.073347 1.683718
N 15.163499 -13.954965 1.206349
C 14.329758 -14.446473 1.858082
C 13.287768 -15.077722 2.633832
H 12.626374 -15.654796 1.964993
H 13.723984 -15.741449 3.392356
H 12.675503 -14.3192 3.132855
N 18.757997 -12.676251 -0.712129
C 19.059489 -12.61057 -1.837318
C 19.401599 -12.526179 -3.238648
H 20.443936 -12.217421 -3.364512
H 19.239811 -13.499284 -3.711803
H 18.747768 -11.790845 -3.722302
N 16.980652 -10.237912 -2.545178
C 16.251431 -9.403785 -2.182175
C 15.339071 -8.386194 -1.71245
H 14.473274 -8.317568 -2.377815
H 14.993853 -8.658214 -0.709452
H 15.835736 -7.412032 -1.669984
N 12.088658 -20.330834 3.997662
C 11.497746 -21.116666 4.621001
C 10.760839 -22.093054 5.393443

H 10.464219 -21.673499 6.359665
H 9.856891 -22.398418 4.857537
H 11.373681 -22.981878 5.572372
N 15.643353 -20.559191 2.678025
C 16.102038 -20.555788 1.606448
C 16.659743 -20.536691 0.27237
H 16.575068 -21.524226 -0.191518
H 16.113031 -19.812316 -0.340654
H 17.71537 -20.249371 0.30402
N 13.401158 -20.288285 -0.165392
C 12.866022 -20.687194 -1.120161
C 12.198035 -21.187594 -2.299289
H 12.065264 -22.272148 -2.233856
H 11.215492 -20.711001 -2.401994
H 12.782667 -20.95288 -3.194342
N 15.894783 -17.423655 1.166231
C 16.670628 -16.704026 0.676049
C 17.612385 -15.799174 0.058595
H 17.677809 -14.871774 0.637832
H 18.609309 -16.249423 0.010856
H 17.302818 -15.54322 -0.966429
N 14.433456 -17.613283 4.643723
C 14.875209 -18.531018 5.209597
C 15.411731 -19.687424 5.889398
H 14.693483 -20.064862 6.623891
H 15.614492 -20.466584 5.146195
H 16.342113 -19.433842 6.406513

TS_A-B_ortho_2Kcation_MicroSolvation_acetonitrile_conf16
C 0.907416493 0.3806697672 0.7497400253
C 1.9161137731 -0.717417998 -0.8515172091
C 4.0985701566 0.3045852606 -1.1683036954
C -2.3869542362 0.9361263307 -0.9851413079
C -2.3174663055 2.2968043166 -1.1479488262
C -1.2329673181 3.0594009367 -0.7039971105
C -0.1618347926 2.4792465004 -0.0887875798
C -0.1685404213 1.0677898613 0.101942512
C -1.3026799838 0.2824933874 -0.3446764042

C -2.6858976661 4.4258055466 -1.6943628566
H -3.2349167549 0.3810469909 -1.3712073452
H 0.6522666729 3.0880808385 0.2891937737
H -3.3898216772 5.1098567948 -1.2065203976
O -3.2469217162 3.1100572804 -1.7095825068
O -1.4712707634 4.3842758543 -0.9596561054
H -2.4874460541 4.7419036364 -2.7285407937
N -1.243723217 -1.0695952529 -0.1080034934
S -2.4032709246 -2.0601012107 -0.5842303227
O -2.7594427787 -1.9974534956 -2.0188632358
O -1.9638167746 -3.3996957888 -0.1103176966
C 3.8359539117 2.5474697751 1.8499692162
C 4.1435120804 1.9479034821 3.0675880518
C 2.7818207539 2.0720206715 1.075266014
H 4.9668617146 2.325525713 3.6705935354
H 2.5916392731 2.5232032419 0.1050289108
C 3.3912041191 0.8577003741 3.5063598537
C 2.0062924462 0.9798128333 1.5056984799
H 3.6155626139 0.3869061033 4.4629635627
C 2.3461346681 0.375299629 2.7316367616
H 1.7666269593 -0.482506562 3.0734901711
H 4.4270724168 3.3882139603 1.4917288811
C -5.1130913633 -1.5339614609 -0.3729199645
C -6.2955076335 -1.3651576241 0.3430492228
C -3.9161027427 -1.7241067661 0.3136244972
H -7.2320082079 -1.2180115358 -0.1942808943
C -6.3038413931 -1.3774100934 1.7409882103
C -3.8980808606 -1.7279660667 1.7095046403
H -2.962630843 -1.854334899 2.2509216619
C -5.0845689263 -1.5589467927 2.4097316321
H -5.0662739519 -1.569779585 3.4994305678
H -5.1070845149 -1.5200965147 -1.4614421878
C -7.5735191719 -1.1775116863 2.5090572477
H -7.6540869744 -1.8766222443 3.349243551
H -8.4573447015 -1.30506978 1.8759617348
H -7.623085387 -0.1674596899 2.9361889967
H 0.6016061919 -0.5872273306 1.1399583548
H 4.740704801 1.0622843737 -1.6169308384

C 2.7741351528 0.2209436228 -1.4907632057
C 2.4701149295 -1.770014531 -0.0332889168
C 3.8761416492 -1.6378269477 0.3482362453
C 4.672986076 -0.6013306374 -0.2160645341
C 6.0279014484 -0.5178737163 0.1649492913
C 6.5780400929 -1.4264081475 1.0541150917
C 5.7895457087 -2.4529145418 1.5970444887
C 4.4520592984 -2.5494373695 1.2473727231
H 2.3335898092 0.9230526061 -2.2012639332
H 0.9387750722 -0.9169357429 -1.2821558159
O 1.7674149949 -2.7378678819 0.3651623355
H 3.8151830164 -3.3282737588 1.6690977516
H 6.228366987 -3.1725202211 2.2872048207
H 7.6280092336 -1.3447372667 1.3300000865
H 6.6435217551 0.278332455 -0.2540696901
K -0.2507582212 -3.0847827374 2.0579338403
K 0.2805919654 -3.7358723442 -1.9438970562
N 0.4534940197 -5.6708276019 0.8937714944
C 1.5686505992 -5.7299532671 0.5530343685
C 2.9433396808 -5.7659184586 0.1119757305
H 3.2580758872 -4.7324340143 -0.0756539482
H 3.5810303313 -6.1942624253 0.8916990731
H 3.0150370962 -6.3402123601 -0.8166707649
N 3.3846881503 -5.7372442211 3.3603618575
C 2.2970360649 -5.9246220588 3.7386168461
C 0.9450412779 -6.1371798805 4.2083237744
H 0.296414024 -6.4239799344 3.3738097007
H 0.9185683674 -6.9267070519 4.9659442667
H 0.550344852 -5.215577204 4.6507489333
N 1.769130102 -2.7626552843 4.0471191304
C 2.877249756 -2.7935076879 4.4054315405
C 4.2572462772 -2.8533919779 4.8285842614
H 4.3429529586 -2.6737221275 5.9048594087
H 4.6564066485 -3.8475545641 4.5965679404
H 4.8468638611 -2.1025923484 4.2907270396
N -1.1007070298 -0.6294917369 3.2762663187
C -1.1133359526 0.535582912 3.2745553268
C -1.1130146051 1.9813845749 3.2681939177

H -1.4917123023 2.3719669551 4.2180131312
H -0.0921508722 2.3510833394 3.1153907651
H -1.7392397935 2.3562047224 2.4513653148
N -2.3044591589 -4.6443141107 3.3031059167
C -2.9834322116 -5.0205441856 2.4331932214
C -3.817781118 -5.4860168233 1.3485000611
H -3.1917322695 -5.6757289808 0.4703262576
H -4.3418158566 -6.4040806449 1.6320682335
H -4.5566233985 -4.7162421855 1.0951061132
N 1.3371034521 -6.3764976369 -2.619012428
C 0.7872028485 -6.7872404085 -3.5606122663
C 0.0915892143 -7.2918239324 -4.7214664585
H 0.1718385846 -8.3818419689 -4.7774904919
H -0.9637795221 -7.0092159282 -4.6511057057
H 0.5091145026 -6.8472621047 -5.6292899375
N 0.1986248645 -4.1331753927 -5.1602161377
C -0.8135039335 -3.5529988743 -5.1809121312
C -2.0570502539 -2.8140212547 -5.1760410475
H -1.9959169477 -1.9666315589 -5.866217115
H -2.8846241898 -3.4616277025 -5.4847206031
H -2.2546883506 -2.4234366847 -4.1692344047
N -2.05016531 -5.355965882 -2.8871131174
C -3.1261766599 -5.0067460017 -2.6063257287
C -4.4646639486 -4.5828488733 -2.2627868864
H -5.1485652977 -5.4376525953 -2.2411149059
H -4.4542018557 -4.0975658103 -1.2806084644
H -4.8280877939 -3.8495767198 -2.9897957209
N 0.2091877688 -1.1020178853 -3.6085399668
C 1.1953260034 -1.1205000597 -4.2311557014
C 2.418709025 -1.1656229521 -4.9996226904
H 2.6085829962 -2.1961263882 -5.317163191
H 2.3462408033 -0.5234273904 -5.8829317753
H 3.258361436 -0.8315516714 -4.3805908604
N 3.0708940619 -3.316023144 -2.680993293
C 4.2017801808 -3.424238822 -2.420131971
C 5.6005330747 -3.5623761296 -2.0775391634
H 5.7476344196 -4.4328091432 -1.4290170886
H 6.207838594 -3.6948133973 -2.9784405933

H 5.9485458893 -2.6724289773 -1.5388232026

TS_A-B_ortho_2Kcation_MicroSolvation_acetonitrile_conf17
C 0.496807 0.409241 0.63765
C 1.150755 -0.413562 -1.198407
C 3.497155 0.20101 -1.180357
C 3.418609 2.691329 1.323527
C 3.984163 2.01459 2.374356
C 3.406409 0.872154 2.933442
C 2.255195 0.337494 2.431779
C 1.650792 0.960282 1.30601
C 2.21473 2.176776 0.767262
C 5.347267 1.226377 3.935451
H 3.895304 3.577947 0.918164
H 1.817236 -0.54253 2.888666
H 5.6112 1.599103 4.929623
O 5.114461 2.334482 3.063516
O 4.153694 0.465008 4.011024
H 6.158762 0.607663 3.516093
N 1.511998 2.785627 -0.248105
S 2.215504 3.855621 -1.209275
O 3.595884 3.555208 -1.614941
O 1.238192 4.071366 -2.313569
C -1.10062 -2.540691 2.45998
C -2.44877 -2.220464 2.321618
C -0.121227 -1.722445 1.909446
H -3.213145 -2.863846 2.75326
H 0.919776 -2.027722 1.980167
C -2.810436 -1.076165 1.61338
C -0.461429 -0.543043 1.222312
H -3.860362 -0.820007 1.483904
C -1.830169 -0.256481 1.068936
H -2.124325 0.636688 0.517605
H -0.806002 -3.443084 2.993774
C 3.396436 6.224062 -0.401646
C 3.396678 7.458573 0.241457
C 2.264448 5.41667 -0.331488
H 4.281648 8.092182 0.186152

C 2.282927 7.901789 0.963793
C 1.140389 5.836189 0.380557
H 0.256091 5.199542 0.419383
C 1.155629 7.070289 1.019897
H 0.277008 7.400627 1.575169
H 4.264588 5.875302 -0.957615
C 2.306729 9.215056 1.683861
H 2.926775 9.953426 1.164203
H 2.724919 9.105038 2.693235
H 1.302054 9.635866 1.799635
H -0.023294 1.177648 0.074304
H 4.302047 0.886184 -1.442651
C 2.238294 0.394371 -1.663528
C 1.447401 -1.744984 -0.692761
C 2.776371 -1.895753 -0.083881
C 3.770475 -0.899994 -0.30057
C 5.026262 -1.064572 0.317273
C 5.286701 -2.160006 1.124309
C 4.307257 -3.143979 1.326144
C 3.070407 -3.009715 0.713448
H 2.034507 1.228282 -2.33075
H 0.185411 -0.299278 -1.69009
O 0.604858 -2.667229 -0.709939
H 2.283616 -3.749241 0.863674
H 4.515598 -3.997636 1.967817
H 6.259832 -2.260698 1.6029
H 5.792942 -0.305855 0.15532
K -1.049533 2.750938 -1.74081
K -0.676201 -2.34642 -3.194402
N 0.184153 0.129653 -4.37713
C 0.633786 1.143651 -4.735432
C 1.208593 2.398794 -5.16359
H 1.467431 3.01393 -4.290729
H 2.112584 2.219474 -5.754114
H 0.483732 2.950248 -5.769684
N -0.732214 -3.787307 -5.747646
C -0.056788 -4.728651 -5.618099
C 0.776974 -5.895802 -5.43883

H 1.197149 -6.224592 -6.394239
H 1.59389 -5.649442 -4.75212
H 0.191655 -6.712891 -5.00486
N 2.102602 -3.508582 -3.366725
C 3.212979 -3.327465 -3.060017
C 4.593889 -3.106674 -2.688884
H 4.832495 -2.040386 -2.759038
H 4.766896 -3.424339 -1.653958
H 5.262585 -3.664162 -3.352576
N -1.029461 -5.299428 -2.637008
C -0.3382 -5.447645 -1.709484
C 0.519552 -5.642647 -0.563388
H 0.510845 -6.692066 -0.252162
H 1.540844 -5.34018 -0.816298
H 0.182635 -5.003672 0.258278
N -3.507212 -2.824815 -3.781167
C -3.550813 -3.884313 -4.26528
C -3.590906 -5.200871 -4.859258
H -4.594468 -5.630276 -4.78147
H -3.304496 -5.142667 -5.913735
H -2.879156 -5.846361 -4.333316
N -1.573736 3.306399 1.08923
C -0.863729 3.2483 2.012507
C 0.018028 3.189449 3.15638
H 0.141644 2.151757 3.484783
H 1.006714 3.577993 2.88396
H -0.386243 3.782207 3.983297
N -4.063667 3.00075 -1.586751
C -4.173345 3.830883 -0.774708
C -4.297879 4.876144 0.214519
H -4.045905 4.484927 1.204264
H -3.594904 5.67902 -0.032864
H -5.317304 5.274014 0.231119
N -1.76867 5.739416 -1.837517
C -0.839387 6.346024 -2.19822
C 0.312118 7.090907 -2.654801
H 0.703089 7.718026 -1.846069
H 1.0909 6.379922 -2.950813

H 0.047641 7.726871 -3.505488
N -1.961025 3.253634 -4.439122
C -2.900633 3.941972 -4.38431
C -4.059426 4.802674 -4.303912
H -4.95428 4.212046 -4.08431
H -3.914028 5.525338 -3.49358
H -4.207682 5.341526 -5.244824
N -2.176065 0.138828 -2.251194
C -3.141547 0.234115 -2.897661
C -4.336065 0.363672 -3.696051
H -4.868491 -0.591755 -3.714296
H -4.97192 1.144957 -3.267148
H -4.069668 0.636409 -4.722486

TS_A-B_ortho_2Kcation_MicroSolvation_acetonitrile_conf18
C 0.877585 0.736898 0.344689
C 1.55476 0.135866 -1.662806
C 3.461441 1.417124 -2.501309
C 2.050742 -2.249407 2.298938
C 0.836683 -2.838291 2.523825
C -0.372818 -2.280467 2.093957
C -0.404791 -1.107512 1.405785
C 0.827666 -0.45994 1.105648
C 2.080771 -1.035969 1.554601
C -0.80723 -4.249822 3.028701
H 2.960209 -2.723681 2.650069
H -1.3459 -0.646749 1.129752
H -1.239906 -4.498935 4.002959
O 0.591371 -3.996635 3.191832
O -1.412295 -3.061106 2.532844
H -0.938712 -5.06291 2.301043
N 3.213298 -0.35207 1.21165
S 4.65691 -0.969547 1.574897
O 4.874838 -2.378265 1.203297
O 5.640558 0.0125 1.055394
C -2.519495 1.791979 -1.000084
C -2.501086 3.15851 -0.720243
C -1.433943 0.993253 -0.65681

H -3.353263 3.784208 -0.978938
H -1.460798 -0.066183 -0.90836
C -1.381144 3.71367 -0.101903
C -0.300797 1.535297 -0.024798
H -1.362618 4.77696 0.136412
C -0.287735 2.916465 0.228954
H 0.580055 3.34889 0.727616
H -3.376742 1.339284 -1.498306
C 5.501389 -1.912159 4.02987
C 5.670844 -1.83069 5.408625
C 4.797141 -0.911418 3.363957
H 6.220131 -2.614212 5.930481
C 5.138548 -0.763287 6.142143
C 4.250735 0.158239 4.072843
H 3.682692 0.92629 3.547426
C 4.427147 0.226017 5.450042
H 3.993184 1.057463 6.006828
H 5.903588 -2.748965 3.461644
C 5.288584 -0.702019 7.631278
H 5.326355 0.330299 7.996
H 6.193289 -1.216658 7.97206
H 4.439507 -1.184654 8.133106
H 1.768332 1.331809 0.519135
H 3.88951 2.354476 -2.85963
C 2.131446 1.326231 -2.198467
C 2.367235 -1.057616 -1.510995
C 3.784719 -0.954784 -1.892116
C 4.312078 0.266586 -2.396011
C 5.650304 0.294326 -2.840492
C 6.435523 -0.845101 -2.803154
C 5.909261 -2.049988 -2.31191
C 4.597979 -2.096399 -1.869803
H 1.48946 2.202416 -2.316145
H 0.499602 -0.057779 -1.848712
O 1.864637 -2.135219 -1.116796
H 4.163798 -3.020034 -1.49513
H 6.529466 -2.943744 -2.279587
H 7.460232 -0.810855 -3.17276

H 6.052204 1.227625 -3.235861
K -0.458523 -3.109733 -2.179789
N -2.785607 -2.538856 -0.621492
C -3.461661 -2.173201 0.252874
C -4.265709 -1.700646 1.357961
H -3.812769 -2.022711 2.301355
H -4.31725 -0.606314 1.347712
H -5.283659 -2.097552 1.297605
N -0.68909 -5.474631 -3.760731
C -0.894657 -6.460151 -4.344549
C -1.151755 -7.685372 -5.069599
H -1.48279 -8.471967 -4.38459
H -1.932266 -7.528079 -5.820356
H -0.245137 -8.027093 -5.578325

6. References

1. Liu, X.; Wang, K.; Guo, W.; Liu, Y.; Li, C. An organic-base catalyzed asymmetric 1,4-addition of tritylthiol to *in situ* generated aza-*o*-quinone methides at the H_2O/DCM interface. *Chem. Communs.* **2019**, *55*, 2668.
2. Yang, F.; Zhou, X.; Wei, Y.; Wang, L.; Jiang, J. Hydroquinine-catalyzed asymmetric 1,4-hydrophosphination of *in situ* generated aza-*o*-quinone methides with *H*-phosphine oxides. *Org. Chem. Front.* **2021**, *8*, 5064.
3. Zhao, Y.; Truhlar, D. G., A new local density functional for main-group thermochemistry, transition metal bonding, thermochemical kinetics, and noncovalent interactions. *J. Chem. Phys.* **2006,** *19*, 125.
4. Marenich, A. V.; Cramer, C. J.; Truhlar, D. G., Universal Solvation Model Based on Solute Electron Density and on a Continuum Model of the Solvent Defined by the Bulk Dielectric Constant and Atomic Surface Tensions. *J. Phys. Chem. B* **2009,** *113,* 6378.
5. Frisch, M. J.; Trucks, G. W.; Schlegel, H. B.; Scuseria, G. E.; Robb, M. A.; Cheeseman, J. R.; Scalmani, G.; Barone, V.; Petersson, G. A.; Nakatsuji, H.; Li, X.; Caricato, M.; Marenich, A. V.; Bloino, J.; Janesko, B. G.; Gomperts, R.; Mennucci, B.; Hratchian, H. P.; Ortiz, J. V.; Izmaylov, A. F.; Sonnenberg, J. L.; Williams; Ding, F.; Lipparini, F.; Egidi, F.; Goings, J.; Peng, B.; Petrone, A.; Henderson, T.; Ranasinghe, D.; Zakrzewski, V. G.; Gao, J.; Rega, N.; Zheng, G.; Liang, W.; Hada, M.; Ehara, M.; Toyota, K.; Fukuda, R.; Hasegawa, J.; Ishida, M.; Nakajima, T.; Honda, Y.; Kitao, O.; Nakai, H.; Vreven, T.; Throssell, K.; Montgomery Jr., J. A.; Peralta, J. E.; Ogliaro, F.; Bearpark, M. J.; Heyd, J. J.; Brothers, E. N.; Kudin, K. N.; Staroverov, V. N.; Keith, T. A.; Kobayashi, R.; Normand, J.; Raghavachari, K.; Rendell, A. P.; Burant, J. C.; Iyengar, S. S.; Tomasi, J.; Cossi, M.; Millam, J. M.; Klene, M.; Adamo, C.; Cammi, R.; Ochterski, J. W.; Martin, R. L.; Morokuma, K.; Farkas, O.; Foresman, J. B.; Fox, D. J. *Gaussian 16 Rev. B.01*, Wallingford, CT, 2016.
6. Maurer, L. R.; Bursch, M.; Grimme, S.; Hansen, A., Assessing Density Functional Theory for Chemically Relevant Open-Shell Transition Metal Reactions. *J. Chem. Theory Comput.* **2021,** *17*, 6134.
7. Neese, F., The ORCA program system. *WIRES Comp Mol Sci* **2012,** *2* , 73.
8. Neese, F., Software update: the ORCA program system, version 4.0. *WIRES Comp Mol Sci* **2018,** *8* , e1327.
9. Lu, T.; Chen, F., Multiwfn: A multifunctional wavefunction analyzer. *J. Comput. Chem.* **2012,** *33* , 580.
10. Zhurko, G. A., http://www.chemcraftprog.com.
11. Spartan 18, D., B.J. Driessen, A.J. Hehre, W.J. Hehre, T.S. Johnson, J.A. Ohlinger, S. Klunzinger , P.E. Wavefunction Inc. Irvine CA.
12. Pracht, P.; Bohle, F.; Grimme, S., Automated exploration of the low-energy chemical space

with fast quantum chemical methods. *Phys. Chem. Chem. Phys.* **2020,** *22* , 7169.

13. Spicher, S.; Plett, C.; Pracht, P.; Hansen, A.; Grimme, S., Automated Molecular Cluster Growing for Explicit Solvation by Efficient Force Field and Tight Binding Methods. *J. Chem. Theory. and Comput.* **2022,** *18*, 3174.
14. Luchini, G.; Alegre-Requena, J.; Funes-Ardoiz, I.; Paton, R., GoodVibes: automated thermochemistry for heterogeneous computational chemistry data [version 1; peer review: 2 approved with reservations]. *F1000Research* **2020,** *9* (291).
15. Gogoi, R.; Jindal, G., Cesium and Phenoxide Interaction Enabled by Rh Overrides the Innate Solvent-Mediated Chemoselectivity. *ACS Catal.* **2024,** *14*, 12351.
16. Spångberg, D.; Hermansson, K., The solvation of Li+ and Na+ in acetonitrile from ab initio-derived many-body ion–solvent potentials. *Chem. Phys.* **2004,** *300*, 165.

7. X-Ray Analysis Data

CCDC 2387869 (6)

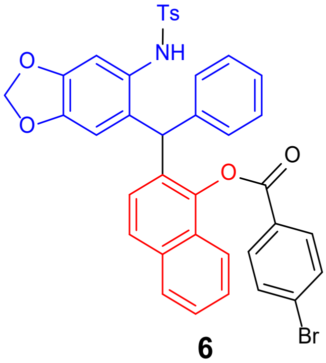

6

ORTEP drawing of **5g** showing thermal ellipsoids at the 50% probability level

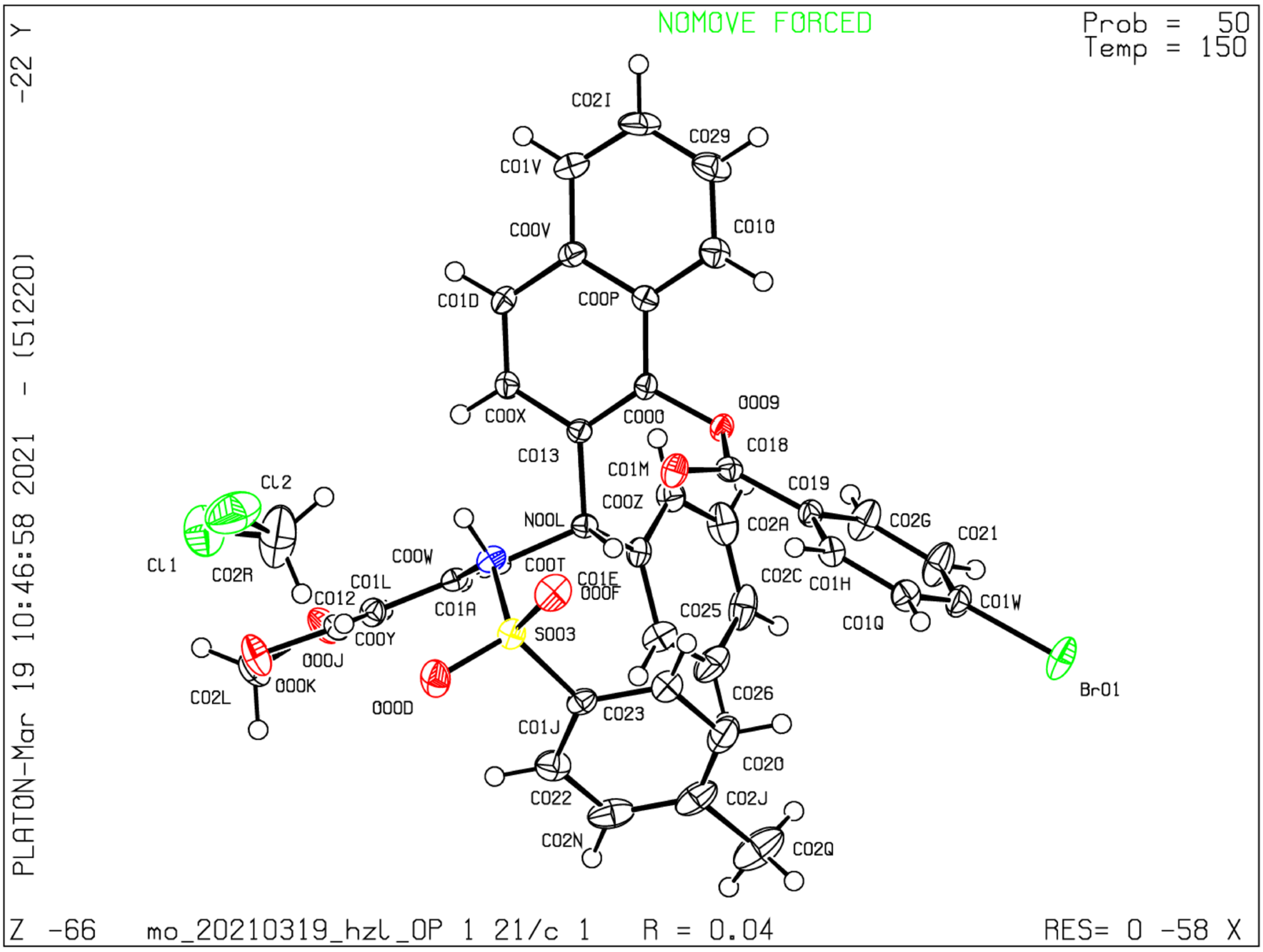

The crystal was obtained by slow evaporation of **6** in Hexane : DCM = 1:1 at room temperature.

Table S15. Crystal data and structure refinement for **6**.

Identification code	mo_20210319_hzl_0m_pl	
Empirical formula	C39 H30 Br Cl2 N O6 S	
Formula weight	791.51	
Temperature	150.15 K	
Wavelength	0.71076 Å	
Crystal system	Monoclinic	
Space group	P 1 21/c 1	
Unit cell dimensions	a = 17.6075(16) Å	a= 90°.
	b = 9.4996(9) Å	b= 90.271(4)°.
	c = 21.1754(17) Å	g = 90°.
Volume	3541.8(5) $Å^3$	
Z	4	
Density (calculated)	1.484 Mg/m^3	
Absorption coefficient	1.420 mm^{-1}	
F(000)	1616	
Crystal size	0.13 x 0.101 x 0.1 mm^3	
Theta range for data collection	2.314 to 26.395°.	
Index ranges	-22<=h<=22, -11<=k<=11, -26<=l<=26	
Reflections collected	102346	
Independent reflections	7229 [R(int) = 0.0544]	
Completeness to theta = 25.243°	99.8 %	
Absorption correction	None	
Max. and min. transmission	0.7454 and 0.6148	
Refinement method	Full-matrix least-squares on F^2	
Data / restraints / parameters	7229 / 0 / 452	
Goodness-of-fit on F^2	1.143	
Final R indices [I>2sigma(I)]	R1 = 0.0389, wR2 = 0.1085	
R indices (all data)	R1 = 0.0480, wR2 = 0.1256	
Extinction coefficient	n/a	
Largest diff. peak and hole	1.262 and -1.061 e.$Å^{-3}$	

Table S16. Atomic coordinates (x 10^4) and equivalent isotropic displacement parameters ($Å^2$x 10^3) for **6**. U(eq) is defined as one third of the trace of the orthogonalized U^{ij} tensor.

	x	y	z	U(eq)
Br(01)	3092(1)	-996(1)	7478(1)	38(1)
S(003)	1205(1)	3289(1)	4112(1)	19(1)
O(009)	4065(1)	1603(2)	4615(1)	18(1)
O(00C)	3147(1)	3191(2)	4829(1)	22(1)
O(00D)	552(1)	3563(2)	3728(1)	30(1)
O(00F)	1436(1)	4285(2)	4582(1)	29(1)
O(00J)	1857(1)	-335(2)	1542(1)	28(1)
O(00K)	977(1)	1477(2)	1601(1)	32(1)
N(00L)	1942(1)	3138(2)	3637(1)	18(1)
C(00O)	4177(1)	2195(2)	4013(1)	16(1)
C(00P)	4820(1)	3068(2)	3942(1)	17(1)
C(00T)	2443(1)	1012(2)	3108(1)	16(1)
C(00V)	4998(1)	3539(3)	3321(1)	19(1)
C(00W)	1927(1)	2133(2)	3127(1)	16(1)
C(00X)	3899(1)	2312(3)	2914(1)	20(1)
C(00Y)	1934(1)	356(3)	2108(1)	20(1)
C(00Z)	3035(1)	828(2)	3631(1)	16(1)
C(012)	1412(1)	1436(3)	2143(1)	20(1)
C(013)	3710(1)	1808(2)	3524(1)	16(1)
C(018)	3521(1)	2181(2)	4986(1)	17(1)
C(019)	3440(1)	1399(3)	5587(1)	18(1)
C(01A)	2451(1)	112(3)	2580(1)	19(1)
C(01D)	4518(1)	3140(3)	2815(1)	23(1)
C(01E)	3267(1)	-707(3)	3737(1)	18(1)
C(01H)	2962(1)	1945(3)	6049(1)	19(1)
C(01J)	1093(1)	1627(3)	4461(1)	20(1)
C(01L)	1393(1)	2356(3)	2642(1)	19(1)
C(01M)	4005(2)	-1193(3)	3661(1)	23(1)
C(01O)	5302(1)	3455(3)	4451(1)	23(1)
C(01Q)	2851(1)	1227(3)	6611(1)	23(1)
C(01V)	5657(2)	4362(3)	3229(1)	28(1)
C(01W)	3218(2)	-41(3)	6704(1)	24(1)
C(021)	3687(2)	-612(3)	6249(1)	35(1)

C(022)	673(2)	588(3)	4162(1)	28(1)
C(023)	1475(2)	1344(3)	5022(1)	28(1)
C(025)	2715(2)	-1655(3)	3943(1)	26(1)
C(026)	2898(2)	-3052(3)	4072(1)	33(1)
C(029)	5938(2)	4246(3)	4341(1)	32(1)
C(02A)	4185(2)	-2594(3)	3790(1)	32(1)
C(02C)	3635(2)	-3516(3)	3994(1)	33(1)
C(02G)	3801(2)	113(3)	5689(1)	31(1)
C(02I)	6119(2)	4692(3)	3724(2)	33(1)
C(02J)	1042(2)	-1055(3)	4986(2)	34(1)
C(02L)	1161(2)	193(3)	1273(1)	29(1)
C(02N)	649(2)	-754(3)	4431(2)	35(1)
C(02O)	1448(2)	2(3)	5276(1)	35(1)
C(02Q)	1031(2)	-2516(4)	5266(2)	51(1)
Cl(1)	984(1)	5870(1)	1962(1)	56(1)
Cl(2)	618(1)	8225(1)	2793(1)	65(1)
C(02R)	917(3)	6463(6)	2748(2)	71(1)

Table S17. Bond lengths [Å] and angles [°] for **6**.

Br(01)-C(01W)	1.887(2)
S(003)-O(00D)	1.4295(19)
S(003)-O(00F)	1.431(2)
S(003)-N(00L)	1.651(2)
S(003)-C(01J)	1.755(3)
O(009)-C(00O)	1.407(3)
O(009)-C(018)	1.359(3)
O(00C)-C(018)	1.208(3)
O(00J)-C(00Y)	1.373(3)
O(00J)-C(02L)	1.439(3)
O(00K)-C(012)	1.376(3)
O(00K)-C(02L)	1.441(3)
N(00L)-C(00W)	1.441(3)
C(00O)-C(00P)	1.411(3)
C(00O)-C(013)	1.369(3)
C(00P)-C(00V)	1.425(3)
C(00P)-C(01O)	1.417(3)
C(00T)-C(00W)	1.400(3)
C(00T)-C(00Z)	1.527(3)
C(00T)-C(01A)	1.407(3)
C(00V)-C(01D)	1.414(3)
C(00V)-C(01V)	1.413(3)
C(00W)-C(01L)	1.405(3)
C(00X)-C(013)	1.419(3)
C(00X)-C(01D)	1.362(3)
C(00Y)-C(012)	1.379(4)
C(00Y)-C(01A)	1.369(3)
C(00Z)-C(013)	1.528(3)
C(00Z)-C(01E)	1.530(3)
C(012)-C(01L)	1.373(3)
C(018)-C(019)	1.480(3)
C(019)-C(01H)	1.393(3)
C(019)-C(02G)	1.394(4)
C(01E)-C(01M)	1.390(3)
C(01E)-C(025)	1.397(4)
C(01H)-C(01Q)	1.386(4)

C(01J)-C(022)	1.385(4)
C(01J)-C(023)	1.387(4)
C(01M)-C(02A)	1.395(4)
C(01O)-C(029)	1.370(4)
C(01Q)-C(01W)	1.381(4)
C(01V)-C(02I)	1.360(4)
C(01W)-C(021)	1.383(4)
C(021)-C(02G)	1.387(4)
C(022)-C(02N)	1.398(4)
C(023)-C(02O)	1.385(4)
C(025)-C(026)	1.392(4)
C(026)-C(02C)	1.382(4)
C(029)-C(02I)	1.411(4)
C(02A)-C(02C)	1.377(4)
C(02J)-C(02N)	1.390(5)
C(02J)-C(02O)	1.376(5)
C(02J)-C(02Q)	1.509(4)
Cl(1)-C(02R)	1.762(5)
Cl(2)-C(02R)	1.757(5)
O(00D)-S(003)-O(00F)	120.04(12)
O(00D)-S(003)-N(00L)	107.50(11)
O(00D)-S(003)-C(01J)	108.18(12)
O(00F)-S(003)-N(00L)	105.12(11)
O(00F)-S(003)-C(01J)	109.47(12)
N(00L)-S(003)-C(01J)	105.59(11)
C(018)-O(009)-C(00O)	117.71(18)
C(00Y)-O(00J)-C(02L)	104.94(19)
C(012)-O(00K)-C(02L)	104.6(2)
C(00W)-N(00L)-S(003)	120.18(16)
O(009)-C(00O)-C(00P)	116.57(19)
C(013)-C(00O)-O(009)	119.4(2)
C(013)-C(00O)-C(00P)	123.7(2)
C(00O)-C(00P)-C(00V)	117.6(2)
C(00O)-C(00P)-C(01O)	123.3(2)
C(01O)-C(00P)-C(00V)	119.0(2)
C(00W)-C(00T)-C(00Z)	120.5(2)
C(00W)-C(00T)-C(01A)	119.6(2)

C(01A)-C(00T)-C(00Z)	119.8(2)
C(01D)-C(00V)-C(00P)	118.7(2)
C(01V)-C(00V)-C(00P)	119.1(2)
C(01V)-C(00V)-C(01D)	122.2(2)
C(00T)-C(00W)-N(00L)	121.0(2)
C(00T)-C(00W)-C(01L)	121.7(2)
C(01L)-C(00W)-N(00L)	117.2(2)
C(01D)-C(00X)-C(013)	121.7(2)
O(00J)-C(00Y)-C(012)	109.9(2)
C(01A)-C(00Y)-O(00J)	128.3(2)
C(01A)-C(00Y)-C(012)	121.8(2)
C(00T)-C(00Z)-C(013)	110.62(18)
C(00T)-C(00Z)-C(01E)	113.38(19)
C(013)-C(00Z)-C(01E)	113.26(19)
O(00K)-C(012)-C(00Y)	110.2(2)
C(01L)-C(012)-O(00K)	127.4(2)
C(01L)-C(012)-C(00Y)	122.3(2)
C(00O)-C(013)-C(00X)	117.1(2)
C(00O)-C(013)-C(00Z)	121.1(2)
C(00X)-C(013)-C(00Z)	121.7(2)
O(009)-C(018)-C(019)	111.5(2)
O(00C)-C(018)-O(009)	123.1(2)
O(00C)-C(018)-C(019)	125.4(2)
C(01H)-C(019)-C(018)	118.5(2)
C(01H)-C(019)-C(02G)	119.6(2)
C(02G)-C(019)-C(018)	121.8(2)
C(00Y)-C(01A)-C(00T)	117.9(2)
C(00X)-C(01D)-C(00V)	121.0(2)
C(01M)-C(01E)-C(00Z)	123.3(2)
C(01M)-C(01E)-C(025)	118.3(2)
C(025)-C(01E)-C(00Z)	118.3(2)
C(01Q)-C(01H)-C(019)	120.5(2)
C(022)-C(01J)-S(003)	120.6(2)
C(022)-C(01J)-C(023)	120.7(2)
C(023)-C(01J)-S(003)	118.6(2)
C(012)-C(01L)-C(00W)	116.6(2)
C(01E)-C(01M)-C(02A)	120.4(3)
C(029)-C(01O)-C(00P)	120.1(2)

C(01W)-C(01Q)-C(01H)	119.0(2)
C(02I)-C(01V)-C(00V)	120.7(3)
C(01Q)-C(01W)-Br(01)	119.14(19)
C(01Q)-C(01W)-C(021)	121.6(2)
C(021)-C(01W)-Br(01)	119.3(2)
C(01W)-C(021)-C(02G)	119.3(3)
C(01J)-C(022)-C(02N)	118.7(3)
C(02O)-C(023)-C(01J)	119.5(3)
C(026)-C(025)-C(01E)	121.0(3)
C(02C)-C(026)-C(025)	119.9(3)
C(01O)-C(029)-C(02I)	120.7(3)
C(02C)-C(02A)-C(01M)	120.6(3)
C(02A)-C(02C)-C(026)	119.8(3)
C(021)-C(02G)-C(019)	120.0(2)
C(01V)-C(02I)-C(029)	120.5(2)
C(02N)-C(02J)-C(02Q)	120.9(3)
C(02O)-C(02J)-C(02N)	118.9(3)
C(02O)-C(02J)-C(02Q)	120.2(3)
O(00J)-C(02L)-O(00K)	107.34(19)
C(02J)-C(02N)-C(022)	121.1(3)
C(02J)-C(02O)-C(023)	121.1(3)
Cl(2)-C(02R)-Cl(1)	112.2(2)

Symmetry transformations used to generate equivalent atoms:

Table S18. Anisotropic displacement parameters ($Å^2$x 10^3) for **6**. The anisotropic displacement factor exponent takes the form: $-2p^2[h^2 a^{*2}U^{11} + ... + 2 h k a^* b^* U^{12}]$

	U^{11}	U^{22}	U^{33}	U^{23}	U^{13}	U^{12}
Br(01)	52(1)	40(1)	21(1)	13(1)	7(1)	4(1)
S(003)	21(1)	17(1)	19(1)	-1(1)	3(1)	6(1)
O(009)	18(1)	21(1)	14(1)	3(1)	2(1)	2(1)
O(00C)	29(1)	18(1)	20(1)	2(1)	1(1)	6(1)
O(00D)	24(1)	37(1)	28(1)	5(1)	2(1)	13(1)
O(00F)	39(1)	20(1)	28(1)	-7(1)	6(1)	3(1)
O(00J)	25(1)	36(1)	24(1)	-14(1)	-6(1)	2(1)
O(00K)	31(1)	39(1)	25(1)	-9(1)	-12(1)	8(1)
N(00L)	21(1)	13(1)	20(1)	-1(1)	4(1)	0(1)
C(00O)	17(1)	17(1)	14(1)	2(1)	1(1)	3(1)
C(00P)	16(1)	16(1)	19(1)	-2(1)	1(1)	2(1)
C(00T)	12(1)	17(1)	18(1)	-1(1)	2(1)	-3(1)
C(00V)	18(1)	17(1)	22(1)	2(1)	2(1)	0(1)
C(00W)	17(1)	14(1)	16(1)	-1(1)	3(1)	-2(1)
C(00X)	18(1)	27(1)	16(1)	2(1)	-1(1)	-1(1)
C(00Y)	18(1)	21(1)	20(1)	-5(1)	2(1)	-5(1)
C(00Z)	15(1)	16(1)	16(1)	-2(1)	2(1)	0(1)
C(012)	16(1)	26(1)	18(1)	1(1)	-3(1)	-3(1)
C(013)	13(1)	18(1)	17(1)	1(1)	2(1)	2(1)
C(018)	18(1)	17(1)	16(1)	-4(1)	0(1)	-2(1)
C(019)	19(1)	20(1)	15(1)	0(1)	0(1)	0(1)
C(01A)	15(1)	19(1)	22(1)	-4(1)	2(1)	1(1)
C(01D)	22(1)	28(1)	18(1)	7(1)	1(1)	-2(1)
C(01E)	22(1)	18(1)	14(1)	-1(1)	1(1)	2(1)
C(01H)	22(1)	19(1)	18(1)	-3(1)	0(1)	3(1)
C(01J)	18(1)	20(1)	22(1)	1(1)	6(1)	2(1)
C(01L)	20(1)	18(1)	20(1)	2(1)	0(1)	3(1)
C(01M)	22(1)	24(1)	23(1)	-1(1)	0(1)	4(1)
C(01O)	25(1)	24(1)	20(1)	-5(1)	1(1)	-1(1)
C(01Q)	24(1)	28(1)	18(1)	-4(1)	3(1)	3(1)
C(01V)	26(1)	26(1)	31(1)	4(1)	6(1)	-6(1)
C(01W)	28(1)	29(1)	15(1)	5(1)	2(1)	0(1)
C(021)	45(2)	32(2)	28(1)	11(1)	11(1)	17(1)

C(022)	26(1)	31(1)	27(1)	-5(1)	5(1)	-3(1)
C(023)	28(1)	29(1)	26(1)	4(1)	1(1)	-2(1)
C(025)	28(1)	22(1)	28(1)	1(1)	7(1)	0(1)
C(026)	50(2)	21(1)	28(1)	2(1)	6(1)	-5(1)
C(029)	26(1)	34(2)	35(2)	-10(1)	-2(1)	-9(1)
C(02A)	36(2)	29(1)	29(1)	-5(1)	-5(1)	15(1)
C(02C)	57(2)	20(1)	22(1)	1(1)	-3(1)	10(1)
C(02G)	39(2)	29(1)	25(1)	6(1)	13(1)	16(1)
C(02I)	26(1)	34(2)	41(2)	-6(1)	4(1)	-16(1)
C(02J)	38(2)	26(1)	39(2)	5(1)	22(1)	5(1)
C(02L)	25(1)	38(2)	24(1)	-7(1)	-6(1)	0(1)
C(02N)	37(2)	27(1)	41(2)	-11(1)	16(1)	-9(1)
C(02O)	38(2)	36(2)	30(1)	13(1)	5(1)	3(1)
C(02Q)	65(2)	28(2)	60(2)	10(2)	28(2)	5(2)
Cl(1)	64(1)	53(1)	50(1)	1(1)	-11(1)	7(1)
Cl(2)	80(1)	58(1)	56(1)	-9(1)	16(1)	-29(1)
C(02R)	75(3)	98(4)	42(2)	20(2)	6(2)	35(3)

Table S19. Atomic coordinates (x 10^4) and equivalent isotropic displacement parameters ($Å^2$x 10^3) for **6**.

	x	y	z	U(eq)
H(00L)	2085	3970	3499	21
H(00X)	3584	2065	2565	24
H(00Z)	2789	1147	4031	19
H(01A)	2804	-641	2553	23
H(01D)	4629	3455	2399	27
H(01H)	2711	2817	5978	23
H(01L)	1037	3106	2659	23
H(01M)	4390	-568	3519	28
H(01O)	5184	3165	4868	28
H(01Q)	2527	1601	6926	27
H(01V)	5779	4687	2818	33
H(021)	3930	-1492	6320	42
H(022)	406	785	3781	34
H(023)	1754	2066	5229	34
H(025)	2206	-1342	3996	31
H(026)	2517	-3685	4213	39
H(029)	6261	4497	4684	38
H(02A)	4692	-2915	3736	38
H(02C)	3762	-4469	4081	39
H(02G)	4125	-268	5375	37
H(02I)	6567	5227	3654	40
H(02B)	749	-504	1326	35
H(02D)	1226	380	817	35
H(02N)	359	-1473	4232	42
H(02O)	1714	-192	5657	42
H(02E)	773	-2493	5675	76
H(02F)	1553	-2850	5324	76
H(02H)	759	-3154	4980	76
H(02J)	552	5861	2979	86
H(02K)	1419	6368	2957	86

Table S6. Torsion angles [°] for **6**.

Br(01)-C(01W)-C(021)-C(02G)	-177.8(2)
S(003)-N(00L)-C(00W)-C(00T)	-116.9(2)
S(003)-N(00L)-C(00W)-C(01L)	65.2(3)
S(003)-C(01J)-C(022)-C(02N)	174.8(2)
S(003)-C(01J)-C(023)-C(02O)	-174.4(2)
O(009)-C(00O)-C(00P)-C(00V)	172.45(19)
O(009)-C(00O)-C(00P)-C(01O)	-5.5(3)
O(009)-C(00O)-C(013)-C(00X)	-173.1(2)
O(009)-C(00O)-C(013)-C(00Z)	4.5(3)
O(009)-C(018)-C(019)-C(01H)	173.3(2)
O(009)-C(018)-C(019)-C(02G)	-9.5(3)
O(00C)-C(018)-C(019)-C(01H)	-8.1(4)
O(00C)-C(018)-C(019)-C(02G)	169.1(3)
O(00D)-S(003)-N(00L)-C(00W)	-55.4(2)
O(00D)-S(003)-C(01J)-C(022)	28.9(2)
O(00D)-S(003)-C(01J)-C(023)	-155.4(2)
O(00F)-S(003)-N(00L)-C(00W)	175.68(18)
O(00F)-S(003)-C(01J)-C(022)	161.3(2)
O(00F)-S(003)-C(01J)-C(023)	-23.0(2)
O(00J)-C(00Y)-C(012)-O(00K)	0.3(3)
O(00J)-C(00Y)-C(012)-C(01L)	-175.9(2)
O(00J)-C(00Y)-C(01A)-C(00T)	176.8(2)
O(00K)-C(012)-C(01L)-C(00W)	-176.5(2)
N(00L)-S(003)-C(01J)-C(022)	-86.0(2)
N(00L)-S(003)-C(01J)-C(023)	89.7(2)
N(00L)-C(00W)-C(01L)-C(012)	176.6(2)
C(00O)-O(009)-C(018)-O(00C)	-1.9(3)
C(00O)-O(009)-C(018)-C(019)	176.75(19)
C(00O)-C(00P)-C(00V)-C(01D)	1.4(3)
C(00O)-C(00P)-C(00V)-C(01V)	-177.2(2)
C(00O)-C(00P)-C(01O)-C(029)	176.6(2)
C(00P)-C(00O)-C(013)-C(00X)	0.7(3)
C(00P)-C(00O)-C(013)-C(00Z)	178.4(2)
C(00P)-C(00V)-C(01D)-C(00X)	-0.5(4)
C(00P)-C(00V)-C(01V)-C(02I)	0.5(4)
C(00P)-C(01O)-C(029)-C(02I)	0.4(4)

C(00T)-C(00W)-C(01L)-C(012)	-1.3(3)
C(00T)-C(00Z)-C(013)-C(00O)	157.6(2)
C(00T)-C(00Z)-C(013)-C(00X)	-24.9(3)
C(00T)-C(00Z)-C(01E)-C(01M)	119.0(2)
C(00T)-C(00Z)-C(01E)-C(025)	-63.4(3)
C(00V)-C(00P)-C(01O)-C(029)	-1.3(4)
C(00V)-C(01V)-C(02I)-C(029)	-1.5(5)
C(00W)-C(00T)-C(00Z)-C(013)	-80.5(3)
C(00W)-C(00T)-C(00Z)-C(01E)	151.0(2)
C(00W)-C(00T)-C(01A)-C(00Y)	-1.6(3)
C(00Y)-O(00J)-C(02L)-O(00K)	17.2(3)
C(00Y)-C(012)-C(01L)-C(00W)	-1.0(4)
C(00Z)-C(00T)-C(00W)-N(00L)	0.0(3)
C(00Z)-C(00T)-C(00W)-C(01L)	177.8(2)
C(00Z)-C(00T)-C(01A)-C(00Y)	-176.9(2)
C(00Z)-C(01E)-C(01M)-C(02A)	177.4(2)
C(00Z)-C(01E)-C(025)-C(026)	-177.5(2)
C(012)-O(00K)-C(02L)-O(00J)	-17.0(3)
C(012)-C(00Y)-C(01A)-C(00T)	-0.6(4)
C(013)-C(00O)-C(00P)-C(00V)	-1.6(3)
C(013)-C(00O)-C(00P)-C(01O)	-179.6(2)
C(013)-C(00X)-C(01D)-C(00V)	-0.3(4)
C(013)-C(00Z)-C(01E)-C(01M)	-8.2(3)
C(013)-C(00Z)-C(01E)-C(025)	169.5(2)
C(018)-O(009)-C(00O)-C(00P)	103.5(2)
C(018)-O(009)-C(00O)-C(013)	-82.2(3)
C(018)-C(019)-C(01H)-C(01Q)	178.0(2)
C(018)-C(019)-C(02G)-C(021)	-177.6(3)
C(019)-C(01H)-C(01Q)-C(01W)	-0.3(4)
C(01A)-C(00T)-C(00W)-N(00L)	-175.2(2)
C(01A)-C(00T)-C(00W)-C(01L)	2.6(3)
C(01A)-C(00T)-C(00Z)-C(013)	94.7(2)
C(01A)-C(00T)-C(00Z)-C(01E)	-33.8(3)
C(01A)-C(00Y)-C(012)-O(00K)	178.2(2)
C(01A)-C(00Y)-C(012)-C(01L)	1.9(4)
C(01D)-C(00V)-C(01V)-C(02I)	-178.0(3)
C(01D)-C(00X)-C(013)-C(00O)	0.3(4)
C(01D)-C(00X)-C(013)-C(00Z)	-177.3(2)

C(01E)-C(00Z)-C(013)-C(00O)	-73.9(3)
C(01E)-C(00Z)-C(013)-C(00X)	103.6(3)
C(01E)-C(01M)-C(02A)-C(02C)	0.1(4)
C(01E)-C(025)-C(026)-C(02C)	-0.2(4)
C(01H)-C(019)-C(02G)-C(021)	-0.4(4)
C(01H)-C(01Q)-C(01W)-Br(01)	178.13(19)
C(01H)-C(01Q)-C(01W)-C(021)	-0.5(4)
C(01J)-S(003)-N(00L)-C(00W)	60.0(2)
C(01J)-C(022)-C(02N)-C(02J)	-0.5(4)
C(01J)-C(023)-C(02O)-C(02J)	-0.6(4)
C(01M)-C(01E)-C(025)-C(026)	0.3(4)
C(01M)-C(02A)-C(02C)-C(026)	0.0(4)
C(01O)-C(00P)-C(00V)-C(01D)	179.5(2)
C(01O)-C(00P)-C(00V)-C(01V)	0.9(3)
C(01O)-C(029)-C(02I)-C(01V)	1.1(5)
C(01Q)-C(01W)-C(021)-C(02G)	0.8(5)
C(01V)-C(00V)-C(01D)-C(00X)	178.0(3)
C(01W)-C(021)-C(02G)-C(019)	-0.4(5)
C(022)-C(01J)-C(023)-C(02O)	1.3(4)
C(023)-C(01J)-C(022)-C(02N)	-0.8(4)
C(025)-C(01E)-C(01M)-C(02A)	-0.3(4)
C(025)-C(026)-C(02C)-C(02A)	0.1(4)
C(02G)-C(019)-C(01H)-C(01Q)	0.7(4)
C(02L)-O(00J)-C(00Y)-C(012)	-11.0(3)
C(02L)-O(00J)-C(00Y)-C(01A)	171.4(3)
C(02L)-O(00K)-C(012)-C(00Y)	10.4(3)
C(02L)-O(00K)-C(012)-C(01L)	-173.6(3)
C(02N)-C(02J)-C(02O)-C(023)	-0.7(4)
C(02O)-C(02J)-C(02N)-C(022)	1.2(4)
C(02Q)-C(02J)-C(02N)-C(022)	-178.4(3)
C(02Q)-C(02J)-C(02O)-C(023)	179.0(3)

Symmetry transformations used to generate equivalent atoms:

CCDC 2387872 (7)

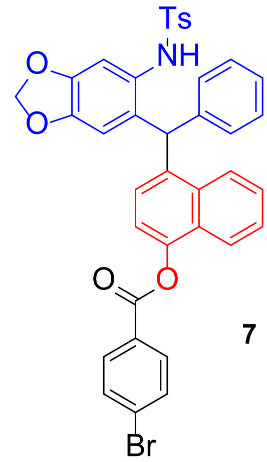

7

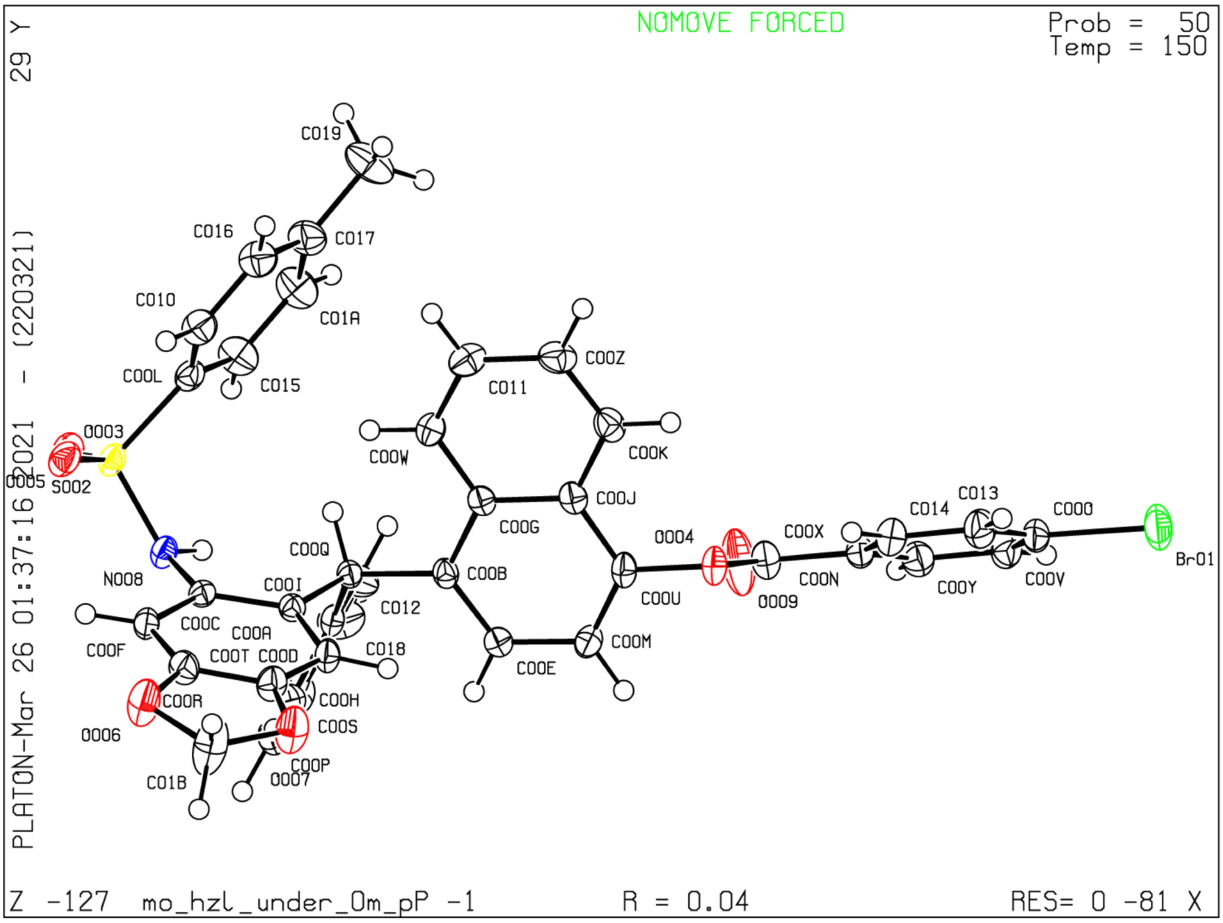

ORTEP drawing of **8** showing thermal ellipsoids at the 50% probability level

The crystal was obtained by slow evaporation of **7** in Hexane : DCM = 1:1 at room temperature.

Table S20. Crystal data and structure refinement for **7**.

Identification code	mo_hzl_under_0m_pl	
Empirical formula	C38 H28 Br N O6 S	
Formula weight	706.58	
Temperature	150.15 K	
Wavelength	0.71076 Å	
Crystal system	Triclinic	
Space group	P-1	
Unit cell dimensions	a = 9.4775(7) Å	a= 104.246(3)°.
	b = 11.0642(7) Å	b= 103.202(3)°.
	c = 16.3217(10) Å	g = 98.673(3)°.
Volume	1575.70(18) $Å^3$	
Z	2	
Density (calculated)	1.489 Mg/m^3	
Absorption coefficient	1.422 mm^{-1}	
F(000)	724	
Crystal size	? x ? x ? mm^3	
Theta range for data collection	2.262 to 26.396°.	
Index ranges	-11<=h<=11, -13<=k<=13, -20<=l<=20	
Reflections collected	37505	
Independent reflections	6422 [R(int) = 0.0443]	
Completeness to theta = 25.243°	99.4 %	
Absorption correction	None	
Max. and min. transmission	0.7454 and 0.6756	
Refinement method	Full-matrix least-squares on F^2	
Data / restraints / parameters	6422 / 0 / 425	
Goodness-of-fit on F^2	1.038	
Final R indices [I>2sigma(I)]	R1 = 0.0390, wR2 = 0.1017	
R indices (all data)	R1 = 0.0472, wR2 = 0.1082	
Extinction coefficient	n/a	
Largest diff. peak and hole	1.718 and -0.587 e.$Å^{-3}$	

Table S21. Atomic coordinates (x 10^4) and equivalent isotropic displacement parameters (Å^{2}x 10^3) for **7**. U(eq) is defined as one third of the trace of the orthogonalized U^{ij} tensor.

	x	y	z	U(eq)
Br(01)	506(1)	6950(1)	9863(1)	42(1)
S(002)	5006(1)	-364(1)	1298(1)	23(1)
O(003)	6070(2)	367(2)	1015(1)	30(1)
O(004)	1934(2)	4583(2)	5916(1)	26(1)
O(005)	4414(2)	-1694(2)	853(1)	33(1)
O(006)	4732(2)	4371(2)	643(1)	39(1)
O(007)	3836(2)	5443(2)	1757(1)	38(1)
O(009)	333(3)	2877(2)	5934(1)	54(1)
N(008)	3568(2)	287(2)	1231(1)	21(1)
C(00A)	3151(2)	2260(2)	2123(1)	18(1)
C(00B)	2412(2)	2382(2)	3534(1)	19(1)
C(00C)	3677(2)	1636(2)	1436(1)	19(1)
C(00D)	3139(3)	3560(2)	2259(1)	22(1)
C(00E)	1104(3)	2739(2)	3580(2)	23(1)
C(00F)	4234(3)	2272(2)	896(2)	22(1)
C(00G)	3669(2)	2851(2)	4296(1)	19(1)
C(00H)	227(3)	570(2)	1382(2)	24(1)
C(00I)	1105(2)	523(2)	2168(1)	20(1)
C(00J)	3500(3)	3588(2)	5106(1)	21(1)
C(00K)	4724(3)	4015(2)	5866(2)	26(1)
C(00L)	5796(3)	-222(2)	2416(2)	24(1)
C(00M)	934(3)	3503(2)	4370(2)	25(1)
C(00N)	963(3)	4746(2)	7147(2)	24(1)
C(00O)	734(3)	6061(2)	8763(2)	30(1)
C(00P)	-1033(3)	-390(3)	909(2)	30(1)
C(00Q)	2543(2)	1511(2)	2684(1)	19(1)
C(00R)	4244(3)	3548(2)	1072(2)	26(1)
C(00S)	-1435(3)	-1389(2)	1240(2)	33(1)
C(00T)	3708(3)	4174(2)	1731(2)	25(1)
C(00U)	2090(3)	3870(2)	5109(2)	23(1)
C(00V)	-224(3)	4895(3)	8315(2)	31(1)
C(00W)	5098(3)	2610(2)	4290(2)	24(1)
C(00X)	1020(3)	3948(2)	6280(2)	28(1)

C(00Y)	-109(3)	4239(2)	7501(2)	29(1)
C(00Z)	6079(3)	3744(2)	5827(2)	30(1)
C(010)	7171(3)	576(2)	2864(2)	29(1)
C(011)	6269(3)	3046(2)	5034(2)	29(1)
C(012)	678(3)	-485(2)	2491(2)	32(1)
C(013)	1807(3)	6590(2)	8426(2)	32(1)
C(014)	1921(3)	5924(2)	7611(2)	29(1)
C(015)	5048(3)	-964(3)	2827(2)	33(1)
C(016)	7805(3)	636(3)	3730(2)	33(1)
C(017)	7086(3)	-93(2)	4156(2)	33(1)
C(018)	-600(3)	-1423(3)	2023(2)	38(1)
C(019)	7781(4)	-50(3)	5087(2)	47(1)
C(01A)	5698(3)	-880(3)	3696(2)	37(1)
C(01B)	4418(5)	5561(3)	1041(2)	55(1)

Table S22. Bond lengths [Å] and angles [°] for **7**.

Br(01)-C(00O)	1.899(2)
S(002)-O(003)	1.4271(18)
S(002)-O(005)	1.4309(18)
S(002)-N(008)	1.6301(19)
S(002)-C(00L)	1.764(2)
O(004)-C(00U)	1.411(3)
O(004)-C(00X)	1.362(3)
O(006)-C(00R)	1.366(3)
O(006)-C(01B)	1.425(3)
O(007)-C(00T)	1.379(3)
O(007)-C(01B)	1.425(3)
O(009)-C(00X)	1.190(3)
N(008)-C(00C)	1.429(3)
C(00A)-C(00C)	1.400(3)
C(00A)-C(00D)	1.403(3)
C(00A)-C(00Q)	1.525(3)
C(00B)-C(00E)	1.370(3)
C(00B)-C(00G)	1.433(3)
C(00B)-C(00Q)	1.525(3)
C(00C)-C(00F)	1.399(3)
C(00D)-C(00T)	1.374(3)
C(00E)-C(00M)	1.415(3)
C(00F)-C(00R)	1.367(3)
C(00G)-C(00J)	1.430(3)
C(00G)-C(00W)	1.421(3)
C(00H)-C(00I)	1.379(3)
C(00H)-C(00P)	1.393(3)
C(00I)-C(00Q)	1.528(3)
C(00I)-C(012)	1.391(3)
C(00J)-C(00K)	1.412(3)
C(00J)-C(00U)	1.418(3)
C(00K)-C(00Z)	1.373(4)
C(00L)-C(010)	1.382(4)
C(00L)-C(015)	1.393(3)
C(00M)-C(00U)	1.356(3)
C(00N)-C(00X)	1.490(3)

C(00N)-C(00Y)	1.392(3)
C(00N)-C(014)	1.393(4)
C(00O)-C(00V)	1.379(4)
C(00O)-C(013)	1.384(4)
C(00P)-C(00S)	1.385(4)
C(00R)-C(00T)	1.373(3)
C(00S)-C(018)	1.354(4)
C(00V)-C(00Y)	1.385(3)
C(00W)-C(011)	1.368(3)
C(00Z)-C(011)	1.401(4)
C(010)-C(016)	1.386(4)
C(012)-C(018)	1.391(4)
C(013)-C(014)	1.390(3)
C(015)-C(01A)	1.385(4)
C(016)-C(017)	1.386(4)
C(017)-C(019)	1.499(4)
C(017)-C(01A)	1.391(4)
O(003)-S(002)-O(005)	120.71(11)
O(003)-S(002)-N(008)	107.50(10)
O(003)-S(002)-C(00L)	107.67(11)
O(005)-S(002)-N(008)	105.11(10)
O(005)-S(002)-C(00L)	107.32(11)
N(008)-S(002)-C(00L)	107.97(10)
C(00X)-O(004)-C(00U)	115.82(18)
C(00R)-O(006)-C(01B)	105.49(19)
C(00T)-O(007)-C(01B)	105.09(19)
C(00C)-N(008)-S(002)	123.37(15)
C(00C)-C(00A)-C(00D)	118.93(19)
C(00C)-C(00A)-C(00Q)	119.90(19)
C(00D)-C(00A)-C(00Q)	121.12(19)
C(00E)-C(00B)-C(00G)	119.0(2)
C(00E)-C(00B)-C(00Q)	120.80(19)
C(00G)-C(00B)-C(00Q)	120.24(19)
C(00A)-C(00C)-N(008)	119.89(19)
C(00F)-C(00C)-N(008)	117.62(19)
C(00F)-C(00C)-C(00A)	122.4(2)
C(00T)-C(00D)-C(00A)	117.9(2)

C(00B)-C(00E)-C(00M)	122.0(2)
C(00R)-C(00F)-C(00C)	116.6(2)
C(00J)-C(00G)-C(00B)	119.5(2)
C(00W)-C(00G)-C(00B)	122.9(2)
C(00W)-C(00G)-C(00J)	117.6(2)
C(00I)-C(00H)-C(00P)	120.8(2)
C(00H)-C(00I)-C(00Q)	122.6(2)
C(00H)-C(00I)-C(012)	118.4(2)
C(012)-C(00I)-C(00Q)	119.0(2)
C(00K)-C(00J)-C(00G)	119.7(2)
C(00K)-C(00J)-C(00U)	122.5(2)
C(00U)-C(00J)-C(00G)	117.8(2)
C(00Z)-C(00K)-C(00J)	120.5(2)
C(010)-C(00L)-S(002)	120.12(18)
C(010)-C(00L)-C(015)	120.7(2)
C(015)-C(00L)-S(002)	119.14(19)
C(00U)-C(00M)-C(00E)	119.1(2)
C(00Y)-C(00N)-C(00X)	116.2(2)
C(00Y)-C(00N)-C(014)	119.8(2)
C(014)-C(00N)-C(00X)	124.0(2)
C(00V)-C(00O)-Br(01)	117.53(19)
C(00V)-C(00O)-C(013)	122.0(2)
C(013)-C(00O)-Br(01)	120.5(2)
C(00S)-C(00P)-C(00H)	120.0(2)
C(00A)-C(00Q)-C(00B)	112.35(17)
C(00A)-C(00Q)-C(00I)	112.87(17)
C(00B)-C(00Q)-C(00I)	112.56(17)
O(006)-C(00R)-C(00F)	127.5(2)
O(006)-C(00R)-C(00T)	110.3(2)
C(00F)-C(00R)-C(00T)	122.1(2)
C(018)-C(00S)-C(00P)	119.6(2)
C(00D)-C(00T)-O(007)	128.0(2)
C(00R)-C(00T)-O(007)	110.0(2)
C(00R)-C(00T)-C(00D)	122.0(2)
O(004)-C(00U)-C(00J)	117.2(2)
C(00M)-C(00U)-O(004)	120.5(2)
C(00M)-C(00U)-C(00J)	122.3(2)
C(00O)-C(00V)-C(00Y)	118.9(2)

C(011)-C(00W)-C(00G)	121.5(2)
O(004)-C(00X)-C(00N)	112.6(2)
O(009)-C(00X)-O(004)	123.3(2)
O(009)-C(00X)-C(00N)	124.1(2)
C(00V)-C(00Y)-C(00N)	120.4(2)
C(00K)-C(00Z)-C(011)	120.5(2)
C(00L)-C(010)-C(016)	119.4(2)
C(00W)-C(011)-C(00Z)	120.2(2)
C(018)-C(012)-C(00I)	120.4(2)
C(00O)-C(013)-C(014)	118.8(2)
C(013)-C(014)-C(00N)	120.0(2)
C(01A)-C(015)-C(00L)	118.8(2)
C(010)-C(016)-C(017)	121.1(2)
C(016)-C(017)-C(019)	121.3(3)
C(016)-C(017)-C(01A)	118.5(2)
C(01A)-C(017)-C(019)	120.2(2)
C(00S)-C(018)-C(012)	120.9(2)
C(015)-C(01A)-C(017)	121.4(2)
O(006)-C(01B)-O(007)	108.9(2)

Symmetry transformations used to generate equivalent atoms:

Table S23. Anisotropic displacement parameters ($Å^2$x 10^3) for **7**. The anisotropic displacement factor exponent takes the form: $-2p^2[h^2 a^{*2}U^{11} + ... + 2 h k a^* b^* U^{12}]$

	U^{11}	U^{22}	U^{33}	U^{23}	U^{13}	U^{12}
Br(01)	67(1)	42(1)	27(1)	8(1)	24(1)	22(1)
S(002)	32(1)	20(1)	26(1)	9(1)	15(1)	13(1)
O(003)	34(1)	35(1)	36(1)	19(1)	22(1)	17(1)
O(004)	32(1)	23(1)	22(1)	1(1)	14(1)	4(1)
O(005)	49(1)	22(1)	32(1)	6(1)	16(1)	16(1)
O(006)	67(1)	27(1)	47(1)	22(1)	40(1)	21(1)
O(007)	65(1)	20(1)	44(1)	15(1)	34(1)	15(1)
O(009)	84(2)	32(1)	40(1)	-5(1)	36(1)	-15(1)
N(008)	26(1)	18(1)	24(1)	7(1)	13(1)	8(1)
C(00A)	20(1)	18(1)	18(1)	6(1)	6(1)	5(1)
C(00B)	26(1)	17(1)	18(1)	6(1)	9(1)	5(1)
C(00C)	21(1)	18(1)	19(1)	6(1)	6(1)	7(1)
C(00D)	30(1)	19(1)	20(1)	4(1)	12(1)	7(1)
C(00E)	23(1)	26(1)	20(1)	6(1)	6(1)	7(1)
C(00F)	29(1)	23(1)	20(1)	7(1)	12(1)	11(1)
C(00G)	24(1)	15(1)	18(1)	6(1)	7(1)	3(1)
C(00H)	25(1)	30(1)	23(1)	11(1)	10(1)	8(1)
C(00I)	24(1)	18(1)	19(1)	4(1)	11(1)	6(1)
C(00J)	28(1)	16(1)	20(1)	6(1)	9(1)	3(1)
C(00K)	35(1)	20(1)	21(1)	4(1)	6(1)	5(1)
C(00L)	30(1)	22(1)	28(1)	12(1)	14(1)	13(1)
C(00M)	26(1)	26(1)	25(1)	6(1)	11(1)	10(1)
C(00N)	30(1)	25(1)	21(1)	7(1)	10(1)	10(1)
C(00O)	43(1)	32(1)	21(1)	8(1)	13(1)	20(1)
C(00P)	26(1)	42(2)	20(1)	4(1)	6(1)	8(1)
C(00Q)	23(1)	19(1)	17(1)	6(1)	8(1)	7(1)
C(00R)	32(1)	26(1)	26(1)	14(1)	15(1)	8(1)
C(00S)	24(1)	29(1)	36(1)	-6(1)	8(1)	1(1)
C(00T)	35(1)	15(1)	28(1)	6(1)	13(1)	7(1)
C(00U)	31(1)	18(1)	21(1)	3(1)	12(1)	5(1)
C(00V)	37(1)	35(1)	30(1)	15(1)	18(1)	15(1)
C(00W)	25(1)	21(1)	25(1)	5(1)	9(1)	5(1)

C(00X)	36(1)	27(1)	23(1)	5(1)	12(1)	6(1)
C(00Y)	33(1)	28(1)	27(1)	9(1)	10(1)	8(1)
C(00Z)	29(1)	24(1)	28(1)	2(1)	-3(1)	4(1)
C(010)	30(1)	29(1)	36(1)	15(1)	15(1)	8(1)
C(011)	23(1)	23(1)	36(1)	5(1)	6(1)	5(1)
C(012)	34(1)	30(1)	32(1)	16(1)	4(1)	4(1)
C(013)	38(1)	28(1)	28(1)	3(1)	11(1)	8(1)
C(014)	31(1)	28(1)	28(1)	5(1)	13(1)	7(1)
C(015)	36(1)	28(1)	36(1)	15(1)	10(1)	0(1)
C(016)	30(1)	34(1)	37(1)	13(1)	10(1)	6(1)
C(017)	44(2)	29(1)	33(1)	16(1)	11(1)	13(1)
C(018)	36(1)	23(1)	55(2)	17(1)	10(1)	2(1)
C(019)	59(2)	44(2)	35(2)	22(1)	2(1)	3(1)
C(01A)	46(2)	33(1)	38(1)	21(1)	14(1)	2(1)
C(01B)	100(3)	29(2)	70(2)	28(2)	64(2)	28(2)

Table S24. Hydrogen coordinates (x 10^4) and isotropic displacement parameters ($Å^2$x 10^3) for **7**.

	x	y	z	U(eq)
H(008)	2976	-41	1503	25
H(00D)	2750	4002	2703	27
H(00E)	286	2466	3067	27
H(00F)	4586	1840	430	27
H(00H)	485	1264	1159	29
H(00K)	4608	4492	6407	32
H(00M)	23	3756	4383	30
H(00P)	-1616	-358	361	37
H(00Q)	3299	1017	2864	22
H(00S)	-2291	-2046	919	39
H(00V)	-950	4549	8560	37
H(00W)	5244	2134	3758	28
H(00Y)	-766	3437	7183	35
H(00Z)	6893	4033	6343	36
H(010)	7676	1079	2580	35
H(011)	7214	2875	5012	35
H(012)	1262	-533	3034	38
H(013)	2455	7395	8746	39
H(014)	2654	6272	7370	35
H(015)	4108	-1518	2517	40
H(016)	8749	1185	4038	39
H(018)	-890	-2097	2256	46
H(01A)	8190	-811	5093	71
H(01B)	7028	-28	5411	71
H(01C)	8581	717	5367	71
H(01D)	5185	-1371	3984	45
H(01E)	3686	5798	604	66
H(01F)	5338	6240	1257	66

Table S25. Torsion angles [°] for **7**.

Br(01)-C(00O)-C(00V)-C(00Y)	178.44(19)
Br(01)-C(00O)-C(013)-C(014)	-178.70(19)
S(002)-N(008)-C(00C)-C(00A)	-118.2(2)
S(002)-N(008)-C(00C)-C(00F)	65.6(3)
S(002)-C(00L)-C(010)-C(016)	-177.22(19)
S(002)-C(00L)-C(015)-C(01A)	177.8(2)
O(003)-S(002)-N(008)-C(00C)	-37.3(2)
O(003)-S(002)-C(00L)-C(010)	4.4(2)
O(003)-S(002)-C(00L)-C(015)	-172.80(19)
O(005)-S(002)-N(008)-C(00C)	-167.08(17)
O(005)-S(002)-C(00L)-C(010)	135.76(19)
O(005)-S(002)-C(00L)-C(015)	-41.4(2)
O(006)-C(00R)-C(00T)-O(007)	-0.3(3)
O(006)-C(00R)-C(00T)-C(00D)	179.6(2)
N(008)-S(002)-C(00L)-C(010)	-111.4(2)
N(008)-S(002)-C(00L)-C(015)	71.4(2)
N(008)-C(00C)-C(00F)-C(00R)	176.2(2)
C(00A)-C(00C)-C(00F)-C(00R)	0.1(3)
C(00A)-C(00D)-C(00T)-O(007)	-178.6(2)
C(00A)-C(00D)-C(00T)-C(00R)	1.5(4)
C(00B)-C(00E)-C(00M)-C(00U)	-1.5(4)
C(00B)-C(00G)-C(00J)-C(00K)	177.9(2)
C(00B)-C(00G)-C(00J)-C(00U)	-2.6(3)
C(00B)-C(00G)-C(00W)-C(011)	-178.5(2)
C(00C)-C(00A)-C(00D)-C(00T)	-2.7(3)
C(00C)-C(00A)-C(00Q)-C(00B)	164.70(19)
C(00C)-C(00A)-C(00Q)-C(00I)	-66.7(3)
C(00C)-C(00F)-C(00R)-O(006)	179.8(2)
C(00C)-C(00F)-C(00R)-C(00T)	-1.4(4)
C(00D)-C(00A)-C(00C)-N(008)	-174.0(2)
C(00D)-C(00A)-C(00C)-C(00F)	2.0(3)
C(00D)-C(00A)-C(00Q)-C(00B)	-17.9(3)
C(00D)-C(00A)-C(00Q)-C(00I)	110.6(2)
C(00E)-C(00B)-C(00G)-C(00J)	5.3(3)
C(00E)-C(00B)-C(00G)-C(00W)	-175.2(2)
C(00E)-C(00B)-C(00Q)-C(00A)	94.3(2)

C(00E)-C(00B)-C(00Q)-C(00I)	-34.4(3)
C(00E)-C(00M)-C(00U)-O(004)	-177.1(2)
C(00E)-C(00M)-C(00U)-C(00J)	4.4(4)
C(00F)-C(00R)-C(00T)-O(007)	-179.3(2)
C(00F)-C(00R)-C(00T)-C(00D)	0.6(4)
C(00G)-C(00B)-C(00E)-C(00M)	-3.3(3)
C(00G)-C(00B)-C(00Q)-C(00A)	-85.0(2)
C(00G)-C(00B)-C(00Q)-C(00I)	146.2(2)
C(00G)-C(00J)-C(00K)-C(00Z)	1.1(3)
C(00G)-C(00J)-C(00U)-O(004)	179.16(18)
C(00G)-C(00J)-C(00U)-C(00M)	-2.3(3)
C(00G)-C(00W)-C(011)-C(00Z)	0.3(4)
C(00H)-C(00I)-C(00Q)-C(00A)	-18.6(3)
C(00H)-C(00I)-C(00Q)-C(00B)	109.9(2)
C(00H)-C(00I)-C(012)-C(018)	0.5(4)
C(00H)-C(00P)-C(00S)-C(018)	0.2(4)
C(00I)-C(00H)-C(00P)-C(00S)	1.5(4)
C(00I)-C(012)-C(018)-C(00S)	1.3(4)
C(00J)-C(00G)-C(00W)-C(011)	1.0(3)
C(00J)-C(00K)-C(00Z)-C(011)	0.2(4)
C(00K)-C(00J)-C(00U)-O(004)	-1.3(3)
C(00K)-C(00J)-C(00U)-C(00M)	177.3(2)
C(00K)-C(00Z)-C(011)-C(00W)	-0.9(4)
C(00L)-S(002)-N(008)-C(00C)	78.61(19)
C(00L)-C(010)-C(016)-C(017)	0.1(4)
C(00L)-C(015)-C(01A)-C(017)	-1.2(4)
C(00O)-C(00V)-C(00Y)-C(00N)	0.5(4)
C(00O)-C(013)-C(014)-C(00N)	0.2(4)
C(00P)-C(00H)-C(00I)-C(00Q)	176.5(2)
C(00P)-C(00H)-C(00I)-C(012)	-1.9(3)
C(00P)-C(00S)-C(018)-C(012)	-1.6(4)
C(00Q)-C(00A)-C(00C)-N(008)	3.4(3)
C(00Q)-C(00A)-C(00C)-C(00F)	179.4(2)
C(00Q)-C(00A)-C(00D)-C(00T)	179.9(2)
C(00Q)-C(00B)-C(00E)-C(00M)	177.3(2)
C(00Q)-C(00B)-C(00G)-C(00J)	-175.31(19)
C(00Q)-C(00B)-C(00G)-C(00W)	4.2(3)
C(00Q)-C(00I)-C(012)-C(018)	-177.9(2)

C(00R)-O(006)-C(01B)-O(007)	4.3(4)
C(00T)-O(007)-C(01B)-O(006)	-4.4(4)
C(00U)-O(004)-C(00X)-O(009)	-3.1(4)
C(00U)-O(004)-C(00X)-C(00N)	177.60(19)
C(00U)-C(00J)-C(00K)-C(00Z)	-178.4(2)
C(00V)-C(00O)-C(013)-C(014)	0.0(4)
C(00W)-C(00G)-C(00J)-C(00K)	-1.7(3)
C(00W)-C(00G)-C(00J)-C(00U)	177.9(2)
C(00X)-O(004)-C(00U)-C(00J)	-112.6(2)
C(00X)-O(004)-C(00U)-C(00M)	68.8(3)
C(00X)-C(00N)-C(00Y)-C(00V)	178.4(2)
C(00X)-C(00N)-C(014)-C(013)	-178.6(2)
C(00Y)-C(00N)-C(00X)-O(004)	170.8(2)
C(00Y)-C(00N)-C(00X)-O(009)	-8.5(4)
C(00Y)-C(00N)-C(014)-C(013)	0.0(4)
C(010)-C(00L)-C(015)-C(01A)	0.6(4)
C(010)-C(016)-C(017)-C(019)	178.8(3)
C(010)-C(016)-C(017)-C(01A)	-0.7(4)
C(012)-C(00I)-C(00Q)-C(00A)	159.8(2)
C(012)-C(00I)-C(00Q)-C(00B)	-71.7(3)
C(013)-C(00O)-C(00V)-C(00Y)	-0.3(4)
C(014)-C(00N)-C(00X)-O(004)	-10.5(3)
C(014)-C(00N)-C(00X)-O(009)	170.2(3)
C(014)-C(00N)-C(00Y)-C(00V)	-0.3(4)
C(015)-C(00L)-C(010)-C(016)	-0.1(4)
C(016)-C(017)-C(01A)-C(015)	1.2(4)
C(019)-C(017)-C(01A)-C(015)	-178.2(3)
C(01B)-O(006)-C(00R)-C(00F)	176.4(3)
C(01B)-O(006)-C(00R)-C(00T)	-2.5(3)
C(01B)-O(007)-C(00T)-C(00D)	-176.9(3)
C(01B)-O(007)-C(00T)-C(00R)	2.9(3)

Symmetry transformations used to generate equivalent atoms:

8. Copies of NMR Spectra of Products

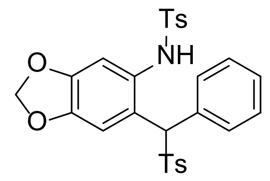

1a

(^{1}H NMR, 400MHz, $CDCl_3$)

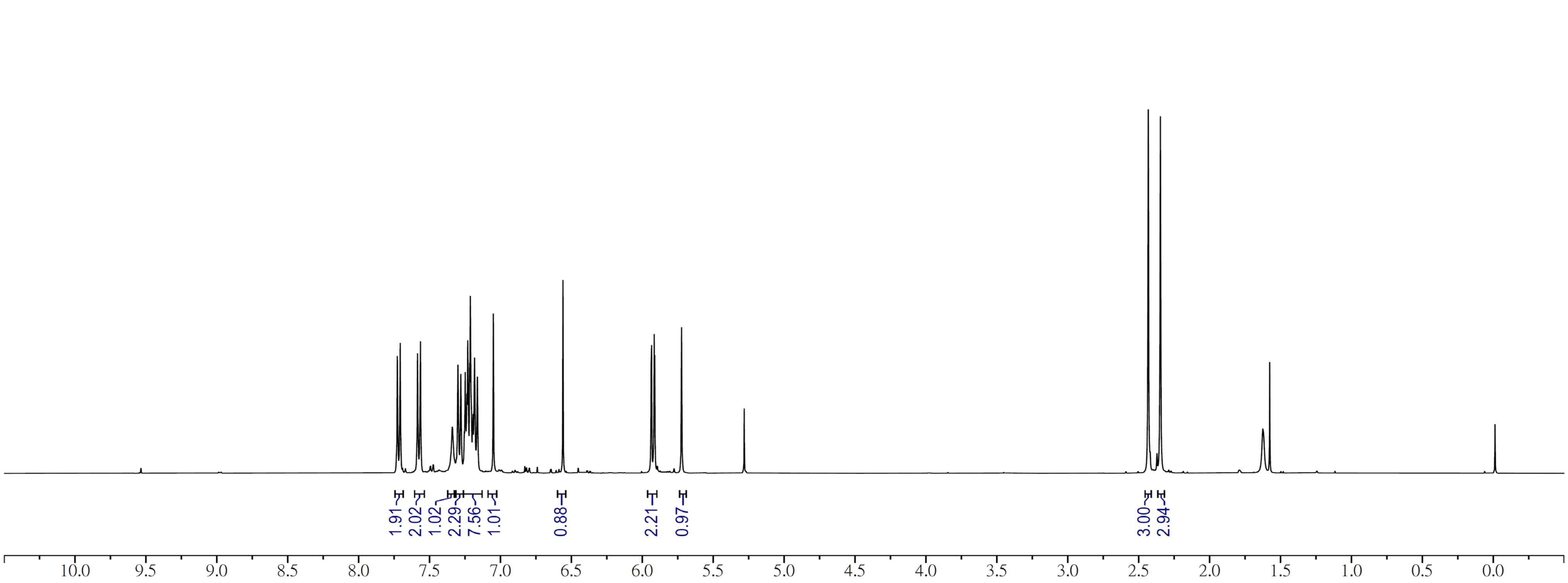

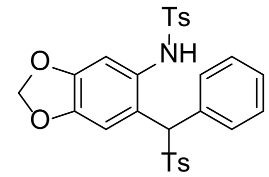

1a

($^{13}C\{^1H\}$ NMR, 101 MHz, $CDCl_3$)

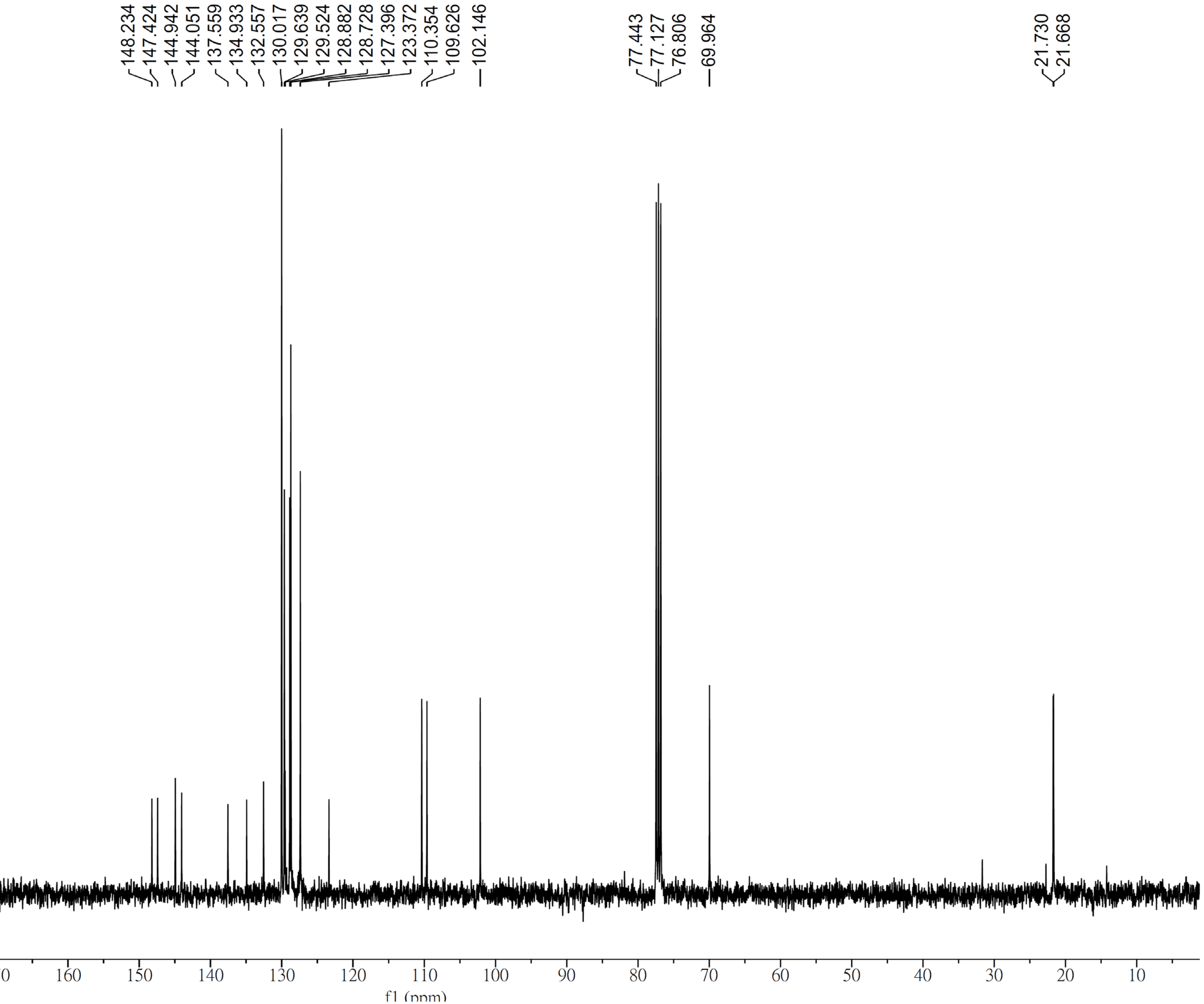

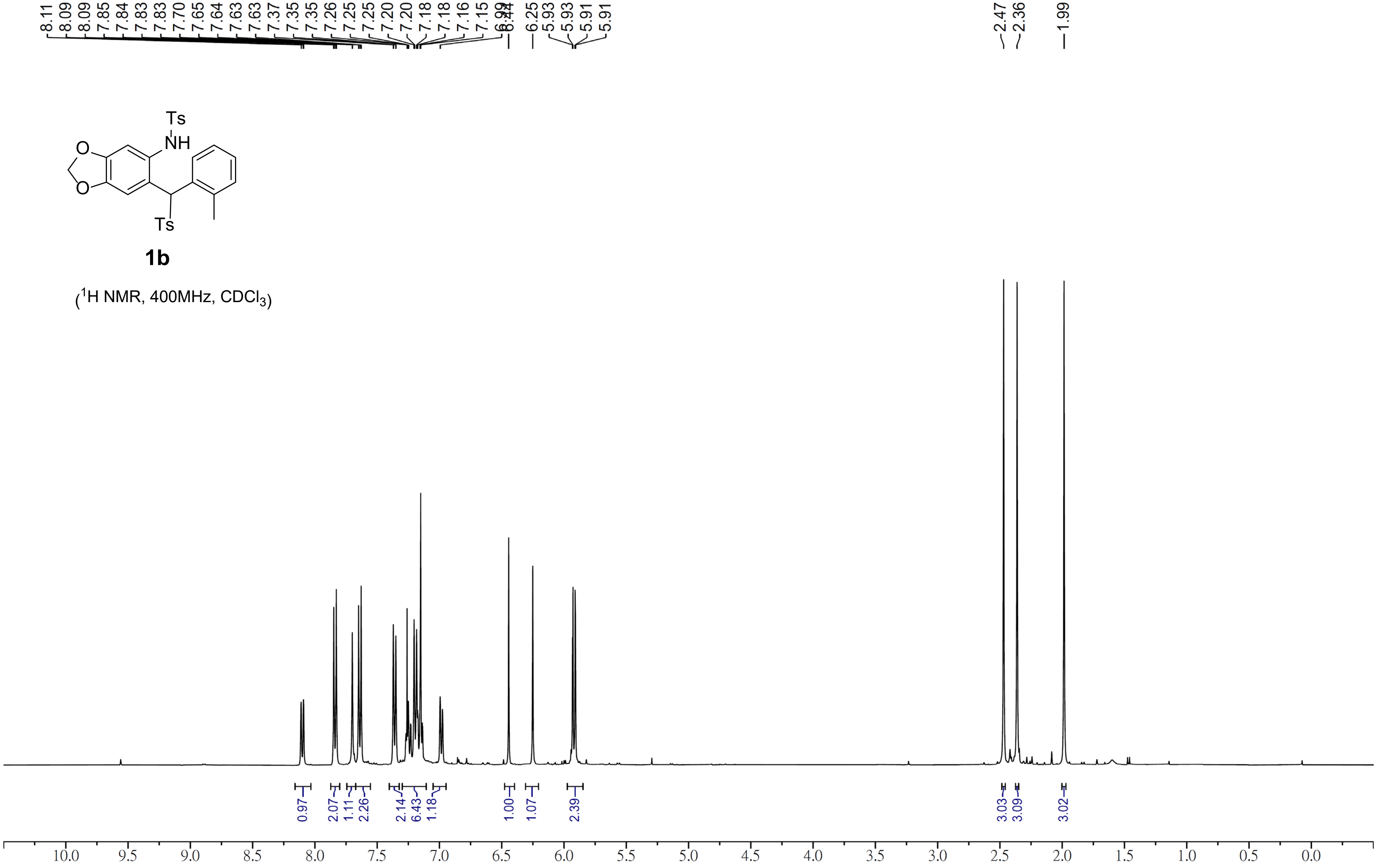

1b

(^{1}H NMR, 400MHz, $CDCl_3$)

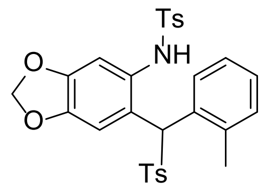

1b

($^{13}C\{^{1}H\}$ NMR, 101 MHz, $CDCl_3$)

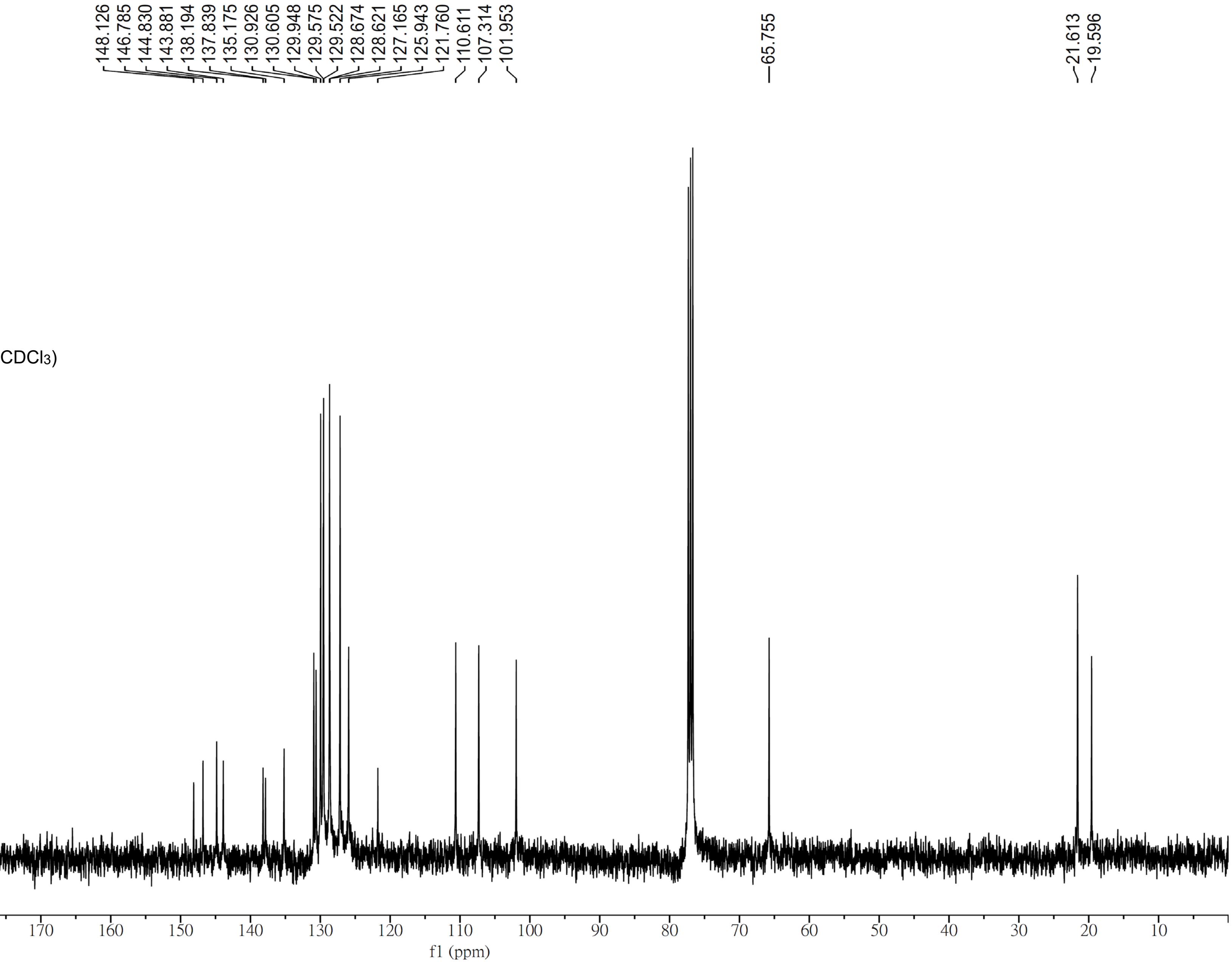

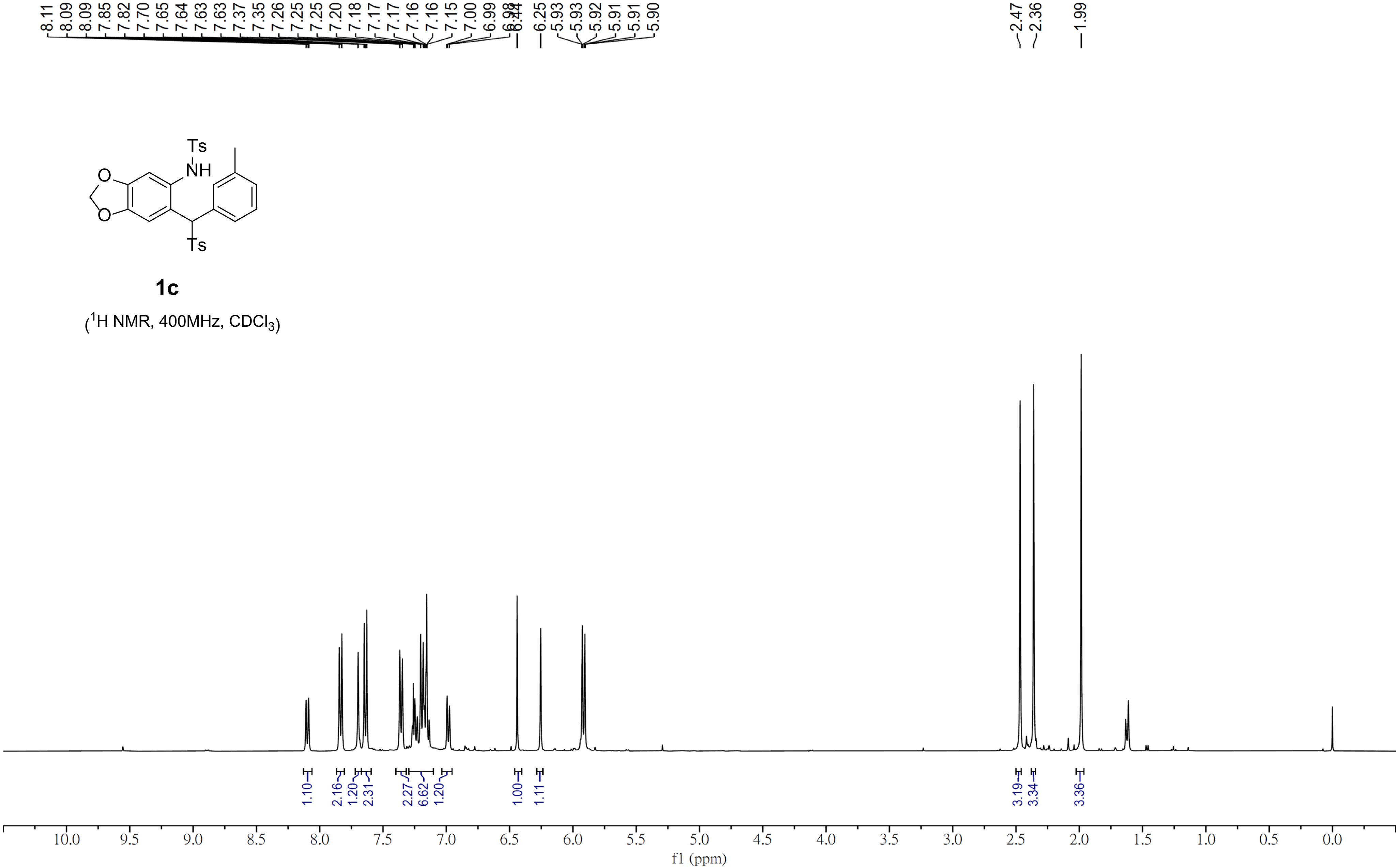

8.11
8.09
8.09
7.85
7.82
7.70
7.65
7.64
7.63
7.63
7.37
7.35
7.26
7.25
7.25
7.20
7.18
7.17
7.17
7.16
7.16
7.15
7.00
6.99
6.98
6.44
6.25
5.93
5.93
5.92
5.91
5.91
5.90
2.47
2.36
1.99
Ts
NH
O
O
Ts
1c
(¹H NMR, 400MHz, CDCl3)
1.10
2.16
1.20
2.31
2.27
6.62
1.20
1.00
1.11
3.19
3.34
3.36
10.0
9.5
9.0
8.5
8.0
7.5
7.0
6.5
6.0
5.5
5.0
4.5
4.0
3.5
3.0
2.5
2.0
1.5
1.0
0.5
0.0
f1 (ppm)

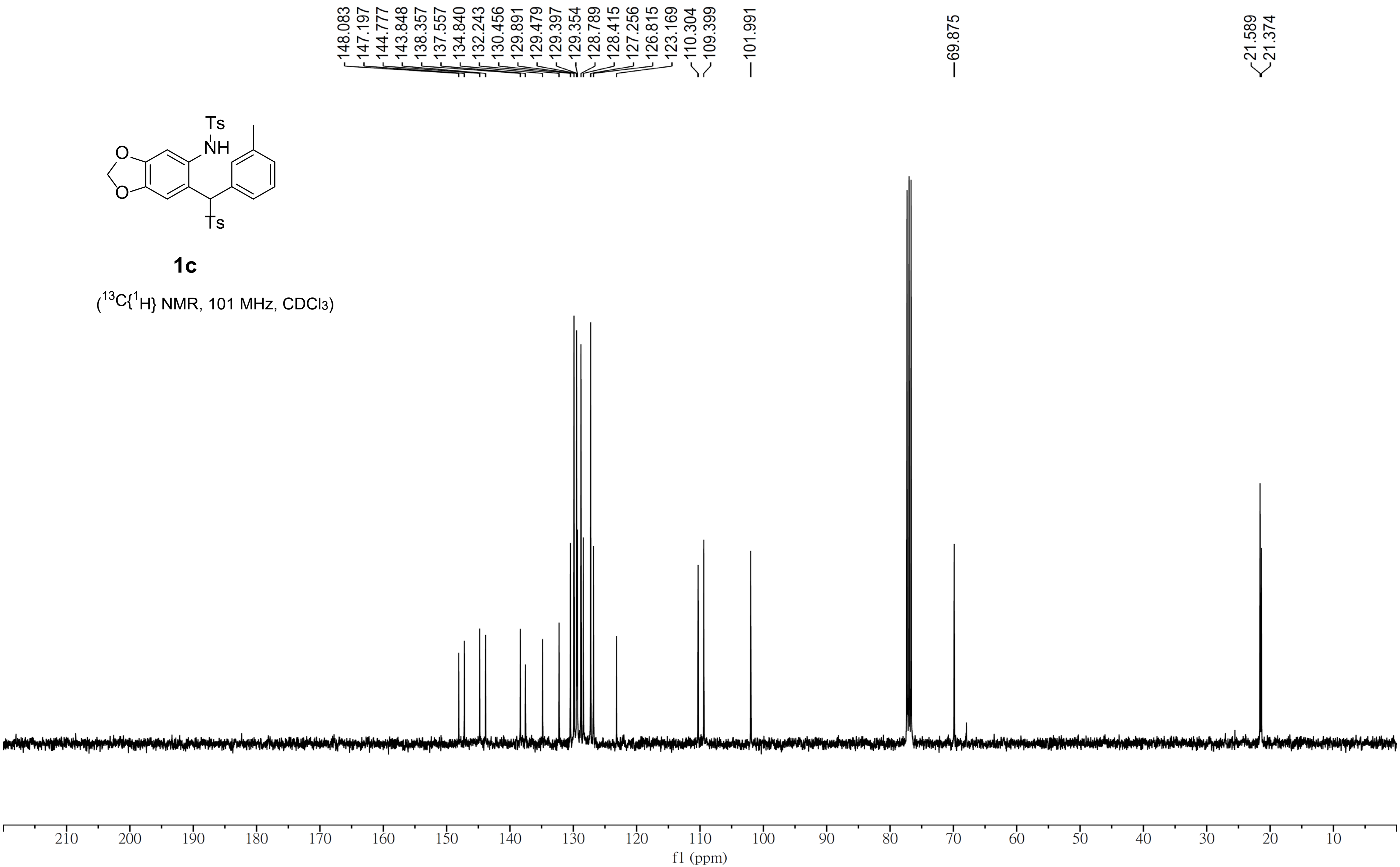

1c

($^{13}C\{^{1}H\}$ NMR, 101 MHz, $CDCl_3$)

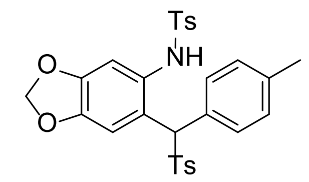

1d

(^{1}H NMR, 400MHz, $CDCl_3$)

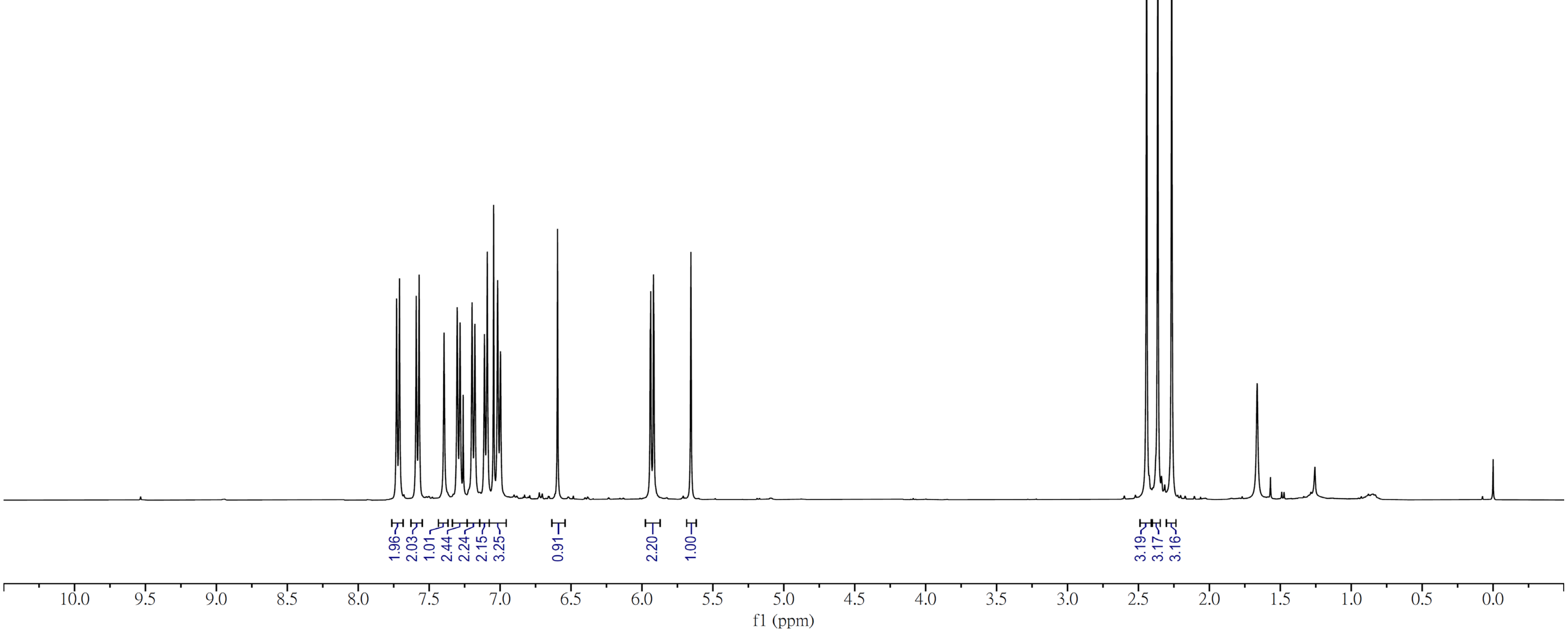

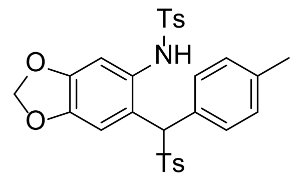

1d

($^{13}C\{^{1}H\}$ NMR, 101 MHz, CDCl₃)

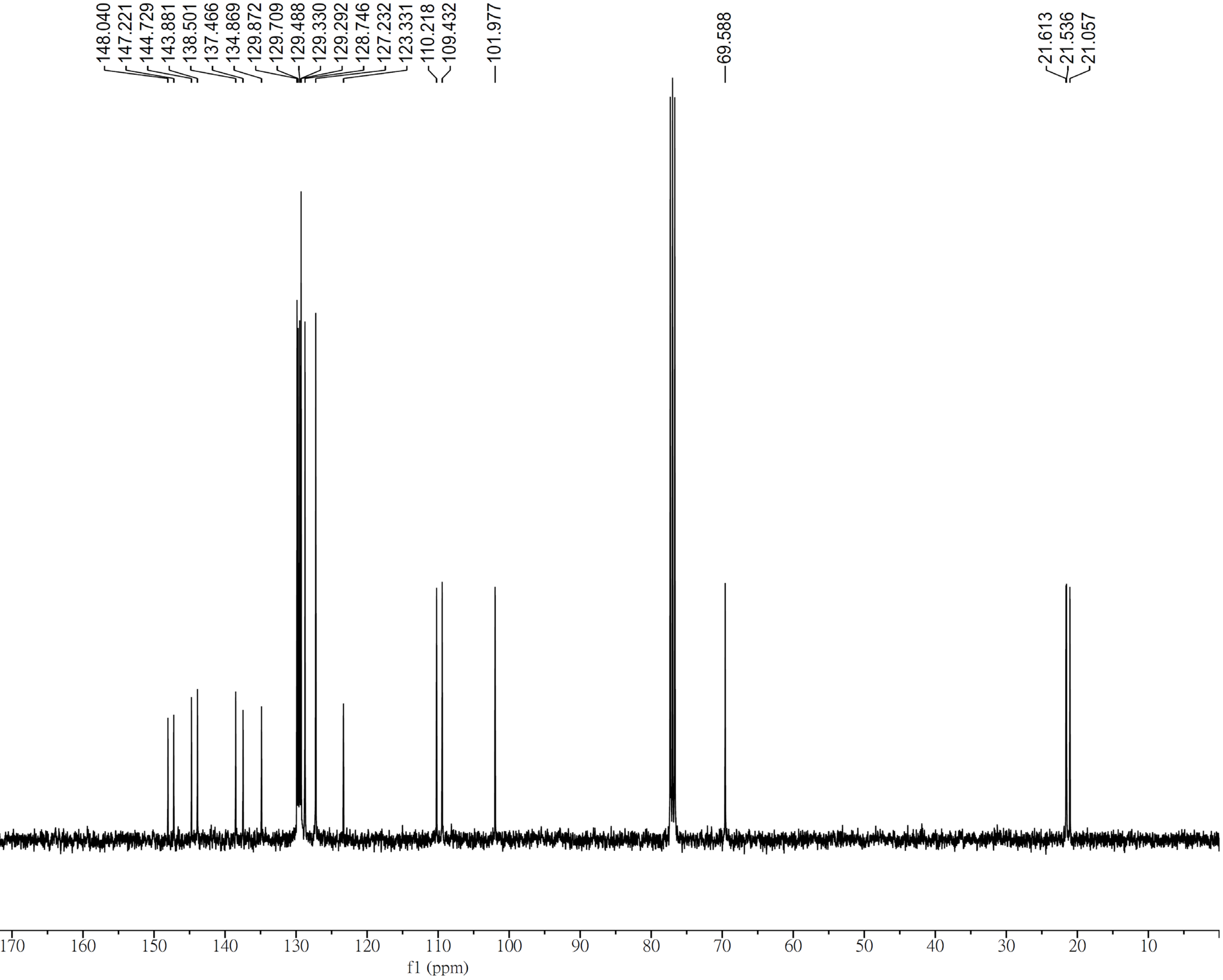

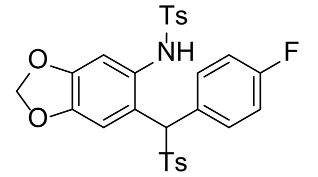

1e

(^{1}H NMR, 400MHz, $CDCl_3$)

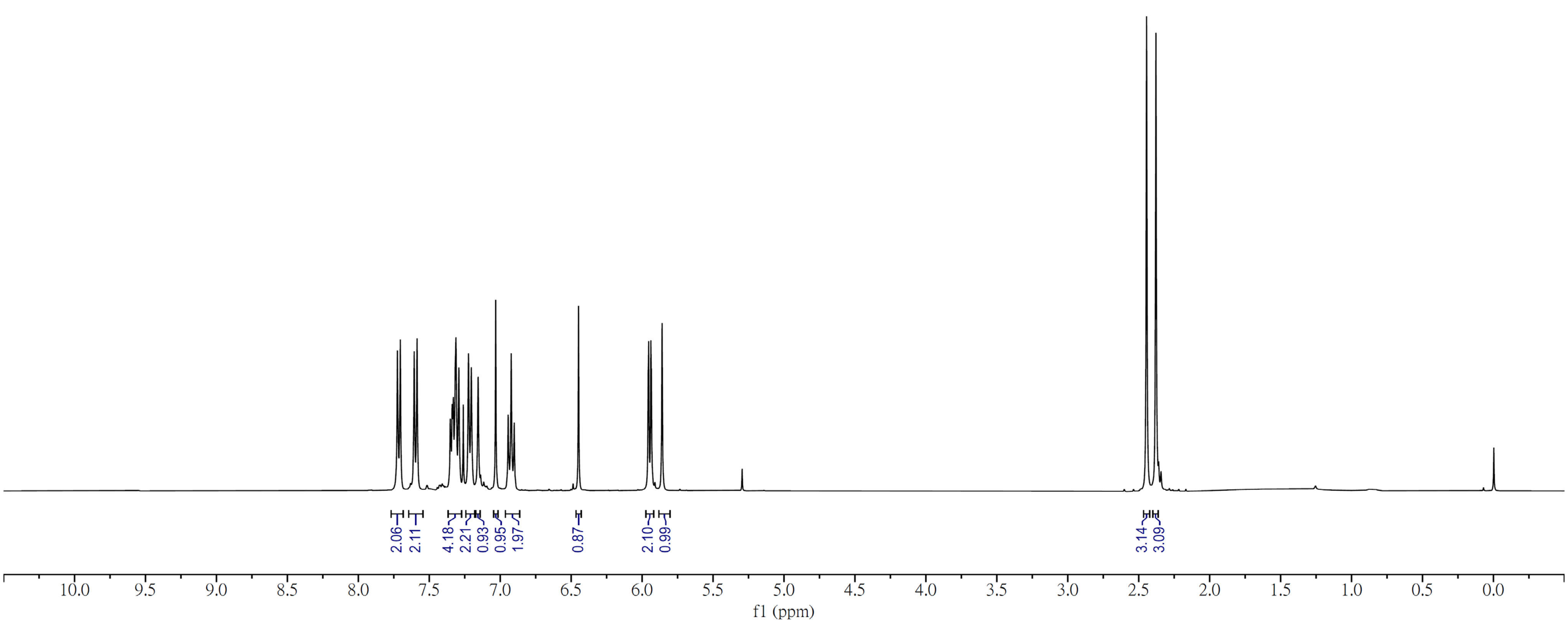

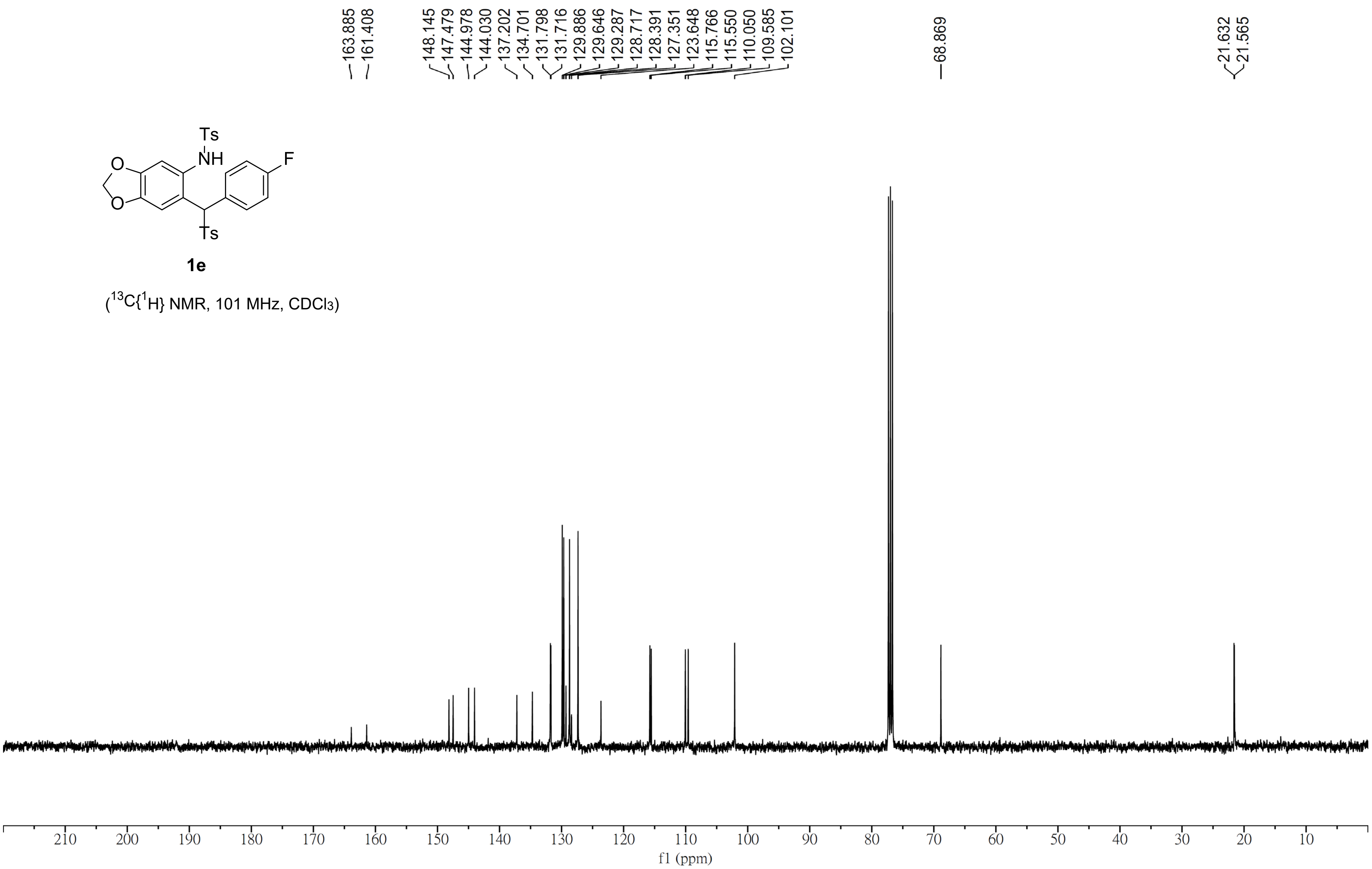

1e

($^{13}C\{^1H\}$ NMR, 101 MHz, $CDCl_3$)

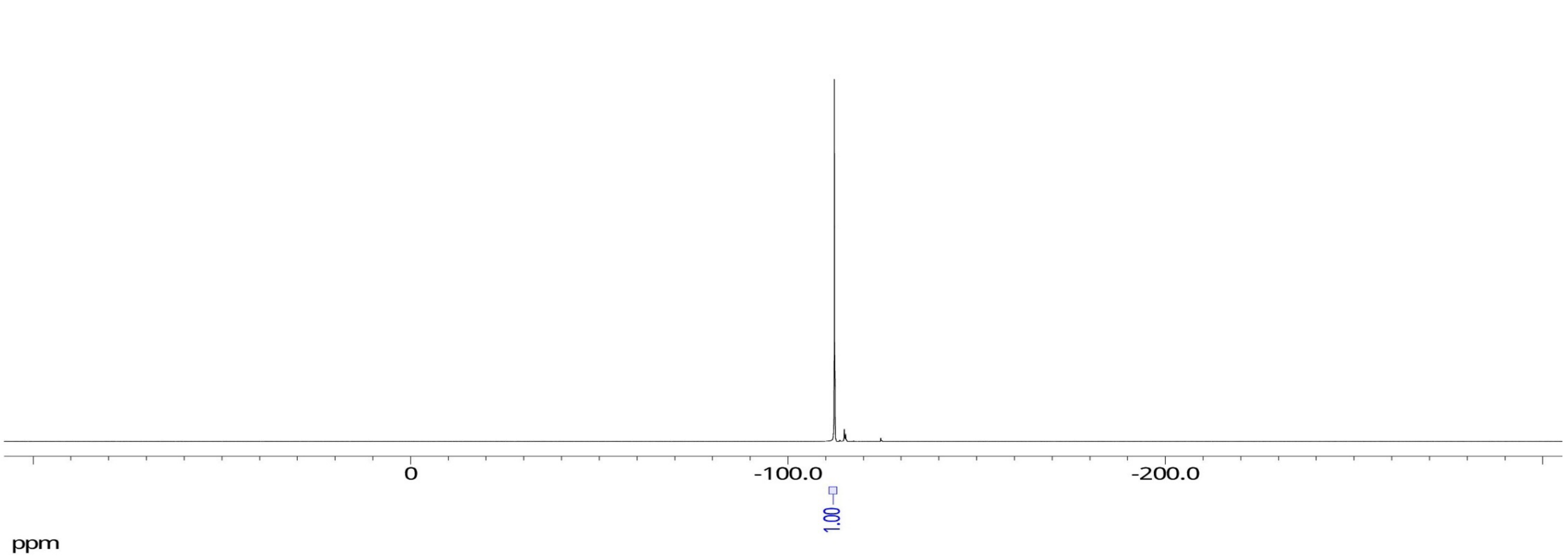

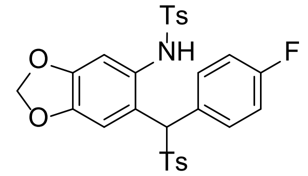

1e

(^{19}F NMR, 376MHz, $CDCl_3$)

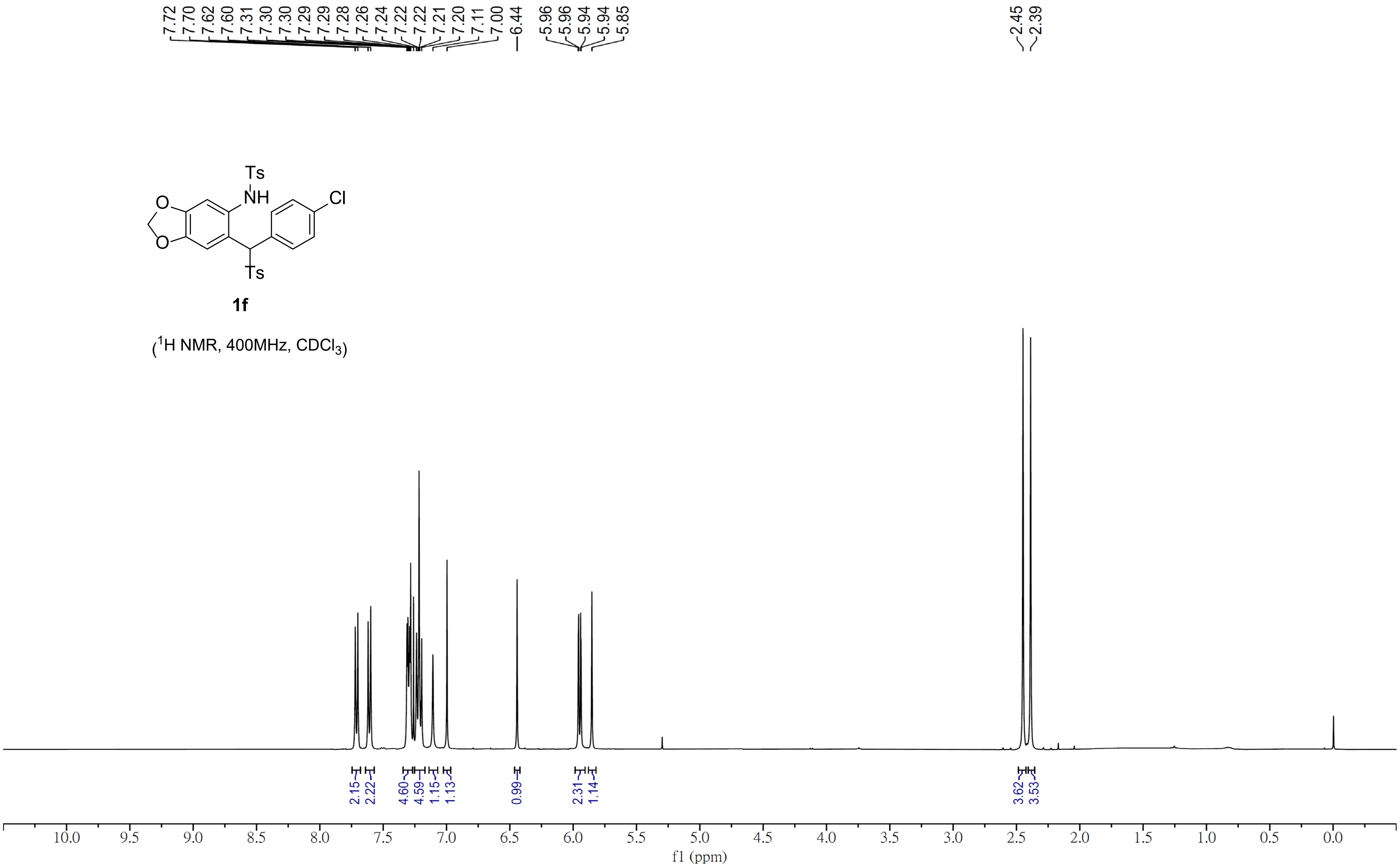

1f

(^{1}H NMR, 400MHz, $CDCl_3$)

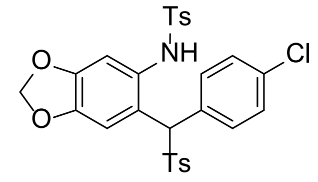

1f

($^{13}C\{^{1}H\}$ NMR, 101 MHz, $CDCl_3$)

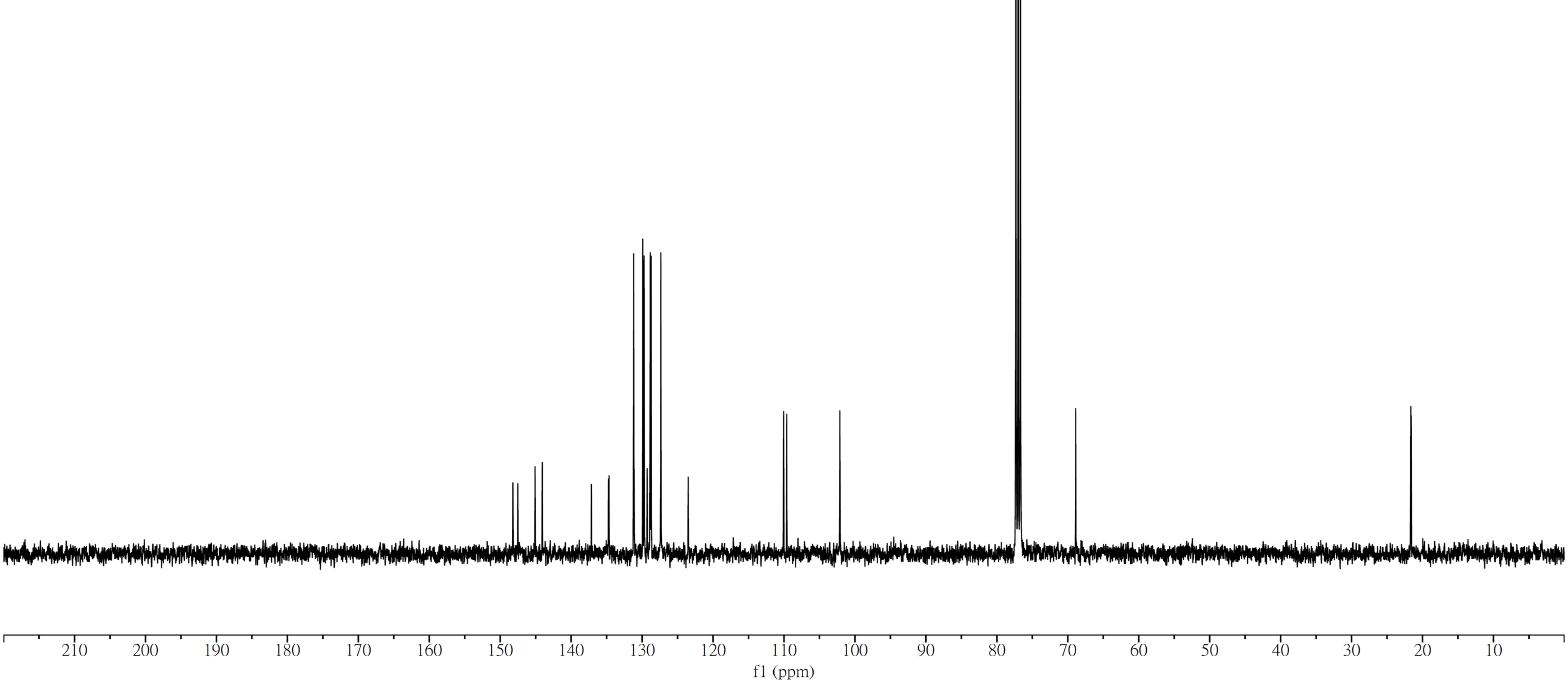

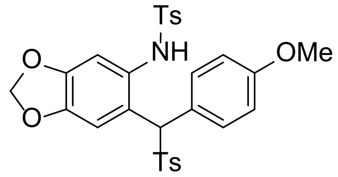

1g

(^{1}H NMR, 400MHz, CDCl$_3$)

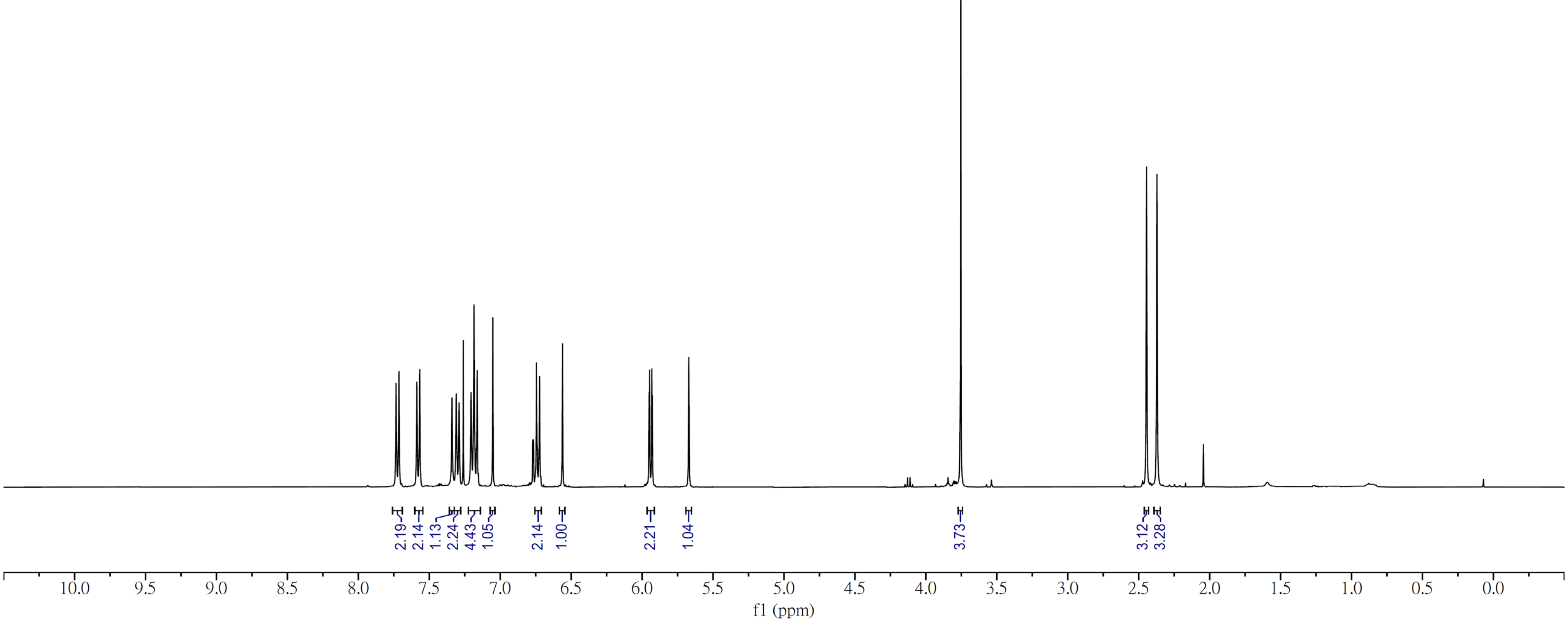

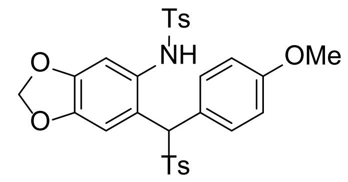

1g

($^{13}C\{^1H\}$ NMR, 101 MHz, $CDCl_3$)

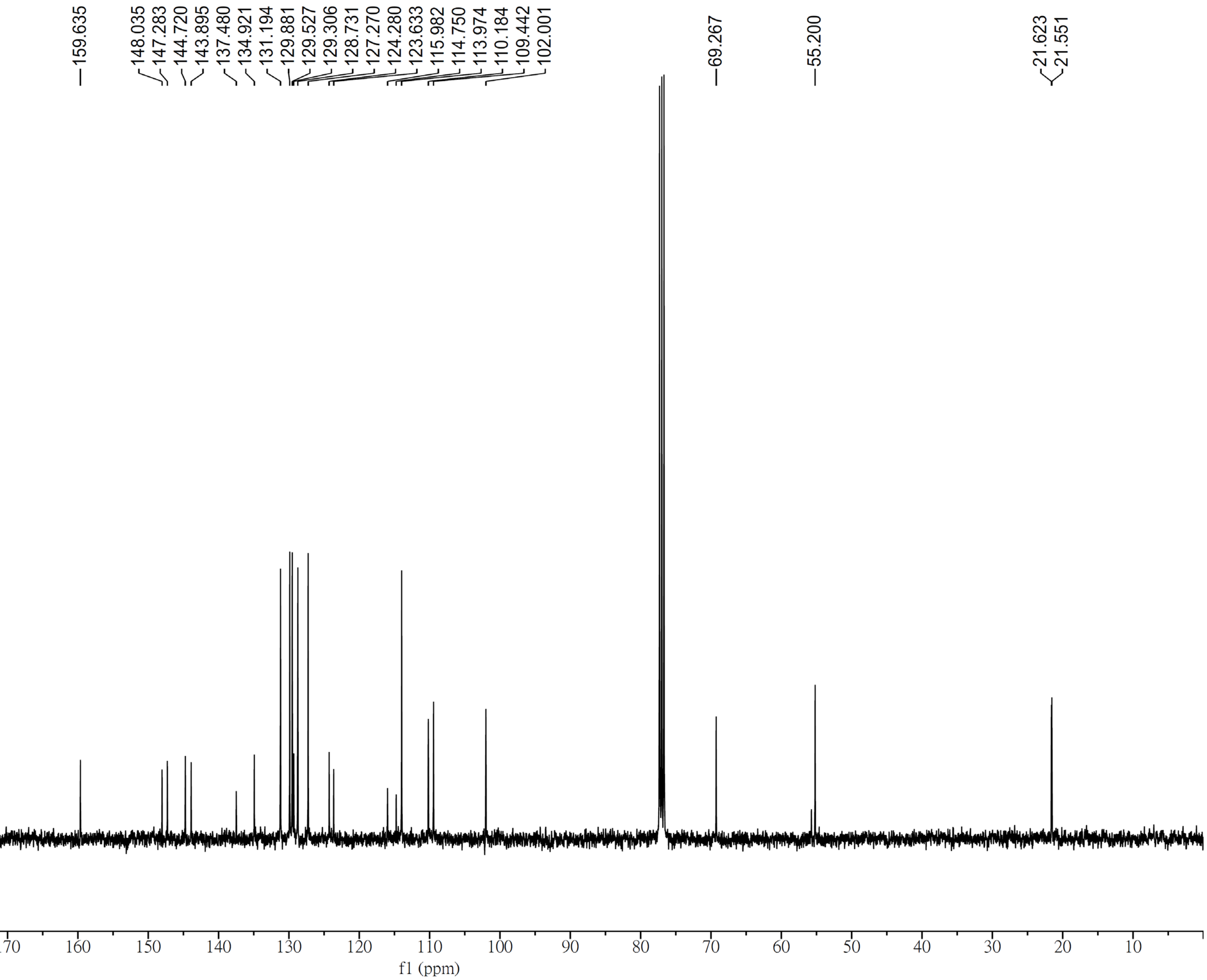

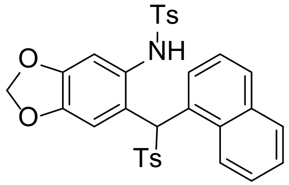

1h

(^{1}H NMR, 400MHz, $CDCl_3$)

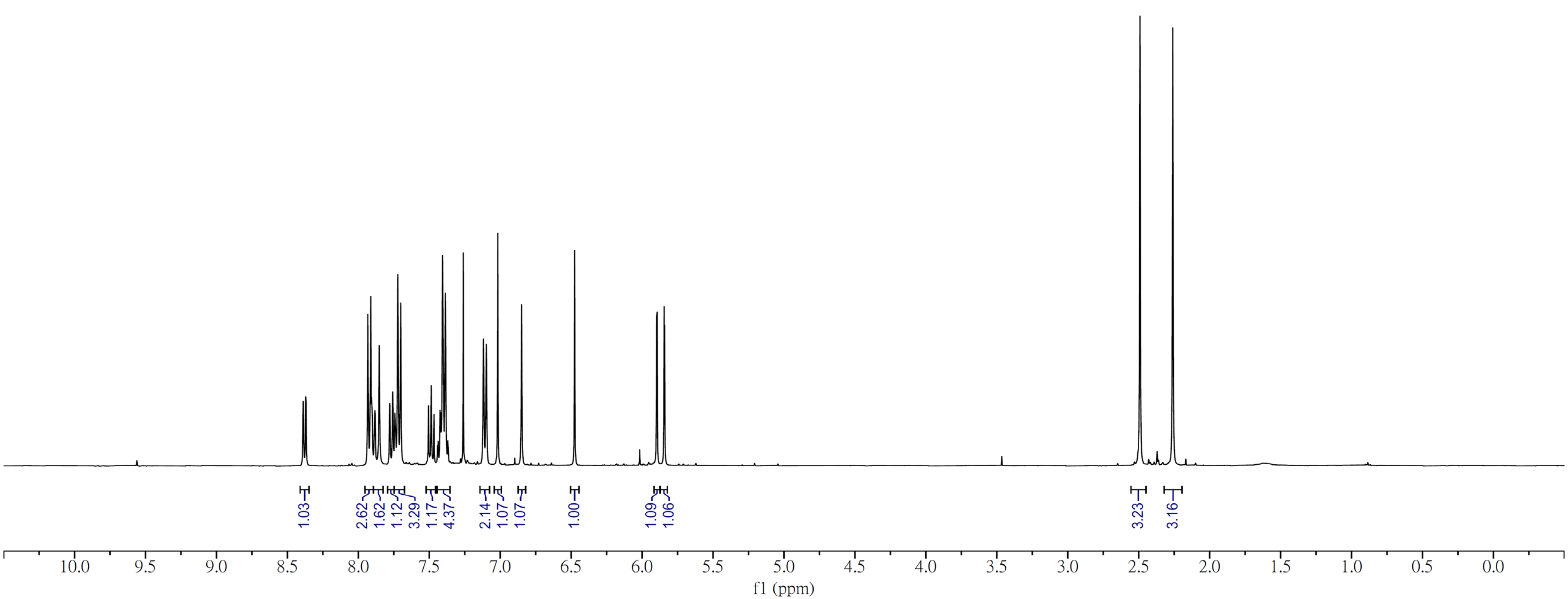

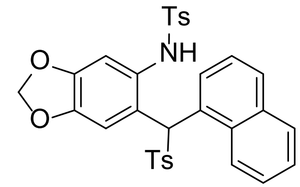

1h

($^{13}C\{^{1}H\}$ NMR, 101 MHz, $CDCl_3$)

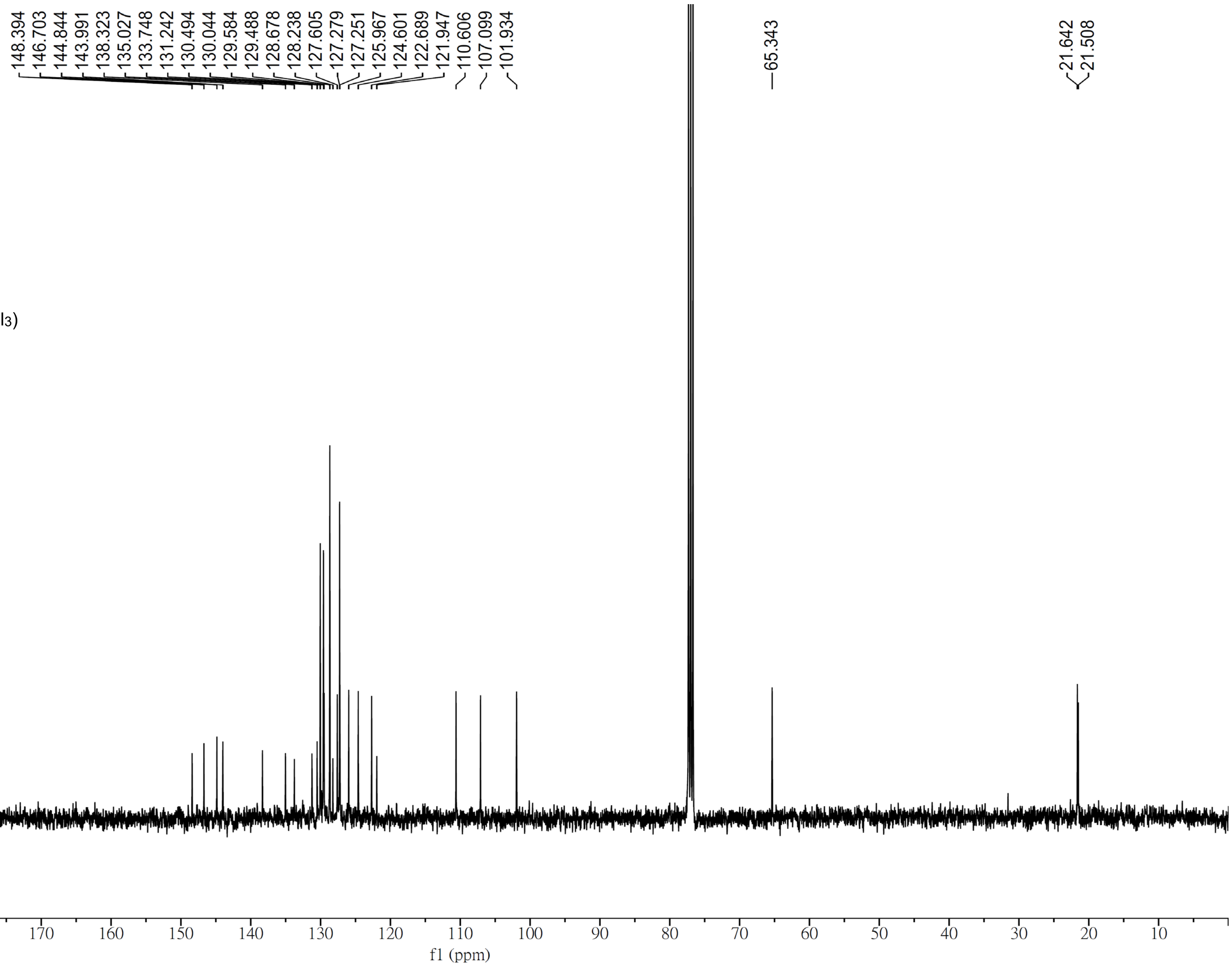

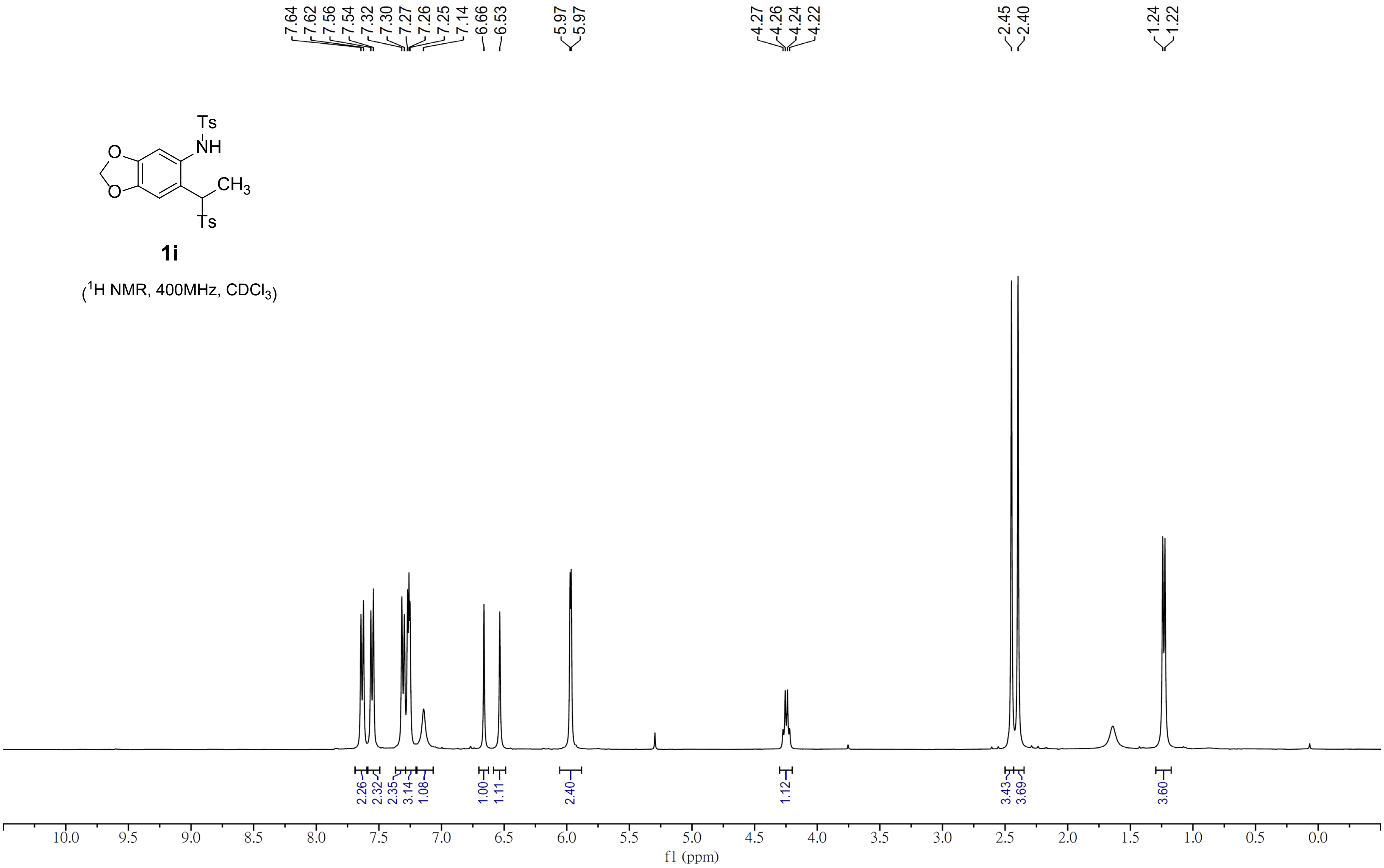
7.64
7.62
7.56
7.54
7.32
7.30
7.27
7.26
7.25
7.14
6.66
6.53
5.97
5.97
4.27
4.26
4.24
4.22
2.45
2.40
1.24
1.22
Ts
NH
O
O
CH3
Ts
1i
(1H NMR, 400MHz, CDCl3)
2.26
2.32
2.35
3.14
1.08
1.00
1.11
2.40
1.12
3.43
3.69
3.60
10.0
9.5
9.0
8.5
8.0
7.5
7.0
6.5
6.0
5.5
5.0
4.5
4.0
3.5
3.0
2.5
2.0
1.5
1.0
0.5
0.0
f1 (ppm)

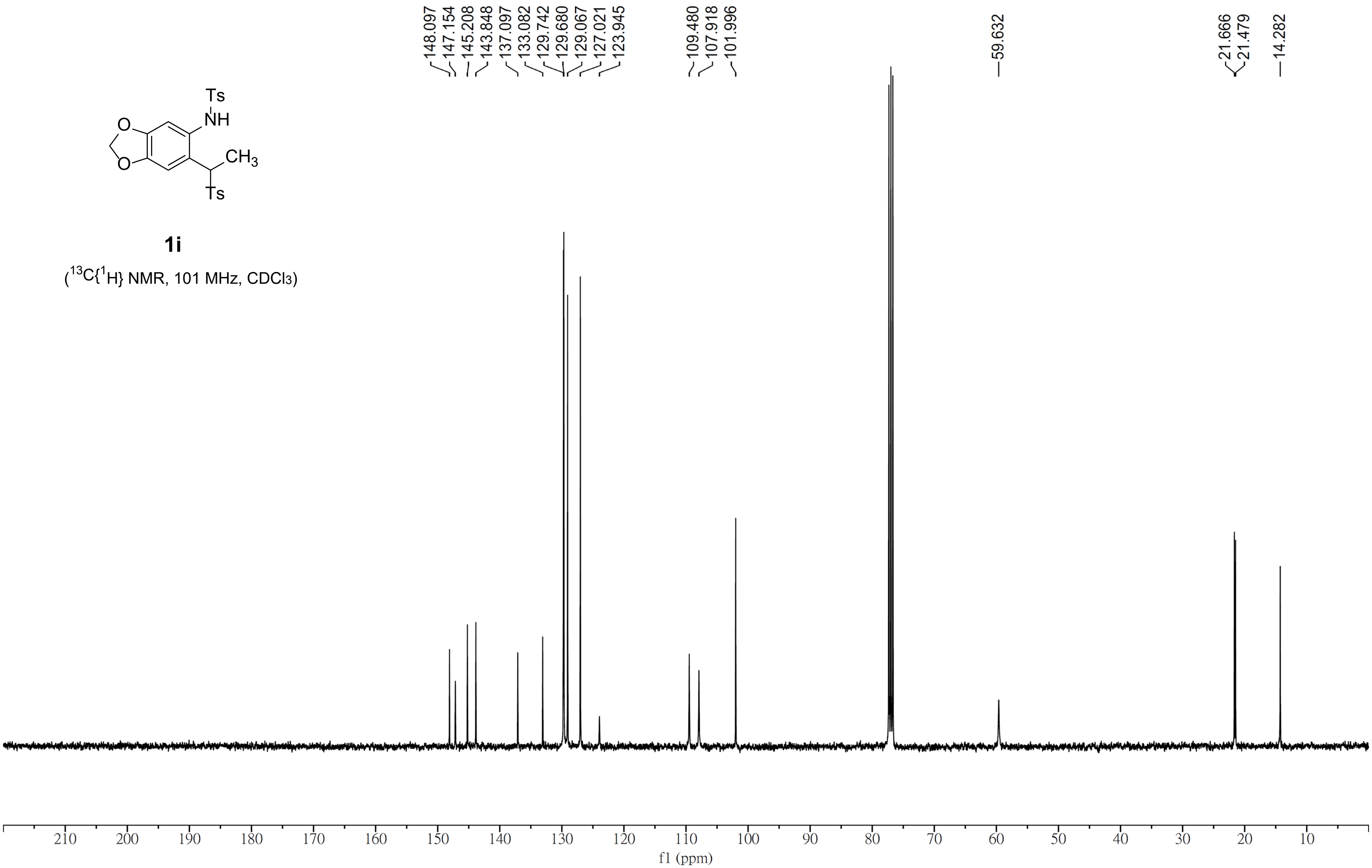

1i

($^{13}C\{^{1}H\}$ NMR, 101 MHz, $CDCl_3$)

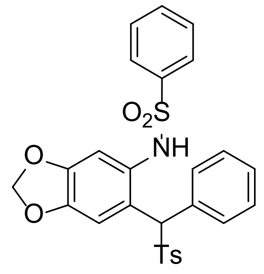

1j

(^{1}H NMR, 400MHz, $CDCl_3$)

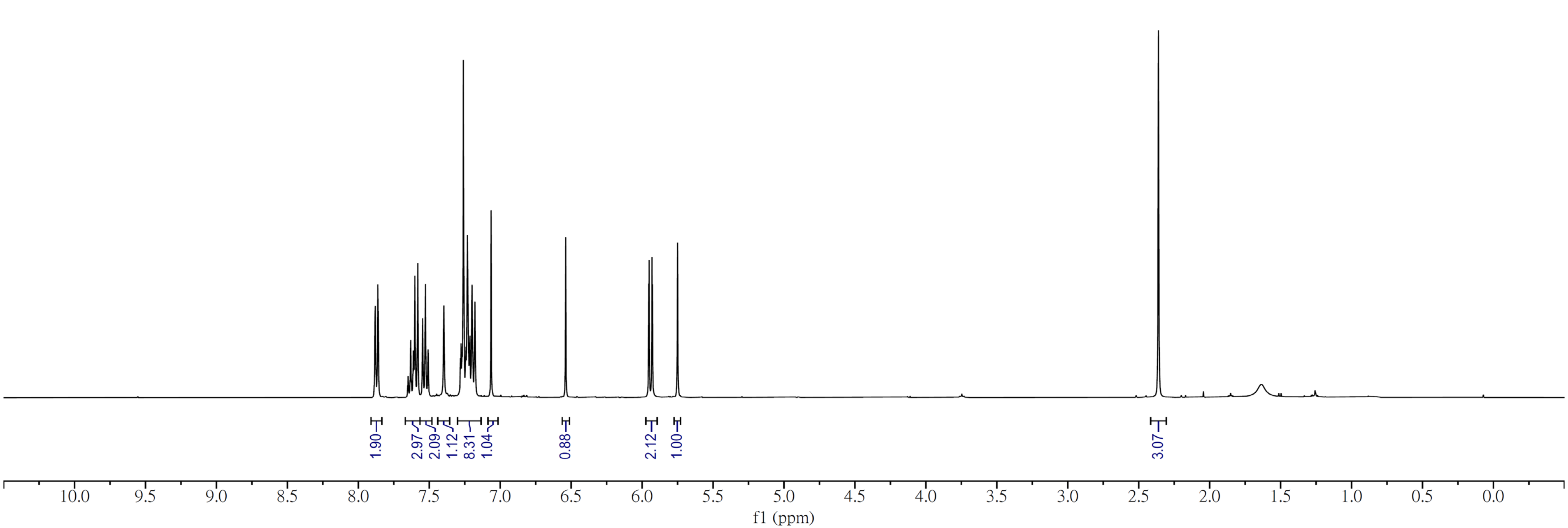

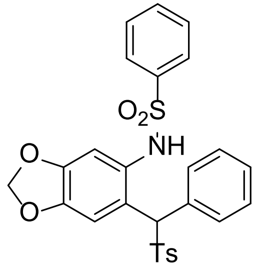

1j

($^{13}C\{^1H\}$ NMR, 101 MHz, $CDCl_3$)

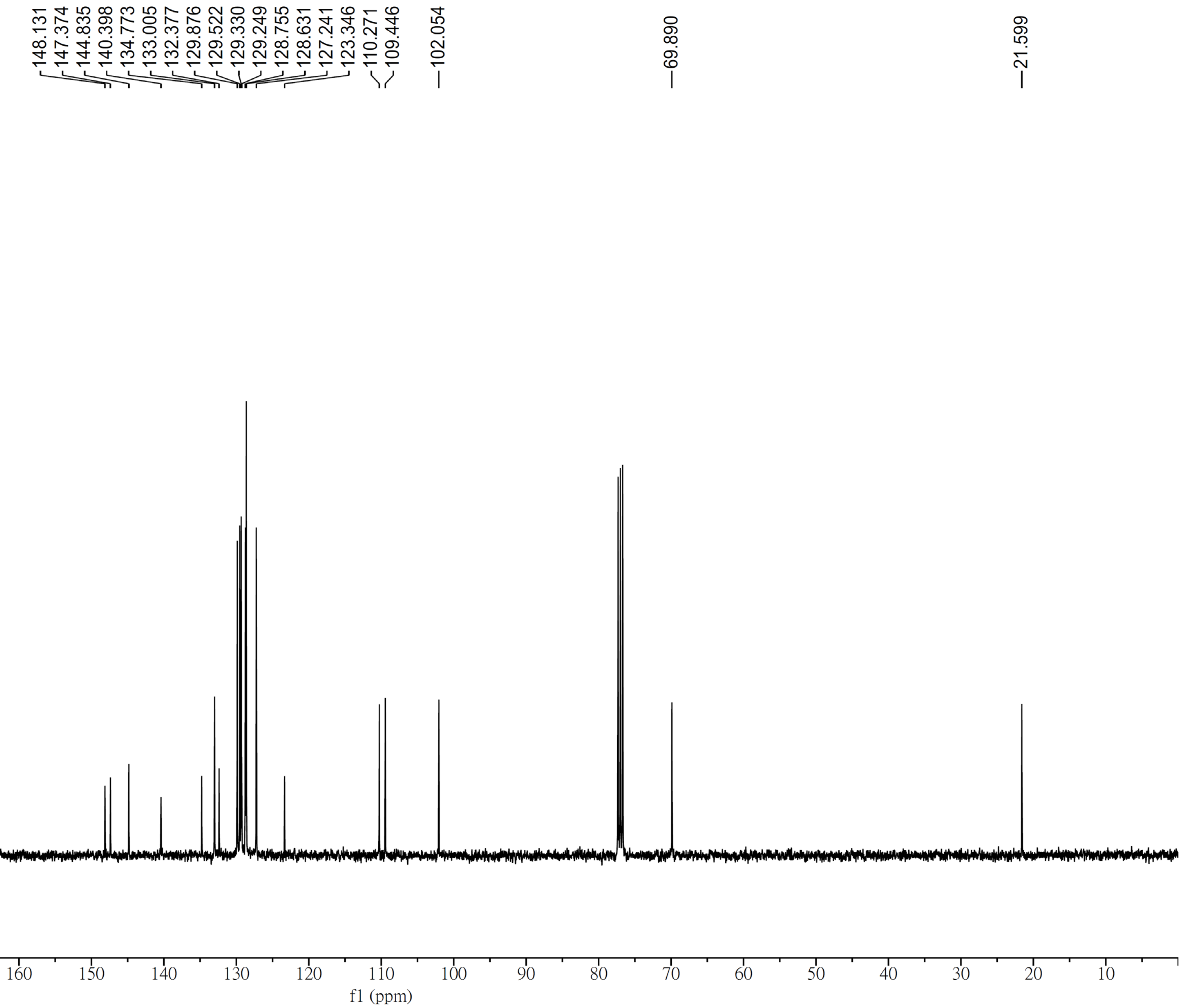

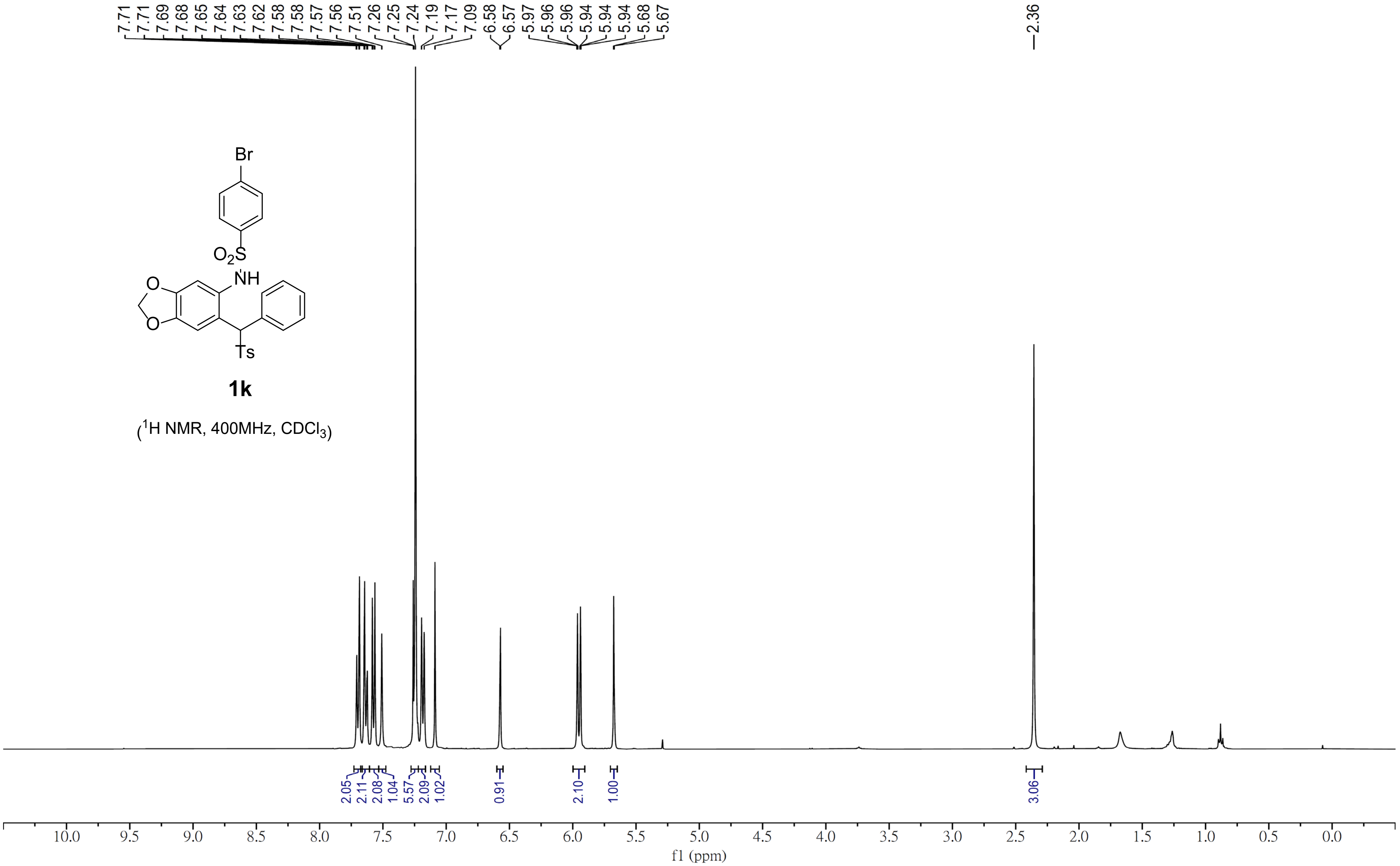

1k

(^{1}H NMR, 400MHz, $CDCl_3$)

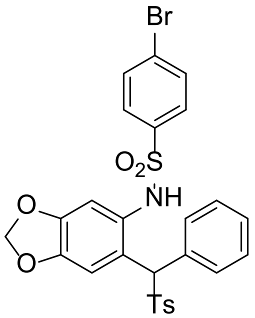

1k

($^{13}C\{^1H\}$ NMR, 101 MHz, $CDCl_3$)

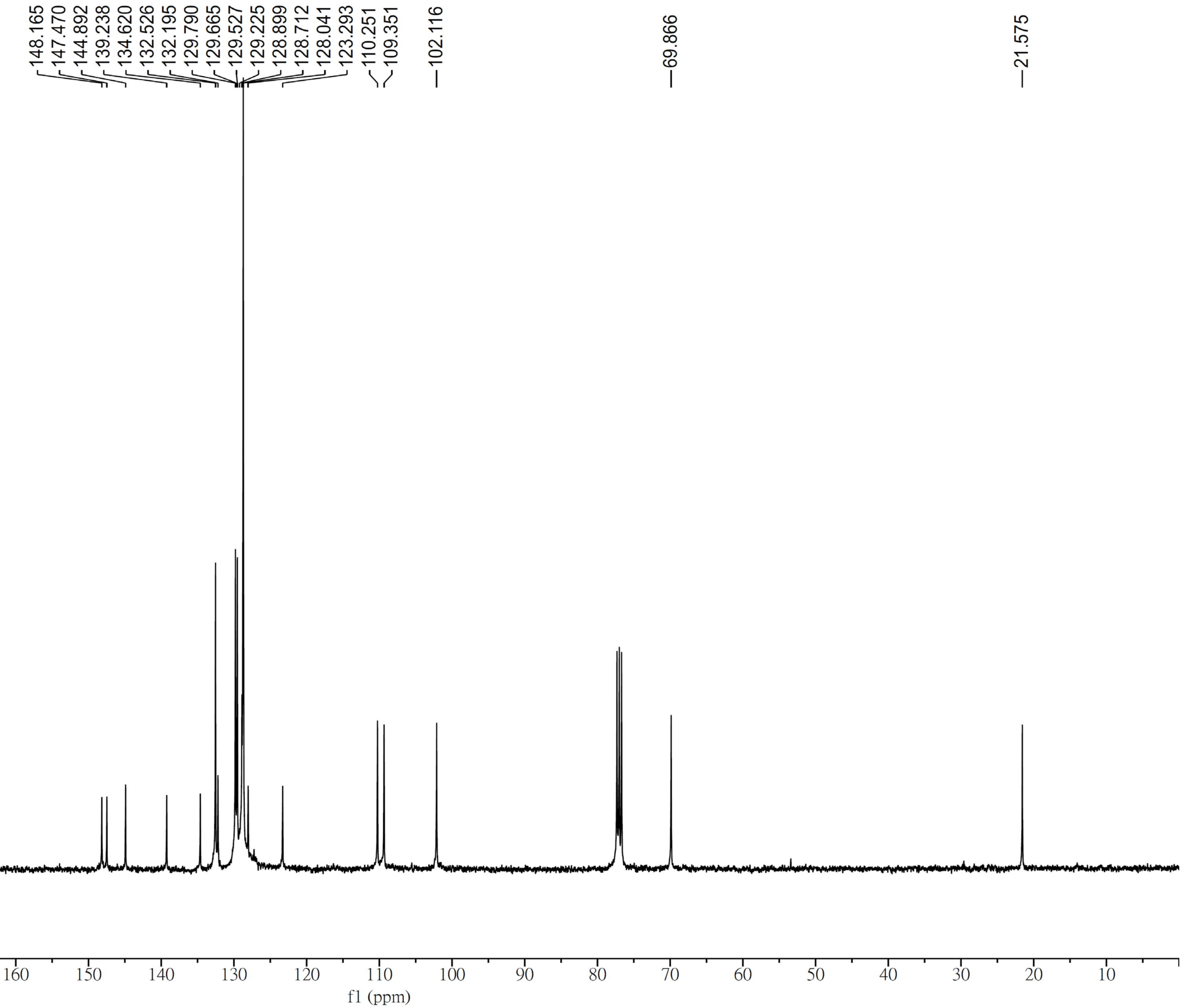

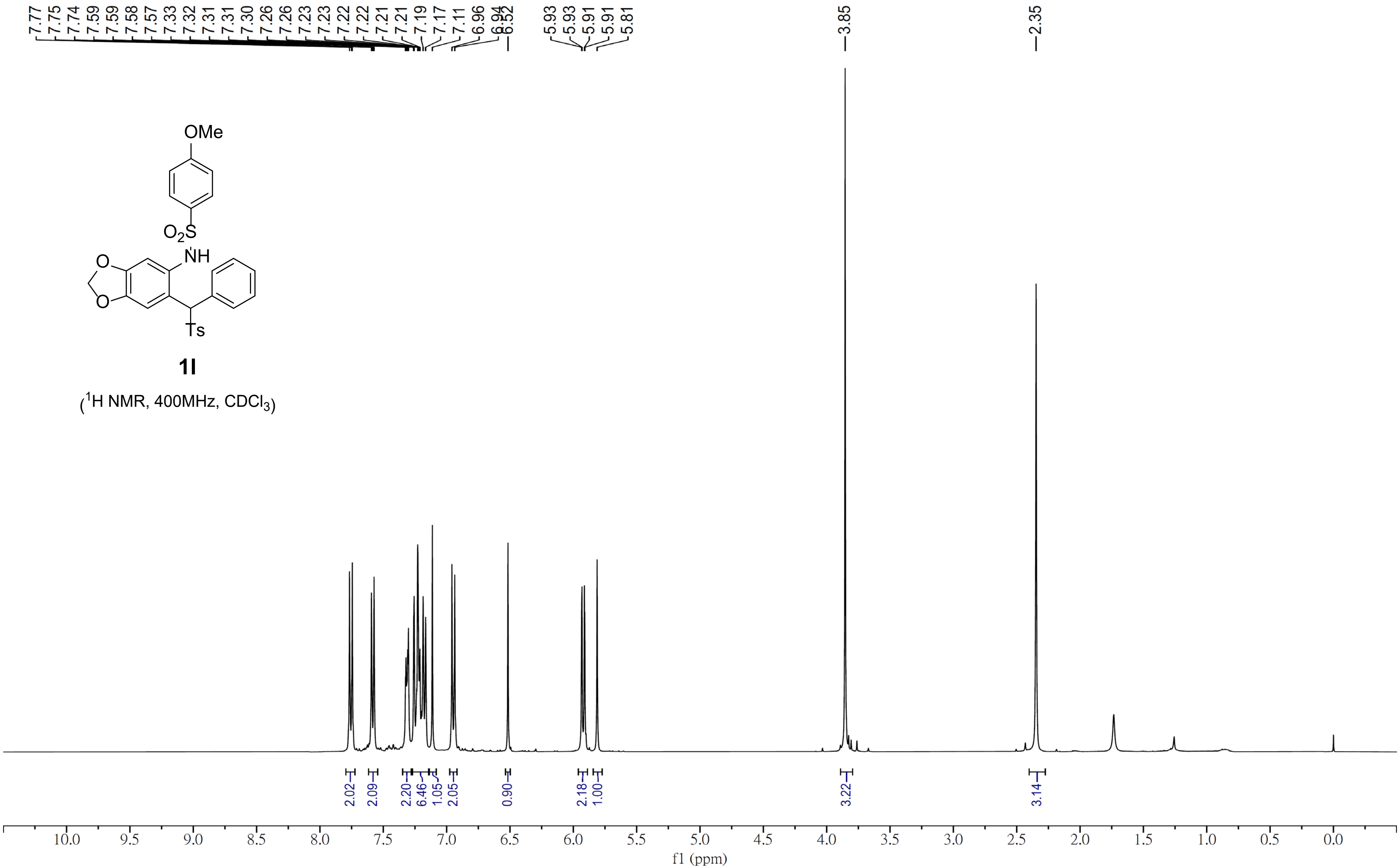

1l

(^{1}H NMR, 400MHz, $CDCl_3$)

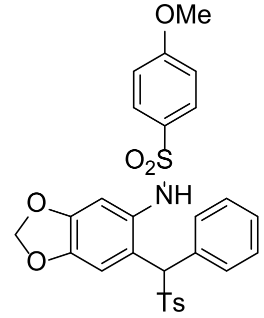

1l

($^{13}C\{^{1}H\}$ NMR, 101 MHz, $CDCl_3$)

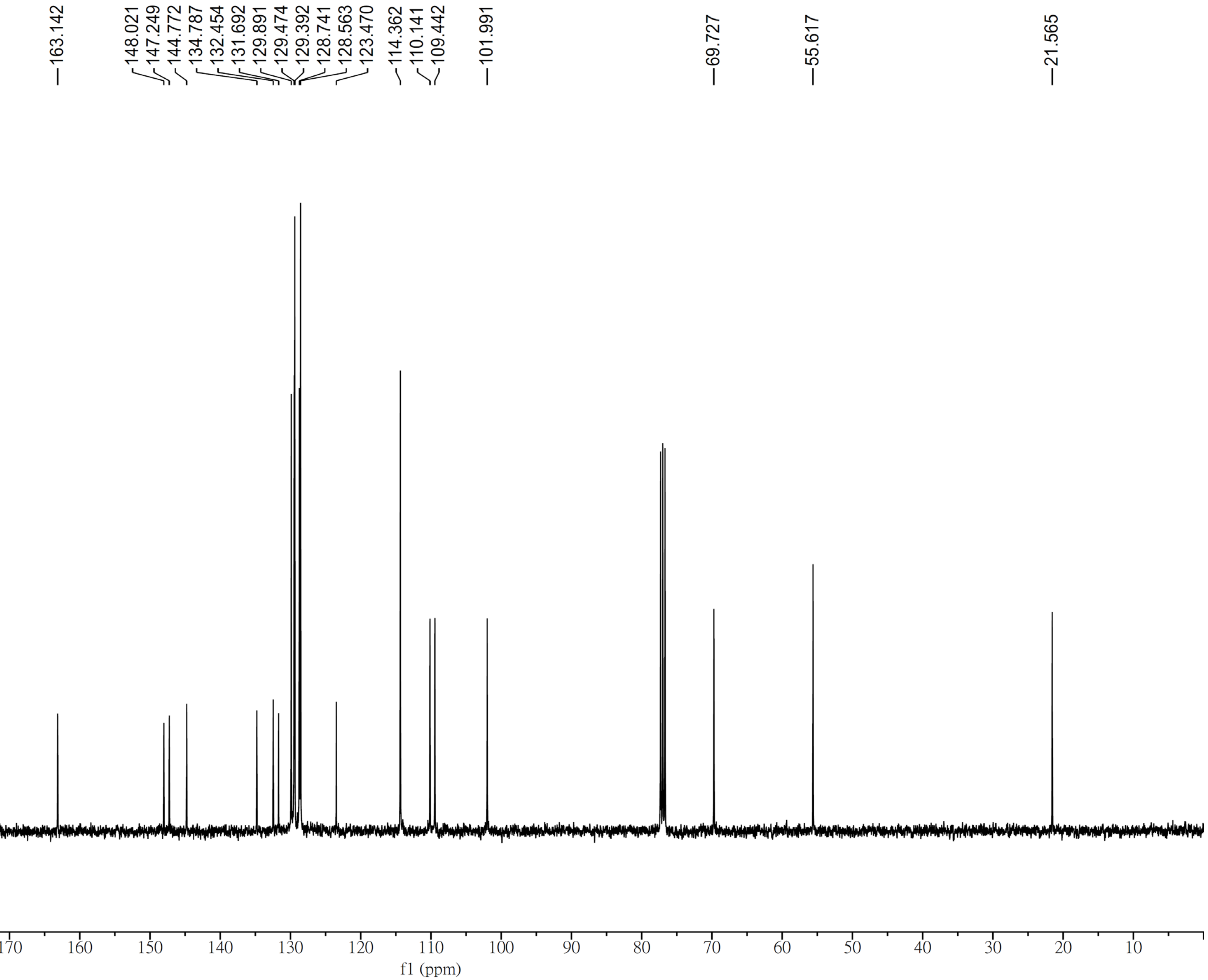

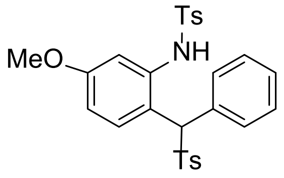

1n

(^{1}H NMR, 400MHz, $CDCl_3$)

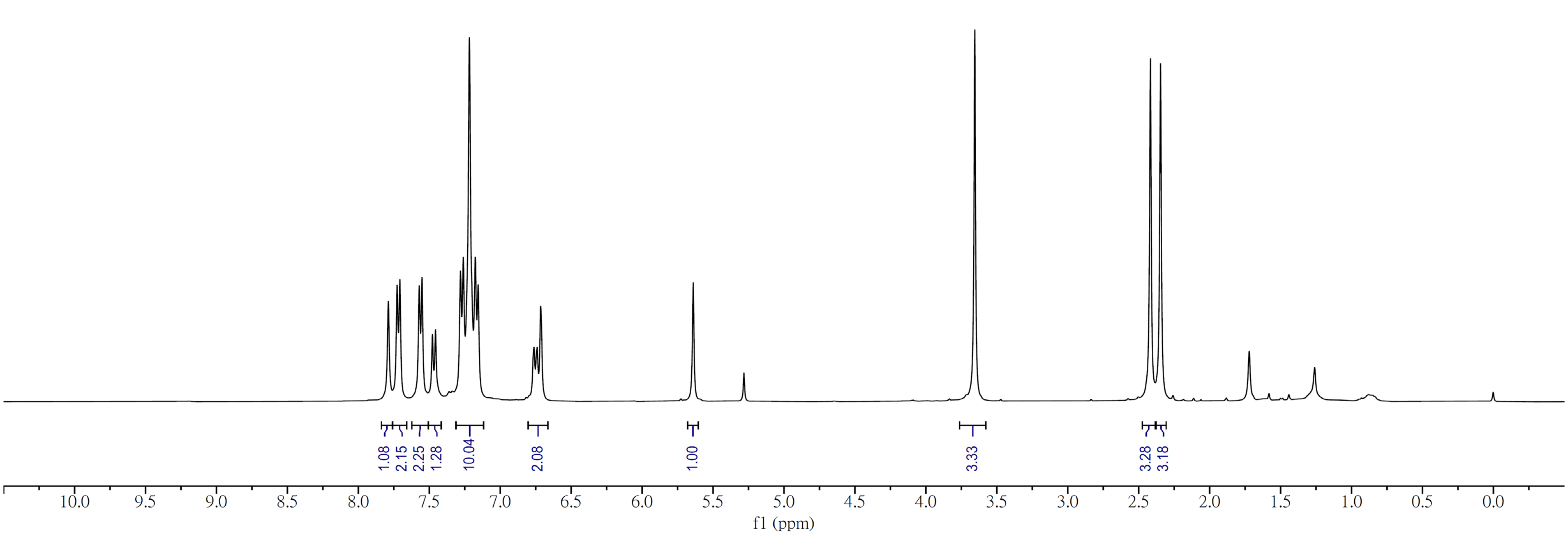

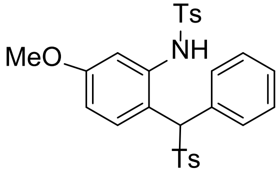

1n

($^{13}C\{^{1}H\}$ NMR, 101 MHz, $CDCl_3$)

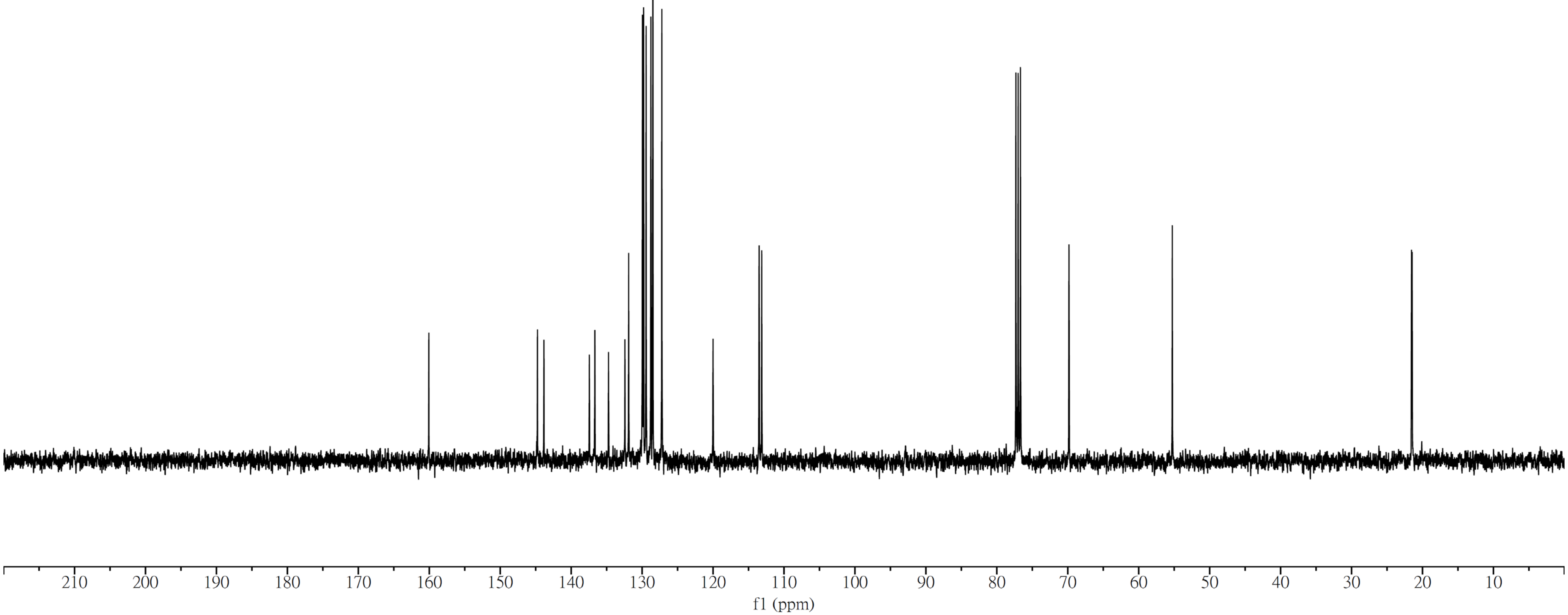

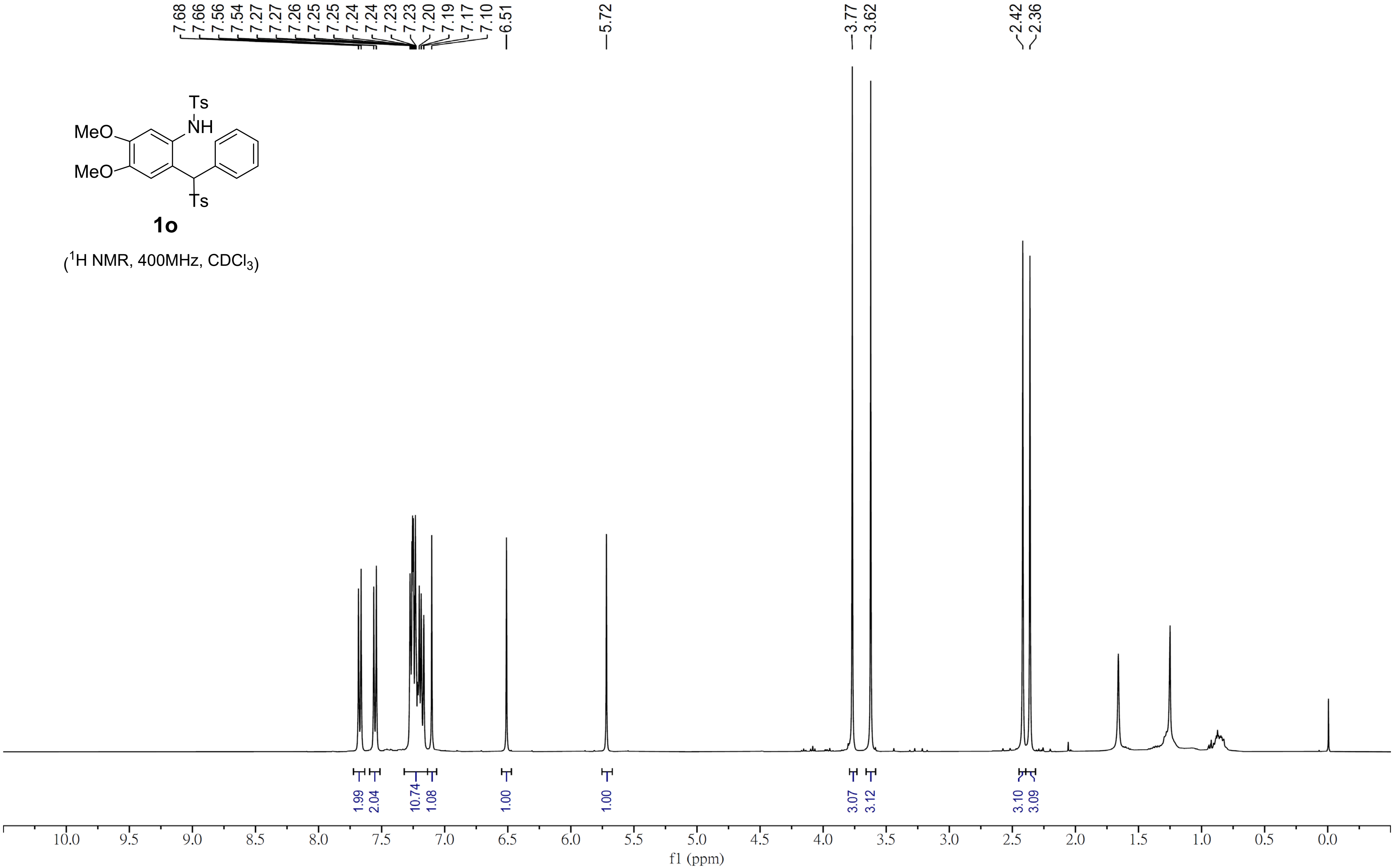

7.68
7.66
7.56
7.54
7.27
7.27
7.26
7.25
7.25
7.24
7.24
7.23
7.23
7.20
7.19
7.17
7.10
6.51
5.72
3.77
3.62
2.42
2.36
Ts
NH
MeO
MeO
Ts
1o
(^{1}H NMR, 400MHz, CDCl$_3$)
1.99
2.04
10.74
1.08
1.00
1.00
3.07
3.12
3.10
3.09
10.0
9.5
9.0
8.5
8.0
7.5
7.0
6.5
6.0
5.5
5.0
4.5
4.0
3.5
3.0
2.5
2.0
1.5
1.0
0.5
0.0
f1 (ppm)

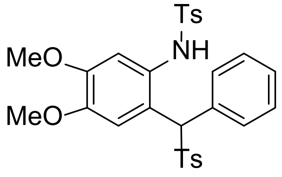

1o

($^{13}C\{^{1}H\}$ NMR, 101 MHz, $CDCl_3$)

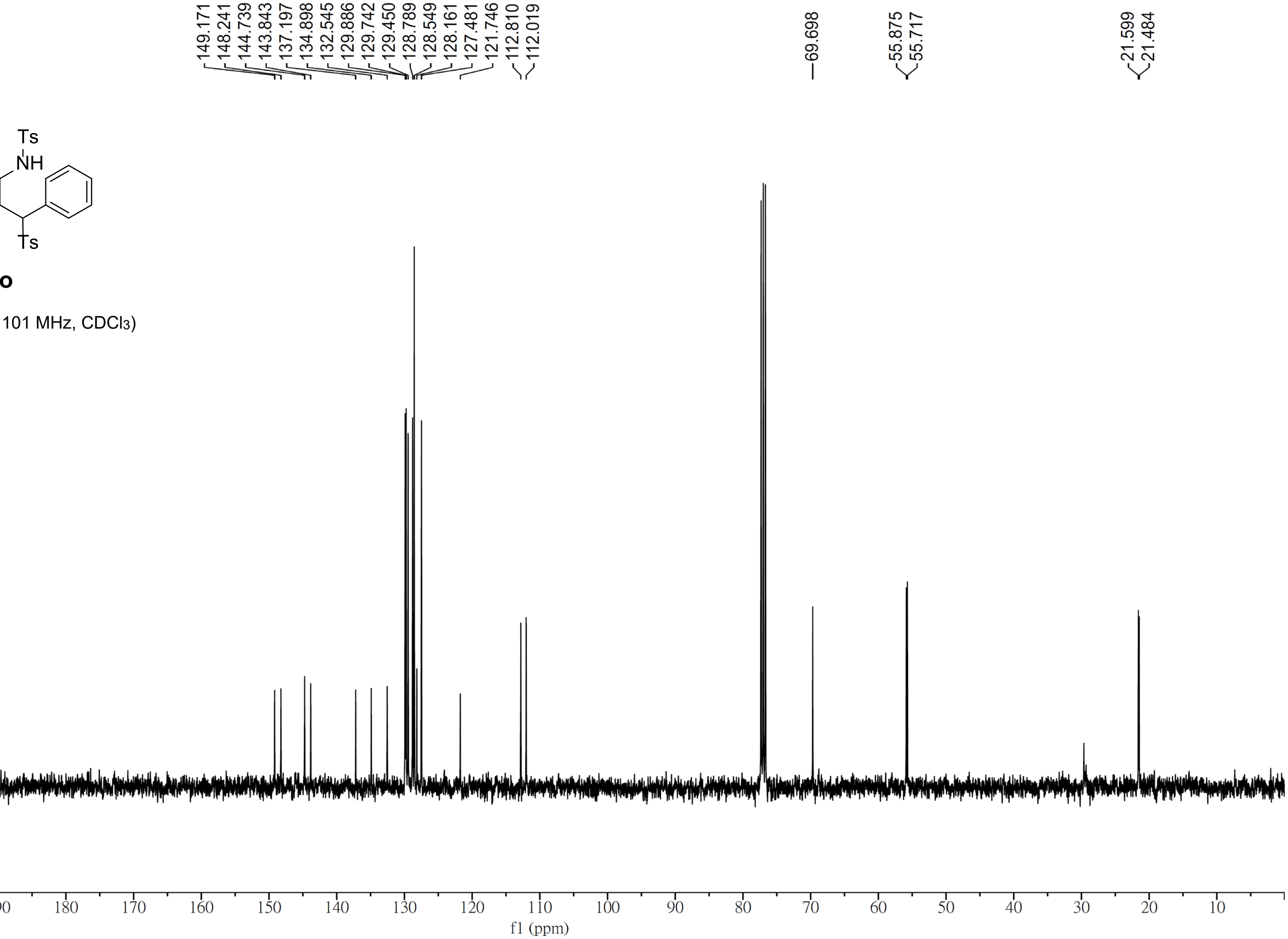

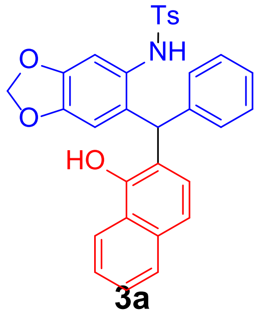

3a

(¹H NMR, 400MHz, $CDCl_3$)

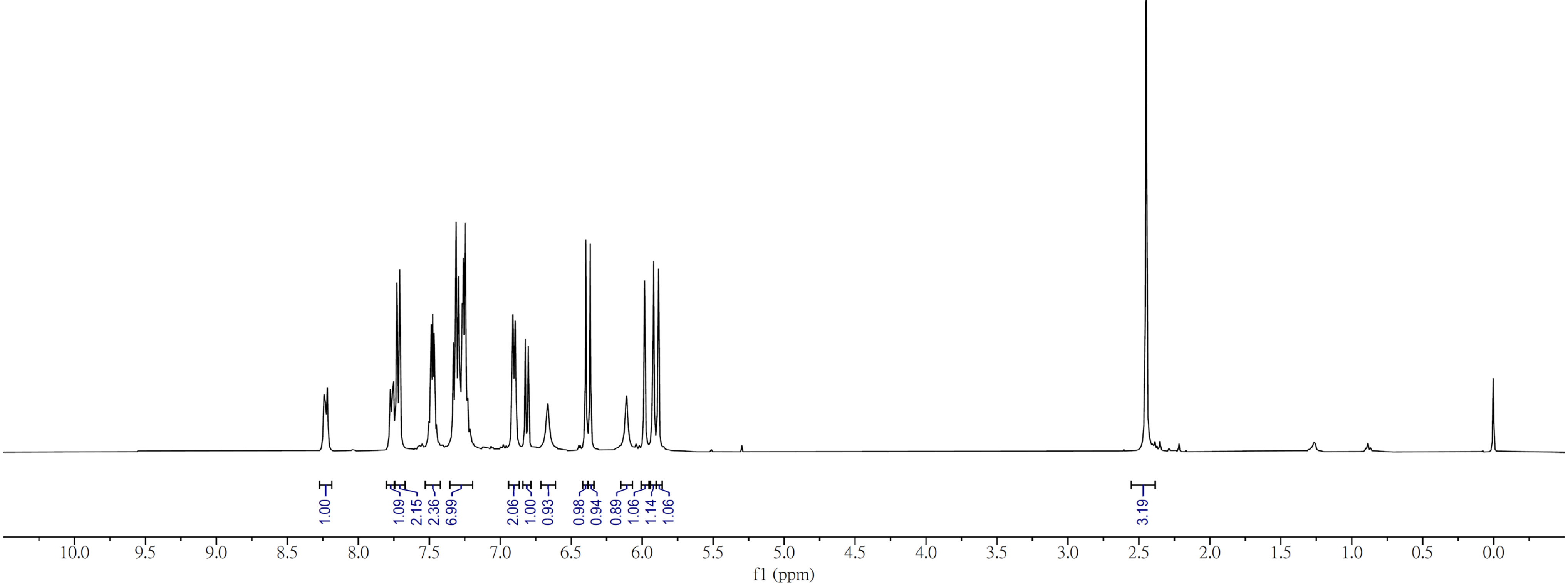

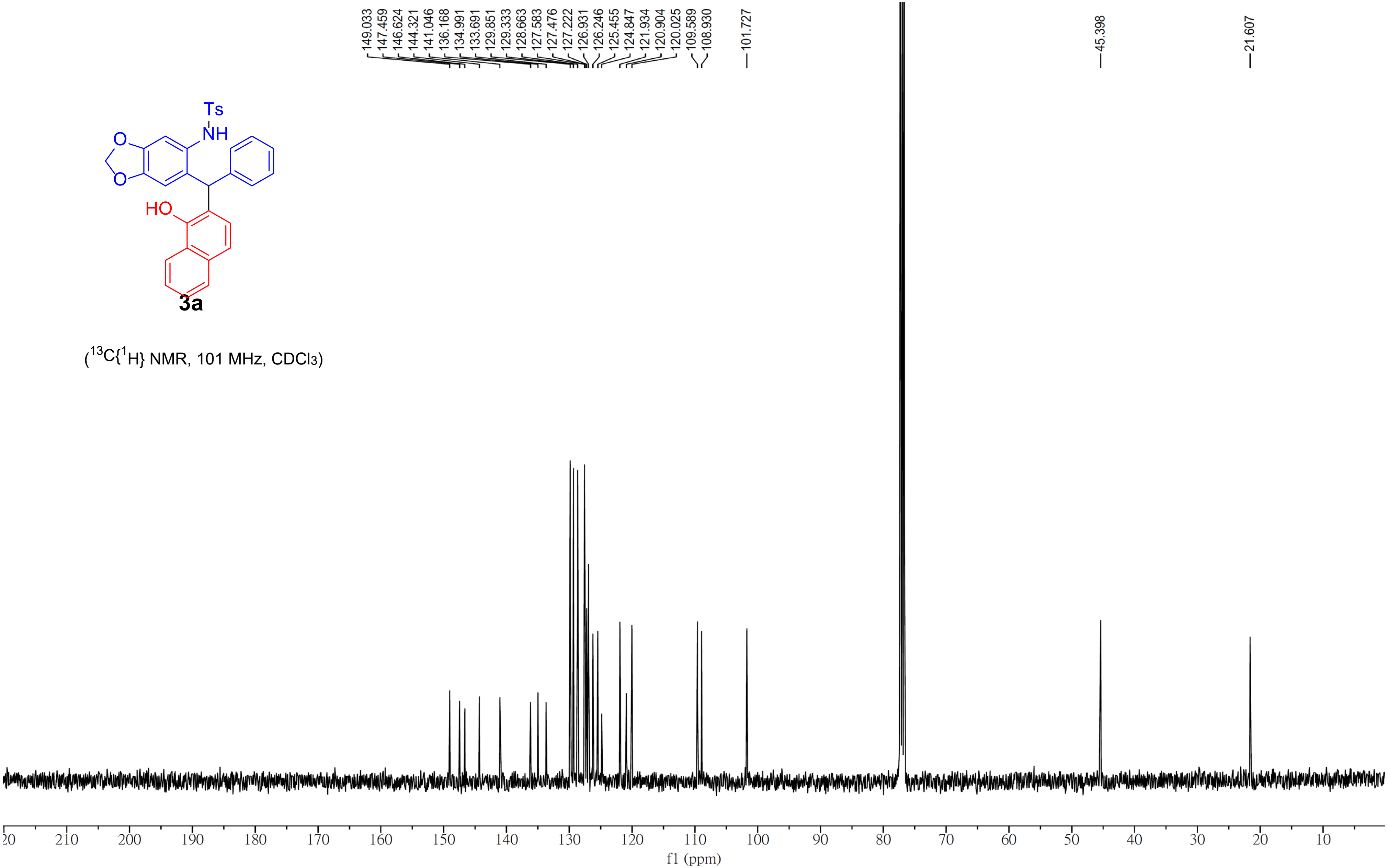

3a

($^{13}C\{^{1}H\}$ NMR, 101 MHz, $CDCl_3$)

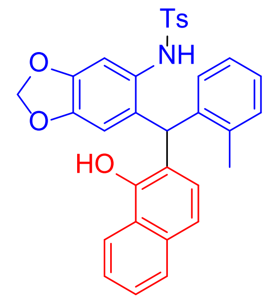

3b

(^{1}H NMR, 400MHz, $CDCl_3$)

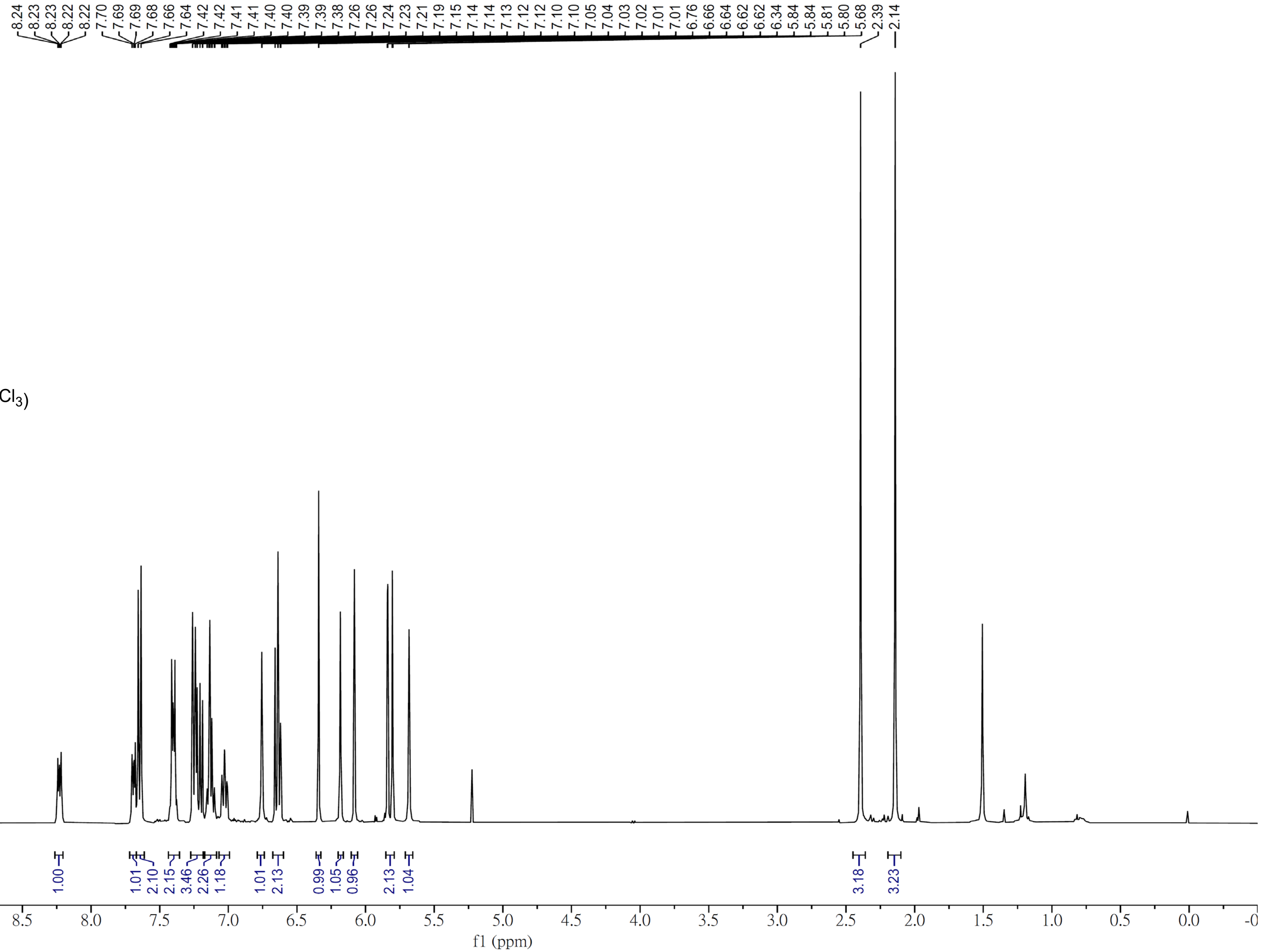

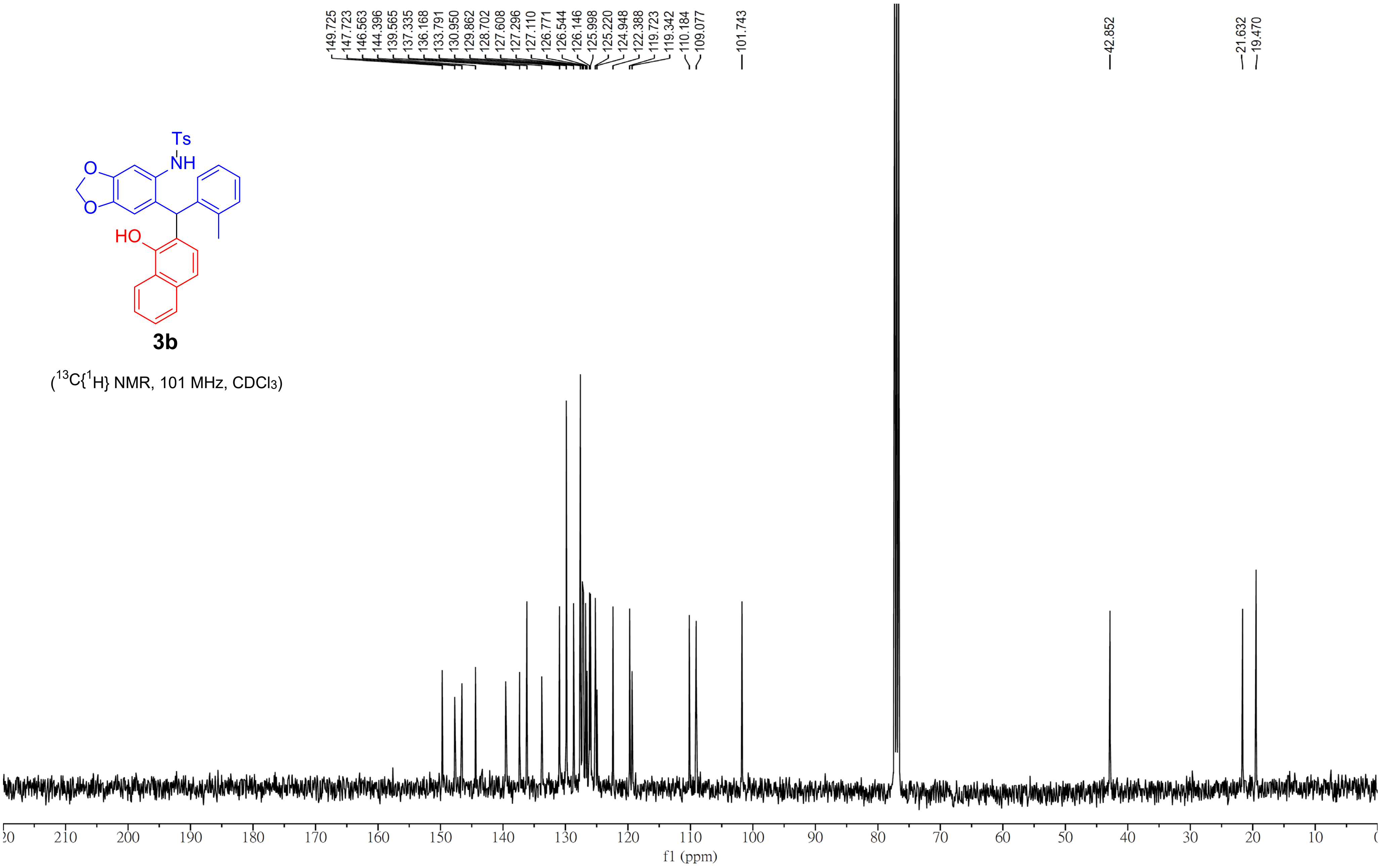
149.725
147.723
146.563
144.396
139.565
137.335
136.168
133.791
130.950
129.862
128.702
127.608
127.296
127.110
126.771
126.544
126.146
125.998
125.220
124.948
122.388
119.723
119.342
110.184
109.077
101.743
42.852
21.632
19.470
Ts
NH
O
O
HO
3b
(^{13}C{^{1}H} NMR, 101 MHz, CDCl$_3$)
f1 (ppm)

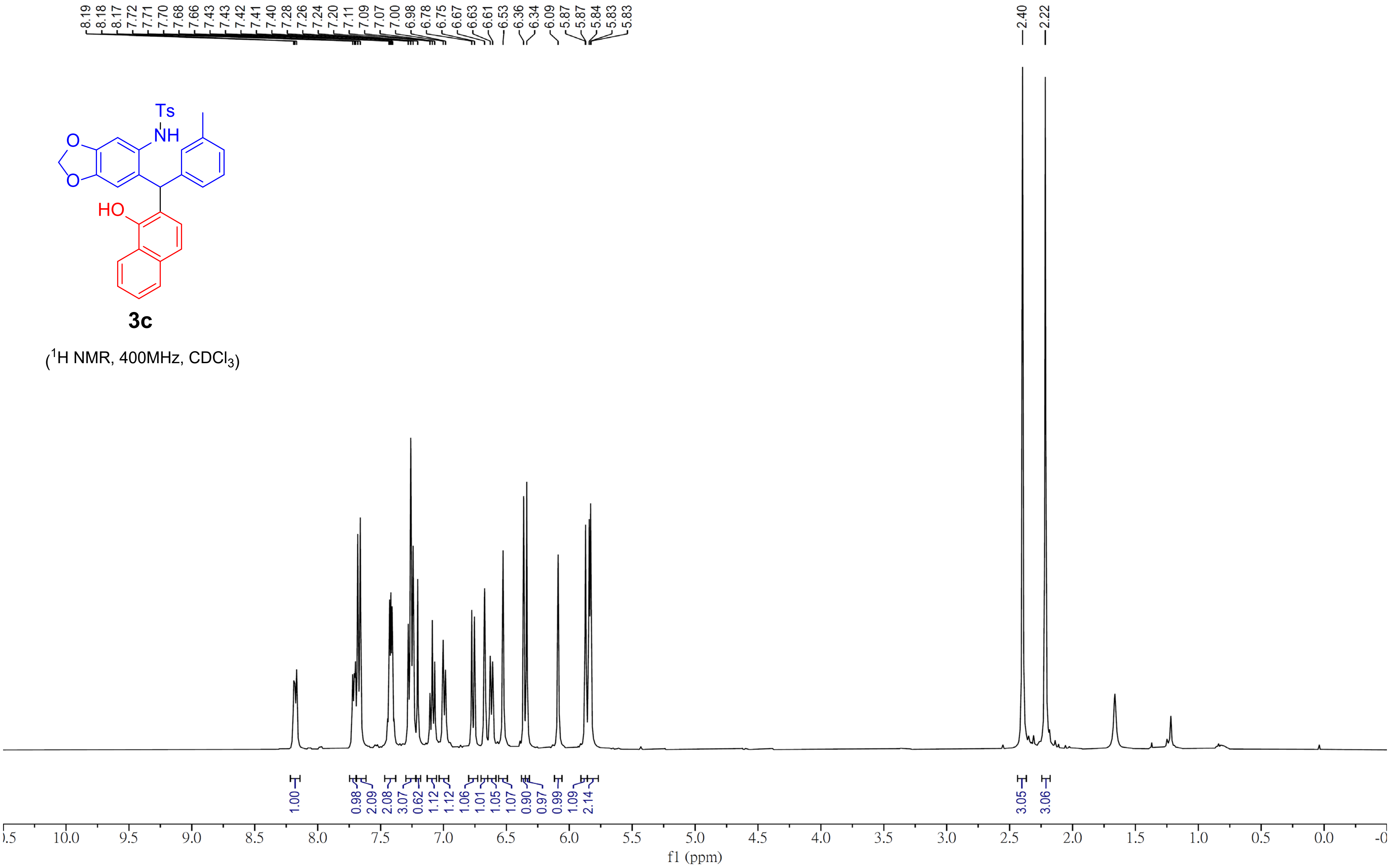
Ts
NH
O
O
HO
3c
(1H NMR, 400MHz, CDCl3)
8.19 8.18 8.17 7.72 7.71 7.70 7.68 7.66 7.43 7.43 7.42 7.41 7.40 7.28 7.26 7.24 7.20 7.11 7.09 7.07 7.00 6.98 6.78 6.75 6.67 6.63 6.61 6.53 6.36 6.34 6.09 5.87 5.87 5.84 5.83 5.83
2.40 2.22
1.00 0.98 2.09 2.08 3.07 0.62 1.12 1.12 1.06 1.01 1.05 1.07 0.90 0.97 0.99 1.09 2.14 3.05 3.06
f1 (ppm)

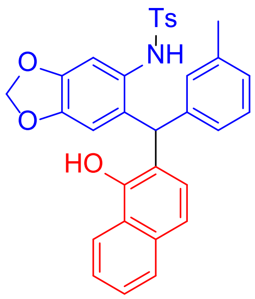

3c

($^{13}C\{^1H\}$ NMR, 101 MHz, $CDCl_3$)

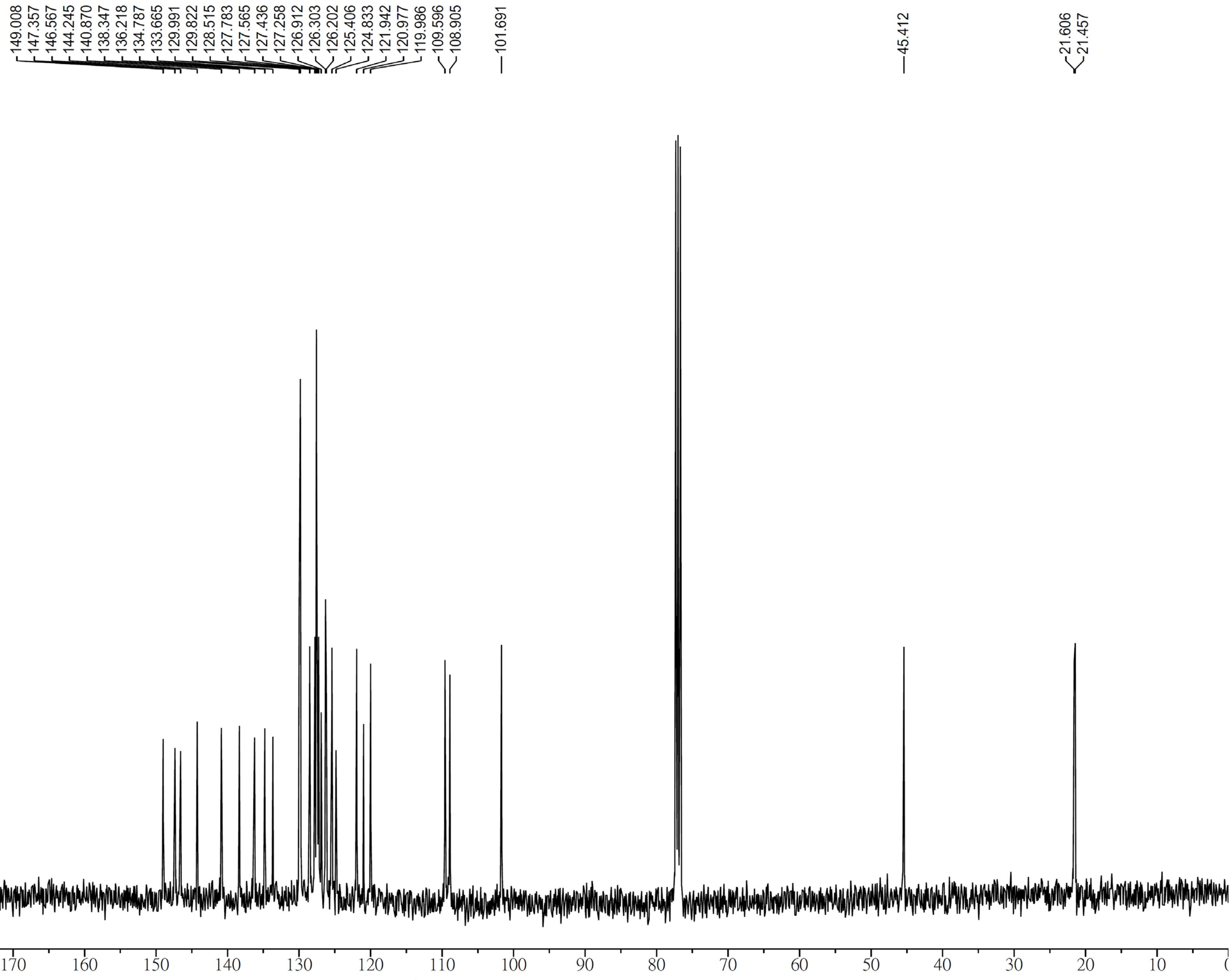

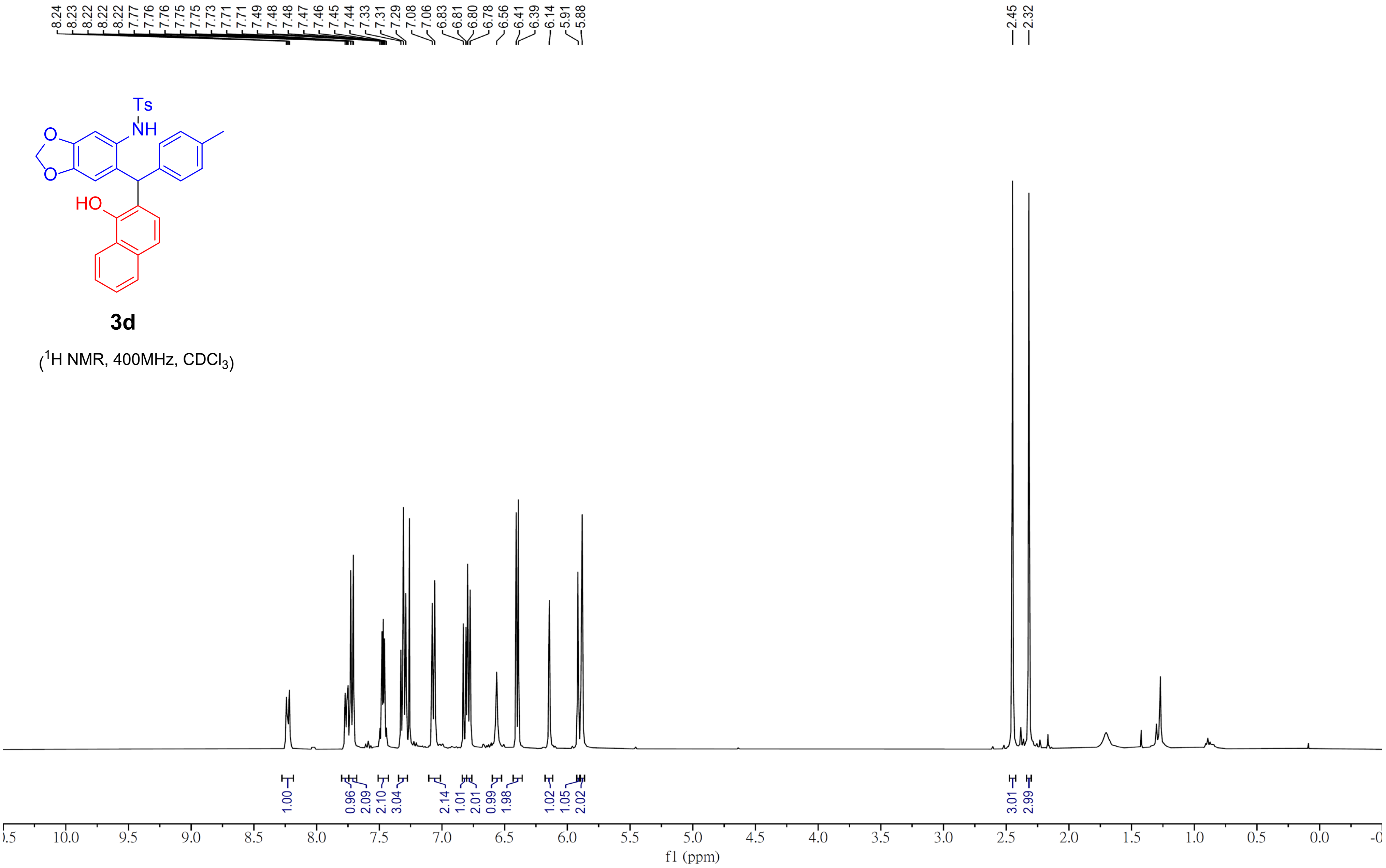

8.24
8.23
8.22
8.22
8.22
7.77
7.76
7.76
7.75
7.75
7.73
7.71
7.71
7.49
7.48
7.48
7.47
7.46
7.45
7.44
7.33
7.31
7.29
7.08
7.06
6.83
6.81
6.80
6.78
6.56
6.41
6.39
6.14
5.91
5.88
2.45
2.32
Ts
NH
O
O
HO
3d
(1H NMR, 400MHz, CDCl3)
1.00
0.96
2.09
2.10
3.04
2.14
1.01
2.01
0.99
1.98
1.02
1.05
2.02
3.01
2.99
f1 (ppm)

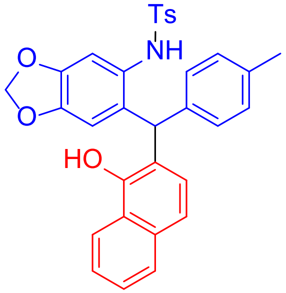

3d

($^{13}C\{^{1}H\}$ NMR, 101 MHz, $CDCl_3$)

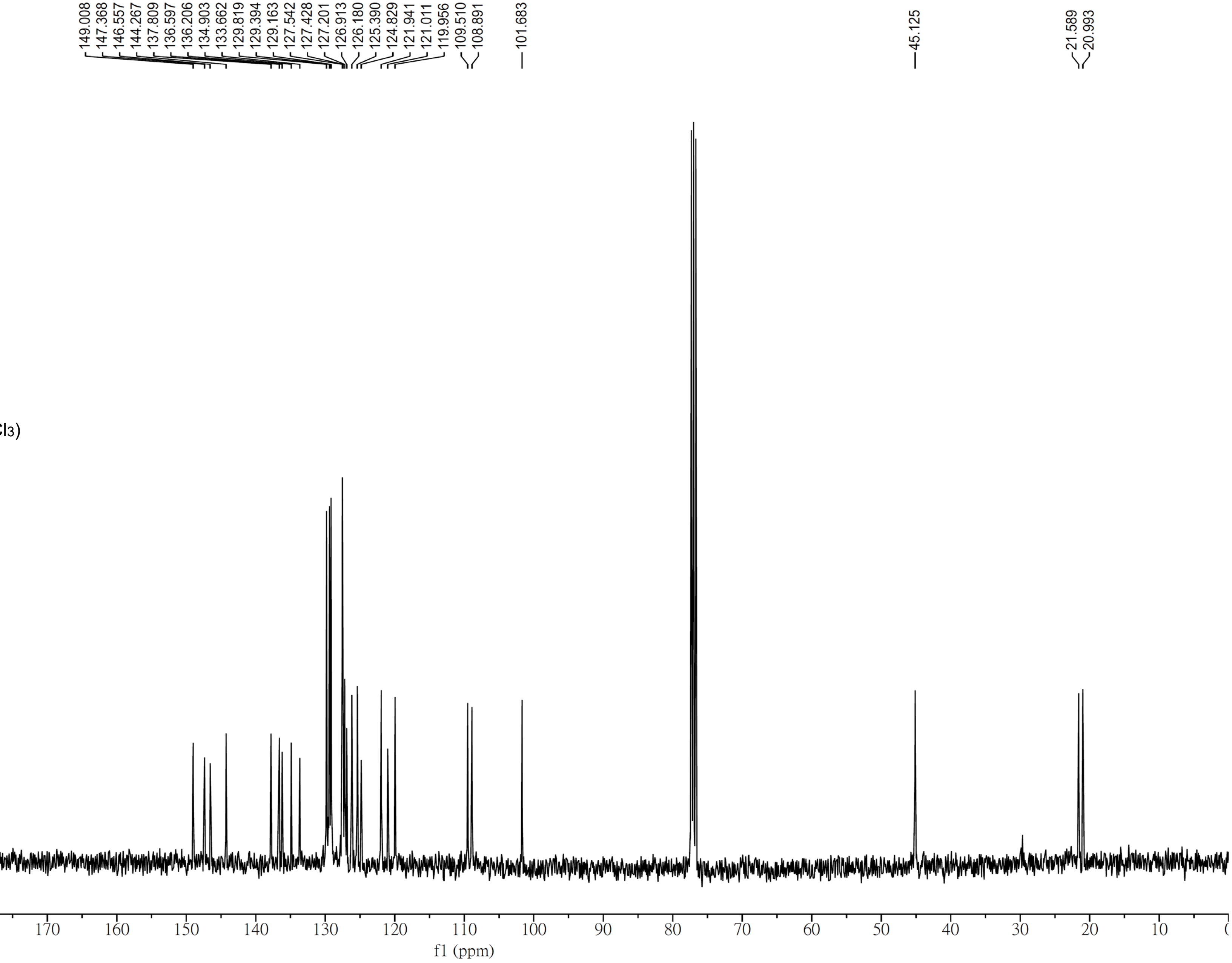

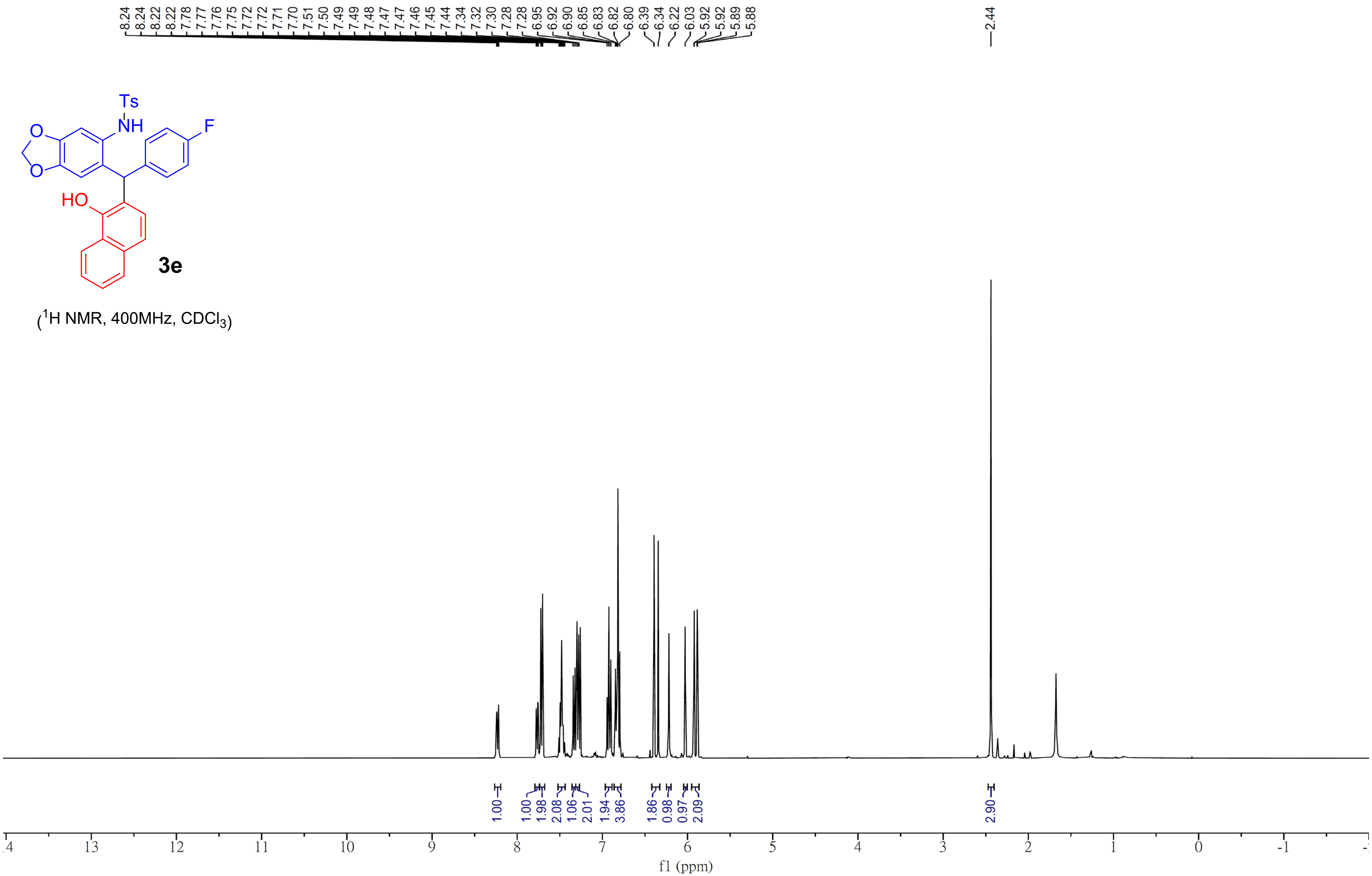

8.24
8.24
8.22
8.22
7.78
7.77
7.76
7.75
7.72
7.72
7.71
7.70
7.51
7.50
7.49
7.49
7.48
7.47
7.47
7.46
7.45
7.44
7.34
7.32
7.30
7.28
7.28
6.95
6.92
6.90
6.85
6.83
6.82
6.80
6.39
6.34
6.22
6.03
5.92
5.92
5.89
5.88
2.44
Ts
NH
F
O
O
HO
3e
(1H NMR, 400MHz, CDCl3)
1.00
1.00
1.98
2.08
1.06
2.01
1.94
3.86
1.86
0.98
0.97
2.09
2.90
13
12
11
10
9
8
7
6
5
4
3
2
1
0
-1
f1 (ppm)

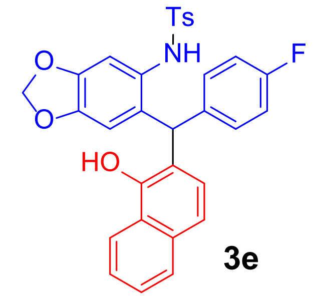

3e

($^{13}C\{^{1}H\}$ NMR, 101 MHz, $CDCl_3$)

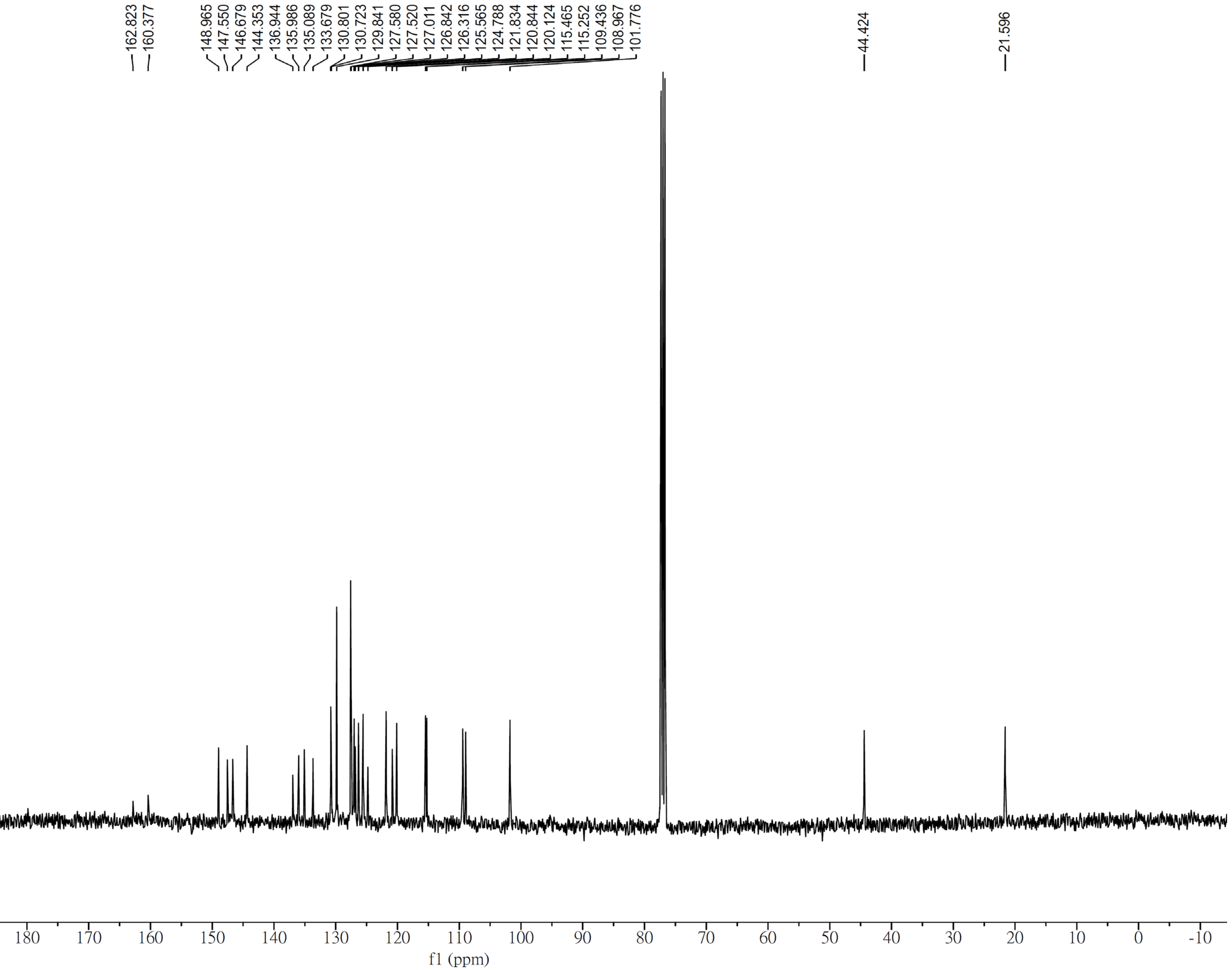

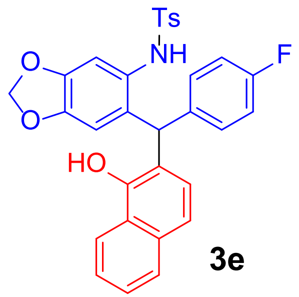

3e

(^{19}F NMR, 376MHz, CDCl$_3$)

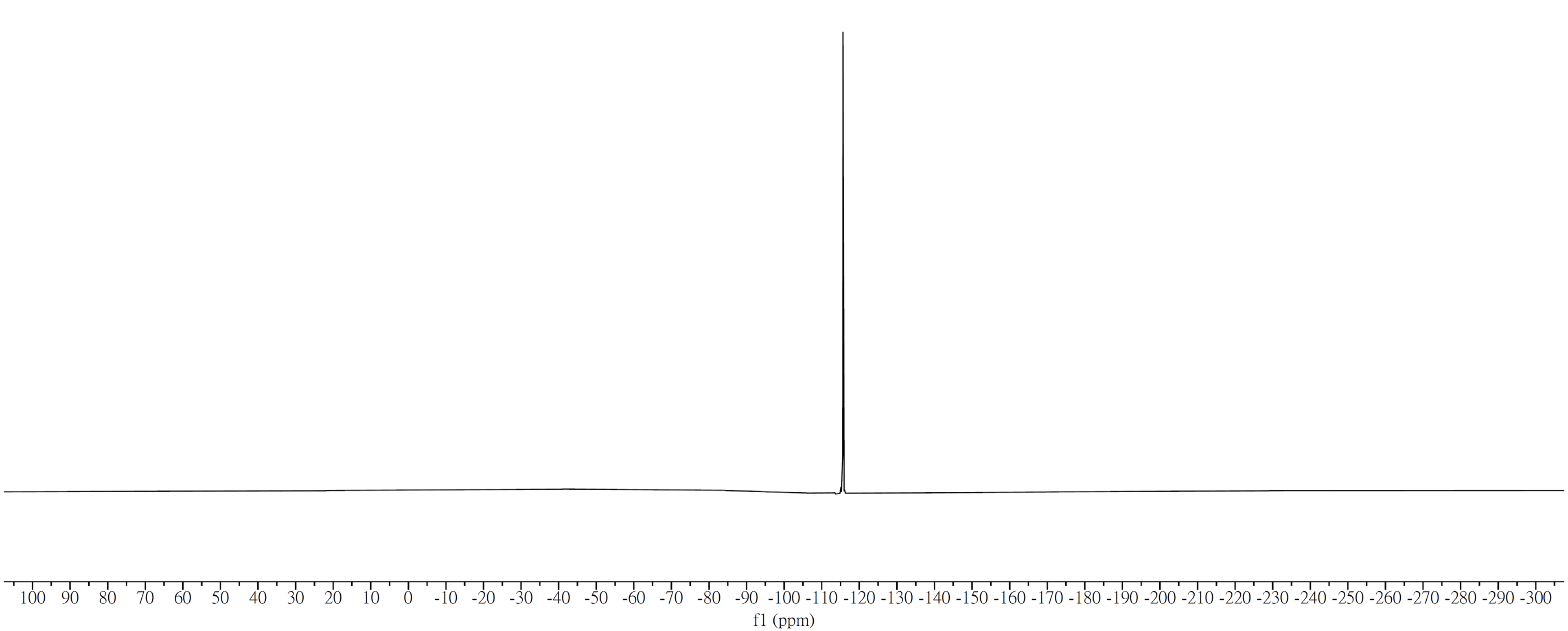

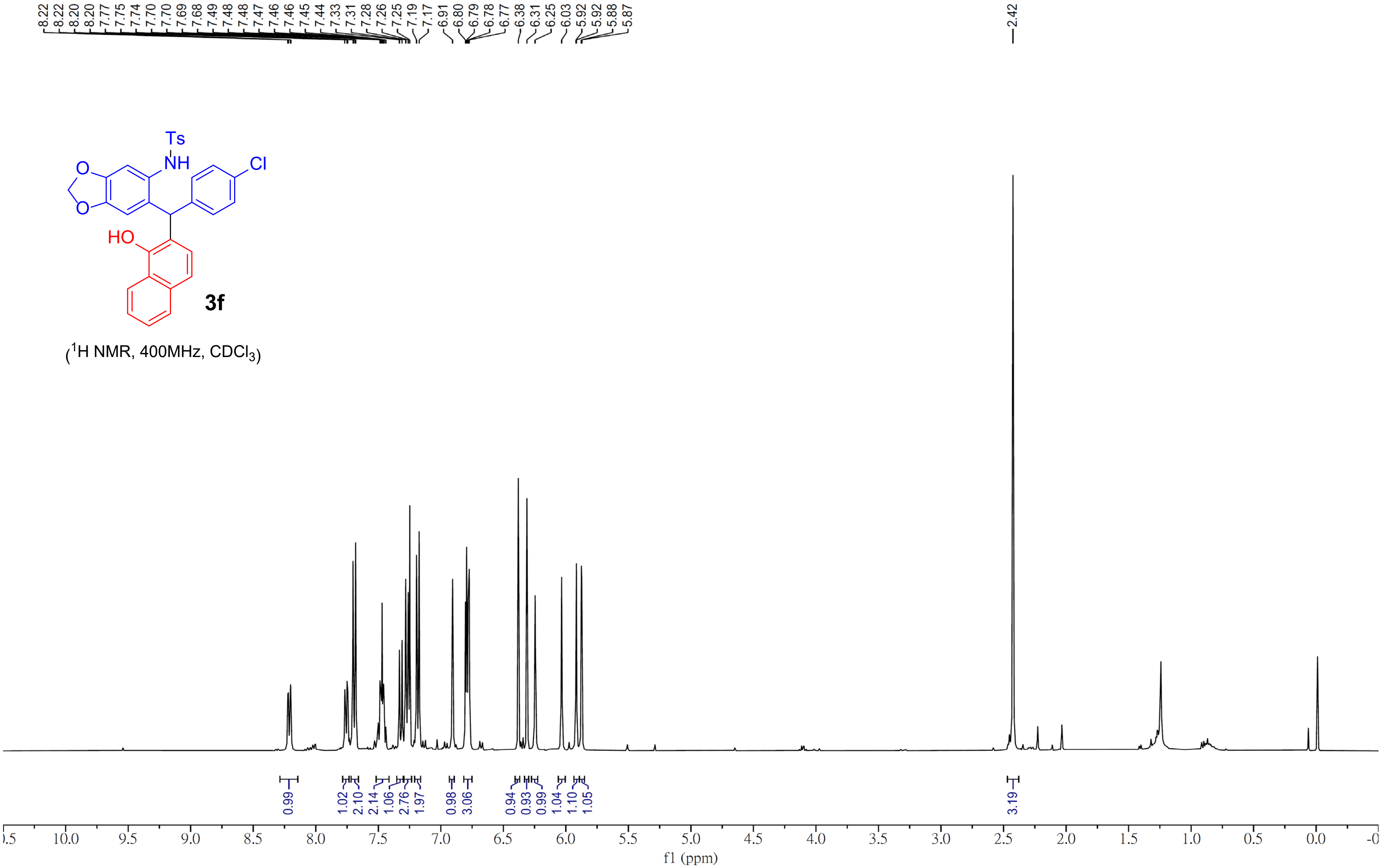

3f

(^{1}H NMR, 400MHz, $CDCl_3$)

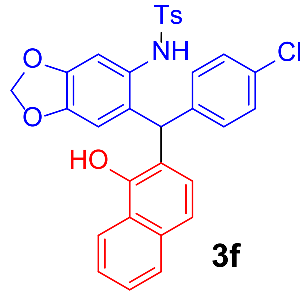

3f

($^{13}C\{^{1}H\}$ NMR, 101 MHz, $CDCl_3$)

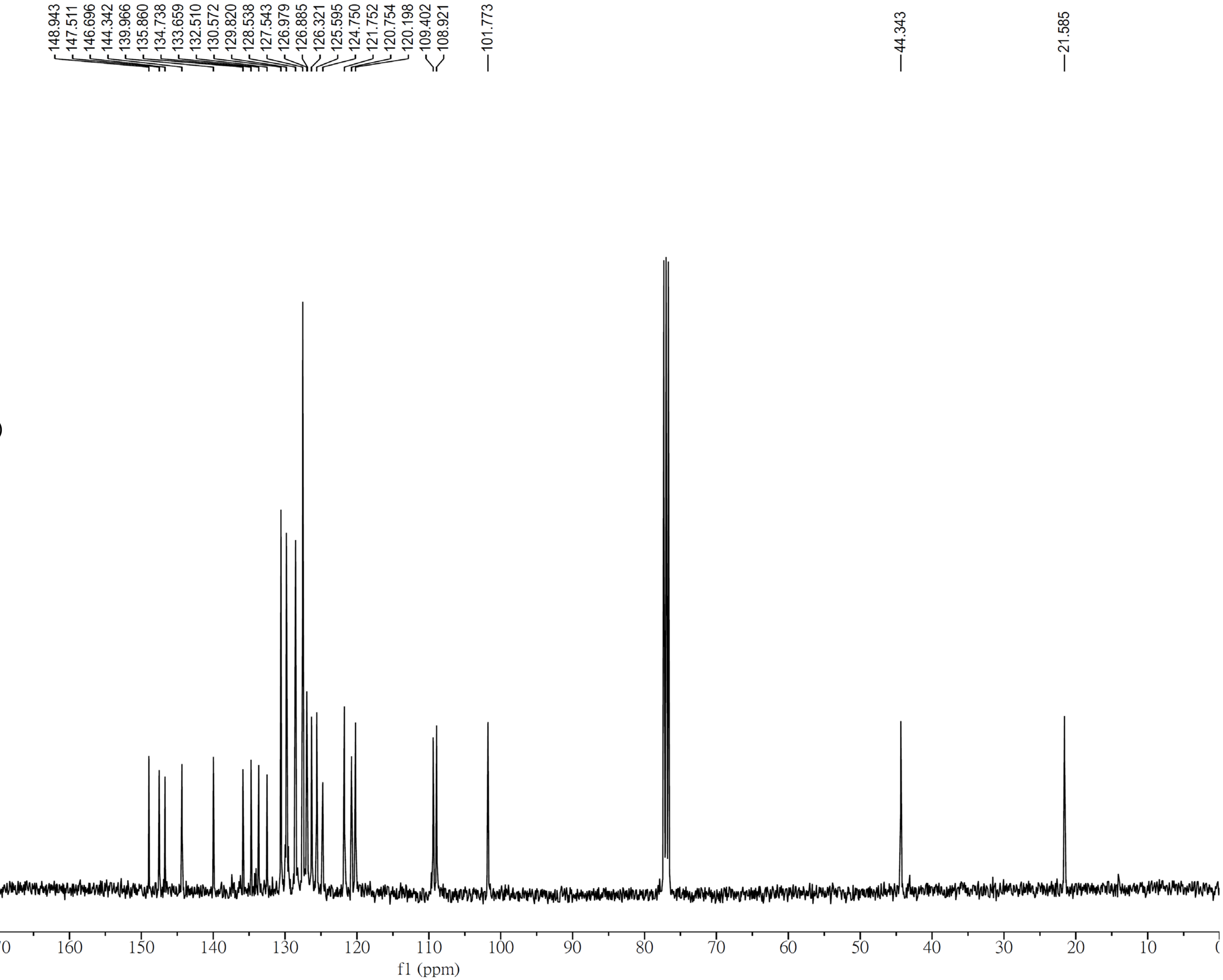

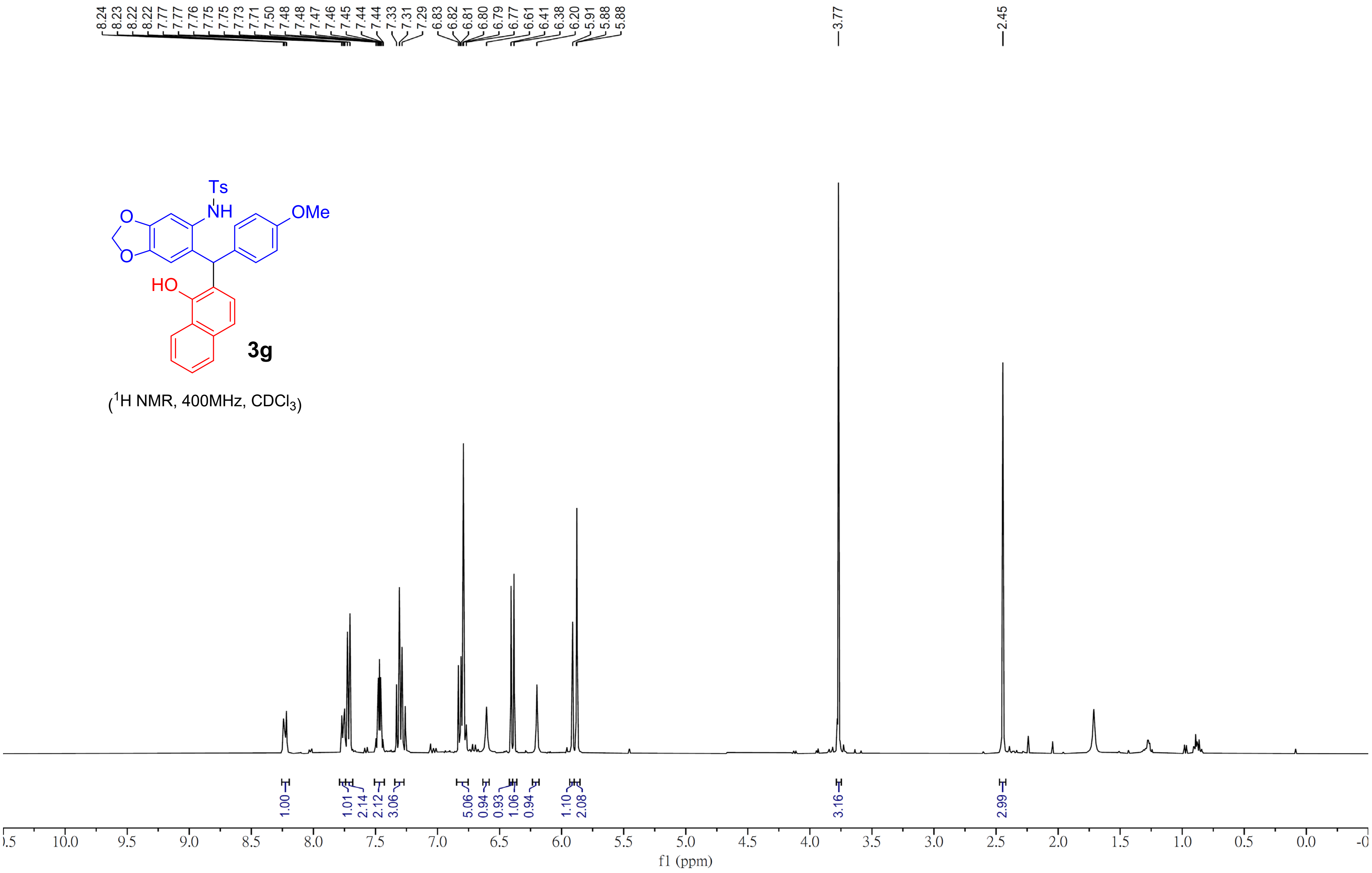
Ts
NH
O
O
OMe
HO
3g
(1H NMR, 400MHz, CDCl3)
f1 (ppm)

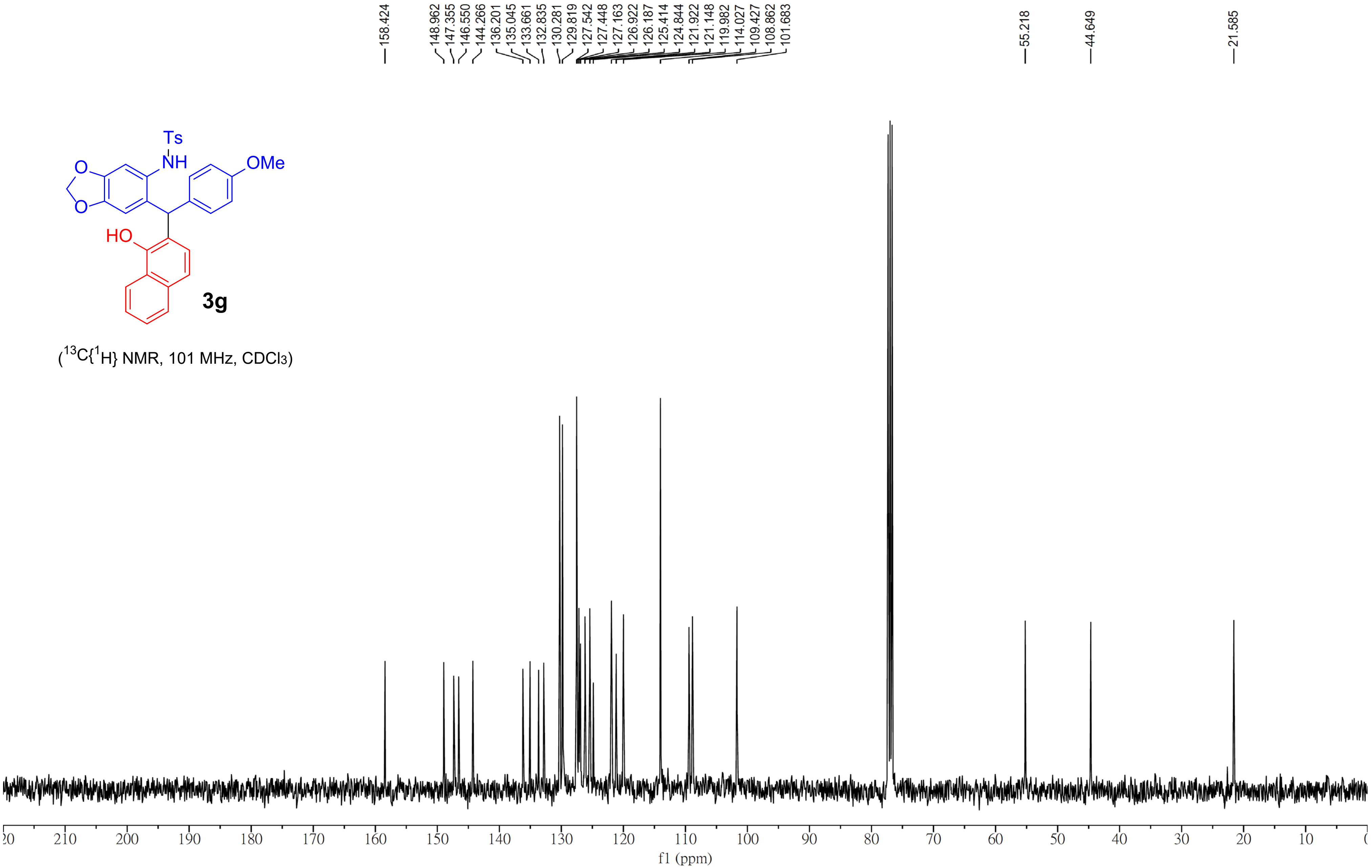
158.424
148.962
147.355
146.550
144.266
136.201
135.045
133.661
132.835
130.281
129.819
127.542
127.448
127.163
126.922
126.187
125.414
124.844
121.922
121.148
119.982
114.027
109.427
108.862
101.683
55.218
44.649
21.585
Ts
NH
OMe
O
O
HO
3g
($^{13}C\{^1H\}$ NMR, 101 MHz, $CDCl_3$)
f1 (ppm)

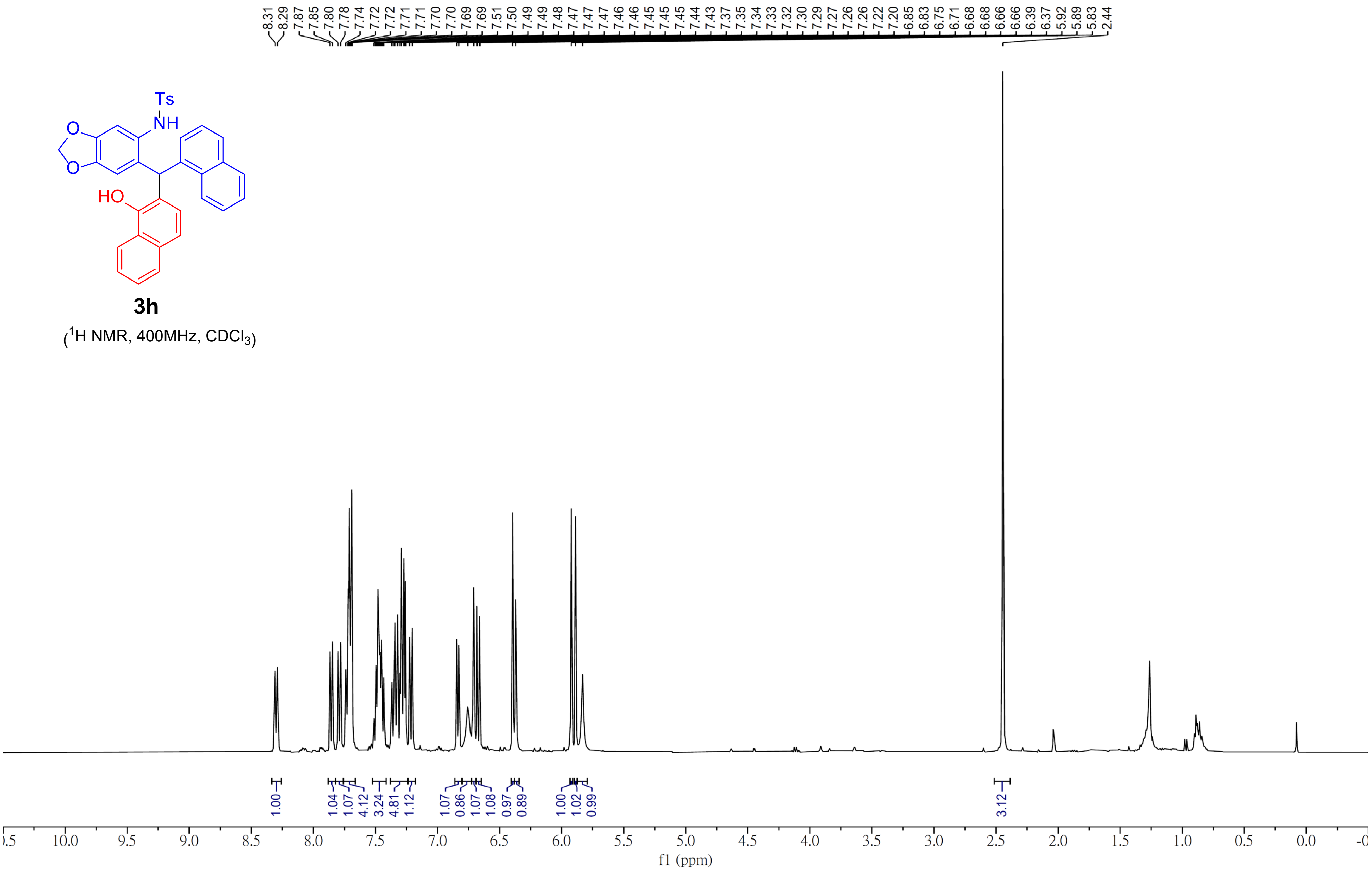
Ts
NH
O
O
HO
3h
(1H NMR, 400MHz, CDCl3)
f1 (ppm)

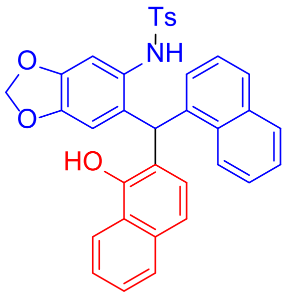

3h

($^{13}C\{^1H\}$ NMR, 101 MHz, $CDCl_3$)

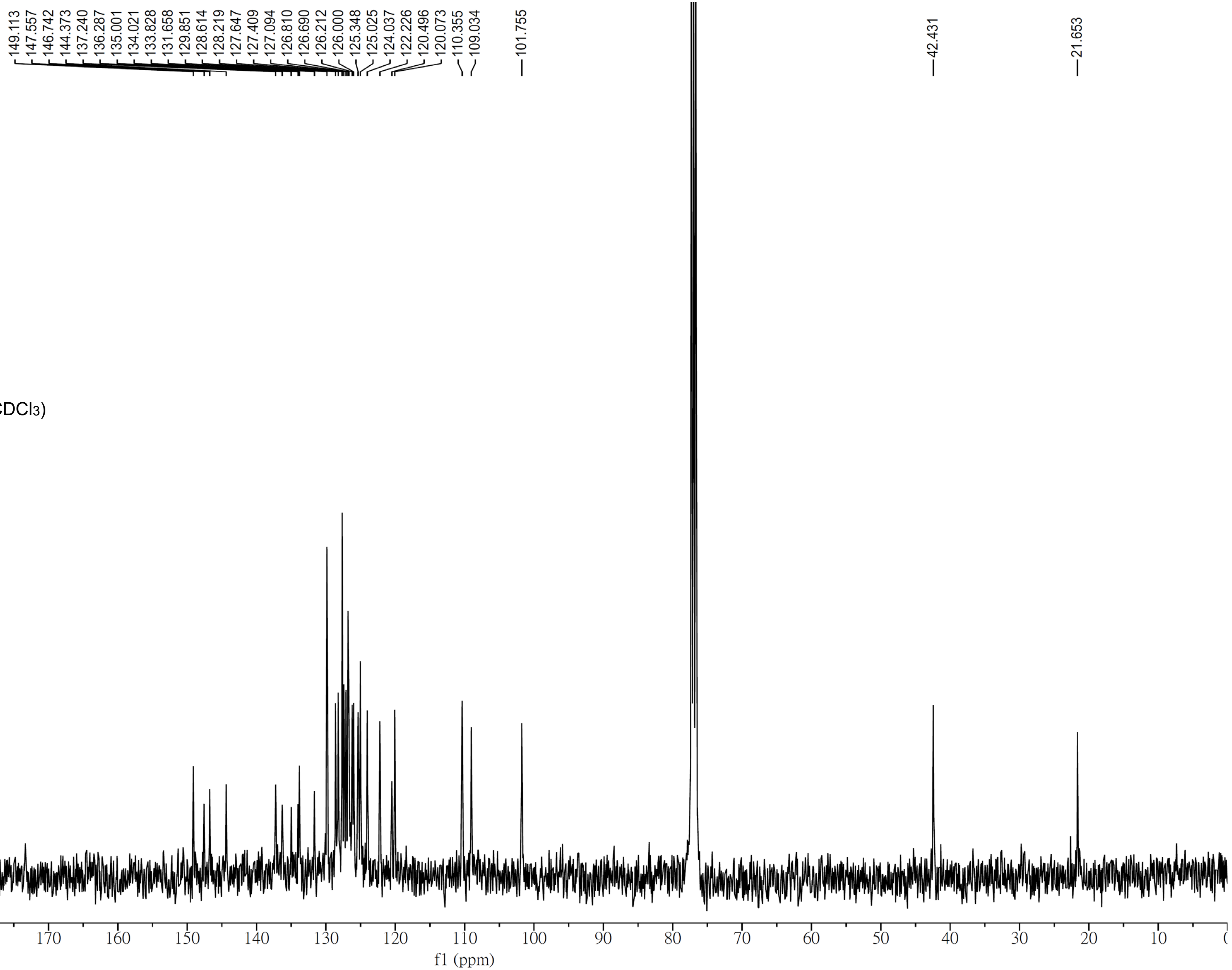

R=m-up-2.jdf

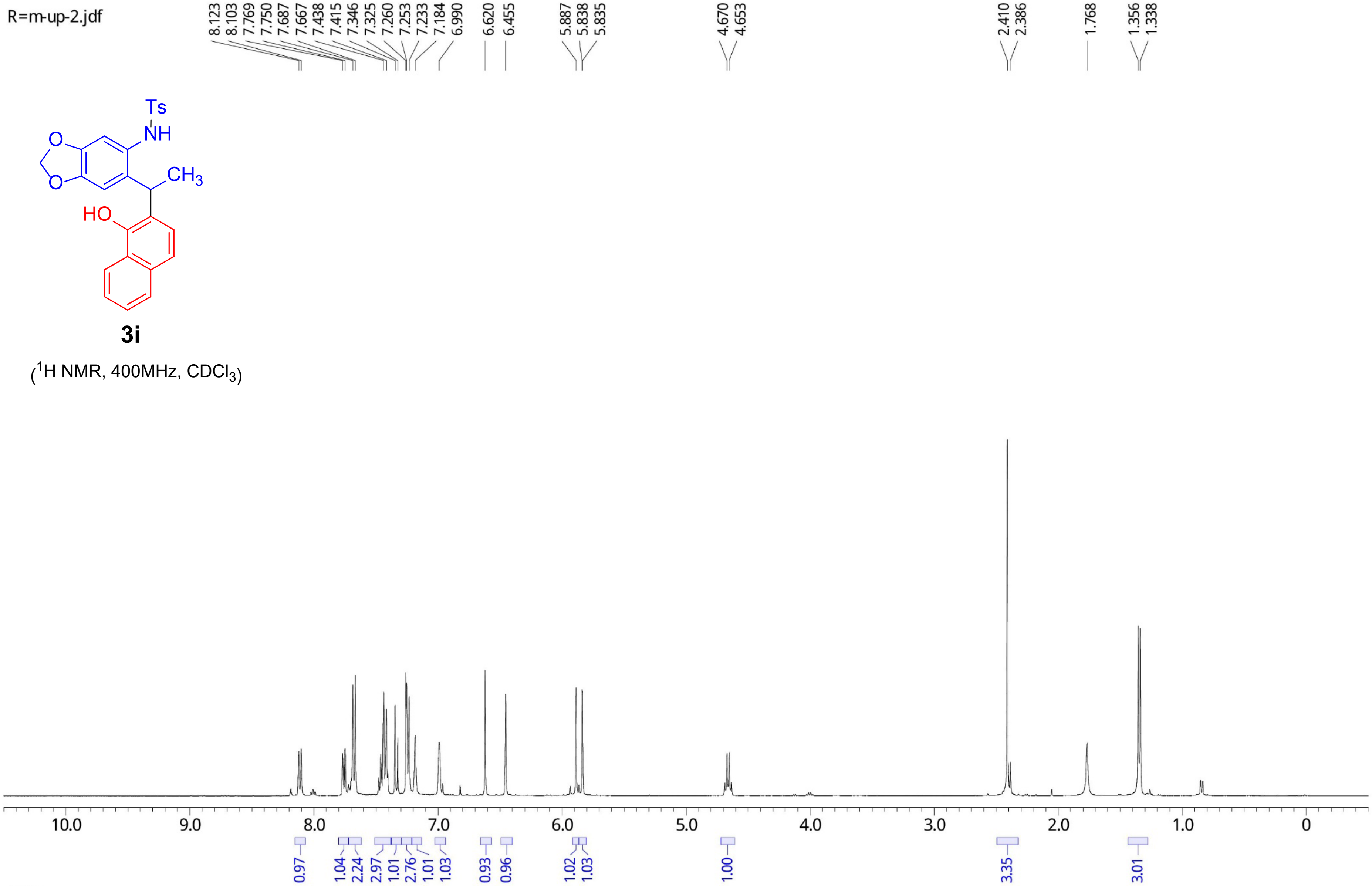

3i

(^{1}H NMR, 400MHz, $CDCl_3$)

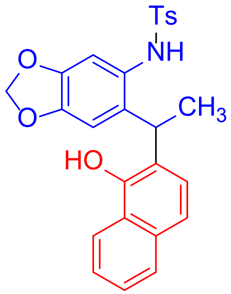

3i

($^{13}C\{^{1}H\}$ NMR, 101 MHz, $CDCl_3$)

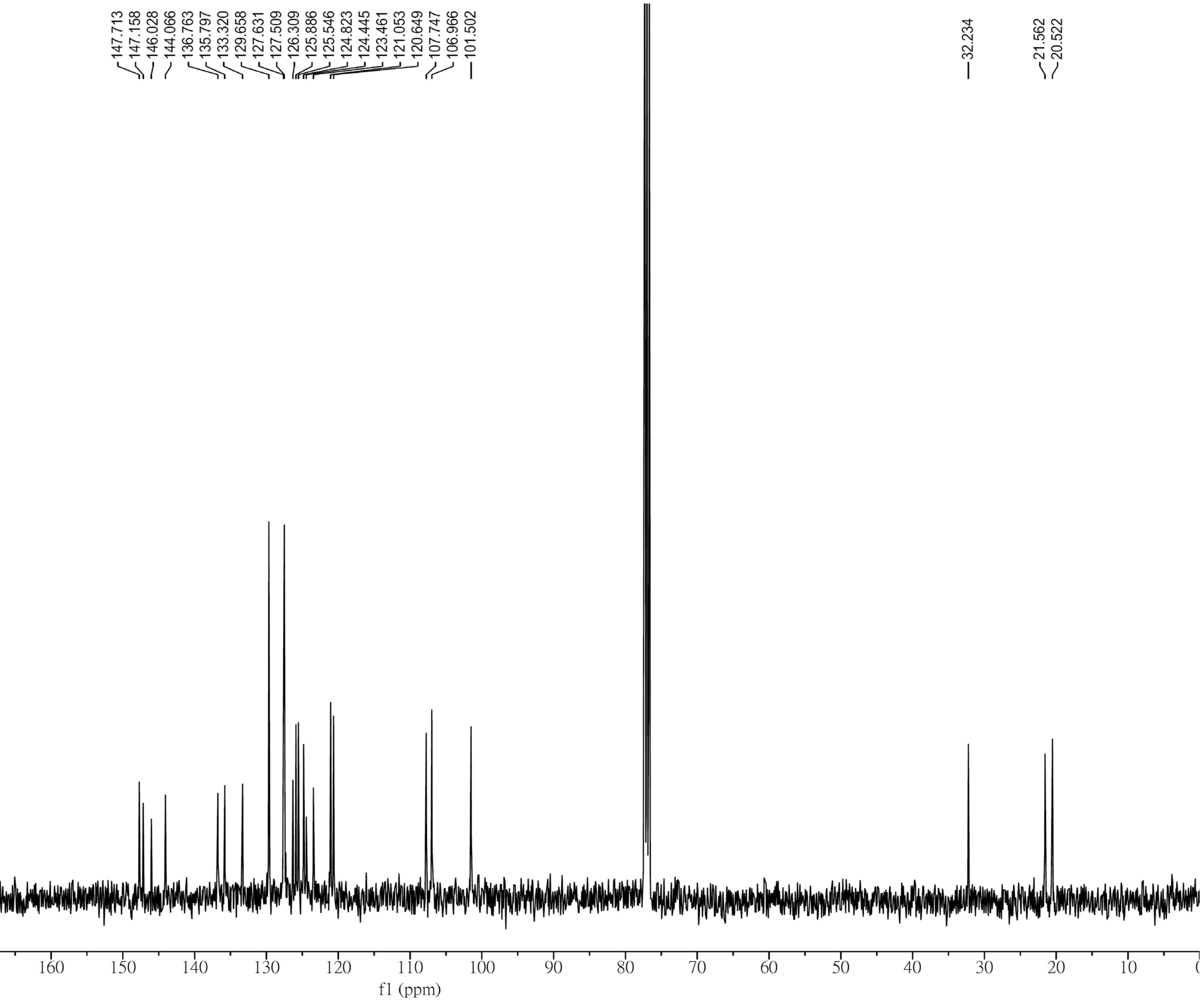

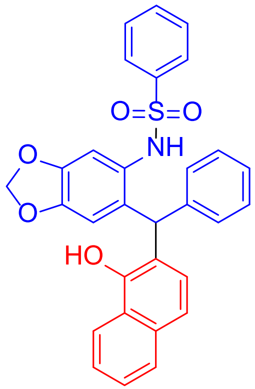

3j
(^{1}H NMR, 400MHz, $CDCl_3$)

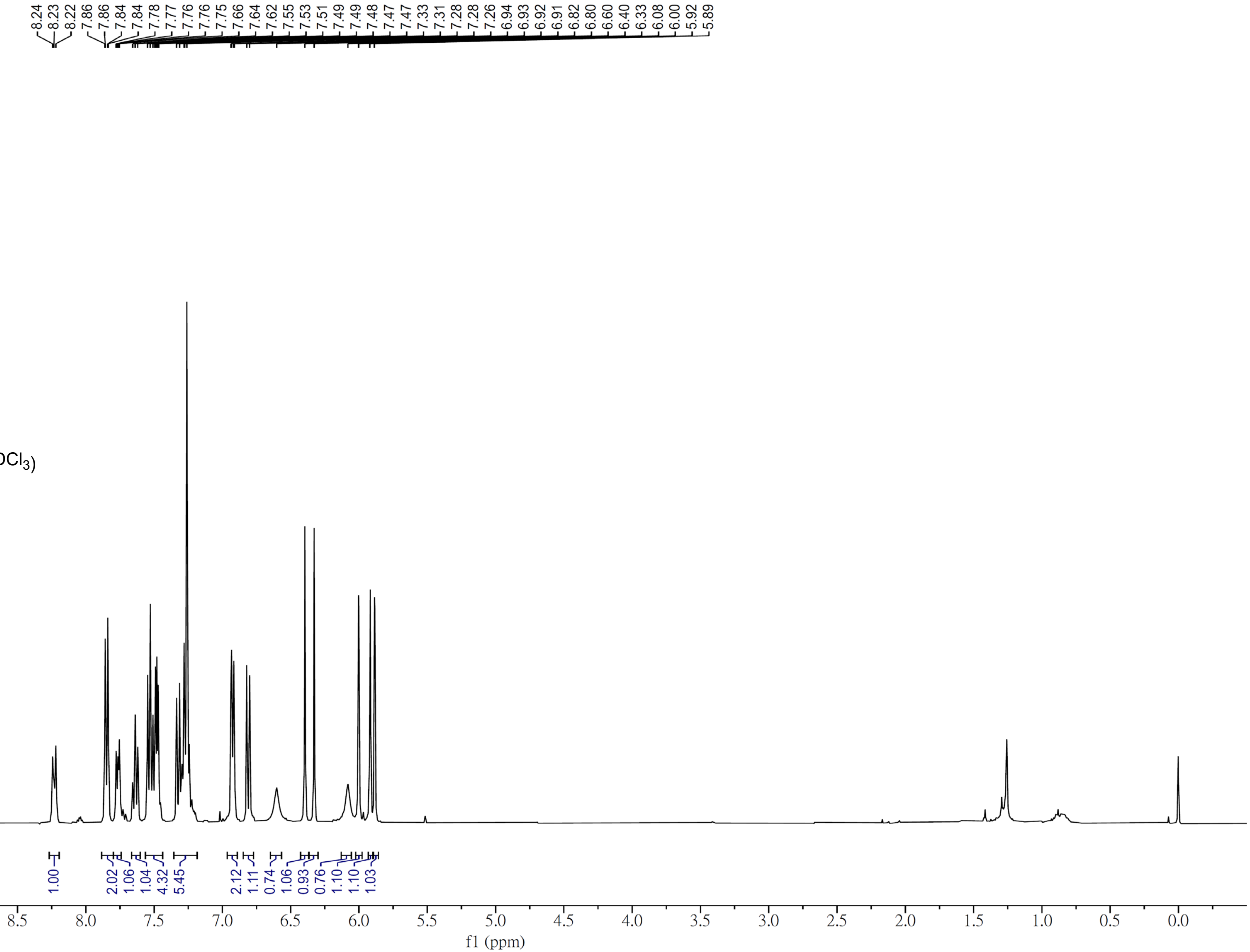

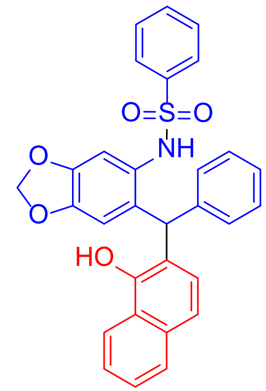

3j

($^{13}C\{^1H\}$ NMR, 101 MHz, $CDCl_3$)

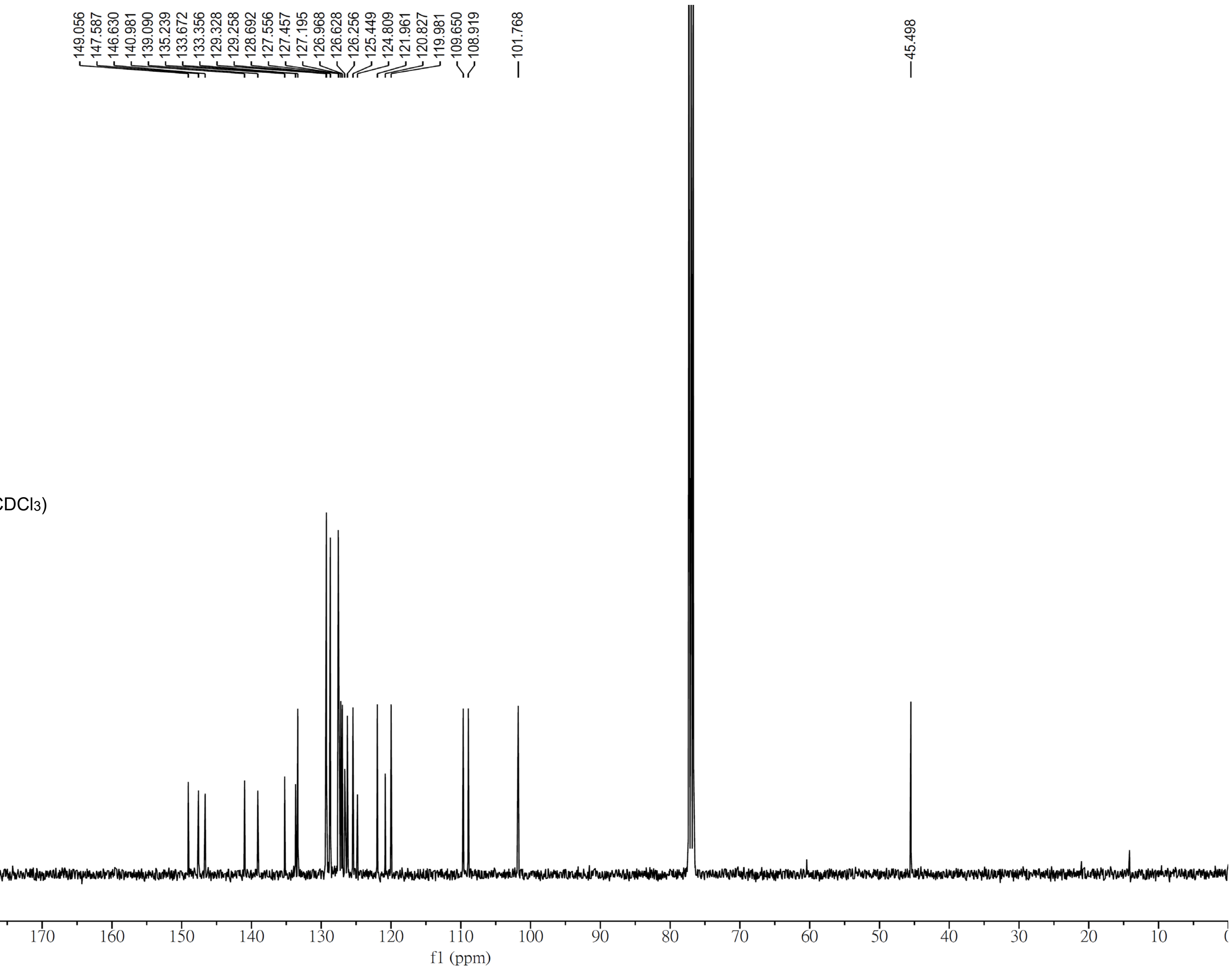

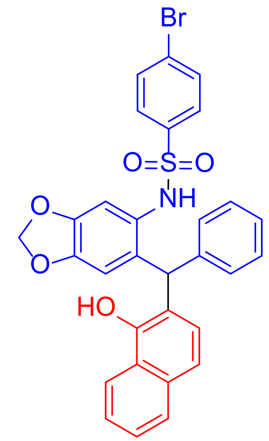

3k

(^{1}H NMR, 400MHz, $CDCl_3$)

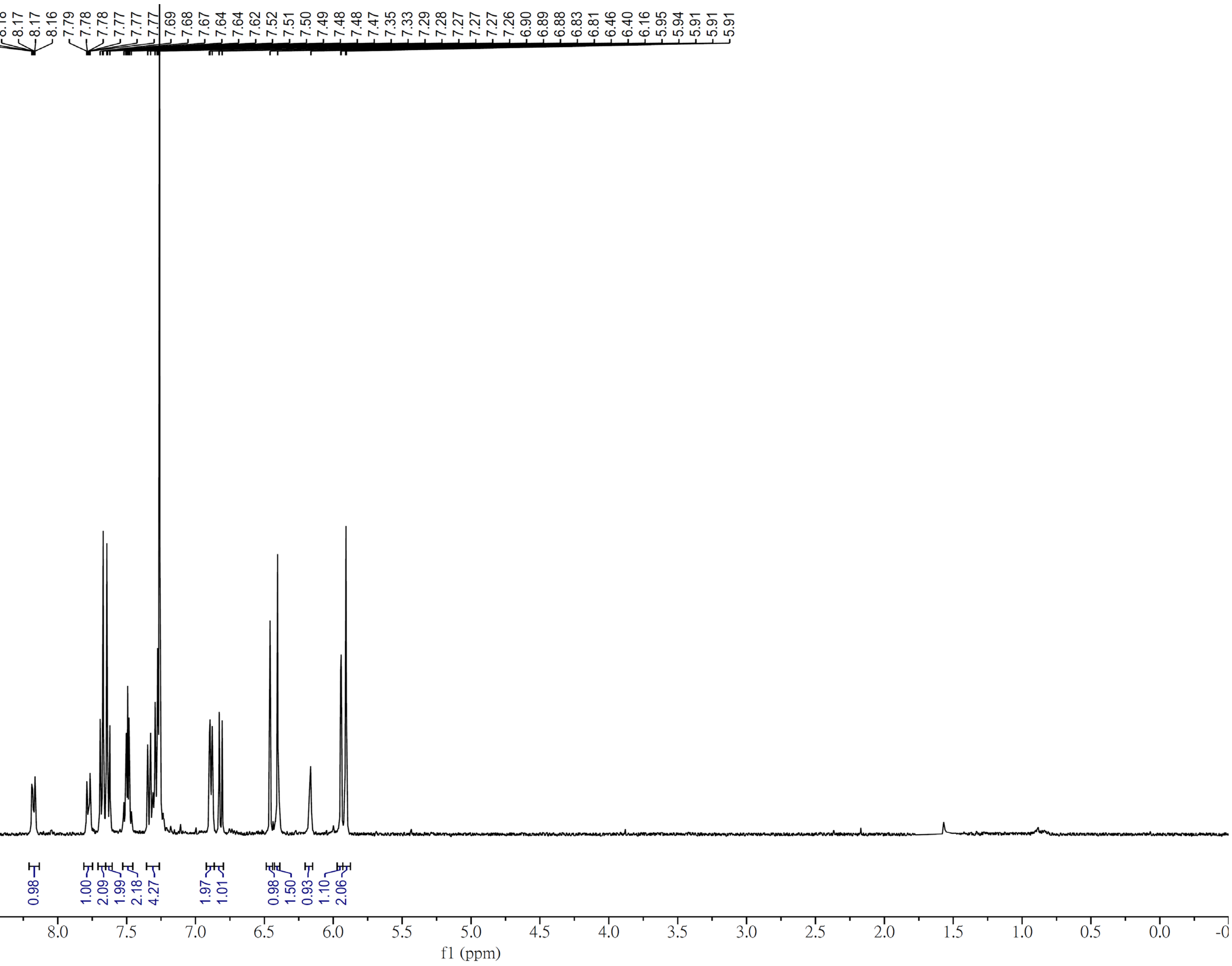

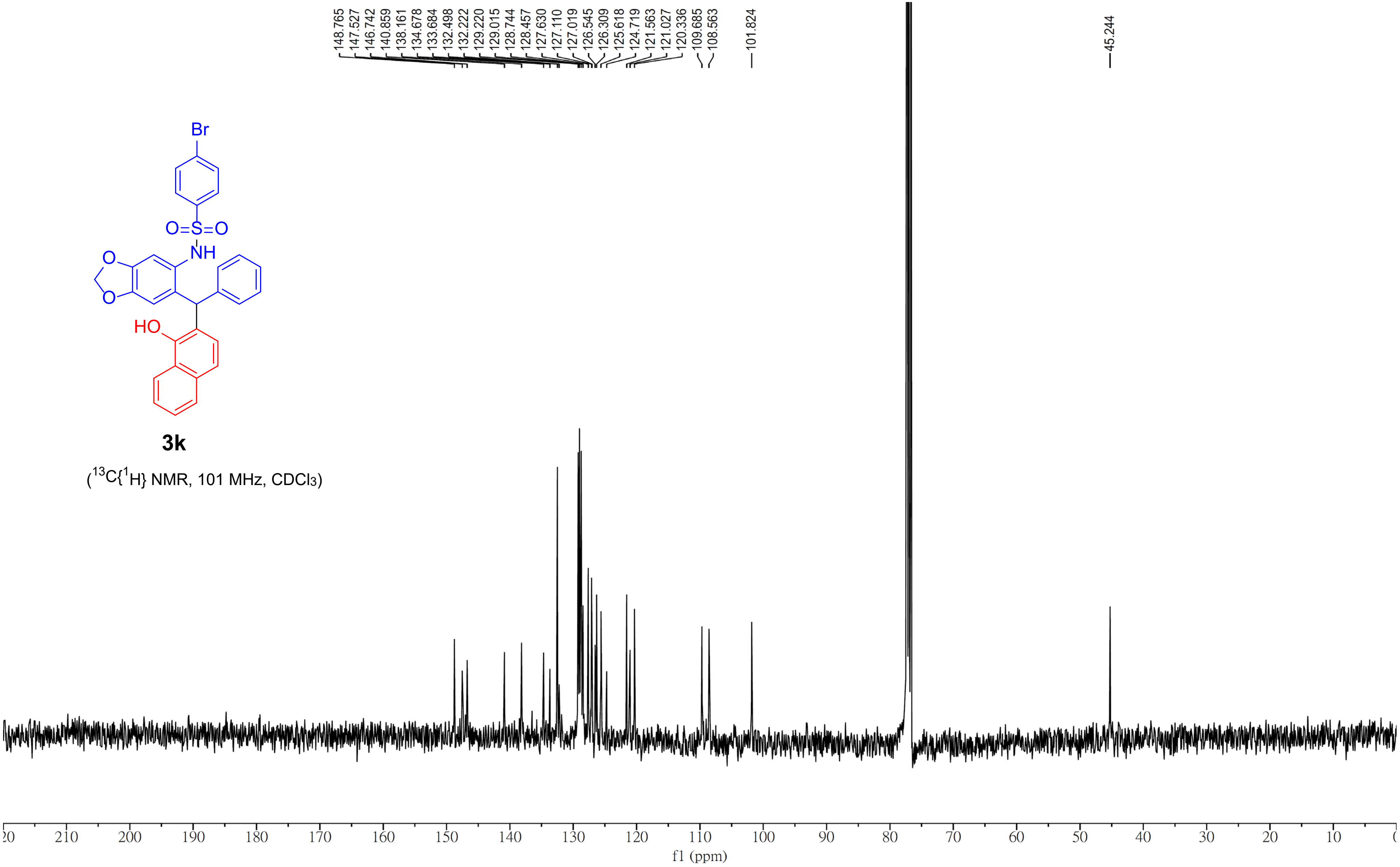

3k

($^{13}C\{^{1}H\}$ NMR, 101 MHz, $CDCl_3$)

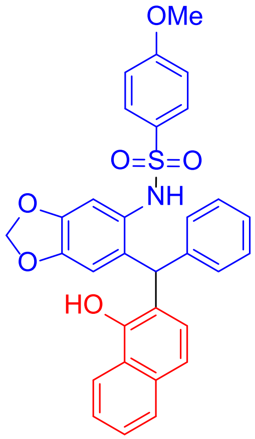

3l

(^{1}H NMR, 400MHz, CDCl$_3$)

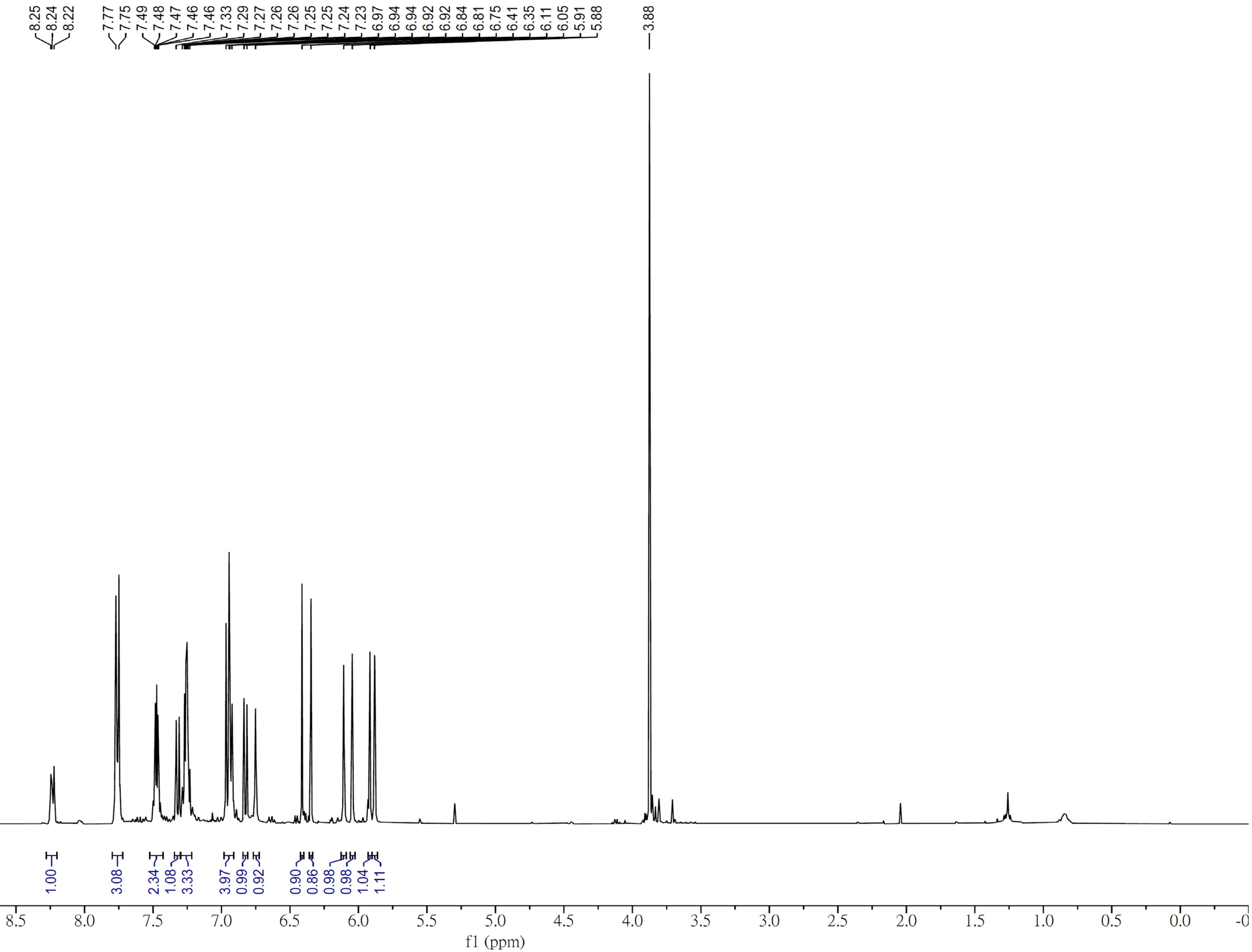

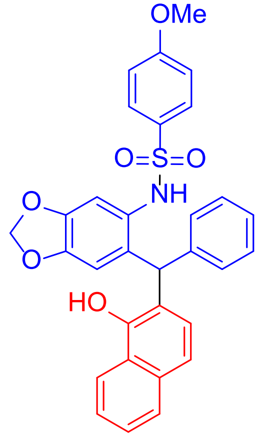

3l

($^{13}C\{^{1}H\}$ NMR, 101 MHz, $CDCl_3$)

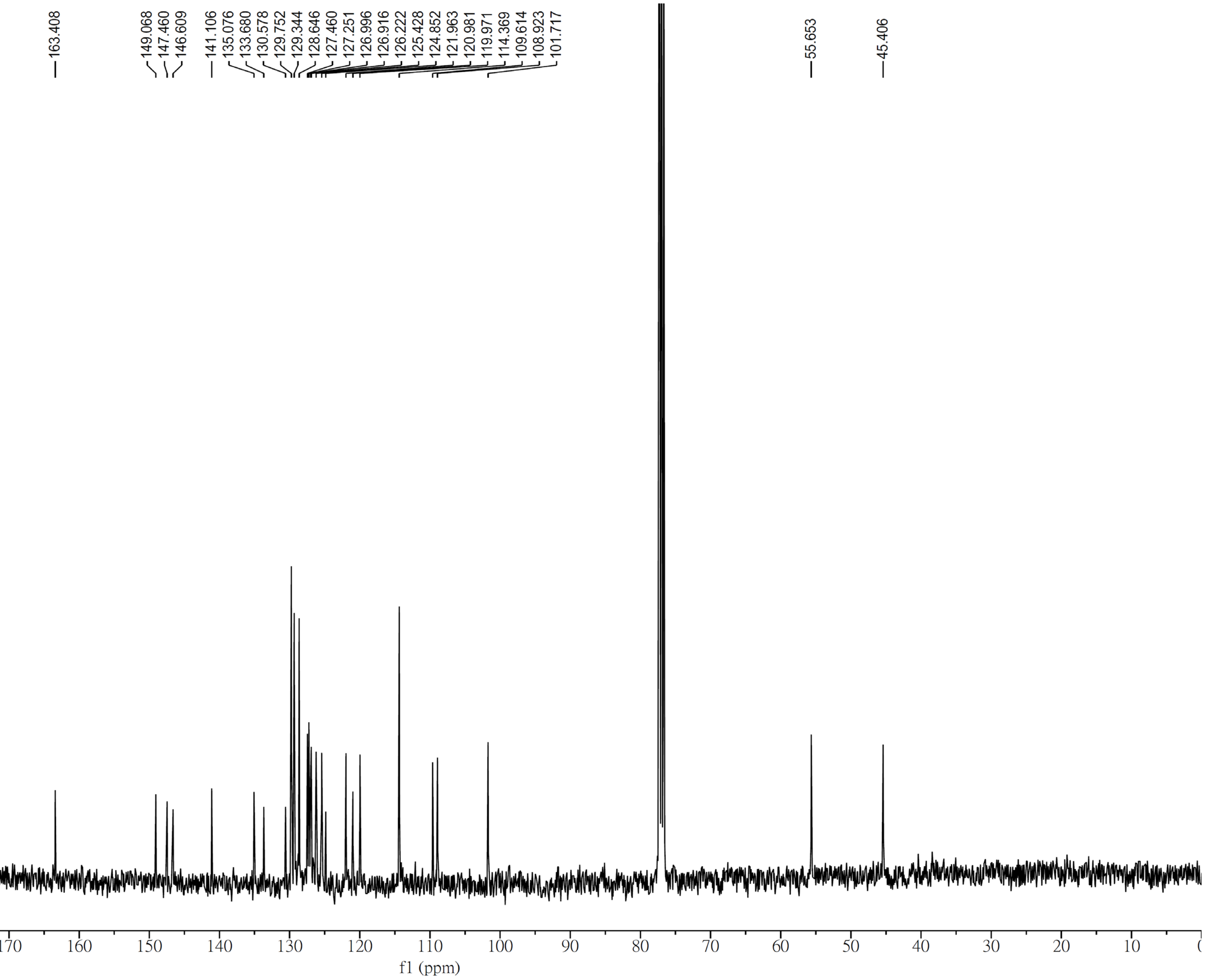

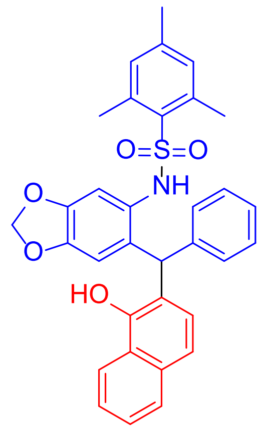

3m

(^{1}H NMR, 400MHz, $CDCl_3$)

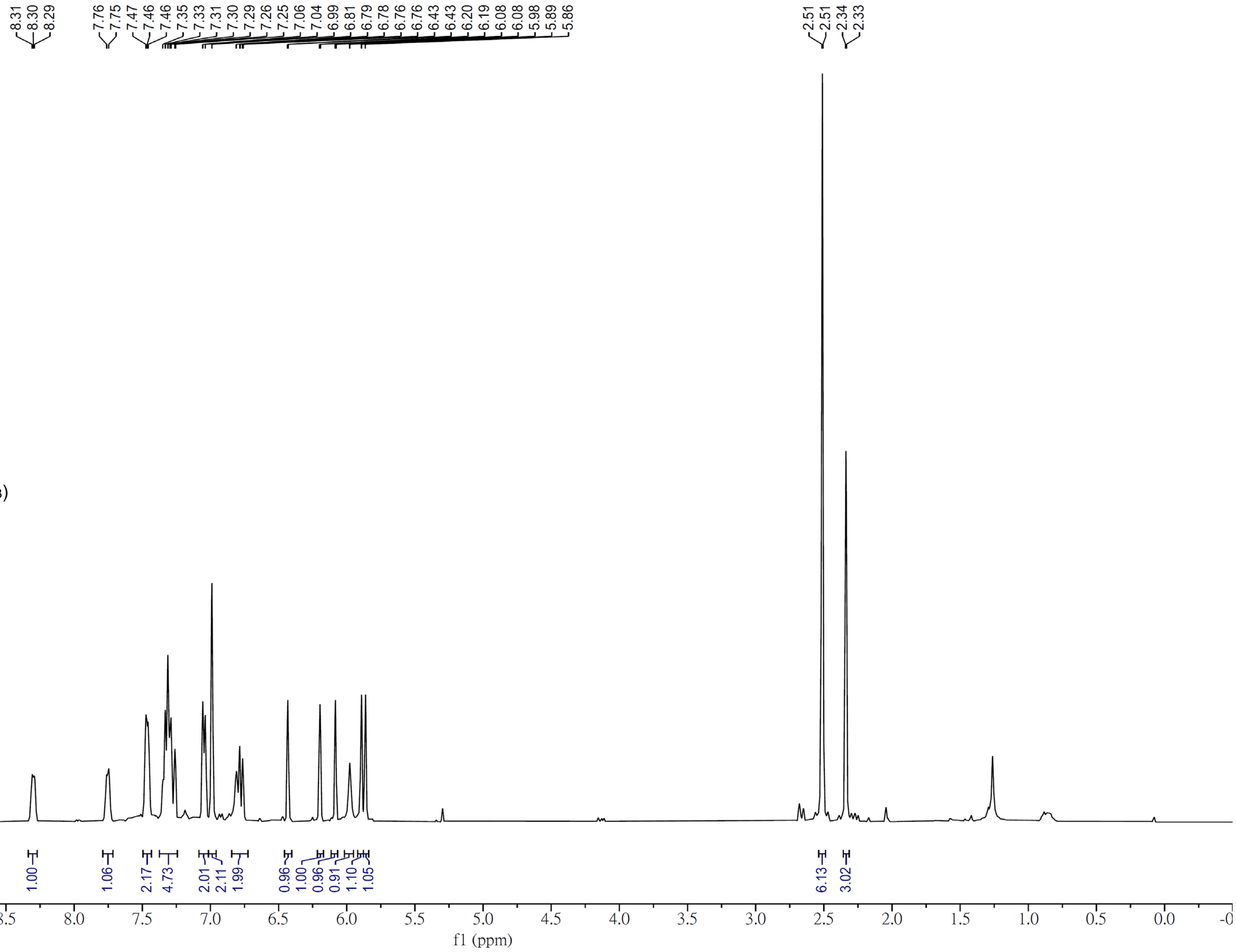

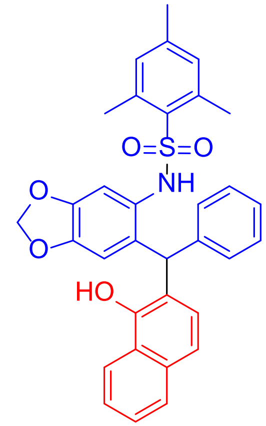

3m

($^{13}C\{^1H\}$ NMR, 101 MHz, $CDCl_3$)

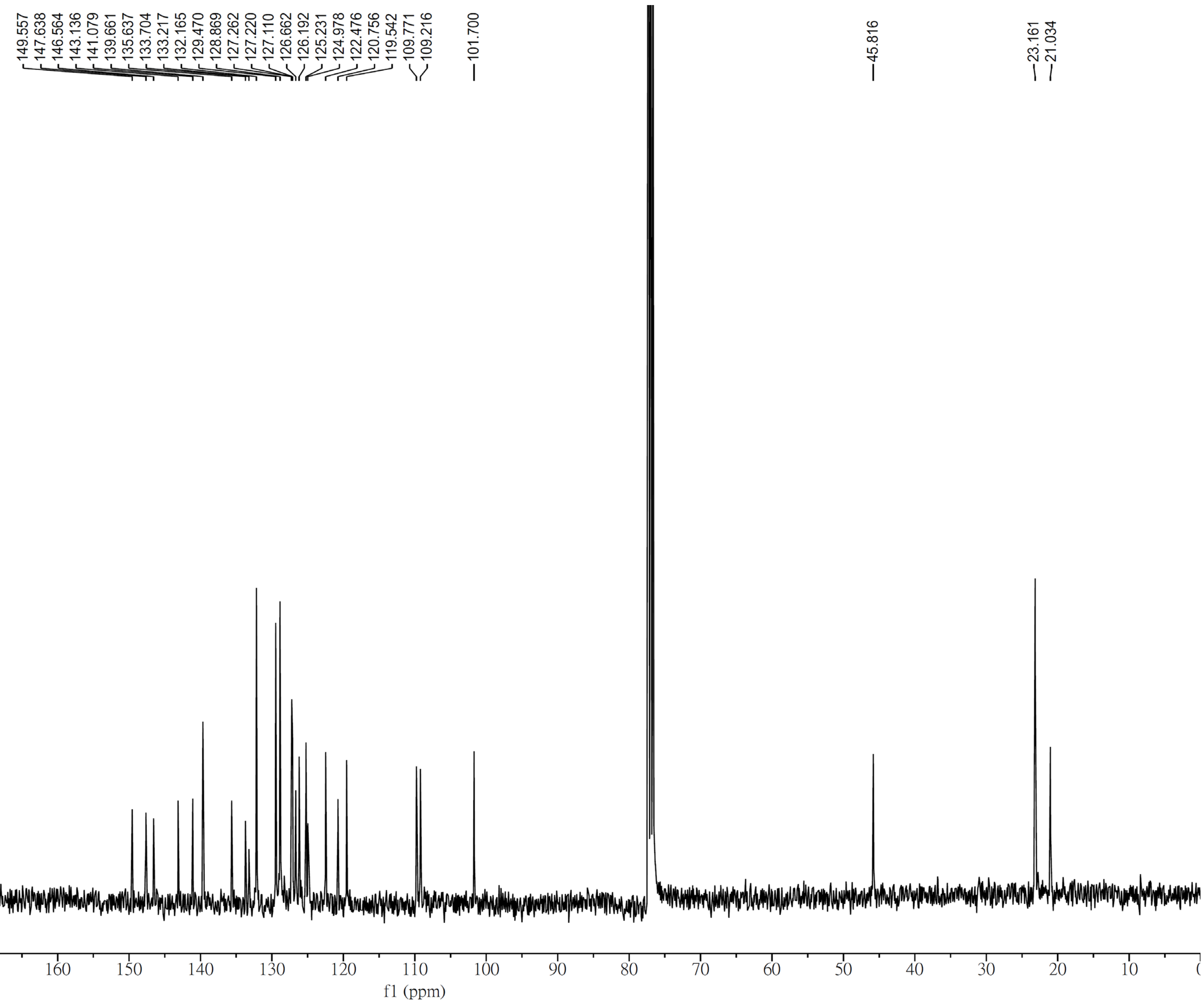

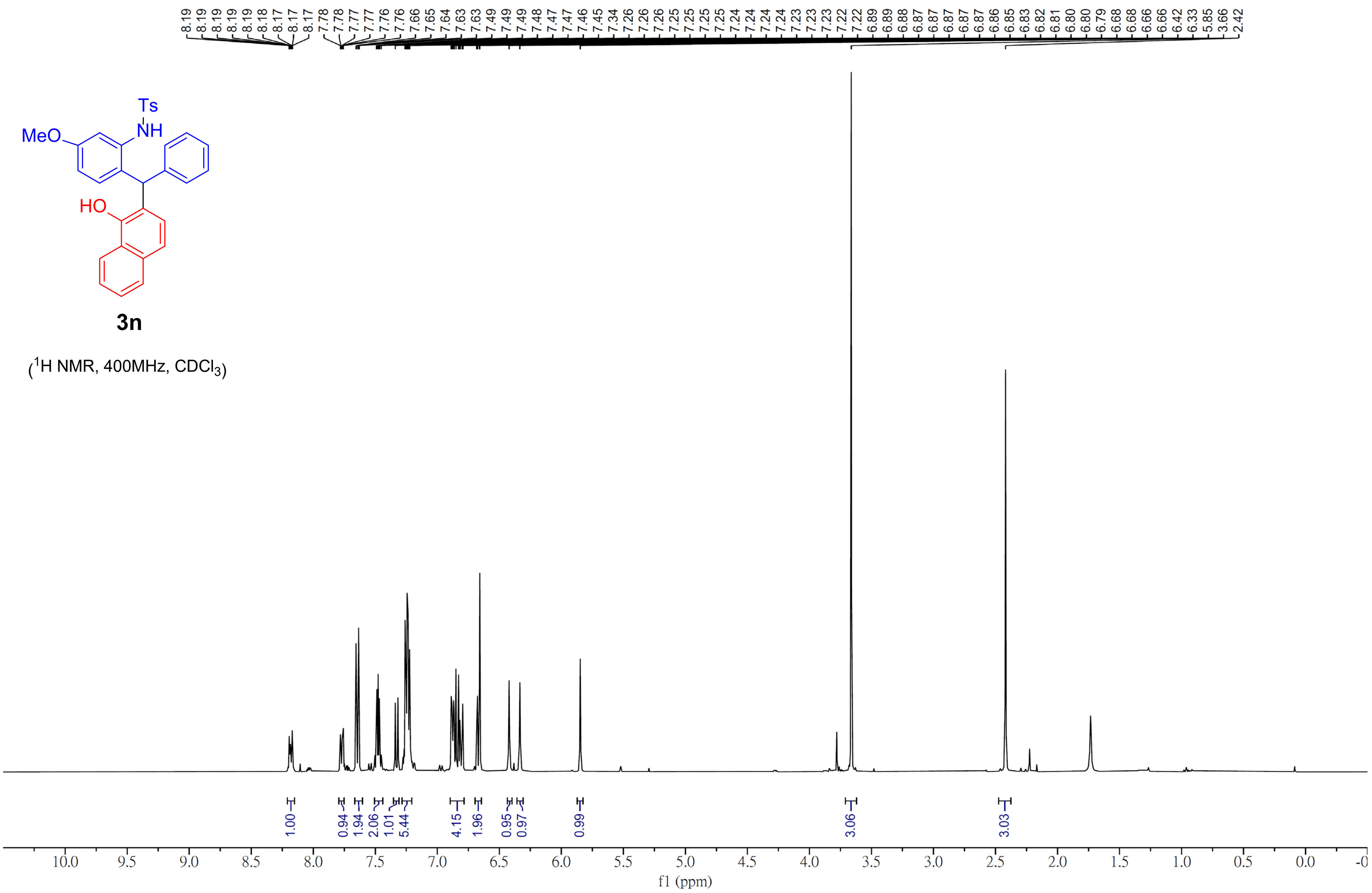

MeO
Ts
NH
HO
3n
(1H NMR, 400MHz, CDCl3)
8.19 8.19 8.19 8.19 8.19 8.18 8.17 8.17 8.17 7.78 7.78 7.77 7.77 7.76 7.76 7.66 7.65 7.64 7.63 7.63 7.49 7.49 7.49 7.48 7.47 7.47 7.46 7.45 7.34 7.26 7.26 7.26 7.25 7.25 7.25 7.25 7.24 7.24 7.24 7.24 7.23 7.23 7.23 7.22 7.22 6.89 6.89 6.88 6.87 6.87 6.87 6.87 6.87 6.86 6.85 6.83 6.82 6.81 6.80 6.80 6.79 6.68 6.68 6.66 6.66 6.42 6.33 5.85 3.66 2.42
1.00 0.94 1.94 2.06 1.01 5.44 4.15 1.96 0.95 0.97 0.99 3.06 3.03
10.0 9.5 9.0 8.5 8.0 7.5 7.0 6.5 6.0 5.5 5.0 4.5 4.0 3.5 3.0 2.5 2.0 1.5 1.0 0.5 0.0
f1 (ppm)

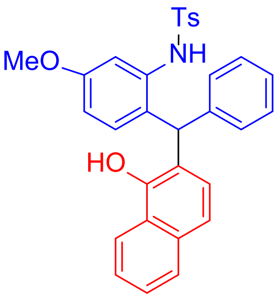

3n

($^{13}C\{^1H\}$ NMR, 101 MHz, $CDCl_3$)

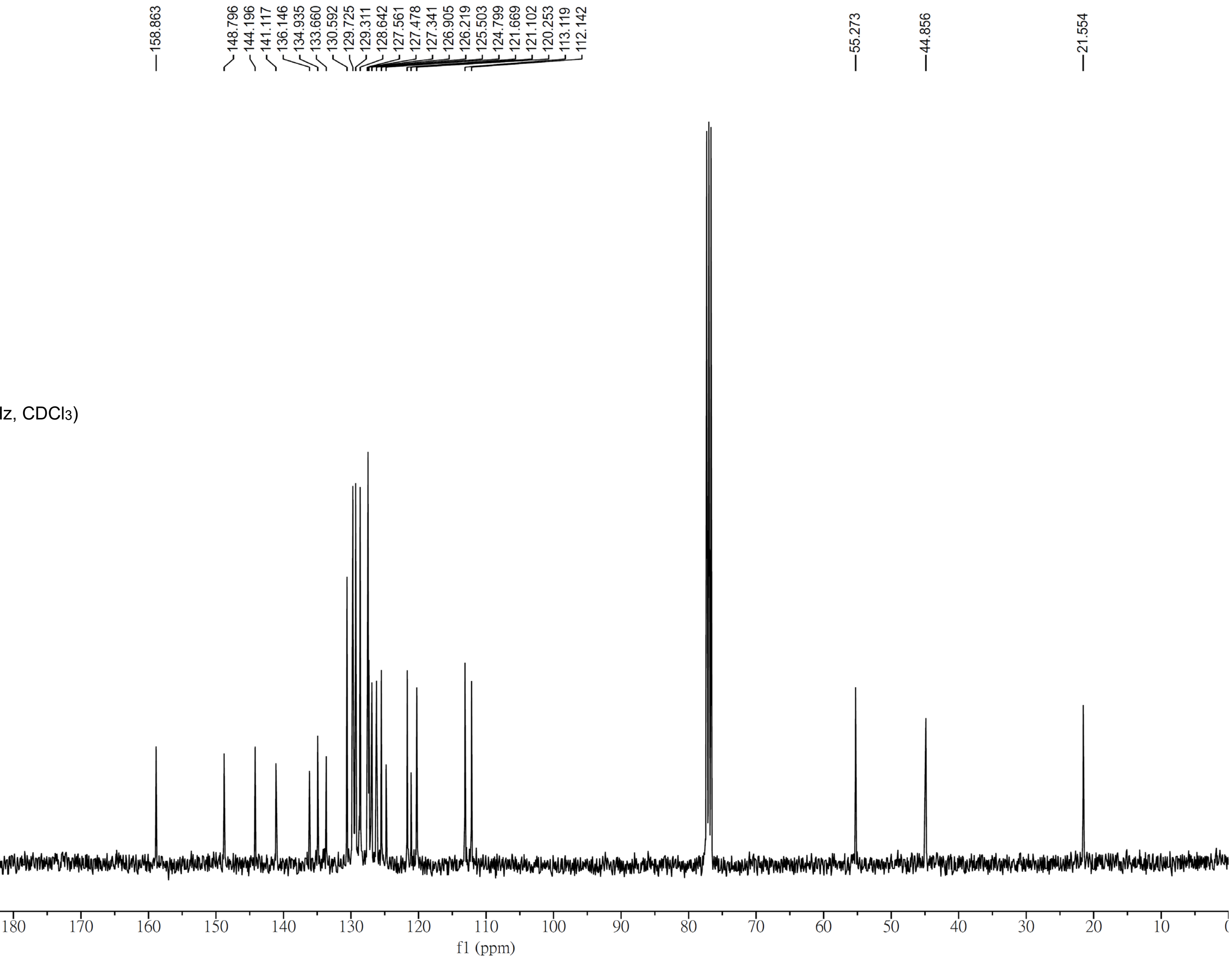

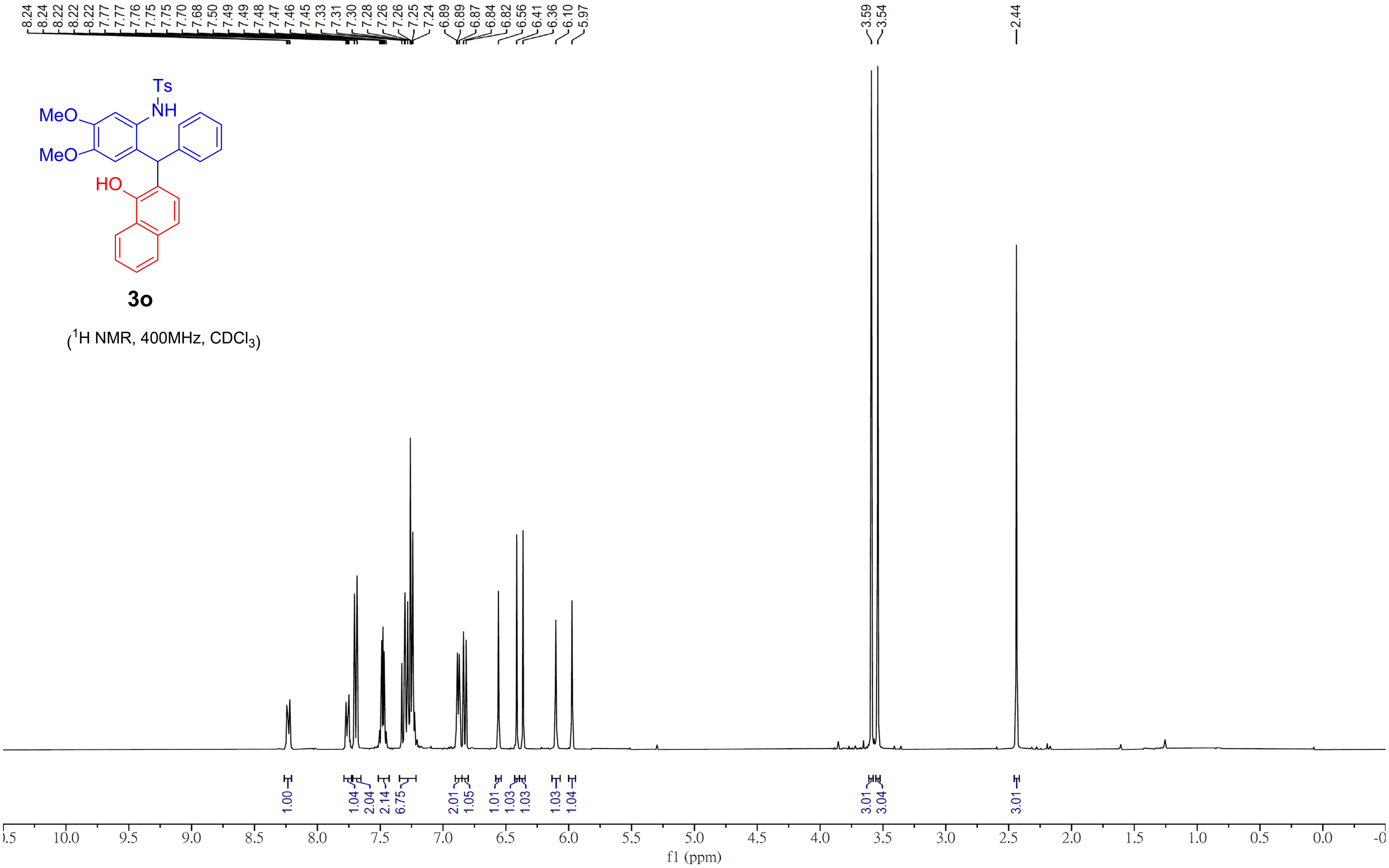
8.24
8.24
8.22
8.22
8.22
7.77
7.77
7.76
7.75
7.75
7.70
7.68
7.50
7.49
7.49
7.48
7.47
7.46
7.45
7.33
7.31
7.30
7.28
7.26
7.26
7.25
7.24
6.89
6.89
6.87
6.84
6.82
6.56
6.41
6.36
6.10
5.97
3.59
3.54
2.44
MeO
MeO
Ts
NH
HO
3o
(^{1}H NMR, 400MHz, CDCl$_3$)
1.00
1.04
2.04
2.14
6.75
2.01
1.05
1.01
1.03
1.03
1.03
1.04
3.01
3.04
3.01
f1 (ppm)

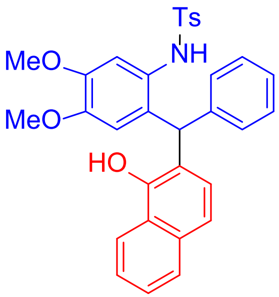

3o

($^{13}C\{^{1}H\}$ NMR, 101 MHz, $CDCl_3$)

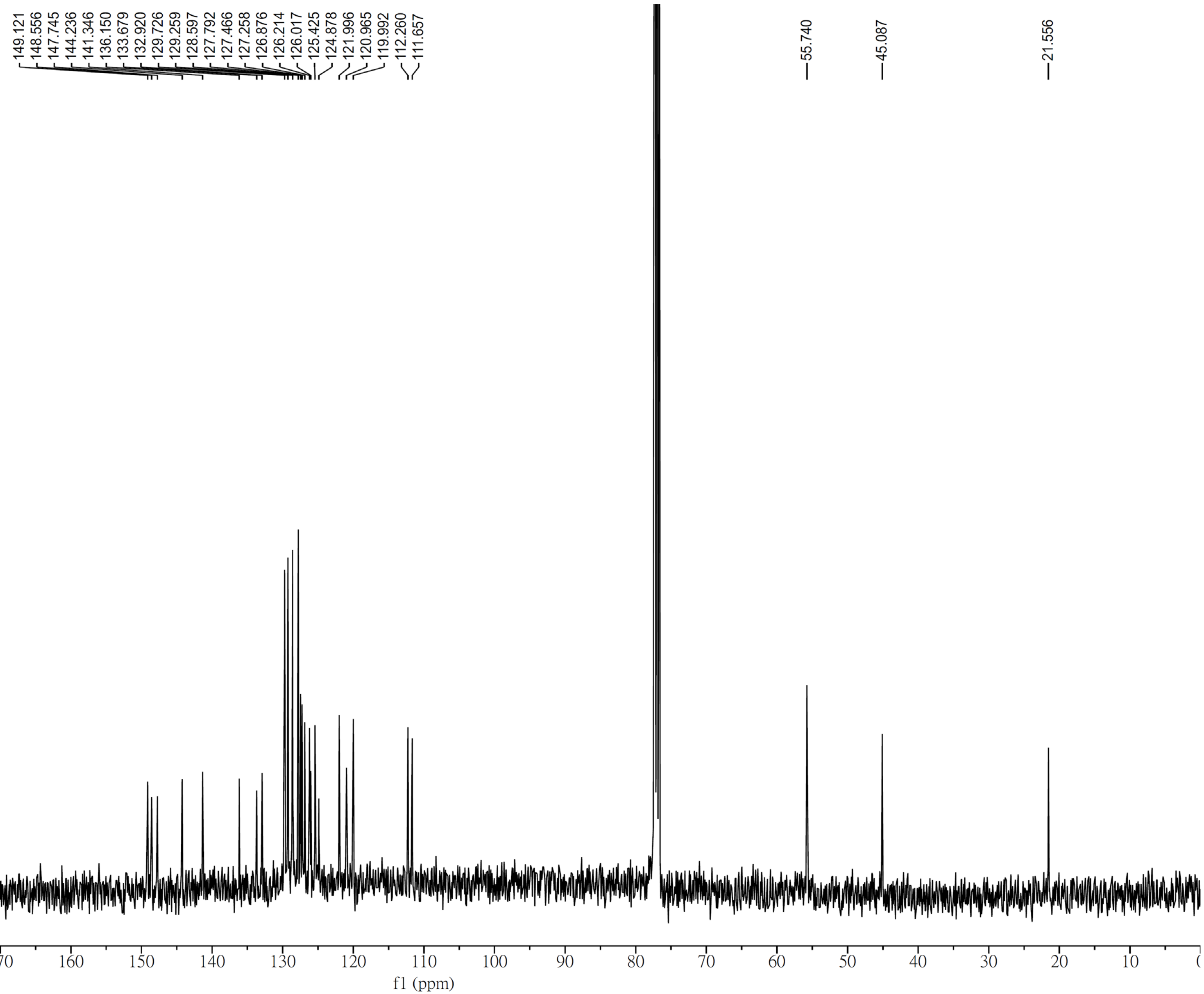

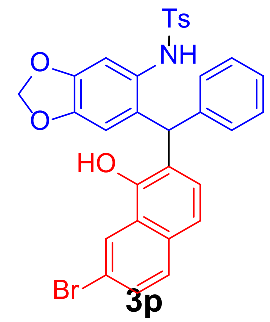

3p

(^{1}H NMR, 400MHz, $CDCl_3$)

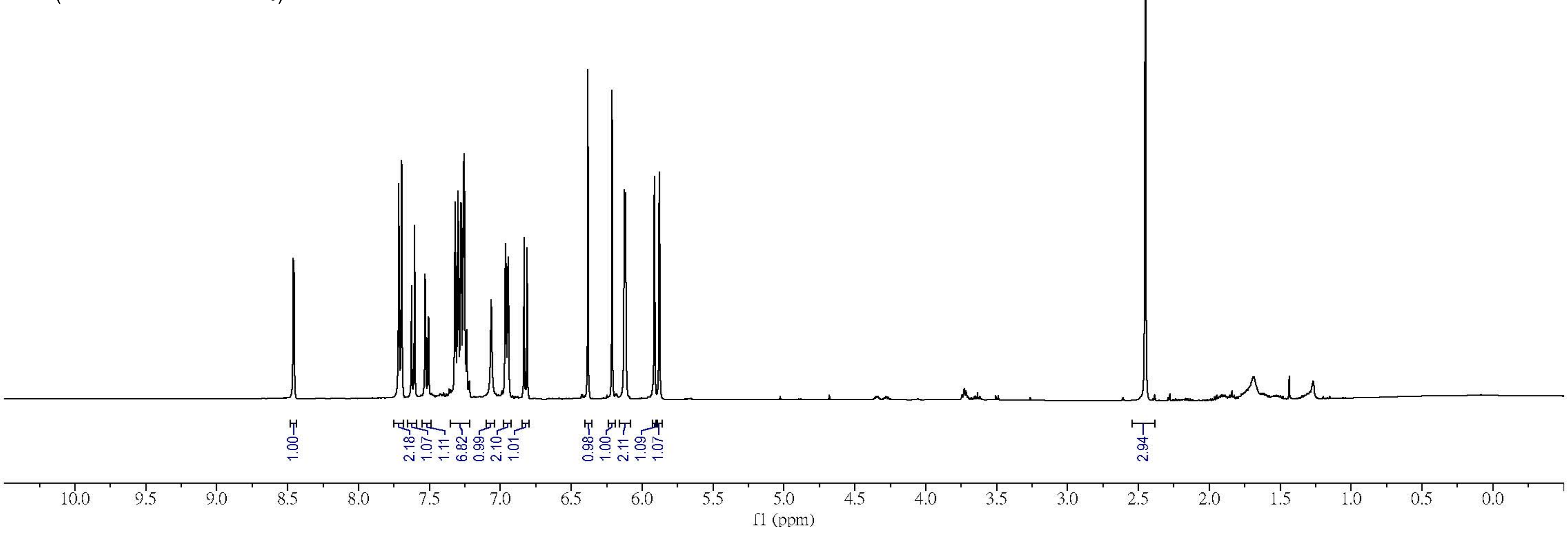

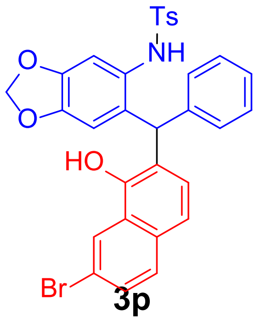

3p

($^{13}C\{^1H\}$ NMR, 101 MHz, $CDCl_3$)

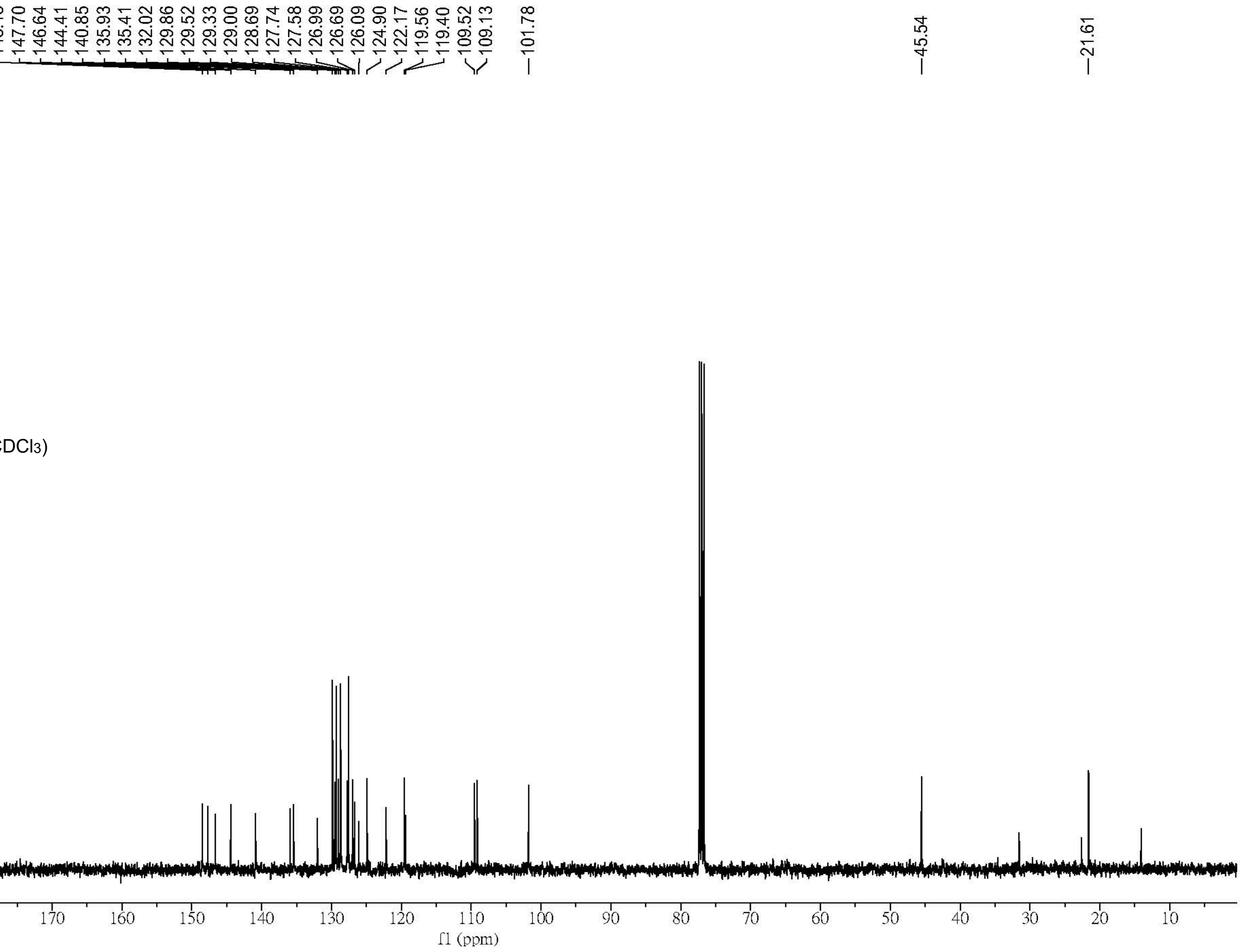

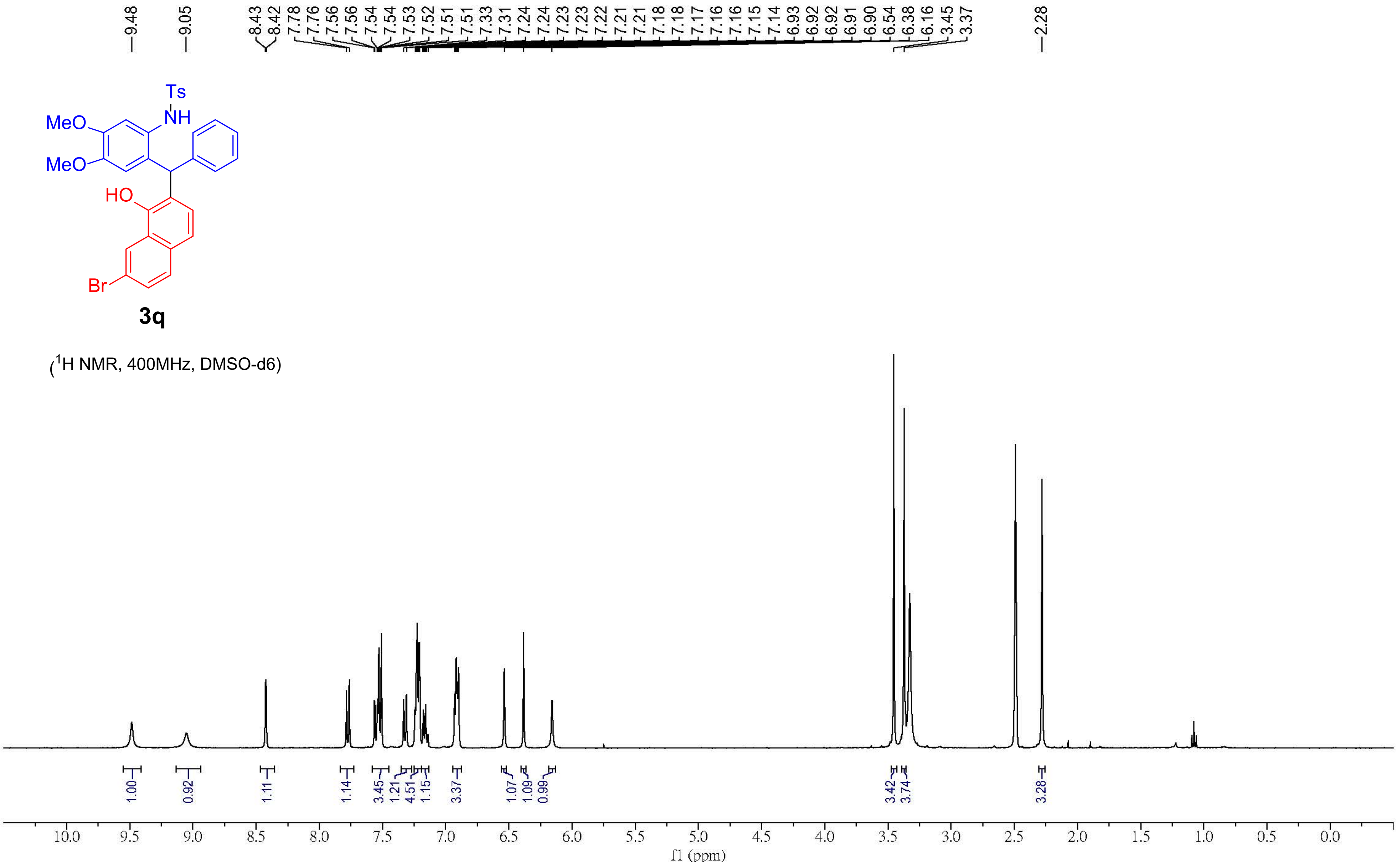

9.48
9.05
8.43 8.42 7.78 7.76 7.56 7.56 7.54 7.54 7.53 7.52 7.51 7.51 7.33 7.31 7.24 7.24 7.23 7.23 7.22 7.21 7.21 7.18 7.18 7.17 7.16 7.16 7.15 7.14 6.93 6.92 6.92 6.91 6.90 6.54 6.38 6.16 3.45 3.37
2.28
Ts
NH
MeO
MeO
HO
Br
3q
(1H NMR, 400MHz, DMSO-d6)
1.00 0.92 1.11 1.14 3.45 1.21 4.51 1.15 3.37 1.07 1.09 0.99 3.42 3.74 3.28
10.0 9.5 9.0 8.5 8.0 7.5 7.0 6.5 6.0 5.5 5.0 4.5 4.0 3.5 3.0 2.5 2.0 1.5 1.0 0.5 0.0
f1 (ppm)

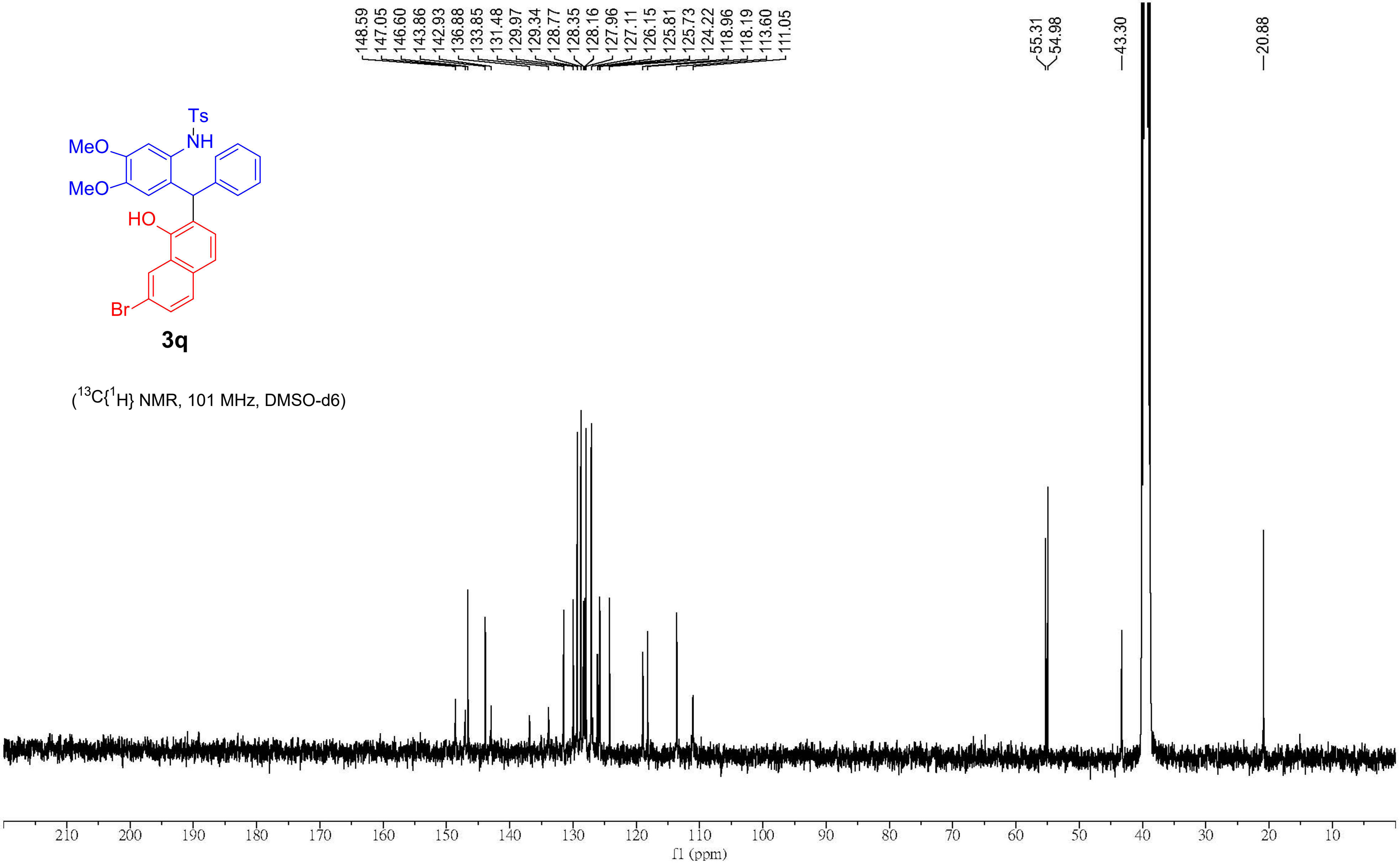

3q

($^{13}C\{^{1}H\}$ NMR, 101 MHz, DMSO-d6)

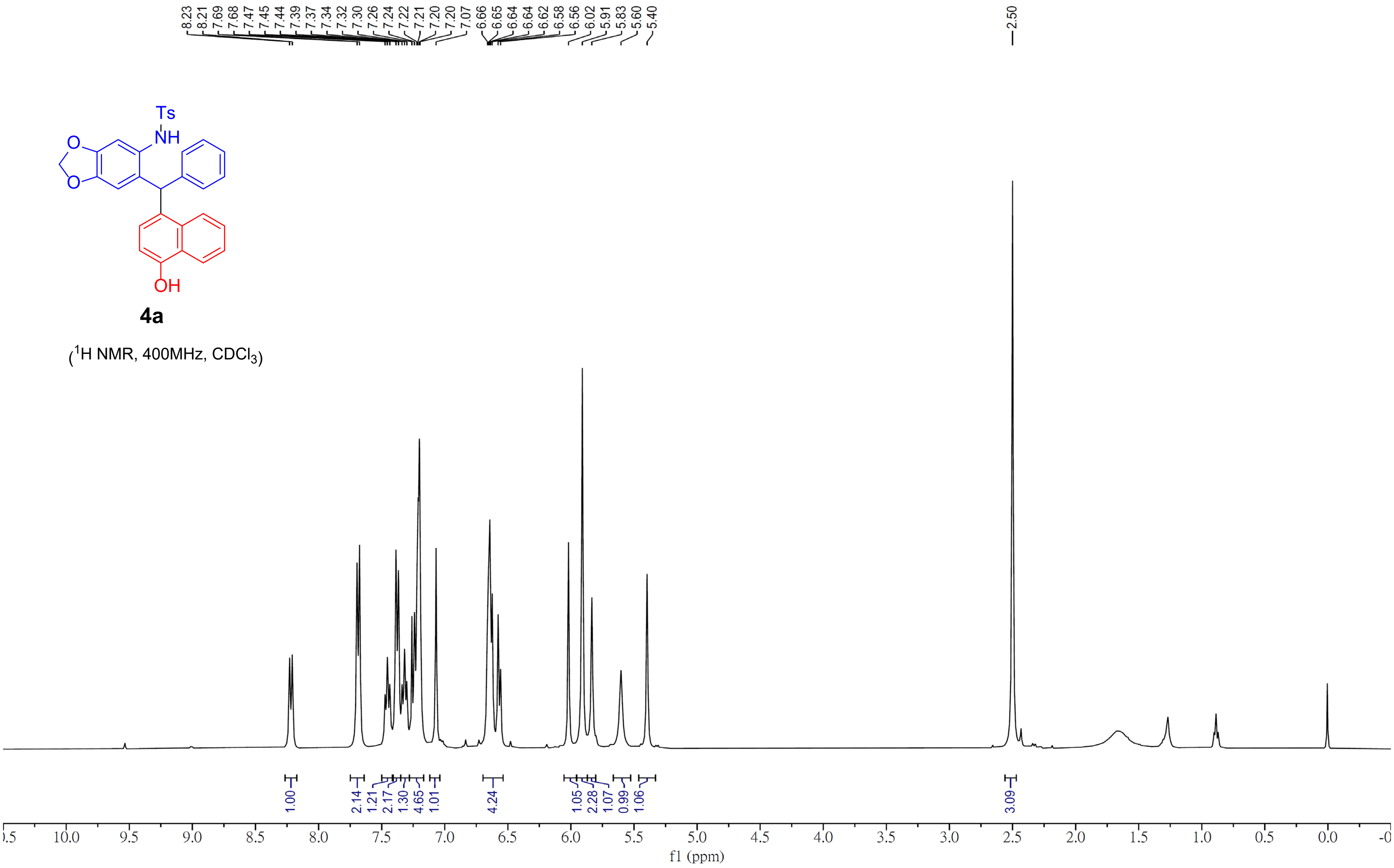

4a

(^{1}H NMR, 400MHz, CDCl$_3$)

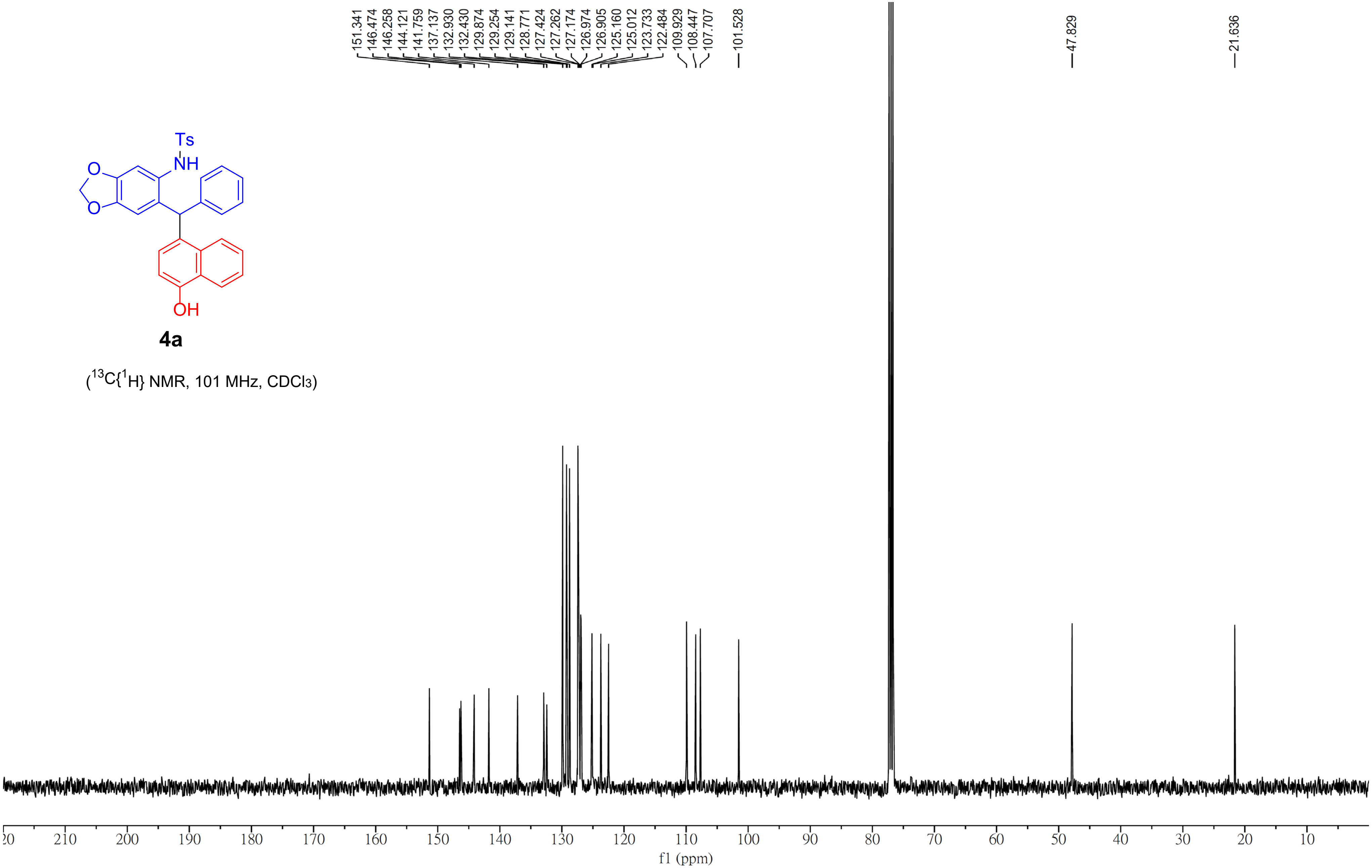

4a

($^{13}C\{^1H\}$ NMR, 101 MHz, $CDCl_3$)

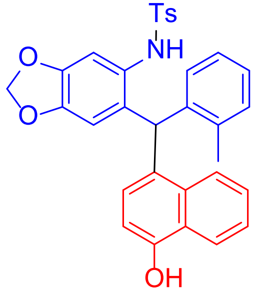

4b

(^{1}H NMR, 400MHz, $CDCl_3$)

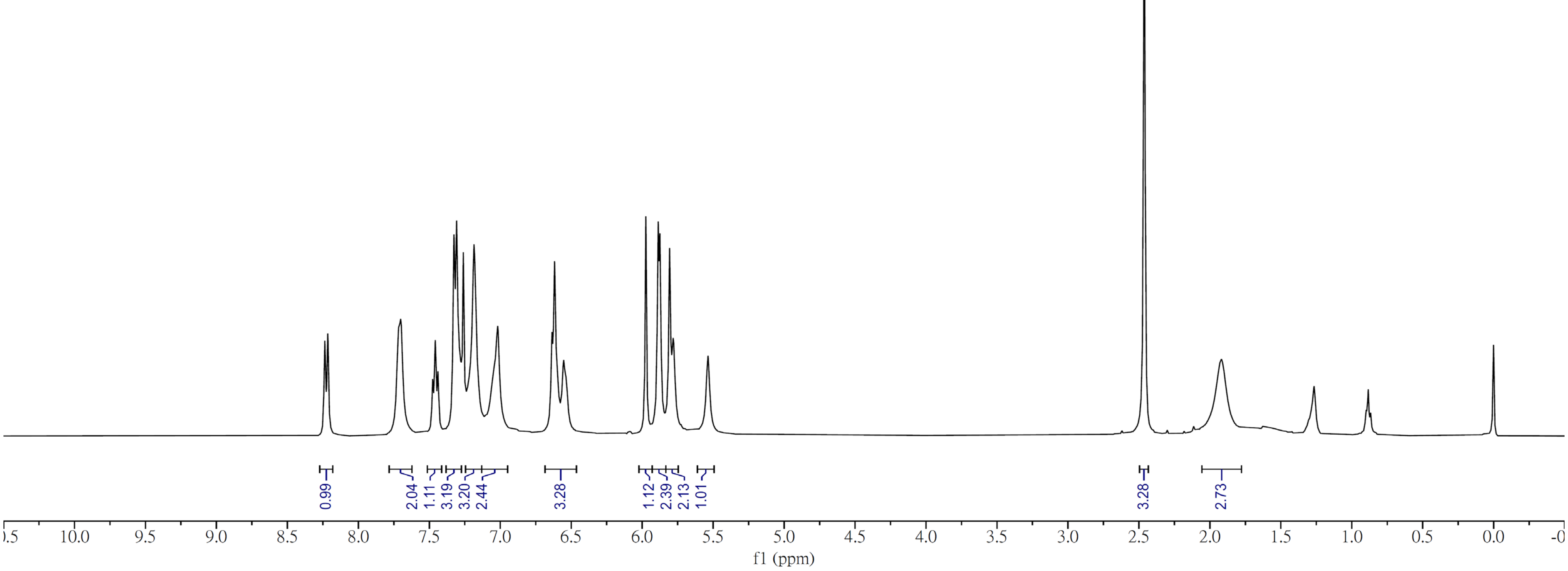

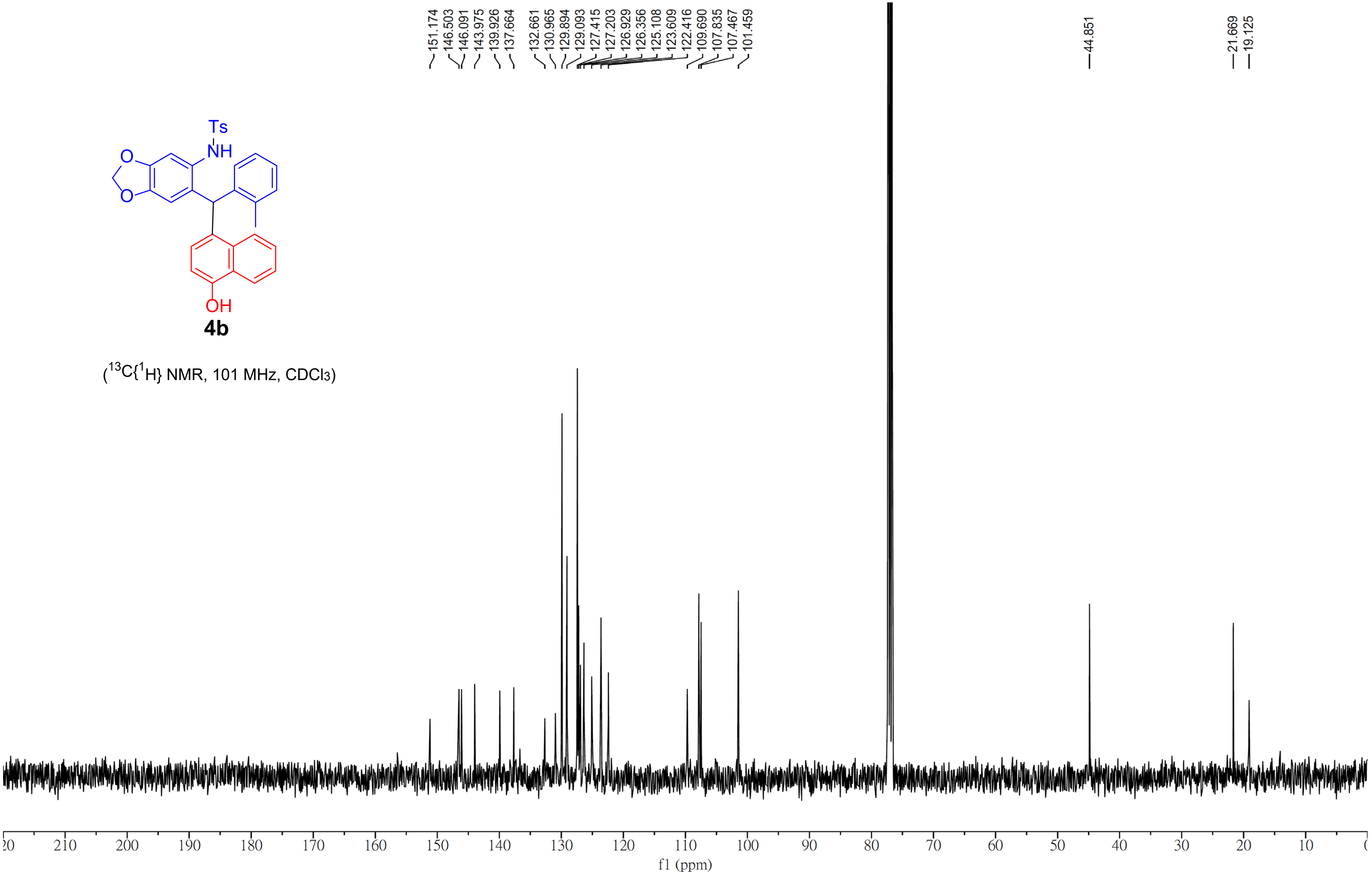
151.174
146.503
146.091
143.975
139.926
137.664
132.661
130.965
129.894
129.093
127.415
127.203
126.929
126.356
125.108
123.609
122.416
109.690
107.835
107.467
101.459
44.851
21.669
19.125
Ts
NH
O
O
OH
4b
($^{13}C\{^1H\}$ NMR, 101 MHz, $CDCl_3$)
f1 (ppm)

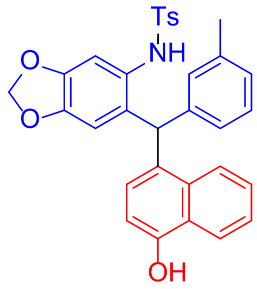

4c

(^{1}H NMR, 400MHz, $CDCl_3$)

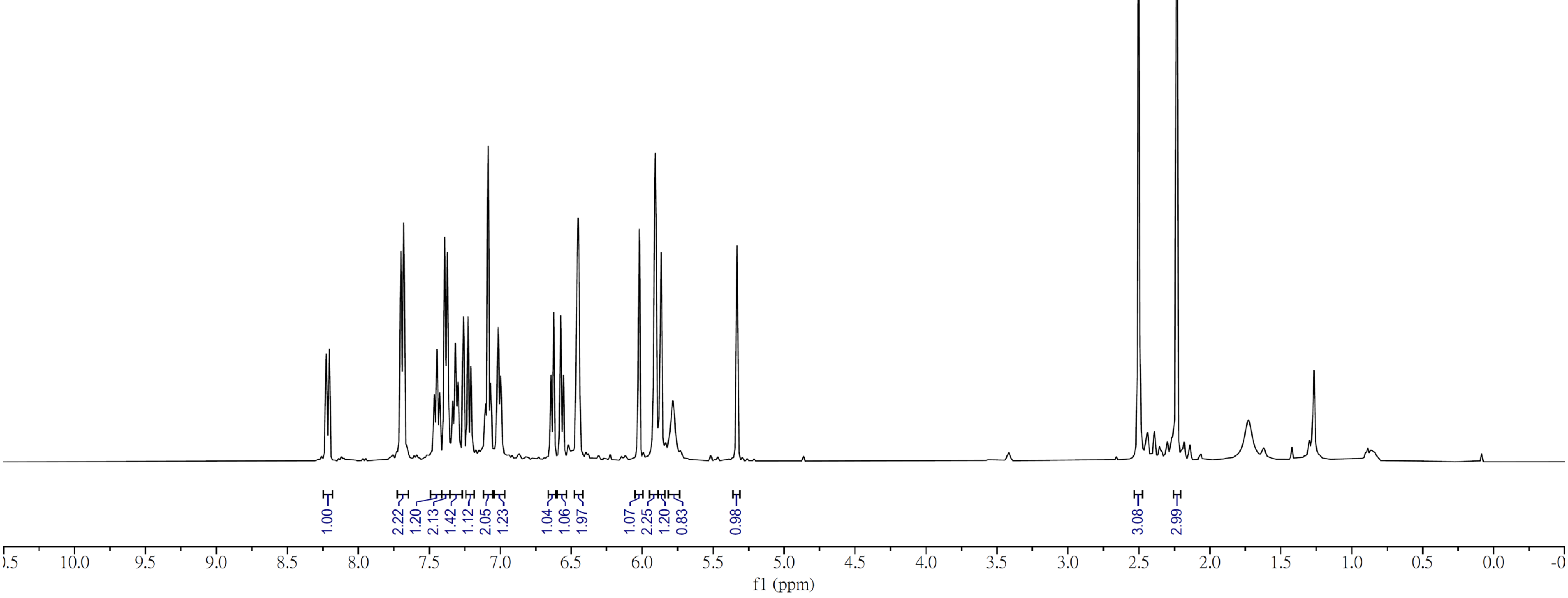

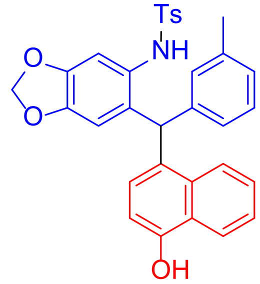

4c

($^{13}C\{^{1}H\}$ NMR, 101 MHz, $CDCl_3$)

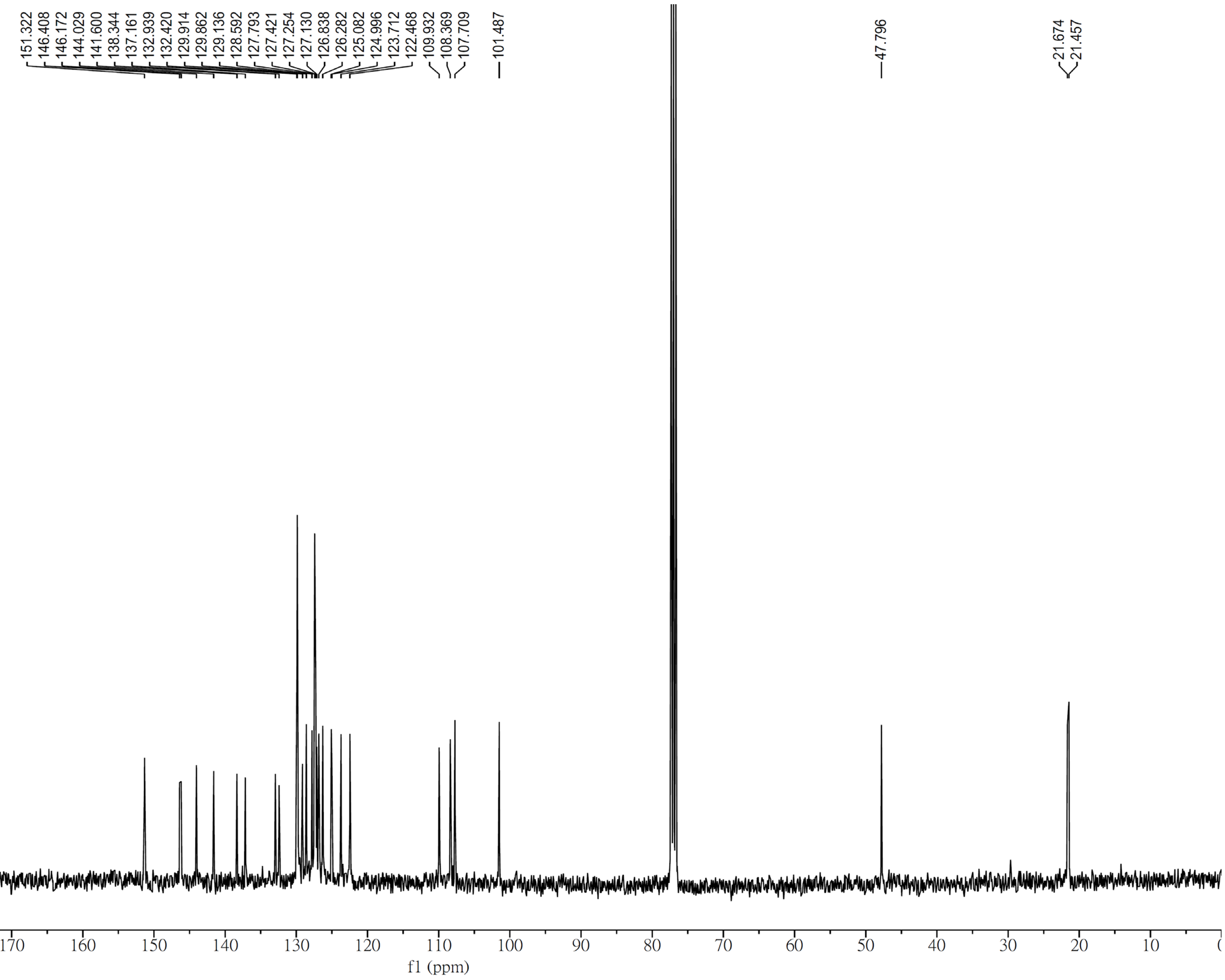

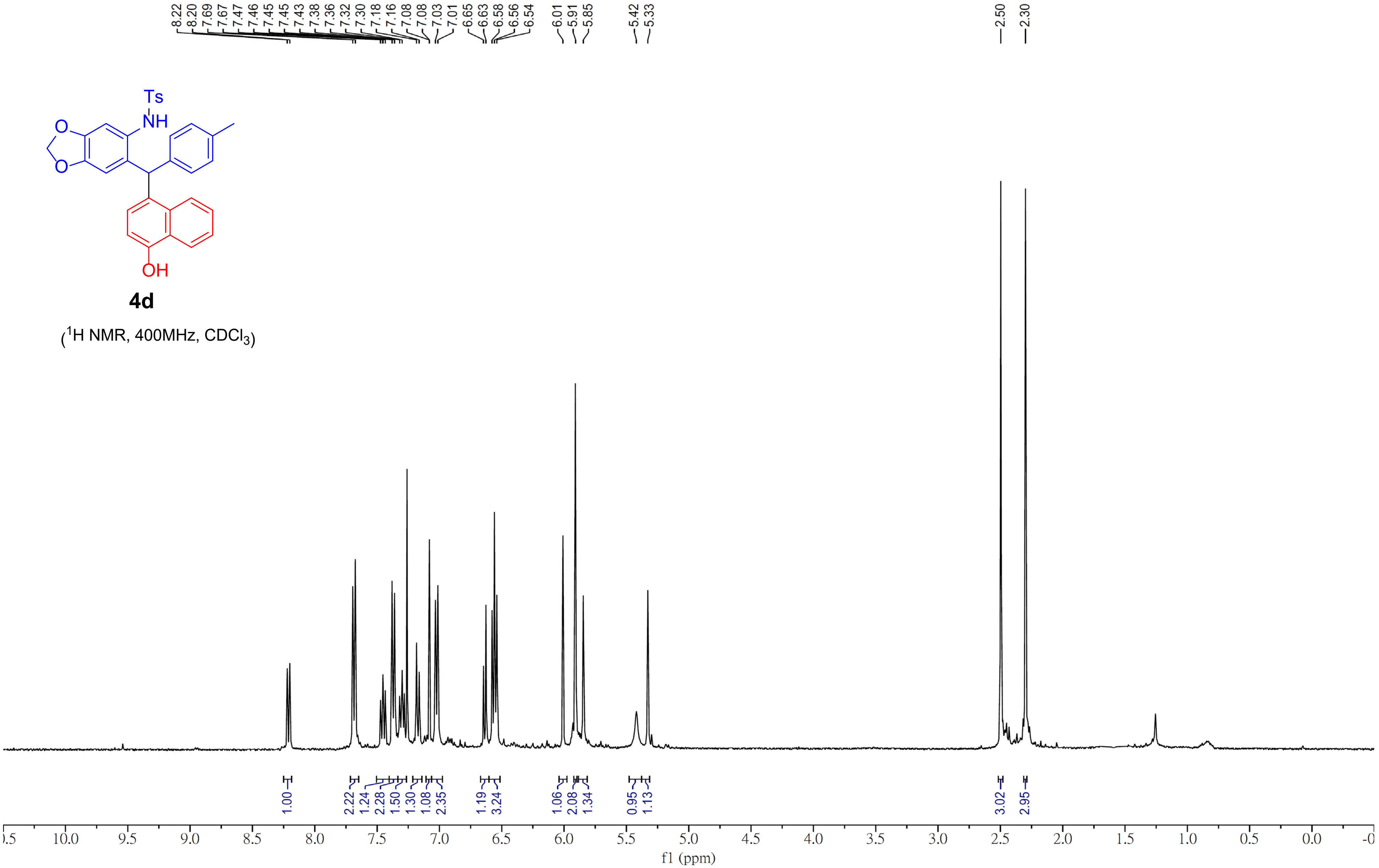

4d

(^{1}H NMR, 400MHz, $CDCl_3$)

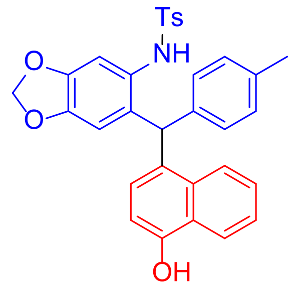

4d

($^{13}C\{^1H\}$ NMR, 101 MHz, $CDCl_3$)

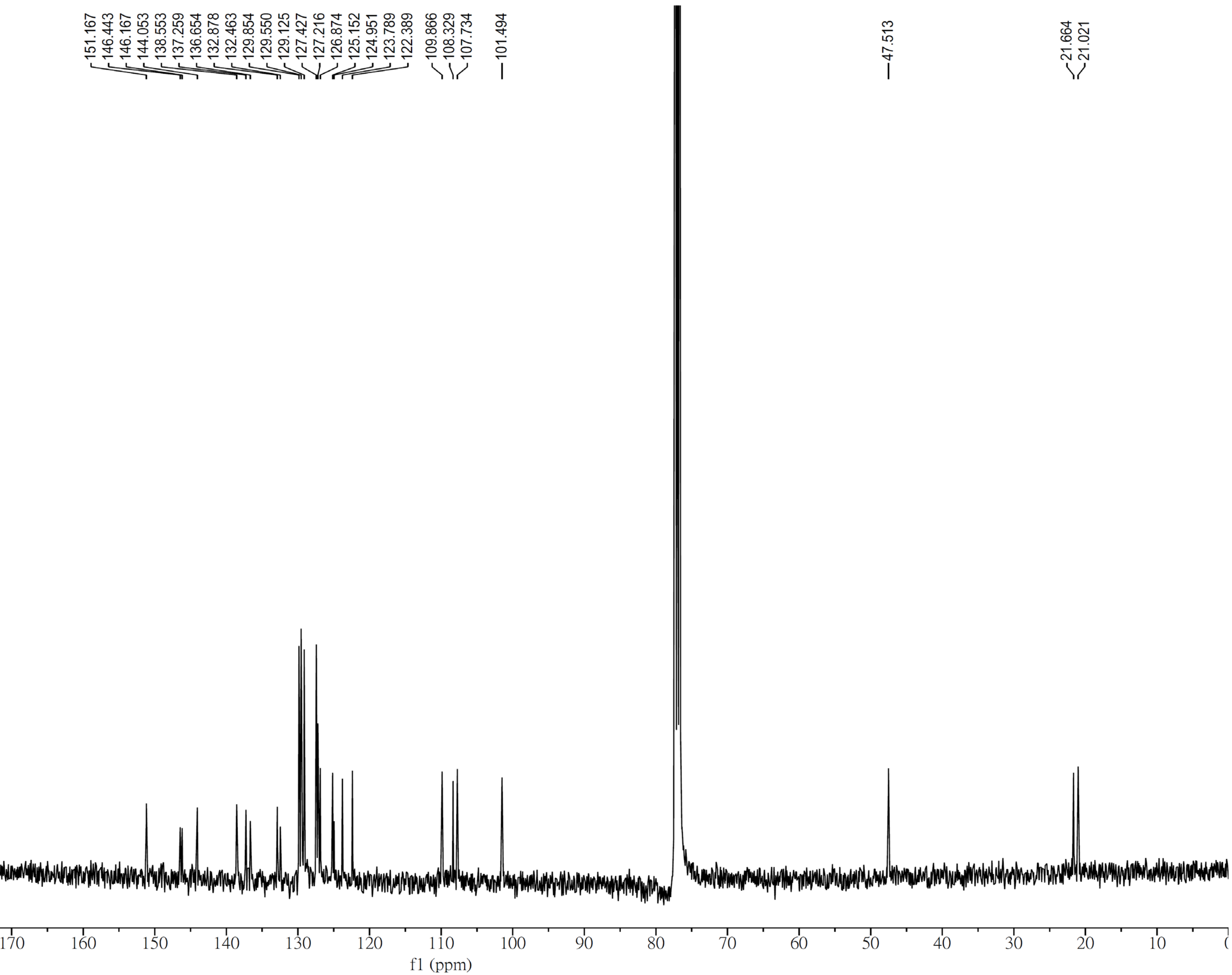

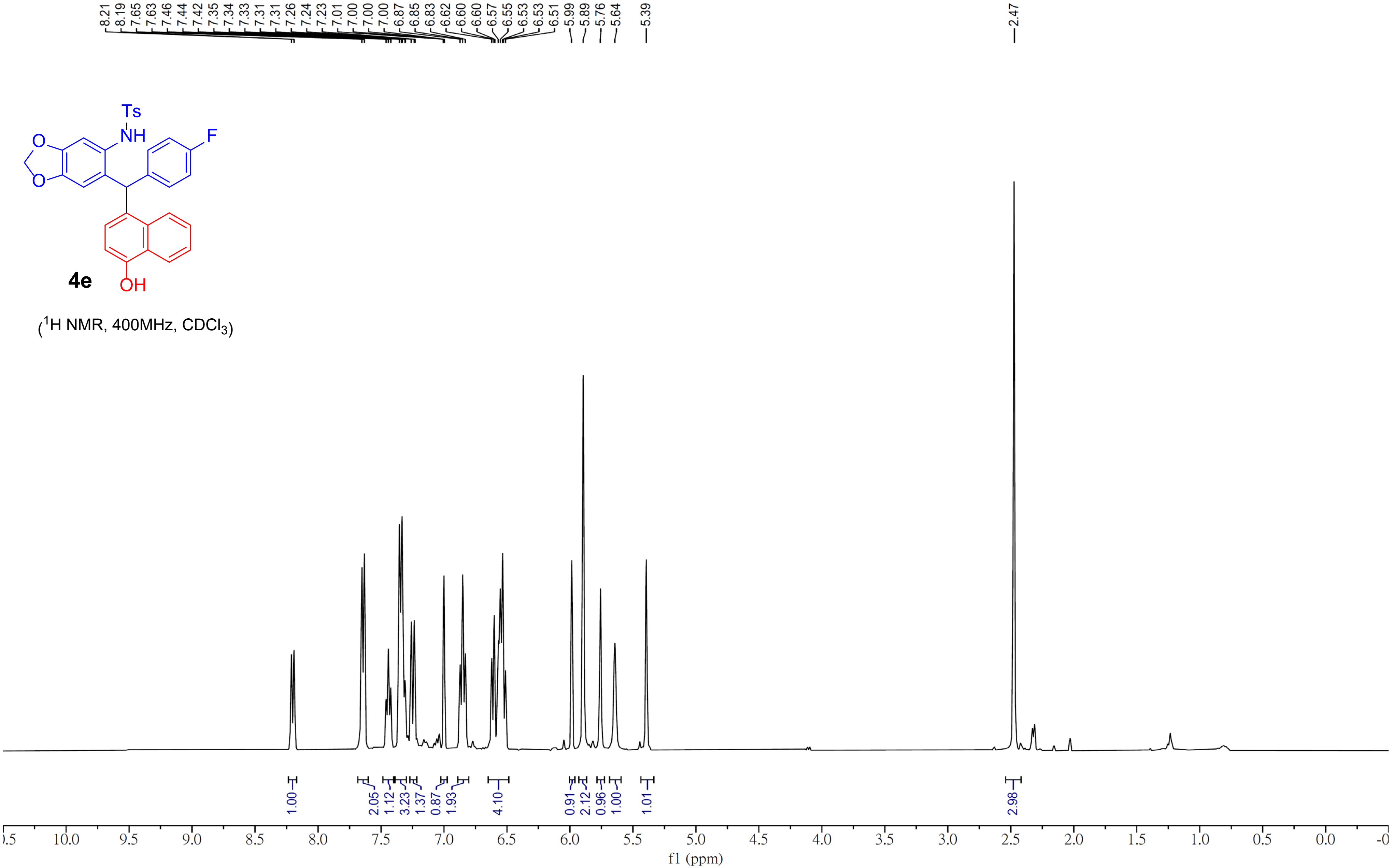
8.21
8.19
7.65
7.63
7.46
7.44
7.42
7.35
7.34
7.33
7.31
7.31
7.26
7.24
7.23
7.01
7.00
7.00
7.00
6.87
6.85
6.83
6.62
6.60
6.60
6.57
6.55
6.53
6.53
6.51
5.99
5.89
5.76
5.64
5.39
2.47
Ts
NH
F
O
O
4e
OH
(1H NMR, 400MHz, CDCl3)
1.00
2.05
1.12
3.23
1.37
0.87
1.93
4.10
0.91
2.12
0.96
1.00
1.01
2.98
f1 (ppm)

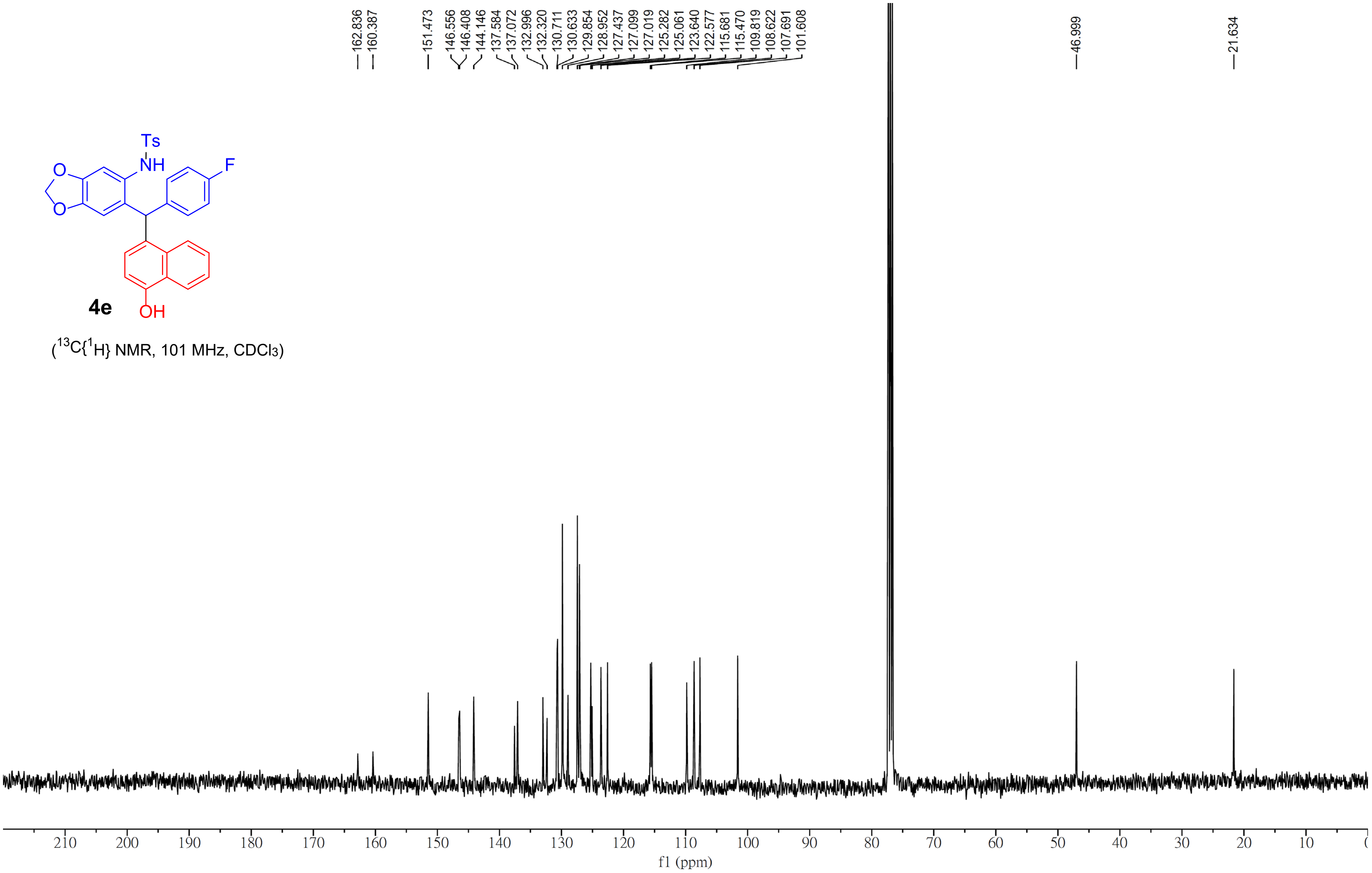

4e

($^{13}C\{^1H\}$ NMR, 101 MHz, CDCl$_3$)

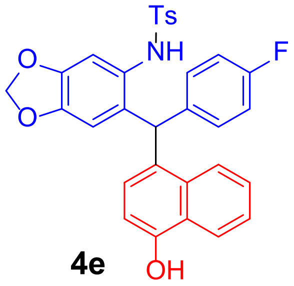

4e

(^{19}F NMR, 376MHz, CDCl$_3$)

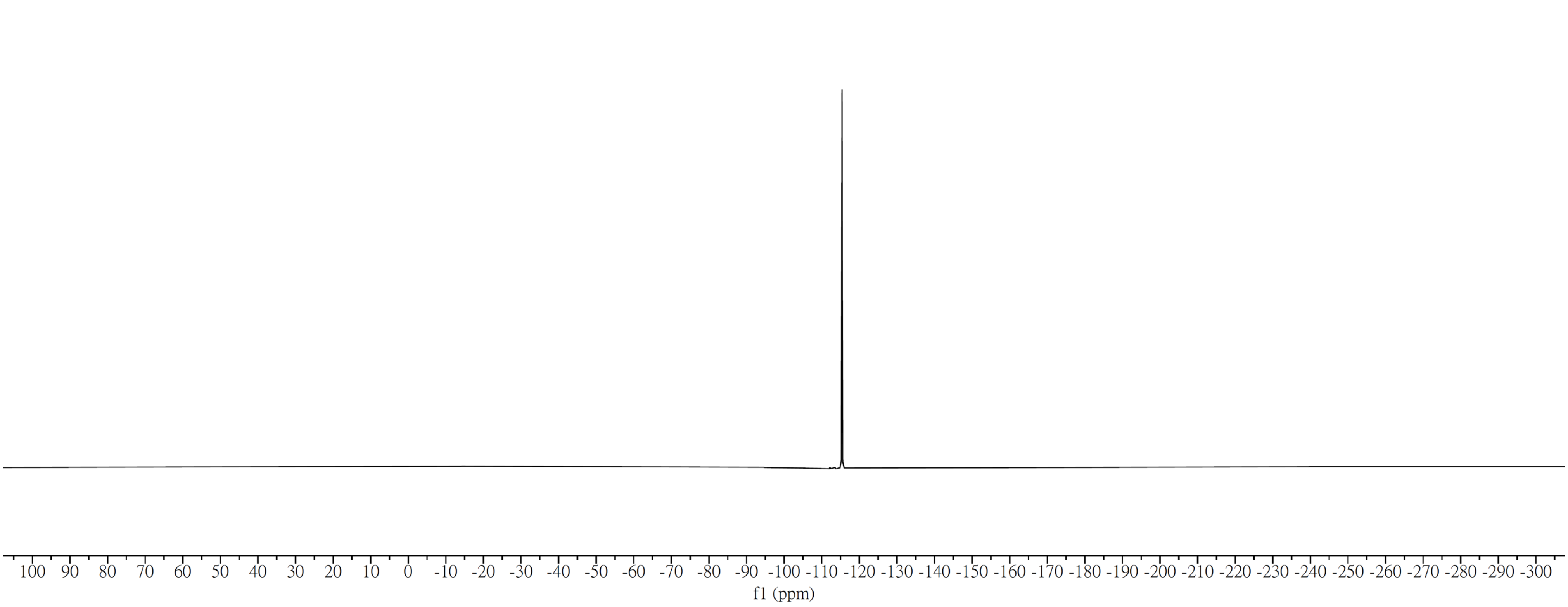

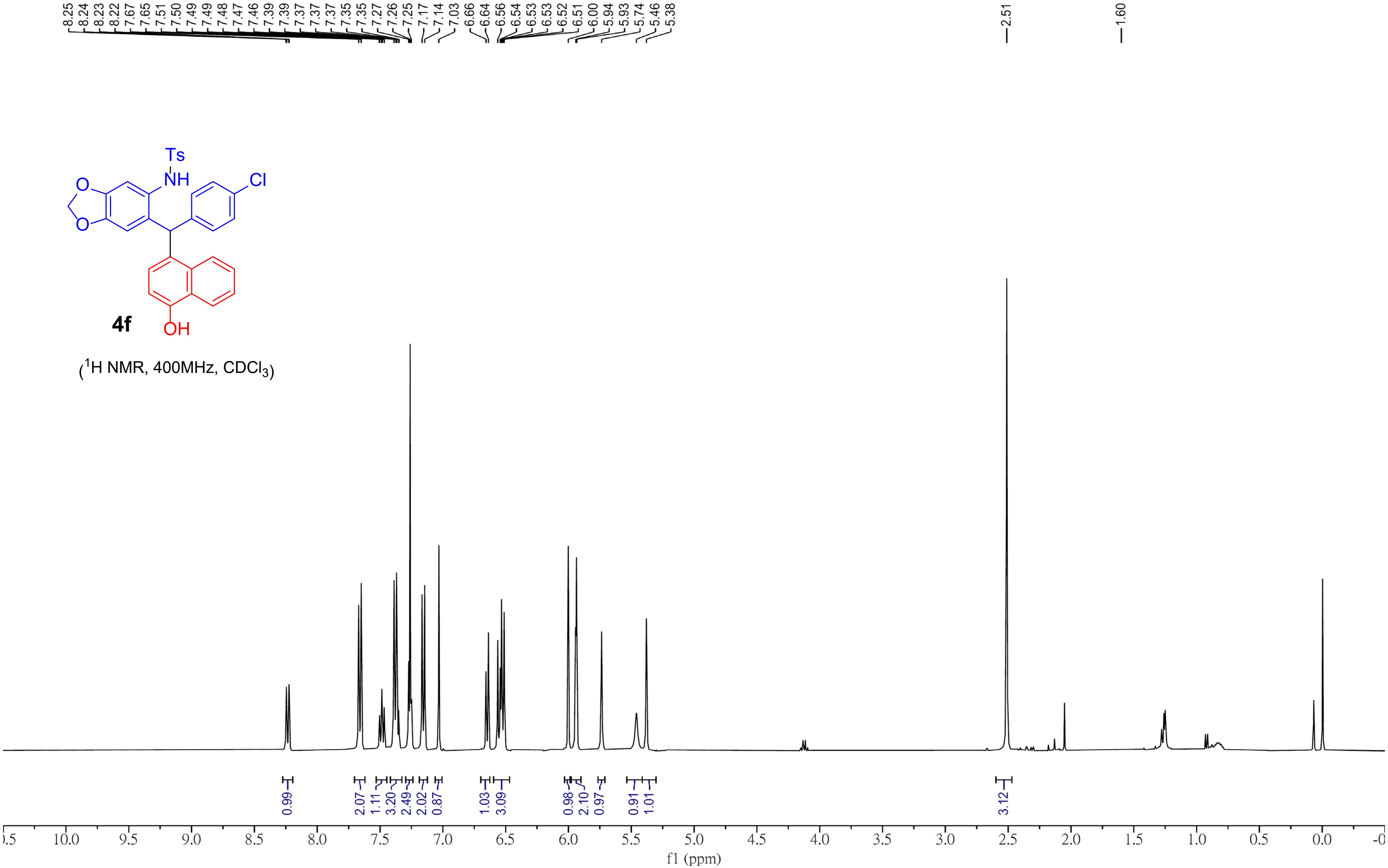

4f

(^{1}H NMR, 400MHz, $CDCl_3$)

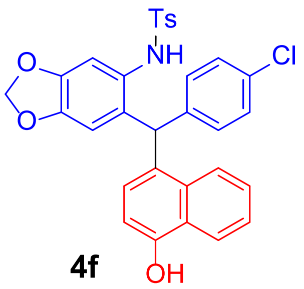

4f

($^{13}C\{^{1}H\}$ NMR, 101 MHz, $CDCl_3$)

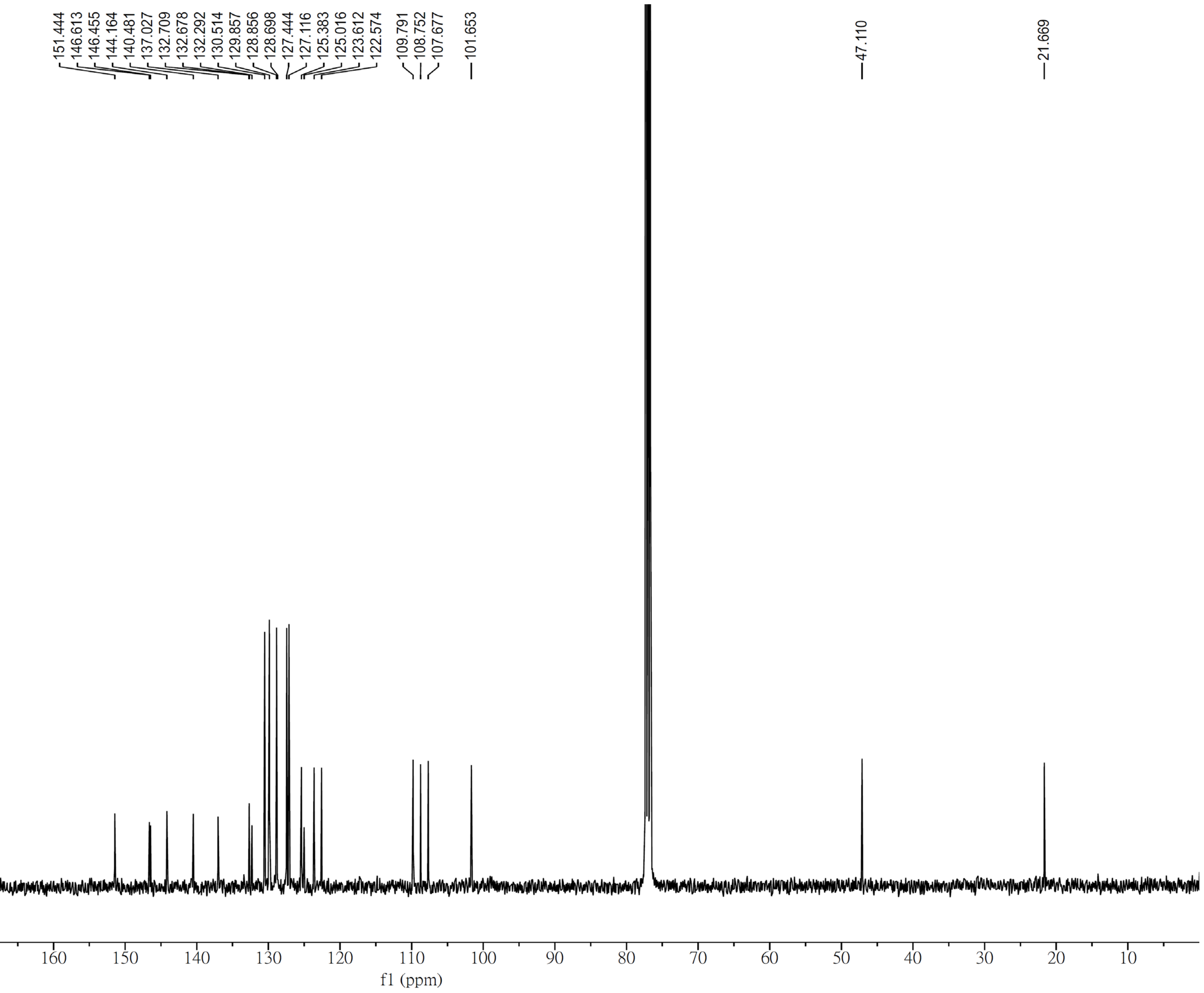

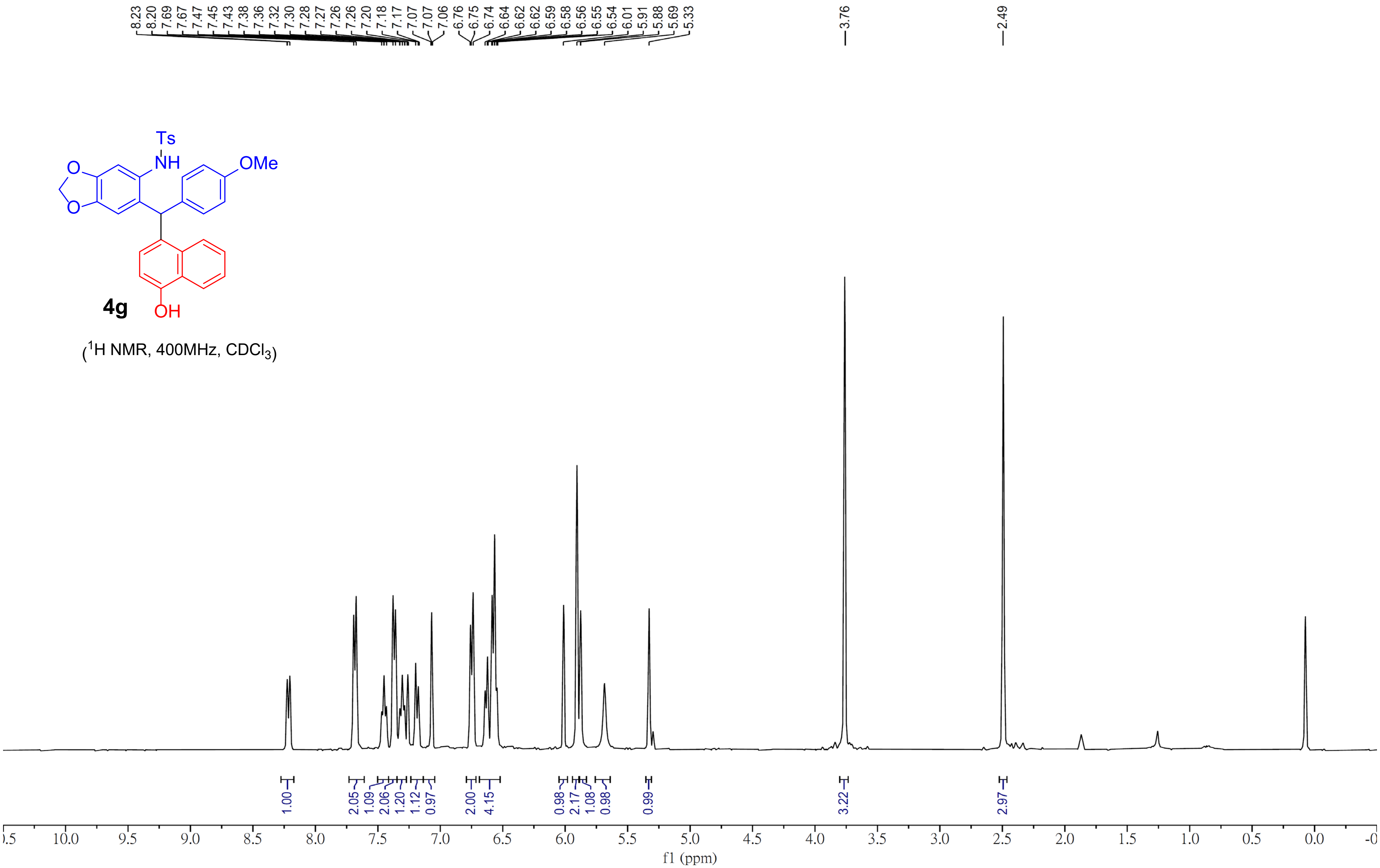

Ts
NH
OMe
O
O
4g
OH
(1H NMR, 400MHz, CDCl3)
8.23
8.20
7.69
7.67
7.47
7.45
7.43
7.38
7.36
7.32
7.30
7.28
7.27
7.26
7.26
7.20
7.18
7.17
7.07
7.07
7.06
6.76
6.75
6.74
6.64
6.62
6.62
6.59
6.58
6.56
6.55
6.54
6.01
5.91
5.88
5.69
5.33
3.76
2.49
1.00
2.05
1.09
2.06
1.20
1.12
0.97
2.00
4.15
0.98
2.17
1.08
0.98
0.99
3.22
2.97
f1 (ppm)

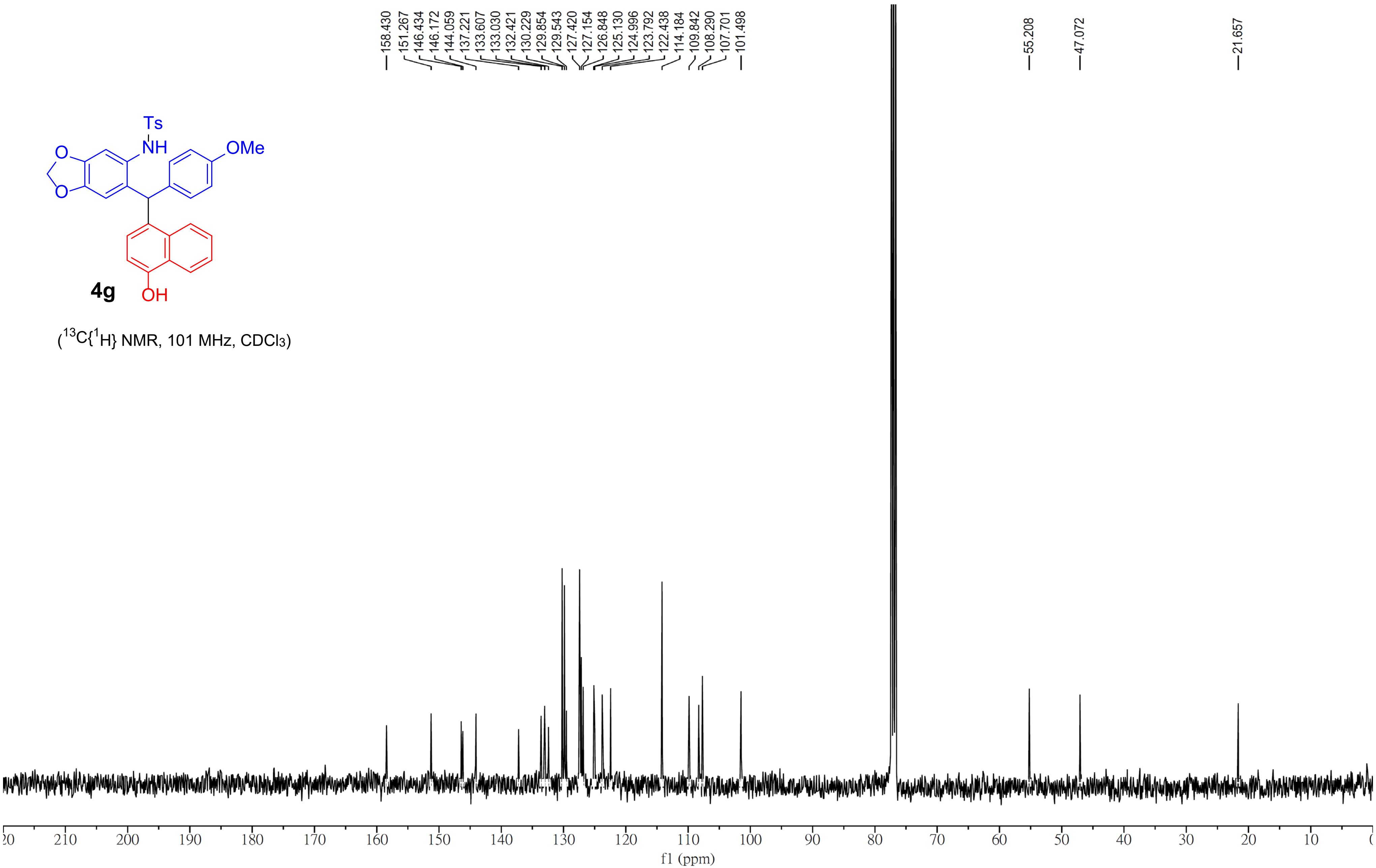
158.430
151.267
146.434
146.172
144.059
137.221
133.607
133.030
132.421
130.229
129.854
129.543
127.420
127.154
126.848
125.130
124.996
123.792
122.438
114.184
109.842
108.290
107.701
101.498
55.208
47.072
21.657
Ts
NH
OMe
O
O
OH
4g
($^{13}C\{^{1}H\}$ NMR, 101 MHz, CDCl3)
f1 (ppm)

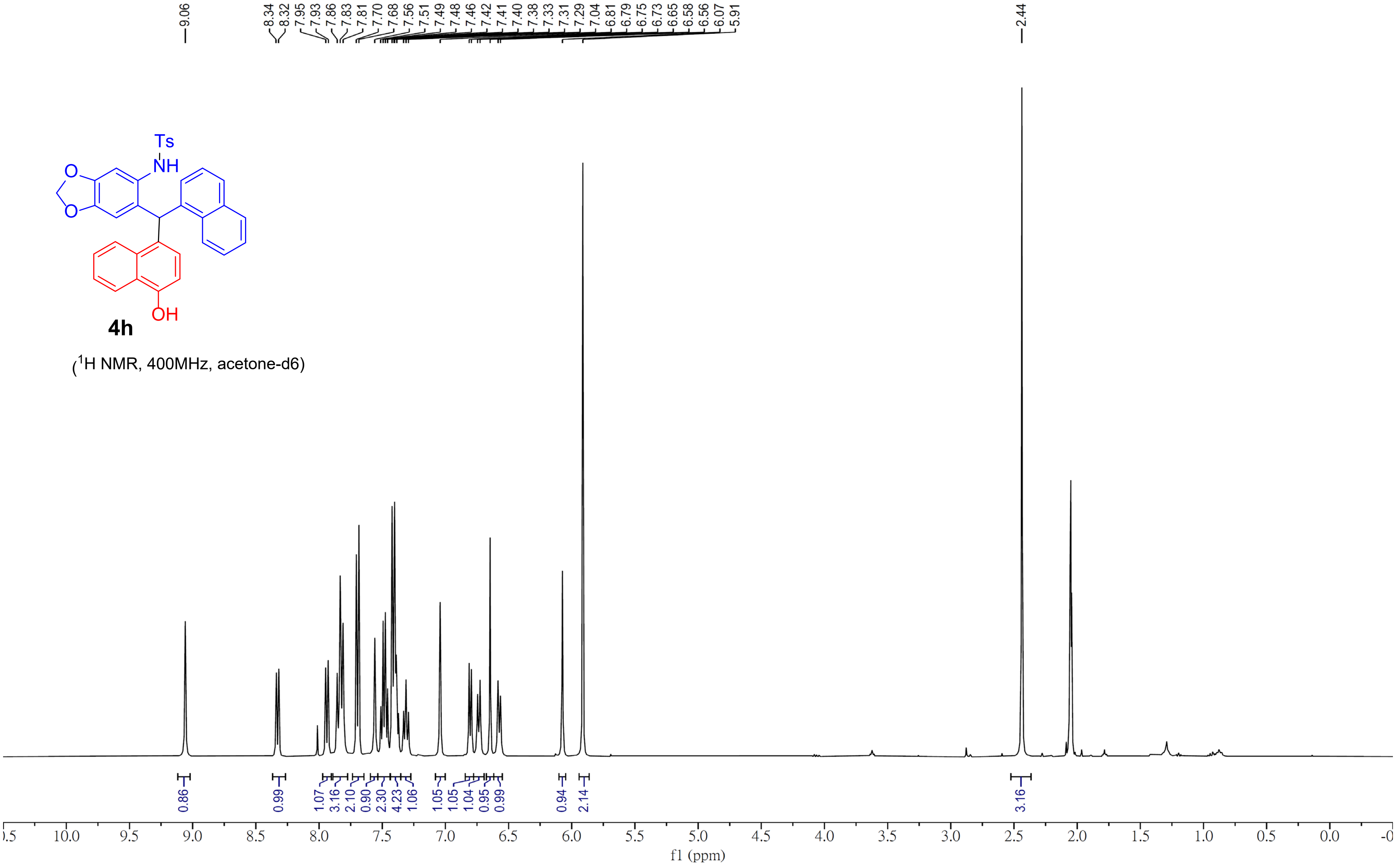
Ts
NH
O
O
OH
4h
(1H NMR, 400MHz, acetone-d6)
9.06
8.34
8.32
7.95
7.93
7.86
7.83
7.81
7.70
7.68
7.56
7.51
7.49
7.48
7.46
7.42
7.41
7.40
7.38
7.33
7.31
7.29
7.04
6.81
6.79
6.75
6.73
6.65
6.58
6.56
6.07
5.91
2.44
0.86
0.99
1.07
3.16
2.10
0.90
2.30
4.23
1.06
1.05
1.05
1.04
0.95
0.99
0.94
2.14
3.16
f1 (ppm)

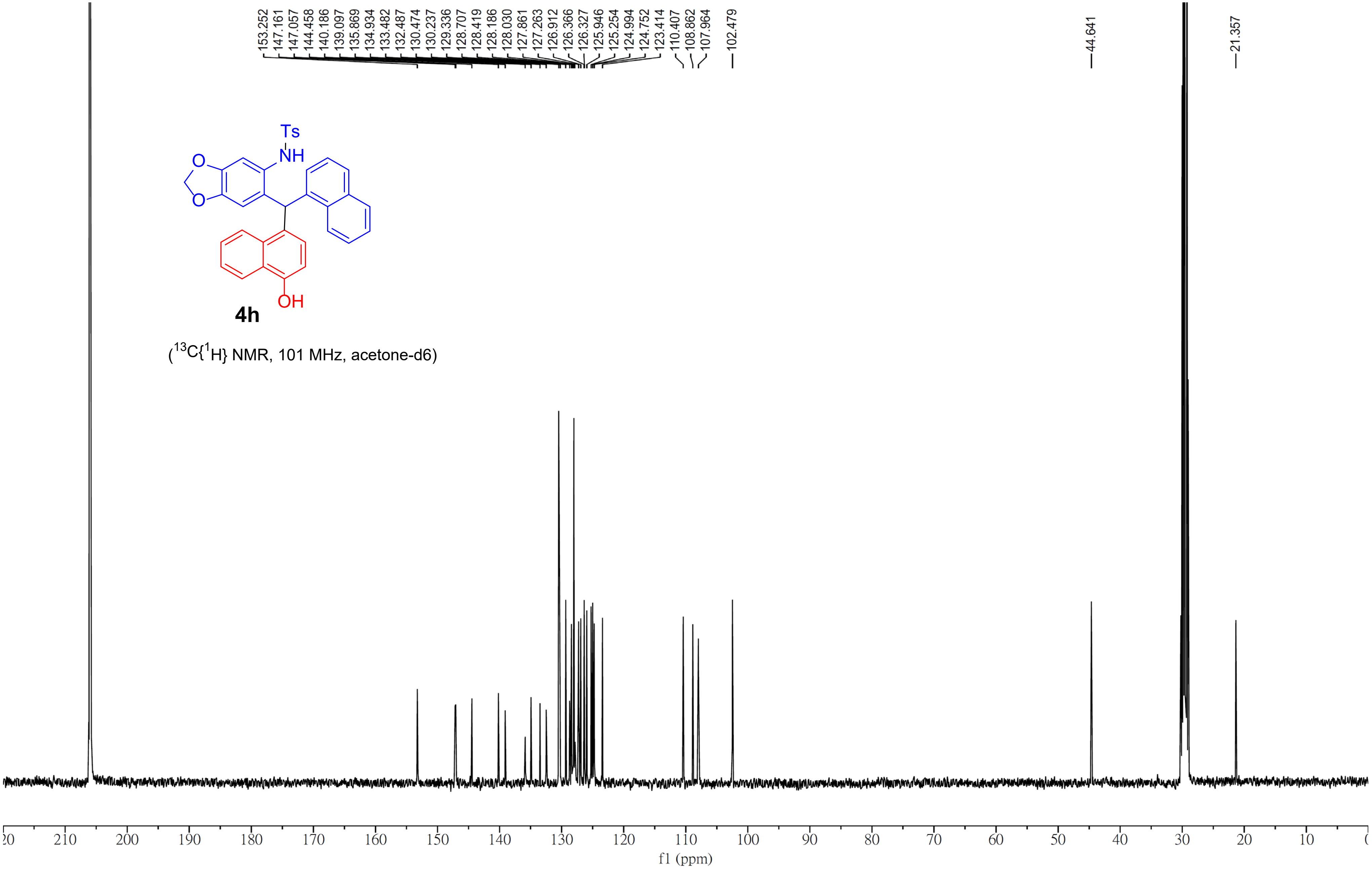
153.252
147.161
147.057
144.458
140.186
139.097
135.869
134.934
133.482
132.487
130.474
130.237
129.336
128.707
128.419
128.186
128.030
127.861
127.263
126.912
126.366
126.327
125.946
125.254
124.994
124.752
123.414
110.407
108.862
107.964
102.479
44.641
21.357
Ts
NH
O
O
OH
4h
(13C{1H} NMR, 101 MHz, acetone-d6)
f1 (ppm)

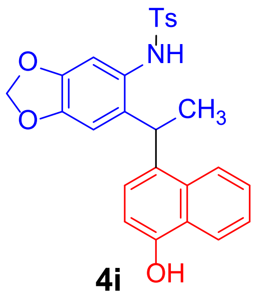

4i

(^{1}H NMR, 400MHz, $CDCl_3$)

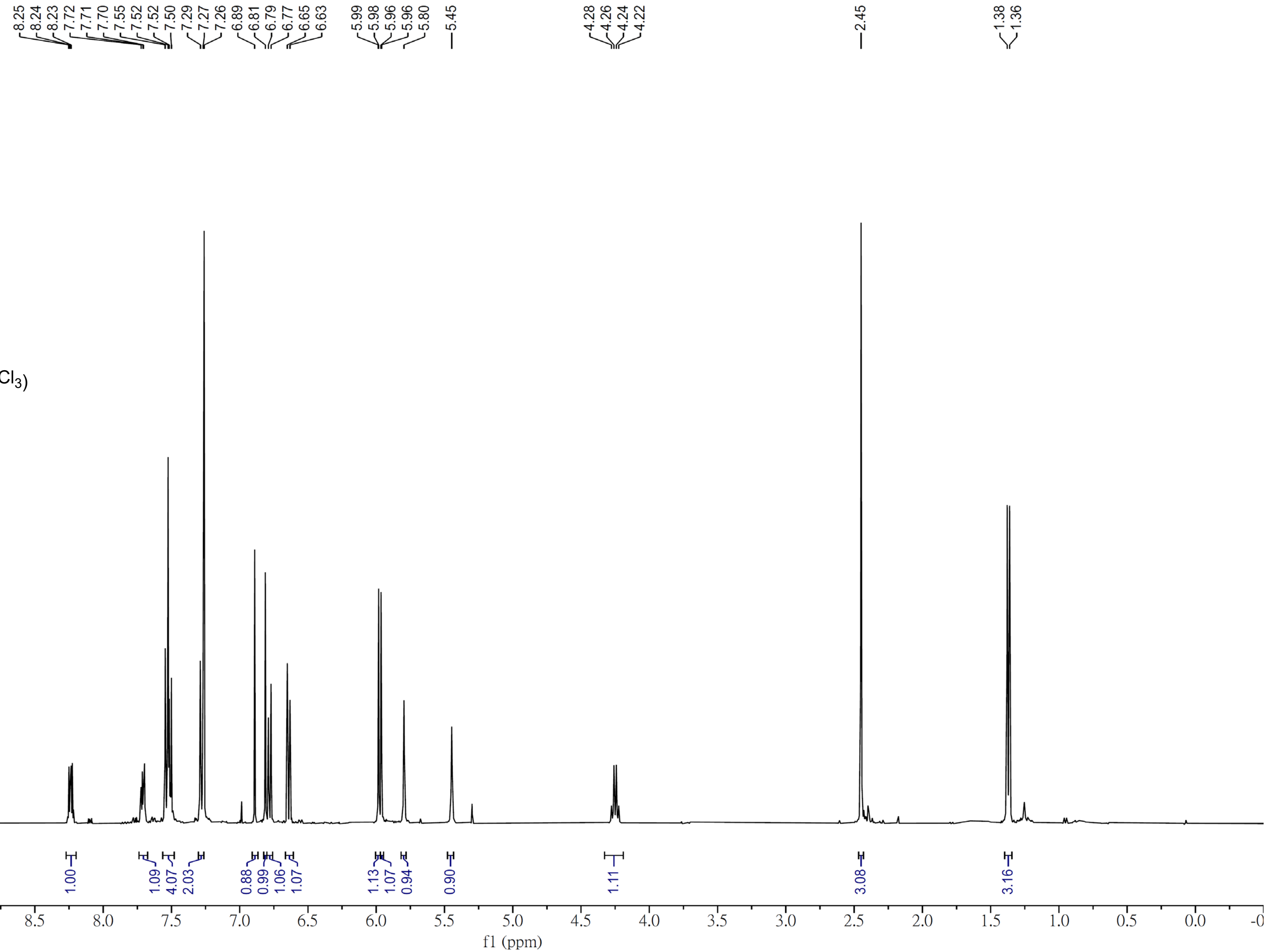

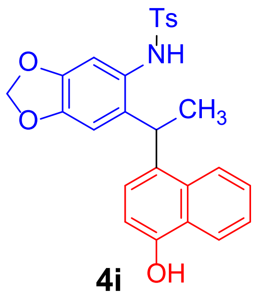

4i

(^{13}C{1H} NMR, 101 MHz, CDCl3)

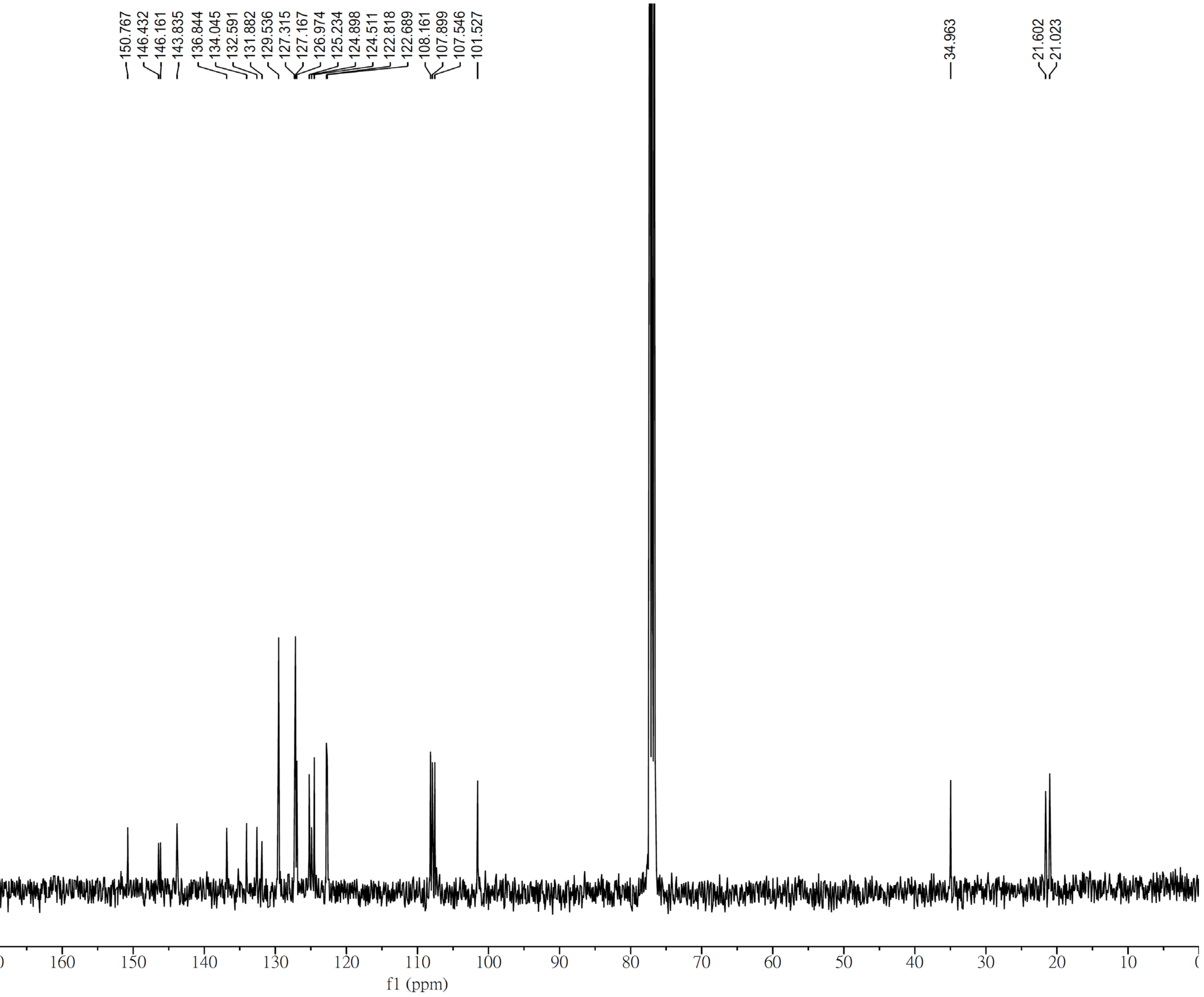

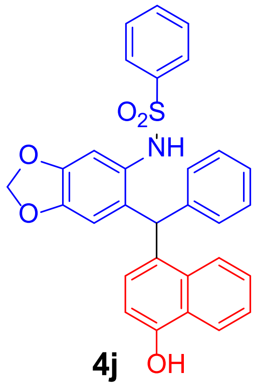

4j

(^{1}H NMR, 400MHz, acetone-d6)

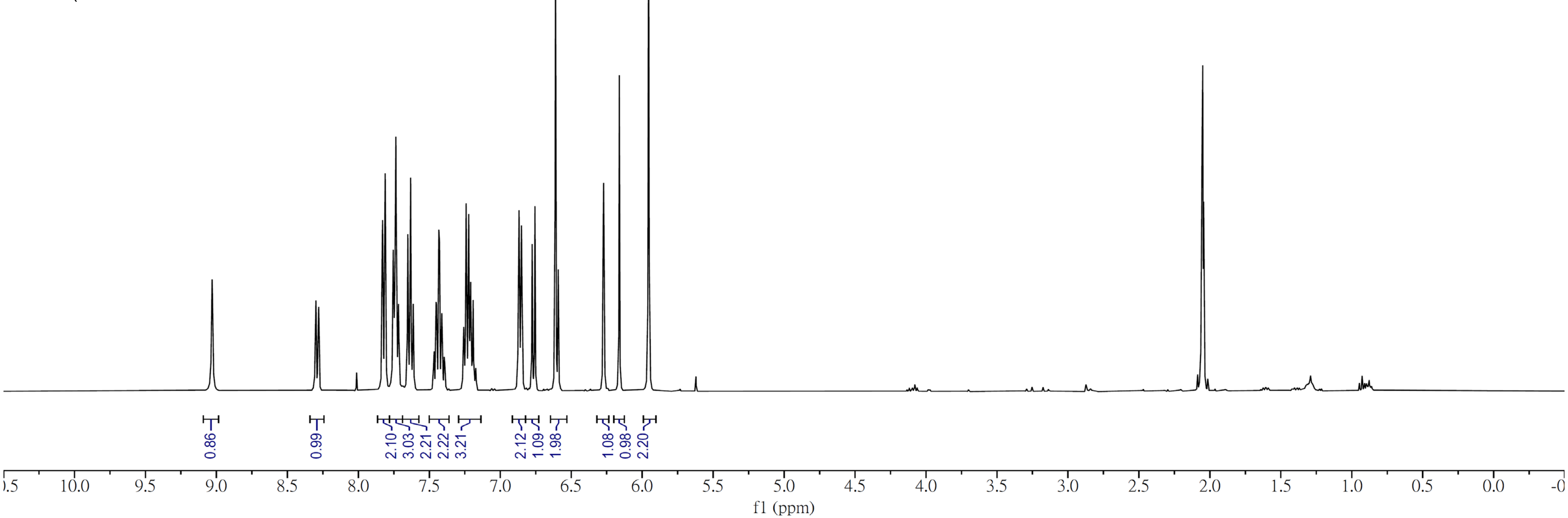

4j

(^{13}C{1H} NMR, 101 MHz,acetone-d6)

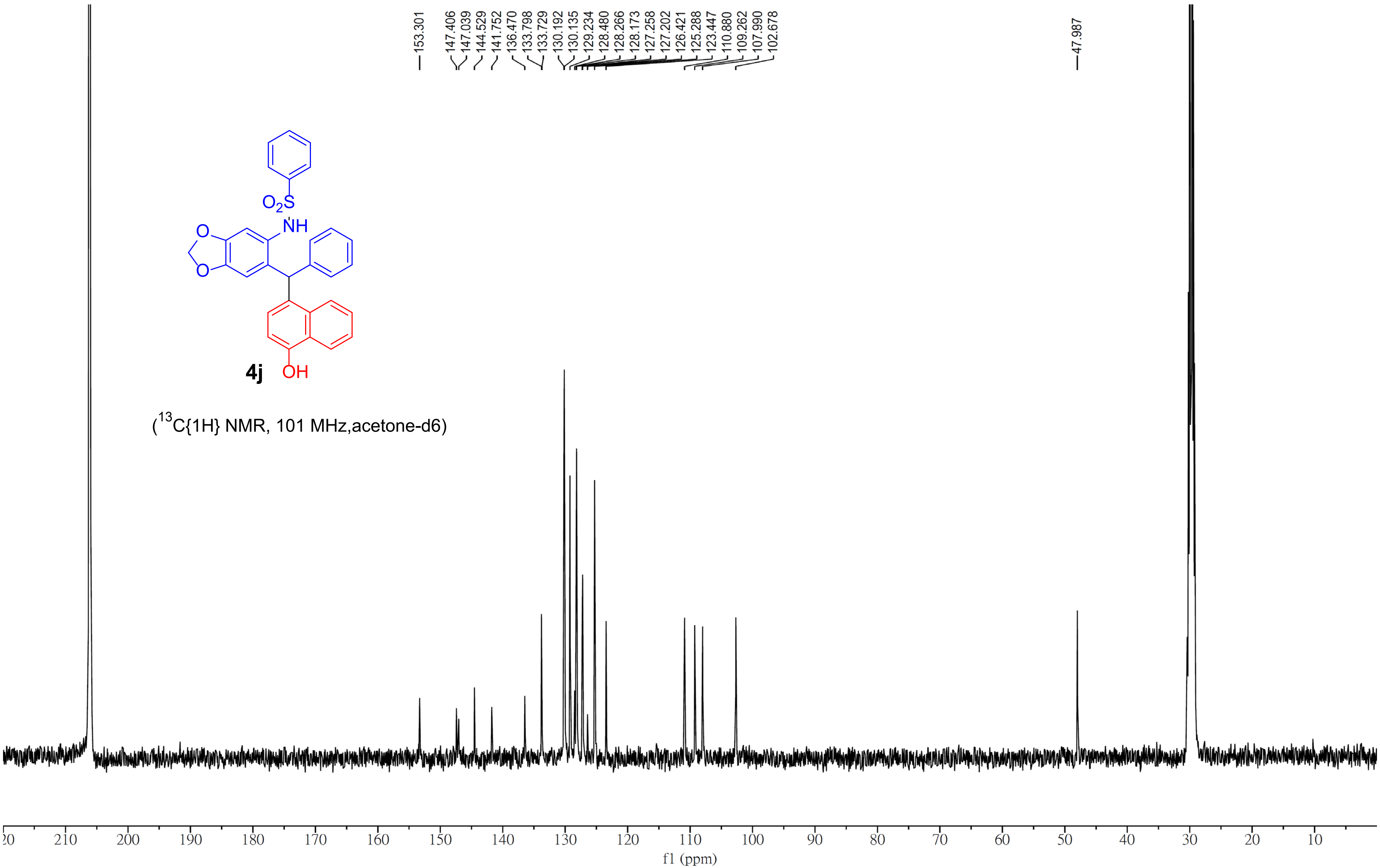

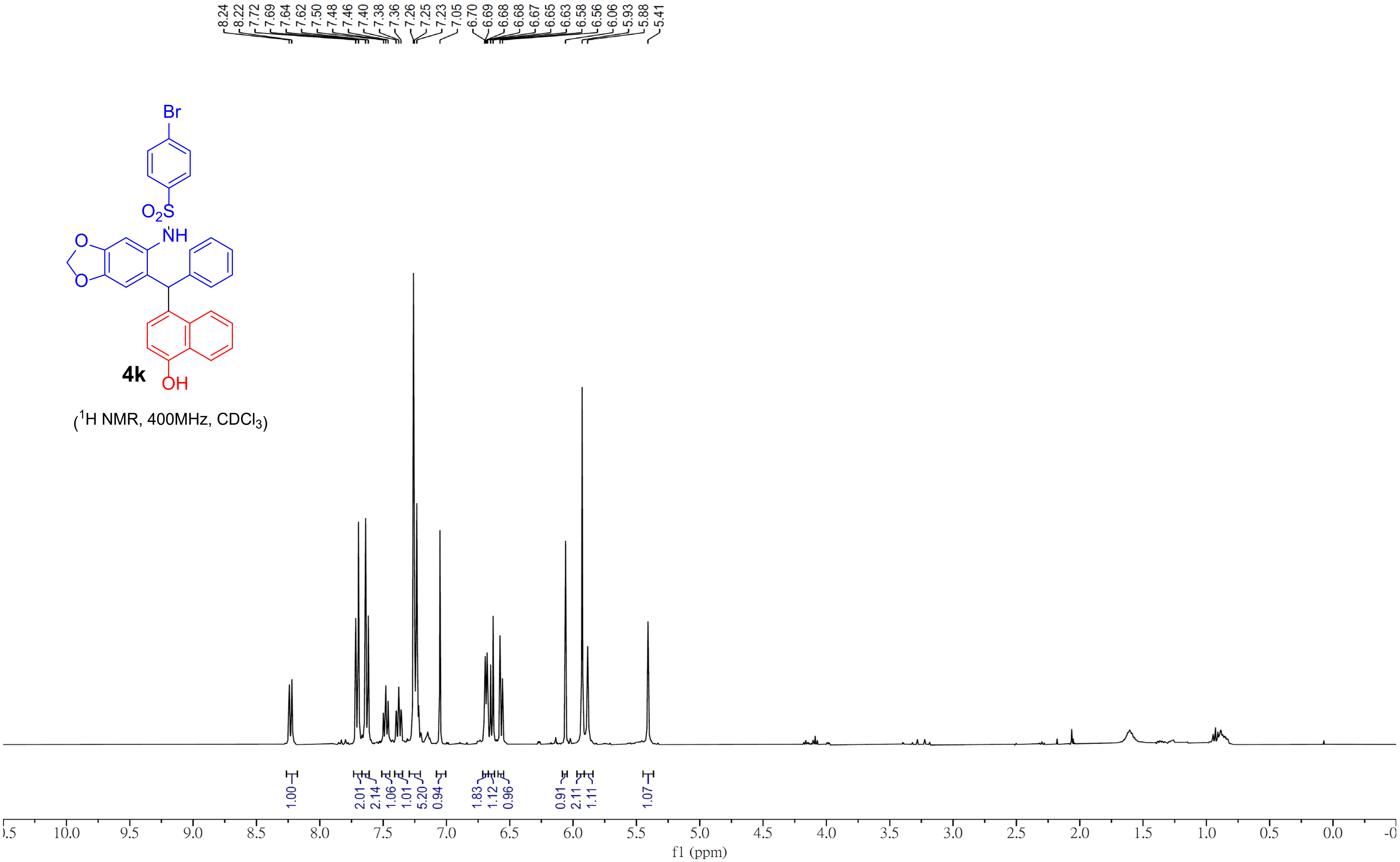

4k

(^{1}H NMR, 400MHz, $CDCl_3$)

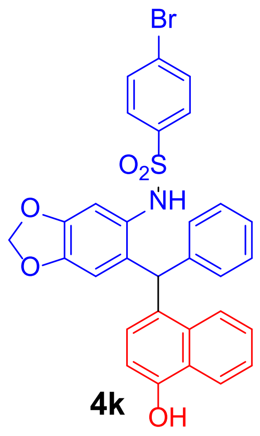

4k

($^{13}C\{^1H\}$ NMR, 101 MHz, $CDCl_3$)

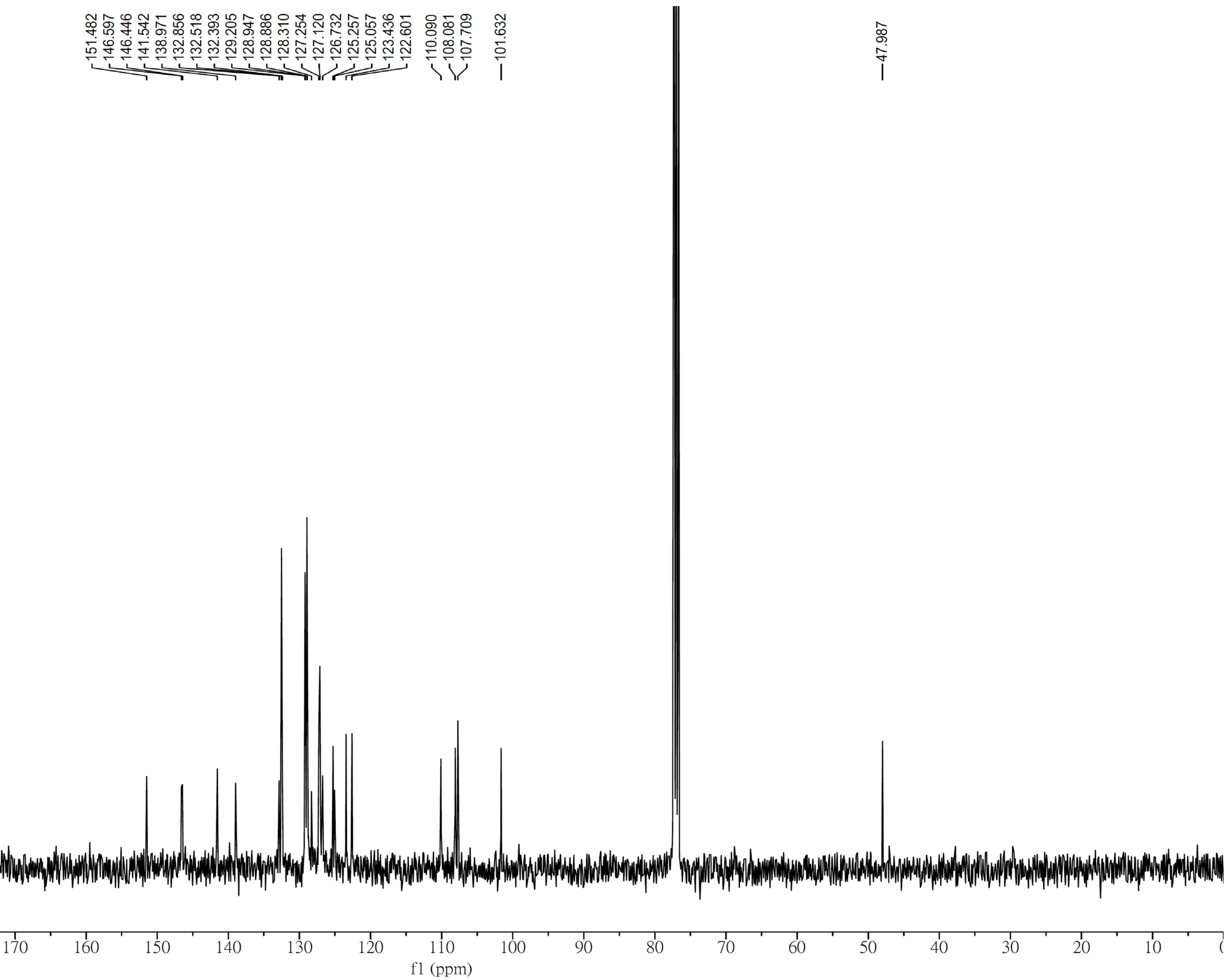

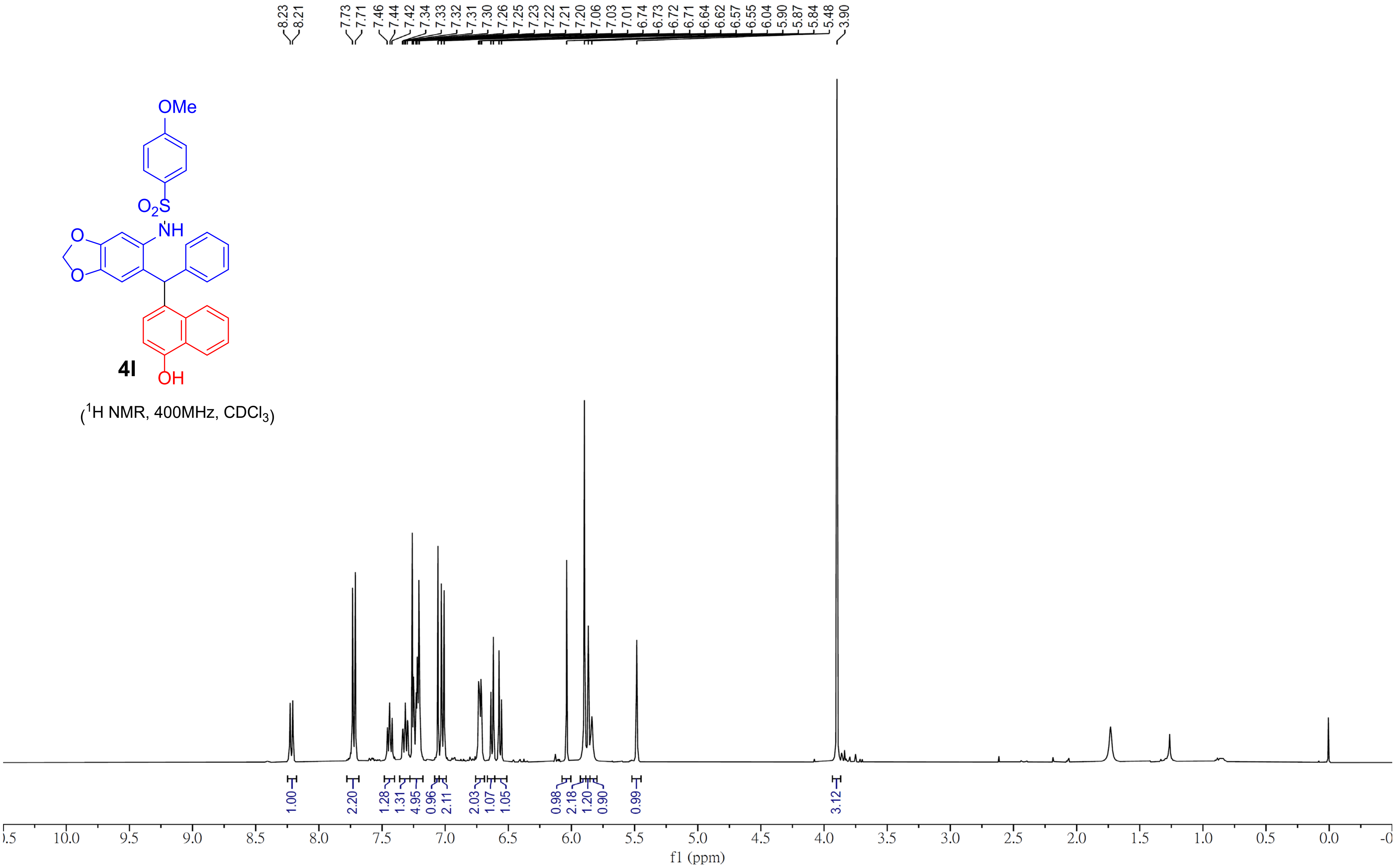

OMe
O_2S
NH
O
O
OH
4l
(¹H NMR, 400MHz, CDCl₃)
8.23
8.21
7.73
7.71
7.46
7.44
7.42
7.34
7.33
7.32
7.31
7.30
7.26
7.25
7.23
7.22
7.21
7.20
7.06
7.03
7.01
6.74
6.73
6.72
6.71
6.64
6.62
6.57
6.55
6.04
5.90
5.87
5.84
5.48
3.90
1.00
2.20
1.28
1.31
4.95
0.96
2.11
2.03
1.07
1.05
0.98
2.18
1.20
0.90
0.99
3.12
f1 (ppm)

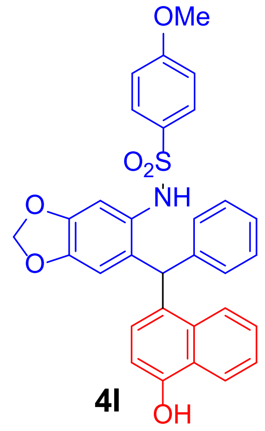

4l

($^{13}C\{^1H\}$ NMR, 101 MHz, $CDCl_3$)

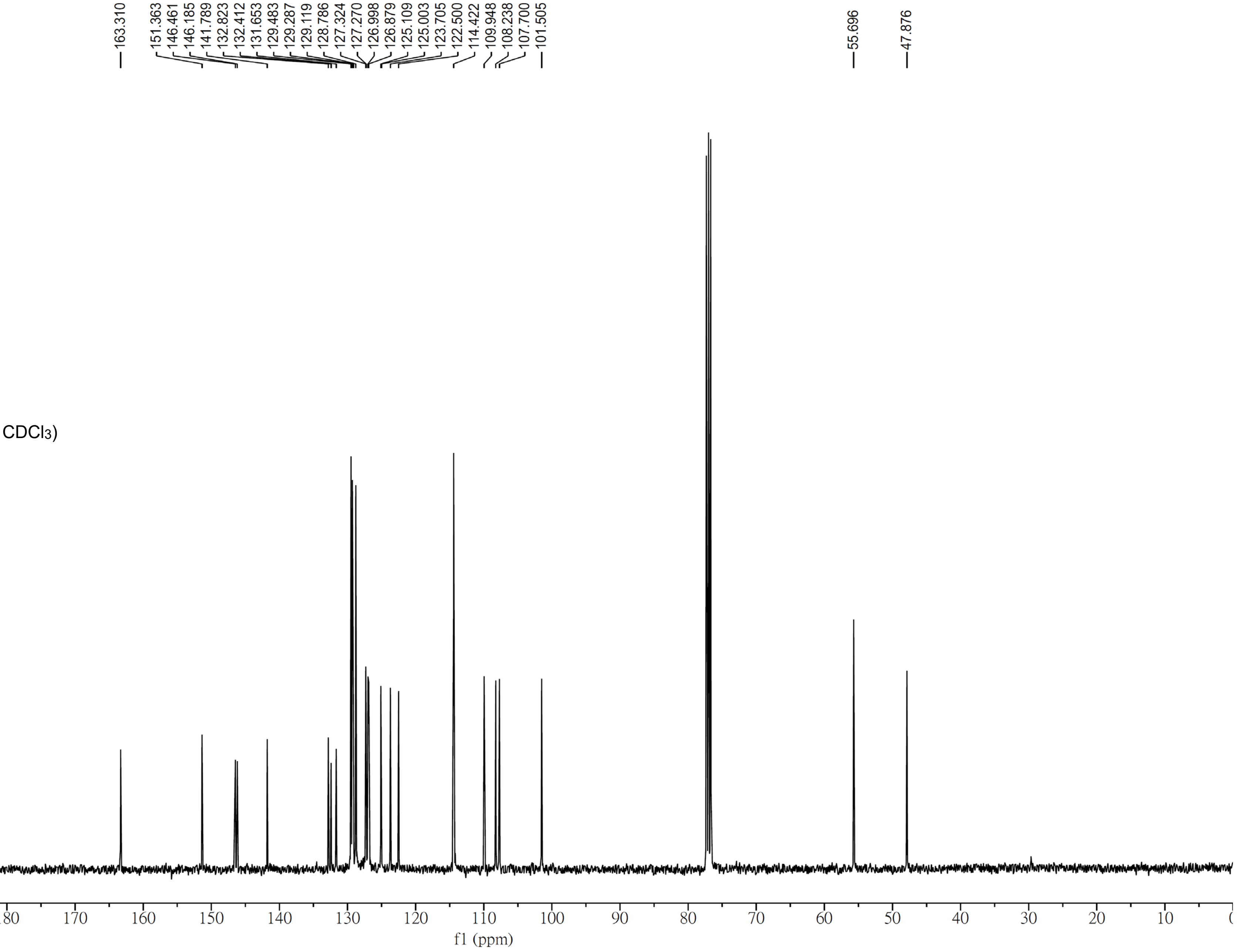

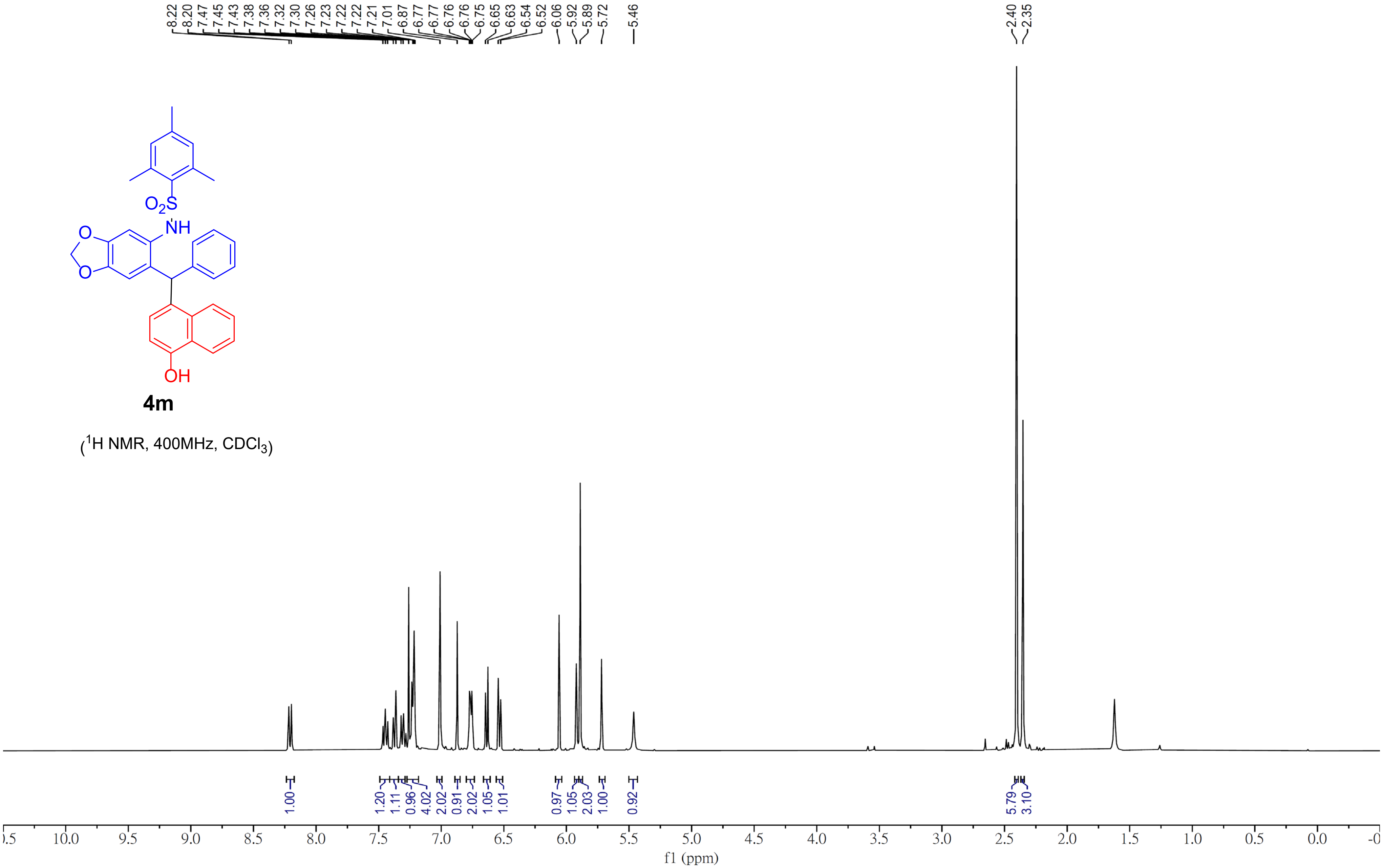

4m

(^{1}H NMR, 400MHz, $CDCl_3$)

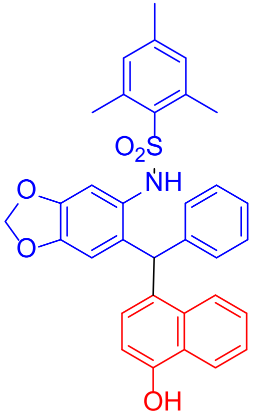

4m

($^{13}C\{^{1}H\}$ NMR, 101 MHz, $CDCl_3$)

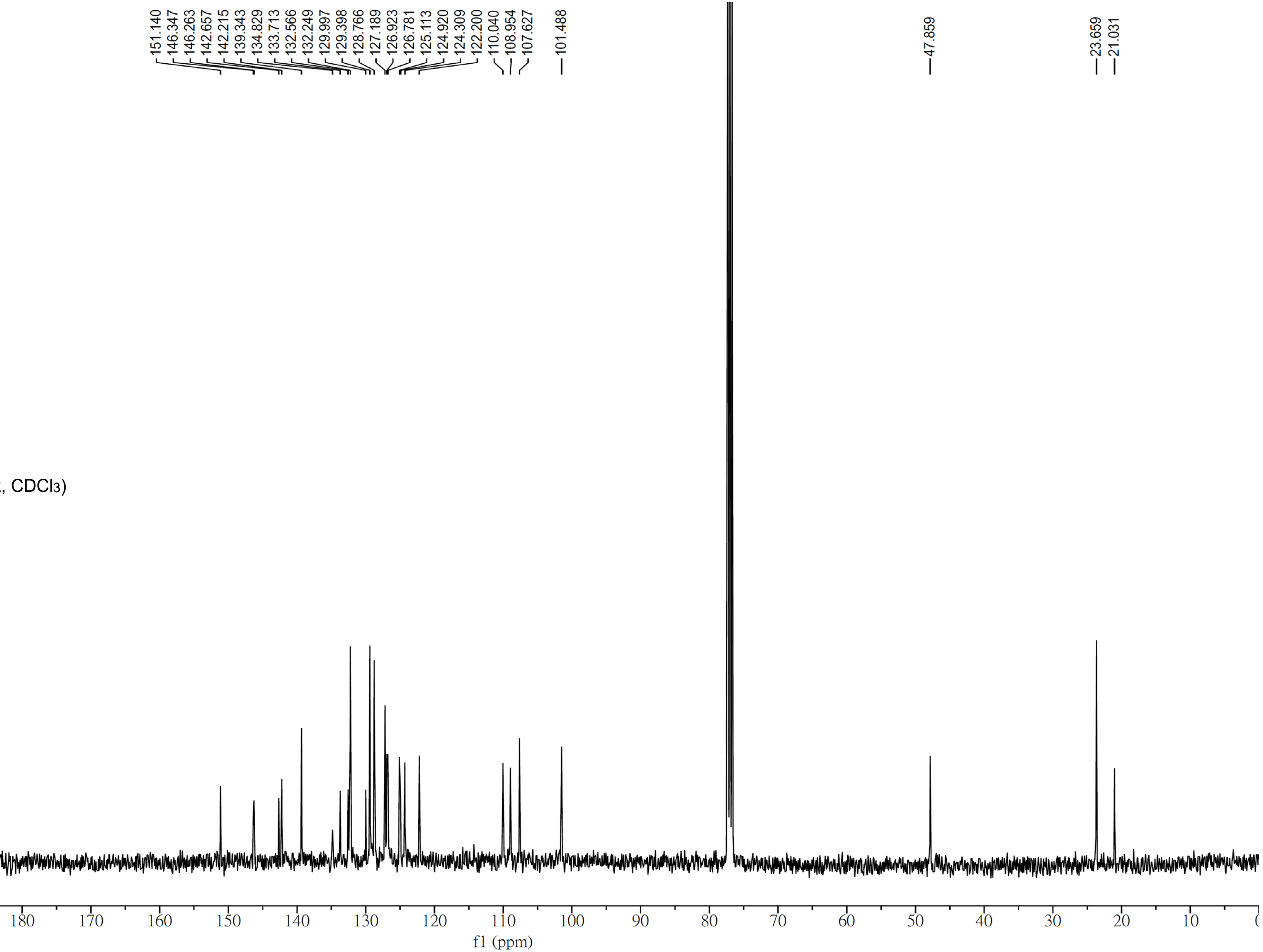

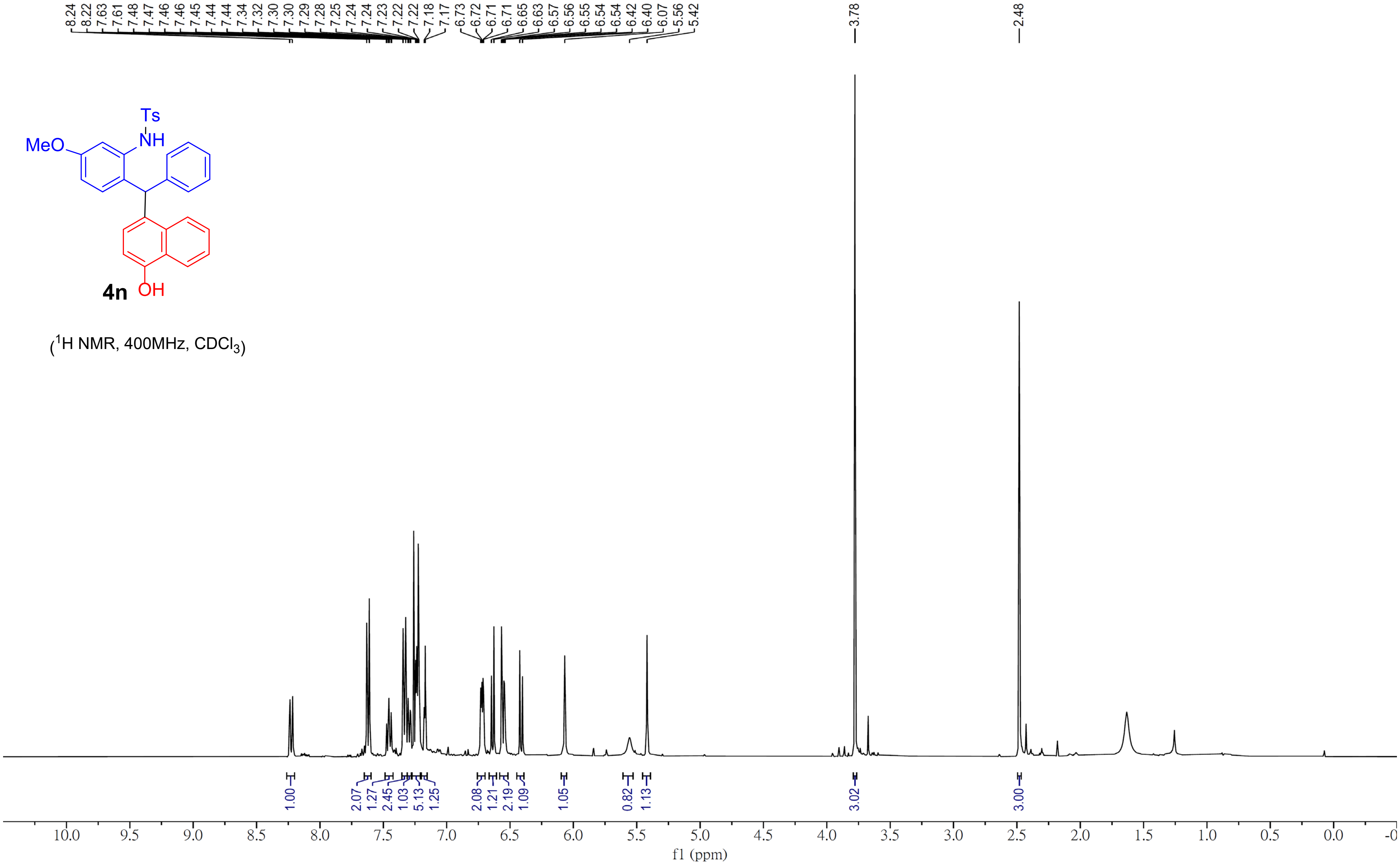
Ts
NH
MeO
OH
4n
(¹H NMR, 400MHz, CDCl₃)
8.24
8.22
7.63
7.61
7.48
7.47
7.46
7.46
7.45
7.44
7.44
7.34
7.32
7.30
7.29
7.28
7.25
7.24
7.24
7.23
7.22
7.22
7.18
7.17
6.73
6.72
6.71
6.71
6.65
6.63
6.57
6.56
6.55
6.54
6.54
6.42
6.40
6.07
5.56
5.42
3.78
2.48
1.00
2.07
1.27
2.45
1.03
5.13
1.25
2.08
1.21
2.19
1.09
1.05
0.82
1.13
3.02
3.00
f1 (ppm)

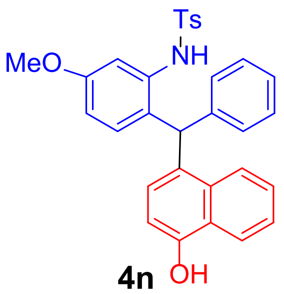

4n

($^{13}C\{^1H\}$ NMR, 101 MHz, $CDCl_3$)

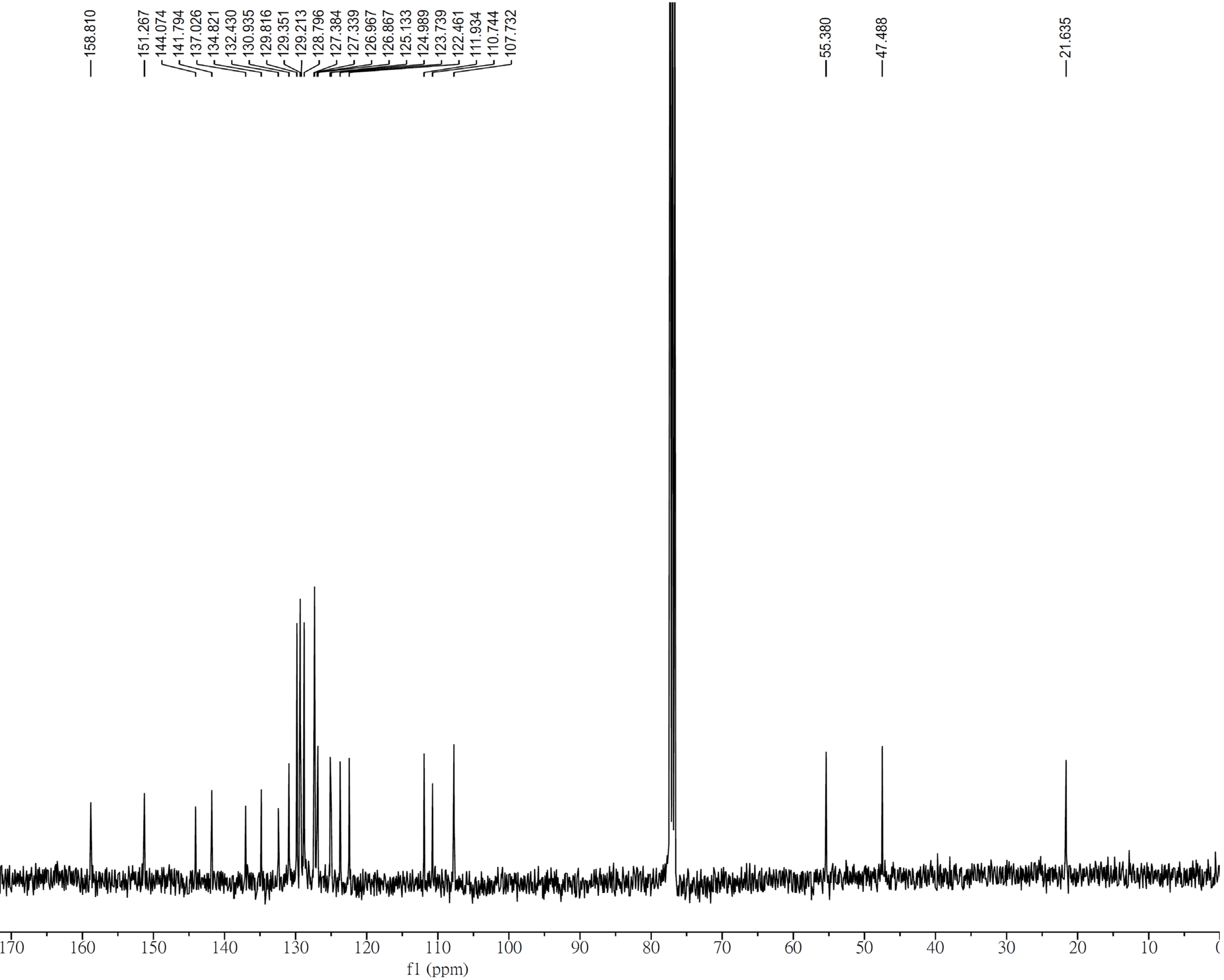

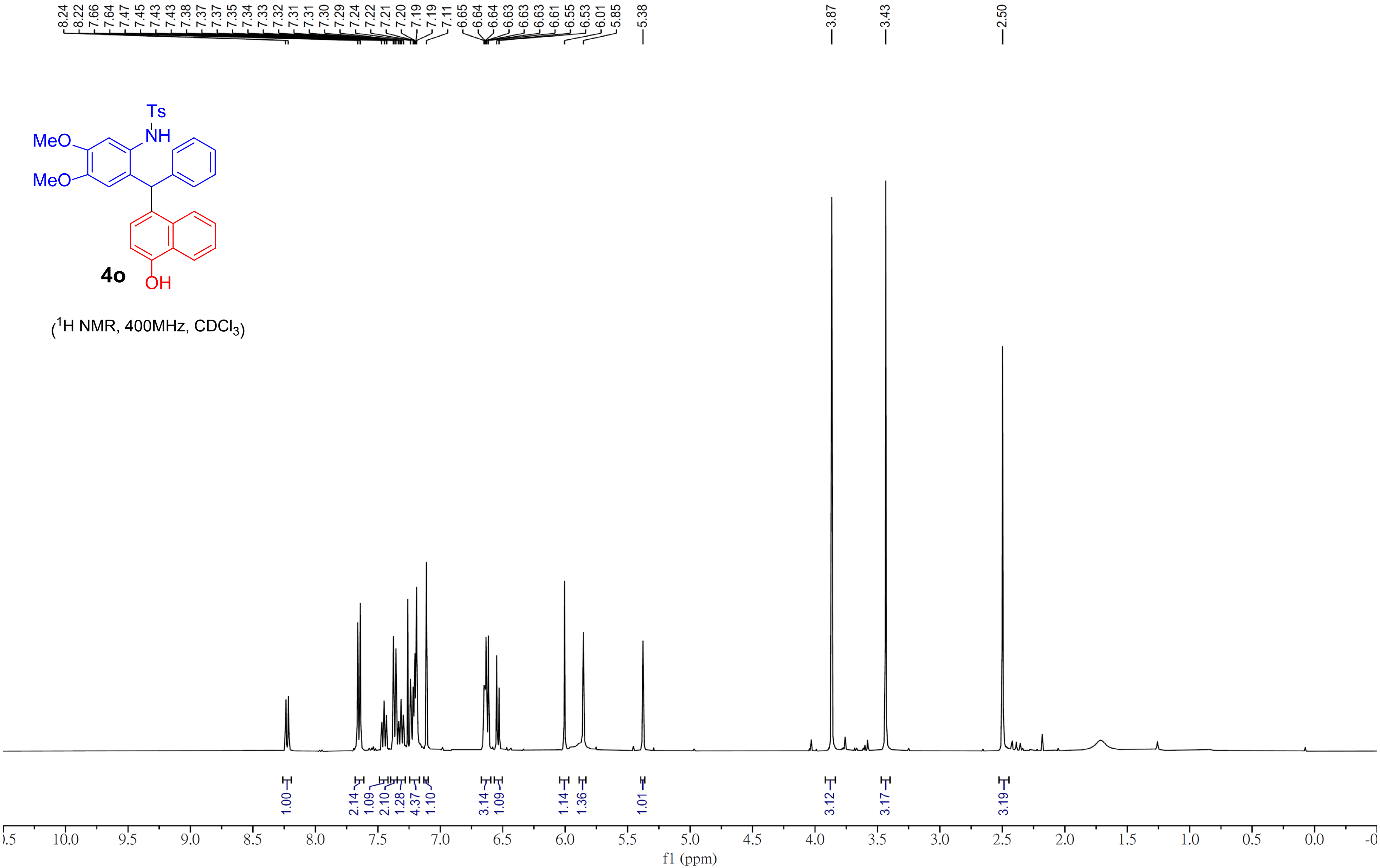

(^{1}H NMR, 400MHz, $CDCl_3$)

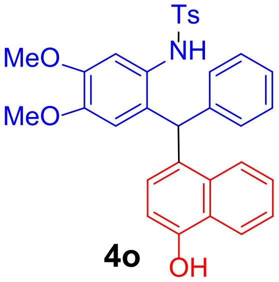

4o

($^{13}C\{^{1}H\}$ NMR, 101 MHz, $CDCl_3$)

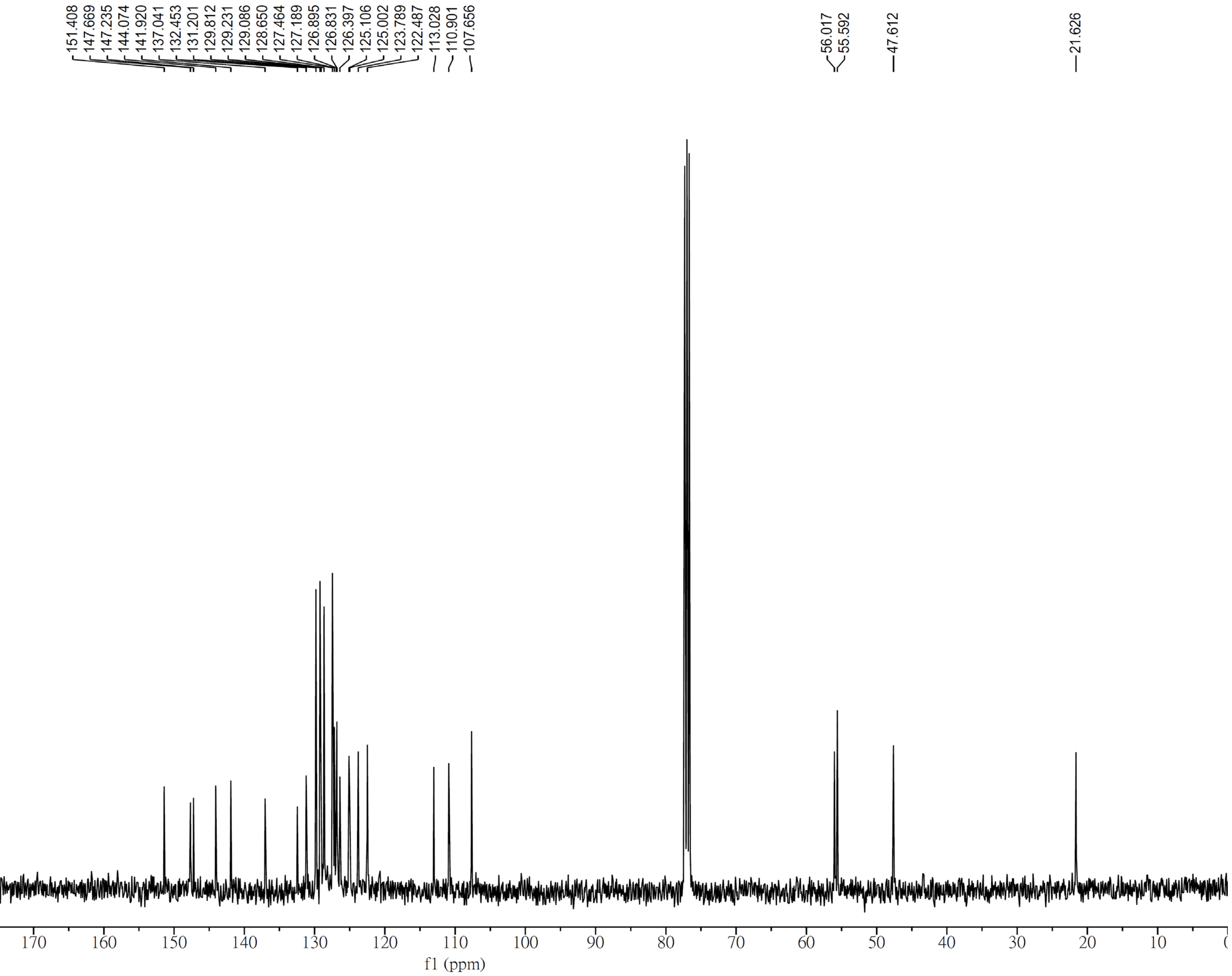

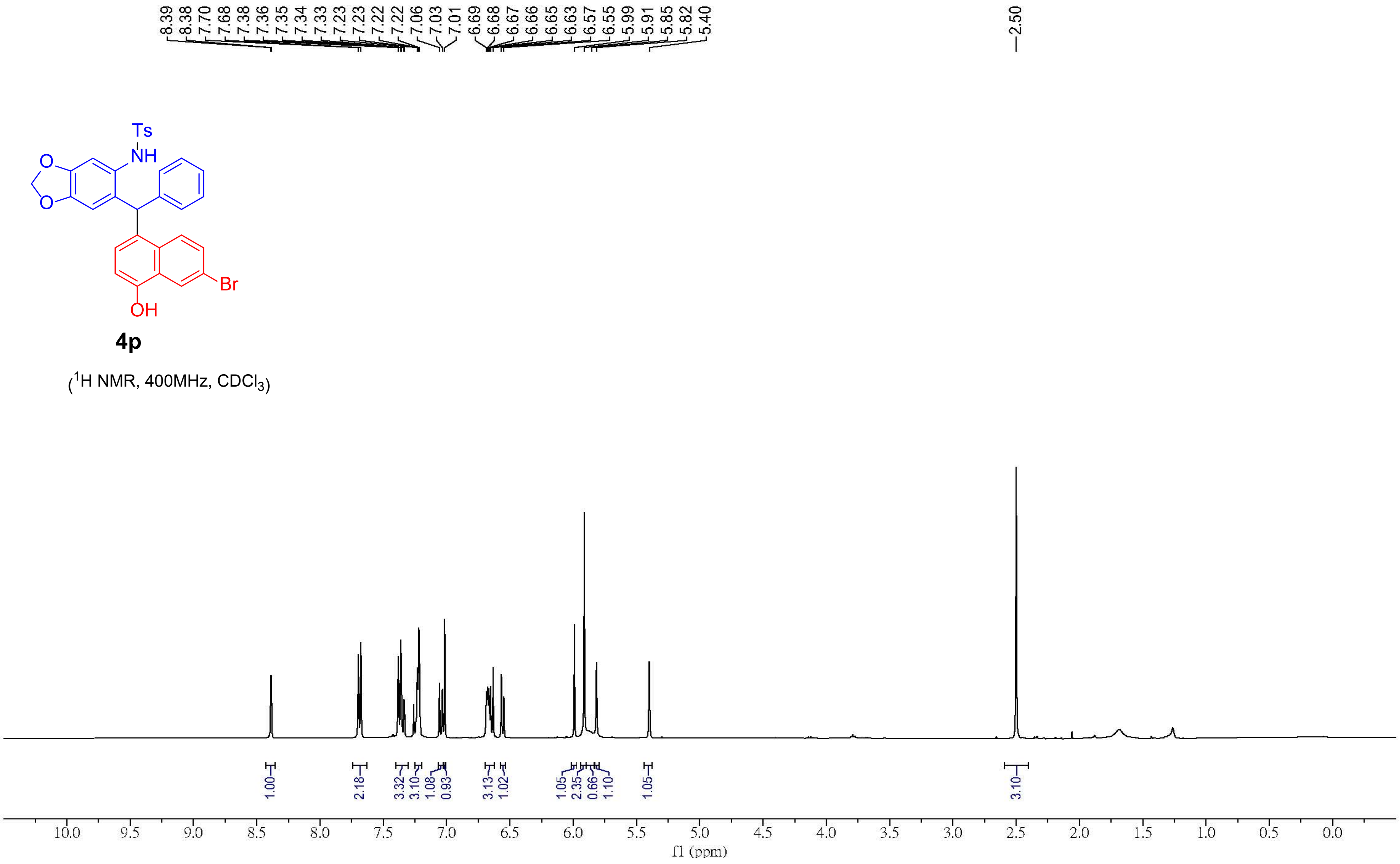

4p

(^{1}H NMR, 400MHz, $CDCl_3$)

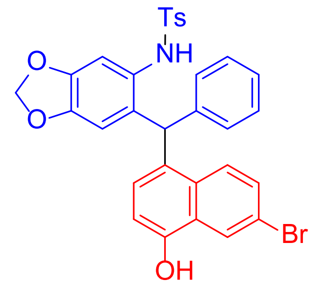

4p

($^{13}C\{^{1}H\}$ NMR, 101 MHz, $CDCl_3$)

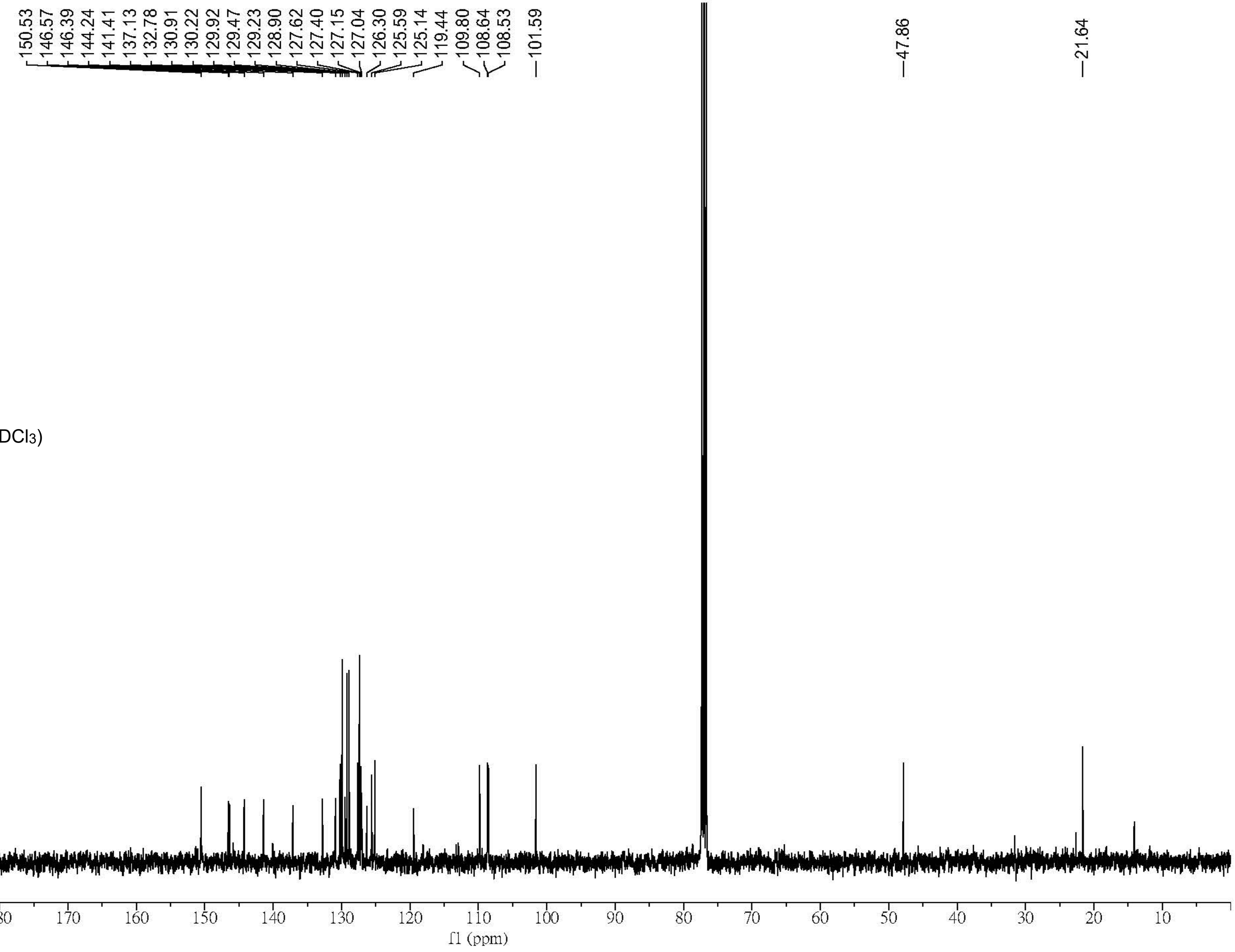

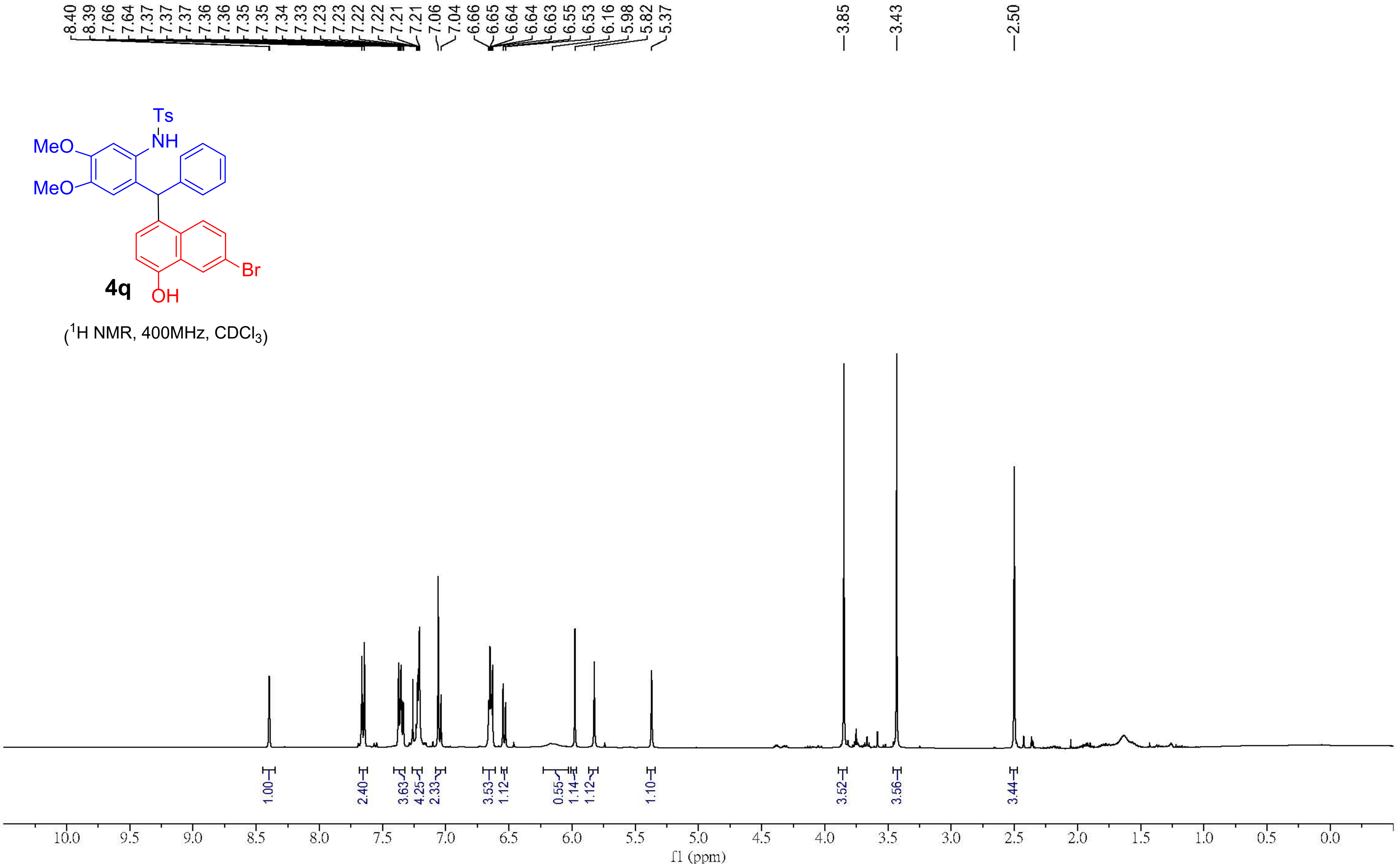
8.40
8.39
7.66
7.64
7.37
7.37
7.37
7.36
7.36
7.35
7.35
7.34
7.33
7.23
7.23
7.22
7.22
7.21
7.21
7.06
7.04
6.66
6.65
6.64
6.64
6.63
6.55
6.53
6.16
5.98
5.82
5.37
3.85
3.43
2.50
Ts
NH
MeO
MeO
Br
OH
4q
(1H NMR, 400MHz, CDCl3)
1.00
2.40
3.63
4.25
2.33
3.53
1.12
0.55
1.14
1.12
1.10
3.52
3.56
3.44
10.0
9.5
9.0
8.5
8.0
7.5
7.0
6.5
6.0
5.5
5.0
4.5
4.0
3.5
3.0
2.5
2.0
1.5
1.0
0.5
0.0
f1 (ppm)

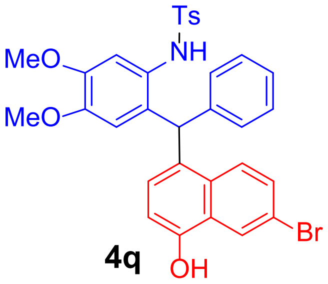

4q

($^{13}C\{^1H\}$ NMR, 101 MHz, $CDCl_3$)

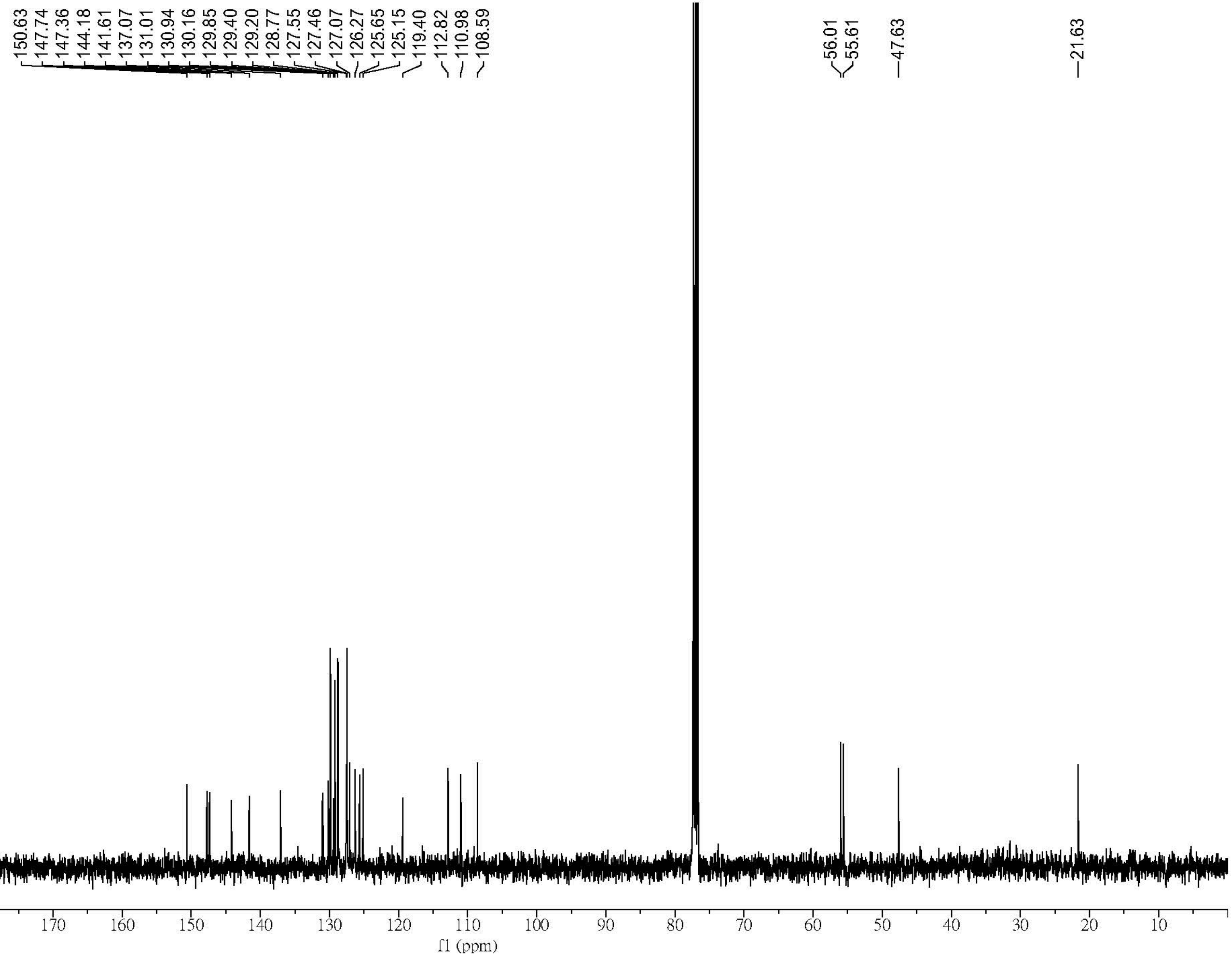

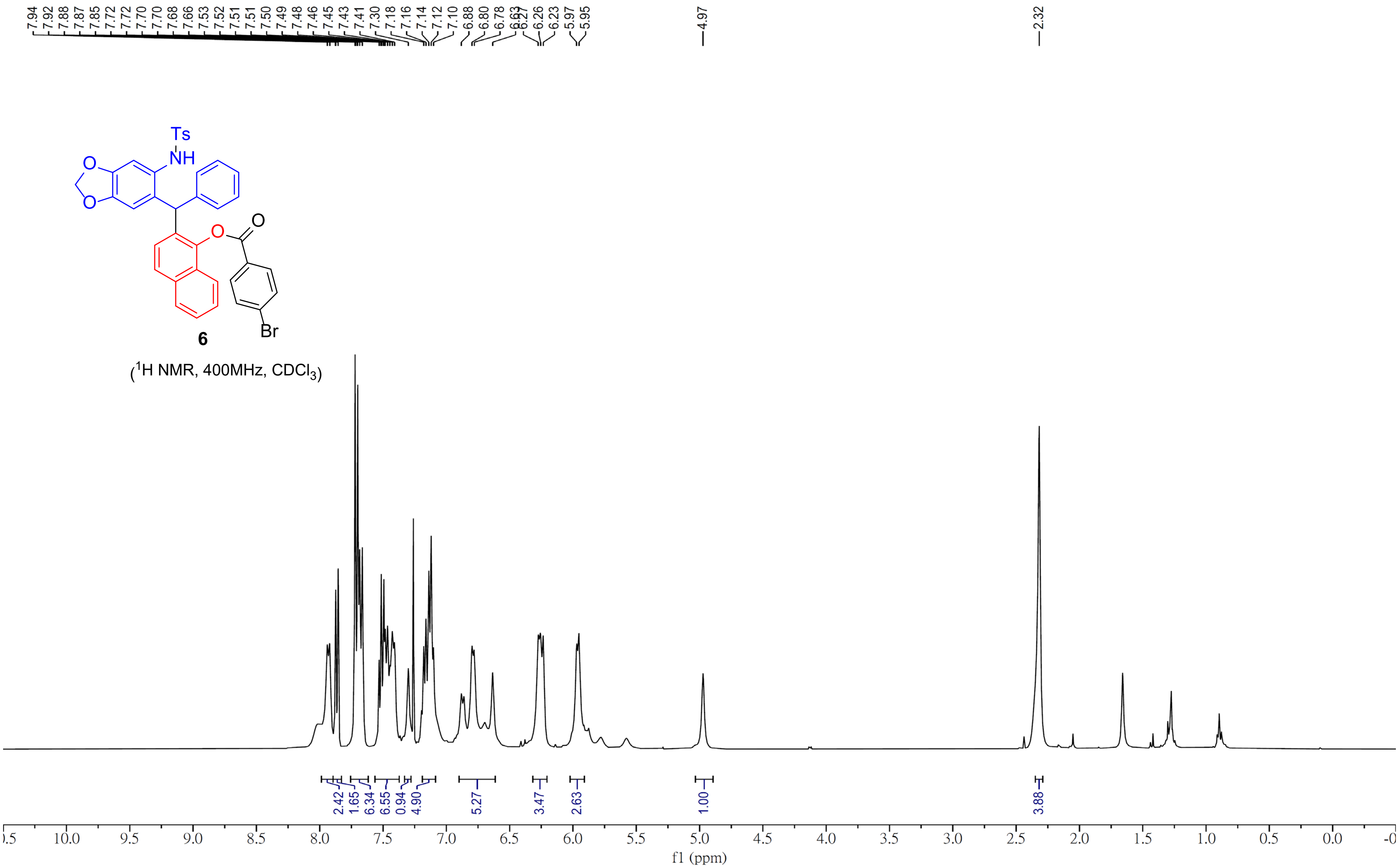

Ts
NH
O
O
O
O
Br
6
(1H NMR, 400MHz, CDCl3)
7.94
7.92
7.88
7.87
7.85
7.72
7.72
7.70
7.70
7.68
7.66
7.53
7.52
7.51
7.51
7.50
7.49
7.48
7.46
7.45
7.43
7.41
7.30
7.18
7.16
7.14
7.12
7.10
6.88
6.80
6.78
6.26
6.23
5.97
5.95
4.97
2.32
2.42
1.65
6.34
6.55
0.94
4.90
5.27
3.47
2.63
1.00
3.88
f1 (ppm)

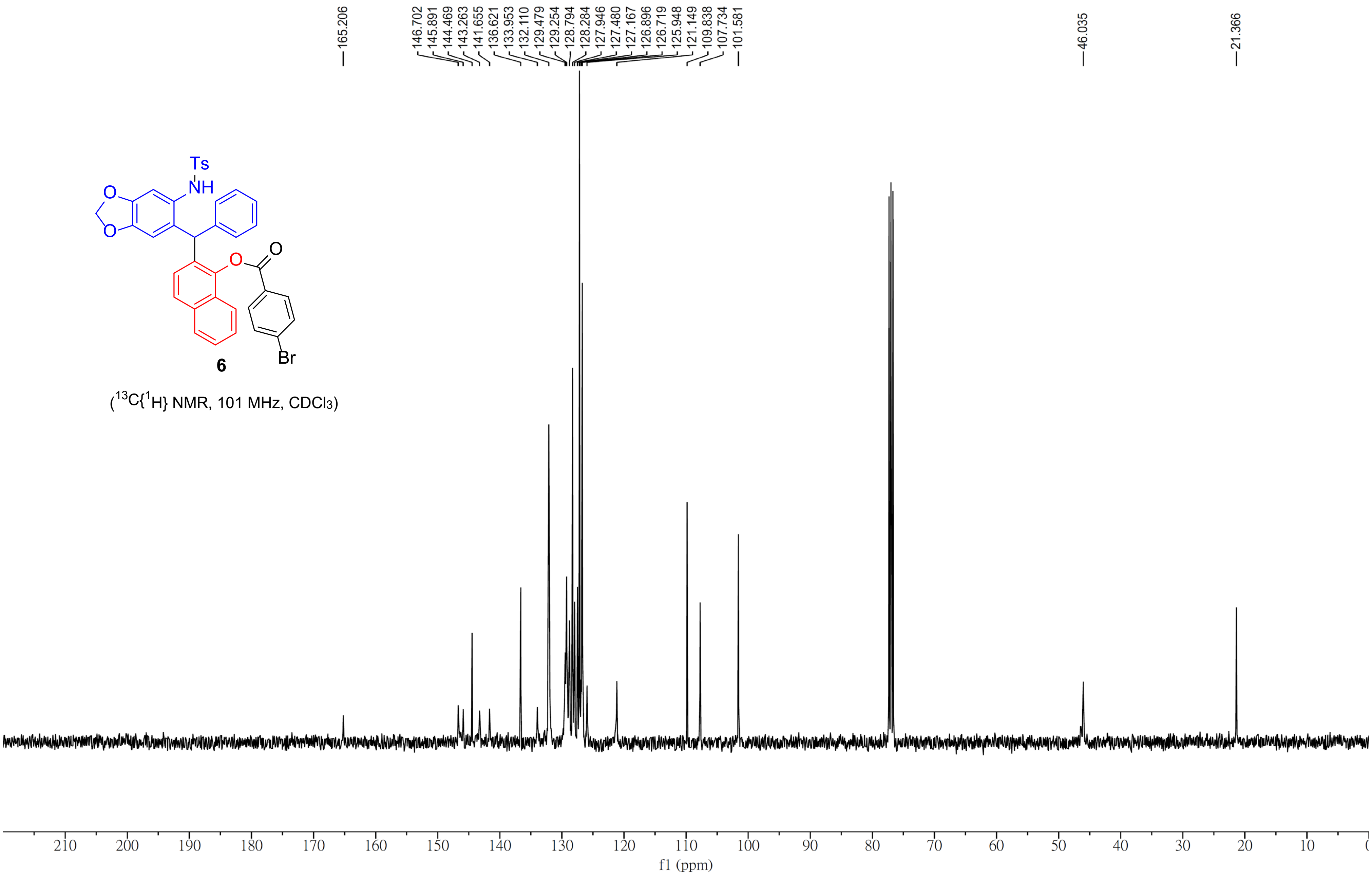

165.206
146.702
145.891
144.469
143.263
141.655
136.621
133.953
132.110
129.479
129.254
128.794
128.284
127.946
127.480
127.167
126.896
126.719
125.948
121.149
109.838
107.734
101.581
46.035
21.366
Ts
NH
O
O
O
O
Br
6
($^{13}C\{^{1}H\}$ NMR, 101 MHz, $CDCl_3$)
210
200
190
180
170
160
150
140
130
120
110
100
90
80
70
60
50
40
30
20
10
f1 (ppm)

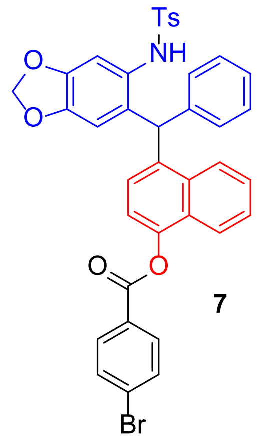

(^{1}H NMR, 400MHz, CDCl$_3$)

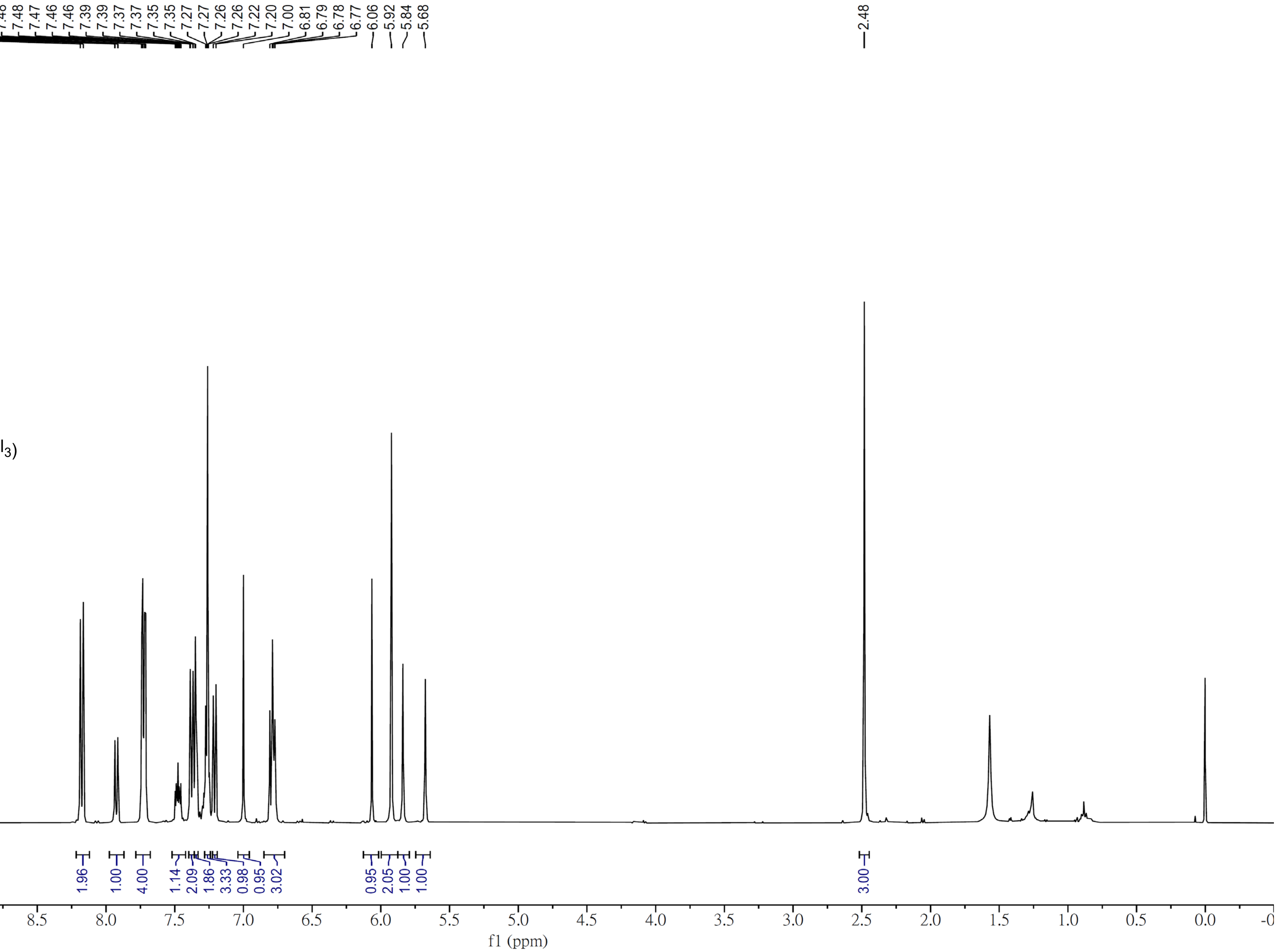

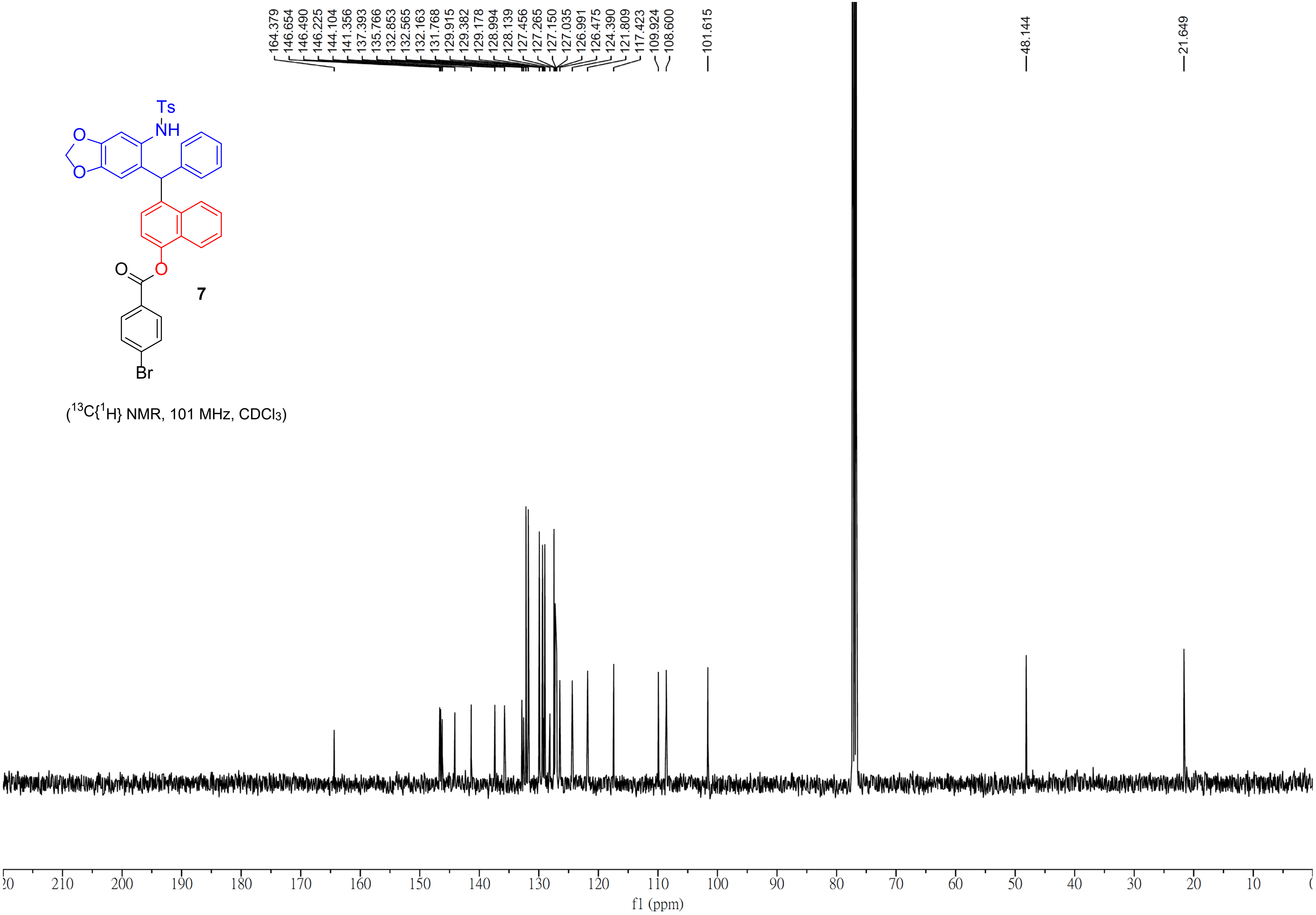

7

($^{13}C\{^{1}H\}$ NMR, 101 MHz, $CDCl_3$)
